# Modelling Skylarks (*Alauda arvensis*) to Predict Impacts of Changes in Land Management and Policy: Development and Testing of an Agent-Based Model

**DOI:** 10.1371/journal.pone.0065803

**Published:** 2013-06-06

**Authors:** Christopher J. Topping, Peter Odderskær, Johnny Kahlert

**Affiliations:** Department of Bioscience, University of Aarhus, Rønde, Denmark; Utrecht University, The Netherlands

## Abstract

Agent-based simulation models provide a viable approach for developing applied models of species and systems for predictive management. However, there has been some reluctance to use these models for policy applications due to complexity and the need for improved testing and communication of the models. We present the development and testing of a comprehensive model for Skylark (*Alauda arvensis*) in Danish agricultural landscapes. The model is part of the ALMaSS system, which considers not only individual skylarks, but also the detailed dynamic environment from which they obtain the information necessary to simulate their behaviour. Population responses emerge from individuals interacting with each other and the environment. Model development and testing was carried out using pattern-oriented modelling. The testing procedure was based on the model's ability to represent detailed real world patterns of distribution and density, reproductive performance and seasonal changes in territory numbers. Data to support this was collected over a 13-year period and comprised detailed field observations of breeding birds and intensive surveys. The model was able to recreate the real world data patterns accurately; it was also able to simultaneously fit a number of other secondary system properties which were not formally a part of the testing procedure. The correspondence of model output to real world data and sensitivity analysis are presented and discussed, and the model's description is provided in ODdox format (a formal description inter-linked to the program code). Detailed and stringent tests for model performance were carried out, and standardised model description and open access to the source code were provided to open development of the skylark model to others. Over and above documenting the utility of the model, this open process is essential to engender the user trust and ensure continued development of these comprehensive systems for applied purposes.

## Introduction

In western European countries, agricultural intensification over the latter half of the 20^th^ century has been strongly implicated in declines of farmland birds [1]–[3]. This intensification has involved many concurrent changes, but has largely manifested itself in higher yields as well as in a simplification of agricultural landscapes both in terms of crop diversity and landscape structure.

As a result of the Convention on Biological Diversity most countries, including the European Union, are committed to manage areas under agriculture in sustainable manner by 2020, ensuring conservation of biodiversity (CBD 2012). One of the focus groups comprises farmland birds. However, although there is strong evidence that agricultural intensity has played the major role in the farmland bird decline, it is not feasible to simply reverse this process. Nevertheless, a number of strategies are being implemented for mitigation of effects of agriculture. This includes national programmes, such as the UK Biodiversity Action Plans, which typically involve agri-environmental schemes with a variety of management options e.g. [4], [5]. However, due to the plethora of interacting drivers and the need for immediate action, there is a demand for a quick and flexible approach to assessing feasible options.

Agent-based simulation can cope with this complexity, at least if sufficient data is available to construct comprehensive models. However, there has been some reluctance to use this form of modelling, notably by policy makers. According to Smith *et al* there are three main objections to the use of models for policy development [6]. These are: that unbelievable results may arise from errors in the model; a lack of validation; and that a model may be thought to be “too complex”, or maybe “does not include some critical component”. To overcome these issues, it is necessary to improve communication and testing of complex models, making them if not more approachable, at least better understood and accepted by those capable of evaluating them.

This study addresses some of these concerns by documenting the development and detailed testing carried out for a skylark (*Alauda arvensis*) model. The skylark is an excellent example of a farmland bird species which has undergone widespread declines, is subject to conservation management plans, and for which the drivers of decline are complex [7]. Here we describe the extensive field data collected for the purpose of model evaluation and resulting successful real world emulation. The skylark model was developed within the Animal Landscape and Man Simulation System (ALMaSS) [8]. ALMaSS is a spatially and behaviourally explicit simulation model, incorporating dynamic modelling of the biotic and abiotic components of the organism's environment, together with agent-based models of the organisms themselves. The combination of detailed environmental simulation with a mechanistic agent-based modelling approach results in a system that is capable of handling at the population level the hitherto largely intractable problems associated with the spatio-temporal dynamics and non-equilibrium properties of the interactions between organisms and their environment. Models developed as part of this framework have previously been applied to risk assessment e.g. [9]–[11] as well as for population genetics [12], impact assessment e.g. [13], [14] and behavioural and landscape ecology e.g. [15]–[17].

In common with other models of this type, ALMaSS models tend to be large, parameter rich and data hungry. On the other hand they represent some of the most output-signal rich agent-based simulations available, capable of including highly detailed farm management, spatial structure and individual-based ecology. As a consequence, traditional approaches to model testing using uncertainty and sensitivity analysis are cumbersome, and recourse has to be made to other methods. One such method is pattern-oriented modelling (POM) [18]. POM refers to the multi-criteria design, selection, and calibration of models of complex systems [19]. This approach uses real-world data patterns that characterize the system of study to elucidate the mechanisms that create these patterns, and thus provide key elements in the model used to represent the system [18]. The approach taken to test and improve the model followed the post-hoc POM tests described by Topping *et al* [20], simultaneously develop and calibrate the model. Modifications made to the original version of the skylark model [11], [21] are detailed as the results of this testing procedure. The final model structure and documentation is the primary result of the present study, hence a comprehensive model description is provided as online supplementary material (see Supporting Information S1).

## Methods

All animal work was conducted according to relevant national and international guidelines. No permits were required for the described study, which complied with all relevant regulations, and no protected species were sampled. Permission was obtained from all landowners prior to carrying out this work.

### 3.1. ALMaSS

#### 3.1.1. Animal Models

All animals in ALMaSS are modelled using the agent-based approach, meaning that the individuals ‘sense’ information from their local environment and can act on it to make ‘decisions’. These decisions may be complex behaviour (e.g. finding a territory) or simple consequences (e.g. probability of nest destruction due to mowing). All animal modelling in ALMaSS is based on a state-machine concept, with states defined as time-variable behavioural or physiological states linked by condition-based transitions. Each agent has a set of behavioural rules defining the state-machine together with a set of interface functions that define its interactions with its environment. The resulting models are scaled to the information level available, with the aim of representing the current state of ecological knowledge for the species modelled as far as practicable.

An animal in ALMaSS is assigned to a life-stage associated with certain species and life-stage specific behaviours. These behaviours are described as a set of states and transitions, and each individual is described as a set of properties. As in the real world, conspecifics form part of the environment of any individual; hence social behaviour is an important component of the agent's rule base permitting the simulation of processes such as mate selection and territoriality. Since organisms are modelled at the individual level, and have individual attributes such as body size or condition, typical population model components such as vital rates must be implemented mechanistically, e.g. by linking mortality to local habitat factors such as agricultural operations. The resulting population dynamics, such as population fluctuations, are emergent properties of the sum of the organism's daily activity and their interactions with their local environment, rather than pre-programmed population-level behaviours. Hence, the model can also be used to predict how demographic parameters behave in transitory periods (e.g. climate change), or with other spatio-temporal factors such as predators.

#### 3.1.2. Landscape model

The landscape model in ALMaSS is an environment in which the modelled animals behave. What differentiates the ALMaSS landscape model from most other ecological simulation models is that here the environment is modelled in a highly dynamic way. The landscape model is based on a very detailed map of habitat types modelled at a resolution of 1 m^2^, allowing narrow linear habitats (e.g. field margins), to be simulated. The model is typically simulated in an area of 10×10 km and comprises a detailed reproduction with 35 landscape elements such as woodlands, hedgerows, field edges, fields, roads, buildings, lakes and streams and almost 70 vegetation types e.g. crop types and semi-natural vegetation types. All vegetation types in the landscape were subject to daily growth, which is a function of weather and management [22]. A weather simulator based on historical records is linked to the growth model. Farm management (and to a lesser extent other human activities e.g. mowing roadside verges, traffic) is modelled in detail. Each field in the landscape is associated with a farm based on management information obtained from EU subsidy claim information, and each farm has a specific farm type. Farms can select from over 50 crop variants to construct a crop rotation applied to the farm's fields. Each crop variant has a detailed management plan comprised of up to 50 different farming operations (e.g. sowing, harvesting, spraying, ploughing etc.). In order to create realistic management of fields, farming operations are made probabilistically dependent on weather, crop growth, soil type and previous farming; they also feedback to affect crop growth, weed biomass and insect biomass. Should detailed information be available, farms in the landscape can be simulated individually or otherwise as generally defined farm types (typically conventional pig farm, arable, dairy, or mixed, and organic variants of these). The landscape model is linked to the animal models by the presence or absence of farming operations and habitat variables such as vegetation height. For instance mechanical weeding may destroy skylark nests with a certain probability, or the lack of spraying results in fewer tramlines (tracks where the tractors drive through the crop) which provide useful skylark foraging habitat [23], [24]. It also removes plant biomass and thus affects the available insect food for the skylarks. Landscape heterogeneity is therefore controlled spatially by the topography and by cropping choices of the farmer, as well as temporally by weather, vegetation development and management.

For this study virtual skylarks were simulated in copies of two real farmland landscapes from Denmark, the area around Bjerringbro (56°22′39 N, 9°39′19 E), a 10×10 km area, and Kalø (56°17′58 N, 10°29′57 E), a 7×7 km area. The landscape model was provided by a digitisation from orthophotos and the national field block maps used at the time to administer EU arable area payment schemes. A field inspection was carried out to correct for possible errors and inconsistencies in the digital data. Further information on height and structure of individual hedgerows was also incorporated as attributes to the hedgerows was collected during field inspections.

#### 3.1.3. Overview of the skylark model

The final detailed model description is provided in Supporting Information S1, however a short overview is provided here to aid reading. The individual model skylarks are categorised as being members of five life-stages, clutch, nestlings, pre-fledglings, males, and females. The main drivers of the skylark model are the topography and habitat quality of the landscape elements being modelled, farming activities (crop choice, physical disturbance), crop growth, and weather. Available insect food biomass is determined by vegetation structure in each landscape element and type, see [25], and by its availability in terms of physical accessibility to the birds. It is updated daily in the model. During the breeding period, defined here as incubation and care of young up to 30 days old, the model considers the energetic balance of the adults, the food requirements for maintenance, requirements of young, and the weather constraints both as a limit to foraging success and as increased energetic costs for cold weather. The initiation of breeding depends upon firstly finding a suitable territory, and secondly, upon vegetation structure being suitable for a nest site. Breeding success depends on the habitat being able to fulfil the energetic requirements of the birds during the breeding period and the survival of eggs and nestlings. Birds may also be disturbed during nesting e.g. by farming activities, but this is rare during the breeding season.

### 3.2. Post-hoc Pattern-Oriented Model (POM) testing

Post-hoc POM development of a model is an iterative procedure comprising two basic phases, which are iterated until a satisfactory result has been achieved (see POM testing below for testing criteria used). The first phase is to contrast and test different potential sub-models or mechanisms; the second is to provide calibration and sensitivity analysis of the resultant model. Overall this approach is similar to “inverse modelling” or “Monte Carlo filtering” techniques used in other disciplines. Here we need to: 1) identify the mechanisms to be tested or the parameters to be calibrated; 2) observe the model in the same way as the real-world data was generated, for example using the approach of the virtual ecologist [26]; 3) finally run the model varying the parameter values in order to determine the best fit between the model output and real world patterns. If the fit reached is acceptable then the process stops, otherwise steps 1 to 3 are repeated. Whilst this approach for a single output is relatively weak in terms of significance of the resulting fit, the POM use of multiple pattern fitting based on the same model configuration rapidly reduces the possible parameter space. However, it is important to distinguish here between emergent patterns from the model and those that are pre-determined. POM testing is only concerned with patterns that emerge via interactions between model inputs and modelled mechanisms.

### 3.3. Data collection for POM-testing

**Figure 1:**
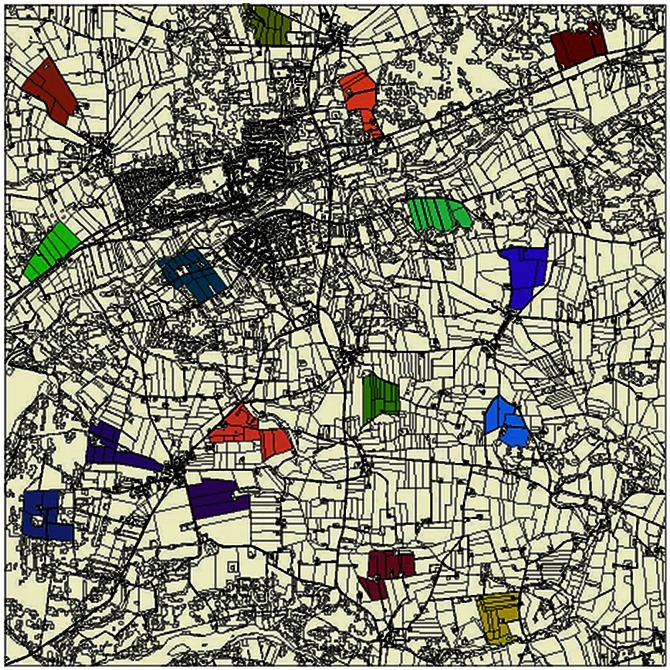
Map of the landscape around the town of Bjerringbro, showing the 16 study areas (shaded) in which data on skylark breeding densities were collected and virtual skylarks were sampled using ALMaSS.

A comprehensive data collection was undertaken for the POM procedure. In the Bjerringbro area, skylark territories were mapped eight times in 16 plots (each 35–59 ha) within a 10×10 km area during each of the three breeding seasons (mid-April to late July), 1998, 1999 and 2000 (Fig. 1). The mappings were carried out during favourable weather conditions and comprised data from 150 mainly conventionally farmed fields with an average size of 3.9 ha. Each mapping involved two observers who walked across fields in parallel at a distance of 25 to 100 m dependent on field topography, ensuring total coverage of individual fields. In order to locate skylarks accurately, markers were placed in the fields to provide smaller field units and reference points. Observers recorded mating status of the territory owner and breeding behaviours to establish the number of breeding pairs. If a territory covered fields outside the plots, the proportion of the territory that was inside the plot was assessed to the nearest 25%. The number of pairs recorded during the mappings in each breeding season defined the number of breeding pairs, which was then averaged across years and divided by plot size to establish overall pair densities in the 16 plots. Crops were recorded on each field for incorporation into the simulation of the farm types over the three study years.

In the Kalø area, the model predictions of the length of the incubation period (days), fate of non-hatched nests (predation, other mortality), the length of the period that chicks spent in the nest, referred to as the nestling period (days) and the fate of non-fledged chicks (predation, other mortality) were tested with field data from 1992–1995 and 2003–2005 from spring barley fields. During the first period, the area held a mixture of pesticide treated and untreated fields, whereas from 2004 and onwards all fields were organically farmed. In order to provide the necessary field data for the model testing, nests were located by means of observations of nest-building, flushing of incubating females or adults feeding the nestlings, obtained during systematic morning searches on foot or from a vehicle in potential breeding areas. When a nest was located, the position was drawn on a field map and a thin plastic rod was placed 12 m away from the nest in a selected compass direction in order to avoid predators using the rod as a food associated cue. The fate of the located nest, mainly found during egg-laying and incubation, was determined during daily visits until the end of the incubation period. Hatched clutches were checked regularly, see [27], until the end of the nestling period in order to record chick growth rates, and if the brood died, to determine the cause. Incomplete data or instances where predation or death was thought to be due to experimental disturbance were removed from the analysis. While the length of the incubation period was defined as the number of days from the first day of incubation to the day of hatching (both days included), the length of the nestling period comprised the number of days from hatching to the day when the chicks left the nest.

Data were also collected to describe the within field effect of providing skylark scrapes. Skylark scrapes comprised 100 unsown plots of an average of 40 m^2^ (Odderskær et al. 1997), placed in a 22 ha field of spring barley. The densities of breeding pairs were obtained from this field and a larger spring barley field (35 ha) without scrapes in the Kalø study area during 1991. Territory mapping followed the same protocol as in the Bjerringbro study site, except that they were carried out approximately weekly during the breeding season.

### 3.4. POM testing procedure

In order to simplify the POM procedure and description, we developed a set of three tests for fit against the three data sets constructed for this analysis. As in previous ALMaSS POM-testing [20], [28], [29], a summary statistic was created as the mean of the statistics used to test the fit for each individual pattern. This statistic was used to guide the fit since maximising fits to all patterns simultaneously may not be possible and trade-offs may be needed.

Because the data used for the patterns came from three separate real world data sets (Bjerringbro 1998–2000, Kalø 1992–1995 & 2003–2005, and Kalø 1991), pattern fitting required running three concurrent but independent simulations for each parameter set generated (see ‘General procedure for guided fitting’ below). For each set two overall measures of fit were created resulting in six statistics (Table 1), which were then scaled and combined in the overall fit statistic. The procedure for each set was:

**Table 1:** Overview of the fit statistics used to determine the fit to the observed real world pattern data.

Statistic Name	Pattern Set	Description
Bjerringbro (slope)	1	Bjerringbro landscape slope of the regression of model predicted skylark pairs against observed skylark pairs
Bjerringbro (R^2^)	1	Bjerringbro landscape r^2^ of the regression of model predicted skylark pairs against observed skylark pairs
Hatch Fit	2	Summed squared difference between real world and modelled proportions of incubation time (9–17 days), or otherwise the cause of death (predated or other mortality).
Nest Leaving Fit	2	Summed squared difference between real world and modelled nest leaving day (6–11 days), or otherwise the cause of death (predated or other mortality).
Density No Scrapes	3	Summed squared differences between real world skylark pair density in a large spring barley field *without* skylark scrapes and the model predicted density.
Density With Scrapes	3	Summed squared differences between real world skylark pair density in a large spring barley field *with* skylark scrapes and the model predicted density.

#### 3.4.1. Pattern Set 1: Bjerringbro landscape densities

A detailed copy of the 10×10 km landscape from which the skylark data was obtained was used as map input. Weather input was based on the years of sampling 1998–2000 from the Tranebjerg weather station (55°50′N, 10°37′E), and was looped repeating every three years. Farm management was based on a previous study from this area, and used farms classified as either pig farms, arable farms or mixed/hobby farms [30]. The 16 study areas from which skylark data was sampled were handled separately. For each of these areas the crops that were grown during the study period and their areas were used to create specific crop rotations. ALMaSS is not capable of recreating the exact pattern of crop/field usage for each year, but over a period of years the mean crop area can be made to match. To achieve this, the rotation is used on each field, and thus over a period of simulated years all fields will experience all crops in the correct proportion. The disadvantage here is that in the real world crops are not rotated round all fields because of soil, field size, aspect, or other constraints.

The simulation was run for 60 simulated years for each parameter combination tested. To avoid possible error due to a burn in period, only the last 20 years of simulation data were used to create 20-year means of the numbers of maximum number skylark pairs holding territories located with the centre inside any of the study areas.

The measure for goodness of fit were slope and coefficient of determination (R^2^) obtained by fitting a linear regression forced through the origin to a plot of densities predicted by the model against densities measured in the real world (n = 16). Both slope and R^2^ were given equal weight in the overall fitting. For slope, a fit statistic was used, calculated as the absolute values of (1-*b*)/*M* and (1-*M*), where *M* is the range over which the slope (*b*) varies in scenarios with stable populations. For R^2^ the same approach was used but M was the highest R^2^ observed. In both cases, the scaling in this way increased sensitivity to central (close to best fit) values and better described the range of possible outcomes when excluding extreme parameter value responses. In these and all other comparisons between model and observed data, the general expression for coefficient of determination was used to ensure a consistent measure of goodness of fit, given the fact that some comparisons were based on linear regression through the origin and others were direct comparisons of proportions or number of pairs (see pattern set 2 and 3). Thus, the coefficient of determination is expressed as R^2^ = 1−(SS_err_/SS_tot_), where SS_err_ is the sum of the squared residuals between observed and modelled data, and SS_tot_ is the sum of the squared difference between the observed data and the mean of the observed data.

#### 3.4.2 Pattern Set 2: Reproduction data (nest period)

To create model data comparable to the reproductive data collected in the field experiments, it was necessary to generate data from a spring barley crop to match the field conditions. This was achieved by using a landscape map from the Kalø area (in this case 7×7 km), but assuming that all crops were spring barley. Weather input was based on data from the Tranebjerg weather station from 1992 to 1995 and 2003 to 2005, to match the periods during which the field data was collected. The length of incubation for all clutches and their fate if not hatching (i.e. either predated or other mortality) was recorded for 20 years from a sixty year simulation. Similarly, from the same simulations, the length of time a nestling spent in the nest or its mortality cause was also recorded for the same period. These measurements were converted into two summary statistics describing the summed squared difference between real world and modelled proportions of incubation time (9–17 days), and nest leaving day (6–11 days), or otherwise the cause of death (predated or other mortality). The summary statistics were scaled to a 0–1 range to be comparable to the range in variation for Pattern Set 1.

#### 3.4.3. Pattern Set 3: Skylark scrapes

The impact of skylark scrapes was tested using data from two fields during 1991. These fields were mapped within the Kalø map used for Pattern Set 2, and the experiment with and without scrapes was reconstructed in ALMaSS using weather station data for 1991. As in the real experiment, the two fields were assumed to grow spring barley. In the first field, tramlines were kept open and skylark scrapes were provided, while in the other one, tramlines were allowed to close and no scrapes were provided. The simulations were run for 60 years and the last 20 years data used to calculate means for the number of breeding pairs in each of the two fields on the dates when the territories were mapped in the real world. This data was then compared to field data. Comparison statistics were mean squared differences between the two data sets for each field. To enable direct comparison with the other pattern fit statistics, the resulting values were scaled by the score given by an extinct population (the maximum negative deviation possible).

#### 3.4.4. General procedure for guided fitting

The procedure for guided parameter fitting followed the strategy adopted by Topping *et al.* [20]. This is an iterative procedure whereby the model parameters are adjusted *ad hoc* until a suitable fit is obtained to all pattern data, or if a fit is not considered possible, the model is altered structurally. In theory, determining the stopping rule (when a fit is accepted), should be a case of achieving a pre-defined minimum deviance between real world and model output. In practice, however, due to the use of an overall fit statistic, the stopping rule became the best fit that was achievable in terms of minimising the overall mean deviance. The resulting fit needed to be acceptable to the modeller and biologists involved, if not, the model was modified and the cycle restarted. Although this sounds somewhat imprecise, the overall aim is to maximise the fit as much as possible, and probably it is more conservative than accepting an arbitrary deviation.

In the iterations where a satisfactory fit was not achievable, the model structure itself was modified; the modifications being dependent upon an analysis of the cause of failure to fit. After modification the fitting procedure was repeated.

#### 3.4.5. Sensitivity analysis

Following the calibration and model development phases, a sensitivity analysis was carried out to determine which of the parameters varied in the fitting had the greatest impact on the results. All in all, 30 parameters were evaluated in this process (Table 2). Each was altered by ±5, 10, 20, and 40% either side of the chosen fitted parameter value, while keeping all other parameters at their fitted value. In the cases where parameters were small integers, these were varied by ±1, 2, 4, and 8. All six pattern-fit tests were carried out, and the six fit statistics and the overall fit statistic calculated and graphed (see Supporting Information S2). Those parameters that created no more than ±0.1 variation in the overall fit statistic (e.g. parameter SK_HQTALL, Supporting Information S2) were considered to be insensitive and were not considered further.

**Table 2:** Description of the input parameters modified during the pattern-oriented modelling procedures and subsequent sensitivity analyses with each parameter assigned to the biological mechanisms that they are related to: E = Energetics, R = Reproduction, M = Mortality and T = Territory quality.

Parameter Name	Major Mechanism	Parameter Description
EXTRACTION_RATE	E	Rate of food extraction per arbitrary unit food m^−2^ minute^−1^
HINDCONSTH_B	E	The linear rate of decrease of accessibility (expressed as 0 to 1.0) as a result of increasing height about a threshold (HEIGHTCONST_C)
HINDCONSTD_B	E	The linear rate of decrease of accessibility (expressed as 0 to 1.0) as a result of increasing height above a threshold (DENSITYCONST_C)
MAXFEEDRAIN	E	Precipitation level (mm day^−1^) at which foraging is assumed to be prevented.
PEMAX	E	Maximum growth rate of nestlings (g day^−1^).
RAINHINDPOW	E	The power of the curve relating feeding hindrance to rainfall.
TRAMLINE_FORAGING	E	Reduce hindrance proportion as a result of tramlines or skylark scrapes.
ADULTRETURNMORT	M	Mortality associated with overwintering and return to breeding area for adults.
CLUTCH_MORT_PROB	M	Daily probability of predation mortality for a clutch.
JUVRETURNMORT	M	Mortality associated with overwintering and return to breeding area for fledglings.
NEST_MORT_PROB	M	Daily probability of predation mortality for a nestling.
COOLING_RATE_EGGS	R	The cooling rate of eggs when not incubated (°C hr ^−1^ °C ^−1^)
EGGTEMP	R	Incubation temperature (°C).
MD_THRESHOLD	R	Threshold for physiological development of eggs (°C).
MINDAYSTOHATCH	R	Minimum incubation time assuming optimum incubating temperature (days).
NESTLEAVECHANCE	R	The probability of leaving the nest when reaching minimum nest leaving age.
TRIPLENGTH	R	The interval of time in minutes when a female is not incubating the clutch due to a feeding trip.
DENSITYCONST_B	T	Exponent for the reduction in habitat score due to increasing vegetation density for vegetation between 0.03 m and 1.1 m but above a threshold given by DENSITYCONST_C.
HEIGHTCONST_B	T	Exponent for the reduction in habitat score due to increasing vegetation height for vegetation between 0.03 m and 1.1 m above a threshold (HEIGHTCONST_C)
HQBAREEARTH	T	The quality value/m^2^ per square metre of habitat with vegetation below 3 cm tall.
HQHEDGE	T	The quality value/m^2^ associated with hedgerows, forests etc. (above 3 m or other tall objects).
HQTALL	T	Territory quality score/m^2^ for vegetation between >1.1 m and <2 m.
HQTALLVEG	T	The quality value/m^2^ associated with vegetation between 2 m and 3 m high.
MINFEMACCEPTSCORE	T	The minimum total habitat score before the female will accept a territory (used by both sexes).
PATCHYPREMIUM	T	The extra score/m^2^ for habitats assumed to be patchy and accessible to skylarks and less than 2 m tall.
SKSCRAPESPREMIUM	T	The extra score/m^2^ or fields with skylark scrapes.
TRAMLINEPREMIUM	T	The extra score/m^2^ or fields with open tramlines.
DENSITYCONST_C	T/E	Threshold for a reduction in quality due to increasing vegetation density for vegetation between 0.03 m and 1.1 m.
HEIGHTCONST_C	T/E	Threshold for a height mediated reduction in quality for vegetation between 0.03 m and 1.1 m.
TRAMLINE_DECAYTIME	T/E	The length of time the tramlines are assumed to be open in days.

#### 3.4.6. Secondary predictions

One of the best methods of evaluating model performance is the use of secondary predictions [31]. These are predictions made by the model which can be evaluated against real-world data, but which were not part of the original model fitting procedure. Whilst not considered as powerful as the detailed POM testing, they do provide further indications of the models performance.

In this case, secondary predictions were evaluated in three ways. Firstly, by carrying out further comparisons between fitted values of selected parameters obtained in the calibrated model and those found in the literature. This comprised parameters for incubation temperature and the threshold for physiological development of eggs, which were all obtained from a study on house sparrow [32]. Secondly, although it was not possible to extract within season variation in number of pairs for other fields, data did exist for the total number of territories in the field used for Pattern Set 2 without scrapes in 1991, but with scrapes being provided in 1992–1995. This provided the opportunity to test the prediction for number of pairs for this field but with scrapes. Finally, the length of time the female spends off the nest during incubation was recorded by additional field observations and compared to the fitted parameter value for the same behaviour.

#### 3.4.7. Feeding trip duration observations and analysis

In order to determine the number and duration of foraging trips, temperature loggers were placed under skylark nests found in two fields at the Kalø study area during incubation. Temperatures were logged every two minutes and recorded to the nearest 0.1°C. While the daily variation in temperatures was reflected in regular sine curves, the presence or absence of incubating females resulted in within day irregular patterns of temperature increases and drops of smaller magnitude than the daily pattern. When egg hatched and nestlings were present, the daily variation in temperature became less prominent.

In total seven nests were followed during incubation during the period 17 June to 1 August 2005. Temperature logging was only interrupted shortly by battery check and change. Two control loggers were placed at random near the nests and covered with a thin layer of soil (3 cm) to resemble the environment in a skylark nest as much as possible. As the micro-climate differed between locations and affected the local temperatures, absolute temperatures were not comparable, and hence the drops in temperatures were used in the determination of the time when females left the nest.

The analysis procedure was as follows: 1) Visual inspection of the temperature curves from each logger from skylark nests in order to determine the extent of the incubation period; 2) Determination of the duration of significant temperature drops in skylark nests defined as at least two consecutive two-minute periods with temperature drops of at least 0.2°C, excluding short temperature drops during a two-minute period e.g. due to egg-turning; 3) Determination of significant “natural” temperature drops during at least two consecutive two-minute periods recorded by the control loggers, using the same definition as for the “skylark-loggers”; 4) Calculation of the daily rate of significant temperature drops, broken into the periods of 4, 6, 8 etc. minutes; 5) Subtraction of the “natural” occurrence rate of temperature drops from the overall occurrence rate of temperature drops in order to determine the daily rate of female absence from the nest, assumed to be feeding trips; 6) Calculation of the arithmetic mean duration of the feeding trips based on the discrete distribution of feeding trips .

## Results

### 4.1. Results of POM testing

#### 4.1.1. Model modifications & final model description

The final model is definitively described by the ODdox documentation. ODdox is a method for providing both an overview of an individual-based model as well as access to the source code [29]. It is html-based and provides a useful alternative to long written documentation for larger models such as this. It also has the advantage that the reader can navigate through the program code. However, for ease of reading a short overview is provided in Supporting Information S1.

The overall model structure remains close to the original version from 2004 [21], but the POM testing altered a number of model implementation details in order either to simplify the model, or to improve the model performance (see Supporting Information S3).

#### 4.1.2. Final Fits to Pattern Sets

**Figure 2:**
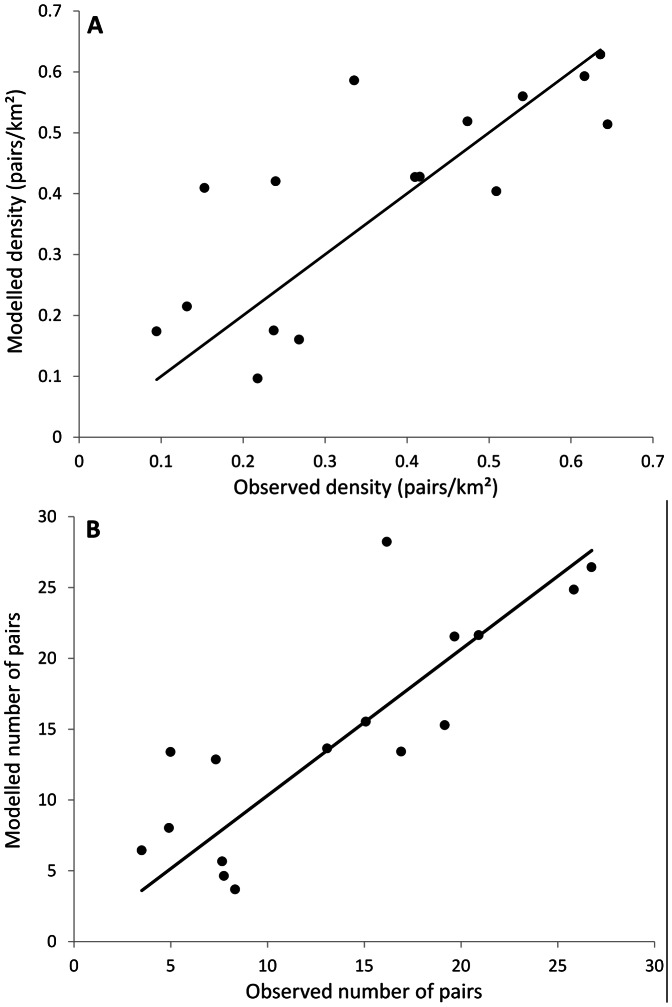
The relationship between the modelled mean densities (A) and pairs (B) of skylarks in the 16 study areas in Bjerringbro derived from the ALMaSS skylark model and the mean observed densities (A) and pairs (B) obtained from surveys carried out 1998 to 2000. * is Farm 14 referred to in the text.

Naturally it was not possible to precisely fit all the POM testing patterns, however, acceptable fits were obtained for all patterns used. The overall best fit model and parameterisation produced a good fit to the predicted densities of skylarks in the Bjerringbro study area. A linear regression, of the modelled within season maximum territory density against the same metric measured from the field had a slope of 1.00 (R^2^ = 0.553, n = 16) (Fig. 2A). Using actual numbers of territories the slope was comparable (1.03), however, the fit increased slightly (Fig. 2B) (R^2^ = 0.622), suggesting that the major determinant of numbers was not simply area. This was confirmed by regressing observed number of pairs against area, which resulted in a significant (t = 8.20, P<0.0001), but poor relationship (R^2^ = 0.221). There was, however, one clear outlier in the data, representing farm 14. This farm was also the farm with the largest field sizes and largest area at 48 ha, with only three fields.

**Figure 3:**
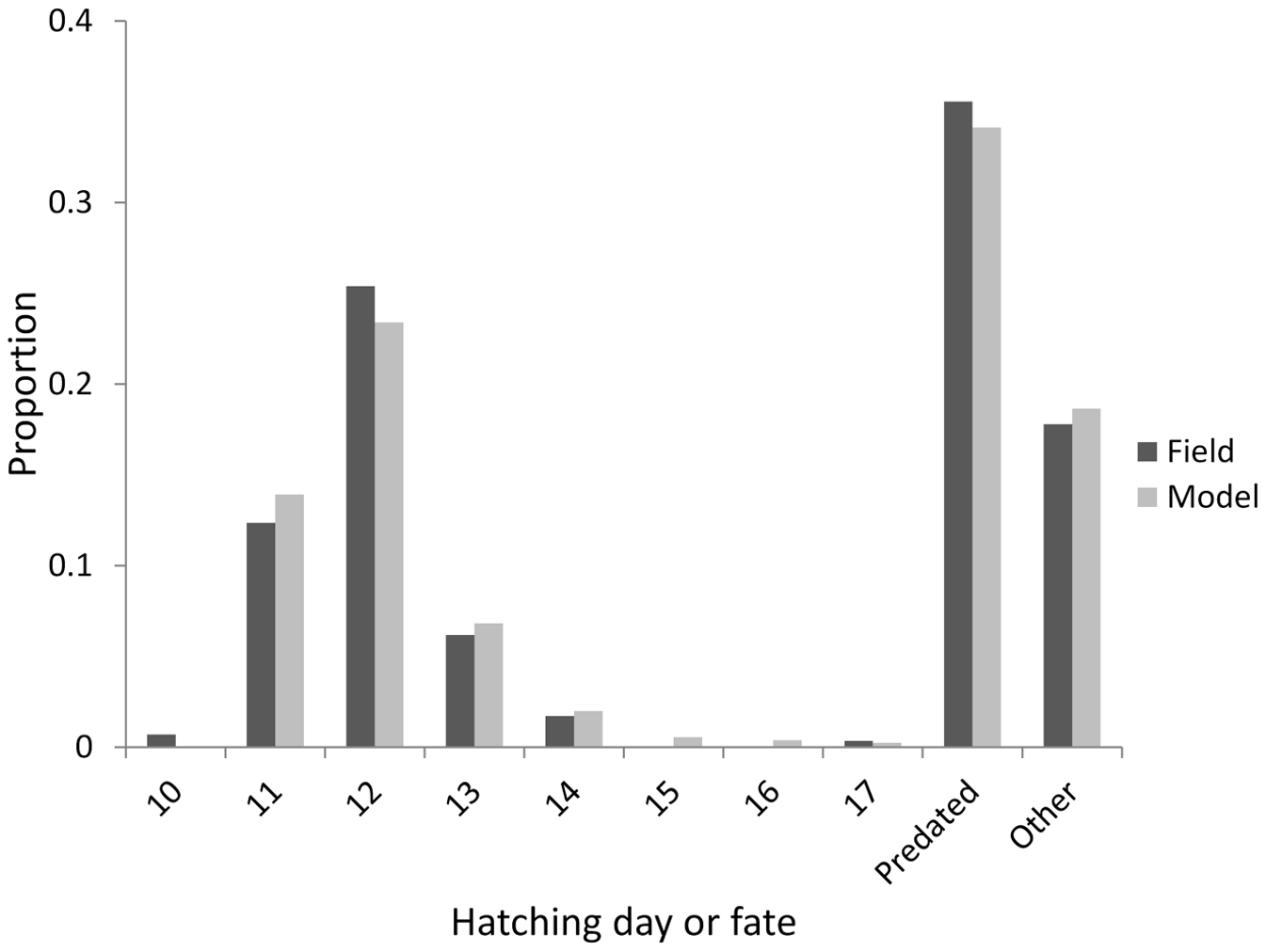
Comparison of the hatching day of successful clutches (minimum one egg hatched), and causes of nest failure observed in the Kalø study area and those predicted by the ALMaSS skylark model.

An excellent fit (R^2^ = 0.993) was obtained to the distribution of egg hatching times, and the associated mortality if not hatching (Fig. 3). Hatch date distribution and mortality causes were accurately predicted by the model, although it was not possible to maintain this fit and have any model birds hatching on day 10.

**Figure 4:**
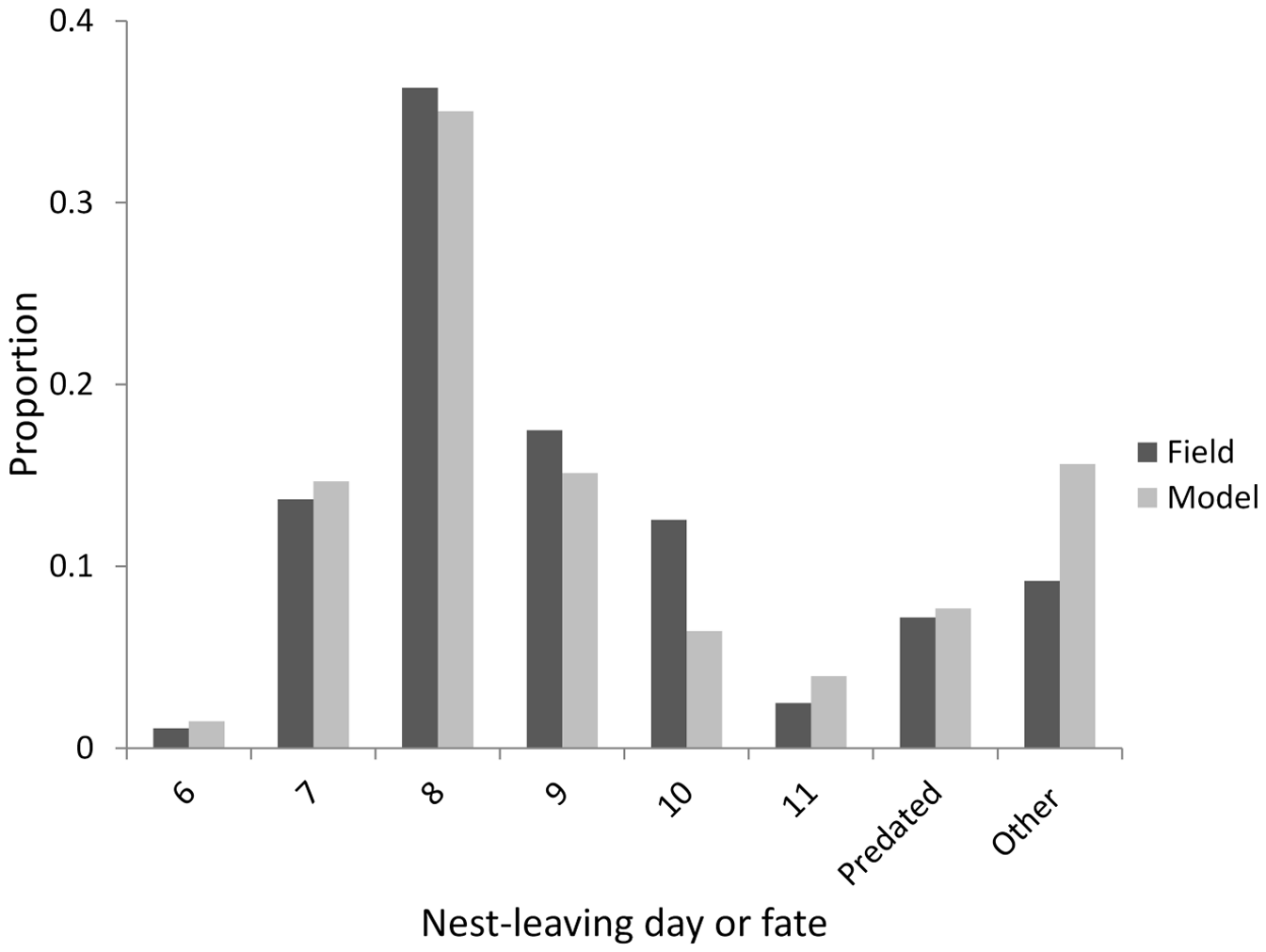
Comparison of the nest-leaving day of fledglings and causes of nestling mortality observed in the Kalø study area and those predicted by the ALMaSS skylark model.

Fits to the nest leaving date were also good (R^2^ = 0.896), but not as close as egg hatch day (Fig. 4). In particular, it was difficult to match mortality caused by other factors with the best fit resulting in an over-estimate of nestling mortality of 6.4%.

**Figure 5:**
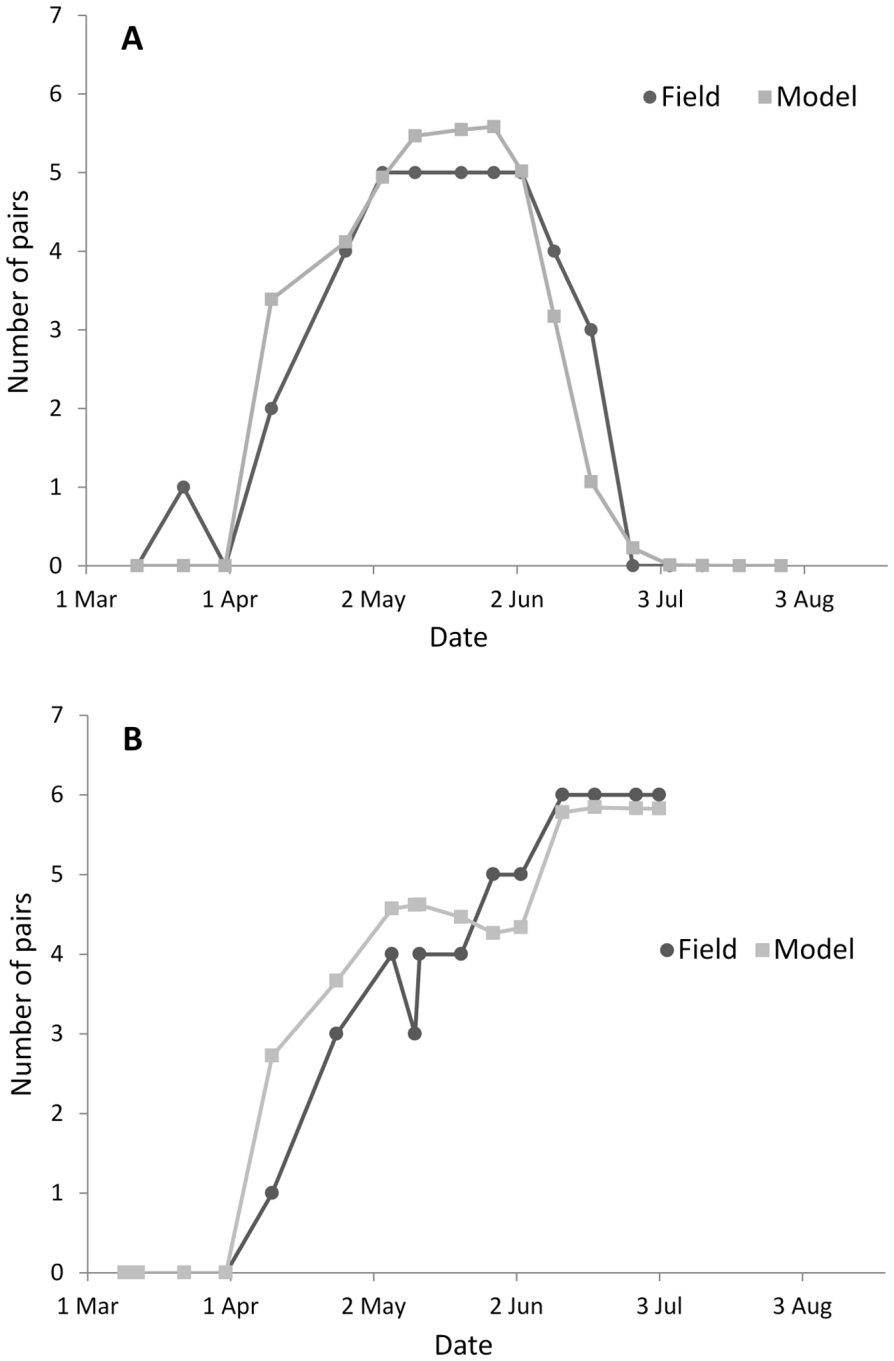
Comparison of the seasonal distribution of the number of territorial pairs observed in the Kalø study area on fields without (A) and with (B) scrapes, and those predicted by the ALMaSS skylark model.

Fits to the within season patterns for the two Kalø fields with and without scrapes were surprisingly good (R2 = 0.906 and 0.898, respectively) considering that the number of pairs present were low (5 & 6), and hence variation of only one pair would have altered the fit dramatically. Without scrapes the model birds increased in numbers between day March 16^th^ and May 2^nd^, remained constant until day June 2^nd^, then began to give up their territories until day June 24^th^ (Fig. 5A). With scrapes the pattern was similar up to day May 2^nd^, at which point there was another increase in pair numbers, which was maintained until the end of the observations on day July 3^rd^ (Fig. 5B). In this case, model predictions tended to overestimate numbers in early season.

#### 4.1.3. Sensitivity Analysis

Of the 29 parameters tested for responses to all six patterns, 13 were found to be relatively insensitive (mean response deviation less than 0.1). Of the 16 remaining parameters, 13 had overall fit statistic responses between 0.1 & 0.4, the remaining three parameters were more sensitive with fit statistic values of over 0.5 (Table 3).

**Table 3:** Input parameters modified during the pattern-oriented modelling procedures and the subsequent fitted values, sensitivity analyses with the overall fit statistic, and the biological mechanisms that each parameter is related to: E = Energetics, R = reproduction, M = Mortality and T = Territory quality.

Parameter Name	Value	Maximum Overall Fit Deviation	Major Mechanism
PEMAX	4.54	0.54	E
MINDAYSTOHATCH	10.2	0.53	R
DENSITYCONST_C	10	0.32	T/E
MINFEMACCEPTSCORE	300000	0.26	T
MD_THRESHOLD	25.8	0.25	R
EXTRACTION_RATE	0.00053	0.23	E
ADULTRETURNMORT	35	0.22	M
EGGTEMP	36.1	0.22	R
JUVRETURNMORT	35.0	0.20	M
RAINHINDPOW	4	0.20	E
MAXFEEDRAIN	4.7	0.18	E
CLUTCH_MORT_PROB	350	0.16	M
TRAMLINEPREMIUM	6	0.14	T
HEIGHTCONST_C	38.0	0.11	T/E
TRIPLENGTH	10.5	0.11	R
COOLING_RATE_EGGS	3	0.09	R
HEIGHTCONST_B	−0.22	0.06	T
PATCHYPREMIUM	47	0.06	T
NESTLEAVECHANCE	23	0.05	R
HQBAREEARTH	3	0.05	T
NEST_MORT_PROB	23	0.05	M
DENSITYCONST_B	−0.26	0.05	T
HQHEDGE	−250	0.04	T
HINDCONSTH_B	−0.025	0.04	E
HINDCONSTD_B	−0.22	0.04	E
HQTALL	−2	0.04	E
HQTALLVEG	−10	0.04	T
TRAMLINE_FORAGING	0.45	0.04	T
TRAMLINE_DECAYTIME	21	0.04	T/E
SKSCRAPESPREMIUM	0.24	NA	T

The parameters are ordered from the largest to the smallest impact in the sensitivity analysis. Parameter-specific value precision indicates the precision of variability considered during parameter fitting.

**Figure 6:**
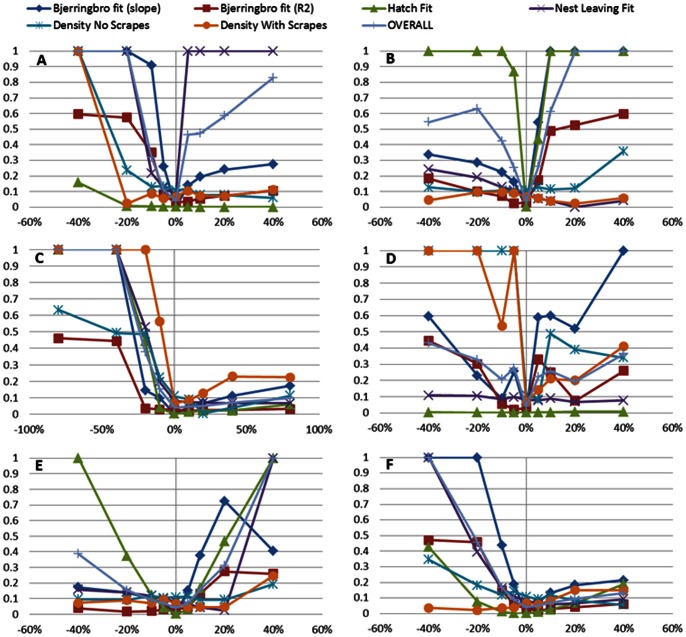
Parameter sensitivity for the six most sensitive parameters using 7 fit statistics. Each panel represents the deviation in model output relative to field data for particular parameters. Each point represents the deviation of model output from the corresponding field data pattern averaged across replicates. Each line in each panel represents the response to variation (±5, 10, 20, and 40%) in one particular parameter; hence lines of the same colour come from the same simulations and can be directly compared between panels. The parameters presented are: maximum daily growth rate of nestlings *PEMAX* (A), minimum incubation time *MINDAYSTOHATCH* (B), threshold for reduction in habitat quality due to increasing vegetation density *DENSITYCONST_C* (C), minimum habitat score for female acceptance of a territory *MINFEMACCEPTSCORE* (D), the threshold for physiological development of eggs *MD_THRESHOLD* (E), and food extraction rate *EXTRACTION_RATE* (F).

Model responses were not universally sensitive to all parameters, but the method of scaling the results to the overall parameter variability results in all response variables being sensitive to at least one parameter. This does not, however, result in high overall sensitivity scores for a parameter if other response variables are insensitive. Examples of these parameters are *HQBAREEARTH* and *PATCHYPREMIUM*, which both affect the slope of the Bjerringbro pair density regression, but have minimal impacts on other response variables (Supporting Information S2). Thus, parameters listed as sensitive affect multiple response variables. The six most sensitive parameters produce complex response patterns across the size response variables (Fig. 6). Slope and R^2^ of the Bjerringbro regression are the only response variables that vary over the full range for all six parameters. No response variables showed monotonic trends, but two parameters (*DENSITYCONST_C* Fig. 6C and *EXTRACTION_RATE* Fig. 6F) are close to having a threshold response with increasing values above the fitted point affecting the response variables only slightly, but decreasing values result in strong response variable changes. Most other response patterns are roughly U-shaped indicating that we have an optimum fit around the central parameter value. However, responses due to changes in *MINFEMACCEPTSCORE* (Fig. 6D) are more complex, with no clear pattern for the Bjerringbro R^2^ and ‘Density No Scrapes’ for values above the fitted minimum, but with significant variation. The overall feature of this model is therefore that one of interacting parameters produces complex emergent responses.

Two parameters control the impact of skylark scrapes, *SKSCRAPESPREMIUMNEST* and *TRAMLINE_FORAGING*. The latter also affected other response variables, whereas the former could only affect patterns measured where scrapes were used, i.e. ‘Density with Scrapes’, and hence was not evaluated using all seven patterns.

### 4.2. Secondary Predictions

#### 4.2.1. Fit to literature parameter values

Two parameters were available from the literature, although not directly for skylarks, and thus were allowed to vary freely. The final values used were close to literature values in all cases: *EGG_TEMP*, 36.1°C [32], fitted 35.0; *MD_THRESHOLD*, 26°C [33], fitted 25.8°C. Whilst this is not a strong test, *EGG_TEMP* and *MD_THRESHOLD* were both found to be sensitive parameters, and hence the fitted values are not arbitrary. Both parameters emerge from interactions between environmental temperature, female incubation activity, and energetic mechanisms controlling egg development resulting in the patterns of egg hatch fitted by the POM fitting procedure.

#### 4.2.2. Feeding trip duration

**Figure 7:**
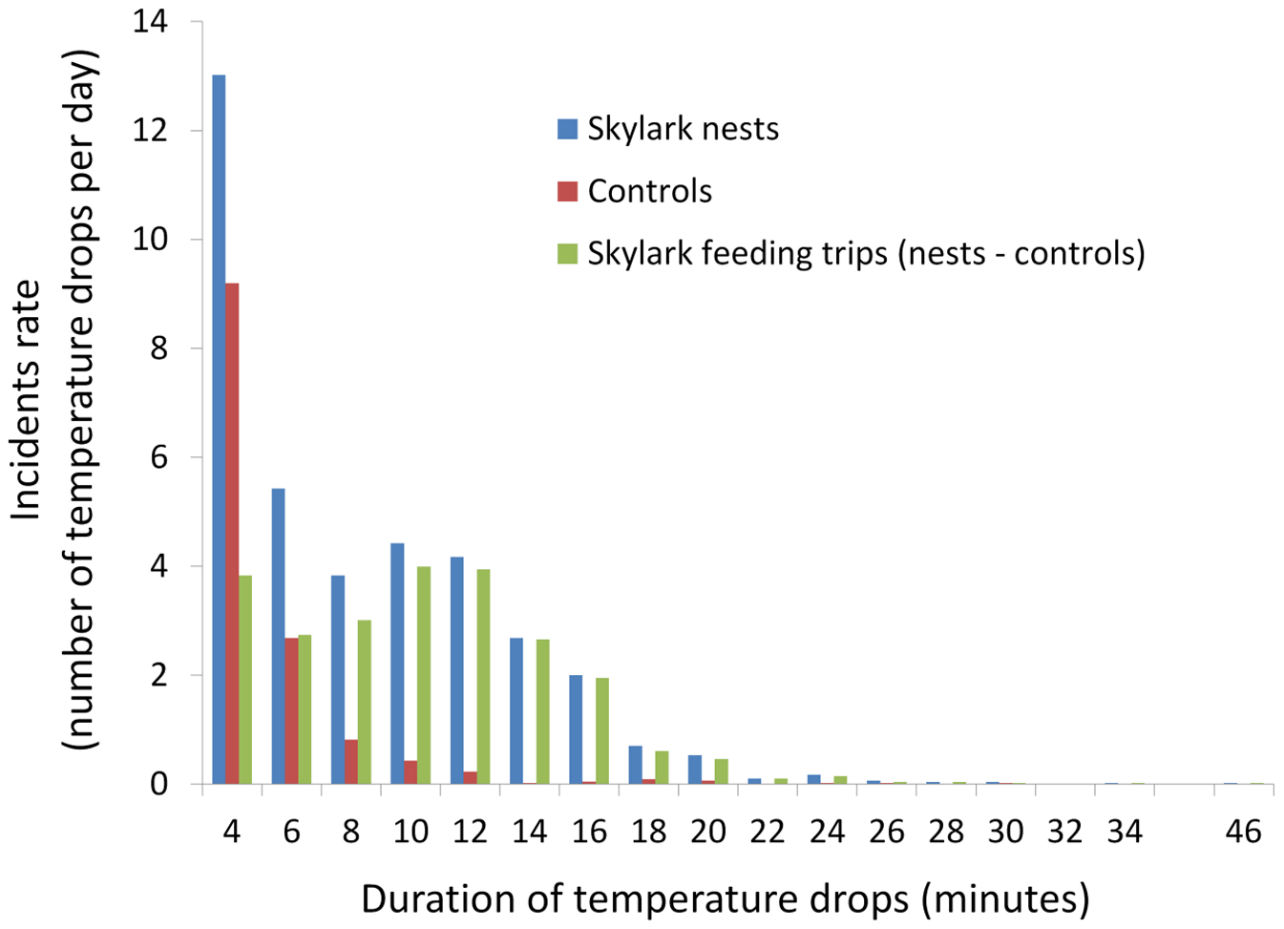
Frequency distribution of the length of temperature drops measured with temperature loggers placed in seven skylark nest and two control locations in the Kalø study area during the breeding season 2005 on which the frequency distribution of the length of foraging trips of incubating females was estimated.

The original 2004 model assumed 30 trips per day, and calculated trip length according to the amount of time required to obtain enough energy to fulfil the skylark's requirements during incubation. The current version replaced this with a parameter for the length of each trip and thus trip number became emergent. The mean trip-length was therefore a result of the requirement for food to maintain the female's energy balance with the availability of food from the home range, and the rate of nutrient extraction determined by weather and vegetation structure on a day to day basis. Thus, *TRIPLENGTH* was a fitting parameter allowed to vary freely, and its optimum fit point was established to be 10.5 minutes. The independent analysis of field data indicated that the length of trips in reality was a mean of 10.3 minutes, with most feeding trips lasting from 10 to 12 minutes (Fig. 7).

#### 4.2.3. Skylark scrapes on 2nd field

The number of territorial pairs for the field without scrapes in the model in 1991 was five, fitting with numbers observed in the real world (Pattern Set 3; see also Fig. 5A). For 1992–1995, the observed values with scrapes were 8, 17, 17, and 13, giving a mean of 13.75. Model predictions for the same period varied for any particular year, but the mean of 10 replicates of 60-year simulations was 12.1 pairs with a range of single-year pair number of 10 to 15. The mean was therefore close, but a little lower in the model than in the real world, with slightly less variation in year-to-year numbers.

## Discussion

### 5.1. Predictive population modelling

Models are key components of wildlife management programmes as they provide a method to predict the outcomes of alternative management strategies. As such the use of models is becoming more commonplace. Traditionally, ecological models have been relatively simple, with the emphasis on generality rather than accuracy. In these models uncertainty is reduced in an effort to improve the precision of their (general) predictions. A predictive population ecology approach, however, is concerned with modelling population impacts arising from complex systems change, placing the emphasis on producing more accurate, if somewhat imprecise predictions about likely future population states in an uncertain world. This addresses what can be subtle, but important, differences between the approach of design sciences such as engineering and the traditional analytic scientific approach to systems understanding.

These design-science agent-based models are generally complex, requiring significant development time and testing, which can be a significant cost, particularly against the policy requirement for models to be as simple and clear as possible. However, once they are developed they can be very flexible due to their modularity e.g. [34], [35]. An advantage of this complexity is that a wide range of novel questions can be asked of the model without a need for re-writing, allowing the model to address a range of research and policy requirements; but the cost is that there may be some redundancy, i.e. parameters that are not important to address the problem in hand.

The ALMaSS skylark model presented here has many of these complex attributes, but we approach the problem from a synthetic design science angle, rather than traditional analytic modelling. Population responses are emergent as a result of interactions between skylark ecology and behaviour, and the landscape, crops and their management, the latter being model inputs. An obvious future application of this model would be to evaluate the impact of recent CAP reforms on skylarks in different agricultural systems. However, regular use of this kind of model for management and policy decisions requires greater confidence in the model and its performance. Hence, exercises such as the one undertaken in this study are required. This is not a case of ‘rubber-stamping’ the model, but of describing its performance and providing a starting place for improvement, ideally based on usage and model users' feedback.

### 5.2. Model testing and sources of error

This study was facilitated by the very large data sets that were available to test the model. Many of these (all post 1995) were specifically created with model testing in mind. Because of this fact the interpretation of the data used for POM testing was easier than in a typical case where patterns are extracted from published data sets or studies. This ensured that we were able to extract data from the model in ways that made it comparative to that measured in the field. This is a critical issue when developing these tests. The study also benefited from the very long period over which data were obtained resulting in a high volume of data, and the fact that inter-annual variation in weather could be factored into the model runs. As a result, many of the fits to the patterns were extremely good; but does this make them correct? Naturally the answer is no, these fits could probably be fitted just as well by other combinations of parameters. However, the extent to which this would have given a model substantially different behaviour is perhaps debatable since experience with refitting of the model during the POM testing suggests that the final fit is very stable, i.e. goodness of fit and patterns of fit re-asserted themselves at each model iteration with only minor variation. In addition, one advantage of the complex agent-based models is that observations can be made at different hierarchical levels, such that a fit to general patterns at a higher level that was achieved by unrealistic behaviour at lower levels can normally be identified and eliminated.

Unlike statistical analysis, with POM testing it is an advantage that many of the parameters are not independent. For instance PE_Max (the daily maximum nestling growth) is a highly sensitive parameter linked to both foraging and growth, but perhaps most importantly it is linked to fledgling mortality via mortality as a result of delayed development. Fledgling mortality was identified as the most sensitive parameter in the previous skylark model version [36]. Since there is no furtFigher behaviour in the model between nest leaving and returning that is not a probabilistic mortality, it is not surprising that PE_Max is now identified as one of the most sensitive parameters. Thus, variations in PE_Max could by and large be compensated for by changing juvenile return mortality, although at a cost of a poorer fit to Pattern Set 2 nest leaving data. Hence, if we trust our Pattern Set 2 data, then we quickly refine the scope for parameter changes in other parts of the model. This is therefore one of the strengths of this approach; new hypotheses can be formulated and tested directly using field data. Further refinements can be included iteratively as more detailed ecological information becomes available, quickly reducing the parameter space further at each iteration.

Despite the acceptable performance of the model, there are a number of clear sources of potential error in the procedure used here. In Bjerringbro there are three issues which could have a bearing on the residuals seen in Fig. 2. Firstly, obtaining a precise match between crop and field and year is currently not possible in ALMaSS, and thus only averages were used. This means any interaction between field location and crop will be missed. For instance, if in reality a very suitable crop was always grown by woodland, it would be unattractive to the skylark, but in the model the crop would be assumed to rotate through all fields and thus skylark estimates would be increased compared to reality. Secondly, it was not possible to account for precise crops and crop management of surrounding fields which were not part of the skylark study areas. Since skylarks' territories overlap field boundaries, we know that significant deviations here will have an effect on the results. The effect of surrounding crop types may also have been an issue with the skylark scrape tests. To get the correct densities for Pattern Set 3, not only the field conditions (crop, hedges etc.), but also the surrounding crops have to be correctly set as they may alter the result. Since the surrounding crops were not recorded, there is further potential for incorrect parameter fitting. Thirdly, we do not take account of the intensiveness of management in the current model, but this could certainly have had an impact on the skylarks. For example, farm 14, with very large fields of 16 ha (mean of the study sites was 3.9 ha), had a low number of skylarks compared to the model prediction. This ‘farm’ was part of a large estate managed very intensively, which could easily explain the discrepancy. Individual residuals notwithstanding, both the fact that the relationship between skylark numbers and the total area in which they were recorded was poor, and the fact that density and skylark numbers were predicted acceptably suggests that the model could be useful to predict changes in skylark numbers resulting from modifications in crops and landscape structure.

Another general source of error that is difficult to deal with but is probably very important is over-fitting in simulation models [37]. Here the problem is that we believe implicitly in our field data. A good example is the Pattern 3 Set (skylark scrapes). A mean statistic was fitted, generated from many replicate model-year runs. However, the field count data were from 1991, and would probably have varied between years if we have had more annual counts. This we can see from the second field where the number of pairs varied between 8 and 17 during the next four years. Thus, by precisely tuning the model to this pattern we may in fact be missing the average case widely. This can of course be true for all other patterns, although Pattern Sets 1 and 2 are built on very substantial data sets and errors of this type are less likely. This is also why, when creating the overall fitting statistic, the individual pattern fit metrics were scaled to be within the same range, and then pattern sets 1 and 2 were weighted double compared to pattern set 3. Given our assessment regarding the reliability of the data, this is probably reasonable, but it is still based on a somewhat subjective assessment.

### 5.3. Future and Open Development

There are two clear next steps to further improve the current model's performance. The first is to develop a more mechanistic module for finding and maintaining skylark territories. The current approach of developing grids is practical, but does not match the reality of plastic territories changing with season and conditions and allowing gradual entry for new birds if conditions improve. For instance, the step-like response visible in Figure 5B was due to a threshold for male territory splitting, and the fact that the interiors of large fields are assumed to be homogenous. This could be improved, which would also probably eliminate the irregular shaped response curves resulting from changing the threshold of territory quality acceptance (see Fig. 6D). The second and more important improvement could come with re-fitting after data collection from other crops. Ideally data on seasonal development of skylark territories in a number of fields in a similar format to that of Pattern Set 3 could be used to be more certain that model skylark responses to different crop structures are accurate.

Improving the usefulness of the model by expanding the geographic area over which it can be used is much more difficult. While it would be possible to take farming systems from another country and use the skylark model directly, there would need to be a number of calibration tests carried out to ensure that other co-varying factors did not unduly influence the results (e.g. testing submodels for vegetation growth and insect biomass), and in non-migratory systems winter behaviour would also need to be incorporated. Development of the model for policy/management evaluation will also require similar data expansions and tests, but in addition would need to involve a wider spectrum of disciplines including socio-economic and political angles leading towards adaptive management concepts [38]. Iterative testing and development cycles would be ideal to tackle these issues [39], but very difficult to maintain over a long period as a scientific project. It may however, be possible as an internet community function.

A major aim in presenting this paper with its model testing is to provide an access point for more general usage, and further improvement of the model. To this end we have provided the model description in ODdox format and have opened the source code for ALMaSS to the research community at http://ccpforge.cse.rl.ac.uk/gf/project/almass/. This is done primarily because we believe that predictive models used in real applied applications are often necessarily complex and therefore require a long-term commitment to development. Without this becoming a community activity, these models will be difficult to build and maintain in the future.

## Supporting Information

Supporting Information S1 **ALMaSS Skylark ODdox Documentation.**
(DOC)

Supporting Information S2 **Sensitivity graphs for all 30 parameters varied during pattern oriented modelling testing.**
(DOC)

Supporting Information S3 **Model modifications resulting from the pattern oriented modelling testing.**
(DOC)

Supporting Information S4 **The skylark ODdox as a zipped archive.**
(ZIP)
