# Supplementary material for: Modelling Skylarks (Alauda arvensis) to Predict Impacts of Changes in Land Management and Policy: Development and Testing of an Agent-Based Model
Source: PLoS One. 2013 Jun 6;8(6):e65803. doi: 10.1371/journal.pone.0065803 (PMC3675089; doi:10.1371/journal.pone.0065803)
Supplement: Supporting Information S1 — ALMaSS Skylark ODdox Documentation. (DOC) [file pone.0065803.s001.doc]

# Supplementary Information S1: ALMaSS Skylark ODdox Documentation

This appendix describes the skylark model based on the ODdox format overview (Topping et al, 2010), but as a text document for journal reproduction. It is strongly recommended to read the ODdox documentation online at: <http://bios.au.dk/fileadmin/Attachments/ODdox/Skylark_V2.0/index.html> where hyperlinks to program code and further description can be found. ODdox is a new documentation method for larger ABM simulations providing access to the source code as well as an overview in close to ODD format (Grimm, Berger et al. 2010) (suitable for smaller models). The skylark ODdox is also available in zipped archive file as Supporting Information S4.

# Overview

## 1. Purpose

The skylark model simulates the ecology and behaviour of the skylark (*Alauda arvensis*) in Denmark. The skylark is a passerine bird, originating in steppes and thus preferring an open habitat for breeding. It is a common bird in agricultural areas in Denmark and N. Europe but has undergone declines over much of its range (Petersen 2007). Its food is both plant and arthropods, but it relies on arthropods during the breeding season, and for feeding the young.

The skylark model was developed to enable impacts of landscape, management, climate and policy changes on skylark populations in Denmark. A key element in its development, along with the other ALMaSS models, was to integrate population modelling with a high resolution dynamic environment. Initially, the skylark model was developed for use in pesticide risk assessment, but subsequently it has been utilised in many other forms of impact assessment. A primary design aim was therefore flexibility to cope with a range of inputs and provision of many output signals. This is achieved by the use of mechanistic modelling processes and high level of realism. 

## 2. State variables and scales

Skylarks are modelled as individuals belonging to one of five classes: 
1) [**Skylark_Clutch**](../../../../../Users/cjt/AppData/Local/Microsoft/Windows/Temporary%20Internet%20Files/DoxygenOutput/Skylark/Skylark_ODdox/class_skylark___clutch.html) - containing a number of eggs. Existing between the first day of laying and hatch (inclusive). 
2) [**Skylark_Nestling**](../../../../../Users/cjt/AppData/Local/Microsoft/Windows/Temporary%20Internet%20Files/DoxygenOutput/Skylark/Skylark_ODdox/class_skylark___nestling.html) - existing from hatch until nest-leaving. 
3) [**Skylark_PreFledgeling**](../../../../../Users/cjt/AppData/Local/Microsoft/Windows/Temporary%20Internet%20Files/DoxygenOutput/Skylark/Skylark_ODdox/class_skylark___pre_fledgeling.html) - existing from nest-leaving until maturity.
4) [**Skylark_Male**](../../../../../Users/cjt/AppData/Local/Microsoft/Windows/Temporary%20Internet%20Files/DoxygenOutput/Skylark/Skylark_ODdox/class_skylark___male.html) - existing from maturity to death.
5) [**Skylark_Female**](../../../../../Users/cjt/AppData/Local/Microsoft/Windows/Temporary%20Internet%20Files/DoxygenOutput/Skylark/Skylark_ODdox/class_skylark___female.html) - existing from maturity to death.

## 3. Process Overview and Scheduling

As with all ALMaSS models the skylark model is based on a state machine which can be described by a state-transition diagram. Birds are in a behavioural state and due to decisions or external events move via transitions to another state where they will exhibit different behaviour.

### Male States

**
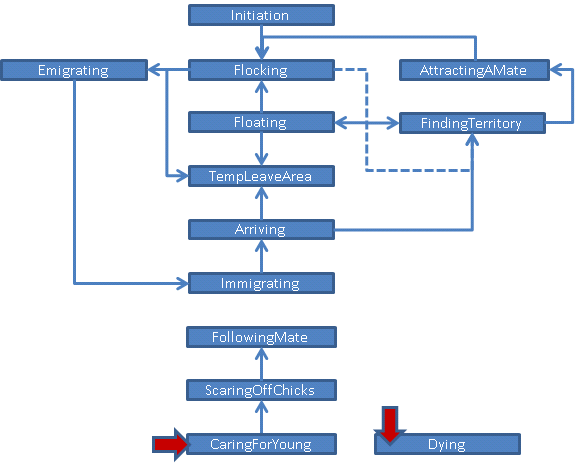
**

**Figure S1:** Male Skylark state-transition diagram. Red arrows indicate external events causing transition to this state.

**Male Initiation** The skylark enters the simulation as an adult. Immediate transition to floating.
**Male Immigration** **[Skylark_Male::st_Immigrating](../../../../../Users/cjt/AppData/Local/Microsoft/Windows/Temporary%20Internet%20Files/DoxygenOutput/Skylark/Skylark_ODdox/class_skylark___male.html#a34705a02beadc3633fd8a059b6744189)**
An instantaneous state which determines the chance of migration mortality. If he does die then he has to inform any old mate that he has gone. If she is already paired with another bird then the model bird just forgets her. If not dying then the bird transitions to st_Arrival.
**Male Temporarily Leave Area** **[Skylark_Male::st_TempLeavingArea](../../../../../Users/cjt/AppData/Local/Microsoft/Windows/Temporary%20Internet%20Files/DoxygenOutput/Skylark/Skylark_ODdox/class_skylark___male.html#a0473e262c63e3b3ea7e7244cf9cc7e31)**
Waits for one week of weather not classified as bad by **[Skylark_Adult::GetBadWeather](../../../../../Users/cjt/AppData/Local/Microsoft/Windows/Temporary%20Internet%20Files/DoxygenOutput/Skylark/Skylark_ODdox/class_skylark___adult.html#a6fe8cc189b4fee2dcf0718672996a433)** 
**Male Attract a Mate [Skylark_Male::st_AttractingAMate](../../../../../Users/cjt/AppData/Local/Microsoft/Windows/Temporary%20Internet%20Files/DoxygenOutput/Skylark/Skylark_ODdox/class_skylark___male.html#a2258d50bc62e93c809be7449ed85ab47)**
Called once each day. The male waits for a female to select and accept his territory. If the breeding season comes to an end, there is a transition to flocking. If a female comes along and pairs then she will trigger a move to st_FollowMate via **[Skylark_Male::OnPairing](../../../../../Users/cjt/AppData/Local/Microsoft/Windows/Temporary%20Internet%20Files/DoxygenOutput/Skylark/Skylark_ODdox/class_skylark___male.html#ab903d7e9903fb182ff8502bfc19ea952)**.
**Male Floating** **[Skylark_Male::st_Floating](../../../../../Users/cjt/AppData/Local/Microsoft/Windows/Temporary%20Internet%20Files/DoxygenOutput/Skylark/Skylark_ODdox/class_skylark___male.html#afceb1d6f6731eb9c3a563539ed50a78e)**
The bird moves around the simulation area continually trying to find vacant territories of suitable quality via regular transition to Find Territory. If the end of the breeding season is reached, then there is a transition to flocking.
**Male Find Territory** **[Skylark_Male::st_FindingTerritory](../../../../../Users/cjt/AppData/Local/Microsoft/Windows/Temporary%20Internet%20Files/DoxygenOutput/Skylark/Skylark_ODdox/class_skylark___male.html#aebdd28cca2c37b172ebc0233bdca0c72)**
The determination of the territory positions and sizes is complicated. The model achieves this through a pre-processing of the landscape on the day in the year that the first bird returns. This pre-processing determines where in the landscape territories can physically fit, and places them ready for the returning birds to select from. Pre-processing is based upon habitat quality criteria related to the height of vegetation and habitat types, and results in a density of skylark territories per 100 ha. Vegetation height in potentially suitable habitats was evaluated based on nesting acceptance criteria. Selection of these territories is based upon the nesting suitability at the time of evaluation; hence, what was a good territory early in the year may be poor later due to vegetation growth (e.g. if sown with winter rape). The result of pre-processing is that territories are therefore fixed each year, and unlike real territories cannot vary in size or shape throughout the season, but may continually change their occupancy status as the season progresses. If a territory quality increases to double that needed to house a single territory then it is assumed to split and dual occupancy is permitted. This simulates the territory plasticity seen during the season in real birds. The male bird therefore assesses territories for acceptance as he moves through the landscape. When he finds a suitable territory he will discontinue searching and make a transition to attract a mate. An older male skylark can remove a younger male from his territory if that male is not already paired.
**Male Emigration [Skylark_Male::st_Emigrating](../../../../../Users/cjt/AppData/Local/Microsoft/Windows/Temporary%20Internet%20Files/DoxygenOutput/Skylark/Skylark_ODdox/class_skylark___male.html#afd58ebcdbc55c579af614c7fc6a4bd49)**
The birds are assumed to be in their over-wintering areas. Date and weather conditions are checked until they are suitable for immigration, at which time there is a transition to **[Skylark_Male::st_Immigrating](../../../../../Users/cjt/AppData/Local/Microsoft/Windows/Temporary%20Internet%20Files/DoxygenOutput/Skylark/Skylark_ODdox/class_skylark___male.html#a34705a02beadc3633fd8a059b6744189)**.
**Male Flocking** **[Skylark_Male::st_Flocking](../../../../../Users/cjt/AppData/Local/Microsoft/Windows/Temporary%20Internet%20Files/DoxygenOutput/Skylark/Skylark_ODdox/class_skylark___male.html#ae04bd46aeaaee3e89860f28de09162bd)**
The bird is assumed to move around the area waiting until weather conditions are suitable to begin emigration, depending upon the time of year. Only arrives here after breeding, so poor conditions will trigger a transition to Skylark_Male::st_Emigration. A transition to st_Emigration will also occur if the bird has not emigrated before October.
**Male Scare off Chicks** **[Skylark_Male::st_ScaringOffChicks](../../../../../Users/cjt/AppData/Local/Microsoft/Windows/Temporary%20Internet%20Files/DoxygenOutput/Skylark/Skylark_ODdox/class_skylark___male.html#a4a8aed44854721887b6d9d8c55d252ea)**
When the chicks reach 30 days old then they are scared out of the territory by the male. After this there is a transition to st_FollowingMate
**Male Follow Mate** **[Skylark_Male::st_FollowingMate](../../../../../Users/cjt/AppData/Local/Microsoft/Windows/Temporary%20Internet%20Files/DoxygenOutput/Skylark/Skylark_ODdox/class_skylark___male.html#a7419bc375d0910db4b97e645ce309022)**
This is simply waiting for a message to say that either female is dead, female leaves or the eggs hatch (NB it does not correspond to mate following behaviour of the real bird, which is shorter duration). The male occupies the same location as the female. When the female's eggs hatch there is a transition to st_CaringForYoung. If the female dies or abandons the territory, there is a transition to AttractingAMate. This method also has another function in that it sets up the food tables for the female, hence must be called before female activity starts.
**Male Die** **[Skylark_Male::st_Dying](../../../../../Users/cjt/AppData/Local/Microsoft/Windows/Temporary%20Internet%20Files/DoxygenOutput/Skylark/Skylark_ODdox/class_skylark___male.html#aba7a14c20d263564ada7938e53d48ec4)**
Removes the skylark from the simulation and ensures that any dependent skylarks are told that the bird has died (mate, and/or young).
**Male Caring for Young [Skylark_Male::st_CaringForYoung](../../../../../Users/cjt/AppData/Local/Microsoft/Windows/Temporary%20Internet%20Files/DoxygenOutput/Skylark/Skylark_ODdox/class_skylark___male.html#a2fa4334226a8245fbcae5e162493d2fd)**
Males use the time available to them for foraging to obtain food resources from their home range. These resources are then reduced by the existence metabolism of the bird, the remainder is then fed to the chicks in lots corresponding to hourly feedings. Feeding is even between chicks if there is sufficient food to satiate all chicks - if less food is available, the largest chick will obtain the food. Males stay in this state until the chicks are 30 days old, when there is a transition of Scare Off Chicks.


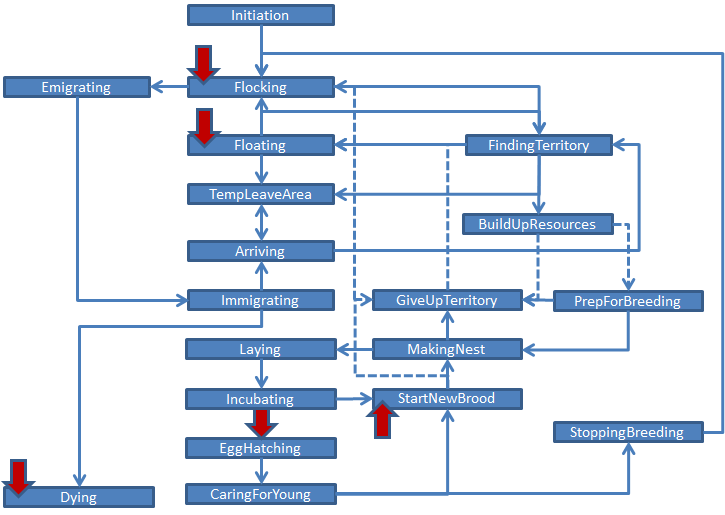


**Figure S2:**Female Skylark state-transition diagram. Red arrows indicate external events causing transition to this state. Hatched arrow is a crossing arrow that does not connect to those transitions it crosses.

### Female States

**Female Initiation** The skylark enters the simulation as an adult and makes an immediate transition to **[Skylark_Female::st_Floating](../../../../../Users/cjt/AppData/Local/Microsoft/Windows/Temporary%20Internet%20Files/DoxygenOutput/Skylark/Skylark_ODdox/class_skylark___female.html#a9dd0ce85e4f9ac9754634438ada9a8b3)**.
**Female Floating** **[Skylark_Female::st_Floating](../../../../../Users/cjt/AppData/Local/Microsoft/Windows/Temporary%20Internet%20Files/DoxygenOutput/Skylark/Skylark_ODdox/class_skylark___female.html#a9dd0ce85e4f9ac9754634438ada9a8b3)**
The bird moves around the simulation area continually trying to find vacant territories of suitable quality via regular transition to Find Territory. If the end of the breeding season is reached, then there is a transition to flocking.
**Female Immigration** **[Skylark_Female::st_Immigrating](../../../../../Users/cjt/AppData/Local/Microsoft/Windows/Temporary%20Internet%20Files/DoxygenOutput/Skylark/Skylark_ODdox/class_skylark___female.html#ac8fe82a57fea485ca9262f9c37f2a8a7)**
An instantaneous state which determines the chance of migration mortality. If she does die then she has to inform any old mate that she has gone, unless he is already paired with another bird. If not dying then breeding success for this year is reset and the bird transitions to st_Arrival.
**Female Arriving** **[Skylark_Female::st_Arriving](../../../../../Users/cjt/AppData/Local/Microsoft/Windows/Temporary%20Internet%20Files/DoxygenOutput/Skylark/Skylark_ODdox/class_skylark___female.html#afbcc74299cd28adef353517b5a1fc693)**
Ensures that the bird is not paired on arrival and checks the weather status. If bad weather then initiates a transition to st_TempLeavingArea otherwise to st_FindTerritory.
**Female Temporarily Leave Area** **[Skylark_Female::st_TempLeavingArea](../../../../../Users/cjt/AppData/Local/Microsoft/Windows/Temporary%20Internet%20Files/DoxygenOutput/Skylark/Skylark_ODdox/class_skylark___female.html#a29e7f929d77e0cc656970f6b77bee2da)**
Waits for one week of weather not classified as bad by **[Skylark_Adult::GetBadWeather](../../../../../Users/cjt/AppData/Local/Microsoft/Windows/Temporary%20Internet%20Files/DoxygenOutput/Skylark/Skylark_ODdox/class_skylark___adult.html#a6fe8cc189b4fee2dcf0718672996a433)** 
**Female Find Territory [Skylark_Female::st_Finding_Territory](../../../../../Users/cjt/AppData/Local/Microsoft/Windows/Temporary%20Internet%20Files/DoxygenOutput/Skylark/Skylark_ODdox/class_skylark___female.html#acd83bcd09f9e5b6512803b5a83882a58)**
This behaviour is the similar to the Male st_Finding_Territory, except that the female looks for territories containing males trying to attract a mate. If her previous mate is present, she will assess his territory first.
**Female Emigration** **[Skylark_Female::st_Emigrating](../../../../../Users/cjt/AppData/Local/Microsoft/Windows/Temporary%20Internet%20Files/DoxygenOutput/Skylark/Skylark_ODdox/class_skylark___female.html#aa3cce931e5577b99cf20b0169652d47a)**
Is called once a day. The birds are assumed to be in their over-wintering areas. This state determines the return to breeding areas based on probabilities that alter with date. Once suitable conditions prevail there will be a transition to **[Skylark_Female::st_Immigrating](../../../../../Users/cjt/AppData/Local/Microsoft/Windows/Temporary%20Internet%20Files/DoxygenOutput/Skylark/Skylark_ODdox/class_skylark___female.html#ac8fe82a57fea485ca9262f9c37f2a8a7)**
**Female Flocking** **[Skylark_Female::st_Flocking](../../../../../Users/cjt/AppData/Local/Microsoft/Windows/Temporary%20Internet%20Files/DoxygenOutput/Skylark/Skylark_ODdox/class_skylark___female.html#a13db7918a1570ea03571469963f51283)**
The bird is assumed to move around the area waiting until weather conditions are suitable to begin Emigration, depending upon the time of year. Only arrives here after breeding, so poor conditions will trigger a transition to st_Emigration. A transition to Emigration will also occur if the bird has not emigrated before October.
**Female Die** **[Skylark_Female::st_Dying](../../../../../Users/cjt/AppData/Local/Microsoft/Windows/Temporary%20Internet%20Files/DoxygenOutput/Skylark/Skylark_ODdox/class_skylark___female.html#a89819138b393537f10b16413b8b2b343)**
Remove the female from the simulation and ensures any dependent skylarks are told that the bird has died (mate, and/or young).
**Female Caring for Young [Skylark_Female::st_CaringForYoung](../../../../../Users/cjt/AppData/Local/Microsoft/Windows/Temporary%20Internet%20Files/DoxygenOutput/Skylark/Skylark_ODdox/class_skylark___female.html#afd47c00c5a8b4a99b332b4527c31e64e)**
Females use the time available to them for foraging to obtain food resources from their home range. These resources are then reduced by the existence metabolism of the bird, the remainder is then fed to the chicks in lots corresponding to hourly feedings. Feeding is even between chicks if there is sufficient food to satiate all chicks - if less food is available, the largest chick will obtain the food. Females stay in this state until the chicks are 18 days old, when they either make a transition to **[Skylark_Female::st_StartingNewBrood](../../../../../Users/cjt/AppData/Local/Microsoft/Windows/Temporary%20Internet%20Files/DoxygenOutput/Skylark/Skylark_ODdox/class_skylark___female.html#a00d2a6fabf6eabf0ecb3a47b6d9ba1b5)**, or if this is their fourth breeding attempt will wait until the chicks are 30 days old and then do Stopping Breeding.
**Egg Hatch** **[Skylark_Female::st_EggHatching](../../../../../Users/cjt/AppData/Local/Microsoft/Windows/Temporary%20Internet%20Files/DoxygenOutput/Skylark/Skylark_ODdox/class_skylark___female.html#aea15943a6ac4e2738976f1de7c5be569)**
A transition is made to **[Skylark_Female::st_CaringForYoung](../../../../../Users/cjt/AppData/Local/Microsoft/Windows/Temporary%20Internet%20Files/DoxygenOutput/Skylark/Skylark_ODdox/class_skylark___female.html#afd47c00c5a8b4a99b332b4527c31e64e)**.
**Incubation** **[Skylark_Female::st_Incubating](../../../../../Users/cjt/AppData/Local/Microsoft/Windows/Temporary%20Internet%20Files/DoxygenOutput/Skylark/Skylark_ODdox/class_skylark___female.html#acbdb342bf765850f40da3e6af9dfb480)**
Incubation occurs as described in development below. The female spends that time off the nest required to find energy to cover her basal metabolic requirements, plus that energy required to warm the eggs. Incubation continues until the eggs hatch and there is a transition to Care For Young, or the incubation period is exceeded, at which point the female will Start New Brood. This state can only be left by a call to OnEggHatch being altered on creation of a nestling or to **[Skylark_Female::st_StartingNewBrood](../../../../../Users/cjt/AppData/Local/Microsoft/Windows/Temporary%20Internet%20Files/DoxygenOutput/Skylark/Skylark_ODdox/class_skylark___female.html#a00d2a6fabf6eabf0ecb3a47b6d9ba1b5)**.
**Egg Laying** **[Skylark_Female::st_Laying](../../../../../Users/cjt/AppData/Local/Microsoft/Windows/Temporary%20Internet%20Files/DoxygenOutput/Skylark/Skylark_ODdox/class_skylark___female.html#acc52654555984e07e388d0727da5f202)**
The female produces one egg per day, if she has built up sufficient energy reserves to accomplish this.
**Stopping Breeding** **[Skylark_Female::st_StoppingBreeding](../../../../../Users/cjt/AppData/Local/Microsoft/Windows/Temporary%20Internet%20Files/DoxygenOutput/Skylark/Skylark_ODdox/class_skylark___female.html#aa34076c2b382f59cf281709e1ff3dc74)**
Called when the female stops breeding for the year. Removes the pair bond and tell the male she is leaving. She deregisters her territory ownership and if there has been no breeding success then she forgets the male, and makes a transition to st_Flocking, otherwise she will pair with him next season if possible.
**Female building up Resources** **[Skylark_Female::st_BuildingUpResources](../../../../../Users/cjt/AppData/Local/Microsoft/Windows/Temporary%20Internet%20Files/DoxygenOutput/Skylark/Skylark_ODdox/class_skylark___female.html#a137f255ea406197182be21175311d1b0)**
The female forages from her home range each day. On 1st April she will make a transition to preparing for breeding which determines the time needed for egg production and nest building.
**Prepare for breeding** **[Skylark_Female::st_PreparingForBreeding](../../../../../Users/cjt/AppData/Local/Microsoft/Windows/Temporary%20Internet%20Files/DoxygenOutput/Skylark/Skylark_ODdox/class_skylark___female.html#a717c8625cf858acbd3db6fb167166e93)**
The female begins construction of a nest in the best nesting habitat in her territory. Construction takes 3 days, after which there is a transition to Egg Laying. Energetic resources are continually built up during this period (see [**Building**](../../../../../Users/cjt/AppData/Local/Microsoft/Windows/Temporary%20Internet%20Files/DoxygenOutput/Skylark/Skylark_ODdox/class_building.html) Up Resources). If the season progresses beyond a fixed end of breeding date then there is a transition to st_GivingUpTerrritory. 
**Make Nest** **[Skylark_Female::st_MakingNest](../../../../../Users/cjt/AppData/Local/Microsoft/Windows/Temporary%20Internet%20Files/DoxygenOutput/Skylark/Skylark_ODdox/class_skylark___female.html#a62ecf31853d4eeae707a4903353708c5)**
Tests for the necessary territory quality, if suitable the female finds a nest location and builds the nest. Nest attempts are assumed to be breeding attempts.
**Give Up Territory** **[Skylark_Female::st_GivingUpTerritory](../../../../../Users/cjt/AppData/Local/Microsoft/Windows/Temporary%20Internet%20Files/DoxygenOutput/Skylark/Skylark_ODdox/class_skylark___female.html#a61df0dd0f11b0bd6baf968101e2c327b)**
The female gives up the territory for this year, informs here mate, and makes a transition to Floating.
**Start New Brood** **[Skylark_Female::st_StartingNewBrood](../../../../../Users/cjt/AppData/Local/Microsoft/Windows/Temporary%20Internet%20Files/DoxygenOutput/Skylark/Skylark_ODdox/class_skylark___female.html#a00d2a6fabf6eabf0ecb3a47b6d9ba1b5)**
Any current nests or clutches are removed. The female assesses the habitat quality of the territory. If still suitable (i.e. aboveMINFEMACCEPTSCORE ) she will make a transition to Make Nest, otherwise she will go to Give Up Territory.

| **Name** | **Description** | **Value** | **References** |
| --- | --- | --- | --- |
| IA | Insect assimilation constant | 0.74 | (Castro et al., 1989) |
| EMA | Existence metabolism for Adults | 31.2 – 0.440T kcal bird ^-1^ day ^-1^  T = ambient temperature | (Kendeigh et al., 1977) |
| AWM | Average weight for adult males | 40.4g | Based on own data (25 individuals) |
| AWF | Average weight for adult females | 36.4g | Based on own data (29 individuals) |

**Table S1:** Inputs and calculations for adult metabolism

### Development of nestlings and eggs

Eggs, nestlings and pre-fledglings have a very simple state/transition diagram existing of Initiation->Development->Maturation/Hatching and Dying.

The model parameters related to reproduction and development are shown in Table S1. The egg-cooling rate (COOLING_RATE_EGGS) during periods of inattentiveness is for isolated eggs in still air and includes heat loss due to radiation, convection and evaporation of moisture through eggshell. Development rate of the eggs and young is controlled by the rate of feeding by the adults. In the eggs this is determined by the amount of time incubated, which is related to the length of time required for the female to obtain enough food to cover her existence metabolism requirements (set as EMA, see Table S1 above). During periods of inattentiveness the eggs cool, and are then warmed up again on the bird's return. The impact on development of the eggs is thus assumed to be:


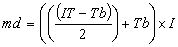


where md is minute degrees, IT is the incubation temperature (EGG_TEMP, Table S2), Tb is the lowest temperature reached during cooling, I is the period of inattentiveness in minutes (based on Drent, 1975). Eggs sum minute degrees until the development total (MINDAYSTOHATCH * EGG_TEMP in minute degrees) is reached. Degrees are summed above a threshold temperature (MD_THRESHOLD, Table S2). Cooling is determined by the number of trips needed to obtain the energy needed for female existence plus incubation costs; this is controlled by the TRIPLENGTH (Table S2) parameter. Mortality of eggs was assumed if development took longer than 17 days (field data observed maximum before desertion of the female).
Nestling growth is determined by the rate of feeding of the adults up to a maximum growth rate determined by the maximum growth rate found in field data and recalculated as a maximum amount of energy used for growth (PE_MAX, Table S2). Conversion efficiencies of dry-weight of insects to bird body weight were obtained from data from the house sparrow (*Passer domesticus*) (Kendeigh, et al., 1977). This data was scaled proportionally for the differences in skylark weight and time taken for growth until adult weight. The extraction rate (EXTRACTION_RATE), of food from habitats is a scaling parameter that was used to adjust the rates of development such that all eggs, nestlings and fledglings developed at the correct rate according to field data (Odderskæer et al., 1997). This same field study provided data on overall mortality rates of eggs and nestlings, which was used to adjust daily predation rates of eggs and nestlings in order to obtain these overall figures.

| **Name** | **Description** | **Value** | **Source** |
| --- | --- | --- | --- |
| COOLING_RATE_EGGS | Egg cooling rate | 2.9 °C Hr ^-1^ °C^-1^  (Fitted 3.0) | Kendeigh et al. 1977 |
| EGG_TEMP | Incubation temperature | 35.0 ° C (Fitted 36.1) | Kendeigh et al. 1977 |
| MINDAYSTOHATCH | Duration of incubation | 9.8 days °C (Fitted 10.2 days) | Own data (N=363) |
| MID | Max. incubation duration | 17 days | Own data |
| MD_THRESHOLD | Threshold for physiological development of eggs | 26 °C (Fitted 25.8) | Drent, 1975 |
| TRIPLENGTH | The length of time spent not incubating per trip off the nest. | 10.5 mins (Fitted) | Fitting parameter |
| MAXFEEDRAIN | Max. Precipitation for parental feeding (daily mean) | 4.7 mm (Fitted) | Fitting parameter |
| ECI | Energy cost of incubation of clutch | 2.4 kcal day^-1^ | Kendeigh et al. 1977 |
| PE_MAX | Max. growth rate of nestlings (1-8 days) | 3.75g day^-1^ (Fitted 4.74 day^-1^) | Own data (N=496) |
| EMJ | Existence metabolism for juveniles (age 1-40) | EMJ = 0.518614 x W^1.1263^ kcal bird^-1^ day^-1^  Calculated from *Passer domesticus* data, adjusted for different skylark weight | Kendeigh et al. 1977 |
| CE | Age-specific conversion efficiency of g dry-weight insects to body weight (age 1-30 days) | CE = 1.180 – 0.035742 x age | Kendeigh et al. 1977 |
| NESTLEAVECHANCE | The chance of leaving the nest once 21g is reached | 23% (Fitted) | Fitting parameter |
| EXTRACTION_RATE | Extraction rate of arthropod food items | 0.00054 (Fitted) | Fitting parameter |

**Table S2:**Skylark parameter inputs and sources . Names longer than three characters in length indicate parameters that were used in recent POM testing.

### Evaluation of territory

Skylark males and females evaluate territory at regular intervals throughout the season, and always whenever there is an important change of state, e.g. to StartingNewBrood using **[SkTerritories::PrePolyNQual](../../../../../Users/cjt/AppData/Local/Microsoft/Windows/Temporary%20Internet%20Files/DoxygenOutput/Skylark/Skylark_ODdox/class_sk_territories.html#a8c1c897c24a11e6fac30dafb0bac766e)**.  Evaluation is by giving each 1m^2^ a score based on habitat characteristics (Table S3) and summing these scores for the whole territory. If a territory score exceeds MINFEMACCEPTSCORE, then the territory is deemed acceptable. The same score is used by both sexes. If the territory score exceeds double the MINFEMACCEPTSCORE, then the male can split the territory to allow another territory to be formed (see **[Skylark_Male::ReEvaluateTerritory](../../../../../Users/cjt/AppData/Local/Microsoft/Windows/Temporary%20Internet%20Files/DoxygenOutput/Skylark/Skylark_ODdox/class_skylark___male.html#aeeade701fe4395418fdd375a53baa715)** ).

Most scores are a direct feature of height or habitat type (e.g. forest results in HQHEDGE), but if a potentially useful nesting location then the height and density of the vegetation will reduce the quality of the habitat above certain values. For any given height (h) above HEIGHTCONST_C or density (d) above DENSITYCONST_C these are expressed as: 
Density reduction = 1 + (( d - *DENSITYCONST_C* )*( d - *DENSITYCONST_C* ) * *DENSITYCONST_B* 
Height reduction = 1 + ( h - *HEIGHTCONST_C* ) * *HEIGHTCONST_B*
- if in either case the result is negative then it is assumed to be zero.

Two other modifiers are also considered. These are premiums added if the habitat has open tramlines or has skylark scrape management, or if it is considered patchy. Skylark scrapes and patchiness are assumed to be a feature of the vegetation and do not change, but tramlines once opened by management activities will close after TRAMLINE_DECAYTIME_DAYS.

### Mortality functions and parameters

All life-stages are subject to mortality. Some of these are directly related to management activities (e.g. ploughing kills clutches and nestlings), others are related to interactions such as nest desertion. There are, however, a number of stochastic parameters that are not attributed to a particular mortality cause but serve to take up those mortalities without direct mechanistic explanation in the model. These fall into two types, those that are applied as daily mortality chances, and those applied once as an overall survival/mortality probability. Two parameters control this daily mortality for all stages until independence at 30 days (CLUTCH_MORT_PROB, NEST_MORT_PROB).
The return rates of adults and young of the year are overall survivorship and are controlled by JUVRETURNMORT, ADULTRETURNMORTMAX.

| **Parameter Name** | **Value** | **Major Mechanism** | **Description** |
| --- | --- | --- | --- |
| DENSITYCONST_B | -0.2625 | T | Exponent for the reduction in habitat score due to increasing vegetation density for vegetation between 0.03m and 1.1m above a threshold given by DENSITYCONST_C. |
| DENSITYCONST_C | 10 | T/E | Threshold for a reduction in quality due to increasing vegetation density for vegetation between 0.03m and 1.1m. |
| HEIGHTCONST_B | -0.22 | T | Exponent for the reduction in habitat score due to increasing vegetation height for vegetation between 0.03m and 1.1m above a threshold (HEIGHTCONST_C) |
| HEIGHTCONST_C | 38 | T/E | Threshold for a height (m) mediated reduction in quality for vegetation between 0.03m and 1.1m. |
| HQBAREEARTH | 3 | T | The quality value/m^2^ per square metre of habitat with vegetation below 3cm tall. |
| HQHEDGE | -250 | T | The quality value/m^2^ associated with hedgerows, forests etc. (above 3m or other tall objects). |
| HQTALL | -2 | T | Territory quality score/m^2^ for vegetation between >1.1m and < 2m. |
| HQTALLVEG | -10 | T | The quality value/m^2^ associated with vegetation between 2m and 3m high. |
| MINFEMACCEPTSCORE | 300000 | T | The minimum total habitat score before the female will accept a territory (used by both sexes). |
| PATCHYPREMIUM | 47 | T | The extra score/m^2^ for habitats assumed to be patchy and accessible to skylarks and less than 2m tall. |
| TRAMLINEPREMIUM | 6 | T | The extra score/m^2^ or fields with open tramlines. |
| TRAMLINE_DECAYTIME | 21 | T/E | The length of time the tramlines are assumed to be open in days. |

**Table S3:**

Input parameters linked to skylark territories with the fitted values (most are scores related to habitat characteristics) and the biological mechanisms that each parameter is related to: E = Energetics and T = Territory quality.

# Design

## 4. Design Concepts

**4.a Emergence**

All aspects of breeding are emergent properties of the energetic relationships resulting from food availability and accessibility. These in turn are functions of weather, vegetation type and farmer management behaviour. Territory occupancy is a function of territory selection behaviour and changes in vegetation structure. Hence, a female will abandon a territory in mid-season if the vegetation grows too tall. Importantly for the skylark, vegetation, management and food are all interlinked in both ALMaSS and the real world, hence emergent patterns are complex including a number of feedback loops.

**4.b. Adaptation**

Adaptation is not used in the model.

**4.c Fitness**

Fitness is determined by age of skylark in any territorial disputes. Otherwise fitness is a question of energy dependent survival as nestlings or pre-fledglings. In a sense fitness is determined by the quality of territory chosen by the male.

**4.d Prediction**

The male predicts the quality of territory based on physical cues when selecting a territory. At the time he selects this territory it is not possible to know how the vegetation will develop, hence prediction is based on location if he has a past breeding history, and in all cases the absence of undesirable structures.

**4.e Sensing**

Skylarks can sense the food levels and accessibility levels in habitats within their home range. They can also sense other skylark territories when searching for a territory.
**4.f Interaction**

Interactions occur between adult skylarks via mate selection, territory competition and caring for young.
**4.g Stochasticity**

Stochasticity is used extensively as the basis for probabilistic decisions, typically associated with mortality events (e.g. see **[Skylark_Clutch::OnFarmEvent](../../../../../Users/cjt/AppData/Local/Microsoft/Windows/Temporary%20Internet%20Files/DoxygenOutput/Skylark/Skylark_ODdox/class_skylark___clutch.html#a19268229bbbe65ed8396ecc59f324370)** ), but also with other distributions, e.g. clutch size with clutch attempt. Typically these are used where there is no mechanical direct way to calculate the effect of an event.

**4.h Collectives**

The [**Skylark_Clutch**](../../../../../Users/cjt/AppData/Local/Microsoft/Windows/Temporary%20Internet%20Files/DoxygenOutput/Skylark/Skylark_ODdox/class_skylark___clutch.html) object is a collective for the eggs in a single clutch. No other collectives are used for the skylark model.
**4.i Observation**

As with all ALMaSS models there are standard outputs in the form of object-class specific probes (see e.g. [**probe_data**](../../../../../Users/cjt/AppData/Local/Microsoft/Windows/Temporary%20Internet%20Files/DoxygenOutput/Skylark/Skylark_ODdox/classprobe__data.html)). The skylark model also has a number of specific output forms relating to breeding success, and pattern oriented modelling data output. 

## 5. Initialization

On starting the model the population is initialized with a number of male and female skylarks (configurable). These birds live one year before immigrating and becoming breeding birds in the simulation.

## 6. Inputs

Many of the parameters of the skylark model are configurable (e.g. migration mortality cfg_adultreturnmort ), although default values are those fitted in a pattern oriented exercise. For a list of skylark parameters and their default values see the configuration variables described in SkylarkConfigs.cpp. Parameters appear in the code identified using a ‘cfg_’ prefix, but in text input in uppercase with ‘SK_’ prefix (e.g. ‘cfg_adultreturnmort’ and ‘SK_ADULTRETURNMORT’).

## 7. Interconnections

Primary connections are between the skylark classes and [**Skylark_Population_Manager**](../../../../../Users/cjt/AppData/Local/Microsoft/Windows/Temporary%20Internet%20Files/DoxygenOutput/Skylark/Skylark_ODdox/class_skylark___population___manager.html) and the [**Landscape**](../../../../../Users/cjt/AppData/Local/Microsoft/Windows/Temporary%20Internet%20Files/DoxygenOutput/Skylark/Skylark_ODdox/class_landscape.html) classes.

Key ways in which the Skylarks interact with the [**Landscape**](../../../../../Users/cjt/AppData/Local/Microsoft/Windows/Temporary%20Internet%20Files/DoxygenOutput/Skylark/Skylark_ODdox/class_landscape.html) class are:

- Finding territory: The model determines territory size and position through a pre-processing of the landscape on the day in the year that the first male bird returns. This pre-processing determines where in the landscape territories can physically fit, and places them ready for the returning male birds to select from. Pre-processing is based upon habitat quality criteria related to the height of vegetation and habitat types, and results in a density of skylark territories per 100 ha. Vegetation height in potentially suitable habitats was evaluated based on nesting acceptance criteria. Selection of these territories is based upon the nesting suitability at the time of evaluation; hence, what was a good territory early in the year may be poor later due to vegetation growth (e.g. if sown with winter rape). The result of pre-processing is that territories are therefore fixed each year, and unlike real territories cannot vary in size or shape throughout the season, but may continually change their occupancy status as the season progresses. The male bird therefore assesses territories for acceptance as he moves through the landscape. When he finds a suitable territory he will discontinue searching and make a transition to attract mate. An older male skylark can remove a younger male from his territory if that male is not already paired.
- Foraging: Skylarks build up resources and care for their young. Skylarks can sense the food levels and accessibility levels in habitats within their home range in the model. Foraging success is determined by the insect biomass and habitat area for each habitat type in the skylark's home range. Insect biomass is in turn dependent upon management and weather.
- Development: all skylark stages are affected by the ambient temperature which determines egg development rate and basal metabolic energetic requirements. Inclement weather during incubation or nestling rearing therefore not only affects the length of time the parents need for foraging but also increases energetic requirements resulting in a positive feedback situation.
- Preparing for breeding/starting new brood: All aspects of breeding are emergent properties of the energetic relationships resulting from food availability and accessibility. These are in turn a function of weather, vegetation type and farmer management behaviour. Territory occupancy is a function of territory selection behaviour and changes in vegetation structure. Hence, a female will abandon a territory in mid-season if the vegetation grows too tall.

[**Skylark_Population_Manager**](../../../../../Users/cjt/AppData/Local/Microsoft/Windows/Temporary%20Internet%20Files/DoxygenOutput/Skylark/Skylark_ODdox/class_skylark___population___manager.html) is responsible for managing lists of the extant birds and carrying out input/output methods. This class is descended from [**Population_Manager**](../../../../../Users/cjt/AppData/Local/Microsoft/Windows/Temporary%20Internet%20Files/DoxygenOutput/Skylark/Skylark_ODdox/class_population___manager.html), which has generally applicable ALMaSS I/O functions, but the [**Skylark_Population_Manager**](../../../../../Users/cjt/AppData/Local/Microsoft/Windows/Temporary%20Internet%20Files/DoxygenOutput/Skylark/Skylark_ODdox/class_skylark___population___manager.html) extends these functions and adds a number that are specific to the skylark. For example, **[Skylark_Population_Manager::ProbePOM](../../../../../Users/cjt/AppData/Local/Microsoft/Windows/Temporary%20Internet%20Files/DoxygenOutput/Skylark/Skylark_ODdox/class_skylark___population___manager.html#add0cc653c518bdeb37a6e125bbe36cd5)** which provides the basic data required for pattern-oriented modelling tests.

## 8. References

Castro G, Stoyan N, Myers JP. 1989. Assimilation efficiency in birds: A function of taxon or food type. Comp Biochem Physiol 92A:271-278.

Drent R. 1975. Incubation. In Farner DS, King JR, Parkes KC, eds, Avian Biology, Vol 5. Academic Press, New York, pp 333-407.

Grimm, V.,Berger, U., DeAngelis, D.L., Polhill, J.G., Giske, J. & Railsback, S.F. 2010. The ODD protocol: A review and first update. Ecological Modelling 221: 2760-2768.

Kendeigh SC, Dol'nik VR, Gavrilov VM. 1977. Avian energetics. In Pinowski J, Kendeigh SC, eds, Granivorous Birds in Ecosystems. Cambridge University Press, UK, pp 129-197.

Odderskær P, Prang A, Poulsen JG, Elmegaard N, Andersen PN.1997. Skylark reproduction in pesticide treated and untreated fields. Pesticide Research No 32. Ministy of Environment and Energy, Denmark.

Petersen, B.S. 2007. Management plan for skylark (*Alauda arvensis*) 2007-2009. Technical Report 006-2007. Commissioned by the European Communities

Topping, C. J., T. T. Hoye and C. R. Olesen (2010). "Opening the black box-Development, testing and documentation of a mechanistically rich agent-based model." Ecological Modelling 221(2): 245-255.
