# Supplementary material for: Modelling Skylarks (Alauda arvensis) to Predict Impacts of Changes in Land Management and Policy: Development and Testing of an Agent-Based Model
Source: PLoS One. 2013 Jun 6;8(6):e65803. doi: 10.1371/journal.pone.0065803 (PMC3675089; doi:10.1371/journal.pone.0065803)

# Supplementary Information S2: Sensitivity graphs for all 30 parameters varied during pattern oriented modelling testing

These graphs represent the full data set generated as the final part of the parameter calibration and sensitivity testing procedure documented in the main text. Parameter names are listed above each graph. To save space on each graph the legend and axes are not labelled. In all cases the x-axis is the percentage that the parameter was varied from the accepted best fit starting point. The y-axis in the size of the fitting metric (zero is perfect fit). The graph legend is below and is common to all graphs:

ADULTRETURNMORT


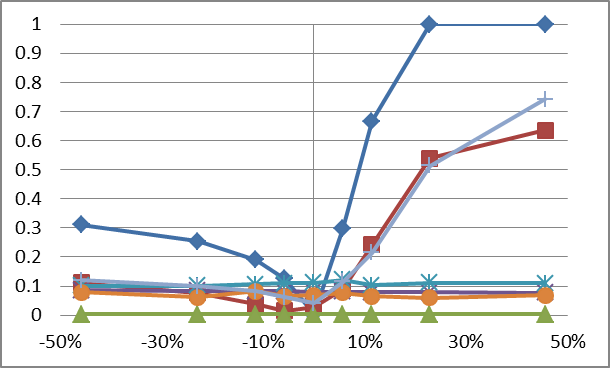


CLUTCH_MORT_PROB


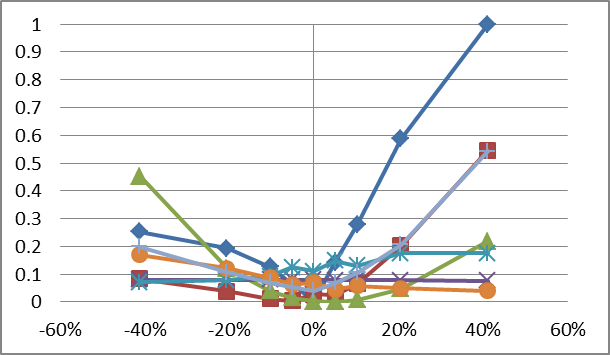


COOLING_RATE_EGGS


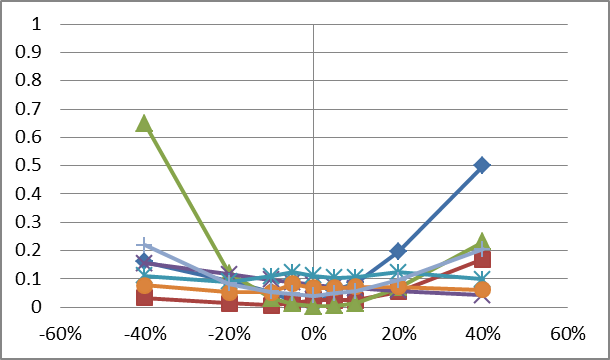


DENSITYCONST_B


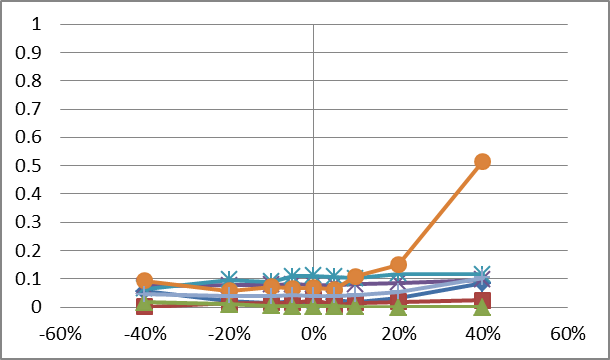


DENSITYCONST_C


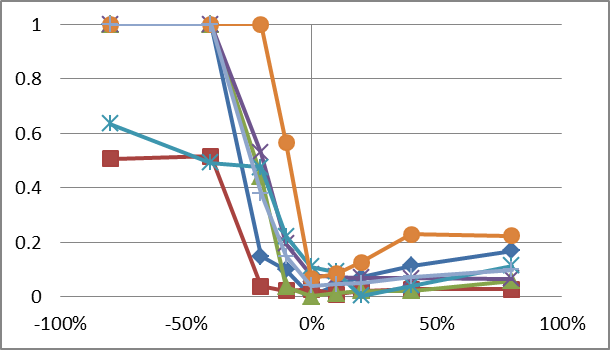


EGGTEMP


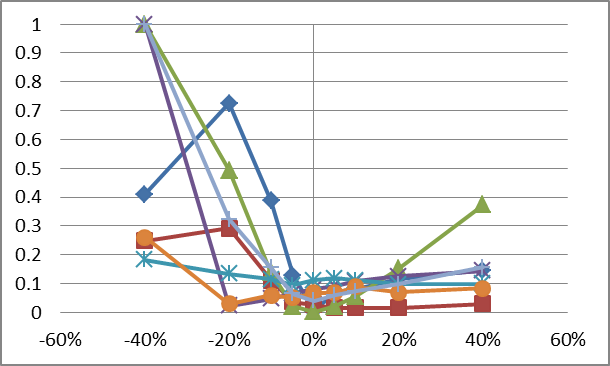


ELEM_TRAMLINE_DECAYTIME_DAYS


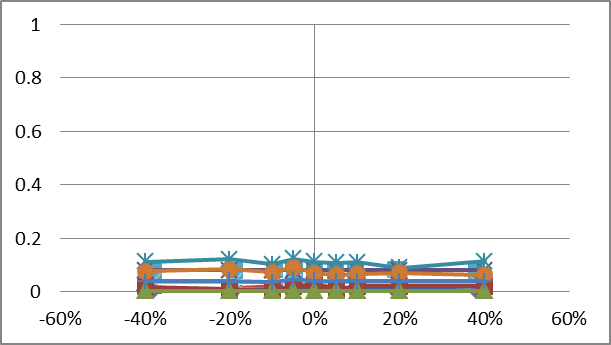


EXTRACTION_RATE


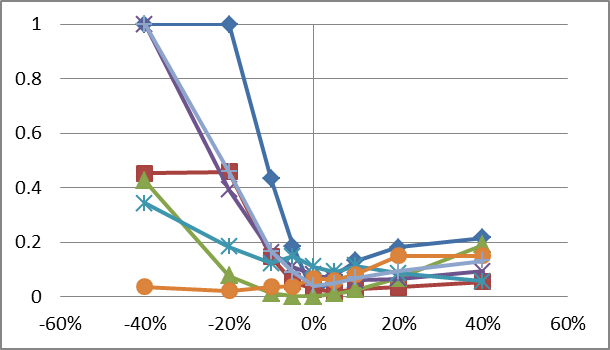


HEIGHTCONST_B


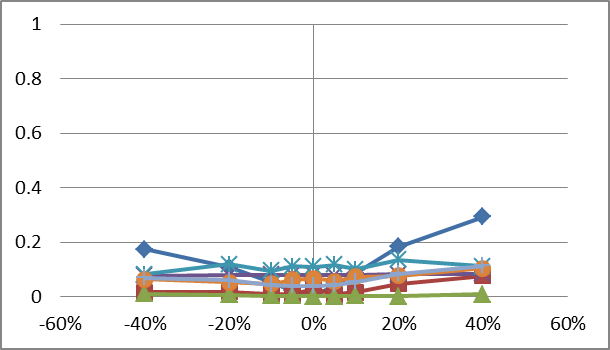


HEIGHTCONST_C


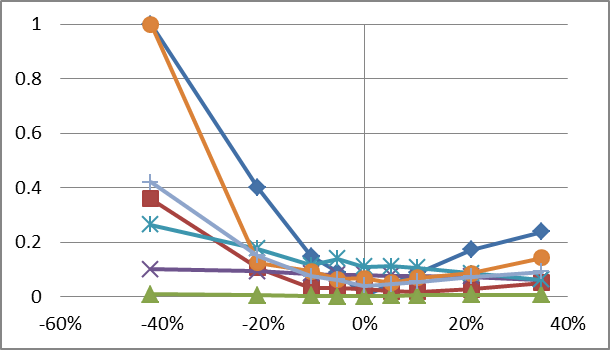


HINDCONSTD_B


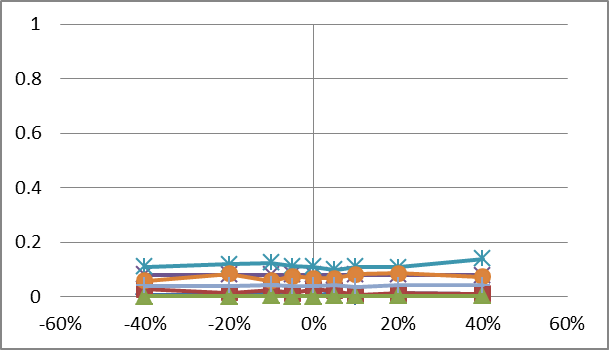


HINDCONSTH_B


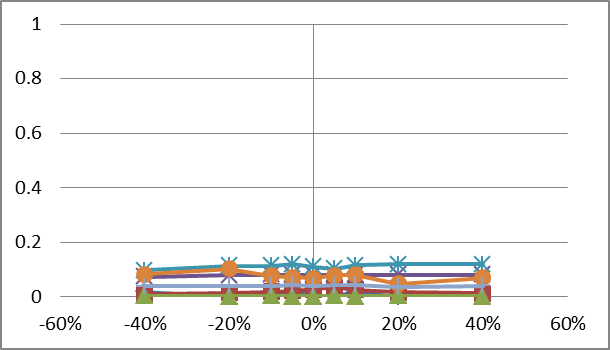


HQBAREEARTH


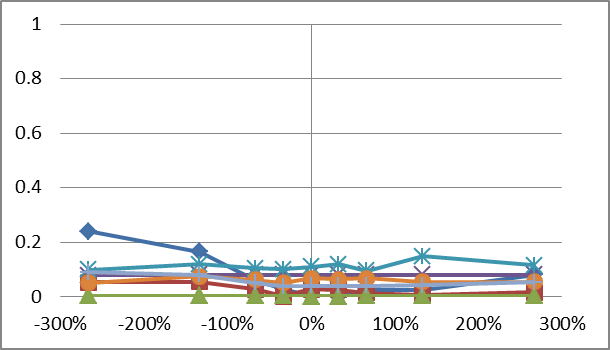


HQHEDGE


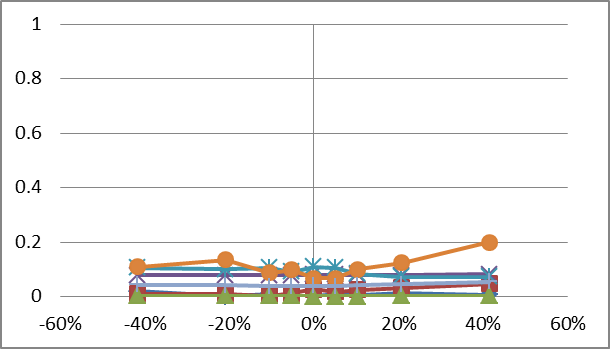


HQTALL


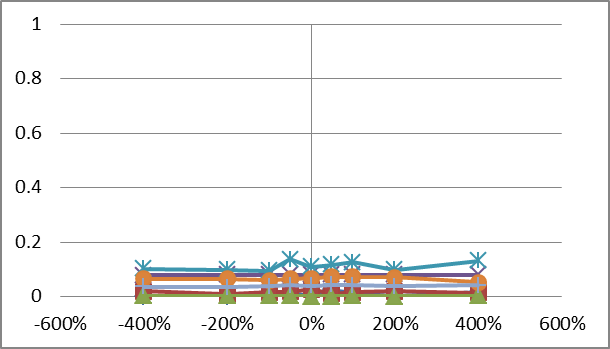


HQTALLVEG


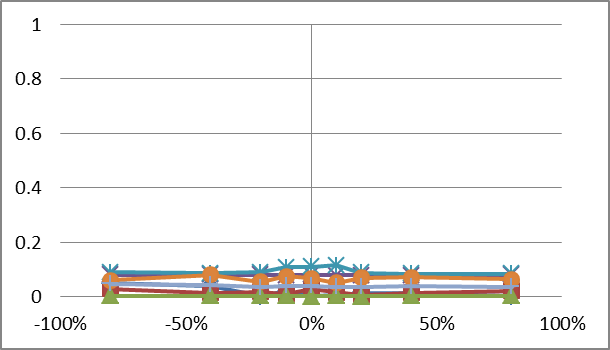


JUVRETURNMORT


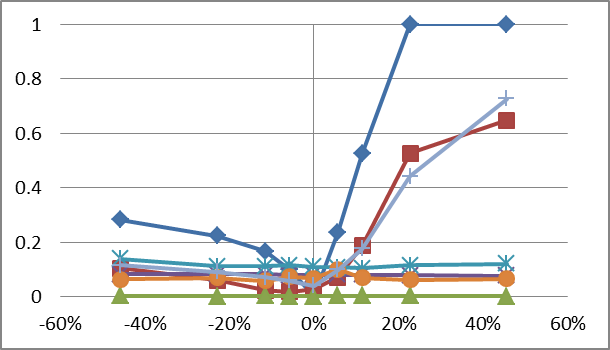


MAXFEEDRAIN


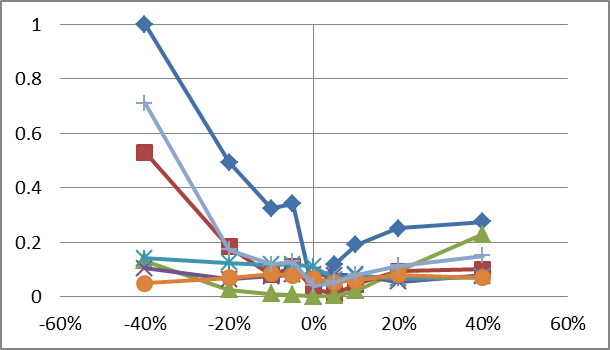


MD_THRESHOLD


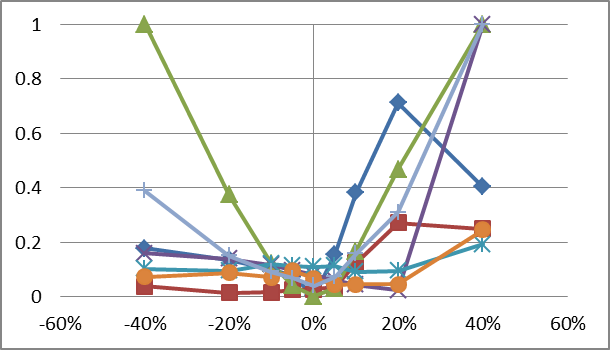


MINDAYSTOHATCH


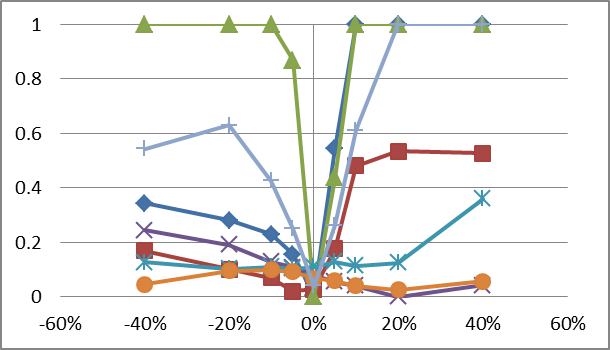


MINFEMACCEPTSCORE


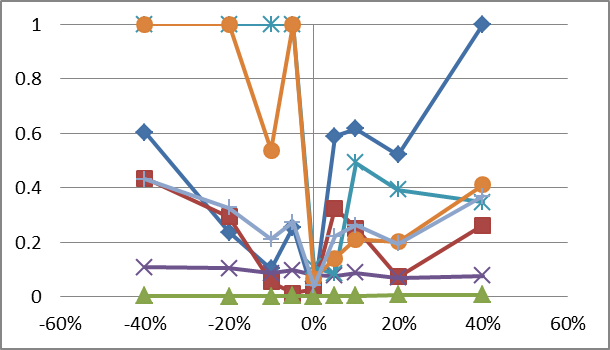


NEST_MORT_PROB


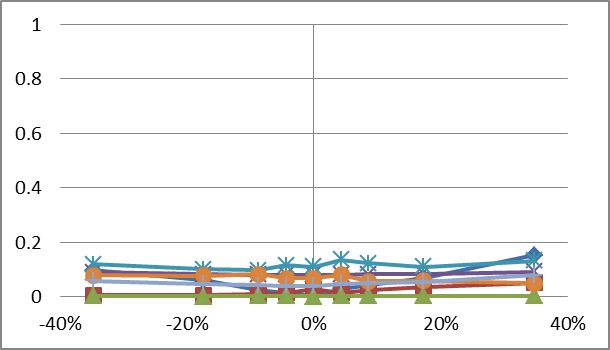


NESTLEAVECHANCE


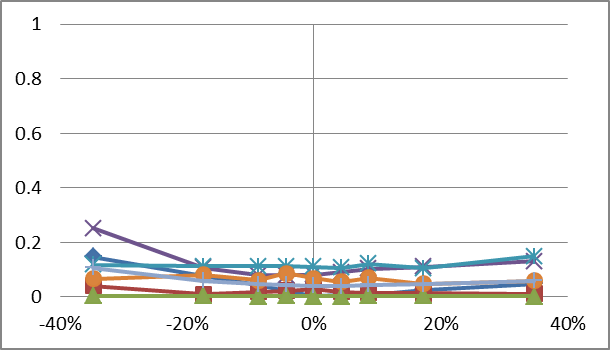


PATCHYPREMIUM


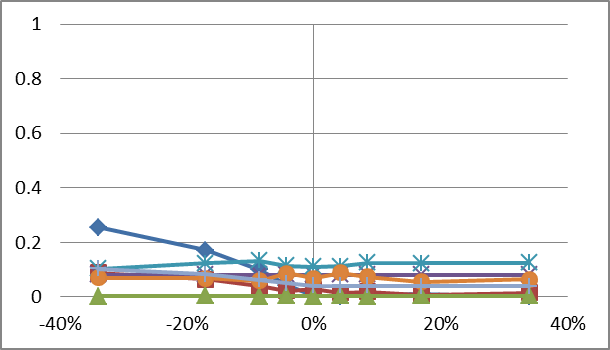


PEMAX


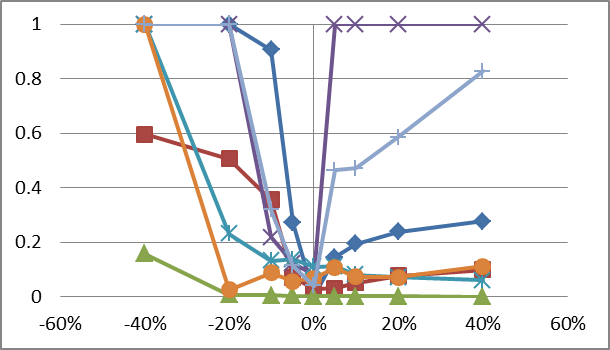


RAINHINDPOW


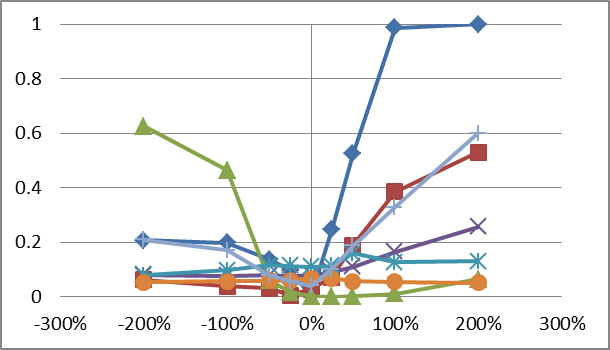


TRAMLINE_FORAGING


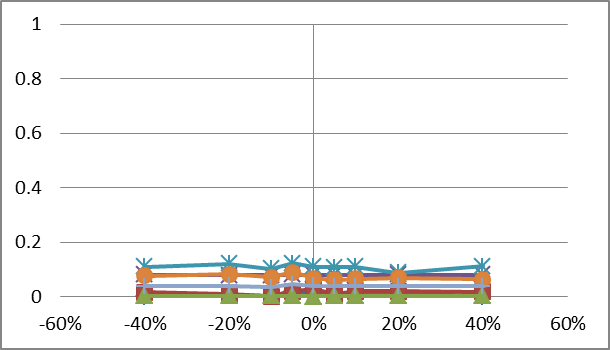


TRAMLINEPREMIUM


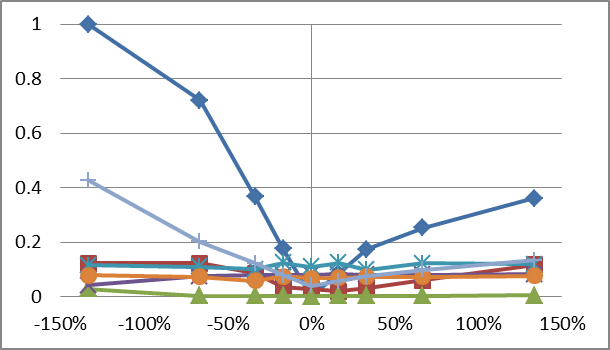


TRIPLENGTH


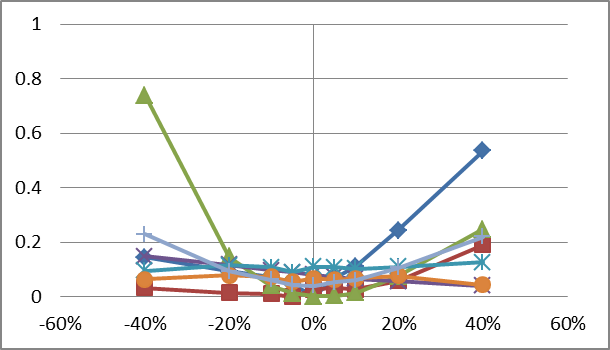


SKSCRAPESPREMIUM (NB this parameter was only tested with the skylark scrape data from Kalø)


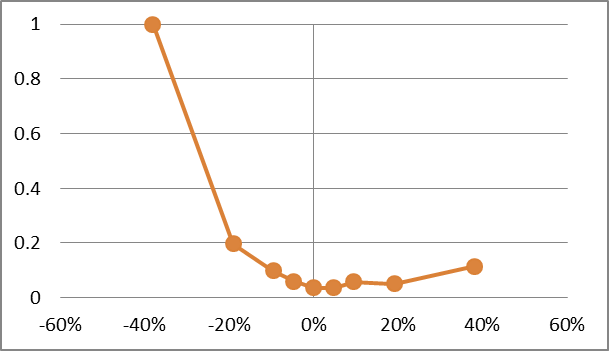

Supplement: Supporting Information S2 — Sensitivity graphs for all 30 parameters varied during pattern oriented modelling testing. (DOC) [file pone.0065803.s002.doc]
