# Supplementary material for: Modelling Skylarks (Alauda arvensis) to Predict Impacts of Changes in Land Management and Policy: Development and Testing of an Agent-Based Model
Source: PLoS One. 2013 Jun 6;8(6):e65803. doi: 10.1371/journal.pone.0065803 (PMC3675089; doi:10.1371/journal.pone.0065803)
Supplement: Supporting Information S3 — Model modifications resulting from the pattern oriented modelling testing. (DOC) [file pone.0065803.s003.doc]

# Supplementary Information S3: Model modifications resulting from the pattern oriented modelling testing

The final model is definitively described by the ODdox documentation in Supplementary Information S1. The overall post-POM testing model structure remains close to the original version from 2004 [1], but the POM testing altered a number of model implementation details in order either to simplify the model, or to improve the model performance. The changes are summarized below:

# Simplifications

- The number of parameters were reduced; four vegetation hindrance equation parameters providing capability of setting thresholds were replaced by constants (0,1); separate male and female territory quality acceptance scores were replaced by a single parameter; there was a reduction in the number of territory quality categories for classification of habitat types (tall, >3m, categories merged).
- The behavioural states MaleEstablishTerritory and FemaleAttractMate were removed. The former was merged with MaleFindingTerritory, the latter was considered to be a rare event and unimportant.
- Nestling growth energetics were simplified to remove loss of weight under low food conditions since this was found not to affect the fit and was difficult to parameterize on the data available.
- The pre-fledgling class behaviour has been largely removed. This life-stage can only affect the overall behaviour of the model via mortality, but we know so little that we cannot reliably distinguish mortality at this stage from overwintering juvenile mortality. The one exception being agricultural mortality due to e.g. harvest killing of very young birds, which has been retained.
- The previous version attempted to use energetics to build up resources for breeding before initiating nest building. This was determined to be unsupportable given current knowledge and was removed in favour of a fixed period of time.

# Model performance improvements

- Assessment of initial territory quality now also uses vegetation height, but not density, as part of the assessment. This improved the fit to the Bjerringbro pair density patterns.
- The effect of increasing density on decreasing habitat quality was altered from linear to a second order geometric curve. This was necessary to derive the observed rapid fall in pair numbers in spring barley without tramlines.
- If skylark males evaluate their territory to be greater than twice the minimum quality, they can now divide their territory and allow another pair to use one half. This simulates the late-season increase in territory density observed in the field data.
- Male and female territory quality assessment for potentially suitable habitat is now altered to take into account the potential for later splitting of territories and additional quality premiums, such as improved quality scores for skylark scrapes or patchy vegetation. The assessment now starts at an assumed score of 50% of the optimum, subtracts vegetation quality reduction factors based on height and density for vegetation above 30cm tall, and then adds premiums. Previously quality was assumed to be optimal, and reductions and premiums applied at all heights. This change was primarily a necessity as a result of the introduction of skylark scrapes, however, it also allows for more general behaviour of increasing pair density with season under good conditions.
- Skylark scrape response code was added to allow simulation of skylark scrape management.
- Males regularly re-evaluate their territories, which may shrink to a smaller size, if the smaller territory has sufficient resources.
- Instead of the number of trips per day, the feeding trip duration is now incorporated as parameter. As a consequence, the number of trips became variable, whereas previously it was the trip duration that varied.
- Feeding is now altered by day-length (i.e. the time available for feeding is daylight hours, and changes with date). This improved the fit to the Patterns Set 2.
- The previous version assessed ‘bad food days’ which were days when food was difficult to obtain due to weather conditions. Accumulation of five such days led to nest abandonment. This was removed in favour of an extra hindrance parameter based on the amount of rainfall each day. This also improved Pattern Set 2 fits.

# References

1. Topping, C.J., et al., Risk assessment of UK skylark populations using life-history and individual-based landscape models. Ecotoxicology, 2005. 14(8): p. 925-936.
