# Supplementary material for: Modelling Skylarks (Alauda arvensis) to Predict Impacts of Changes in Land Management and Policy: Development and Testing of an Agent-Based Model
Source: PLoS One. 2013 Jun 6;8(6):e65803. doi: 10.1371/journal.pone.0065803 (PMC3675089; doi:10.1371/journal.pone.0065803)
Supplement: Supporting Information S4 — The skylark ODdox as a zipped archive. (ZIP) [file pone.0065803.s004.zip › Skylark_ODdox/_a_l_ma_s_s___cmd_line_8cpp.html]

ALMaSS Skylark ODdox: ALMaSS\_CmdLine.cpp File Reference


|  |
| --- |
| ALMaSS Skylark ODdox  2.0 |


- Main Page
- Related Pages
- Classes
- Files

- File List
- File Members

Macros |
Functions |
Variables

ALMaSS\_CmdLine.cpp File Reference

`#include "assert.h"`  
`#include <cstring>`  
`#include <stdio.h>`  
`#include <stdlib.h>`  
`#include <errno.h>`  
`#include <iostream>`  
`#include <fstream>`  
`#include <time.h>`  
`#include "../BatchALMaSS/ALMaSS_Setup.h"`  
`#include "../ALMaSSDefines.h"`  
`#include "../Landscape/ls.h"`  
`#include "../BatchALMaSS/BinaryMapBase.h"`  
`#include "../BatchALMaSS/populationmanager.h"`  
`#include "../Vole/GeneticMaterial.h"`  
`#include "../Skylark/skylarks_all.h"`  
`#include "../Partridge/Partridge_All.h"`  
`#include "../Partridge/Partridge_Population_Manager.h"`  
`#include "../Vole/vole_all.h"`  
`#include "../Vole/VolePopulationManager.H"`  
`#include "../Vole/Predators.H"`  
`#include "../Bembidion/bembidion_all.h"`  
`#include "../Hare/hare_all.h"`  
`#include "../Spider/spider_all.h"`  
`#include "../Spider/SpiderPopulationManager.h"`  
`#include "../BatchALMaSS/BoostRandomGenerators.h"`

|  |  |
| --- | --- |
| Macros | |
| #define | \_CRT\_SECURE\_NO\_DEPRECATE |

|  |  |
| --- | --- |
| Functions | |
| bool | BeginningOfMonth () |
| int | boost\_random010000 (int) |
| void | CloseDownSim () |
| void | CreateLandscape () |
| bool | CreatePopulationManager () |
| void | delay (int secs) |
| void | DumpVegAreaData (int a\_day) |
| void | FloatToDouble (double &d, float f) |
| gen\_type\_int | g\_chance\_gen01000 (g\_generator, distribution\_type(0, 1000)) |
| gen\_type\_int | g\_chance\_gen010000 (g\_generator, distribution\_type(0, 10000)) |
| base\_generator\_type | g\_generator (static\_cast< unsigned int >(std::time(0))) |
| boost::variate\_generator  < base\_generator\_type   &, boost::uniform\_real<> > | g\_rand\_uni (g\_generator, g\_uni\_dist) |
| boost::uniform\_real | g\_uni\_dist (0, 1) |
| void | GetProbeInput\_ini () |
| void | ImpactProbeReport (int a\_time) |
| int | main () |
| void | PredProbeReportDay0 (int a\_time) |
| void | ProbeReport (int a\_time) |
| int | random (int a\_range) |
| bool | ReadBatchINI () |
| void | RunTheSim () |
| void | SpeciesSpecificActions () |
| void | SpeciesSpecificReporting () |

|  |  |
| --- | --- |
| Variables | |
| static CfgInt | cfg\_DayInMonth ("PRB\_DAYINMONTH", CFG\_CUSTOM, 1) |
| CfgBool | cfg\_dumpvegjan |
| CfgStr | cfg\_dumpvegjanfile |
| CfgBool | cfg\_dumpvegjune |
| CfgStr | cfg\_dumpvegjunefile |
| CfgBool | cfg\_fixed\_random\_sequence |
| static CfgBool | cfg\_UseEasyPop ("VOLE\_USEEASYPOP", CFG\_CUSTOM, false) |
| static CfgInt | cfg\_VoleCatastrophe\_interval ("VOLE\_CATASTROPHE\_I", CFG\_CUSTOM, 365 \*5) |
| static CfgInt | cfg\_VoleCatastrophe\_mortality ("VOLE\_CATASTROPHE\_M", CFG\_CUSTOM, 90) |
| static CfgBool | cfg\_VoleCatastrophe\_on ("VOLE\_CATASTROPHE\_ON", CFG\_CUSTOM, false) |
| Landscape \* | g\_ALandscape |
| Population\_Manager \* | g\_AManager |
| boost::generator\_iterator  < gen\_type\_int > g\_chance01000 & | g\_chance\_gen01000 |
| boost::generator\_iterator  < gen\_type\_int >  g\_chance010000 & | g\_chance\_gen010000 |
| char \* | g\_files [100] |
| unsigned | g\_NoOfPredProbes |
| int | g\_NoProbes |
| TPredator\_Population\_Manager \* | g\_PredatorManager |
| char \* | g\_Predfiles [100] |
| char | g\_PredResultsDir [255] |
| const double | g\_randmaxp = RAND\_MAX + 1.0 |
| char | g\_ResultsDir [255] |
| int | g\_Species |
| int | g\_Steps |
| int | g\_time |
| int | g\_torun |
| int | g\_Year |

---

## Macro Definition Documentation

|  |
| --- |
| #define \_CRT\_SECURE\_NO\_DEPRECATE |

---

## Function Documentation

|  |  |  |  |  |
| --- | --- | --- | --- | --- |
| bool BeginningOfMonth | ( |  | ) |  |

Referenced by Population\_Manager::ImpactProbeReport(), and Population\_Manager::ProbeReport().

|  |  |  |  |  |  |
| --- | --- | --- | --- | --- | --- |
| int boost\_random010000 | ( | int |  | ) |  |

{

unsigned result=\*g\_chance010000++;

return result;

}

|  |  |  |  |  |
| --- | --- | --- | --- | --- |
| void CloseDownSim | ( |  | ) |  |

References probe\_data::CloseFile(), g\_ALandscape, g\_cfg, g\_NoProbes, g\_PredatorManager, g\_Species, and Population\_Manager::TheProbe.

Referenced by main().

{

if ( g\_AManager ) {

// Close the probe file

if ( g\_AManager->TheProbe[ 0 ] != NULL ) g\_AManager->TheProbe[ 0 ]->CloseFile();

// delete all probes

for ( int i = 0; i < g\_NoProbes; i++ ) delete g\_AManager->TheProbe[ i ];

delete g\_AManager;

if ( g\_Species == 1 ) {

delete g\_PredatorManager;

}

}

delete g\_ALandscape;

delete g\_cfg;

}

|  |  |  |  |  |
| --- | --- | --- | --- | --- |
| void CreateLandscape | ( |  | ) |  |

Referenced by main().

{

// Create the landscape

g\_ALandscape = new Landscape( "TIALMaSSConfig.cfg", "ErrorFile.txt" );

}

|  |  |  |  |  |
| --- | --- | --- | --- | --- |
| bool CreatePopulationManager | ( |  | ) |  |

References g\_NoOfPredProbes, g\_NoProbes, g\_PredatorManager, g\_Species, Population\_Manager::OpenTheBreedingPairsProbe(), Population\_Manager::OpenTheBreedingSuccessProbe(), Population\_Manager::OpenTheFledgelingProbe(), and Population\_Manager::SetNoProbes().

Referenced by main().

{

// SET UP THE ANIMAL POPULATION

// THE LANDSCAPE MUST BE SETUP BEFORE THE CALL HERE

if ( g\_Species == 0 ) {

Skylark\_Population\_Manager \* skMan = new Skylark\_Population\_Manager( g\_ALandscape );

g\_AManager = skMan;

g\_AManager->OpenTheBreedingPairsProbe();

g\_AManager->OpenTheBreedingSuccessProbe();

g\_AManager->OpenTheFledgelingProbe();

}

if ( g\_Species == 1 ) {

Vole\_Population\_Manager \* vMan = new Vole\_Population\_Manager( g\_ALandscape );

g\_AManager = vMan;

g\_PredatorManager = new TPredator\_Population\_Manager( g\_ALandscape, vMan );

g\_PredatorManager->SetNoProbes( g\_NoOfPredProbes);

}

if ( g\_Species == 2 ) {

Spider\_Population\_Manager \* spMan = new Spider\_Population\_Manager( g\_ALandscape );

g\_AManager = spMan;

}

if ( g\_Species == 3 ) {

Bembidion\_Population\_Manager \* bMan = new Bembidion\_Population\_Manager( g\_ALandscape );

g\_AManager = bMan;

}

if ( g\_Species == 4 ) { // Hare

THare\_Population\_Manager \* hMan = new THare\_Population\_Manager( g\_ALandscape );

g\_AManager = hMan;

}

if ( g\_Species == 5 ) {

Partridge\_Population\_Manager \* pMan = new Partridge\_Population\_Manager( g\_ALandscape );

g\_AManager = pMan;

}

g\_AManager->SetNoProbes(g\_NoProbes);

return true;

}

|  |  |  |  |  |  |
| --- | --- | --- | --- | --- | --- |
| void delay | ( | int | *secs* | ) |  |

{

time\_t start\_time, cur\_time;

time(&start\_time);

do

{

time(&cur\_time);

} while((cur\_time - start\_time) < secs);

}

|  |  |  |  |  |  |
| --- | --- | --- | --- | --- | --- |
| void DumpVegAreaData | ( | int | *a\_day* | ) |  |

|  |  |  |  |
| --- | --- | --- | --- |
| void FloatToDouble | ( | double & | *d*, |
|  |  | float | *f* |
|  | ) |  |  |

Referenced by CropData::CropData(), VegElement::ReadBugPercentageFile(), Configurator::SetCfgFloat(), and Weather::Weather().

{

char \* num = 0;

num = new char[\_CVTBUFSIZE];

errno\_t err = \_gcvt\_s(num, \_CVTBUFSIZE,f,8);

if (err!=0) {

assert(0);

}

d = atof(num);

delete [] num;

}

|  |  |  |  |
| --- | --- | --- | --- |
| gen\_type\_int g\_chance\_gen01000 | ( | g\_generator | , |
|  |  | distribution\_type(0, 1000) |  |
|  | ) |  |  |

|  |  |  |  |
| --- | --- | --- | --- |
| gen\_type\_int g\_chance\_gen010000 | ( | g\_generator | , |
|  |  | distribution\_type(0, 10000) |  |
|  | ) |  |  |

|  |  |  |  |  |  |
| --- | --- | --- | --- | --- | --- |
| base\_generator\_type g\_generator | ( | static\_cast< unsigned int > | std::time(0) | ) |  |

|  |  |  |  |
| --- | --- | --- | --- |
| boost::variate\_generator<base\_generator\_type&, boost::uniform\_real<> > g\_rand\_uni | ( | g\_generator | , |
|  |  | g\_uni\_dist |  |
|  | ) |  |  |

|  |  |  |  |
| --- | --- | --- | --- |
| boost::uniform\_real g\_uni\_dist | ( | 0 | , |
|  |  | 1 |  |
|  | ) |  |  |

|  |  |  |  |  |
| --- | --- | --- | --- | --- |
| void GetProbeInput\_ini | ( |  | ) |  |

References g\_files, g\_NoOfPredProbes, g\_NoProbes, g\_PredatorManager, g\_Predfiles, g\_ResultsDir, g\_Species, probe\_data::OpenFile(), Population\_Manager::ProbeFileInput(), probe\_data::SetFile(), Population\_Manager::TheProbe, and Landscape::Warn().

Referenced by main().

{

char Nme[ 511 ];

ofstream \* AFile=NULL;

for ( int NProbes = 0; NProbes < g\_NoProbes; NProbes++ ) {

// Must read the probe from a file

g\_AManager->TheProbe[ NProbes ] = new probe\_data;

g\_AManager->ProbeFileInput( ( char \* ) g\_files[ NProbes ], NProbes );

char NoProbesString[ 255 ];

sprintf( NoProbesString, "%sProbe.res", g\_ResultsDir );

if ( NProbes == 0 ) {

AFile = g\_AManager->TheProbe[ NProbes ]->OpenFile( NoProbesString );

if ( !AFile ) {

g\_ALandscape->Warn( "BatchALMSS - cannot open Probe File", NULL );

exit( 1 );

}

} else

g\_AManager->TheProbe[ NProbes ]->SetFile( AFile );

}

if ( g\_Species == 1 ) {

for ( int NProbes = 0; NProbes < ( int )g\_NoOfPredProbes; NProbes++ ) {

g\_PredatorManager->TheProbe[ NProbes ] = new probe\_data;

g\_PredatorManager->ProbeFileInput( ( char \* ) g\_Predfiles[ NProbes ], NProbes );

sprintf( Nme, "%sPredProbe%d.res", g\_ResultsDir, NProbes + 1 );

// strcpy( Nme, g\_PredResultsDir );

if ( NProbes == 0 ) {

AFile = g\_PredatorManager->TheProbe[ NProbes ]->OpenFile( Nme );

if ( !AFile ) {

g\_ALandscape->Warn( "BatchALMSS - cannot open Probe File", Nme );

exit( 1 );

}

} else

g\_PredatorManager->TheProbe[ NProbes ]->SetFile( AFile );

}

}

for ( int NProbes = 0; NProbes < g\_NoProbes; NProbes++ ) {

delete [] g\_files[NProbes];

}

for ( int NProbes = 0; NProbes < ( int )g\_NoOfPredProbes; NProbes++ ) {

delete [] g\_Predfiles[NProbes];

}

}

|  |  |  |  |  |  |
| --- | --- | --- | --- | --- | --- |
| void ImpactProbeReport | ( | int | *a\_time* | ) |  |

Referenced by Population\_Manager::SpeciesSpecificReporting().

|  |  |  |  |  |
| --- | --- | --- | --- | --- |
| int main | ( |  | ) |  |

References CloseDownSim(), CreateLandscape(), CreatePopulationManager(), GetProbeInput\_ini(), ReadBatchINI(), RunTheSim(), and CfgBool::value().

{

if (cfg\_fixed\_random\_sequence.value()) srand(0); else srand( ( int )time( NULL ) );

CreateLandscape();

printf( "Landscape Created\n" ); // So now set up a Population Manager

if ( !ReadBatchINI() ) {

char ch;

cout << "Problem with ini file";

cin >> ch;

}

if ( !CreatePopulationManager() ) return false; else

printf( "Population Created\n" ); // Now got to get the probe files read in

GetProbeInput\_ini();

// Ready to go

RunTheSim();

CloseDownSim();

return 0;

}

|  |  |  |  |  |  |
| --- | --- | --- | --- | --- | --- |
| void PredProbeReportDay0 | ( | int | *a\_time* | ) |  |

|  |  |  |  |  |  |
| --- | --- | --- | --- | --- | --- |
| void ProbeReport | ( | int | *a\_time* | ) |  |

Referenced by Population\_Manager::SpeciesSpecificReporting().

|  |  |  |  |  |  |
| --- | --- | --- | --- | --- | --- |
| int random | ( | int | *a\_range* | ) |  |

References g\_rand\_uni.

Referenced by Landscape::AddBeetleBanks(), UserDefinedFarm::AssignPermanentCrop(), Skylark\_Population\_Manager::Catastrophe(), Skylark\_Base::DailyMortality(), RoadsideVerge::DoDevelopment(), Skylark\_Female::FeedYoung(), Weather::GetSnow(), Landscape::hb\_MarkTheBresenhamWay(), Landscape::hb\_StripingDist(), Skylark\_Population\_Manager::Init(), Landscape::Landscape(), Skylark\_Clutch::OnFarmEvent(), Skylark\_Nestling::OnFarmEvent(), Skylark\_PreFledgeling::OnFarmEvent(), Skylark\_Female::OnFarmEvent(), Skylark\_Male::OnFarmEvent(), SkTerritories::PreProcessLandscape2(), VegElement::RandomVegStartValues(), Landscape::ReadPolys(), Population\_Manager::Shuffle(), Skylark\_Adult::Skylark\_Adult(), Skylark\_Nestling::Skylark\_Nestling(), Skylark\_Male::st\_CaringForYoung(), Skylark\_Nestling::st\_Developing(), Skylark\_Female::st\_Emigrating(), Skylark\_Male::st\_Emigrating(), Skylark\_Male::st\_FindingTerritory(), Skylark\_Female::st\_Floating(), Skylark\_Male::st\_Floating(), Skylark\_Female::st\_Immigrating(), Skylark\_Male::st\_Immigrating(), Landscape::SupplyWindDirection(), Pesticide::Test(), Weather::Tick(), and UnsprayedFieldMargin::UnsprayedFieldMargin().

{

/\* Want to raise exception on this?

if ( a\_range <= 0 )

return 0;

\*/

//int result = (int)(((double) rand() / g\_randmaxp ) \* a\_range);

return (int) (g\_rand\_uni()\*a\_range);

// return result;

}

|  |  |  |  |  |
| --- | --- | --- | --- | --- |
| bool ReadBatchINI | ( |  | ) |  |

References g\_files, g\_NoOfPredProbes, g\_NoProbes, g\_Predfiles, g\_PredResultsDir, g\_ResultsDir, g\_Species, and g\_torun.

Referenced by main().

{

// Must read the TIBatch.INI

// Read the INI file

FILE \* Fi = NULL;

char answer = 'X';

Fi=fopen("BatchALMaSS.ini", "r" );

while ( Fi==NULL ) {

// Issue and error warning

cout << "INI File Missing: ";

cout << "BatchALMaSS.ini" << "\n";

cout << "Try Again?";

cin >> answer;

if ( answer != 'Y' ) exit(0);

Fi = fopen("BatchALMaSS.ini", "r" );

}

char Data[ 255 ];

fscanf( Fi, "%d\r", & g\_NoProbes );

for ( int i = 0; i < g\_NoProbes; i++ ) {

fscanf( Fi, "%s\r", Data);

g\_files[ i ] = new char[ 255 ];

strcpy( g\_files[ i ], Data );

}

fscanf( Fi, "%s\r", Data );

strcpy( g\_ResultsDir, Data );

// Read the PredBatch.INI file

if ( g\_Species == 1) {

FILE \* Fi2 = NULL;

answer = 'X';

while ( !Fi2 ) {

Fi2=fopen("VoleToxPreds.ini", "r" );

if ( !Fi2 ) {

// Issue and error warning

cout << "Predator Batch File Missing: VoleToxPreds.INI";

cout << "Try Again (Y/N) ? ";

cin >> answer;

if ( answer != 'Y' ) return false;

}

}

char Data2[ 255 ];

fscanf( Fi2, "%d\r", & g\_NoOfPredProbes );

for ( int i = 0; i < ( int )g\_NoOfPredProbes; i++ ) {

fscanf( Fi2, "%s\r", Data2 );

g\_Predfiles[ i ] = new char[ 255 ];

strcpy( g\_Predfiles[ i ], Data2 );

}

fscanf( Fi2, "%s\r", Data2 );

strcpy( g\_PredResultsDir, Data2 );

fclose( Fi2 );

}

fscanf( Fi, "%d\r", & g\_torun );

g\_torun \*= 365; // the number of years and multiplied by 365 to get days

fscanf( Fi, "%d\r", & g\_Species );

fclose( Fi );

switch (g\_Species) {

case 0:

printf("Running Skylarks\n");

break;

case 1:

printf("Running Voles\n");

break;

case 2:

printf("Running Spiders\n");

break;

case 3:

printf("Running Beetles\n");

break;

case 4:

printf("Running Hares\n");

break;

case 5:

printf("Running Partridges\n");

break;

}

return true;

}

|  |  |  |  |  |
| --- | --- | --- | --- | --- |
| void RunTheSim | ( |  | ) |  |

References Landscape::DumpVegAreaData(), g\_PredatorManager, g\_Species, g\_time, g\_torun, g\_Year, Population\_Manager::Run(), Population\_Manager::SpeciesSpecificReporting(), Landscape::SupplyDayInMonth(), Landscape::SupplyMonth(), and Landscape::TurnTheWorld().

Referenced by main().

{

for ( int i = 0; i < g\_torun; i++ ) {

g\_ALandscape->TurnTheWorld();

// Update the Date

g\_time++;

printf("%d\r",g\_time);

int day = g\_ALandscape->SupplyDayInMonth();

int month = g\_ALandscape->SupplyMonth();

if ( ( day == 1 ) && ( month == 1 ) ) g\_Year++;

if ( g\_Species == 1 ) {

g\_PredatorManager->Run( 1 );

g\_PredatorManager->ProbeReport( g\_time );

}

g\_AManager->Run( 1 );

char str[255];

strcpy(str,g\_AManager->SpeciesSpecificReporting(g\_Species,g\_time));

g\_ALandscape->DumpVegAreaData( g\_time );

}

}

|  |  |  |  |  |
| --- | --- | --- | --- | --- |
| void SpeciesSpecificActions | ( |  | ) |  |

|  |  |  |  |  |
| --- | --- | --- | --- | --- |
| void SpeciesSpecificReporting | ( |  | ) |  |

---

## Variable Documentation

|  |  |  |
| --- | --- | --- |
| |  | | --- | | CfgInt cfg\_DayInMonth("PRB\_DAYINMONTH", CFG\_CUSTOM, 1) | | static |

|  |
| --- |
| CfgBool cfg\_dumpvegjan |

Referenced by Landscape::Landscape().

|  |
| --- |
| CfgStr cfg\_dumpvegjanfile |

Referenced by Landscape::Landscape().

|  |
| --- |
| CfgBool cfg\_dumpvegjune |

Referenced by Landscape::Landscape().

|  |
| --- |
| CfgStr cfg\_dumpvegjunefile |

Referenced by Landscape::Landscape().

|  |
| --- |
| CfgBool cfg\_fixed\_random\_sequence |

|  |  |  |
| --- | --- | --- |
| |  | | --- | | CfgBool cfg\_UseEasyPop("VOLE\_USEEASYPOP", CFG\_CUSTOM, false) | | static |

|  |  |  |
| --- | --- | --- |
| |  | | --- | | CfgInt cfg\_VoleCatastrophe\_interval("VOLE\_CATASTROPHE\_I", CFG\_CUSTOM, 365 \*5) | | static |

|  |  |  |
| --- | --- | --- |
| |  | | --- | | CfgInt cfg\_VoleCatastrophe\_mortality("VOLE\_CATASTROPHE\_M", CFG\_CUSTOM, 90) | | static |

|  |  |  |
| --- | --- | --- |
| |  | | --- | | CfgBool cfg\_VoleCatastrophe\_on("VOLE\_CATASTROPHE\_ON", CFG\_CUSTOM, false) | | static |

|  |
| --- |
| Landscape\* g\_ALandscape |

Referenced by CloseDownSim().

|  |
| --- |
| Population\_Manager\* g\_AManager |

|  |
| --- |
| boost::generator\_iterator<gen\_type\_int> g\_chance01000& g\_chance\_gen01000 |

|  |
| --- |
| boost::generator\_iterator<gen\_type\_int> g\_chance010000& g\_chance\_gen010000 |

|  |
| --- |
| char\* g\_files[100] |

Referenced by GetProbeInput\_ini(), and ReadBatchINI().

|  |
| --- |
| unsigned g\_NoOfPredProbes |

Referenced by CreatePopulationManager(), GetProbeInput\_ini(), and ReadBatchINI().

|  |
| --- |
| int g\_NoProbes |

Referenced by CloseDownSim(), CreatePopulationManager(), GetProbeInput\_ini(), and ReadBatchINI().

|  |
| --- |
| TPredator\_Population\_Manager\* g\_PredatorManager |

Referenced by CloseDownSim(), CreatePopulationManager(), GetProbeInput\_ini(), and RunTheSim().

|  |
| --- |
| char\* g\_Predfiles[100] |

Referenced by GetProbeInput\_ini(), and ReadBatchINI().

|  |
| --- |
| char g\_PredResultsDir[255] |

Referenced by ReadBatchINI().

|  |
| --- |
| const double g\_randmaxp = RAND\_MAX + 1.0 |

|  |
| --- |
| char g\_ResultsDir[255] |

Referenced by GetProbeInput\_ini(), and ReadBatchINI().

|  |
| --- |
| int g\_Species |

Referenced by CloseDownSim(), CreatePopulationManager(), GetProbeInput\_ini(), ReadBatchINI(), and RunTheSim().

|  |
| --- |
| int g\_Steps |

|  |
| --- |
| int g\_time |

Referenced by RunTheSim().

|  |
| --- |
| int g\_torun |

Referenced by ReadBatchINI(), and RunTheSim().

|  |
| --- |
| int g\_Year |

Referenced by RunTheSim().


- CJT
- MSVC
- ALMaSS Working Source
- BatchALMaSS
- ALMaSS\_CmdLine.cpp
- Generated on Thu Jan 10 2013 13:15:35 for ALMaSS Skylark ODdox by
   1.8.1.1
