# Supplementary material for: Modelling Skylarks (Alauda arvensis) to Predict Impacts of Changes in Land Management and Policy: Development and Testing of an Agent-Based Model
Source: PLoS One. 2013 Jun 6;8(6):e65803. doi: 10.1371/journal.pone.0065803 (PMC3675089; doi:10.1371/journal.pone.0065803)
Supplement: Supporting Information S4 — The skylark ODdox as a zipped archive. (ZIP) [file pone.0065803.s004.zip › Skylark_ODdox/_a_l_ma_s_s___setup_8h.html]

ALMaSS Skylark ODdox: ALMaSS\_Setup.h File Reference


|  |
| --- |
| ALMaSS Skylark ODdox  2.0 |


- Main Page
- Related Pages
- Classes
- Files

- File List
- File Members

Macros |
Typedefs

ALMaSS\_Setup.h File Reference

|  |  |
| --- | --- |
| Macros | |
| #define | \_\_64bit |

|  |  |
| --- | --- |
| Typedefs | |
| typedef uint64 | PointerInt |
| typedef \_\_int32 | uint32 |
| typedef \_\_int64 | uint64 |

---

## Macro Definition Documentation

|  |
| --- |
| #define \_\_64bit |

---

## Typedef Documentation

|  |
| --- |
| typedef uint64 PointerInt |

|  |
| --- |
| typedef \_\_int32 uint32 |

|  |
| --- |
| typedef \_\_int64 uint64 |


- CJT
- MSVC
- ALMaSS Working Source
- BatchALMaSS
- ALMaSS\_Setup.h
- Generated on Thu Jan 10 2013 13:15:35 for ALMaSS Skylark ODdox by
   1.8.1.1
