# Supplementary material for: Modelling Skylarks (Alauda arvensis) to Predict Impacts of Changes in Land Management and Policy: Development and Testing of an Agent-Based Model
Source: PLoS One. 2013 Jun 6;8(6):e65803. doi: 10.1371/journal.pone.0065803 (PMC3675089; doi:10.1371/journal.pone.0065803)
Supplement: Supporting Information S4 — The skylark ODdox as a zipped archive. (ZIP) [file pone.0065803.s004.zip › Skylark_ODdox/_boost_random_generators_8h.html]

ALMaSS Skylark ODdox: BoostRandomGenerators.h File Reference


|  |
| --- |
| ALMaSS Skylark ODdox  2.0 |


- Main Page
- Related Pages
- Classes
- Files

- File List
- File Members

Typedefs

BoostRandomGenerators.h File Reference

**BoostRandomGenerators.h Boost headers for using the boost random number generation utilities**
More...

`#include <ctime>`  
`#include <boost/random/linear_congruential.hpp>`  
`#include <boost/random/uniform_int.hpp>`  
`#include <boost/random/uniform_real.hpp>`  
`#include <boost/random/variate_generator.hpp>`  
`#include <boost/random/lagged_fibonacci.hpp>`  
`#include <boost/generator_iterator.hpp>`

|  |  |
| --- | --- |
| Typedefs | |
| typedef   boost::lagged\_fibonacci19937 | base\_generator\_type |
| typedef boost::uniform\_int | distribution\_type |
| typedef   boost::variate\_generator  < base\_generator\_type   &, distribution\_type > | gen\_type\_int |

---

## Detailed Description

**BoostRandomGenerators.h Boost headers for using the boost random number generation utilities**

---

## Typedef Documentation

|  |
| --- |
| typedef boost::lagged\_fibonacci19937 base\_generator\_type |

|  |
| --- |
| typedef boost::uniform\_int distribution\_type |

|  |
| --- |
| typedef boost::variate\_generator<base\_generator\_type&, distribution\_type> gen\_type\_int |


- CJT
- MSVC
- ALMaSS Working Source
- BatchALMaSS
- BoostRandomGenerators.h
- Generated on Thu Jan 10 2013 13:15:35 for ALMaSS Skylark ODdox by
   1.8.1.1
