# Supplementary material for: Modelling Skylarks (Alauda arvensis) to Predict Impacts of Changes in Land Management and Policy: Development and Testing of an Agent-Based Model
Source: PLoS One. 2013 Jun 6;8(6):e65803. doi: 10.1371/journal.pone.0065803 (PMC3675089; doi:10.1371/journal.pone.0065803)
Supplement: Supporting Information S4 — The skylark ODdox as a zipped archive. (ZIP) [file pone.0065803.s004.zip › Skylark_ODdox/_c_i_p_e_landscape_maker_8h.html]

ALMaSS Skylark ODdox: CIPELandscapeMaker.h File Reference


|  |
| --- |
| ALMaSS Skylark ODdox  2.0 |


- Main Page
- Related Pages
- Classes
- Files

- File List
- File Members

Classes

CIPELandscapeMaker.h File Reference

|  |  |
| --- | --- |
| Classes | |
| class | EdgeGrowingPoints |
| struct | Edges |
| struct | EGP\_Data |
| class | PolygonDataVector |


- CJT
- MSVC
- ALMaSS Working Source
- Landscape
- CIPELandscapeMaker.h
- Generated on Thu Jan 10 2013 13:15:35 for ALMaSS Skylark ODdox by
   1.8.1.1
