# Supplementary material for: Modelling Skylarks (Alauda arvensis) to Predict Impacts of Changes in Land Management and Policy: Development and Testing of an Agent-Based Model
Source: PLoS One. 2013 Jun 6;8(6):e65803. doi: 10.1371/journal.pone.0065803 (PMC3675089; doi:10.1371/journal.pone.0065803)
Supplement: Supporting Information S4 — The skylark ODdox as a zipped archive. (ZIP) [file pone.0065803.s004.zip › Skylark_ODdox/_landscape_8cpp.html]

ALMaSS Skylark ODdox: Landscape.cpp File Reference


|  |
| --- |
| ALMaSS Skylark ODdox  2.0 |


- Main Page
- Related Pages
- Classes
- Files

- File List
- File Members

Variables

Landscape.cpp File Reference

`#include "../ALMaSSDefines.h"`  
`#include <cstdio>`  
`#include <stdlib.h>`  
`#include <iostream>`  
`#include <fstream>`  
`#include "../Landscape/ls.h"`  
`#include "../BatchALMaSS/populationmanager.h"`  
`#include "../Skylark/skylarks_all.h"`  
`#include "../Landscape/map_cfg.h"`

|  |  |
| --- | --- |
| Variables | |
| static CfgBool | cfg\_AddBeetleBanks ("BBANKS\_ADD", CFG\_CUSTOM, false) |
| static CfgInt | cfg\_BeetleBankChance ("BBANK\_CHANCE", CFG\_CUSTOM, 100) |
| static CfgFloat | cfg\_BeetleBankMaxArea ("BBANK\_MAXAREA", CFG\_CUSTOM, 0.05) |
| static CfgInt | cfg\_BeetleBankWidth ("BBANK\_WIDTH", CFG\_CUSTOM, 4) |
| static CfgBool | cfg\_CalculateCentroids ("MAP\_CALCULATE\_CENTROIDS", CFG\_CUSTOM, false) |
| CfgBool | cfg\_dumpvegjan ("G\_VEGAREASJAN\_ON", CFG\_CUSTOM, false) |
| CfgStr | cfg\_dumpvegjanfile ("G\_VEGAREASJAN\_FILENAME", CFG\_CUSTOM,"DUMPVEG\_JAN.TXT") |
| CfgBool | cfg\_dumpvegjune ("G\_VEGAREASJUNE\_ON", CFG\_CUSTOM, false) |
| CfgStr | cfg\_dumpvegjunefile ("G\_VEGAREASJUNE\_FILENAME", CFG\_CUSTOM,"DUMPVEG\_JUNE.TXT") |
| CfgFloat | cfg\_fungi\_app\_prop ("CROPS\_FUNGICIDE\_APPLIC\_PROPORTION", CFG\_CUSTOM, 1.0) |
| CfgFloat | cfg\_greg\_app\_prop ("CROPS\_GROWTHREGULATOR\_APPLIC\_PROPORTION", CFG\_CUSTOM, 1.0) |
| CfgInt | cfg\_HedgeSubtypeMaximum ("HEDGE\_SUBTYPEMAXIMUM", CFG\_CUSTOM, 3) |
| CfgInt | cfg\_HedgeSubtypeMinimum ("HEDGE\_SUBTYPEMINIMUM", CFG\_CUSTOM, 0) |
| CfgFloat | cfg\_herbi\_app\_prop ("CROPS\_HERBICIDE\_APPLIC\_PROPORTION", CFG\_CUSTOM, 1.0) |
| CfgFloat | cfg\_ins\_app\_prop ("CROPS\_INSECTICIDE\_APPLIC\_PROPORTION", CFG\_CUSTOM, 1.0) |
| CfgInt | cfg\_l\_treatment\_size ("LAND\_TREATMENTSIZE", CFG\_CUSTOM,-1) |
| CfgInt | cfg\_l\_treatment\_x ("LAND\_TREATMENTX", CFG\_CUSTOM, 0) |
| CfgInt | cfg\_l\_treatment\_y ("LAND\_TREATMENTY", CFG\_CUSTOM, 0) |
| CfgBool | cfg\_l\_usecustompoly ("LAND\_USECUSTOMPOLY", CFG\_CUSTOM, false) |
| static CfgBool | cfg\_map\_usesoiltypes ("MAP\_USESOILTYPES", CFG\_CUSTOM, false) |
| static CfgInt | cfg\_mintownbuildingdistance ("MAP\_MINTOWNBUILDINGDISTANCE", CFG\_CUSTOM, 100) |
| static CfgInt | cfg\_mintownbuildingnumber ("MAP\_MINTOWNBUILDINGNUMBER", CFG\_CUSTOM, 6) |
| CfgInt | cfg\_pesticidetesttype ("PESTICIDETESTYPE", CFG\_CUSTOM,-1) |
| CfgInt | cfg\_productapplicendyear ("PEST\_PROCTAPPLICENDYEAR", CFG\_CUSTOM,-1) |
| CfgInt | cfg\_productapplicstartyear ("PEST\_PROCTAPPLICSTARTYEAR", CFG\_CUSTOM, 9999999) |
| CfgBool | cfg\_rodenticide\_enable ("RODENTICIDE\_ENABLE", CFG\_CUSTOM, false) |
| CfgBool | cfg\_rodenticide\_reporting\_enable ("RODENTICIDE\_REPORTING\_ENABLE", CFG\_CUSTOM, false) |
| CfgFloat | cfg\_silage\_prop ("CROPS\_SILAGE\_PROPORTION", CFG\_CUSTOM, 1.0) |
| CfgFloat | cfg\_strigling\_prop ("CROPS\_STRIGLING\_PROPORTION", CFG\_CUSTOM, 1.0) |
| Landscape \* | g\_landscape\_p |
| double | g\_SpeedyDivides [2001] |
| static CfgBool | l\_map\_art\_hedgebanks ("MAP\_ART\_HEDGEBANKS", CFG\_CUSTOM, false) |
| static CfgInt | l\_map\_chameleon\_replace\_num ("MAP\_CHAMELEON\_REPLACE\_NUM", CFG\_CUSTOM, 20) |
| static CfgBool | l\_map\_check\_polygon\_xref ("MAP\_CHECK\_POLYGON\_XREF", CFG\_CUSTOM, true) |
| static CfgBool | l\_map\_CIPEmaker\_enable ("MAP\_CIPEMAKER\_ENABLE", CFG\_CUSTOM, false) |
| static CfgStr | l\_map\_cropcurves\_file ("MAP\_CROPCURVES\_FILE", CFG\_CUSTOM,"curves.pre") |
| static CfgBool | l\_map\_dump\_enable ("MAP\_DUMP\_ENABLE", CFG\_CUSTOM, false) |
| static CfgBool | l\_map\_dump\_event\_enable ("MAP\_DUMP\_EVENT\_ENABLE", CFG\_CUSTOM, false) |
| static CfgInt | l\_map\_dump\_event\_x1 ("MAP\_DUMP\_EVENT\_XA", CFG\_CUSTOM, 4287) |
| static CfgInt | l\_map\_dump\_event\_x2 ("MAP\_DUMP\_EVENT\_XB", CFG\_CUSTOM, 4333) |
| static CfgInt | l\_map\_dump\_event\_y1 ("MAP\_DUMP\_EVENT\_YA", CFG\_CUSTOM, 2909) |
| static CfgInt | l\_map\_dump\_event\_y2 ("MAP\_DUMP\_EVENT\_YB", CFG\_CUSTOM, 2889) |
| static CfgBool | l\_map\_dump\_exit ("MAP\_DUMP\_EXIT", CFG\_CUSTOM, false) |
| static CfgBool | l\_map\_dump\_gfx\_enable ("MAP\_DUMP\_GFX\_ENABLE", CFG\_CUSTOM, false) |
| static CfgStr | l\_map\_dump\_gfx\_file ("MAP\_DUMP\_GFX\_FILE", CFG\_CUSTOM,"dump.ppm") |
| CfgStr | l\_map\_dump\_map\_file ("MAP\_DUMP\_MAP\_FILE", CFG\_CUSTOM,"dump.lsb") |
| static CfgStr | l\_map\_dump\_margin\_file ("MAP\_DUMP\_MARGIN\_FILE", CFG\_CUSTOM,"dumpunsprayedmargins.txt") |
| CfgStr | l\_map\_dump\_poly\_file ("MAP\_DUMP\_POLY\_FILE", CFG\_CUSTOM,"dumppolyrefs.txt") |
| static CfgBool | l\_map\_dump\_treatcounts\_enable ("MAP\_DUMP\_TREATCOUNTS\_ENABLE", CFG\_CUSTOM, false) |
| static CfgStr | l\_map\_dump\_treatcounts\_file ("MAP\_DUMP\_TREATCOUNTS\_FILE", CFG\_CUSTOM,"treatment\_counts.txt") |
| static CfgBool | l\_map\_dump\_veg\_enable ("MAP\_DUMP\_VEG\_ENABLE", CFG\_CUSTOM, true) |
| static CfgInt | l\_map\_dump\_veg\_x ("MAP\_DUMP\_VEG\_X", CFG\_CUSTOM, 100) |
| static CfgInt | l\_map\_dump\_veg\_y ("MAP\_DUMP\_VEG\_Y", CFG\_CUSTOM, 100) |
| static CfgBool | l\_map\_exit\_on\_zero\_area ("MAP\_EXIT\_ON\_ZERO\_AREA", CFG\_CUSTOM, true) |
| static CfgStr | l\_map\_map\_file ("MAP\_MAP\_FILE", CFG\_CUSTOM,"map.lsb") |
| static CfgInt | l\_map\_no\_pesticide\_fields ("MAP\_NO\_PESTICIDE\_FIELDS", CFG\_CUSTOM, 0) |
| static CfgStr | l\_map\_poly\_file ("MAP\_POLY\_FILE", CFG\_CUSTOM,"polygonrefs.txt") |
| static CfgBool | l\_map\_print\_version\_info ("MAP\_PRINT\_VERSION\_INFO", CFG\_CUSTOM, true) |
| static CfgBool | l\_map\_renumberpolys ("MAP\_RENUMBERPOLY", CFG\_CUSTOM, true) |
| static CfgInt | l\_map\_umargin\_width ("MAP\_UMARGINWIDTH", CFG\_CUSTOM, 12) |
| static CfgStr | l\_map\_weather\_file ("MAP\_WEATHER\_FILE", CFG\_CUSTOM,"weather.pre") |
| CfgBool | l\_pest\_enable\_pesticide\_engine |
| int \* | m\_polymapping |
| static const char \* | version\_date = "2007-09-26" |
| static const int | version\_major = 1 |
| static const int | version\_minor = 0 |
| static const int | version\_revision = 0 |

---

## Variable Documentation

|  |  |  |
| --- | --- | --- |
| |  | | --- | | CfgBool cfg\_AddBeetleBanks("BBANKS\_ADD", CFG\_CUSTOM, false) | | static |

Referenced by Landscape::Landscape().

|  |  |  |
| --- | --- | --- |
| |  | | --- | | CfgInt cfg\_BeetleBankChance("BBANK\_CHANCE", CFG\_CUSTOM, 100) | | static |

Referenced by Landscape::AddBeetleBanks().

|  |  |  |
| --- | --- | --- |
| |  | | --- | | CfgFloat cfg\_BeetleBankMaxArea("BBANK\_MAXAREA", CFG\_CUSTOM, 0.05) | | static |

Referenced by Landscape::BeetleBankPossible().

|  |  |  |
| --- | --- | --- |
| |  | | --- | | CfgInt cfg\_BeetleBankWidth("BBANK\_WIDTH", CFG\_CUSTOM, 4) | | static |

Referenced by Landscape::BeetleBankAdd(), and Landscape::BeetleBankPossible().

|  |  |  |
| --- | --- | --- |
| |  | | --- | | CfgBool cfg\_CalculateCentroids("MAP\_CALCULATE\_CENTROIDS", CFG\_CUSTOM, false) | | static |

Referenced by Landscape::Landscape().

|  |
| --- |
| CfgBool cfg\_dumpvegjan("G\_VEGAREASJAN\_ON", CFG\_CUSTOM, false) |

Referenced by Landscape::Landscape().

|  |
| --- |
| CfgStr cfg\_dumpvegjanfile("G\_VEGAREASJAN\_FILENAME", CFG\_CUSTOM,"DUMPVEG\_JAN.TXT") |

Referenced by Landscape::Landscape().

|  |
| --- |
| CfgBool cfg\_dumpvegjune("G\_VEGAREASJUNE\_ON", CFG\_CUSTOM, false) |

Referenced by Landscape::Landscape().

|  |
| --- |
| CfgStr cfg\_dumpvegjunefile("G\_VEGAREASJUNE\_FILENAME", CFG\_CUSTOM,"DUMPVEG\_JUNE.TXT") |

Referenced by Landscape::Landscape().

|  |
| --- |
| CfgFloat cfg\_fungi\_app\_prop("CROPS\_FUNGICIDE\_APPLIC\_PROPORTION", CFG\_CUSTOM, 1.0) |

|  |
| --- |
| CfgFloat cfg\_greg\_app\_prop("CROPS\_GROWTHREGULATOR\_APPLIC\_PROPORTION", CFG\_CUSTOM, 1.0) |

|  |
| --- |
| CfgInt cfg\_HedgeSubtypeMaximum("HEDGE\_SUBTYPEMAXIMUM", CFG\_CUSTOM, 3) |

Referenced by Landscape::Landscape().

|  |
| --- |
| CfgInt cfg\_HedgeSubtypeMinimum("HEDGE\_SUBTYPEMINIMUM", CFG\_CUSTOM, 0) |

Referenced by Landscape::Landscape().

|  |
| --- |
| CfgFloat cfg\_herbi\_app\_prop("CROPS\_HERBICIDE\_APPLIC\_PROPORTION", CFG\_CUSTOM, 1.0) |

|  |
| --- |
| CfgFloat cfg\_ins\_app\_prop("CROPS\_INSECTICIDE\_APPLIC\_PROPORTION", CFG\_CUSTOM, 1.0) |

|  |
| --- |
| CfgInt cfg\_l\_treatment\_size("LAND\_TREATMENTSIZE", CFG\_CUSTOM,-1) |

Referenced by Landscape::ChangeMapMapping().

|  |
| --- |
| CfgInt cfg\_l\_treatment\_x("LAND\_TREATMENTX", CFG\_CUSTOM, 0) |

Referenced by Landscape::ChangeMapMapping().

|  |
| --- |
| CfgInt cfg\_l\_treatment\_y("LAND\_TREATMENTY", CFG\_CUSTOM, 0) |

Referenced by Landscape::ChangeMapMapping().

|  |
| --- |
| CfgBool cfg\_l\_usecustompoly("LAND\_USECUSTOMPOLY", CFG\_CUSTOM, false) |

Referenced by Landscape::ChangeMapMapping().

|  |  |  |
| --- | --- | --- |
| |  | | --- | | CfgBool cfg\_map\_usesoiltypes("MAP\_USESOILTYPES", CFG\_CUSTOM, false) | | static |

Referenced by Landscape::ReadPolys().

|  |  |  |
| --- | --- | --- |
| |  | | --- | | CfgInt cfg\_mintownbuildingdistance("MAP\_MINTOWNBUILDINGDISTANCE", CFG\_CUSTOM, 100) | | static |

Referenced by Landscape::BuildingDesignationCalc().

|  |  |  |
| --- | --- | --- |
| |  | | --- | | CfgInt cfg\_mintownbuildingnumber("MAP\_MINTOWNBUILDINGNUMBER", CFG\_CUSTOM, 6) | | static |

Referenced by Landscape::BuildingDesignationCalc().

|  |
| --- |
| CfgInt cfg\_pesticidetesttype("PESTICIDETESTYPE", CFG\_CUSTOM,-1) |

Referenced by Landscape::Landscape().

|  |
| --- |
| CfgInt cfg\_productapplicendyear("PEST\_PROCTAPPLICENDYEAR", CFG\_CUSTOM,-1) |

Referenced by Landscape::Tick().

|  |
| --- |
| CfgInt cfg\_productapplicstartyear("PEST\_PROCTAPPLICSTARTYEAR", CFG\_CUSTOM, 9999999) |

Referenced by Landscape::Tick().

|  |
| --- |
| CfgBool cfg\_rodenticide\_enable("RODENTICIDE\_ENABLE", CFG\_CUSTOM, false) |

Referenced by Landscape::Landscape(), Landscape::SupplyRodenticide(), Landscape::Tick(), and Landscape::~Landscape().

|  |
| --- |
| CfgBool cfg\_rodenticide\_reporting\_enable("RODENTICIDE\_REPORTING\_ENABLE", CFG\_CUSTOM, false) |

|  |
| --- |
| CfgFloat cfg\_silage\_prop("CROPS\_SILAGE\_PROPORTION", CFG\_CUSTOM, 1.0) |

|  |
| --- |
| CfgFloat cfg\_strigling\_prop("CROPS\_STRIGLING\_PROPORTION", CFG\_CUSTOM, 1.0) |

|  |
| --- |
| Landscape\* g\_landscape\_p |

Referenced by Farm::AutumnHarrow(), Farm::AutumnPlough(), Farm::AutumnRoll(), Farm::AutumnSow(), Farm::BurnStrawStubble(), Farm::CattleIsOut(), Farm::CattleIsOutLow(), Farm::CattleOut(), Farm::CattleOutLowGrazing(), Farm::CutToHay(), Farm::CutToSilage(), Farm::CutWeeds(), Farm::DeepPlough(), VegElement::DoDevelopment(), Field::DoDevelopment(), Orchard::DoDevelopment(), OrchardBand::DoDevelopment(), Farm::FA\_AmmoniumSulphate(), Farm::FA\_GreenManure(), Farm::FA\_Manure(), Farm::FA\_NPK(), Farm::FA\_PK(), Farm::FA\_Sludge(), Farm::FA\_Slurry(), Farm::FP\_GreenManure(), Farm::FP\_LiquidNH3(), Farm::FP\_ManganeseSulphate(), Farm::FP\_Manure(), Farm::FP\_NPK(), Farm::FP\_NPKS(), Farm::FP\_PK(), Farm::FP\_Sludge(), Farm::FP\_Slurry(), Farm::Harvest(), Farm::HayBailing(), Farm::HayTurning(), Farm::HillingUp(), Farm::PigsAreOut(), Farm::PigsAreOutForced(), Farm::PigsOut(), VegElement::RecalculateBugsNStuff(), Farm::RowCultivation(), Farm::SleepAllDay(), Farm::SpringHarrow(), Farm::SpringPlough(), Farm::SpringRoll(), Farm::SpringSow(), Farm::StrawChopping(), Farm::Strigling(), Farm::StriglingSow(), Farm::StubbleHarrowing(), Farm::Swathing(), Farm::Water(), and Farm::WinterPlough().

|  |
| --- |
| double g\_SpeedyDivides[2001] |

Referenced by Landscape::Landscape().

|  |  |  |
| --- | --- | --- |
| |  | | --- | | CfgBool l\_map\_art\_hedgebanks("MAP\_ART\_HEDGEBANKS", CFG\_CUSTOM, false) | | static |

Referenced by Landscape::Landscape().

|  |  |  |
| --- | --- | --- |
| |  | | --- | | CfgInt l\_map\_chameleon\_replace\_num("MAP\_CHAMELEON\_REPLACE\_NUM", CFG\_CUSTOM, 20) | | static |

Referenced by Landscape::ReadPolys().

|  |  |  |
| --- | --- | --- |
| |  | | --- | | CfgBool l\_map\_check\_polygon\_xref("MAP\_CHECK\_POLYGON\_XREF", CFG\_CUSTOM, true) | | static |

Referenced by Landscape::ChangeMapMapping().

|  |  |  |
| --- | --- | --- |
| |  | | --- | | CfgBool l\_map\_CIPEmaker\_enable("MAP\_CIPEMAKER\_ENABLE", CFG\_CUSTOM, false) | | static |

|  |  |  |
| --- | --- | --- |
| |  | | --- | | CfgStr l\_map\_cropcurves\_file("MAP\_CROPCURVES\_FILE", CFG\_CUSTOM,"curves.pre") | | static |

Referenced by Landscape::Landscape().

|  |  |  |
| --- | --- | --- |
| |  | | --- | | CfgBool l\_map\_dump\_enable("MAP\_DUMP\_ENABLE", CFG\_CUSTOM, false) | | static |

Referenced by Landscape::Landscape().

|  |  |  |
| --- | --- | --- |
| |  | | --- | | CfgBool l\_map\_dump\_event\_enable("MAP\_DUMP\_EVENT\_ENABLE", CFG\_CUSTOM, false) | | static |

Referenced by Landscape::Tick().

|  |  |  |
| --- | --- | --- |
| |  | | --- | | CfgInt l\_map\_dump\_event\_x1("MAP\_DUMP\_EVENT\_XA", CFG\_CUSTOM, 4287) | | static |

Referenced by Landscape::Tick().

|  |  |  |
| --- | --- | --- |
| |  | | --- | | CfgInt l\_map\_dump\_event\_x2("MAP\_DUMP\_EVENT\_XB", CFG\_CUSTOM, 4333) | | static |

Referenced by Landscape::Tick().

|  |  |  |
| --- | --- | --- |
| |  | | --- | | CfgInt l\_map\_dump\_event\_y1("MAP\_DUMP\_EVENT\_YA", CFG\_CUSTOM, 2909) | | static |

Referenced by Landscape::Tick().

|  |  |  |
| --- | --- | --- |
| |  | | --- | | CfgInt l\_map\_dump\_event\_y2("MAP\_DUMP\_EVENT\_YB", CFG\_CUSTOM, 2889) | | static |

Referenced by Landscape::Tick().

|  |  |  |
| --- | --- | --- |
| |  | | --- | | CfgBool l\_map\_dump\_exit("MAP\_DUMP\_EXIT", CFG\_CUSTOM, false) | | static |

Referenced by Landscape::Landscape().

|  |  |  |
| --- | --- | --- |
| |  | | --- | | CfgBool l\_map\_dump\_gfx\_enable("MAP\_DUMP\_GFX\_ENABLE", CFG\_CUSTOM, false) | | static |

Referenced by Landscape::Landscape().

|  |  |  |
| --- | --- | --- |
| |  | | --- | | CfgStr l\_map\_dump\_gfx\_file("MAP\_DUMP\_GFX\_FILE", CFG\_CUSTOM,"dump.ppm") | | static |

Referenced by Landscape::Landscape().

|  |
| --- |
| CfgStr l\_map\_dump\_map\_file("MAP\_DUMP\_MAP\_FILE", CFG\_CUSTOM,"dump.lsb") |

Referenced by Landscape::Landscape().

|  |  |  |
| --- | --- | --- |
| |  | | --- | | CfgStr l\_map\_dump\_margin\_file("MAP\_DUMP\_MARGIN\_FILE", CFG\_CUSTOM,"dumpunsprayedmargins.txt") | | static |

|  |
| --- |
| CfgStr l\_map\_dump\_poly\_file("MAP\_DUMP\_POLY\_FILE", CFG\_CUSTOM,"dumppolyrefs.txt") |

Referenced by Landscape::Landscape().

|  |  |  |
| --- | --- | --- |
| |  | | --- | | CfgBool l\_map\_dump\_treatcounts\_enable("MAP\_DUMP\_TREATCOUNTS\_ENABLE", CFG\_CUSTOM, false) | | static |

Referenced by Landscape::~Landscape().

|  |  |  |
| --- | --- | --- |
| |  | | --- | | CfgStr l\_map\_dump\_treatcounts\_file("MAP\_DUMP\_TREATCOUNTS\_FILE", CFG\_CUSTOM,"treatment\_counts.txt") | | static |

Referenced by Landscape::~Landscape().

|  |  |  |
| --- | --- | --- |
| |  | | --- | | CfgBool l\_map\_dump\_veg\_enable("MAP\_DUMP\_VEG\_ENABLE", CFG\_CUSTOM, true) | | static |

Referenced by Landscape::Landscape(), and Landscape::Tick().

|  |  |  |
| --- | --- | --- |
| |  | | --- | | CfgInt l\_map\_dump\_veg\_x("MAP\_DUMP\_VEG\_X", CFG\_CUSTOM, 100) | | static |

Referenced by Landscape::Tick().

|  |  |  |
| --- | --- | --- |
| |  | | --- | | CfgInt l\_map\_dump\_veg\_y("MAP\_DUMP\_VEG\_Y", CFG\_CUSTOM, 100) | | static |

Referenced by Landscape::Tick().

|  |  |  |
| --- | --- | --- |
| |  | | --- | | CfgBool l\_map\_exit\_on\_zero\_area("MAP\_EXIT\_ON\_ZERO\_AREA", CFG\_CUSTOM, true) | | static |

|  |  |  |
| --- | --- | --- |
| |  | | --- | | CfgStr l\_map\_map\_file("MAP\_MAP\_FILE", CFG\_CUSTOM,"map.lsb") | | static |

Referenced by Landscape::Landscape().

|  |  |  |
| --- | --- | --- |
| |  | | --- | | CfgInt l\_map\_no\_pesticide\_fields("MAP\_NO\_PESTICIDE\_FIELDS", CFG\_CUSTOM, 0) | | static |

Referenced by Landscape::Landscape().

|  |  |  |
| --- | --- | --- |
| |  | | --- | | CfgStr l\_map\_poly\_file("MAP\_POLY\_FILE", CFG\_CUSTOM,"polygonrefs.txt") | | static |

Referenced by Landscape::Landscape().

|  |  |  |
| --- | --- | --- |
| |  | | --- | | CfgBool l\_map\_print\_version\_info("MAP\_PRINT\_VERSION\_INFO", CFG\_CUSTOM, true) | | static |

Referenced by Landscape::Landscape().

|  |  |  |
| --- | --- | --- |
| |  | | --- | | CfgBool l\_map\_renumberpolys("MAP\_RENUMBERPOLY", CFG\_CUSTOM, true) | | static |

Referenced by Landscape::DumpMap(), and Landscape::PolysDump().

|  |  |  |
| --- | --- | --- |
| |  | | --- | | CfgInt l\_map\_umargin\_width("MAP\_UMARGINWIDTH", CFG\_CUSTOM, 12) | | static |

Referenced by Landscape::UnsprayedMarginAdd().

|  |  |  |
| --- | --- | --- |
| |  | | --- | | CfgStr l\_map\_weather\_file("MAP\_WEATHER\_FILE", CFG\_CUSTOM,"weather.pre") | | static |

Referenced by Landscape::Landscape().

|  |
| --- |
| CfgBool l\_pest\_enable\_pesticide\_engine |

Referenced by Pesticide::Pesticide(), Pesticide::Tick(), and Pesticide::~Pesticide().

|  |
| --- |
| int\* m\_polymapping |

Referenced by Landscape::BeetleBankAdd(), Landscape::BorderAdd(), Landscape::BorderScan(), Landscape::BorderScan2(), Landscape::ChangeMapMapping(), Landscape::hb\_Add(), Landscape::hb\_GenerateHBPolys(), Landscape::OrchardBorderAdd(), Landscape::PolysRemoveInvalid(), Landscape::PolysValidate(), RasterMap::RasterMap(), Landscape::ReadPolys(), Landscape::SetLESignal(), Landscape::SupplyDayDegrees(), Landscape::SupplyDeadBiomass(), Landscape::SupplyElementSubType(), Landscape::SupplyElementType(), Landscape::SupplyFarmArea(), Landscape::SupplyFarmIntensity(), Landscape::SupplyFarmOwner(), Landscape::SupplyFarmOwnerIndex(), Landscape::SupplyFarmType(), Landscape::SupplyGrazingPressure(), Landscape::SupplyGreenBiomass(), Landscape::SupplyHasTramlines(), Landscape::SupplyInsects(), Landscape::SupplyIsGrass(), Landscape::SupplyJustMown(), Landscape::SupplyJustSprayed(), Landscape::SupplyLAGreen(), Landscape::SupplyLastTreatment(), Landscape::SupplyLEPointer(), Landscape::SupplyLESignal(), Landscape::SupplyPesticideCell(), Landscape::SupplySkScrapes(), Landscape::SupplyTrafficLoad(), Landscape::SupplyValidX(), Landscape::SupplyValidY(), Landscape::SupplyVegAge(), Landscape::SupplyVegBiomass(), Landscape::SupplyVegCover(), Landscape::SupplyVegDensity(), Landscape::SupplyVegDigestability(), Landscape::SupplyVegHeight(), Landscape::SupplyVegPatchy(), Landscape::SupplyVegType(), Landscape::SupplyWeedBiomass(), Landscape::UnsprayedMarginAdd(), Landscape::UnsprayedMarginScan(), and Landscape::~Landscape().

|  |  |  |
| --- | --- | --- |
| |  | | --- | | const char\* version\_date = "2007-09-26" | | static |

Referenced by Landscape::Landscape().

|  |  |  |
| --- | --- | --- |
| |  | | --- | | const int version\_major = 1 | | static |

Referenced by Landscape::Landscape().

|  |  |  |
| --- | --- | --- |
| |  | | --- | | const int version\_minor = 0 | | static |

Referenced by Landscape::Landscape().

|  |  |  |
| --- | --- | --- |
| |  | | --- | | const int version\_revision = 0 | | static |

Referenced by Landscape::Landscape().


- CJT
- MSVC
- ALMaSS Working Source
- Landscape
- Landscape.cpp
- Generated on Thu Jan 10 2013 13:15:35 for ALMaSS Skylark ODdox by
   1.8.1.1
