# Supplementary material for: Modelling Skylarks (Alauda arvensis) to Predict Impacts of Changes in Land Management and Policy: Development and Testing of an Agent-Based Model
Source: PLoS One. 2013 Jun 6;8(6):e65803. doi: 10.1371/journal.pone.0065803 (PMC3675089; doi:10.1371/journal.pone.0065803)
Supplement: Supporting Information S4 — The skylark ODdox as a zipped archive. (ZIP) [file pone.0065803.s004.zip › Skylark_ODdox/_movement_map_8h.html]

ALMaSS Skylark ODdox: MovementMap.h File Reference


|  |
| --- |
| ALMaSS Skylark ODdox  2.0 |


- Main Page
- Related Pages
- Classes
- Files

- File List
- File Members

Classes

MovementMap.h File Reference

**Movementmap.h This file contains the headers for the MovementMap class**   
More...

|  |  |
| --- | --- |
| Classes | |
| class | IDMap |
|  | Used to map locations of animals in space. More... |
| class | MovementMap |
|  | Movement maps are used for rapid computing of animal movement. More... |
| class | MovementMap16 |
|  | Movement maps are used for rapid computing of animal movement. More... |

---

## Detailed Description

**Movementmap.h This file contains the headers for the MovementMap class**

by Chris J. Topping   
Version of June 2003   
  
Doxygen formatted comments in July 2008


- CJT
- MSVC
- ALMaSS Working Source
- BatchALMaSS
- MovementMap.h
- Generated on Thu Jan 10 2013 13:15:35 for ALMaSS Skylark ODdox by
   1.8.1.1
