# Supplementary material for: Modelling Skylarks (Alauda arvensis) to Predict Impacts of Changes in Land Management and Policy: Development and Testing of an Agent-Based Model
Source: PLoS One. 2013 Jun 6;8(6):e65803. doi: 10.1371/journal.pone.0065803 (PMC3675089; doi:10.1371/journal.pone.0065803)
Supplement: Supporting Information S4 — The skylark ODdox as a zipped archive. (ZIP) [file pone.0065803.s004.zip › Skylark_ODdox/_population_manager_8cpp.html]

ALMaSS Skylark ODdox: PopulationManager.cpp File Reference


|  |
| --- |
| ALMaSS Skylark ODdox  2.0 |


- Main Page
- Related Pages
- Classes
- Files

- File List
- File Members

Classes |
Macros |
Variables

PopulationManager.cpp File Reference

**PopulationManager.cpp This is the code file for the population manager and associated classes**   
More...

`#include <vector>`  
`#include <algorithm>`  
`#include <iostream>`  
`#include <fstream>`  
`#include <string.h>`  
`#include "../ALMaSSDefines.h"`  
`#include "../Landscape/ls.h"`  
`#include "../BatchALMaSS/populationmanager.h"`

|  |  |
| --- | --- |
| Classes | |
| class | CompareState |
|  | Function to compare to TAnimal's Current behavioural state. More... |
| class | CompareStateR |
|  | Function to compare to TAnimal's CurrentStateNo. More... |
| class | CompareX |
|  | Function to compare to TAnimal's m\_Location\_x. More... |
| class | CompareY |
|  | Function to compare to TAnimal's m\_Location\_y. More... |

|  |  |
| --- | --- |
| Macros | |
| #define | \_CRTDBG\_MAP\_ALLOC |

|  |  |
| --- | --- |
| Variables | |
| CfgInt | cfg\_CatastropheEventStartYear ("PM\_CATASTROPHEEVENTSTARTYEAR", CFG\_CUSTOM, 99999) |
| CfgInt | cfg\_CIPEGridOutput\_day ("G\_CIPEGRIDOUTPUT\_DAY", CFG\_CUSTOM, 1) |
| CfgInt | cfg\_CIPEGridOutput\_day\_b ("G\_CIPEGRIDOUTPUT\_DAY\_B", CFG\_CUSTOM, 270) |
| CfgStr | cfg\_CIPEGridOutput\_filename ("G\_CIPEGRIDOUTPUT\_FILENAME", CFG\_CUSTOM,"CIPEGridOutput.txt") |
| CfgStr | cfg\_CIPEGridOutput\_filenameB ("G\_CIPEGRIDOUTPUT\_FILENAME\_B", CFG\_CUSTOM,"CIPEGridOutputB.txt") |
| CfgInt | cfg\_CIPEGridOutput\_Interval ("G\_CIPEGRIDOUTPUT\_INTERVAL", CFG\_CUSTOM, 1) |
| CfgBool | cfg\_CIPEGridOutput\_used ("G\_CIPEGRIDOUTPUT\_USED", CFG\_CUSTOM, false) |
| static CfgInt | cfg\_CipeGridSize ("G\_CIPEGRIDSIZE", CFG\_CUSTOM, 500) |
| static CfgInt | cfg\_DayInMonth ("PRB\_DAYINMONTH", CFG\_CUSTOM, 1) |
| CfgBool | cfg\_fixed\_random\_sequence ("G\_FIXEDRANDOMSEQUENCE", CFG\_CUSTOM, false) |
| CfgInt | cfg\_pm\_eventfrequency ("PM\_EVENTFREQUENCY", CFG\_CUSTOM, 0) |
| CfgInt | cfg\_pm\_eventsize ("PM\_EVENTSIZE", CFG\_CUSTOM, 100) |
| static CfgInt | cfg\_ReallyBigOutput\_day1 ("G\_REALLYBIGOUTPUT\_DAY\_ONE", CFG\_CUSTOM, 1) |
| static CfgInt | cfg\_ReallyBigOutput\_day2 ("G\_REALLYBIGOUTPUT\_DAY\_TWO", CFG\_CUSTOM, 91) |
| static CfgInt | cfg\_ReallyBigOutput\_day3 ("G\_REALLYBIGOUTPUT\_DAY\_THREE", CFG\_CUSTOM, 182) |
| static CfgInt | cfg\_ReallyBigOutput\_day4 ("G\_REALLYBIGOUTPUT\_DAY\_FOUR", CFG\_CUSTOM, 274) |
| static CfgStr | cfg\_ReallyBigOutput\_filename ("G\_REALLYBIGOUTPUT\_FILENAME", CFG\_CUSTOM,"ReallyBigOutput.txt") |
| static CfgInt | cfg\_ReallyBigOutput\_interval ("G\_REALLYBIGOUTPUT\_INTERVAL", CFG\_CUSTOM, 1) |
| CfgBool | cfg\_ReallyBigOutput\_used ("G\_REALLYBIGOUTPUT\_USED", CFG\_CUSTOM, false) |
| static CfgInt | cfg\_ReallyBigOutputFirstYear ("G\_REALLYBIGOUTPUT\_FIRSTYEAR", CFG\_CUSTOM, 1) |
| static CfgInt | cfg\_RipleysOutput\_day ("G\_RIPLEYSOUTPUT\_DAY", CFG\_CUSTOM, 60) |
| static CfgStr | cfg\_RipleysOutput\_filename ("G\_RIPLEYSOUTPUT\_FILENAME", CFG\_CUSTOM,"RipleysOutput.txt") |
| static CfgInt | cfg\_RipleysOutput\_interval ("G\_RIPLEYSOUTPUT\_INTERVAL", CFG\_CUSTOM, 1) |
| CfgBool | cfg\_RipleysOutput\_used ("G\_RIPLEYSOUTPUT\_USED", CFG\_CUSTOM, true) |
| static CfgInt | cfg\_RipleysOutputFirstYear ("G\_RIPLEYSOUTPUT\_FIRSTYEAR", CFG\_CUSTOM, 1) |
| CfgBool | cfg\_RipleysOutputMonthly\_used ("G\_RIPLEYSOUTPUTMONTHLY\_USED", CFG\_CUSTOM, false) |
| static CfgInt | cfg\_VoleCatastrophe\_interval ("VOLE\_CATASTROPHE\_I", CFG\_CUSTOM, 365 \*5) |
| static CfgInt | cfg\_VoleCatastrophe\_mortality ("VOLE\_CATASTROPHE\_M", CFG\_CUSTOM, 90) |
| static CfgBool | cfg\_VoleCatastrophe\_on ("VOLE\_CATASTROPHE\_ON", CFG\_CUSTOM, false) |
| char | g\_str [255] |

---

## Detailed Description

**PopulationManager.cpp This is the code file for the population manager and associated classes**

by Chris J. Topping   
Version of 23rd July 2003   
All rights reserved.   
  
With additions as noted in:   
January 2008   
Doxygen formatted comments in May 2008

---

## Macro Definition Documentation

|  |
| --- |
| #define \_CRTDBG\_MAP\_ALLOC |

---

## Variable Documentation

|  |
| --- |
| CfgInt cfg\_CatastropheEventStartYear("PM\_CATASTROPHEEVENTSTARTYEAR", CFG\_CUSTOM, 99999) |

Referenced by Population\_Manager::Run().

|  |
| --- |
| CfgInt cfg\_CIPEGridOutput\_day("G\_CIPEGRIDOUTPUT\_DAY", CFG\_CUSTOM, 1) |

Referenced by Population\_Manager::Run().

|  |
| --- |
| CfgInt cfg\_CIPEGridOutput\_day\_b("G\_CIPEGRIDOUTPUT\_DAY\_B", CFG\_CUSTOM, 270) |

|  |
| --- |
| CfgStr cfg\_CIPEGridOutput\_filename("G\_CIPEGRIDOUTPUT\_FILENAME", CFG\_CUSTOM,"CIPEGridOutput.txt") |

Referenced by Population\_Manager::OpenTheCIPEGridOutputProbe().

|  |
| --- |
| CfgStr cfg\_CIPEGridOutput\_filenameB("G\_CIPEGRIDOUTPUT\_FILENAME\_B", CFG\_CUSTOM,"CIPEGridOutputB.txt") |

Referenced by Population\_Manager::OpenTheCIPEGridOutputProbe().

|  |
| --- |
| CfgInt cfg\_CIPEGridOutput\_Interval("G\_CIPEGRIDOUTPUT\_INTERVAL", CFG\_CUSTOM, 1) |

Referenced by Population\_Manager::Run().

|  |
| --- |
| CfgBool cfg\_CIPEGridOutput\_used("G\_CIPEGRIDOUTPUT\_USED", CFG\_CUSTOM, false) |

Referenced by Population\_Manager::Run().

|  |  |  |
| --- | --- | --- |
| |  | | --- | | CfgInt cfg\_CipeGridSize("G\_CIPEGRIDSIZE", CFG\_CUSTOM, 500) | | static |

Referenced by Population\_Manager::Population\_Manager().

|  |  |  |
| --- | --- | --- |
| |  | | --- | | CfgInt cfg\_DayInMonth("PRB\_DAYINMONTH", CFG\_CUSTOM, 1) | | static |

Referenced by Population\_Manager::BeginningOfMonth().

|  |
| --- |
| CfgBool cfg\_fixed\_random\_sequence("G\_FIXEDRANDOMSEQUENCE", CFG\_CUSTOM, false) |

|  |
| --- |
| CfgInt cfg\_pm\_eventfrequency("PM\_EVENTFREQUENCY", CFG\_CUSTOM, 0) |

|  |
| --- |
| CfgInt cfg\_pm\_eventsize("PM\_EVENTSIZE", CFG\_CUSTOM, 100) |

|  |  |  |
| --- | --- | --- |
| |  | | --- | | CfgInt cfg\_ReallyBigOutput\_day1("G\_REALLYBIGOUTPUT\_DAY\_ONE", CFG\_CUSTOM, 1) | | static |

Referenced by Population\_Manager::Run().

|  |  |  |
| --- | --- | --- |
| |  | | --- | | CfgInt cfg\_ReallyBigOutput\_day2("G\_REALLYBIGOUTPUT\_DAY\_TWO", CFG\_CUSTOM, 91) | | static |

Referenced by Population\_Manager::Run().

|  |  |  |
| --- | --- | --- |
| |  | | --- | | CfgInt cfg\_ReallyBigOutput\_day3("G\_REALLYBIGOUTPUT\_DAY\_THREE", CFG\_CUSTOM, 182) | | static |

Referenced by Population\_Manager::Run().

|  |  |  |
| --- | --- | --- |
| |  | | --- | | CfgInt cfg\_ReallyBigOutput\_day4("G\_REALLYBIGOUTPUT\_DAY\_FOUR", CFG\_CUSTOM, 274) | | static |

Referenced by Population\_Manager::Run().

|  |  |  |
| --- | --- | --- |
| |  | | --- | | CfgStr cfg\_ReallyBigOutput\_filename("G\_REALLYBIGOUTPUT\_FILENAME", CFG\_CUSTOM,"ReallyBigOutput.txt") | | static |

Referenced by Population\_Manager::OpenTheReallyBigProbe().

|  |  |  |
| --- | --- | --- |
| |  | | --- | | CfgInt cfg\_ReallyBigOutput\_interval("G\_REALLYBIGOUTPUT\_INTERVAL", CFG\_CUSTOM, 1) | | static |

Referenced by Population\_Manager::Run().

|  |
| --- |
| CfgBool cfg\_ReallyBigOutput\_used("G\_REALLYBIGOUTPUT\_USED", CFG\_CUSTOM, false) |

Referenced by Population\_Manager::Run(), and Population\_Manager::~Population\_Manager().

|  |  |  |
| --- | --- | --- |
| |  | | --- | | CfgInt cfg\_ReallyBigOutputFirstYear("G\_REALLYBIGOUTPUT\_FIRSTYEAR", CFG\_CUSTOM, 1) | | static |

Referenced by Population\_Manager::Run().

|  |  |  |
| --- | --- | --- |
| |  | | --- | | CfgInt cfg\_RipleysOutput\_day("G\_RIPLEYSOUTPUT\_DAY", CFG\_CUSTOM, 60) | | static |

Referenced by Population\_Manager::Run().

|  |  |  |
| --- | --- | --- |
| |  | | --- | | CfgStr cfg\_RipleysOutput\_filename("G\_RIPLEYSOUTPUT\_FILENAME", CFG\_CUSTOM,"RipleysOutput.txt") | | static |

Referenced by Population\_Manager::OpenTheMonthlyRipleysOutputProbe(), and Population\_Manager::OpenTheRipleysOutputProbe().

|  |  |  |
| --- | --- | --- |
| |  | | --- | | CfgInt cfg\_RipleysOutput\_interval("G\_RIPLEYSOUTPUT\_INTERVAL", CFG\_CUSTOM, 1) | | static |

Referenced by Population\_Manager::Run().

|  |
| --- |
| CfgBool cfg\_RipleysOutput\_used("G\_RIPLEYSOUTPUT\_USED", CFG\_CUSTOM, true) |

Referenced by Population\_Manager::Run(), and Population\_Manager::~Population\_Manager().

|  |  |  |
| --- | --- | --- |
| |  | | --- | | CfgInt cfg\_RipleysOutputFirstYear("G\_RIPLEYSOUTPUT\_FIRSTYEAR", CFG\_CUSTOM, 1) | | static |

Referenced by Population\_Manager::Run().

|  |
| --- |
| CfgBool cfg\_RipleysOutputMonthly\_used("G\_RIPLEYSOUTPUTMONTHLY\_USED", CFG\_CUSTOM, false) |

Referenced by Population\_Manager::CloseTheRipleysOutputProbe(), Population\_Manager::Population\_Manager(), Population\_Manager::Run(), and Population\_Manager::~Population\_Manager().

|  |  |  |
| --- | --- | --- |
| |  | | --- | | CfgInt cfg\_VoleCatastrophe\_interval("VOLE\_CATASTROPHE\_I", CFG\_CUSTOM, 365 \*5) | | static |

|  |  |  |
| --- | --- | --- |
| |  | | --- | | CfgInt cfg\_VoleCatastrophe\_mortality("VOLE\_CATASTROPHE\_M", CFG\_CUSTOM, 90) | | static |

|  |  |  |
| --- | --- | --- |
| |  | | --- | | CfgBool cfg\_VoleCatastrophe\_on("VOLE\_CATASTROPHE\_ON", CFG\_CUSTOM, false) | | static |

|  |
| --- |
| char g\_str[255] |

Referenced by Population\_Manager::ProbeReport(), Population\_Manager::ProbeReportTimed(), and Population\_Manager::SpeciesSpecificReporting().


- CJT
- MSVC
- ALMaSS Working Source
- BatchALMaSS
- PopulationManager.cpp
- Generated on Thu Jan 10 2013 13:15:35 for ALMaSS Skylark ODdox by
   1.8.1.1
