# Supplementary material for: Modelling Skylarks (Alauda arvensis) to Predict Impacts of Changes in Land Management and Policy: Development and Testing of an Agent-Based Model
Source: PLoS One. 2013 Jun 6;8(6):e65803. doi: 10.1371/journal.pone.0065803 (PMC3675089; doi:10.1371/journal.pone.0065803)
Supplement: Supporting Information S4 — The skylark ODdox as a zipped archive. (ZIP) [file pone.0065803.s004.zip › Skylark_ODdox/_position_map_8cpp.html]

ALMaSS Skylark ODdox: PositionMap.cpp File Reference


|  |
| --- |
| ALMaSS Skylark ODdox  2.0 |


- Main Page
- Related Pages
- Classes
- Files

- File List
- File Members

Macros

PositionMap.cpp File Reference

**PositionMap.cpp This file contains the source for the PositionMap class**   
More...

`#include "ALMaSS_Setup.h"`  
`#include "../Landscape/ls.h"`  
`#include "../Landscape/tole_declaration.h"`  
`#include "../Landscape/maperrormsg.h"`  
`#include "../BatchALMaSS/positionmap.h"`

|  |  |
| --- | --- |
| Macros | |
| #define | \_\_3264divide   5 |
| #define | \_\_3264minus0   32 |
| #define | \_\_3264minus1   31 |

---

## Detailed Description

**PositionMap.cpp This file contains the source for the PositionMap class**

by Chris J. Topping   
Version of June 2003   
  
Doxygen formatted comments in July 2008

---

## Macro Definition Documentation

|  |
| --- |
| #define \_\_3264divide   5 |

Referenced by PositionMap::ClearMapValue(), PositionMap::GetMapDensity(), PositionMap::GetMapDensity32(), PositionMap::GetMapDensity5x5(), PositionMap::GetMapPositive(), PositionMap::GetMapPositiveB(), PositionMap::GetMapValue(), and PositionMap::SetMapValue().

|  |
| --- |
| #define \_\_3264minus0   32 |

Referenced by PositionMap::GetMapDensity(), PositionMap::GetMapPositive(), PositionMap::Init(), and PositionMap::PositionMap().

|  |
| --- |
| #define \_\_3264minus1   31 |

Referenced by PositionMap::ClearMapValue(), PositionMap::GetMapDensity(), PositionMap::GetMapDensity32(), PositionMap::GetMapDensity5x5(), PositionMap::GetMapPositive(), PositionMap::GetMapPositiveB(), PositionMap::GetMapValue(), PositionMap::PositionMap(), and PositionMap::SetMapValue().


- CJT
- MSVC
- ALMaSS Working Source
- BatchALMaSS
- PositionMap.cpp
- Generated on Thu Jan 10 2013 13:15:35 for ALMaSS Skylark ODdox by
   1.8.1.1
