# Supplementary material for: Modelling Skylarks (Alauda arvensis) to Predict Impacts of Changes in Land Management and Policy: Development and Testing of an Agent-Based Model
Source: PLoS One. 2013 Jun 6;8(6):e65803. doi: 10.1371/journal.pone.0065803 (PMC3675089; doi:10.1371/journal.pone.0065803)
Supplement: Supporting Information S4 — The skylark ODdox as a zipped archive. (ZIP) [file pone.0065803.s004.zip › Skylark_ODdox/_rodenticide_8cpp.html]

ALMaSS Skylark ODdox: Rodenticide.cpp File Reference


|  |
| --- |
| ALMaSS Skylark ODdox  2.0 |


- Main Page
- Related Pages
- Classes
- Files

- File List
- File Members

Variables

Rodenticide.cpp File Reference

`#include <iostream>`  
`#include <fstream>`  
`#include <list>`  
`#include <string>`  
`#include "../ALMaSSDefines.h"`  
`#include "../Landscape/ls.h"`  
`#include "../BatchALMaSS/BoostRandomGenerators.h"`

|  |  |
| --- | --- |
| Variables | |
| CfgFloat | cfg\_rodenticide\_BLcountry\_AnnFreq ("RODENTICIDE\_BLTYPE\_COUNTRY\_ANNFREQ", CFG\_CUSTOM, 0.50) |
| CfgInt | cfg\_rodenticide\_BLcountry\_Length ("RODENTICIDE\_BLTYPE\_COUNTRY\_LENGTH", CFG\_CUSTOM, 365) |
| CfgFloat | cfg\_rodenticide\_BLtown\_AnnFreq ("RODENTICIDE\_BLTYPE\_TOWN\_ANNFREQ", CFG\_CUSTOM, 0.25) |
| CfgInt | cfg\_rodenticide\_BLtown\_Length ("RODENTICIDE\_BLTYPE\_TOWN\_LENGTH", CFG\_CUSTOM, 28) |
| CfgFloat | cfg\_rodenticide\_BLwoodland\_AnnFreq ("RODENTICIDE\_BLTYPE\_WOODLAND\_ANNFREQ", CFG\_CUSTOM, 1.00) |
| CfgInt | cfg\_rodenticide\_BLwoodland\_Length ("RODENTICIDE\_BLTYPE\_WOODLAND\_LENGTH", CFG\_CUSTOM, 180) |
| CfgFloat | cfg\_rodenticide\_deathRate ("RODENTICIDE\_DEATHRATE", CFG\_CUSTOM, 0.001) |
| CfgFloat | cfg\_rodenticide\_diffusionRate ("RODENTICIDE\_DIFFUSIONRATE", CFG\_CUSTOM, 0.5) |
| CfgInt | cfg\_rodenticide\_dumpmapday ("RODENTICIDE\_DUMPMAPDAY", CFG\_CUSTOM, 180) |
| CfgInt | cfg\_rodenticide\_dumpmapendyear ("RODENTICIDE\_DUMPMAPENDYEAR", CFG\_CUSTOM,-1) |
| CfgInt | cfg\_rodenticide\_dumpmapstartyear ("RODENTICIDE\_DUMPMAPSTARTYEAR", CFG\_CUSTOM, 99999) |
| CfgInt | cfg\_rodenticide\_dumppolyendyear ("RODENTICIDE\_DUMPPOLYENDYEAR", CFG\_CUSTOM,-1) |
| CfgInt | cfg\_rodenticide\_dumppolyinterval ("RODENTICIDE\_DUMPPOLYINTERVAL", CFG\_CUSTOM, 180) |
| CfgInt | cfg\_rodenticide\_dumppolystartyear ("RODENTICIDE\_DUMPPOLYSTARTYEAR", CFG\_CUSTOM, 99999) |
| CfgInt | cfg\_rodenticide\_gridsize ("RODENTICIDE\_GRIDSIZE", CFG\_CUSTOM, 2) |
| CfgFloat | cfg\_rodenticide\_immigrationRate ("RODENTICIDE\_IMMIGRATIONRATE", CFG\_CUSTOM, 0.56) |
| boost::variate\_generator  < base\_generator\_type   &, boost::uniform\_real<> > | g\_rand\_uni |

---

## Variable Documentation

|  |
| --- |
| CfgFloat cfg\_rodenticide\_BLcountry\_AnnFreq("RODENTICIDE\_BLTYPE\_COUNTRY\_ANNFREQ", CFG\_CUSTOM, 0.50) |

The annual probability of bait placement at a farm/rural house location.

Referenced by RodenticideManager::ShouldPlaceBait().

|  |
| --- |
| CfgInt cfg\_rodenticide\_BLcountry\_Length("RODENTICIDE\_BLTYPE\_COUNTRY\_LENGTH", CFG\_CUSTOM, 365) |

The length of time in days that bait is available after placement in a rural building location.

Referenced by RodenticideManager::DoPlaceBait().

|  |
| --- |
| CfgFloat cfg\_rodenticide\_BLtown\_AnnFreq("RODENTICIDE\_BLTYPE\_TOWN\_ANNFREQ", CFG\_CUSTOM, 0.25) |

The annual probability of bait placement at a town location.

Referenced by RodenticideManager::ShouldPlaceBait().

|  |
| --- |
| CfgInt cfg\_rodenticide\_BLtown\_Length("RODENTICIDE\_BLTYPE\_TOWN\_LENGTH", CFG\_CUSTOM, 28) |

The length of time in days that bait is available after placement in a town location.

Referenced by RodenticideManager::DoPlaceBait().

|  |
| --- |
| CfgFloat cfg\_rodenticide\_BLwoodland\_AnnFreq("RODENTICIDE\_BLTYPE\_WOODLAND\_ANNFREQ", CFG\_CUSTOM, 1.00) |

The annual probability of bait placement at a woodland location.

Referenced by RodenticideManager::ShouldPlaceBait().

|  |
| --- |
| CfgInt cfg\_rodenticide\_BLwoodland\_Length("RODENTICIDE\_BLTYPE\_WOODLAND\_LENGTH", CFG\_CUSTOM, 180) |

The length of time in days that bait is available after placement in a woodland location.

Referenced by RodenticideManager::DoPlaceBait().

|  |
| --- |
| CfgFloat cfg\_rodenticide\_deathRate("RODENTICIDE\_DEATHRATE", CFG\_CUSTOM, 0.001) |

The rate of death of poisoned mice. This is assumed to be a constant for simplicity and also determines the distance to which the mice can disperse when poisoned. It is not the death rate of the mice themselves!

Referenced by RodenticideManager::RodenticideManager().

|  |
| --- |
| CfgFloat cfg\_rodenticide\_diffusionRate("RODENTICIDE\_DIFFUSIONRATE", CFG\_CUSTOM, 0.5) |

The rate of diffusion of the mice per day assumed. Mice diffuse from each cell to surrounding cells as a proportion per time step, this proportion is given by this parameter. High diffusion rates lead to longer distances but lower densities.

Referenced by RodenticideManager::RodenticideManager().

|  |
| --- |
| CfgInt cfg\_rodenticide\_dumpmapday("RODENTICIDE\_DUMPMAPDAY", CFG\_CUSTOM, 180) |

The day in the year to dump the rodenticide map.

Referenced by RodenticideManager::Tick().

|  |
| --- |
| CfgInt cfg\_rodenticide\_dumpmapendyear("RODENTICIDE\_DUMPMAPENDYEAR", CFG\_CUSTOM,-1) |

The last year to dump the rodenticide map.

|  |
| --- |
| CfgInt cfg\_rodenticide\_dumpmapstartyear("RODENTICIDE\_DUMPMAPSTARTYEAR", CFG\_CUSTOM, 99999) |

The first year to dump the rodenticide map.

Referenced by RodenticideManager::Tick().

|  |
| --- |
| CfgInt cfg\_rodenticide\_dumppolyendyear("RODENTICIDE\_DUMPPOLYENDYEAR", CFG\_CUSTOM,-1) |

The last year to dump the rodenticide polygon information. Note that defaults are set so as not to dump this information.

Referenced by RodenticideManager::Tick().

|  |
| --- |
| CfgInt cfg\_rodenticide\_dumppolyinterval("RODENTICIDE\_DUMPPOLYINTERVAL", CFG\_CUSTOM, 180) |

The interval of days during the year after which to dump the polygon information. 180 will result in 3 dumps, day 0 day 180 and day 360.

Referenced by RodenticideManager::Tick().

|  |
| --- |
| CfgInt cfg\_rodenticide\_dumppolystartyear("RODENTICIDE\_DUMPPOLYSTARTYEAR", CFG\_CUSTOM, 99999) |

The first year to dump the rodenticide polygon information. Note that defaults are set so as not to dump this information.

Referenced by RodenticideManager::Tick().

|  |
| --- |
| CfgInt cfg\_rodenticide\_gridsize("RODENTICIDE\_GRIDSIZE", CFG\_CUSTOM, 2) |

This is the power of 2 to raise the grid size to, so 0 = 1x1m, 1 = 2x2m 2 = 4x4m etc..

Referenced by RodenticideManager::RodenticideManager().

|  |
| --- |
| CfgFloat cfg\_rodenticide\_immigrationRate("RODENTICIDE\_IMMIGRATIONRATE", CFG\_CUSTOM, 0.56) |

The rate of immigration to the baitl area assumed. This parameter is used to ensure that the population of mice does not simply die out after a few days as if forms a travelling wave from the bait. If this is set to non-zero then the immigration rate will determine the extent to which the mice will reach (distance from bait) under stable bait replacement conditions.

Referenced by RodenticideManager::RodenticideManager().

|  |
| --- |
| boost::variate\_generator<base\_generator\_type&, boost::uniform\_real<> > g\_rand\_uni |

Referenced by RodenticideManager::GetBaitStartDate(), SkTerritories::PreProcessLandscape2(), random(), RodenticideManager::ShouldPlaceBait(), Skylark\_Male::Skylark\_Male(), and Skylark\_Female::st\_Arriving().


- CJT
- MSVC
- ALMaSS Working Source
- Landscape
- Rodenticide.cpp
- Generated on Thu Jan 10 2013 13:15:35 for ALMaSS Skylark ODdox by
   1.8.1.1
