# Supplementary material for: Modelling Skylarks (Alauda arvensis) to Predict Impacts of Changes in Land Management and Policy: Development and Testing of an Agent-Based Model
Source: PLoS One. 2013 Jun 6;8(6):e65803. doi: 10.1371/journal.pone.0065803 (PMC3675089; doi:10.1371/journal.pone.0065803)
Supplement: Supporting Information S4 — The skylark ODdox as a zipped archive. (ZIP) [file pone.0065803.s004.zip › Skylark_ODdox/_rodenticide_8h.html]

ALMaSS Skylark ODdox: Rodenticide.h File Reference


|  |
| --- |
| ALMaSS Skylark ODdox  2.0 |


- Main Page
- Related Pages
- Classes
- Files

- File List
- File Members

Classes |
Enumerations

Rodenticide.h File Reference

|  |  |
| --- | --- |
| Classes | |
| class | BaitLocation |
|  | Class used for describing the rodenticide bait location. More... |
| class | RodenticideManager |
|  | Class for management of bait locations. More... |

|  |  |
| --- | --- |
| Enumerations | |
| enum | TTypesBaitLocation { tbl\_town = 0, tbl\_country, tbl\_woodland, tbl\_foobar } |
|  | An enumeration listing the types of bait locations possible. More... |

---

## Enumeration Type Documentation

|  |
| --- |
| enum TTypesBaitLocation |

An enumeration listing the types of bait locations possible.

**Enumerator:**
:   |  |  |
    | --- | --- |
    | *tbl\_town* |  |
    | *tbl\_country* |  |
    | *tbl\_woodland* |  |
    | *tbl\_foobar* |  |

{

tbl\_town=0,

tbl\_country,

tbl\_woodland,

tbl\_foobar

} TTypesBaitLocation;


- CJT
- MSVC
- ALMaSS Working Source
- Landscape
- Rodenticide.h
- Generated on Thu Jan 10 2013 13:15:35 for ALMaSS Skylark ODdox by
   1.8.1.1
