# Supplementary material for: Modelling Skylarks (Alauda arvensis) to Predict Impacts of Changes in Land Management and Policy: Development and Testing of an Agent-Based Model
Source: PLoS One. 2013 Jun 6;8(6):e65803. doi: 10.1371/journal.pone.0065803 (PMC3675089; doi:10.1371/journal.pone.0065803)
Supplement: Supporting Information S4 — The skylark ODdox as a zipped archive. (ZIP) [file pone.0065803.s004.zip › Skylark_ODdox/_skylark_configs_8cpp.html]

ALMaSS Skylark ODdox: SkylarkConfigs.cpp File Reference


|  |
| --- |
| ALMaSS Skylark ODdox  2.0 |


- Main Page
- Related Pages
- Classes
- Files

- File List
- File Members

Variables

SkylarkConfigs.cpp File Reference

`#include "../Landscape/ls.h"`

|  |  |
| --- | --- |
| Variables | |
| CfgInt | cfg\_adultreturnmort ("SK\_ADULTRETURNMORT", CFG\_CUSTOM, 35) |
|  | Immigration mortality for juveniles. |
| CfgInt | cfg\_Breed\_Res\_Thresh1 ("SK\_BREED\_RES\_THRESH", CFG\_CUSTOM, 600) |
| CfgInt | cfg\_ClutchMortProb ("SK\_CLUTCH\_MORT\_PROB", CFG\_CUSTOM, 165) |
| CfgFloat | cfg\_ConversionEffReduc ("SK\_CONVEFFREDEC", CFG\_CUSTOM, 0.023077) |
| CfgFloat | cfg\_Cooling\_Rate\_Eggs ("SK\_COOLING\_RATE\_EGGS", CFG\_CUSTOM, 2.9) |
|  | Maximum immigration mortality for females. |
| CfgFloat | cfg\_densityconstant\_a ("SK\_DENSITYCONST\_A", CFG\_CUSTOM, 0) |
| CfgFloat | cfg\_densityconstant\_b ("SK\_DENSITYCONST\_B", CFG\_CUSTOM,-0.1) |
| CfgInt | cfg\_densityconstant\_c ("SK\_DENSITYCONST\_C", CFG\_CUSTOM, 1) |
| CfgFloat | cfg\_EggTemp ("SK\_EGGTEMP", CFG\_CUSTOM, 32.0) |
| CfgFloat | cfg\_EM\_Nestling\_a ("SK\_EM\_NESTLING\_A", CFG\_CUSTOM, 0.8542) |
| CfgFloat | cfg\_EM\_Nestling\_b ("SK\_EM\_NESTLING\_B", CFG\_CUSTOM, 1.353) |
| CfgInt | cfg\_fecundity\_reduc ("SK\_FECUNDITY\_REDUC", CFG\_CUSTOM, 0) |
| CfgInt | cfg\_fecundity\_reduc\_chance ("SK\_FECUNDITY\_REDUC\_CHANCE", CFG\_CUSTOM, 0) |
| CfgFloat | cfg\_FemaleMinTerritoryAcceptScore ("SK\_MINFEMACCEPTSCORE", CFG\_CUSTOM, 300000) |
| CfgInt | cfg\_FoodTripsPerDay ("SK\_FOOD\_TRIPS\_PER\_DAY", CFG\_CUSTOM, 30) |
| CfgFloat | cfg\_heightconstant\_a ("SK\_HEIGHTCONST\_A", CFG\_CUSTOM, 1) |
| CfgFloat | cfg\_heightconstant\_b ("SK\_HEIGHTCONST\_B", CFG\_CUSTOM,-0.1) |
| CfgInt | cfg\_heightconstant\_c ("SK\_HEIGHTCONST\_C", CFG\_CUSTOM, 1) |
| CfgFloat | cfg\_hindconstantD\_b ("SK\_HINDCONSTD\_B", CFG\_CUSTOM,-0.1) |
| CfgFloat | cfg\_hindconstantH\_b ("SK\_HINDCONSTH\_B", CFG\_CUSTOM, 1) |
| CfgInt | cfg\_HQualityBareEarth ("SK\_HQBAREEARTH", CFG\_CUSTOM, 11) |
| CfgInt | cfg\_HQualityHedge ("SK\_HQHEDGE", CFG\_CUSTOM,-1050) |
| CfgInt | cfg\_HQualityHedgeScrub ("SK\_HQHEDGESCRUB", CFG\_CUSTOM, 0) |
| CfgInt | cfg\_HQualityMetalRoad ("SK\_HQMETALROAD", CFG\_CUSTOM,-10) |
| CfgInt | cfg\_HQualityNeutral ("SK\_HQNEUTRAL", CFG\_CUSTOM, 0) |
| CfgInt | cfg\_HQualityOpenTallVeg ("SK\_HQOOPENTALLVEG", CFG\_CUSTOM, 16) |
| CfgInt | cfg\_HQualityTall ("SK\_HQTALL", CFG\_CUSTOM, 0) |
| CfgInt | cfg\_HQualityTall2 ("SK\_HQTALLVEGTWO", CFG\_CUSTOM,-1000) |
| CfgInt | cfg\_HQualityTallVeg ("SK\_HQTALLVEG", CFG\_CUSTOM, 0) |
| CfgInt | cfg\_HQualityTrack ("SK\_HQTRACK", CFG\_CUSTOM, 10) |
| CfgInt | cfg\_HQualityVeg30cm ("SK\_HQVEGTHIRTYCM", CFG\_CUSTOM, 11) |
| CfgInt | cfg\_HQualityWater ("SK\_HQWATER", CFG\_CUSTOM, 0) |
| CfgInt | cfg\_insecticide\_direct\_mortE ("SK\_INSECTICDEDIRECTMORTE", CFG\_CUSTOM, 0) |
| CfgInt | cfg\_insecticide\_direct\_mortF ("SK\_INSECTICDEDIRECTMORTF", CFG\_CUSTOM, 0) |
| CfgInt | cfg\_insecticide\_direct\_mortM ("SK\_INSECTICDEDIRECTMORTM", CFG\_CUSTOM, 0) |
| CfgInt | cfg\_insecticide\_direct\_mortN ("SK\_INSECTICDEDIRECTMORTN", CFG\_CUSTOM, 0) |
| CfgInt | cfg\_insecticide\_direct\_mortP ("SK\_INSECTICDEDIRECTMORTP", CFG\_CUSTOM, 0) |
| CfgInt | cfg\_juvreturnmort ("SK\_JUVRETURNMORT", CFG\_CUSTOM, 35) |
|  | Immigration mortality for juveniles. |
| CfgFloat | cfg\_MaleSplitScale ("SK\_MALESPLITSCALE", CFG\_CUSTOM, 0.5) |
| CfgFloat | cfg\_maxfeedrain ("SK\_MAXFEEDRAIN", CFG\_CUSTOM, 10.0) |
| CfgFloat | cfg\_MD\_Threshold ("SK\_MD\_THRESHOLD", CFG\_CUSTOM, 26.0) |
| CfgFloat | cfg\_MeanExtractionRatePerMinute ("SK\_EXTRACTION\_RATE", CFG\_CUSTOM, 0.00125) |
| CfgFloat | cfg\_MeanHatchingWeight ("SK\_MEAN\_HATCHING\_WEIGHT", CFG\_CUSTOM, 3.23) |
| CfgFloat | cfg\_MinDaysToHatch ("SK\_MINDAYSTOHATCH", CFG\_CUSTOM, 10.5) |
|  | The time taken to egg hatch under optimal conditions. |
| CfgInt | cfg\_NestLeavingChance ("SK\_NESTLEAVECHANCE", CFG\_CUSTOM, 20) |
| CfgFloat | cfg\_NestLeavingWeight ("SK\_NEST\_LEAVING\_WEIGHT", CFG\_CUSTOM, 20.5) |
| CfgInt | cfg\_NestlingMortProb ("SK\_NEST\_MORT\_PROB", CFG\_CUSTOM, 115) |
| CfgFloat | cfg\_NestPlacementMinQual ("SK\_NESTPLACEMENTMINQUAL", CFG\_CUSTOM, 15) |
| CfgInt | cfg\_PatchyPremium ("SK\_PATCHYPREMIUM", CFG\_CUSTOM, 10) |
| CfgFloat | cfg\_PEmax ("SK\_PEMAX", CFG\_CUSTOM, 4.55) |
| CfgInt | cfg\_PreFledgeMortProb ("SK\_PREFLEDGE\_MORT\_PROB", CFG\_CUSTOM, 50) |
| CfgInt | cfg\_rainhindpow ("SK\_RAINHINDPOW", CFG\_CUSTOM, 4) |
| CfgFloat | cfg\_sk\_triplength ("SK\_TRIPLENGTH", CFG\_CUSTOM, 10) |
| CfgFloat | cfg\_SkScrapesPremiumII ("SK\_SKSCRAPESPREMIUMNEST", CFG\_CUSTOM, 5) |
| CfgInt | cfg\_SkStartNos ("SK\_STARTNOS", CFG\_CUSTOM, 6000) |
|  | The number of skylarks that start in the simulation. |
| CfgInt | cfg\_SkTramlinesPremium ("SK\_TRAMLINEPREMIUM", CFG\_CUSTOM, 5) |
| CfgFloat | cfg\_Skylark\_female\_Biodegredation ("SK\_FEMALE\_BIODEG", CFG\_CUSTOM, 0.0) |
|  | The proportion of pesticide remaining from one day to the next for females. |
| CfgFloat | cfg\_Skylark\_female\_NOEL ("SK\_FEMALE\_NOEL", CFG\_CUSTOM, 0.001) |
|  | Can be used to trigger a response to pesticides for the females. |
| CfgFloat | cfg\_Skylark\_male\_Biodegredation ("SK\_MALE\_BIODEG", CFG\_CUSTOM, 0.0) |
|  | The proportion of pesticide remaining from one day to the next for males. |
| CfgFloat | cfg\_Skylark\_male\_NOEL ("SK\_MALE\_NOEL", CFG\_CUSTOM, 0.001) |
|  | Can be used to trigger a response to pesticides for the males. |
| CfgFloat | cfg\_Skylark\_nestling\_Biodegredation ("SK\_NESTLING\_BIODEG", CFG\_CUSTOM, 0.0) |
|  | The proportion of pesticide accumulated from one day to the next. |
| CfgFloat | cfg\_Skylark\_nestling\_NOEL ("SK\_NESTLING\_NOEL", CFG\_CUSTOM, 0.001) |
|  | Can be used to trigger a response to pesticides for the nestlings. |
| CfgFloat | cfg\_Skylark\_prefledegling\_Biodegredation ("SK\_FLEDGE\_BIODEG", CFG\_CUSTOM, 0.0) |
|  | The proportion of pesticide remaining from one day to the next for prefledglings. |
| CfgFloat | cfg\_Skylark\_prefledegling\_NOEL ("SK\_FLEDGE\_NOEL", CFG\_CUSTOM, 0.001) |
|  | Used for determining the pesticide response for prefledglings. |
| CfgInt | cfg\_strigling\_clutch ("SK\_STRIGLING\_C", CFG\_CUSTOM, 72) |
| CfgInt | cfg\_strigling\_nestling ("SK\_STRIGLING\_N", CFG\_CUSTOM, 72) |
| CfgInt | cfg\_strigling\_preflg ("SK\_STRIGLING\_PF", CFG\_CUSTOM, 72) |
| CfgInt | cfg\_temphindpow ("SK\_TEMPHINDPOW", CFG\_CUSTOM, 3) |
| CfgFloat | cfg\_tramline\_foraging ("SK\_TRAMLINE\_FORAGING\_PROP", CFG\_CUSTOM, 0.45) |

---

## Variable Documentation

|  |
| --- |
| CfgInt cfg\_adultreturnmort("SK\_ADULTRETURNMORT", CFG\_CUSTOM, 35) |

Immigration mortality for juveniles.

|  |
| --- |
| CfgInt cfg\_Breed\_Res\_Thresh1("SK\_BREED\_RES\_THRESH", CFG\_CUSTOM, 600) |

|  |
| --- |
| CfgInt cfg\_ClutchMortProb("SK\_CLUTCH\_MORT\_PROB", CFG\_CUSTOM, 165) |

|  |
| --- |
| CfgFloat cfg\_ConversionEffReduc("SK\_CONVEFFREDEC", CFG\_CUSTOM, 0.023077) |

|  |
| --- |
| CfgFloat cfg\_Cooling\_Rate\_Eggs("SK\_COOLING\_RATE\_EGGS", CFG\_CUSTOM, 2.9) |

Maximum immigration mortality for females.

Minimum immigration mortality for females Maximum immigration mortality for males Minimum immigration mortality for males

|  |
| --- |
| CfgFloat cfg\_densityconstant\_a("SK\_DENSITYCONST\_A", CFG\_CUSTOM, 0) |

|  |
| --- |
| CfgFloat cfg\_densityconstant\_b("SK\_DENSITYCONST\_B", CFG\_CUSTOM,-0.1) |

|  |
| --- |
| CfgInt cfg\_densityconstant\_c("SK\_DENSITYCONST\_C", CFG\_CUSTOM, 1) |

|  |
| --- |
| CfgFloat cfg\_EggTemp("SK\_EGGTEMP", CFG\_CUSTOM, 32.0) |

|  |
| --- |
| CfgFloat cfg\_EM\_Nestling\_a("SK\_EM\_NESTLING\_A", CFG\_CUSTOM, 0.8542) |

|  |
| --- |
| CfgFloat cfg\_EM\_Nestling\_b("SK\_EM\_NESTLING\_B", CFG\_CUSTOM, 1.353) |

|  |
| --- |
| CfgInt cfg\_fecundity\_reduc("SK\_FECUNDITY\_REDUC", CFG\_CUSTOM, 0) |

|  |
| --- |
| CfgInt cfg\_fecundity\_reduc\_chance("SK\_FECUNDITY\_REDUC\_CHANCE", CFG\_CUSTOM, 0) |

|  |
| --- |
| CfgFloat cfg\_FemaleMinTerritoryAcceptScore("SK\_MINFEMACCEPTSCORE", CFG\_CUSTOM, 300000) |

|  |
| --- |
| CfgInt cfg\_FoodTripsPerDay("SK\_FOOD\_TRIPS\_PER\_DAY", CFG\_CUSTOM, 30) |

|  |
| --- |
| CfgFloat cfg\_heightconstant\_a("SK\_HEIGHTCONST\_A", CFG\_CUSTOM, 1) |

|  |
| --- |
| CfgFloat cfg\_heightconstant\_b("SK\_HEIGHTCONST\_B", CFG\_CUSTOM,-0.1) |

|  |
| --- |
| CfgInt cfg\_heightconstant\_c("SK\_HEIGHTCONST\_C", CFG\_CUSTOM, 1) |

|  |
| --- |
| CfgFloat cfg\_hindconstantD\_b("SK\_HINDCONSTD\_B", CFG\_CUSTOM,-0.1) |

|  |
| --- |
| CfgFloat cfg\_hindconstantH\_b("SK\_HINDCONSTH\_B", CFG\_CUSTOM, 1) |

|  |
| --- |
| CfgInt cfg\_HQualityBareEarth("SK\_HQBAREEARTH", CFG\_CUSTOM, 11) |

|  |
| --- |
| CfgInt cfg\_HQualityHedge("SK\_HQHEDGE", CFG\_CUSTOM,-1050) |

|  |
| --- |
| CfgInt cfg\_HQualityHedgeScrub("SK\_HQHEDGESCRUB", CFG\_CUSTOM, 0) |

|  |
| --- |
| CfgInt cfg\_HQualityMetalRoad("SK\_HQMETALROAD", CFG\_CUSTOM,-10) |

|  |
| --- |
| CfgInt cfg\_HQualityNeutral("SK\_HQNEUTRAL", CFG\_CUSTOM, 0) |

|  |
| --- |
| CfgInt cfg\_HQualityOpenTallVeg("SK\_HQOOPENTALLVEG", CFG\_CUSTOM, 16) |

|  |
| --- |
| CfgInt cfg\_HQualityTall("SK\_HQTALL", CFG\_CUSTOM, 0) |

|  |
| --- |
| CfgInt cfg\_HQualityTall2("SK\_HQTALLVEGTWO", CFG\_CUSTOM,-1000) |

|  |
| --- |
| CfgInt cfg\_HQualityTallVeg("SK\_HQTALLVEG", CFG\_CUSTOM, 0) |

|  |
| --- |
| CfgInt cfg\_HQualityTrack("SK\_HQTRACK", CFG\_CUSTOM, 10) |

|  |
| --- |
| CfgInt cfg\_HQualityVeg30cm("SK\_HQVEGTHIRTYCM", CFG\_CUSTOM, 11) |

|  |
| --- |
| CfgInt cfg\_HQualityWater("SK\_HQWATER", CFG\_CUSTOM, 0) |

|  |
| --- |
| CfgInt cfg\_insecticide\_direct\_mortE("SK\_INSECTICDEDIRECTMORTE", CFG\_CUSTOM, 0) |

|  |
| --- |
| CfgInt cfg\_insecticide\_direct\_mortF("SK\_INSECTICDEDIRECTMORTF", CFG\_CUSTOM, 0) |

|  |
| --- |
| CfgInt cfg\_insecticide\_direct\_mortM("SK\_INSECTICDEDIRECTMORTM", CFG\_CUSTOM, 0) |

|  |
| --- |
| CfgInt cfg\_insecticide\_direct\_mortN("SK\_INSECTICDEDIRECTMORTN", CFG\_CUSTOM, 0) |

|  |
| --- |
| CfgInt cfg\_insecticide\_direct\_mortP("SK\_INSECTICDEDIRECTMORTP", CFG\_CUSTOM, 0) |

|  |
| --- |
| CfgInt cfg\_juvreturnmort("SK\_JUVRETURNMORT", CFG\_CUSTOM, 35) |

Immigration mortality for juveniles.

|  |
| --- |
| CfgFloat cfg\_MaleSplitScale("SK\_MALESPLITSCALE", CFG\_CUSTOM, 0.5) |

|  |
| --- |
| CfgFloat cfg\_maxfeedrain("SK\_MAXFEEDRAIN", CFG\_CUSTOM, 10.0) |

|  |
| --- |
| CfgFloat cfg\_MD\_Threshold("SK\_MD\_THRESHOLD", CFG\_CUSTOM, 26.0) |

|  |
| --- |
| CfgFloat cfg\_MeanExtractionRatePerMinute("SK\_EXTRACTION\_RATE", CFG\_CUSTOM, 0.00125) |

|  |
| --- |
| CfgFloat cfg\_MeanHatchingWeight("SK\_MEAN\_HATCHING\_WEIGHT", CFG\_CUSTOM, 3.23) |

|  |
| --- |
| CfgFloat cfg\_MinDaysToHatch("SK\_MINDAYSTOHATCH", CFG\_CUSTOM, 10.5) |

The time taken to egg hatch under optimal conditions.

|  |
| --- |
| CfgInt cfg\_NestLeavingChance("SK\_NESTLEAVECHANCE", CFG\_CUSTOM, 20) |

|  |
| --- |
| CfgFloat cfg\_NestLeavingWeight("SK\_NEST\_LEAVING\_WEIGHT", CFG\_CUSTOM, 20.5) |

|  |
| --- |
| CfgInt cfg\_NestlingMortProb("SK\_NEST\_MORT\_PROB", CFG\_CUSTOM, 115) |

|  |
| --- |
| CfgFloat cfg\_NestPlacementMinQual("SK\_NESTPLACEMENTMINQUAL", CFG\_CUSTOM, 15) |

|  |
| --- |
| CfgInt cfg\_PatchyPremium("SK\_PATCHYPREMIUM", CFG\_CUSTOM, 10) |

|  |
| --- |
| CfgFloat cfg\_PEmax("SK\_PEMAX", CFG\_CUSTOM, 4.55) |

|  |
| --- |
| CfgInt cfg\_PreFledgeMortProb("SK\_PREFLEDGE\_MORT\_PROB", CFG\_CUSTOM, 50) |

|  |
| --- |
| CfgInt cfg\_rainhindpow("SK\_RAINHINDPOW", CFG\_CUSTOM, 4) |

|  |
| --- |
| CfgFloat cfg\_sk\_triplength("SK\_TRIPLENGTH", CFG\_CUSTOM, 10) |

|  |
| --- |
| CfgFloat cfg\_SkScrapesPremiumII("SK\_SKSCRAPESPREMIUMNEST", CFG\_CUSTOM, 5) |

|  |
| --- |
| CfgInt cfg\_SkStartNos("SK\_STARTNOS", CFG\_CUSTOM, 6000) |

The number of skylarks that start in the simulation.

Parameters under control of the config file \*\*\*\*\*\*\*\*\*\*\*\*\*\*\*\*\*\*\*

|  |
| --- |
| CfgInt cfg\_SkTramlinesPremium("SK\_TRAMLINEPREMIUM", CFG\_CUSTOM, 5) |

|  |
| --- |
| CfgFloat cfg\_Skylark\_female\_Biodegredation("SK\_FEMALE\_BIODEG", CFG\_CUSTOM, 0.0) |

The proportion of pesticide remaining from one day to the next for females.

|  |
| --- |
| CfgFloat cfg\_Skylark\_female\_NOEL("SK\_FEMALE\_NOEL", CFG\_CUSTOM, 0.001) |

Can be used to trigger a response to pesticides for the females.

|  |
| --- |
| CfgFloat cfg\_Skylark\_male\_Biodegredation("SK\_MALE\_BIODEG", CFG\_CUSTOM, 0.0) |

The proportion of pesticide remaining from one day to the next for males.

|  |
| --- |
| CfgFloat cfg\_Skylark\_male\_NOEL("SK\_MALE\_NOEL", CFG\_CUSTOM, 0.001) |

Can be used to trigger a response to pesticides for the males.

|  |
| --- |
| CfgFloat cfg\_Skylark\_nestling\_Biodegredation("SK\_NESTLING\_BIODEG", CFG\_CUSTOM, 0.0) |

The proportion of pesticide accumulated from one day to the next.

|  |
| --- |
| CfgFloat cfg\_Skylark\_nestling\_NOEL("SK\_NESTLING\_NOEL", CFG\_CUSTOM, 0.001) |

Can be used to trigger a response to pesticides for the nestlings.

|  |
| --- |
| CfgFloat cfg\_Skylark\_prefledegling\_Biodegredation("SK\_FLEDGE\_BIODEG", CFG\_CUSTOM, 0.0) |

The proportion of pesticide remaining from one day to the next for prefledglings.

|  |
| --- |
| CfgFloat cfg\_Skylark\_prefledegling\_NOEL("SK\_FLEDGE\_NOEL", CFG\_CUSTOM, 0.001) |

Used for determining the pesticide response for prefledglings.

|  |
| --- |
| CfgInt cfg\_strigling\_clutch("SK\_STRIGLING\_C", CFG\_CUSTOM, 72) |

|  |
| --- |
| CfgInt cfg\_strigling\_nestling("SK\_STRIGLING\_N", CFG\_CUSTOM, 72) |

|  |
| --- |
| CfgInt cfg\_strigling\_preflg("SK\_STRIGLING\_PF", CFG\_CUSTOM, 72) |

|  |
| --- |
| CfgInt cfg\_temphindpow("SK\_TEMPHINDPOW", CFG\_CUSTOM, 3) |

|  |
| --- |
| CfgFloat cfg\_tramline\_foraging("SK\_TRAMLINE\_FORAGING\_PROP", CFG\_CUSTOM, 0.45) |


- CJT
- MSVC
- ALMaSS Working Source
- Skylark
- SkylarkConfigs.cpp
- Generated on Thu Jan 10 2013 13:15:35 for ALMaSS Skylark ODdox by
   1.8.1.1
