# Supplementary material for: Modelling Skylarks (Alauda arvensis) to Predict Impacts of Changes in Land Management and Policy: Development and Testing of an Agent-Based Model
Source: PLoS One. 2013 Jun 6;8(6):e65803. doi: 10.1371/journal.pone.0065803 (PMC3675089; doi:10.1371/journal.pone.0065803)
Supplement: Supporting Information S4 — The skylark ODdox as a zipped archive. (ZIP) [file pone.0065803.s004.zip › Skylark_ODdox/annotated.html]

ALMaSS Skylark ODdox: Class List


|  |
| --- |
| ALMaSS Skylark ODdox  2.0 |


- Main Page
- Related Pages
- Classes
- Files

- Class List
- Class Index
- Class Hierarchy
- Class Members

Class List

Here are the classes, structs, unions and interfaces with brief descriptions:

|  |  |
| --- | --- |
| **std** |  |
| ActivePit |  |
| aDate |  |
| AdultSkylark\_struct |  |
| AgroChemIndustryCerealFarm1 | Inbuilt special purpose farm type |
| AgroChemIndustryCerealFarm2 | Inbuilt special purpose farm type |
| AgroChemIndustryCerealFarm3 | Inbuilt special purpose farm type |
| AmenityGrass |  |
| AnimalPosition | A class defining an animals position |
| APoint | A struct defining an x,y coordinate set |
| BaitLocation | Class used for describing the rodenticide bait location |
| BareRock |  |
| BeetleBank |  |
| BinaryMapBase |  |
| Building |  |
| BuiltUpWithParkland |  |
| Calendar |  |
| CfgBase | Base class for a configurator entry |
| CfgBool | Bool configurator entry class |
| CfgFloat | Double configurator entry class |
| CfgInt | Integer configurator entry class |
| CfgStr | String configurator entry class |
| Coast |  |
| CompareFarmNum | Function class to compare to farm refs |
| CompareState | Function to compare to TAnimal's Current behavioural state |
| CompareStateR | Function to compare to TAnimal's CurrentStateNo |
| CompareX | Function to compare to TAnimal's m\_Location\_x |
| CompareY | Function to compare to TAnimal's m\_Location\_y |
| Configurator | A class to provide standard parameter entry facilities |
| ConiferousForest |  |
| ConventionalCattle | Inbuilt farm type |
| ConventionalPig | Inbuilt farm type |
| ConventionalPlant | Inbuilt farm type |
| ConvMarginalJord | Inbuilt special purpose farm type |
| Copse |  |
| Crop | The base class for all crops |
| CropData |  |
| CropGrowth |  |
| CropRotation |  |
| DeciduousForest |  |
| Diffusor |  |
| EdgeGrowingPoints |  |
| Edges |  |
| EGP\_Data |  |
| Farm | The base class for all farm types |
| FarmEvent | A struct to hold the information required to trigger a farm event |
| FarmManager | The Farm Manager class |
| Field |  |
| FieldBoundary |  |
| ForestElement |  |
| Freshwater |  |
| Garden |  |
| GreenElement |  |
| Heath |  |
| HedgeBank |  |
| Hedges |  |
| IDMap | Used to map locations of animals in space |
| IntArray100 | A struct of 100 ints |
| Landscape | The landscape class containing all environmental and topographical data |
| LargeRoad |  |
| LE |  |
| LE\_TypeClass |  |
| LowPriority | Used in event handling |
| LowPriPair | Used in event handling |
| MapErrorMsg |  |
| Marsh |  |
| MixedForest |  |
| MovementMap | Movement maps are used for rapid computing of animal movement |
| MovementMap16 | Movement maps are used for rapid computing of animal movement |
| NaturalGrass |  |
| Nestling\_struct |  |
| NonVegElement |  |
| NoPesticideBaseFarm | Inbuilt special purpose farm type |
| NoPesticideNoPFarm | Inbuilt special purpose farm type |
| Orchard |  |
| OrchardBand |  |
| OrchardGrass |  |
| OrganicCattle | A farm that can have its rotation defined by the user at runtime |
| OrganicPig | A farm that can have its rotation defined by the user at runtime |
| OrganicPlant | A farm that can have its rotation defined by the user at runtime |
| Parkland |  |
| PermanentSetaside |  |
| PermCropData | Used for storing permanent crop data for the farm rotation |
| PermPasture |  |
| PermPastureLowYield |  |
| PermPastureTussocky |  |
| Pesticide |  |
| PesticideEvent |  |
| PesticideTrialControl | Inbuilt special purpose farm type |
| PesticideTrialToxicControl | Inbuilt special purpose farm type |
| PesticideTrialTreatment | Inbuilt special purpose farm type |
| PitDisused |  |
| Point |  |
| PolygonDataVector |  |
| Population\_Manager | Base class for all population managers |
| PositionMap | Used to map locations of individuals for density estimates |
| PreFledgeling\_struct |  |
| priority\_queue |  |
| probe\_data | Data structure to hold & output probe data probe data is designed to be used to return the number of objects in a given area or areas in specific element or vegetation types or farms |
| Railway |  |
| RasterMap |  |
| rectangle | A struct defining two x,y coordinate sets of positive co-ords only |
| River |  |
| RiversidePlants |  |
| RiversideTrees |  |
| RoadsideVerge |  |
| RodenticideManager | Class for management of bait locations |
| RoeDeerInfo | Part of the basic ALMaSS system (obselete) |
| Rotation |  |
| RuralResidential |  |
| Saltwater |  |
| SandDune |  |
| ScalablePositionMap | Used to map locations of individuals for density estimates. Each cell can only contain one value (it is binary) |
| Scrub |  |
| SimplePositionMap | Used to map locations of individuals for density estimates - space inefficient but good for testing |
| skClutch\_struct |  |
| SkQualGrid |  |
| skSpiralResult |  |
| SkTerritories |  |
| skTerritory\_struct |  |
| skTTerritory |  |
| Skylark\_Adult |  |
| Skylark\_Base |  |
| Skylark\_Clutch |  |
| Skylark\_Female |  |
| Skylark\_Male |  |
| Skylark\_Nestling |  |
| Skylark\_Population\_Manager |  |
| Skylark\_PreFledgeling |  |
| Skylark\_struct |  |
| SmallRoad |  |
| Starter |  |
| StoneWall |  |
| Suburban |  |
| TALMaSSObject | The base class of all ALMaSS objects requiring Step code |
| TAnimal | The base class for all ALMaSS animal classes |
| tpct | Used for storing farmers field size vectors |
| Track |  |
| TSkylarkList |  |
| UnsprayedFieldMargin |  |
| Urban |  |
| UrbanNoVeg |  |
| UrbanPark |  |
| UserDefinedFarm | A farm that can have its rotation defined by the user at runtime |
| UserDefinedFarm1 |  |
| UserDefinedFarm10 | A farm that can have its rotation defined by the user at runtime |
| UserDefinedFarm11 | A farm that can have its rotation defined by the user at runtime |
| UserDefinedFarm12 | A farm that can have its rotation defined by the user at runtime |
| UserDefinedFarm13 | A farm that can have its rotation defined by the user at runtime |
| UserDefinedFarm14 | A farm that can have its rotation defined by the user at runtime |
| UserDefinedFarm15 | A farm that can have its rotation defined by the user at runtime |
| UserDefinedFarm16 | A farm that can have its rotation defined by the user at runtime |
| UserDefinedFarm17 | A farm that can have its rotation defined by the user at runtime |
| UserDefinedFarm18 | A farm that can have its rotation defined by the user at runtime |
| UserDefinedFarm19 | A farm that can have its rotation defined by the user at runtime |
| UserDefinedFarm2 | A farm that can have its rotation defined by the user at runtime |
| UserDefinedFarm20 | A farm that can have its rotation defined by the user at runtime |
| UserDefinedFarm21 | A farm that can have its rotation defined by the user at runtime |
| UserDefinedFarm22 | A farm that can have its rotation defined by the user at runtime |
| UserDefinedFarm23 | A farm that can have its rotation defined by the user at runtime |
| UserDefinedFarm24 | A farm that can have its rotation defined by the user at runtime |
| UserDefinedFarm25 | A farm that can have its rotation defined by the user at runtime |
| UserDefinedFarm26 | A farm that can have its rotation defined by the user at runtime |
| UserDefinedFarm27 | A farm that can have its rotation defined by the user at runtime |
| UserDefinedFarm28 | A farm that can have its rotation defined by the user at runtime |
| UserDefinedFarm29 | A farm that can have its rotation defined by the user at runtime |
| UserDefinedFarm3 | A farm that can have its rotation defined by the user at runtime |
| UserDefinedFarm30 | A farm that can have its rotation defined by the user at runtime |
| UserDefinedFarm31 | A farm that can have its rotation defined by the user at runtime |
| UserDefinedFarm32 | A farm that can have its rotation defined by the user at runtime |
| UserDefinedFarm33 | A farm that can have its rotation defined by the user at runtime |
| UserDefinedFarm34 | A farm that can have its rotation defined by the user at runtime |
| UserDefinedFarm35 | A farm that can have its rotation defined by the user at runtime |
| UserDefinedFarm36 | A farm that can have its rotation defined by the user at runtime |
| UserDefinedFarm4 | A farm that can have its rotation defined by the user at runtime |
| UserDefinedFarm5 | A farm that can have its rotation defined by the user at runtime |
| UserDefinedFarm6 | A farm that can have its rotation defined by the user at runtime |
| UserDefinedFarm7 | A farm that can have its rotation defined by the user at runtime |
| UserDefinedFarm8 | A farm that can have its rotation defined by the user at runtime |
| UserDefinedFarm9 | A farm that can have its rotation defined by the user at runtime |
| vector |  |
| VegElement |  |
| Weather |  |
| YoungForest |  |


- Generated on Thu Jan 10 2013 13:15:39 for ALMaSS Skylark ODdox by
   1.8.1.1
