# Supplementary material for: Modelling Skylarks (Alauda arvensis) to Predict Impacts of Changes in Land Management and Policy: Development and Testing of an Agent-Based Model
Source: PLoS One. 2013 Jun 6;8(6):e65803. doi: 10.1371/journal.pone.0065803 (PMC3675089; doi:10.1371/journal.pone.0065803)
Supplement: Supporting Information S4 — The skylark ODdox as a zipped archive. (ZIP) [file pone.0065803.s004.zip › Skylark_ODdox/calendar_8h.html]

ALMaSS Skylark ODdox: calendar.h File Reference


|  |
| --- |
| ALMaSS Skylark ODdox  2.0 |


- Main Page
- Related Pages
- Classes
- Files

- File List
- File Members

Classes |
Variables

calendar.h File Reference

|  |  |
| --- | --- |
| Classes | |
| struct | aDate |
| class | Calendar |

|  |  |
| --- | --- |
| Variables | |
| class Calendar \* | g\_date |

---

## Variable Documentation

|  |
| --- |
| class Calendar\* g\_date |

Referenced by Farm::BurnStrawStubble(), Farm::CattleIsOut(), Farm::CattleIsOutLow(), Farm::CheckRotationManagementLoop(), Farm::CutToHay(), VegElement::DoDevelopment(), RoadsideVerge::DoDevelopment(), UnsprayedFieldMargin::DoDevelopment(), Orchard::DoDevelopment(), OrchardBand::DoDevelopment(), OrchardGrass::DoDevelopment(), Skylark\_Population\_Manager::DoFirst(), Landscape::DumpMapInfoByArea(), Skylark\_Clutch::EndStep(), Skylark\_Nestling::EndStep(), Skylark\_PreFledgeling::EndStep(), VegElement::ForceGrowthInitialize(), VegElement::ForceGrowthTest(), Weather::GetSnow(), LargeRoad::GetTrafficLoad(), SmallRoad::GetTrafficLoad(), Farm::HandleEvents(), Farm::Harvest(), Farm::HayBailing(), Farm::HayTurning(), Farm::InitiateManagement(), Landscape::Landscape(), Skylark\_Clutch::OnFarmEvent(), Skylark\_Nestling::OnFarmEvent(), Skylark\_PreFledgeling::OnFarmEvent(), Skylark\_Female::OnFarmEvent(), Skylark\_Male::OnFarmEvent(), Skylark\_Clutch::OnMumGone(), VegElement::ReduceVeg\_Extended(), Farm::RowCultivation(), VegElement::SetGrowthPhase(), Skylark\_Female::Step(), Skylark\_Male::Step(), Farm::StrawChopping(), Farm::Strigling(), Farm::StriglingSow(), Farm::StubbleHarrowing(), Landscape::SupplyDayInMonth(), Landscape::SupplyDayInYear(), Landscape::SupplyDaylength(), Landscape::SupplyGlobalDate(), Landscape::SupplyMonth(), Landscape::SupplyWindDirection(), Landscape::SupplyYear(), Landscape::SupplyYearNumber(), Landscape::Tick(), Weather::Tick(), Landscape::TickHour(), Landscape::TickMinute(), Weather::Weather(), and Landscape::~Landscape().


- CJT
- MSVC
- ALMaSS Working Source
- Landscape
- calendar.h
- Generated on Thu Jan 10 2013 13:15:35 for ALMaSS Skylark ODdox by
   1.8.1.1
