# Supplementary material for: Modelling Skylarks (Alauda arvensis) to Predict Impacts of Changes in Land Management and Policy: Development and Testing of an Agent-Based Model
Source: PLoS One. 2013 Jun 6;8(6):e65803. doi: 10.1371/journal.pone.0065803 (PMC3675089; doi:10.1371/journal.pone.0065803)
Supplement: Supporting Information S4 — The skylark ODdox as a zipped archive. (ZIP) [file pone.0065803.s004.zip › Skylark_ODdox/class_adult_skylark__struct.html]

ALMaSS Skylark ODdox: AdultSkylark\_struct Class Reference


|  |
| --- |
| ALMaSS Skylark ODdox  2.0 |


- Main Page
- Related Pages
- Classes
- Files

- Class List
- Class Index
- Class Hierarchy
- Class Members

Public Attributes

AdultSkylark\_struct Class Reference

`#include <skylarks_all.h>`

List of all members.

|  |  |
| --- | --- |
| Public Attributes | |
| int | age |
| bool | sex |
| double | size |
| Public Attributes inherited from Skylark\_struct | |
| int | bx |
| int | by |
| Landscape \* | L |
| int | mh |
| SkTerritories \* | Terrs |
| int | x |
| int | y |

|  |  |
| --- | --- |
| Additional Inherited Members | |
| Public Member Functions inherited from Skylark\_struct | |
| virtual | ~Skylark\_struct () |

---

## Member Data Documentation

|  |
| --- |
| int AdultSkylark\_struct::age |

Referenced by Skylark\_Population\_Manager::CreateObjects(), Skylark\_Population\_Manager::Init(), and Skylark\_PreFledgeling::st\_Maturing().

|  |
| --- |
| bool AdultSkylark\_struct::sex |

Referenced by Skylark\_Population\_Manager::Init(), and Skylark\_PreFledgeling::st\_Maturing().

|  |
| --- |
| double AdultSkylark\_struct::size |

Referenced by Skylark\_Population\_Manager::CreateObjects(), Skylark\_Population\_Manager::Init(), and Skylark\_PreFledgeling::st\_Maturing().

---

The documentation for this class was generated from the following file:

- skylarks\_all.h


- AdultSkylark\_struct
- Generated on Thu Jan 10 2013 13:15:35 for ALMaSS Skylark ODdox by
   1.8.1.1
