# Supplementary material for: Modelling Skylarks (Alauda arvensis) to Predict Impacts of Changes in Land Management and Policy: Development and Testing of an Agent-Based Model
Source: PLoS One. 2013 Jun 6;8(6):e65803. doi: 10.1371/journal.pone.0065803 (PMC3675089; doi:10.1371/journal.pone.0065803)
Supplement: Supporting Information S4 — The skylark ODdox as a zipped archive. (ZIP) [file pone.0065803.s004.zip › Skylark_ODdox/class_adult_skylark__struct-members.html]

ALMaSS Skylark ODdox: Member List


|  |
| --- |
| ALMaSS Skylark ODdox  2.0 |


- Main Page
- Related Pages
- Classes
- Files

- Class List
- Class Index
- Class Hierarchy
- Class Members

AdultSkylark\_struct Member List

This is the complete list of members for AdultSkylark\_struct, including all inherited members.

|  |  |  |
| --- | --- | --- |
| age | AdultSkylark\_struct |  |
| bx | Skylark\_struct |  |
| by | Skylark\_struct |  |
| L | Skylark\_struct |  |
| mh | Skylark\_struct |  |
| sex | AdultSkylark\_struct |  |
| size | AdultSkylark\_struct |  |
| Terrs | Skylark\_struct |  |
| x | Skylark\_struct |  |
| y | Skylark\_struct |  |
| ~Skylark\_struct() | Skylark\_struct | inlinevirtual |


- Generated on Thu Jan 10 2013 13:15:35 for ALMaSS Skylark ODdox by
   1.8.1.1
