# Supplementary material for: Modelling Skylarks (Alauda arvensis) to Predict Impacts of Changes in Land Management and Policy: Development and Testing of an Agent-Based Model
Source: PLoS One. 2013 Jun 6;8(6):e65803. doi: 10.1371/journal.pone.0065803 (PMC3675089; doi:10.1371/journal.pone.0065803)
Supplement: Supporting Information S4 — The skylark ODdox as a zipped archive. (ZIP) [file pone.0065803.s004.zip › Skylark_ODdox/class_animal_position.html]

ALMaSS Skylark ODdox: AnimalPosition Class Reference


|  |
| --- |
| ALMaSS Skylark ODdox  2.0 |


- Main Page
- Related Pages
- Classes
- Files

- Class List
- Class Index
- Class Hierarchy
- Class Members

Public Attributes

AnimalPosition Class Reference

A class defining an animals position.
More...

`#include <populationmanager.h>`

List of all members.

|  |  |
| --- | --- |
| Public Attributes | |
| TTypesOfLandscapeElement | m\_EleType |
| TTypesOfVegetation | m\_VegType |
| unsigned | m\_x |
| unsigned | m\_y |

---

## Detailed Description

A class defining an animals position.

---

## Member Data Documentation

|  |
| --- |
| TTypesOfLandscapeElement AnimalPosition::m\_EleType |

Referenced by TSkylarkList< T >::Probe(), Population\_Manager::Probe(), and TAnimal::SupplyPosition().

|  |
| --- |
| TTypesOfVegetation AnimalPosition::m\_VegType |

Referenced by TSkylarkList< T >::Probe(), Population\_Manager::Probe(), and TAnimal::SupplyPosition().

|  |
| --- |
| unsigned AnimalPosition::m\_x |

Referenced by Population\_Manager::LOG(), TSkylarkList< T >::Probe(), Population\_Manager::Probe(), and TAnimal::SupplyPosition().

|  |
| --- |
| unsigned AnimalPosition::m\_y |

Referenced by Population\_Manager::LOG(), TSkylarkList< T >::Probe(), Population\_Manager::Probe(), and TAnimal::SupplyPosition().

---

The documentation for this class was generated from the following file:

- populationmanager.h


- AnimalPosition
- Generated on Thu Jan 10 2013 13:15:35 for ALMaSS Skylark ODdox by
   1.8.1.1
