# Supplementary material for: Modelling Skylarks (Alauda arvensis) to Predict Impacts of Changes in Land Management and Policy: Development and Testing of an Agent-Based Model
Source: PLoS One. 2013 Jun 6;8(6):e65803. doi: 10.1371/journal.pone.0065803 (PMC3675089; doi:10.1371/journal.pone.0065803)
Supplement: Supporting Information S4 — The skylark ODdox as a zipped archive. (ZIP) [file pone.0065803.s004.zip › Skylark_ODdox/class_bait_location.html]

ALMaSS Skylark ODdox: BaitLocation Class Reference


|  |
| --- |
| ALMaSS Skylark ODdox  2.0 |


- Main Page
- Related Pages
- Classes
- Files

- Class List
- Class Index
- Class Hierarchy
- Class Members

Public Member Functions |
Protected Attributes

BaitLocation Class Reference

Class used for describing the rodenticide bait location.
More...

`#include <Rodenticide.h>`

List of all members.

|  |  |
| --- | --- |
| Public Member Functions | |
|  | BaitLocation (int a\_x, int a\_y, TTypesBaitLocation a\_type) |
|  | BaitLocation constructor. |
| TTypesBaitLocation | GetBLtype (void) |
|  | Get bait location type. |
| int | GetMass (void) |
|  | Get bait mass. |
| int | GetstartDay (void) |
|  | Get start day. |
| bool | GetUseFlag () |
|  | Get use flag. |
| int | GetX (void) |
|  | Get x-coordinate. |
| int | GetY (void) |
|  | Get y-coordinate. |
| void | ReduceMass (int a\_mass) |
|  | Set bait mass. |
| void | Reset () |
|  | Reset all flags and mass. |
| void | SetBLtype (TTypesBaitLocation a\_BLt) |
|  | Set bait location type. |
| void | SetMass (int a\_mass) |
|  | Set bait mass. |
| void | SetstartDay (int a\_startDay) |
|  | Set start day. |
| void | SetUseFlag (bool a\_flag) |
|  | Set use flag. |
| void | SetX (int a\_x) |
|  | Set x-coordinate. |
| void | SetY (int a\_y) |
|  | Set y-coordinate. |
| virtual | ~BaitLocation (void) |
|  | BaitLocation destructor. |

|  |  |
| --- | --- |
| Protected Attributes | |
| TTypesBaitLocation | m\_BLtype |
|  | bait replenishment frequency |
| int | m\_mass |
|  | bait mass |
| int | m\_startDay |
|  | date for placement of first bait |
| bool | m\_useThisYear |
|  | flag for bait location use this year |
| int | m\_x |
|  | x-coordinate |
| int | m\_y |
|  | y-coordinate |

---

## Detailed Description

Class used for describing the rodenticide bait location.

---

## Constructor & Destructor Documentation

|  |  |  |  |
| --- | --- | --- | --- |
| BaitLocation::BaitLocation | ( | int | *a\_x*, |
|  |  | int | *a\_y*, |
|  |  | TTypesBaitLocation | *a\_type* |
|  | ) |  |  |

BaitLocation constructor.

{

SetX(a\_x);

SetY(a\_y);

SetBLtype(a\_type);

Reset();

}

|  |  |  |  |  |  |  |  |
| --- | --- | --- | --- | --- | --- | --- | --- |
| |  |  |  |  |  |  | | --- | --- | --- | --- | --- | --- | | BaitLocation::~BaitLocation | ( | void |  | ) |  | | virtual |

BaitLocation destructor.

{

;

}

---

## Member Function Documentation

|  |  |  |  |  |  |  |  |
| --- | --- | --- | --- | --- | --- | --- | --- |
| |  |  |  |  |  |  | | --- | --- | --- | --- | --- | --- | | TTypesBaitLocation BaitLocation::GetBLtype | ( | void |  | ) |  | | inline |

Get bait location type.

{ return m\_BLtype; }

|  |  |  |  |  |  |  |  |
| --- | --- | --- | --- | --- | --- | --- | --- |
| |  |  |  |  |  |  | | --- | --- | --- | --- | --- | --- | | int BaitLocation::GetMass | ( | void |  | ) |  | | inline |

Get bait mass.

{ return m\_mass; }

|  |  |  |  |  |  |  |  |
| --- | --- | --- | --- | --- | --- | --- | --- |
| |  |  |  |  |  |  | | --- | --- | --- | --- | --- | --- | | int BaitLocation::GetstartDay | ( | void |  | ) |  | | inline |

Get start day.

{ return m\_startDay; }

|  |  |  |  |  |  |  |
| --- | --- | --- | --- | --- | --- | --- |
| |  |  |  |  |  | | --- | --- | --- | --- | --- | | bool BaitLocation::GetUseFlag | ( |  | ) |  | | inline |

Get use flag.

{ return m\_useThisYear; }

|  |  |  |  |  |  |  |  |
| --- | --- | --- | --- | --- | --- | --- | --- |
| |  |  |  |  |  |  | | --- | --- | --- | --- | --- | --- | | int BaitLocation::GetX | ( | void |  | ) |  | | inline |

Get x-coordinate.

{ return m\_x; }

|  |  |  |  |  |  |  |  |
| --- | --- | --- | --- | --- | --- | --- | --- |
| |  |  |  |  |  |  | | --- | --- | --- | --- | --- | --- | | int BaitLocation::GetY | ( | void |  | ) |  | | inline |

Get y-coordinate.

{ return m\_y; }

|  |  |  |  |  |  |  |  |
| --- | --- | --- | --- | --- | --- | --- | --- |
| |  |  |  |  |  |  | | --- | --- | --- | --- | --- | --- | | void BaitLocation::ReduceMass | ( | int | *a\_mass* | ) |  | | inline |

Set bait mass.

{ m\_mass -= a\_mass; }

|  |  |  |  |  |  |  |  |
| --- | --- | --- | --- | --- | --- | --- | --- |
| |  |  |  |  |  |  | | --- | --- | --- | --- | --- | --- | | void BaitLocation::Reset | ( | void |  | ) |  | | inline |

Reset all flags and mass.

{ m\_mass = 0; m\_useThisYear = false; m\_startDay=-1;}

|  |  |  |  |  |  |  |  |
| --- | --- | --- | --- | --- | --- | --- | --- |
| |  |  |  |  |  |  | | --- | --- | --- | --- | --- | --- | | void BaitLocation::SetBLtype | ( | TTypesBaitLocation | *a\_BLt* | ) |  | | inline |

Set bait location type.

Referenced by RodenticideManager::RodenticideManager().

{ m\_BLtype = a\_BLt; }

|  |  |  |  |  |  |  |  |
| --- | --- | --- | --- | --- | --- | --- | --- |
| |  |  |  |  |  |  | | --- | --- | --- | --- | --- | --- | | void BaitLocation::SetMass | ( | int | *a\_mass* | ) |  | | inline |

Set bait mass.

{ m\_mass = a\_mass; }

|  |  |  |  |  |  |  |  |
| --- | --- | --- | --- | --- | --- | --- | --- |
| |  |  |  |  |  |  | | --- | --- | --- | --- | --- | --- | | void BaitLocation::SetstartDay | ( | int | *a\_startDay* | ) |  | | inline |

Set start day.

{ m\_startDay = a\_startDay; }

|  |  |  |  |  |  |  |  |
| --- | --- | --- | --- | --- | --- | --- | --- |
| |  |  |  |  |  |  | | --- | --- | --- | --- | --- | --- | | void BaitLocation::SetUseFlag | ( | bool | *a\_flag* | ) |  | | inline |

Set use flag.

{ m\_useThisYear = a\_flag; }

|  |  |  |  |  |  |  |  |
| --- | --- | --- | --- | --- | --- | --- | --- |
| |  |  |  |  |  |  | | --- | --- | --- | --- | --- | --- | | void BaitLocation::SetX | ( | int | *a\_x* | ) |  | | inline |

Set x-coordinate.

Referenced by RodenticideManager::RodenticideManager().

{ m\_x = a\_x; }

|  |  |  |  |  |  |  |  |
| --- | --- | --- | --- | --- | --- | --- | --- |
| |  |  |  |  |  |  | | --- | --- | --- | --- | --- | --- | | void BaitLocation::SetY | ( | int | *a\_y* | ) |  | | inline |

Set y-coordinate.

Referenced by RodenticideManager::RodenticideManager().

{ m\_y = a\_y; }

---

## Member Data Documentation

|  |  |  |
| --- | --- | --- |
| |  | | --- | | TTypesBaitLocation BaitLocation::m\_BLtype | | protected |

bait replenishment frequency

|  |  |  |
| --- | --- | --- |
| |  | | --- | | int BaitLocation::m\_mass | | protected |

bait mass

|  |  |  |
| --- | --- | --- |
| |  | | --- | | int BaitLocation::m\_startDay | | protected |

date for placement of first bait

|  |  |  |
| --- | --- | --- |
| |  | | --- | | bool BaitLocation::m\_useThisYear | | protected |

flag for bait location use this year

|  |  |  |
| --- | --- | --- |
| |  | | --- | | int BaitLocation::m\_x | | protected |

x-coordinate

This class has no functionality apart from get/set and arithmetic manipulation of class members.

|  |  |  |
| --- | --- | --- |
| |  | | --- | | int BaitLocation::m\_y | | protected |

y-coordinate

---

The documentation for this class was generated from the following files:

- Rodenticide.h
- Rodenticide.cpp


- BaitLocation
- Generated on Thu Jan 10 2013 13:15:35 for ALMaSS Skylark ODdox by
   1.8.1.1
