# Supplementary material for: Modelling Skylarks (Alauda arvensis) to Predict Impacts of Changes in Land Management and Policy: Development and Testing of an Agent-Based Model
Source: PLoS One. 2013 Jun 6;8(6):e65803. doi: 10.1371/journal.pone.0065803 (PMC3675089; doi:10.1371/journal.pone.0065803)
Supplement: Supporting Information S4 — The skylark ODdox as a zipped archive. (ZIP) [file pone.0065803.s004.zip › Skylark_ODdox/class_bait_location-members.html]

ALMaSS Skylark ODdox: Member List


|  |
| --- |
| ALMaSS Skylark ODdox  2.0 |


- Main Page
- Related Pages
- Classes
- Files

- Class List
- Class Index
- Class Hierarchy
- Class Members

BaitLocation Member List

This is the complete list of members for BaitLocation, including all inherited members.

|  |  |  |
| --- | --- | --- |
| BaitLocation(int a\_x, int a\_y, TTypesBaitLocation a\_type) | BaitLocation |  |
| GetBLtype(void) | BaitLocation | inline |
| GetMass(void) | BaitLocation | inline |
| GetstartDay(void) | BaitLocation | inline |
| GetUseFlag() | BaitLocation | inline |
| GetX(void) | BaitLocation | inline |
| GetY(void) | BaitLocation | inline |
| m\_BLtype | BaitLocation | protected |
| m\_mass | BaitLocation | protected |
| m\_startDay | BaitLocation | protected |
| m\_useThisYear | BaitLocation | protected |
| m\_x | BaitLocation | protected |
| m\_y | BaitLocation | protected |
| ReduceMass(int a\_mass) | BaitLocation | inline |
| Reset() | BaitLocation | inline |
| SetBLtype(TTypesBaitLocation a\_BLt) | BaitLocation | inline |
| SetMass(int a\_mass) | BaitLocation | inline |
| SetstartDay(int a\_startDay) | BaitLocation | inline |
| SetUseFlag(bool a\_flag) | BaitLocation | inline |
| SetX(int a\_x) | BaitLocation | inline |
| SetY(int a\_y) | BaitLocation | inline |
| ~BaitLocation(void) | BaitLocation | virtual |


- Generated on Thu Jan 10 2013 13:15:35 for ALMaSS Skylark ODdox by
   1.8.1.1
