# Supplementary material for: Modelling Skylarks (Alauda arvensis) to Predict Impacts of Changes in Land Management and Policy: Development and Testing of an Agent-Based Model
Source: PLoS One. 2013 Jun 6;8(6):e65803. doi: 10.1371/journal.pone.0065803 (PMC3675089; doi:10.1371/journal.pone.0065803)
Supplement: Supporting Information S4 — The skylark ODdox as a zipped archive. (ZIP) [file pone.0065803.s004.zip › Skylark_ODdox/class_binary_map_base.html]

ALMaSS Skylark ODdox: BinaryMapBase Class Reference


|  |
| --- |
| ALMaSS Skylark ODdox  2.0 |


- Main Page
- Related Pages
- Classes
- Files

- Class List
- Class Index
- Class Hierarchy
- Class Members

Public Member Functions |
Protected Attributes

BinaryMapBase Class Reference

`#include <BinaryMapBase.h>`

List of all members.

|  |  |
| --- | --- |
| Public Member Functions | |
|  | BinaryMapBase (unsigned int a\_width, unsigned int a\_height, unsigned int a\_resolution, unsigned int a\_noValues) |
| void | ClearMap () |
| void | ClearValue (unsigned a\_x, unsigned a\_y) |
| uint64 | GetValue (unsigned a\_x, unsigned a\_y) |
| void | SetValue (unsigned a\_x, unsigned a\_y, unsigned a\_value) |
|  | ~BinaryMapBase () |

|  |  |
| --- | --- |
| Protected Attributes | |
| unsigned int | m\_colourRes |
| unsigned int | m\_colourScaler |
| unsigned int | m\_height |
| uint64 \* | m\_map |
| unsigned int | m\_maplength |
| uint64 | m\_mask |
| unsigned int | m\_maskbits |
| unsigned int | m\_resolution |
| unsigned int | m\_resolutionscaler |
| unsigned int | m\_width |

---

## Constructor & Destructor Documentation

|  |  |  |  |
| --- | --- | --- | --- |
| BinaryMapBase::BinaryMapBase | ( | unsigned int | *a\_width*, |
|  |  | unsigned int | *a\_height*, |
|  |  | unsigned int | *a\_resolution*, |
|  |  | unsigned int | *a\_noValues* |
|  | ) |  |  |

References ClearMap(), m\_colourRes, m\_colourScaler, m\_height, m\_map, m\_maplength, m\_mask, m\_maskbits, m\_resolution, m\_resolutionscaler, and m\_width.

{

// Assign member variables

m\_width = a\_width;

m\_height = a\_height;

m\_colourRes = a\_noValues;

m\_resolution = a\_resolution;

// 2 = 1, 4 = 2, 8 = 3 etc..

m\_resolutionscaler = 0;

unsigned int n=m\_resolution;

do {

n = n >> 1;

m\_resolutionscaler++;

} while (n>0);

m\_resolutionscaler--;

m\_colourScaler = 0;

n=m\_colourRes;

do {

n = n >> 1;

m\_colourScaler++;

} while (n>0);

m\_colourScaler--;

// The total number of 64bit integers needed is height x width / 64 (rounded up);

// But we need to take the resolution into account too - hence:

// ( (height x width)/(resolution\*resolution) ) / 64, then add one in case there is a fractional part

// Then we also need to have the size of the colour resolution calculated in here.

m\_maplength = ( ( ( m\_width \* m\_height \* m\_colourScaler ) / ( m\_resolution \* m\_resolution )) / 64 ) + 1;

// Need to adjust m\_width & m\_height to match resolution

m\_width = m\_width >> m\_resolutionscaler;

m\_height = m\_height >> m\_resolutionscaler;

m\_map = new uint64[m\_maplength];

// create the mask

m\_mask = m\_colourRes-1;

m\_maskbits = m\_colourScaler -1;

ClearMap();

}

|  |  |  |  |  |
| --- | --- | --- | --- | --- |
| BinaryMapBase::~BinaryMapBase | ( |  | ) |  |

{

;

}

---

## Member Function Documentation

|  |  |  |  |  |
| --- | --- | --- | --- | --- |
| void BinaryMapBase::ClearMap | ( |  | ) |  |

References m\_map, and m\_maplength.

Referenced by BinaryMapBase().

{

for (unsigned int i = 0; i < m\_maplength; i++) m\_map[i]=0;

}

|  |  |  |  |
| --- | --- | --- | --- |
| void BinaryMapBase::ClearValue | ( | unsigned | *a\_x*, |
|  |  | unsigned | *a\_y* |
|  | ) |  |  |

Avoids passing one parameter compared to SetValue(unsigned, unsigned, unsigned)

References m\_colourScaler, m\_map, m\_mask, m\_resolutionscaler, and m\_width.

{

// To find an x/y co-ordinate:

// We need to find the 64-bit int that we are in, then the bit or bytes we need

unsigned int ref = ( ( ( a\_y >> m\_resolutionscaler ) \* m\_width ) + ( a\_x >> m\_resolutionscaler ) ) \* m\_colourScaler;

// ref holds the first bit of information we need, i.e. the location within m\_map in terms of element number.

// Next we need to figure out which uint64 this is in.....

unsigned int index = ref >> 6; // ....so divide by 64

// get the value from this location to a temporary variable

uint64 newvalue = m\_map[index];

// identify the bits we are interested in

uint64 bits = ( ref & 63 );

// Our mask is sized based on the colours represented 1 - binary, 2 - 4, 4 - 16 , 8 - 256 (NB only powers of 2 are allowed).

// The mask is created in the constructor.

// So we need to shift our mask to cover the correct bits.

uint64 mask = ~( m\_mask << bits );

// Blank out any current values

newvalue = newvalue & mask;

m\_map[index] = newvalue;

;

}

|  |  |  |  |
| --- | --- | --- | --- |
| uint64 BinaryMapBase::GetValue | ( | unsigned | *a\_x*, |
|  |  | unsigned | *a\_y* |
|  | ) |  |  |

References m\_colourScaler, m\_map, m\_mask, m\_resolutionscaler, and m\_width.

{

// To find an x/y co-ordinate:

// We need to find the 64-bit int that we are in, then the bit or bytes we need

unsigned int ref = ( ( ( a\_y >> m\_resolutionscaler ) \* m\_width ) + ( a\_x >> m\_resolutionscaler ) ) \* m\_colourScaler;

// ref holds the first bit of information we need, i.e. the location within m\_map in terms of element number.

// Next we need to figure out which uint64 this is in.....

unsigned int index = ref >> 6; // ....so divide by 64

// get the value from this location to a temporary variable

uint64 returnvalue = m\_map[index];

// identify the bits we are interested in

uint64 bits = ( ref & 63 );

// Our mask is sized based on the colours represented 1 - binary, 2 - 4, 4 - 16 , 8 - 256 (NB only powers of 2 are allowed).

// The mask is created in the constructor.

// So we need to shift our return value and mask it.

returnvalue = ( returnvalue >> bits ) & m\_mask;

return returnvalue;

}

|  |  |  |  |
| --- | --- | --- | --- |
| void BinaryMapBase::SetValue | ( | unsigned | *a\_x*, |
|  |  | unsigned | *a\_y*, |
|  |  | unsigned | *a\_value* |
|  | ) |  |  |

References m\_colourScaler, m\_map, m\_mask, m\_resolutionscaler, and m\_width.

{

// To find an x/y co-ordinate:

// We need to find the 64-bit int that we are in, then the bit or bytes we need

unsigned int ref = ( ( ( a\_y >> m\_resolutionscaler ) \* m\_width ) + ( a\_x >> m\_resolutionscaler ) ) \* m\_colourScaler;

// ref holds the first bit of information we need, i.e. the location within m\_map in terms of element number.

// Next we need to figure out which uint64 this is in.....

unsigned int index = ref >> 6; // ....so divide by 64

// get the value from this location to a temporary variable

uint64 newvalue = m\_map[index];

// identify the bits we are interested in

uint64 bits = ( ref & 63 );

// Our mask is sized based on the colours represented 1 - binary, 2 - 4, 4 - 16 , 8 - 256 (NB only powers of 2 are allowed).

// The mask is created in the constructor.

// So we need to shift our mask to cover the correct bits.

uint64 mask = ~( m\_mask << bits );

// Blank out any current values

newvalue = newvalue & mask;

// Pop our new value in

uint64 value = a\_value;

newvalue = newvalue | ( value << bits);

m\_map[index] = newvalue;

}

---

## Member Data Documentation

|  |  |  |
| --- | --- | --- |
| |  | | --- | | unsigned int BinaryMapBase::m\_colourRes | | protected |

Referenced by BinaryMapBase().

|  |  |  |
| --- | --- | --- |
| |  | | --- | | unsigned int BinaryMapBase::m\_colourScaler | | protected |

Referenced by BinaryMapBase(), ClearValue(), GetValue(), and SetValue().

|  |  |  |
| --- | --- | --- |
| |  | | --- | | unsigned int BinaryMapBase::m\_height | | protected |

Referenced by BinaryMapBase().

|  |  |  |
| --- | --- | --- |
| |  | | --- | | uint64\* BinaryMapBase::m\_map | | protected |

Referenced by BinaryMapBase(), ClearMap(), ClearValue(), GetValue(), and SetValue().

|  |  |  |
| --- | --- | --- |
| |  | | --- | | unsigned int BinaryMapBase::m\_maplength | | protected |

Referenced by BinaryMapBase(), and ClearMap().

|  |  |  |
| --- | --- | --- |
| |  | | --- | | uint64 BinaryMapBase::m\_mask | | protected |

Referenced by BinaryMapBase(), ClearValue(), GetValue(), and SetValue().

|  |  |  |
| --- | --- | --- |
| |  | | --- | | unsigned int BinaryMapBase::m\_maskbits | | protected |

Referenced by BinaryMapBase().

|  |  |  |
| --- | --- | --- |
| |  | | --- | | unsigned int BinaryMapBase::m\_resolution | | protected |

Referenced by BinaryMapBase().

|  |  |  |
| --- | --- | --- |
| |  | | --- | | unsigned int BinaryMapBase::m\_resolutionscaler | | protected |

Referenced by BinaryMapBase(), ClearValue(), GetValue(), and SetValue().

|  |  |  |
| --- | --- | --- |
| |  | | --- | | unsigned int BinaryMapBase::m\_width | | protected |

Referenced by BinaryMapBase(), ClearValue(), GetValue(), and SetValue().

---

The documentation for this class was generated from the following files:

- BinaryMapBase.h
- BinaryMapBase.cpp


- BinaryMapBase
- Generated on Thu Jan 10 2013 13:15:35 for ALMaSS Skylark ODdox by
   1.8.1.1
