# Supplementary material for: Modelling Skylarks (Alauda arvensis) to Predict Impacts of Changes in Land Management and Policy: Development and Testing of an Agent-Based Model
Source: PLoS One. 2013 Jun 6;8(6):e65803. doi: 10.1371/journal.pone.0065803 (PMC3675089; doi:10.1371/journal.pone.0065803)
Supplement: Supporting Information S4 — The skylark ODdox as a zipped archive. (ZIP) [file pone.0065803.s004.zip › Skylark_ODdox/class_binary_map_base-members.html]

ALMaSS Skylark ODdox: Member List


|  |
| --- |
| ALMaSS Skylark ODdox  2.0 |


- Main Page
- Related Pages
- Classes
- Files

- Class List
- Class Index
- Class Hierarchy
- Class Members

BinaryMapBase Member List

This is the complete list of members for BinaryMapBase, including all inherited members.

|  |  |  |
| --- | --- | --- |
| BinaryMapBase(unsigned int a\_width, unsigned int a\_height, unsigned int a\_resolution, unsigned int a\_noValues) | BinaryMapBase |  |
| ClearMap() | BinaryMapBase |  |
| ClearValue(unsigned a\_x, unsigned a\_y) | BinaryMapBase |  |
| GetValue(unsigned a\_x, unsigned a\_y) | BinaryMapBase |  |
| m\_colourRes | BinaryMapBase | protected |
| m\_colourScaler | BinaryMapBase | protected |
| m\_height | BinaryMapBase | protected |
| m\_map | BinaryMapBase | protected |
| m\_maplength | BinaryMapBase | protected |
| m\_mask | BinaryMapBase | protected |
| m\_maskbits | BinaryMapBase | protected |
| m\_resolution | BinaryMapBase | protected |
| m\_resolutionscaler | BinaryMapBase | protected |
| m\_width | BinaryMapBase | protected |
| SetValue(unsigned a\_x, unsigned a\_y, unsigned a\_value) | BinaryMapBase |  |
| ~BinaryMapBase() | BinaryMapBase |  |


- Generated on Thu Jan 10 2013 13:15:35 for ALMaSS Skylark ODdox by
   1.8.1.1
