# Supplementary material for: Modelling Skylarks (Alauda arvensis) to Predict Impacts of Changes in Land Management and Policy: Development and Testing of an Agent-Based Model
Source: PLoS One. 2013 Jun 6;8(6):e65803. doi: 10.1371/journal.pone.0065803 (PMC3675089; doi:10.1371/journal.pone.0065803)
Supplement: Supporting Information S4 — The skylark ODdox as a zipped archive. (ZIP) [file pone.0065803.s004.zip › Skylark_ODdox/class_calendar.html]

ALMaSS Skylark ODdox: Calendar Class Reference


|  |
| --- |
| ALMaSS Skylark ODdox  2.0 |


- Main Page
- Related Pages
- Classes
- Files

- Class List
- Class Index
- Class Hierarchy
- Class Members

Public Member Functions |
Private Attributes |
Static Private Attributes

Calendar Class Reference

`#include <calendar.h>`

List of all members.

|  |  |
| --- | --- |
| Public Member Functions | |
|  | Calendar (void) |
| long | Date (void) |
| long | DayInYear (void) |
| int | DayInYear (int a\_day, int a\_month) |
| int | DayLength (void) |
| int | DayLength (int a\_day\_in\_year) |
| int | GetDayInMonth (void) |
| int | GetFirstYear (void) |
| int | GetHour (void) |
| int | GetLastYear (void) |
| int | GetMinute (void) |
| int | GetMonth (void) |
| int | GetYear (void) |
| int | GetYearNumber (void) |
| long | GlobalDate (int a\_day, int a\_month, int a\_year) |
| bool | JanFirst (void) |
| bool | MarchFirst (void) |
| long | OldDays (void) |
| void | Reset (void) |
| void | SetFirstYear (int a\_year) |
| void | SetLastYear (int a\_year) |
| void | Tick (void) |
| bool | TickHour (void) |
| bool | TickMinute (void) |
| bool | ValidDate (int a\_day, int a\_month) |

|  |  |
| --- | --- |
| Private Attributes | |
| long | m\_date |
| int | m\_day\_in\_month |
| int | m\_day\_in\_year |
| int | m\_firstyear |
| int | m\_hours |
| bool | m\_janfirst |
| int | m\_lastyear |
| bool | m\_marchfirst |
| int | m\_minutes |
| int | m\_month |
| long | m\_olddays |
| int | m\_simulationyear |
| int | m\_todayslength |
| int | m\_year |

|  |  |
| --- | --- |
| Static Private Attributes | |
| static int | m\_daylength [] |

---

## Constructor & Destructor Documentation

|  |  |  |  |  |  |
| --- | --- | --- | --- | --- | --- |
| Calendar::Calendar | ( | void |  | ) |  |

References Reset().

{

Reset();

}

---

## Member Function Documentation

|  |  |  |  |  |  |  |  |
| --- | --- | --- | --- | --- | --- | --- | --- |
| |  |  |  |  |  |  | | --- | --- | --- | --- | --- | --- | | long Calendar::Date | ( | void |  | ) |  | | inline |

References m\_date.

Referenced by Farm::BurnStrawStubble(), Farm::CheckRotationManagementLoop(), Farm::CutToHay(), VegElement::DoDevelopment(), UnsprayedFieldMargin::DoDevelopment(), Skylark\_Population\_Manager::DoFirst(), Skylark\_Clutch::EndStep(), Skylark\_Nestling::EndStep(), Skylark\_PreFledgeling::EndStep(), Weather::GetSnow(), Farm::HandleEvents(), Farm::Harvest(), Farm::HayBailing(), Farm::HayTurning(), Farm::InitiateManagement(), Skylark\_Clutch::OnFarmEvent(), Skylark\_Nestling::OnFarmEvent(), Skylark\_PreFledgeling::OnFarmEvent(), Skylark\_Female::OnFarmEvent(), Skylark\_Male::OnFarmEvent(), Skylark\_Clutch::OnMumGone(), Farm::RowCultivation(), VegElement::SetGrowthPhase(), Skylark\_Female::Step(), Skylark\_Male::Step(), Farm::StrawChopping(), Farm::Strigling(), Farm::StriglingSow(), Farm::StubbleHarrowing(), Landscape::SupplyGlobalDate(), and Weather::Tick().

{ return m\_date; }

|  |  |  |  |  |  |  |  |
| --- | --- | --- | --- | --- | --- | --- | --- |
| |  |  |  |  |  |  | | --- | --- | --- | --- | --- | --- | | long Calendar::DayInYear | ( | void |  | ) |  | | inline |

References m\_day\_in\_year.

Referenced by Farm::CattleIsOut(), Farm::CattleIsOutLow(), RoadsideVerge::DoDevelopment(), Orchard::DoDevelopment(), OrchardBand::DoDevelopment(), OrchardGrass::DoDevelopment(), Landscape::DumpMapInfoByArea(), VegElement::ForceGrowthInitialize(), VegElement::ForceGrowthTest(), GlobalDate(), VegElement::ReduceVeg\_Extended(), Landscape::SupplyDayInYear(), Landscape::Tick(), and Weather::Tick().

{ return m\_day\_in\_year; }

|  |  |  |  |
| --- | --- | --- | --- |
| int Calendar::DayInYear | ( | int | *a\_day*, |
|  |  | int | *a\_month* |
|  | ) |  |  |

{

const int m\_monthsum[12] = {

0, 31, 59, 90, 120, 151, 181, 212, 243, 273, 304, 334 };

return m\_monthsum[ a\_month - 1 ] + a\_day - 1;

}

|  |  |  |  |  |  |  |  |
| --- | --- | --- | --- | --- | --- | --- | --- |
| |  |  |  |  |  |  | | --- | --- | --- | --- | --- | --- | | int Calendar::DayLength | ( | void |  | ) |  | | inline |

References m\_todayslength.

Referenced by Landscape::SupplyDaylength().

{ return m\_todayslength; }

|  |  |  |  |  |  |
| --- | --- | --- | --- | --- | --- |
| int Calendar::DayLength | ( | int | *a\_day\_in\_year* | ) |  |

References g\_msg, m\_daylength, MapErrorMsg::Warn(), and WARN\_BUG.

{

if ( a\_day\_in\_year<0 || a\_day\_in\_year>364 ) {

g\_msg->Warn( WARN\_BUG, "Calendar::DayLength(): Day outside a year!",

"" );

exit(1);

}

return m\_daylength[ a\_day\_in\_year ];

}

|  |  |  |  |  |  |  |  |
| --- | --- | --- | --- | --- | --- | --- | --- |
| |  |  |  |  |  |  | | --- | --- | --- | --- | --- | --- | | int Calendar::GetDayInMonth | ( | void |  | ) |  | | inline |

References m\_day\_in\_month.

Referenced by Landscape::SupplyDayInMonth().

{ return m\_day\_in\_month; }

|  |  |  |  |  |  |  |  |
| --- | --- | --- | --- | --- | --- | --- | --- |
| |  |  |  |  |  |  | | --- | --- | --- | --- | --- | --- | | int Calendar::GetFirstYear | ( | void |  | ) |  | | inline |

References m\_firstyear.

{ return m\_firstyear; }

|  |  |  |  |  |  |  |  |
| --- | --- | --- | --- | --- | --- | --- | --- |
| |  |  |  |  |  |  | | --- | --- | --- | --- | --- | --- | | int Calendar::GetHour | ( | void |  | ) |  | | inline |

References m\_hours.

Referenced by LargeRoad::GetTrafficLoad(), and SmallRoad::GetTrafficLoad().

{ return m\_hours; }

|  |  |  |  |  |  |  |  |
| --- | --- | --- | --- | --- | --- | --- | --- |
| |  |  |  |  |  |  | | --- | --- | --- | --- | --- | --- | | int Calendar::GetLastYear | ( | void |  | ) |  | | inline |

References m\_lastyear.

Referenced by Weather::Weather().

{ return m\_lastyear; }

|  |  |  |  |  |  |  |  |
| --- | --- | --- | --- | --- | --- | --- | --- |
| |  |  |  |  |  |  | | --- | --- | --- | --- | --- | --- | | int Calendar::GetMinute | ( | void |  | ) |  | | inline |

References m\_minutes.

{ return m\_minutes; }

|  |  |  |  |  |  |  |  |
| --- | --- | --- | --- | --- | --- | --- | --- |
| |  |  |  |  |  |  | | --- | --- | --- | --- | --- | --- | | int Calendar::GetMonth | ( | void |  | ) |  | | inline |

References m\_month.

Referenced by RoadsideVerge::DoDevelopment(), LargeRoad::GetTrafficLoad(), SmallRoad::GetTrafficLoad(), Landscape::SupplyMonth(), and Landscape::SupplyWindDirection().

{ return m\_month + 1; }

|  |  |  |  |  |  |  |  |
| --- | --- | --- | --- | --- | --- | --- | --- |
| |  |  |  |  |  |  | | --- | --- | --- | --- | --- | --- | | int Calendar::GetYear | ( | void |  | ) |  | | inline |

References m\_year.

Referenced by Landscape::SupplyYear().

{ return m\_year; }

|  |  |  |  |  |  |  |  |
| --- | --- | --- | --- | --- | --- | --- | --- |
| |  |  |  |  |  |  | | --- | --- | --- | --- | --- | --- | | int Calendar::GetYearNumber | ( | void |  | ) |  | | inline |

References m\_simulationyear.

Referenced by Landscape::SupplyYearNumber().

{ return m\_simulationyear; }

|  |  |  |  |
| --- | --- | --- | --- |
| long Calendar::GlobalDate | ( | int | *a\_day*, |
|  |  | int | *a\_month*, |
|  |  | int | *a\_year* |
|  | ) |  |  |

References DayInYear(), and m\_olddays.

{

return m\_olddays + DayInYear( a\_day, a\_month) + a\_year\*365;

}

|  |  |  |  |  |  |  |  |
| --- | --- | --- | --- | --- | --- | --- | --- |
| |  |  |  |  |  |  | | --- | --- | --- | --- | --- | --- | | bool Calendar::JanFirst | ( | void |  | ) |  | | inline |

References m\_janfirst.

Referenced by RoadsideVerge::DoDevelopment(), Orchard::DoDevelopment(), OrchardGrass::DoDevelopment(), and Landscape::Tick().

{ return m\_janfirst; }

|  |  |  |  |  |  |  |  |
| --- | --- | --- | --- | --- | --- | --- | --- |
| |  |  |  |  |  |  | | --- | --- | --- | --- | --- | --- | | bool Calendar::MarchFirst | ( | void |  | ) |  | | inline |

References m\_marchfirst.

Referenced by Landscape::Tick().

{ return m\_marchfirst; }

|  |  |  |  |  |  |  |  |
| --- | --- | --- | --- | --- | --- | --- | --- |
| |  |  |  |  |  |  | | --- | --- | --- | --- | --- | --- | | long Calendar::OldDays | ( | void |  | ) |  | | inline |

References m\_olddays.

Referenced by Landscape::DumpMapInfoByArea().

{ return m\_olddays; }

|  |  |  |  |  |  |
| --- | --- | --- | --- | --- | --- |
| void Calendar::Reset | ( | void |  | ) |  |

References m\_date, m\_day\_in\_month, m\_day\_in\_year, m\_firstyear, m\_hours, m\_janfirst, m\_marchfirst, m\_minutes, m\_month, m\_olddays, m\_simulationyear, and m\_year.

Referenced by Calendar(), Landscape::Landscape(), and Weather::Weather().

{

m\_date = 0;

m\_olddays = 0;

m\_day\_in\_month = 1;

m\_minutes = 0;

m\_hours = 0;

m\_day\_in\_year = 0; // [0..364]

m\_month = 0; // [0..11]

m\_year = m\_firstyear;

m\_simulationyear = 0;

m\_janfirst = true;

m\_marchfirst = false;

}

|  |  |  |  |  |  |  |  |
| --- | --- | --- | --- | --- | --- | --- | --- |
| |  |  |  |  |  |  | | --- | --- | --- | --- | --- | --- | | void Calendar::SetFirstYear | ( | int | *a\_year* | ) |  | | inline |

References m\_firstyear.

Referenced by Weather::Weather().

{ m\_firstyear = a\_year; }

|  |  |  |  |  |  |  |  |
| --- | --- | --- | --- | --- | --- | --- | --- |
| |  |  |  |  |  |  | | --- | --- | --- | --- | --- | --- | | void Calendar::SetLastYear | ( | int | *a\_year* | ) |  | | inline |

References m\_lastyear.

Referenced by Weather::Weather().

{ m\_lastyear = a\_year; }

|  |  |  |  |  |  |
| --- | --- | --- | --- | --- | --- |
| void Calendar::Tick | ( | void |  | ) |  |

References m\_date, m\_day\_in\_month, m\_day\_in\_year, m\_daylength, m\_hours, m\_janfirst, m\_marchfirst, m\_minutes, m\_month, m\_olddays, m\_simulationyear, m\_todayslength, and m\_year.

Referenced by Landscape::Tick().

{

const int m\_monthlength[12] = {

31, 28, 31, 30, 31, 30, 31, 31, 30, 31, 30, 31 };

const int m\_maxmonth = 11;

m\_minutes = 0;

m\_hours = 0;

m\_date++;

m\_day\_in\_year++;

m\_day\_in\_month++;

m\_janfirst = false;

m\_marchfirst = false;

if ( m\_day\_in\_month > m\_monthlength[ m\_month ])

{

m\_day\_in\_month = 1;

m\_month++;

if ( m\_month == 2 )

// March 1st.

m\_marchfirst = true;

}

if ( m\_month > m\_maxmonth )

{

m\_month = 0;

m\_year++;

m\_simulationyear++;

m\_olddays += 365;

m\_janfirst = true;

m\_day\_in\_year = 0;

}

m\_todayslength = m\_daylength[ m\_day\_in\_year ];

}

|  |  |  |  |  |  |
| --- | --- | --- | --- | --- | --- |
| bool Calendar::TickHour | ( | void |  | ) |  |

References m\_hours, and m\_minutes.

Referenced by Landscape::TickHour(), and TickMinute().

{

bool dotick = false;

if ( m\_hours++ > 23 ) {

m\_minutes = 0;

m\_hours = 0;

dotick = true;

}

return dotick;

}

|  |  |  |  |  |  |
| --- | --- | --- | --- | --- | --- |
| bool Calendar::TickMinute | ( | void |  | ) |  |

References m\_minutes, and TickHour().

Referenced by Landscape::TickMinute().

{

bool dotick = false;

if ( m\_minutes++ > 59 ) {

m\_minutes = 0;

dotick = TickHour();

}

return dotick;

}

|  |  |  |  |
| --- | --- | --- | --- |
| bool Calendar::ValidDate | ( | int | *a\_day*, |
|  |  | int | *a\_month* |
|  | ) |  |  |

{

const int m\_monthlength[12] = {

31, 28, 31, 30, 31, 30, 31, 31, 30, 31, 30, 31 };

if ( a\_month < 1 ||

a\_month > 12 ||

a\_day < 1 ||

a\_day > m\_monthlength[ a\_month - 1 ] ) {

return false;

} else {

return true;

}

}

---

## Member Data Documentation

|  |  |  |
| --- | --- | --- |
| |  | | --- | | long Calendar::m\_date | | private |

Referenced by Date(), Reset(), and Tick().

|  |  |  |
| --- | --- | --- |
| |  | | --- | | int Calendar::m\_day\_in\_month | | private |

Referenced by GetDayInMonth(), Reset(), and Tick().

|  |  |  |
| --- | --- | --- |
| |  | | --- | | int Calendar::m\_day\_in\_year | | private |

Referenced by DayInYear(), Reset(), and Tick().

|  |  |  |
| --- | --- | --- |
| |  | | --- | | int Calendar::m\_daylength | | staticprivate |

Referenced by DayLength(), and Tick().

|  |  |  |
| --- | --- | --- |
| |  | | --- | | int Calendar::m\_firstyear | | private |

Referenced by GetFirstYear(), Reset(), and SetFirstYear().

|  |  |  |
| --- | --- | --- |
| |  | | --- | | int Calendar::m\_hours | | private |

Referenced by GetHour(), Reset(), Tick(), and TickHour().

|  |  |  |
| --- | --- | --- |
| |  | | --- | | bool Calendar::m\_janfirst | | private |

Referenced by JanFirst(), Reset(), and Tick().

|  |  |  |
| --- | --- | --- |
| |  | | --- | | int Calendar::m\_lastyear | | private |

Referenced by GetLastYear(), and SetLastYear().

|  |  |  |
| --- | --- | --- |
| |  | | --- | | bool Calendar::m\_marchfirst | | private |

Referenced by MarchFirst(), Reset(), and Tick().

|  |  |  |
| --- | --- | --- |
| |  | | --- | | int Calendar::m\_minutes | | private |

Referenced by GetMinute(), Reset(), Tick(), TickHour(), and TickMinute().

|  |  |  |
| --- | --- | --- |
| |  | | --- | | int Calendar::m\_month | | private |

Referenced by GetMonth(), Reset(), and Tick().

|  |  |  |
| --- | --- | --- |
| |  | | --- | | long Calendar::m\_olddays | | private |

Referenced by GlobalDate(), OldDays(), Reset(), and Tick().

|  |  |  |
| --- | --- | --- |
| |  | | --- | | int Calendar::m\_simulationyear | | private |

Referenced by GetYearNumber(), Reset(), and Tick().

|  |  |  |
| --- | --- | --- |
| |  | | --- | | int Calendar::m\_todayslength | | private |

Referenced by DayLength(), and Tick().

|  |  |  |
| --- | --- | --- |
| |  | | --- | | int Calendar::m\_year | | private |

Referenced by GetYear(), Reset(), and Tick().

---

The documentation for this class was generated from the following files:

- calendar.h
- calendar.cpp
- daylength.h


- Calendar
- Generated on Thu Jan 10 2013 13:15:35 for ALMaSS Skylark ODdox by
   1.8.1.1
