# Supplementary material for: Modelling Skylarks (Alauda arvensis) to Predict Impacts of Changes in Land Management and Policy: Development and Testing of an Agent-Based Model
Source: PLoS One. 2013 Jun 6;8(6):e65803. doi: 10.1371/journal.pone.0065803 (PMC3675089; doi:10.1371/journal.pone.0065803)
Supplement: Supporting Information S4 — The skylark ODdox as a zipped archive. (ZIP) [file pone.0065803.s004.zip › Skylark_ODdox/class_calendar-members.html]

ALMaSS Skylark ODdox: Member List


|  |
| --- |
| ALMaSS Skylark ODdox  2.0 |


- Main Page
- Related Pages
- Classes
- Files

- Class List
- Class Index
- Class Hierarchy
- Class Members

Calendar Member List

This is the complete list of members for Calendar, including all inherited members.

|  |  |  |
| --- | --- | --- |
| Calendar(void) | Calendar |  |
| Date(void) | Calendar | inline |
| DayInYear(void) | Calendar | inline |
| DayInYear(int a\_day, int a\_month) | Calendar |  |
| DayLength(void) | Calendar | inline |
| DayLength(int a\_day\_in\_year) | Calendar |  |
| GetDayInMonth(void) | Calendar | inline |
| GetFirstYear(void) | Calendar | inline |
| GetHour(void) | Calendar | inline |
| GetLastYear(void) | Calendar | inline |
| GetMinute(void) | Calendar | inline |
| GetMonth(void) | Calendar | inline |
| GetYear(void) | Calendar | inline |
| GetYearNumber(void) | Calendar | inline |
| GlobalDate(int a\_day, int a\_month, int a\_year) | Calendar |  |
| JanFirst(void) | Calendar | inline |
| m\_date | Calendar | private |
| m\_day\_in\_month | Calendar | private |
| m\_day\_in\_year | Calendar | private |
| m\_daylength | Calendar | privatestatic |
| m\_firstyear | Calendar | private |
| m\_hours | Calendar | private |
| m\_janfirst | Calendar | private |
| m\_lastyear | Calendar | private |
| m\_marchfirst | Calendar | private |
| m\_minutes | Calendar | private |
| m\_month | Calendar | private |
| m\_olddays | Calendar | private |
| m\_simulationyear | Calendar | private |
| m\_todayslength | Calendar | private |
| m\_year | Calendar | private |
| MarchFirst(void) | Calendar | inline |
| OldDays(void) | Calendar | inline |
| Reset(void) | Calendar |  |
| SetFirstYear(int a\_year) | Calendar | inline |
| SetLastYear(int a\_year) | Calendar | inline |
| Tick(void) | Calendar |  |
| TickHour(void) | Calendar |  |
| TickMinute(void) | Calendar |  |
| ValidDate(int a\_day, int a\_month) | Calendar |  |


- Generated on Thu Jan 10 2013 13:15:35 for ALMaSS Skylark ODdox by
   1.8.1.1
