# Supplementary material for: Modelling Skylarks (Alauda arvensis) to Predict Impacts of Changes in Land Management and Policy: Development and Testing of an Agent-Based Model
Source: PLoS One. 2013 Jun 6;8(6):e65803. doi: 10.1371/journal.pone.0065803 (PMC3675089; doi:10.1371/journal.pone.0065803)
Supplement: Supporting Information S4 — The skylark ODdox as a zipped archive. (ZIP) [file pone.0065803.s004.zip › Skylark_ODdox/class_cfg_base.html]

ALMaSS Skylark ODdox: CfgBase Class Reference


|  |
| --- |
| ALMaSS Skylark ODdox  2.0 |


- Main Page
- Related Pages
- Classes
- Files

- Class List
- Class Index
- Class Hierarchy
- Class Members

Public Member Functions |
Private Attributes

CfgBase Class Reference

Base class for a configurator entry.
More...

`#include <configurator.h>`

List of all members.

|  |  |
| --- | --- |
| Public Member Functions | |
|  | CfgBase (const char \*a\_key, CfgSecureLevel a\_level) |
| const string | getkey (void) |
| CfgSecureLevel | getlevel (void) |
| virtual CfgType | gettype (void) |
| virtual | ~CfgBase (void) |

|  |  |
| --- | --- |
| Private Attributes | |
| string | m\_key |
| CfgSecureLevel | m\_level |

---

## Detailed Description

Base class for a configurator entry.

---

## Constructor & Destructor Documentation

|  |  |  |  |
| --- | --- | --- | --- |
| CfgBase::CfgBase | ( | const char \* | *a\_key*, |
|  |  | CfgSecureLevel | *a\_level* |
|  | ) |  |  |

References Configurator::Configurator(), g\_cfg, and Configurator::Register().

{

if ( NULL == g\_cfg ) {

g\_cfg = new Configurator;

}

m\_key = a\_key;

m\_level = a\_level;

g\_cfg->Register( this, a\_key );

}

|  |  |  |  |  |  |  |  |
| --- | --- | --- | --- | --- | --- | --- | --- |
| |  |  |  |  |  |  | | --- | --- | --- | --- | --- | --- | | CfgBase::~CfgBase | ( | void |  | ) |  | | virtual |

{

;

}

---

## Member Function Documentation

|  |  |  |  |  |  |  |  |
| --- | --- | --- | --- | --- | --- | --- | --- |
| |  |  |  |  |  |  | | --- | --- | --- | --- | --- | --- | | const string CfgBase::getkey | ( | void |  | ) |  | | inline |

{ return m\_key; }

|  |  |  |  |  |  |  |  |
| --- | --- | --- | --- | --- | --- | --- | --- |
| |  |  |  |  |  |  | | --- | --- | --- | --- | --- | --- | | CfgSecureLevel CfgBase::getlevel | ( | void |  | ) |  | | inline |

{ return m\_level; }

|  |  |  |  |  |  |  |  |
| --- | --- | --- | --- | --- | --- | --- | --- |
| |  |  |  |  |  |  | | --- | --- | --- | --- | --- | --- | | virtual CfgType CfgBase::gettype | ( | void |  | ) |  | | inlinevirtual |

Reimplemented in CfgStr, CfgBool, CfgFloat, and CfgInt.

References CFG\_NONE.

{ return CFG\_NONE; }

---

## Member Data Documentation

|  |  |  |
| --- | --- | --- |
| |  | | --- | | string CfgBase::m\_key | | private |

|  |  |  |
| --- | --- | --- |
| |  | | --- | | CfgSecureLevel CfgBase::m\_level | | private |

---

The documentation for this class was generated from the following files:

- configurator.h
- configurator.cpp


- CfgBase
- Generated on Thu Jan 10 2013 13:15:35 for ALMaSS Skylark ODdox by
   1.8.1.1
