# Supplementary material for: Modelling Skylarks (Alauda arvensis) to Predict Impacts of Changes in Land Management and Policy: Development and Testing of an Agent-Based Model
Source: PLoS One. 2013 Jun 6;8(6):e65803. doi: 10.1371/journal.pone.0065803 (PMC3675089; doi:10.1371/journal.pone.0065803)
Supplement: Supporting Information S4 — The skylark ODdox as a zipped archive. (ZIP) [file pone.0065803.s004.zip › Skylark_ODdox/class_cfg_base-members.html]

ALMaSS Skylark ODdox: Member List


|  |
| --- |
| ALMaSS Skylark ODdox  2.0 |


- Main Page
- Related Pages
- Classes
- Files

- Class List
- Class Index
- Class Hierarchy
- Class Members

CfgBase Member List

This is the complete list of members for CfgBase, including all inherited members.

|  |  |  |
| --- | --- | --- |
| CfgBase(const char \*a\_key, CfgSecureLevel a\_level) | CfgBase |  |
| getkey(void) | CfgBase | inline |
| getlevel(void) | CfgBase | inline |
| gettype(void) | CfgBase | inlinevirtual |
| m\_key | CfgBase | private |
| m\_level | CfgBase | private |
| ~CfgBase(void) | CfgBase | virtual |


- Generated on Thu Jan 10 2013 13:15:35 for ALMaSS Skylark ODdox by
   1.8.1.1
