# Supplementary material for: Modelling Skylarks (Alauda arvensis) to Predict Impacts of Changes in Land Management and Policy: Development and Testing of an Agent-Based Model
Source: PLoS One. 2013 Jun 6;8(6):e65803. doi: 10.1371/journal.pone.0065803 (PMC3675089; doi:10.1371/journal.pone.0065803)
Supplement: Supporting Information S4 — The skylark ODdox as a zipped archive. (ZIP) [file pone.0065803.s004.zip › Skylark_ODdox/class_cfg_bool.html]

ALMaSS Skylark ODdox: CfgBool Class Reference


|  |
| --- |
| ALMaSS Skylark ODdox  2.0 |


- Main Page
- Related Pages
- Classes
- Files

- Class List
- Class Index
- Class Hierarchy
- Class Members

Public Member Functions |
Private Attributes

CfgBool Class Reference

Bool configurator entry class.
More...

`#include <configurator.h>`

List of all members.

|  |  |
| --- | --- |
| Public Member Functions | |
|  | CfgBool (const char \*a\_key, CfgSecureLevel a\_level, bool a\_defval) |
| virtual CfgType | gettype (void) |
| void | set (bool a\_newval) |
| bool | value (void) |
| Public Member Functions inherited from CfgBase | |
|  | CfgBase (const char \*a\_key, CfgSecureLevel a\_level) |
| const string | getkey (void) |
| CfgSecureLevel | getlevel (void) |
| virtual | ~CfgBase (void) |

|  |  |
| --- | --- |
| Private Attributes | |
| bool | m\_bool |

---

## Detailed Description

Bool configurator entry class.

---

## Constructor & Destructor Documentation

|  |  |  |  |
| --- | --- | --- | --- |
| CfgBool::CfgBool | ( | const char \* | *a\_key*, |
|  |  | CfgSecureLevel | *a\_level*, |
|  |  | bool | *a\_defval* |
|  | ) |  |  |

References m\_bool.

:CfgBase( a\_key, a\_level )

{

m\_bool = a\_defval;

}

---

## Member Function Documentation

|  |  |  |  |  |  |  |  |
| --- | --- | --- | --- | --- | --- | --- | --- |
| |  |  |  |  |  |  | | --- | --- | --- | --- | --- | --- | | virtual CfgType CfgBool::gettype | ( | void |  | ) |  | | inlinevirtual |

Reimplemented from CfgBase.

References CFG\_BOOL.

{ return CFG\_BOOL; }

|  |  |  |  |  |  |  |  |
| --- | --- | --- | --- | --- | --- | --- | --- |
| |  |  |  |  |  |  | | --- | --- | --- | --- | --- | --- | | void CfgBool::set | ( | bool | *a\_newval* | ) |  | | inline |

{ m\_bool = a\_newval; }

|  |  |  |  |  |  |  |  |
| --- | --- | --- | --- | --- | --- | --- | --- |
| |  |  |  |  |  |  | | --- | --- | --- | --- | --- | --- | | bool CfgBool::value | ( | void |  | ) |  | | inline |

Referenced by Landscape::ChangeMapMapping(), Population\_Manager::CloseTheRipleysOutputProbe(), FarmManager::CreateFarms(), Landscape::DumpMap(), Configurator::DumpSymbols(), Landscape::DumpVegAreaData(), FarmManager::FarmManager(), Farm::GetFirstCropIndex(), Farm::GetNextCropIndex(), Farm::GetNextCropStartDate(), Farm::HandleEvents(), Farm::HerbicideTreat(), Skylark\_Population\_Manager::Init(), Farm::InitiateManagement(), Farm::InsecticideTreat(), Landscape::Landscape(), main(), Pesticide::Pesticide(), Landscape::PolysDump(), Population\_Manager::Population\_Manager(), Farm::ProductApplication0(), Farm::ProductApplication1(), RasterMap::RasterMap(), Landscape::ReadPolys(), Population\_Manager::Run(), Configurator::SetCfgGatekeeper(), Landscape::SupplyPesticide(), Landscape::SupplyRodenticide(), Farm::SynInsecticideTreat(), Pesticide::Tick(), Landscape::Tick(), Pesticide::TwinMapSpray(), VegElement::VegElement(), Landscape::~Landscape(), Pesticide::~Pesticide(), and Population\_Manager::~Population\_Manager().

{ return m\_bool; }

---

## Member Data Documentation

|  |  |  |
| --- | --- | --- |
| |  | | --- | | bool CfgBool::m\_bool | | private |

Referenced by CfgBool().

---

The documentation for this class was generated from the following files:

- configurator.h
- configurator.cpp


- CfgBool
- Generated on Thu Jan 10 2013 13:15:35 for ALMaSS Skylark ODdox by
   1.8.1.1
