# Supplementary material for: Modelling Skylarks (Alauda arvensis) to Predict Impacts of Changes in Land Management and Policy: Development and Testing of an Agent-Based Model
Source: PLoS One. 2013 Jun 6;8(6):e65803. doi: 10.1371/journal.pone.0065803 (PMC3675089; doi:10.1371/journal.pone.0065803)
Supplement: Supporting Information S4 — The skylark ODdox as a zipped archive. (ZIP) [file pone.0065803.s004.zip › Skylark_ODdox/class_cfg_float.html]

ALMaSS Skylark ODdox: CfgFloat Class Reference


|  |
| --- |
| ALMaSS Skylark ODdox  2.0 |


- Main Page
- Related Pages
- Classes
- Files

- Class List
- Class Index
- Class Hierarchy
- Class Members

Public Member Functions |
Private Attributes

CfgFloat Class Reference

Double configurator entry class.
More...

`#include <configurator.h>`

List of all members.

|  |  |
| --- | --- |
| Public Member Functions | |
|  | CfgFloat (const char \*a\_key, CfgSecureLevel a\_level, double a\_defval) |
| virtual CfgType | gettype (void) |
| void | set (double a\_newval) |
| double | value (void) |
| Public Member Functions inherited from CfgBase | |
|  | CfgBase (const char \*a\_key, CfgSecureLevel a\_level) |
| const string | getkey (void) |
| CfgSecureLevel | getlevel (void) |
| virtual | ~CfgBase (void) |

|  |  |
| --- | --- |
| Private Attributes | |
| double | m\_float |

---

## Detailed Description

Double configurator entry class.

---

## Constructor & Destructor Documentation

|  |  |  |  |
| --- | --- | --- | --- |
| CfgFloat::CfgFloat | ( | const char \* | *a\_key*, |
|  |  | CfgSecureLevel | *a\_level*, |
|  |  | double | *a\_defval* |
|  | ) |  |  |

References m\_float.

:CfgBase( a\_key, a\_level )

{

m\_float = a\_defval;

}

---

## Member Function Documentation

|  |  |  |  |  |  |  |  |
| --- | --- | --- | --- | --- | --- | --- | --- |
| |  |  |  |  |  |  | | --- | --- | --- | --- | --- | --- | | virtual CfgType CfgFloat::gettype | ( | void |  | ) |  | | inlinevirtual |

Reimplemented from CfgBase.

References CFG\_FLOAT.

{ return CFG\_FLOAT; }

|  |  |  |  |  |  |  |  |
| --- | --- | --- | --- | --- | --- | --- | --- |
| |  |  |  |  |  |  | | --- | --- | --- | --- | --- | --- | | void CfgFloat::set | ( | double | *a\_newval* | ) |  | | inline |

{ m\_float = a\_newval; }

|  |  |  |  |  |  |  |  |
| --- | --- | --- | --- | --- | --- | --- | --- |
| |  |  |  |  |  |  | | --- | --- | --- | --- | --- | --- | | double CfgFloat::value | ( | void |  | ) |  | | inline |

Referenced by Landscape::BeetleBankPossible(), Farm::CattleIsOut(), Farm::CattleIsOutLow(), Farm::CattleOut(), Farm::CattleOutLowGrazing(), Orchard::Cutting(), OrchardGrass::Cutting(), Pesticide::DiffusionFunction(), VegElement::DoDevelopment(), BeetleBank::DoDevelopment(), Orchard::DoDevelopment(), OrchardBand::DoDevelopment(), Configurator::DumpSymbols(), Skylark\_Adult::GetVegHindrance(), Landscape::hb\_Add(), Landscape::hb\_MarkTheBresenhamWay(), Skylark\_Population\_Manager::LoadParameters(), Pesticide::MainMapDecay(), Pesticide::Pesticide(), Farm::PigsAreOutForced(), Farm::PigsOut(), SkTerritories::PrePolyNQual(), SkTerritories::PreProcessLandscape2(), Farm::ProductApplication0(), Farm::ProductApplication1(), RodenticideManager::RodenticideManager(), RodenticideManager::ShouldPlaceBait(), Skylark\_Male::Skylark\_Male(), Skylark\_Nestling::Skylark\_Nestling(), SkTerritories::Split(), Skylark\_Clutch::st\_Developing(), Skylark\_Nestling::st\_Developing(), and Skylark\_Female::st\_Immigrating().

{ return m\_float; }

---

## Member Data Documentation

|  |  |  |
| --- | --- | --- |
| |  | | --- | | double CfgFloat::m\_float | | private |

Referenced by CfgFloat().

---

The documentation for this class was generated from the following files:

- configurator.h
- configurator.cpp


- CfgFloat
- Generated on Thu Jan 10 2013 13:15:35 for ALMaSS Skylark ODdox by
   1.8.1.1
