# Supplementary material for: Modelling Skylarks (Alauda arvensis) to Predict Impacts of Changes in Land Management and Policy: Development and Testing of an Agent-Based Model
Source: PLoS One. 2013 Jun 6;8(6):e65803. doi: 10.1371/journal.pone.0065803 (PMC3675089; doi:10.1371/journal.pone.0065803)
Supplement: Supporting Information S4 — The skylark ODdox as a zipped archive. (ZIP) [file pone.0065803.s004.zip › Skylark_ODdox/class_cfg_int.html]

ALMaSS Skylark ODdox: CfgInt Class Reference


|  |
| --- |
| ALMaSS Skylark ODdox  2.0 |


- Main Page
- Related Pages
- Classes
- Files

- Class List
- Class Index
- Class Hierarchy
- Class Members

Public Member Functions |
Private Attributes

CfgInt Class Reference

Integer configurator entry class.
More...

`#include <configurator.h>`

List of all members.

|  |  |
| --- | --- |
| Public Member Functions | |
|  | CfgInt (const char \*a\_key, CfgSecureLevel a\_level, int a\_defval) |
| virtual CfgType | gettype (void) |
| void | set (int a\_newval) |
| int | value (void) |
| Public Member Functions inherited from CfgBase | |
|  | CfgBase (const char \*a\_key, CfgSecureLevel a\_level) |
| const string | getkey (void) |
| CfgSecureLevel | getlevel (void) |
| virtual | ~CfgBase (void) |

|  |  |
| --- | --- |
| Private Attributes | |
| int | m\_int |

---

## Detailed Description

Integer configurator entry class.

---

## Constructor & Destructor Documentation

|  |  |  |  |
| --- | --- | --- | --- |
| CfgInt::CfgInt | ( | const char \* | *a\_key*, |
|  |  | CfgSecureLevel | *a\_level*, |
|  |  | int | *a\_defval* |
|  | ) |  |  |

References m\_int.

:CfgBase( a\_key, a\_level )

{

m\_int = a\_defval;

}

---

## Member Function Documentation

|  |  |  |  |  |  |  |  |
| --- | --- | --- | --- | --- | --- | --- | --- |
| |  |  |  |  |  |  | | --- | --- | --- | --- | --- | --- | | virtual CfgType CfgInt::gettype | ( | void |  | ) |  | | inlinevirtual |

Reimplemented from CfgBase.

References CFG\_INT.

{ return CFG\_INT; }

|  |  |  |  |  |  |  |  |
| --- | --- | --- | --- | --- | --- | --- | --- |
| |  |  |  |  |  |  | | --- | --- | --- | --- | --- | --- | | void CfgInt::set | ( | int | *a\_newval* | ) |  | | inline |

{ m\_int = a\_newval; }

|  |  |  |  |  |  |  |  |
| --- | --- | --- | --- | --- | --- | --- | --- |
| |  |  |  |  |  |  | | --- | --- | --- | --- | --- | --- | | int CfgInt::value | ( | void |  | ) |  | | inline |

Referenced by Landscape::AddBeetleBanks(), Landscape::BeetleBankAdd(), Landscape::BeetleBankPossible(), Population\_Manager::BeginningOfMonth(), Landscape::BuildingDesignationCalc(), Skylark\_Population\_Manager::Catastrophe(), Landscape::ChangeMapMapping(), FarmManager::CreateFarms(), Pesticide::DiffusionMaskInit(), Pesticide::DiffusionMaskInitTest(), Orchard::DoDevelopment(), OrchardBand::DoDevelopment(), OrchardGrass::DoDevelopment(), Skylark\_Population\_Manager::DoFirst(), RodenticideManager::DoPlaceBait(), Configurator::DumpSymbols(), Farm::HandleEvents(), Landscape::hb\_GenerateHBPolys(), Landscape::hb\_StripingDist(), Skylark\_Population\_Manager::Init(), Farm::InitiateManagement(), Landscape::Landscape(), Skylark\_Population\_Manager::LoadParameters(), RasterMap::Manipulation1(), Skylark\_Clutch::OnFarmEvent(), Skylark\_Nestling::OnFarmEvent(), Skylark\_PreFledgeling::OnFarmEvent(), Skylark\_Female::OnFarmEvent(), Skylark\_Male::OnFarmEvent(), Population\_Manager::Population\_Manager(), SkTerritories::PrePolyNQual(), Landscape::ReadPolys(), RodenticideManager::RodenticideManager(), Population\_Manager::Run(), Skylark\_Nestling::Skylark\_Nestling(), Skylark\_Population\_Manager::Skylark\_Population\_Manager(), RodenticideManager::Tick(), Landscape::Tick(), UnsprayedFieldMargin::UnsprayedFieldMargin(), Landscape::UnsprayedMarginAdd(), and Weather::Weather().

{ return m\_int; }

---

## Member Data Documentation

|  |  |  |
| --- | --- | --- |
| |  | | --- | | int CfgInt::m\_int | | private |

Referenced by CfgInt().

---

The documentation for this class was generated from the following files:

- configurator.h
- configurator.cpp


- CfgInt
- Generated on Thu Jan 10 2013 13:15:35 for ALMaSS Skylark ODdox by
   1.8.1.1
