# Supplementary material for: Modelling Skylarks (Alauda arvensis) to Predict Impacts of Changes in Land Management and Policy: Development and Testing of an Agent-Based Model
Source: PLoS One. 2013 Jun 6;8(6):e65803. doi: 10.1371/journal.pone.0065803 (PMC3675089; doi:10.1371/journal.pone.0065803)
Supplement: Supporting Information S4 — The skylark ODdox as a zipped archive. (ZIP) [file pone.0065803.s004.zip › Skylark_ODdox/class_cfg_str.html]

ALMaSS Skylark ODdox: CfgStr Class Reference


|  |
| --- |
| ALMaSS Skylark ODdox  2.0 |


- Main Page
- Related Pages
- Classes
- Files

- Class List
- Class Index
- Class Hierarchy
- Class Members

Public Member Functions |
Private Attributes

CfgStr Class Reference

String configurator entry class.
More...

`#include <configurator.h>`

List of all members.

|  |  |
| --- | --- |
| Public Member Functions | |
|  | CfgStr (const char \*a\_key, CfgSecureLevel a\_level, const char \*a\_defval) |
| virtual CfgType | gettype (void) |
| void | set (char \*a\_newval) |
| const char \* | value (void) |
| Public Member Functions inherited from CfgBase | |
|  | CfgBase (const char \*a\_key, CfgSecureLevel a\_level) |
| const string | getkey (void) |
| CfgSecureLevel | getlevel (void) |
| virtual | ~CfgBase (void) |

|  |  |
| --- | --- |
| Private Attributes | |
| string | m\_string |

---

## Detailed Description

String configurator entry class.

---

## Constructor & Destructor Documentation

|  |  |  |  |
| --- | --- | --- | --- |
| CfgStr::CfgStr | ( | const char \* | *a\_key*, |
|  |  | CfgSecureLevel | *a\_level*, |
|  |  | const char \* | *a\_defval* |
|  | ) |  |  |

References m\_string.

:CfgBase( a\_key, a\_level )

{

m\_string = a\_defval;

}

---

## Member Function Documentation

|  |  |  |  |  |  |  |  |
| --- | --- | --- | --- | --- | --- | --- | --- |
| |  |  |  |  |  |  | | --- | --- | --- | --- | --- | --- | | virtual CfgType CfgStr::gettype | ( | void |  | ) |  | | inlinevirtual |

Reimplemented from CfgBase.

References CFG\_STRING.

{ return CFG\_STRING; }

|  |  |  |  |  |  |  |  |
| --- | --- | --- | --- | --- | --- | --- | --- |
| |  |  |  |  |  |  | | --- | --- | --- | --- | --- | --- | | void CfgStr::set | ( | char \* | *a\_newval* | ) |  | | inline |

{ m\_string = a\_newval; }

|  |  |  |  |  |  |  |  |
| --- | --- | --- | --- | --- | --- | --- | --- |
| |  |  |  |  |  |  | | --- | --- | --- | --- | --- | --- | | const char\* CfgStr::value | ( | void |  | ) |  | | inline |

Referenced by CropRotation::CropRotation(), Configurator::DumpSymbols(), Landscape::DumpVegAreaData(), FarmManager::FarmManager(), Landscape::Landscape(), Population\_Manager::OpenTheCIPEGridOutputProbe(), Population\_Manager::OpenTheMonthlyRipleysOutputProbe(), Population\_Manager::OpenTheReallyBigProbe(), Population\_Manager::OpenTheRipleysOutputProbe(), VegElement::ReadBugPercentageFile(), and Landscape::~Landscape().

{ return m\_string.c\_str(); }

---

## Member Data Documentation

|  |  |  |
| --- | --- | --- |
| |  | | --- | | string CfgStr::m\_string | | private |

Referenced by CfgStr().

---

The documentation for this class was generated from the following files:

- configurator.h
- configurator.cpp


- CfgStr
- Generated on Thu Jan 10 2013 13:15:35 for ALMaSS Skylark ODdox by
   1.8.1.1
