# Supplementary material for: Modelling Skylarks (Alauda arvensis) to Predict Impacts of Changes in Land Management and Policy: Development and Testing of an Agent-Based Model
Source: PLoS One. 2013 Jun 6;8(6):e65803. doi: 10.1371/journal.pone.0065803 (PMC3675089; doi:10.1371/journal.pone.0065803)
Supplement: Supporting Information S4 — The skylark ODdox as a zipped archive. (ZIP) [file pone.0065803.s004.zip › Skylark_ODdox/class_compare_state.html]

ALMaSS Skylark ODdox: CompareState Class Reference


|  |
| --- |
| ALMaSS Skylark ODdox  2.0 |


- Main Page
- Related Pages
- Classes
- Files

- Class List
- Class Index
- Class Hierarchy
- Class Members

Public Member Functions

CompareState Class Reference

Function to compare to TAnimal's Current behavioural state.
More...

List of all members.

|  |  |
| --- | --- |
| Public Member Functions | |
| bool | operator() (TAnimal \*A1, TAnimal \*A2) const |

---

## Detailed Description

Function to compare to TAnimal's Current behavioural state.

NB WhatState must be reimplemented by all descendents of TAnimal that use this functionality (not all do)

---

## Member Function Documentation

|  |  |  |  |  |  |  |  |  |  |  |  |  |  |
| --- | --- | --- | --- | --- | --- | --- | --- | --- | --- | --- | --- | --- | --- |
| |  |  |  |  | | --- | --- | --- | --- | | bool CompareState::operator() | ( | TAnimal \* | *A1*, | |  |  | TAnimal \* | *A2* | |  | ) |  | const | | inline |

References TAnimal::WhatState().

{

return (A1->WhatState() < A2->WhatState());

}

---

The documentation for this class was generated from the following file:

- PopulationManager.cpp


- CompareState
- Generated on Thu Jan 10 2013 13:15:35 for ALMaSS Skylark ODdox by
   1.8.1.1
