# Supplementary material for: Modelling Skylarks (Alauda arvensis) to Predict Impacts of Changes in Land Management and Policy: Development and Testing of an Agent-Based Model
Source: PLoS One. 2013 Jun 6;8(6):e65803. doi: 10.1371/journal.pone.0065803 (PMC3675089; doi:10.1371/journal.pone.0065803)
Supplement: Supporting Information S4 — The skylark ODdox as a zipped archive. (ZIP) [file pone.0065803.s004.zip › Skylark_ODdox/class_configurator.html]

ALMaSS Skylark ODdox: Configurator Class Reference


|  |
| --- |
| ALMaSS Skylark ODdox  2.0 |


- Main Page
- Related Pages
- Classes
- Files

- Class List
- Class Index
- Class Hierarchy
- Class Members

Public Member Functions |
Private Member Functions |
Private Attributes

Configurator Class Reference

A class to provide standard parameter entry facilities.
More...

`#include <configurator.h>`

List of all members.

|  |  |
| --- | --- |
| Public Member Functions | |
|  | Configurator (void) |
| void | DumpAllSymbolsAndExit (const char \*a\_dumpfile) |
| void | DumpPublicSymbols (const char \*a\_dumpfile, CfgSecureLevel a\_level) |
| bool | ReadSymbols (const char \*a\_cfgfile) |
| bool | Register (CfgBase \*a\_cfgval, const char \*a\_key) |
|  | ~Configurator (void) |

|  |  |
| --- | --- |
| Private Member Functions | |
| void | DumpSymbols (const char \*a\_dumpfile, CfgSecureLevel a\_level) |
| char \* | ExtractString (char \*a\_line) |
| bool | LastDoubleQuote (char \*a\_rest\_of\_line) |
| void | ParseCfgLine (char \*a\_line) |
| void | SetCfgBool (char \*a\_key, char \*a\_val) |
| void | SetCfgFloat (char \*a\_key, char \*a\_val) |
| bool | SetCfgGatekeeper (const char \*a\_method, const char \*a\_key, CfgSecureLevel a\_level) |
| void | SetCfgInt (char \*a\_key, char \*a\_val) |
| void | SetCfgStr (char \*a\_key, char \*a\_val) |
| void | ShowIdType (unsigned int a\_i) |

|  |  |
| --- | --- |
| Private Attributes | |
| map< string, unsigned int > | CfgI |
| vector< CfgBase \* > | CfgVals |
| unsigned int | m\_lineno |

---

## Detailed Description

A class to provide standard parameter entry facilities.

---

## Constructor & Destructor Documentation

|  |  |  |  |  |  |
| --- | --- | --- | --- | --- | --- |
| Configurator::Configurator | ( | void |  | ) |  |

References m\_lineno.

Referenced by CfgBase::CfgBase().

{

m\_lineno = 0;

}

|  |  |  |  |  |  |
| --- | --- | --- | --- | --- | --- |
| Configurator::~Configurator | ( | void |  | ) |  |

{

;

}

---

## Member Function Documentation

|  |  |  |  |  |  |
| --- | --- | --- | --- | --- | --- |
| void Configurator::DumpAllSymbolsAndExit | ( | const char \* | *a\_dumpfile* | ) |  |

References CFG\_PRIVATE, and DumpSymbols().

Referenced by Landscape::DumpAllSymbolsAndExit().

{

DumpSymbols( a\_dumpfile, CFG\_PRIVATE );

exit(1);

}

|  |  |  |  |
| --- | --- | --- | --- |
| void Configurator::DumpPublicSymbols | ( | const char \* | *a\_dumpfile*, |
|  |  | CfgSecureLevel | *a\_level* |
|  | ) |  |  |

References CFG\_PUBLIC, and DumpSymbols().

Referenced by Landscape::DumpPublicSymbols().

{

if ( a\_level > CFG\_PUBLIC ) {

a\_level = CFG\_PUBLIC;

}

DumpSymbols( a\_dumpfile, a\_level );

}

|  |  |  |  |  |  |  |  |  |  |  |  |  |  |
| --- | --- | --- | --- | --- | --- | --- | --- | --- | --- | --- | --- | --- | --- |
| |  |  |  |  | | --- | --- | --- | --- | | void Configurator::DumpSymbols | ( | const char \* | *a\_dumpfile*, | |  |  | CfgSecureLevel | *a\_level* | |  | ) |  |  | | private |

References CFG\_BOOL, CFG\_FLOAT, CFG\_INT, CFG\_MAX\_LINE\_LENGTH, CFG\_STRING, CfgI, CfgSecureStrings, CfgTypeStrings, CfgVals, g\_msg, CfgInt::value(), CfgFloat::value(), CfgBool::value(), CfgStr::value(), MapErrorMsg::Warn(), and WARN\_FILE.

Referenced by DumpAllSymbolsAndExit(), and DumpPublicSymbols().

{

FILE \*l\_dumpfile;

char l\_oprefix[ CFG\_MAX\_LINE\_LENGTH ] = {""};

char l\_nprefix[ CFG\_MAX\_LINE\_LENGTH ];

const char\* l\_id;

l\_dumpfile=fopen( a\_dumpfile, "w" );

if (!l\_dumpfile) {

g\_msg->Warn( WARN\_FILE, "Configurator::DumpSymbols() "

"Unable to open file for writing:",

a\_dumpfile );

exit(1);

}

typedef map<string,unsigned int>::const\_iterator MI;

for ( MI ii = CfgI.begin(); ii != CfgI.end(); ii++ ) {

unsigned int i = ii->second;

// Skip 'secret' variables.

if ( CfgVals[ i ]->getlevel() > a\_level ) {

continue;

}

// Weird hack to separate different groups of

// configuration names.

string rubbish=CfgVals[ i ]->getkey();

l\_id=rubbish.c\_str();

//l\_id = CfgVals[ i ]->getkey().c\_str();

sscanf( l\_id, "%[A-Z]", l\_nprefix );

if ( strcmp( l\_oprefix, l\_nprefix ) != 0 ) {

fprintf( l\_dumpfile, "\n" );

strcpy( l\_oprefix, l\_nprefix );

}

fprintf( l\_dumpfile, "%s (%s) = ",

l\_id,

CfgTypeStrings[ CfgVals[ i ]->gettype() ]

);

switch( CfgVals[ i ]->gettype() ) {

case CFG\_INT:

{

CfgInt\* l\_p = dynamic\_cast<CfgInt\*>(CfgVals[ i ]);

fprintf( l\_dumpfile, "%d", l\_p->value() );

break;

}

case CFG\_FLOAT:

{

CfgFloat\* l\_p = dynamic\_cast<CfgFloat\*>(CfgVals[ i ]);

fprintf( l\_dumpfile, "%f", l\_p->value() );

break;

}

case CFG\_BOOL:

{

CfgBool\* l\_p = dynamic\_cast<CfgBool\*>(CfgVals[ i ]);

if ( l\_p->value() ) {

fprintf( l\_dumpfile, "true" );

} else {

fprintf( l\_dumpfile, "false" );

}

break;

}

case CFG\_STRING:

{

CfgStr\* l\_p = dynamic\_cast<CfgStr\*>(CfgVals[ i ]);

fprintf( l\_dumpfile, "\"%s\"", l\_p->value() );

break;

}

default:

{

char l\_errno[20];

sprintf( l\_errno, "%d", CfgVals[ i ]->gettype() );

g\_msg->Warn( WARN\_FILE, "Configurator::DumpSymbols() "

"Unknown symbol type read:",

l\_errno );

exit(1);

}

}

fprintf( l\_dumpfile, " # %s\n",

CfgSecureStrings[ CfgVals[ i ]->getlevel() ]

);

}

}

|  |  |  |  |  |  |  |  |
| --- | --- | --- | --- | --- | --- | --- | --- |
| |  |  |  |  |  |  | | --- | --- | --- | --- | --- | --- | | char \* Configurator::ExtractString | ( | char \* | *a\_line* | ) |  | | private |

References g\_msg, LastDoubleQuote(), m\_lineno, MapErrorMsg::Warn(), and WARN\_FILE.

Referenced by ParseCfgLine().

{

char lineno[ 20 ];

// scan for the first double quote or end of line.

while ( \*a\_line != '"' && \*a\_line != '\0' ) {

a\_line++;

}

// The first char in the string had better contain a '"':

if ( \*a\_line != '"' ) {

sprintf( lineno, "%d", m\_lineno );

g\_msg->Warn( WARN\_FILE, "Configurator::ExtractString()\n"

" String not enclosed in double quotes at "

"config line ", lineno );

exit(1);

}

char\* endline = ++a\_line;

bool escaped = false, found = false;

while ( \*endline != '\0' ) {

if ( \*endline == '\\' ) {

escaped = true;

endline++;

if ( \*endline == '"' &&

LastDoubleQuote( endline )) {

escaped = false;

} else {

continue;

}

}

if ( \*endline == '"' && !escaped ) {

// Found end of string, terminate properly and break the loop.

\*endline++ = '\0';

found = true;

break;

}

escaped = false;

endline++;

}

if ( !found ) {

sprintf( lineno, "%d", m\_lineno );

g\_msg->Warn( WARN\_FILE, "Configurator::ExtractString() "

"No ending double quote after string at "

"config line ", lineno );

exit(1);

}

// Check for comment if remainder of line isn't empty.

if ( sscanf( endline, "%\*s" ) == 1 ) {

// Non-empty comment line.

if ( sscanf( endline, "%\*[#]" ) != 1 ) {

// But not initiated by '#'.

sprintf( lineno, "%d", m\_lineno );

g\_msg->Warn( WARN\_FILE, "Configurator::ExtractString() "

"Illegal comment at "

"config line ", lineno );

exit(1);

}

}

return a\_line;

}

|  |  |  |  |  |  |  |  |
| --- | --- | --- | --- | --- | --- | --- | --- |
| |  |  |  |  |  |  | | --- | --- | --- | --- | --- | --- | | bool Configurator::LastDoubleQuote | ( | char \* | *a\_rest\_of\_line* | ) |  | | private |

Referenced by ExtractString().

{

a\_rest\_of\_line++;

while ( \*a\_rest\_of\_line != '\0' && \*a\_rest\_of\_line != '#' ) {

if ( \*a\_rest\_of\_line == '"' ) {

return false;

}

a\_rest\_of\_line++;

}

return true;

}

|  |  |  |  |  |  |  |  |
| --- | --- | --- | --- | --- | --- | --- | --- |
| |  |  |  |  |  |  | | --- | --- | --- | --- | --- | --- | | void Configurator::ParseCfgLine | ( | char \* | *a\_line* | ) |  | | private |

References CFG\_MAX\_LINE\_LENGTH, CfgI, ExtractString(), g\_msg, m\_lineno, SetCfgBool(), SetCfgFloat(), SetCfgInt(), SetCfgStr(), MapErrorMsg::Warn(), and WARN\_FILE.

Referenced by ReadSymbols().

{

char l\_id [ CFG\_MAX\_LINE\_LENGTH ];

char l\_type[ CFG\_MAX\_LINE\_LENGTH ];

char l\_sep [ CFG\_MAX\_LINE\_LENGTH ];

char l\_val [ CFG\_MAX\_LINE\_LENGTH ];

char l\_comm[ CFG\_MAX\_LINE\_LENGTH ];

char lineno[20];

if ( sscanf( a\_line, "%[#]", l\_id ) == 1 ) {

// Comment line.

return;

}

if ( sscanf( a\_line, "%s", l\_id) == EOF ) {

// Empty line consisting only of white spaces.

return;

}

//int l\_conv = sscanf( a\_line, "%[A-Z\_] (%[a-z]) %s", l\_id, sizeof(l\_id),l\_type,sizeof(l\_type), l\_sep,sizeof(l\_sep) );

int l\_conv = sscanf( a\_line, "%[A-Z\_] (%[a-z]) %s", l\_id, l\_type, l\_sep );

if ( l\_conv < 3 ) {

// Syntax terror.

sprintf( lineno, "%d", m\_lineno );

g\_msg->Warn( WARN\_FILE, "Configurator::ParseCfgLine() "

"Syntax error at config line ",

lineno );

exit(1);

}

if ( strcmp( l\_sep, "=" ) != 0 ) {

// Missing '=' assignment separator.

sprintf( lineno, "%d", m\_lineno );

g\_msg->Warn( WARN\_FILE, "Configurator::ParseCfgLine() "

"Missing '=' assignment operator at config line ",

lineno );

exit(1);

}

if ( CfgI.find( l\_id ) == CfgI.end() ) {

// Key doesn't exists among the predefined, global configuration

// variables. Ignore quietly.

return;

}

if ( strlen( l\_type ) == 6 &&

strncmp( l\_type, "string", 6 ) == 0 ) {

// We are not yet ready to do the assignment.

// If we really have a string enclosed in non-escaped

// double quotes at the end of the line, then we need to

// extract it first from our input.

SetCfgStr( l\_id, ExtractString( a\_line ));

return;

}

// Not a string, so extract data value and possible comment.

l\_conv = sscanf( a\_line, "%\*[A-Z\_] (%\*[a-z]) %\*s %s %s",

l\_val, l\_comm );

if ( l\_conv == 2 && l\_comm[0] != '#' ) {

// Illegal comment at end of line.

sprintf( lineno, "%d", m\_lineno );

g\_msg->Warn( WARN\_FILE, "Configurator::ParseCfgLine() "

"Syntax error at end of config line ",

lineno );

exit(1);

}

if ( strlen( l\_type ) == 5 &&

strncmp( l\_type, "float", 5 ) == 0 ) {

SetCfgFloat( l\_id, l\_val );

return;

}

if ( strlen( l\_type ) == 4 &&

strncmp( l\_type, "bool", 4 ) == 0 ) {

SetCfgBool( l\_id, l\_val );

return;

}

if ( strlen( l\_type ) == 3 &&

strncmp( l\_type, "int", 3 ) == 0 ) {

SetCfgInt( l\_id, l\_val );

return;

}

sprintf( lineno, "%d", m\_lineno );

g\_msg->Warn( WARN\_FILE, "Configurator::ParseCfgLine() "

"Unknown type specifier at config line ",

lineno );

exit(1);

}

|  |  |  |  |  |  |
| --- | --- | --- | --- | --- | --- |
| bool Configurator::ReadSymbols | ( | const char \* | *a\_cfgfile* | ) |  |

References CFG\_MAX\_LINE\_LENGTH, g\_msg, m\_lineno, ParseCfgLine(), MapErrorMsg::Warn(), and WARN\_FILE.

Referenced by Landscape::Landscape(), and Landscape::ReadSymbols().

{

ifstream cf\_file;

char cfgline[ CFG\_MAX\_LINE\_LENGTH ];

cf\_file.open(a\_cfgfile,fstream::in);

if ( !cf\_file.is\_open() ) {

g\_msg->Warn( WARN\_FILE, "Configurator::ReadSymbols() Unable to open file for reading: ", a\_cfgfile );

exit(1);

}

while ( !cf\_file.eof()) {

for (unsigned i=0; i< CFG\_MAX\_LINE\_LENGTH; i++) cfgline[i]=' '; // Done to get rid of the rubbish that otherwise messes up the parse

cf\_file.getline(cfgline,CFG\_MAX\_LINE\_LENGTH);

ParseCfgLine( cfgline );

m\_lineno++;

}

cf\_file.close();

return true;

}

|  |  |  |  |
| --- | --- | --- | --- |
| bool Configurator::Register | ( | CfgBase \* | *a\_cfgval*, |
|  |  | const char \* | *a\_key* |
|  | ) |  |  |

References CfgI, and CfgVals.

Referenced by CfgBase::CfgBase().

{

string l\_key = a\_key;

if ( CfgI.find( l\_key ) != CfgI.end() ) {

// Couldn't register, already exists.

return false;

}

unsigned int i = (int) CfgVals.size();

CfgI[ l\_key ] = i;

CfgVals.resize( i+1 );

CfgVals[ i ] = a\_cfgval;

return true;

}

|  |  |  |  |  |  |  |  |  |  |  |  |  |  |
| --- | --- | --- | --- | --- | --- | --- | --- | --- | --- | --- | --- | --- | --- |
| |  |  |  |  | | --- | --- | --- | --- | | void Configurator::SetCfgBool | ( | char \* | *a\_key*, | |  |  | char \* | *a\_val* | |  | ) |  |  | | private |

References CFG\_BOOL, CfgI, CfgVals, g\_msg, m\_lineno, SetCfgGatekeeper(), ShowIdType(), MapErrorMsg::Warn(), and WARN\_FILE.

Referenced by ParseCfgLine().

{

char lineno[20];

string l\_key = a\_key;

bool l\_val = false;

if ( strcmp ( a\_val, "false" ) == 0 ) {

; // l\_val defaults to false.

} else if ( strcmp ( a\_val, "true" ) == 0 ) {

l\_val = true;

} else {

sprintf( lineno, "%d", m\_lineno );

g\_msg->Warn( WARN\_FILE, "Configurator::SetCfgBool() "

"Not a boolean data value at config line",

lineno );

exit(1);

}

// Check access security.

unsigned int i = CfgI[ l\_key ];

if ( SetCfgGatekeeper( "Configurator::SetCfgBool() "

"Attempting to set public config variable in line",

a\_key,

CfgVals[ i ]->getlevel()

)) {

return;

}

if ( CfgVals[ i ]->gettype() != CFG\_BOOL ) {

sprintf( lineno, "%d", m\_lineno );

g\_msg->Warn( WARN\_FILE, "Configurator::SetCfgBool() "

"Non-boolean identifier specified at config line",

lineno );

ShowIdType( i );

exit(1);

}

dynamic\_cast<CfgBool\*>(CfgVals[ i ])->set( l\_val );

}

|  |  |  |  |  |  |  |  |  |  |  |  |  |  |
| --- | --- | --- | --- | --- | --- | --- | --- | --- | --- | --- | --- | --- | --- |
| |  |  |  |  | | --- | --- | --- | --- | | void Configurator::SetCfgFloat | ( | char \* | *a\_key*, | |  |  | char \* | *a\_val* | |  | ) |  |  | | private |

References CFG\_FLOAT, CfgI, CfgVals, FloatToDouble(), g\_msg, m\_lineno, SetCfgGatekeeper(), ShowIdType(), MapErrorMsg::Warn(), and WARN\_FILE.

Referenced by ParseCfgLine().

{

double l\_val;

float f;

char lineno[20];

string l\_key = a\_key;

if ( sscanf( a\_val, "%f", &f) != 1 ) {

sprintf( lineno, "%d", m\_lineno );

g\_msg->Warn( WARN\_FILE, "Configurator::SetCfgFloat() "

"Not a floating point data value at config line",

lineno );

exit(1);

}

FloatToDouble(l\_val,f);

// Check access security.

unsigned int i = CfgI[ l\_key ];

if ( SetCfgGatekeeper( "Configurator::SetCfgFloat() "

"Attempting to set public config variable in line",

a\_key,

CfgVals[ i ]->getlevel()

)) {

return;

}

if ( CfgVals[ i ]->gettype() != CFG\_FLOAT ) {

sprintf( lineno, "%d", m\_lineno );

g\_msg->Warn( WARN\_FILE, "Configurator::SetCfgFloat() "

"Non-floating point identifier specified at config line",

lineno );

ShowIdType( i );

exit(1);

}

dynamic\_cast<CfgFloat\*>(CfgVals[ i ])->set( l\_val );

}

|  |  |  |  |  |  |  |  |  |  |  |  |  |  |  |  |  |  |
| --- | --- | --- | --- | --- | --- | --- | --- | --- | --- | --- | --- | --- | --- | --- | --- | --- | --- |
| |  |  |  |  | | --- | --- | --- | --- | | bool Configurator::SetCfgGatekeeper | ( | const char \* | *a\_method*, | |  |  | const char \* | *a\_key*, | |  |  | CfgSecureLevel | *a\_level* | |  | ) |  |  | | private |

References CFG\_PRIVATE, CFG\_PUBLIC, g\_msg, l\_cfg\_public\_exit\_on\_set, l\_cfg\_public\_warn\_on\_set, m\_lineno, CfgBool::value(), MapErrorMsg::Warn(), and WARN\_FILE.

Referenced by SetCfgBool(), SetCfgFloat(), SetCfgInt(), and SetCfgStr().

{

if ( a\_level == CFG\_PRIVATE ) {

// Attempting to set private config variable. Ignore quietly.

return true;

}

if ( a\_level == CFG\_PUBLIC &&

l\_cfg\_public\_warn\_on\_set.value()) {

// Attempting to set public config variable. Warn and

// possibly exit if this is configured.

char lineno[20];

sprintf( lineno, "%d", m\_lineno );

g\_msg->Warn( WARN\_FILE, a\_method, lineno );

if ( l\_cfg\_public\_exit\_on\_set.value()) {

exit(1);

}

return true;

}

return false;

}

|  |  |  |  |  |  |  |  |  |  |  |  |  |  |
| --- | --- | --- | --- | --- | --- | --- | --- | --- | --- | --- | --- | --- | --- |
| |  |  |  |  | | --- | --- | --- | --- | | void Configurator::SetCfgInt | ( | char \* | *a\_key*, | |  |  | char \* | *a\_val* | |  | ) |  |  | | private |

References CFG\_INT, CfgI, CfgVals, g\_msg, m\_lineno, SetCfgGatekeeper(), ShowIdType(), MapErrorMsg::Warn(), and WARN\_FILE.

Referenced by ParseCfgLine().

{

int l\_val;

char lineno[20];

string l\_key = a\_key;

if ( sscanf( a\_val, "%d", &l\_val ) != 1 ) {

sprintf( lineno, "%d", m\_lineno );

g\_msg->Warn( WARN\_FILE, "Configurator::SetCfgInt() "

"Not an integer data value at config line",

lineno );

exit(1);

}

// Check access security.

unsigned int i = CfgI[ l\_key ];

if ( SetCfgGatekeeper( "Configurator::SetCfgInt() "

"Attempting to set public config variable in line",

a\_key,

CfgVals[ i ]->getlevel()

)) {

return;

}

if ( CfgVals[ i ]->gettype() != CFG\_INT ) {

sprintf( lineno, "%d", m\_lineno );

g\_msg->Warn( WARN\_FILE, "Configurator::SetCfgInt() "

"Non-integer identifier specified at config line",

lineno );

ShowIdType( i );

exit(1);

}

dynamic\_cast<CfgInt\*>(CfgVals[ i ])->set( l\_val );

}

|  |  |  |  |  |  |  |  |  |  |  |  |  |  |
| --- | --- | --- | --- | --- | --- | --- | --- | --- | --- | --- | --- | --- | --- |
| |  |  |  |  | | --- | --- | --- | --- | | void Configurator::SetCfgStr | ( | char \* | *a\_key*, | |  |  | char \* | *a\_val* | |  | ) |  |  | | private |

References CFG\_STRING, CfgI, CfgVals, g\_msg, m\_lineno, SetCfgGatekeeper(), ShowIdType(), MapErrorMsg::Warn(), and WARN\_FILE.

Referenced by ParseCfgLine().

{

char lineno[20];

string l\_key = a\_key;

// Check access security.

unsigned int i = CfgI[ l\_key ];

if ( SetCfgGatekeeper( "Configurator::SetCfgStr() "

"Attempting to set public config variable in line",

a\_key,

CfgVals[ i ]->getlevel()

)) {

return;

}

if ( CfgVals[ i ]->gettype() != CFG\_STRING ) {

sprintf( lineno, "%d", m\_lineno );

g\_msg->Warn( WARN\_FILE, "Configurator::SetCfgStr() "

"Non-string identifier specified at config line",

lineno );

ShowIdType( i );

exit(1);

}

dynamic\_cast<CfgStr\*>(CfgVals[ i ])->set( a\_val );

}

|  |  |  |  |  |  |  |  |
| --- | --- | --- | --- | --- | --- | --- | --- |
| |  |  |  |  |  |  | | --- | --- | --- | --- | --- | --- | | void Configurator::ShowIdType | ( | unsigned int | *a\_i* | ) |  | | private |

References CfgTypeStrings, CfgVals, g\_msg, WARN\_FILE, and MapErrorMsg::WarnAddInfo().

Referenced by SetCfgBool(), SetCfgFloat(), SetCfgInt(), and SetCfgStr().

{

g\_msg->WarnAddInfo( WARN\_FILE,

"Type for identifier ",

CfgVals[ a\_i ]->getkey().c\_str() );

g\_msg->WarnAddInfo( WARN\_FILE, " is (",

CfgTypeStrings[ CfgVals[ a\_i ]->gettype() ] );

g\_msg->WarnAddInfo( WARN\_FILE, ")\n", "" );

}

---

## Member Data Documentation

|  |  |  |
| --- | --- | --- |
| |  | | --- | | map<string,unsigned int> Configurator::CfgI | | private |

Referenced by DumpSymbols(), ParseCfgLine(), Register(), SetCfgBool(), SetCfgFloat(), SetCfgInt(), and SetCfgStr().

|  |  |  |
| --- | --- | --- |
| |  | | --- | | vector<CfgBase\*> Configurator::CfgVals | | private |

Referenced by DumpSymbols(), Register(), SetCfgBool(), SetCfgFloat(), SetCfgInt(), SetCfgStr(), and ShowIdType().

|  |  |  |
| --- | --- | --- |
| |  | | --- | | unsigned int Configurator::m\_lineno | | private |

Referenced by Configurator(), ExtractString(), ParseCfgLine(), ReadSymbols(), SetCfgBool(), SetCfgFloat(), SetCfgGatekeeper(), SetCfgInt(), and SetCfgStr().

---

The documentation for this class was generated from the following files:

- configurator.h
- configurator.cpp


- Configurator
- Generated on Thu Jan 10 2013 13:15:35 for ALMaSS Skylark ODdox by
   1.8.1.1
