# Supplementary material for: Modelling Skylarks (Alauda arvensis) to Predict Impacts of Changes in Land Management and Policy: Development and Testing of an Agent-Based Model
Source: PLoS One. 2013 Jun 6;8(6):e65803. doi: 10.1371/journal.pone.0065803 (PMC3675089; doi:10.1371/journal.pone.0065803)
Supplement: Supporting Information S4 — The skylark ODdox as a zipped archive. (ZIP) [file pone.0065803.s004.zip › Skylark_ODdox/class_configurator-members.html]

ALMaSS Skylark ODdox: Member List


|  |
| --- |
| ALMaSS Skylark ODdox  2.0 |


- Main Page
- Related Pages
- Classes
- Files

- Class List
- Class Index
- Class Hierarchy
- Class Members

Configurator Member List

This is the complete list of members for Configurator, including all inherited members.

|  |  |  |
| --- | --- | --- |
| CfgI | Configurator | private |
| CfgVals | Configurator | private |
| Configurator(void) | Configurator |  |
| DumpAllSymbolsAndExit(const char \*a\_dumpfile) | Configurator |  |
| DumpPublicSymbols(const char \*a\_dumpfile, CfgSecureLevel a\_level) | Configurator |  |
| DumpSymbols(const char \*a\_dumpfile, CfgSecureLevel a\_level) | Configurator | private |
| ExtractString(char \*a\_line) | Configurator | private |
| LastDoubleQuote(char \*a\_rest\_of\_line) | Configurator | private |
| m\_lineno | Configurator | private |
| ParseCfgLine(char \*a\_line) | Configurator | private |
| ReadSymbols(const char \*a\_cfgfile) | Configurator |  |
| Register(CfgBase \*a\_cfgval, const char \*a\_key) | Configurator |  |
| SetCfgBool(char \*a\_key, char \*a\_val) | Configurator | private |
| SetCfgFloat(char \*a\_key, char \*a\_val) | Configurator | private |
| SetCfgGatekeeper(const char \*a\_method, const char \*a\_key, CfgSecureLevel a\_level) | Configurator | private |
| SetCfgInt(char \*a\_key, char \*a\_val) | Configurator | private |
| SetCfgStr(char \*a\_key, char \*a\_val) | Configurator | private |
| ShowIdType(unsigned int a\_i) | Configurator | private |
| ~Configurator(void) | Configurator |  |


- Generated on Thu Jan 10 2013 13:15:35 for ALMaSS Skylark ODdox by
   1.8.1.1
