# Supplementary material for: Modelling Skylarks (Alauda arvensis) to Predict Impacts of Changes in Land Management and Policy: Development and Testing of an Agent-Based Model
Source: PLoS One. 2013 Jun 6;8(6):e65803. doi: 10.1371/journal.pone.0065803 (PMC3675089; doi:10.1371/journal.pone.0065803)
Supplement: Supporting Information S4 — The skylark ODdox as a zipped archive. (ZIP) [file pone.0065803.s004.zip › Skylark_ODdox/class_conventional_plant-members.html]

ALMaSS Skylark ODdox: Member List


|  |
| --- |
| ALMaSS Skylark ODdox  2.0 |


- Main Page
- Related Pages
- Classes
- Files

- Class List
- Class Index
- Class Hierarchy
- Class Members

ConventionalPlant Member List

This is the complete list of members for ConventionalPlant, including all inherited members.

|  |  |  |
| --- | --- | --- |
| AddField(LE \*a\_newfield) | Farm |  |
| AddNewEvent(TTypesOfVegetation a\_event, long a\_date, LE \*a\_field, int a\_todo, long a\_num, bool a\_lock, int a\_start, bool a\_first\_year, TTypesOfVegetation a\_crop) | Farm |  |
| AutumnHarrow(LE \*a\_field, double a\_user, int a\_days) | Farm | virtual |
| AutumnPlough(LE \*a\_field, double a\_user, int a\_days) | Farm | virtual |
| AutumnRoll(LE \*a\_field, double a\_user, int a\_days) | Farm | virtual |
| AutumnSow(LE \*a\_field, double a\_user, int a\_days) | Farm | virtual |
| BurnStrawStubble(LE \*a\_field, double a\_user, int a\_days) | Farm | virtual |
| CattleIsOut(LE \*a\_field, double a\_user, int a\_days, int a\_max) | Farm | virtual |
| CattleIsOutLow(LE \*a\_field, double a\_user, int a\_days, int a\_max) | Farm | virtual |
| CattleOut(LE \*a\_field, double a\_user, int a\_days) | Farm | virtual |
| CattleOutLowGrazing(LE \*a\_field, double a\_user, int a\_days) | Farm | virtual |
| CheckRotationManagementLoop(FarmEvent \*ev) | Farm | protected |
| ConventionalPlant(void) | ConventionalPlant |  |
| CutToHay(LE \*a\_field, double a\_user, int a\_days) | Farm | virtual |
| CutToSilage(LE \*a\_field, double a\_user, int a\_days) | Farm | virtual |
| CutWeeds(LE \*a\_field, double a\_user, int a\_days) | Farm | virtual |
| DeepPlough(LE \*a\_field, double a\_user, int a\_days) | Farm | virtual |
| DoIt(int a\_probability) | Farm |  |
| FA\_AmmoniumSulphate(LE \*a\_field, double a\_user, int a\_days) | Farm | virtual |
| FA\_GreenManure(LE \*a\_field, double a\_user, int a\_days) | Farm | virtual |
| FA\_Manure(LE \*a\_field, double a\_user, int a\_days) | Farm | virtual |
| FA\_NPK(LE \*a\_field, double a\_user, int a\_days) | Farm | virtual |
| FA\_PK(LE \*a\_field, double a\_user, int a\_days) | Farm | virtual |
| FA\_Sludge(LE \*a\_field, double a\_user, int a\_days) | Farm | virtual |
| FA\_Slurry(LE \*a\_field, double a\_user, int a\_days) | Farm | virtual |
| Farm(void) | Farm |  |
| FP\_GreenManure(LE \*a\_field, double a\_user, int a\_days) | Farm | virtual |
| FP\_LiquidNH3(LE \*a\_field, double a\_user, int a\_days) | Farm | virtual |
| FP\_ManganeseSulphate(LE \*a\_field, double a\_user, int a\_days) | Farm | virtual |
| FP\_Manure(LE \*a\_field, double a\_user, int a\_days) | Farm | virtual |
| FP\_NPK(LE \*a\_field, double a\_user, int a\_days) | Farm | virtual |
| FP\_NPKS(LE \*a\_field, double a\_user, int a\_days) | Farm | virtual |
| FP\_PK(LE \*a\_field, double a\_user, int a\_days) | Farm | virtual |
| FP\_Sludge(LE \*a\_field, double a\_user, int a\_days) | Farm | virtual |
| FP\_Slurry(LE \*a\_field, double a\_user, int a\_days) | Farm | virtual |
| FungicideTreat(LE \*a\_field, double a\_user, int a\_days) | Farm | virtual |
| GetArea(void) | Farm |  |
| GetFarmNumber(void) | Farm | inline |
| GetFirstCropIndex(TTypesOfLandscapeElement a\_type) | Farm | protectedvirtual |
| GetFirstDate(TTypesOfVegetation a\_tov) | Farm | protected |
| GetIntensity(void) | Farm | inline |
| GetNextCropIndex(int a\_rot\_index) | Farm | protectedvirtual |
| GetNextCropStartDate(LE \*a\_field, TTypesOfVegetation &a\_curr\_veg) | Farm | protected |
| GetType(void) | Farm | inline |
| Glyphosate(LE \*a\_field, double a\_user, int a\_days) | Farm | virtual |
| GrowthRegulator(LE \*a\_field, double a\_user, int a\_days) | Farm | virtual |
| HandleEvents(void) | Farm | protected |
| Harvest(LE \*a\_field, double a\_user, int a\_days) | Farm | virtual |
| HayBailing(LE \*a\_field, double a\_user, int a\_days) | Farm | virtual |
| HayTurning(LE \*a\_field, double a\_user, int a\_days) | Farm | virtual |
| HerbicideTreat(LE \*a\_field, double a\_user, int a\_days) | Farm | virtual |
| HillingUp(LE \*a\_field, double a\_user, int a\_days) | Farm | virtual |
| InitiateManagement(void) | Farm | virtual |
| InsecticideTreat(LE \*a\_field, double a\_user, int a\_days) | Farm | virtual |
| IsStockFarmer(void) | Farm | inline |
| LeSwitch(FarmEvent \*ev) | Farm | protected |
| m\_agrochemindustrycereal | Farm | protected |
| m\_carrots | Farm | protected |
| m\_CGG1 | Farm | protected |
| m\_CGG2 | Farm | protected |
| m\_farm\_num | Farm | protected |
| m\_farmtype | Farm | protected |
| m\_fieldpeas | Farm | protected |
| m\_fieldpeasstrigling | Farm | protected |
| m\_fields | Farm | protected |
| m\_fodderbeet | Farm | protected |
| m\_foddergrass | Farm | protected |
| m\_intensity | Farm | protected |
| m\_maize | Farm | protected |
| m\_maizesilage | Farm | protected |
| m\_maizestrigling | Farm | protected |
| m\_oats | Farm | protected |
| m\_OBarleyPCG | Farm | protected |
| m\_ocarrots | Farm | protected |
| m\_OCGG1 | Farm | protected |
| m\_OCGG2 | Farm | protected |
| m\_OCGS1 | Farm | protected |
| m\_ofieldpeas | Farm | protected |
| m\_ofieldpeassilage | Farm | protected |
| m\_ofirstyeardanger | Farm | protected |
| m\_ograzingpigs | Farm | protected |
| m\_omaizesilage | Farm | protected |
| m\_ooats | Farm | protected |
| m\_opermgrassgrazed | Farm | protected |
| m\_opotatoes | Farm | protected |
| m\_osbarleysilage | Farm | protected |
| m\_ospringbarley | Farm | protected |
| m\_ospringbarleyext | Farm | protected |
| m\_ospringbarleypigs | Farm | protected |
| m\_owinterbarley | Farm | protected |
| m\_owinterbarleyext | Farm | protected |
| m\_owinterrape | Farm | protected |
| m\_owinterrye | Farm | protected |
| m\_owinterwheatundersown | Farm | protected |
| m\_owinterwheatundersownext | Farm | protected |
| m\_permanentsetaside | Farm | protected |
| m\_PermCrops | Farm | protected |
| m\_permgrassgrazed | Farm | protected |
| m\_permgrasslowyield | Farm | protected |
| m\_permgrasstussocky | Farm | protected |
| m\_potatoes | Farm | protected |
| m\_potatoesindustry | Farm | protected |
| m\_queue | Farm | protected |
| m\_rotation | Farm | protected |
| m\_rotation\_sync\_index | Farm | protected |
| m\_sbarleyclovergrass | Farm | protected |
| m\_seedgrass1 | Farm | protected |
| m\_seedgrass2 | Farm | protected |
| m\_setaside | Farm | protected |
| m\_springbarley | Farm | protected |
| m\_springbarleyclovergrassstrigling | Farm | protected |
| m\_springbarleypeaclovergrassstrigling | Farm | protected |
| m\_springbarleyptreatment | Farm | protected |
| m\_springbarleyseed | Farm | protected |
| m\_springbarleysilage | Farm | protected |
| m\_springbarleyskmanagement | Farm | protected |
| m\_springbarleystrigling | Farm | protected |
| m\_springbarleystriglingculm | Farm | protected |
| m\_springbarleystriglingsingle | Farm | protected |
| m\_springrape | Farm | protected |
| m\_stockfarmer | Farm | protected |
| m\_triticale | Farm | protected |
| m\_winterbarley | Farm | protected |
| m\_winterbarleystrigling | Farm | protected |
| m\_winterrape | Farm | protected |
| m\_winterrapestrigling | Farm | protected |
| m\_winterrye | Farm | protected |
| m\_winterryestrigling | Farm | protected |
| m\_winterwheat | Farm | protected |
| m\_winterwheatstrigling | Farm | protected |
| m\_winterwheatstriglingculm | Farm | protected |
| m\_winterwheatstriglingsingle | Farm | protected |
| m\_wwheatpcontrol | Farm | protected |
| m\_wwheatptoxiccontrol | Farm | protected |
| m\_wwheatptreatment | Farm | protected |
| m\_youngforest | Farm | protected |
| MakeStockFarmer(void) | ConventionalPlant | inlinevirtual |
| Management(void) | Farm | virtual |
| Molluscicide(LE \*a\_field, double a\_user, int a\_days) | Farm | virtual |
| PigsAreOut(LE \*a\_field, double a\_user, int a\_days) | Farm | virtual |
| PigsAreOutForced(LE \*a\_field, double a\_user, int a\_days) | Farm | virtual |
| PigsOut(LE \*a\_field, double a\_user, int a\_days) | Farm | virtual |
| ProductApplication0(LE \*a\_field, double a\_user, int a\_days) | Farm | virtual |
| ProductApplication1(LE \*a\_field, double a\_user, int a\_days) | Farm | virtual |
| ReadRotation(std::string fname) | Farm | protected |
| RemoveField(LE \*a\_field) | Farm |  |
| RowCultivation(LE \*a\_field, double a\_user, int a\_days) | Farm | virtual |
| SetFarmNumber(int a\_farm\_num) | Farm | inline |
| SleepAllDay(LE \*a\_field, double a\_user, int a\_days) | Farm | virtual |
| SpringHarrow(LE \*a\_field, double a\_user, int a\_days) | Farm | virtual |
| SpringPlough(LE \*a\_field, double a\_user, int a\_days) | Farm | virtual |
| SpringRoll(LE \*a\_field, double a\_user, int a\_days) | Farm | virtual |
| SpringSow(LE \*a\_field, double a\_user, int a\_days) | Farm | virtual |
| StrawChopping(LE \*a\_field, double a\_user, int a\_days) | Farm | virtual |
| Strigling(LE \*a\_field, double a\_user, int a\_days) | Farm | virtual |
| StriglingSow(LE \*a\_field, double a\_user, int a\_days) | Farm | virtual |
| StubbleHarrowing(LE \*a\_field, double a\_user, int a\_days) | Farm | virtual |
| Swathing(LE \*a\_field, double a\_user, int a\_days) | Farm | virtual |
| SynInsecticideTreat(LE \*a\_field, double a\_user, int a\_days) | Farm | virtual |
| TranslateCropCodes(std::string &str) | Farm |  |
| Trial\_Control(LE \*a\_field, double a\_user, int a\_days) | Farm | virtual |
| Trial\_PesticideTreat(LE \*a\_field, double a\_user, int a\_days) | Farm | virtual |
| Trial\_PesticideTreat\_GS(LE \*a\_field, double a\_user, int a\_days) | Farm | virtual |
| Trial\_ToxicControl(LE \*a\_field, double a\_user, int a\_days) | Farm | virtual |
| Water(LE \*a\_field, double a\_user, int a\_days) | Farm | virtual |
| WinterPlough(LE \*a\_field, double a\_user, int a\_days) | Farm | virtual |
| ~Farm(void) | Farm | virtual |


- Generated on Thu Jan 10 2013 13:15:36 for ALMaSS Skylark ODdox by
   1.8.1.1
