# Supplementary material for: Modelling Skylarks (Alauda arvensis) to Predict Impacts of Changes in Land Management and Policy: Development and Testing of an Agent-Based Model
Source: PLoS One. 2013 Jun 6;8(6):e65803. doi: 10.1371/journal.pone.0065803 (PMC3675089; doi:10.1371/journal.pone.0065803)
Supplement: Supporting Information S4 — The skylark ODdox as a zipped archive. (ZIP) [file pone.0065803.s004.zip › Skylark_ODdox/class_crop.html]

ALMaSS Skylark ODdox: Crop Class Reference


|  |
| --- |
| ALMaSS Skylark ODdox  2.0 |


- Main Page
- Related Pages
- Classes
- Files

- Class List
- Class Index
- Class Hierarchy
- Class Members

Public Member Functions |
Protected Member Functions |
Protected Attributes

Crop Class Reference

The base class for all crops.
More...

`#include <farm.h>`

List of all members.

|  |  |
| --- | --- |
| Public Member Functions | |
|  | Crop () |
| virtual bool | Do (Farm \*a\_farm, LE \*a\_field, FarmEvent \*a\_ev) |
| int | GetFirstDate (void) |
| virtual | ~Crop () |

|  |  |
| --- | --- |
| Protected Member Functions | |
| void | SimpleEvent (long a\_date, int a\_todo, bool a\_lock) |
|  | Adds an event to this crop management. |

|  |  |
| --- | --- |
| Protected Attributes | |
| int | m\_count |
| FarmEvent \* | m\_ev |
| Farm \* | m\_farm |
| LE \* | m\_field |
| int | m\_first\_date |
| int | m\_last\_date |

---

## Detailed Description

The base class for all crops.

---

## Constructor & Destructor Documentation

|  |  |  |  |  |  |  |
| --- | --- | --- | --- | --- | --- | --- |
| |  |  |  |  |  | | --- | --- | --- | --- | --- | | virtual Crop::~Crop | ( |  | ) |  | | inlinevirtual |

{}

|  |  |  |  |  |
| --- | --- | --- | --- | --- |
| Crop::Crop | ( |  | ) |  |

{

;

}

---

## Member Function Documentation

|  |  |  |  |  |  |  |  |  |  |  |  |  |  |  |  |  |  |
| --- | --- | --- | --- | --- | --- | --- | --- | --- | --- | --- | --- | --- | --- | --- | --- | --- | --- |
| |  |  |  |  | | --- | --- | --- | --- | | bool Crop::Do | ( | Farm \* | *a\_farm*, | |  |  | LE \* | *a\_field*, | |  |  | FarmEvent \* | *a\_ev* | |  | ) |  |  | | virtual |

{

return true;

}

|  |  |  |  |  |  |  |  |
| --- | --- | --- | --- | --- | --- | --- | --- |
| |  |  |  |  |  |  | | --- | --- | --- | --- | --- | --- | | int Crop::GetFirstDate | ( | void |  | ) |  | | inline |

References m\_first\_date.

{ return m\_first\_date; }

|  |  |  |  |  |  |  |  |  |  |  |  |  |  |  |  |  |  |
| --- | --- | --- | --- | --- | --- | --- | --- | --- | --- | --- | --- | --- | --- | --- | --- | --- | --- |
| |  |  |  |  | | --- | --- | --- | --- | | void Crop::SimpleEvent | ( | long | *a\_date*, | |  |  | int | *a\_todo*, | |  |  | bool | *a\_lock* | |  | ) |  |  | | protected |

Adds an event to this crop management.

{

m\_farm->AddNewEvent( m\_field->GetVegType(), a\_date, m\_field, a\_todo, m\_field->GetRunNum(), a\_lock, 0, false, (TTypesOfVegetation) 0 );

}

---

## Member Data Documentation

|  |  |  |
| --- | --- | --- |
| |  | | --- | | int Crop::m\_count | | protected |

|  |  |  |
| --- | --- | --- |
| |  | | --- | | FarmEvent\* Crop::m\_ev | | protected |

|  |  |  |
| --- | --- | --- |
| |  | | --- | | Farm\* Crop::m\_farm | | protected |

|  |  |  |
| --- | --- | --- |
| |  | | --- | | LE\* Crop::m\_field | | protected |

|  |  |  |
| --- | --- | --- |
| |  | | --- | | int Crop::m\_first\_date | | protected |

Referenced by GetFirstDate().

|  |  |  |
| --- | --- | --- |
| |  | | --- | | int Crop::m\_last\_date | | protected |

---

The documentation for this class was generated from the following files:

- farm.h
- farm.cpp


- Crop
- Generated on Thu Jan 10 2013 13:15:36 for ALMaSS Skylark ODdox by
   1.8.1.1
