# Supplementary material for: Modelling Skylarks (Alauda arvensis) to Predict Impacts of Changes in Land Management and Policy: Development and Testing of an Agent-Based Model
Source: PLoS One. 2013 Jun 6;8(6):e65803. doi: 10.1371/journal.pone.0065803 (PMC3675089; doi:10.1371/journal.pone.0065803)
Supplement: Supporting Information S4 — The skylark ODdox as a zipped archive. (ZIP) [file pone.0065803.s004.zip › Skylark_ODdox/class_crop_data.html]

ALMaSS Skylark ODdox: CropData Class Reference


|  |
| --- |
| ALMaSS Skylark ODdox  2.0 |


- Main Page
- Related Pages
- Classes
- Files

- Class List
- Class Index
- Class Hierarchy
- Class Members

Public Member Functions |
Private Member Functions |
Private Attributes

CropData Class Reference

`#include <plants.h>`

List of all members.

|  |  |
| --- | --- |
| Public Member Functions | |
|  | CropData (const char \*a\_cropcurvefile) |
| double | GetBugPercentA (TTypesOfVegetation a\_letype) |
| double | GetBugPercentB (TTypesOfVegetation a\_letype) |
| double | GetBugPercentC (TTypesOfVegetation a\_letype) |
| double | GetBugPercentD (TTypesOfVegetation a\_letype) |
| double | GetHeightDiff (double a\_ddegs, double a\_yddegs, int a\_plant, int a\_phase) |
| double | GetLAgreenDiff (double a\_ddegs, double a\_yddegs, int a\_plant, int a\_phase) |
| double | GetLAtotalDiff (double a\_ddegs, double a\_yddegs, int a\_plant, int a\_phase) |
| int | GetNumCrops () |
| bool | GetNutStatus (int a\_plant\_num) |
| bool | GetNutStatusExt (int a\_plant) |
| double | GetStartValue (int a\_veg\_type, int a\_phase, int a\_type) |
| double | GetWeedPercent (TTypesOfVegetation a\_letype) |
| bool | StartValid (int a\_veg\_type, int a\_phase) |
| int | VegTypeToCurveNum (TTypesOfVegetation VegReference) |
|  | ~CropData () |

|  |  |
| --- | --- |
| Private Member Functions | |
| unsigned int | FindCropNum (const char \*a\_cropcurvefile) |
| double | FindDiff (double a\_ddegs, double a\_yddegs, int a\_plant, int a\_phase, int a\_type) |
| void | MakeBugPercentArray (void) |
| void | ReadBugPercentageFile (void) |
| void | SetVegNum (unsigned int a\_i, const char \*a\_cropcurvefile) |

|  |  |
| --- | --- |
| Private Attributes | |
| double \* | m\_bug\_percent\_a |
| double \* | m\_bug\_percent\_b |
| double \* | m\_bug\_percent\_c |
| int \* | m\_bug\_percent\_d |
| vector< CropGrowth \* > | m\_growth |
| FILE \* | m\_ifile |
| int | m\_num\_crops |
| vector< int > | m\_numbers |
| double \* | m\_weed\_percent |

---

## Constructor & Destructor Documentation

|  |  |  |  |  |  |
| --- | --- | --- | --- | --- | --- |
| CropData::CropData | ( | const char \* | *a\_cropcurvefile* | ) |  |

References FindCropNum(), FloatToDouble(), m\_growth, m\_ifile, MaxNoInflections, and SetVegNum().

{

// Just finds out how many veg curves there are.

unsigned int NoPlants = FindCropNum( a\_vegcurvefile );

for ( unsigned int i=0; i<NoPlants; i++) {

CropGrowth\* temp;

temp = new CropGrowth;

m\_growth[ i ] = temp;

SetVegNum( i, a\_vegcurvefile );

for (unsigned int j=0; j<5; j++) { // for each growth phase

// 'Local' index into crop growth curves.

int lk = 0;

for (unsigned int k=0; k<MaxNoInflections; k++) {

// for each inflection point

int entry;

fscanf( m\_ifile, "%d", &entry );

float f1=0,f2=0,f0=0;

if ( entry == -1 ) {

// Crop start data.

m\_growth[ i ]->m\_start\_valid[j] = true;

fscanf( m\_ifile, "%g %g %g",&f1,&f0,&f2);

FloatToDouble(m\_growth[ i ]->m\_start[j][1],f1);

FloatToDouble(m\_growth[ i ]->m\_start[j][0],f0);

FloatToDouble(m\_growth[ i ]->m\_start[j][2],f2);

} else {

// Add inflection point to normal growth curves.

m\_growth[ i ]->m\_dds[j][lk] = (double)entry;

fscanf( m\_ifile, "%g %g %g",&f1,&f0,&f2);

FloatToDouble(m\_growth[ i ]->m\_slopes[j][1][lk],f1);

FloatToDouble(m\_growth[ i ]->m\_slopes[j][0][lk],f0);

FloatToDouble(m\_growth[ i ]->m\_slopes[j][2][lk],f2);

lk++;

}

} // MaxNoInflections

} // Growth Phases

} // NoPlants

fclose( m\_ifile );

}

|  |  |  |  |  |
| --- | --- | --- | --- | --- |
| CropData::~CropData | ( |  | ) |  |

References m\_growth.

{

for ( unsigned int i=0; i<m\_growth.size(); i++ )

delete m\_growth[i];

}

---

## Member Function Documentation

|  |  |  |  |  |  |  |  |
| --- | --- | --- | --- | --- | --- | --- | --- |
| |  |  |  |  |  |  | | --- | --- | --- | --- | --- | --- | | unsigned int CropData::FindCropNum | ( | const char \* | *a\_cropcurvefile* | ) |  | | private |

References g\_msg, m\_growth, m\_ifile, m\_num\_crops, m\_numbers, MapErrorMsg::Warn(), and WARN\_FILE.

Referenced by CropData().

{

int NoPlants;

m\_numbers.resize(201);

for ( unsigned int i=0; i<201; i++) {

m\_numbers[ i ] = -1;

}

m\_ifile = fopen(a\_cropcurvefile, "r" );

if (!m\_ifile){

g\_msg->Warn(WARN\_FILE, "CropData::CropData: Unable to open file",

a\_cropcurvefile );

exit(1);

}

fscanf( m\_ifile , "%d", &NoPlants ); // How many tables to read in

m\_growth.resize( NoPlants );

m\_num\_crops = NoPlants;

return NoPlants;

}

|  |  |  |  |  |  |  |  |  |  |  |  |  |  |  |  |  |  |  |  |  |  |  |  |  |  |
| --- | --- | --- | --- | --- | --- | --- | --- | --- | --- | --- | --- | --- | --- | --- | --- | --- | --- | --- | --- | --- | --- | --- | --- | --- | --- |
| |  |  |  |  | | --- | --- | --- | --- | | double CropData::FindDiff | ( | double | *a\_ddegs*, | |  |  | double | *a\_yddegs*, | |  |  | int | *a\_plant*, | |  |  | int | *a\_phase*, | |  |  | int | *a\_type* | |  | ) |  |  | | private |

References m\_growth, m\_numbers, and MaxNoInflections.

Referenced by GetHeightDiff(), GetLAgreenDiff(), and GetLAtotalDiff().

{

// Check for valid plant number at runtime?

// This is broken for growth curves where one can risk passing

// more than a single inflection point in the growth curve in a

// single day...

int index = m\_numbers[ a\_plant ];

unsigned int oldindex=0, newindex=0;

if ( m\_growth[ index ]->m\_dds[ a\_phase ][ 0 ] == 99999 ) {

return 0.0;

}

for ( unsigned int i=0; i<MaxNoInflections; i++ ) {

// In other words: If the current value for summed day degrees

// is smaller than the X position of the \*next\* inflection

// point, then we are in the correct interval.

if ( m\_growth[ index ]->m\_dds[ a\_phase ][ i+1 ] > a\_ddegs ) {

newindex = i;

break;

// return m\_growth[ index ]->m\_slopes[ a\_phase ][ a\_type ][i];

}

}

for ( unsigned int i=0; i<MaxNoInflections; i++ ) {

if ( m\_growth[ index ]->m\_dds[ a\_phase ][ i+1 ] > a\_yddegs ) {

oldindex = i;

break;

// return m\_growth[ index ]->m\_slopes[ a\_phase ][ a\_type ][i];

}

}

double diff;

if ( newindex > oldindex ) {

// We have passed an inflection point between today and yesterday.

// First add the increment from yesterdays day degree sum up to

// the inflection point.

double dddif =

m\_growth[ index ]->m\_dds[ a\_phase ][ newindex ] - a\_yddegs;

diff =

m\_growth[ index ]->m\_slopes[ a\_phase ][ a\_type ][oldindex]\*

dddif;

// Then from the inflection point up to today.

dddif = a\_ddegs -

m\_growth[ index ]->m\_dds[ a\_phase ][ newindex ];

diff +=

m\_growth[ index ]->m\_slopes[ a\_phase ][ a\_type ][ newindex ]\*

dddif;

} else {

// No inflection point passed.

diff = m\_growth[ index ]->m\_slopes[ a\_phase ][ a\_type ][ newindex ] \*

(a\_ddegs - a\_yddegs);

}

return diff;

}

|  |  |  |  |  |  |  |  |
| --- | --- | --- | --- | --- | --- | --- | --- |
| |  |  |  |  |  |  | | --- | --- | --- | --- | --- | --- | | double CropData::GetBugPercentA | ( | TTypesOfVegetation | *a\_letype* | ) |  | | inline |

{

return m\_bug\_percent\_a[ a\_letype ];

}

|  |  |  |  |  |  |  |  |
| --- | --- | --- | --- | --- | --- | --- | --- |
| |  |  |  |  |  |  | | --- | --- | --- | --- | --- | --- | | double CropData::GetBugPercentB | ( | TTypesOfVegetation | *a\_letype* | ) |  | | inline |

{

return m\_bug\_percent\_b[ a\_letype ];

}

|  |  |  |  |  |  |  |  |
| --- | --- | --- | --- | --- | --- | --- | --- |
| |  |  |  |  |  |  | | --- | --- | --- | --- | --- | --- | | double CropData::GetBugPercentC | ( | TTypesOfVegetation | *a\_letype* | ) |  | | inline |

{

return m\_bug\_percent\_c[ a\_letype ];

}

|  |  |  |  |  |  |  |  |
| --- | --- | --- | --- | --- | --- | --- | --- |
| |  |  |  |  |  |  | | --- | --- | --- | --- | --- | --- | | double CropData::GetBugPercentD | ( | TTypesOfVegetation | *a\_letype* | ) |  | | inline |

{

return (double) m\_bug\_percent\_d[ a\_letype ];

}

|  |  |  |  |
| --- | --- | --- | --- |
| double CropData::GetHeightDiff | ( | double | *a\_ddegs*, |
|  |  | double | *a\_yddegs*, |
|  |  | int | *a\_plant*, |
|  |  | int | *a\_phase* |
|  | ) |  |  |

References FindDiff().

Referenced by VegElement::DoDevelopment(), and VegElement::ForceGrowthSpringTest().

{

return FindDiff( a\_ddegs , a\_yddegs, a\_plant, a\_phase, 2 );

}

|  |  |  |  |
| --- | --- | --- | --- |
| double CropData::GetLAgreenDiff | ( | double | *a\_ddegs*, |
|  |  | double | *a\_yddegs*, |
|  |  | int | *a\_plant*, |
|  |  | int | *a\_phase* |
|  | ) |  |  |

References FindDiff().

Referenced by VegElement::DoDevelopment(), and VegElement::ForceGrowthSpringTest().

{

return FindDiff( a\_ddegs, a\_yddegs, a\_plant, a\_phase, 0 );

}

|  |  |  |  |
| --- | --- | --- | --- |
| double CropData::GetLAtotalDiff | ( | double | *a\_ddegs*, |
|  |  | double | *a\_yddegs*, |
|  |  | int | *a\_plant*, |
|  |  | int | *a\_phase* |
|  | ) |  |  |

References FindDiff().

Referenced by VegElement::DoDevelopment(), UnsprayedFieldMargin::DoDevelopment(), and VegElement::ForceGrowthSpringTest().

{

return FindDiff( a\_ddegs, a\_yddegs, a\_plant, a\_phase, 1 );

}

|  |  |  |  |  |  |  |
| --- | --- | --- | --- | --- | --- | --- |
| |  |  |  |  |  | | --- | --- | --- | --- | --- | | int CropData::GetNumCrops | ( |  | ) |  | | inline |

Referenced by CropRotation::GetFirstCrop().

{

return m\_num\_crops;

}

|  |  |  |  |  |  |  |  |
| --- | --- | --- | --- | --- | --- | --- | --- |
| |  |  |  |  |  |  | | --- | --- | --- | --- | --- | --- | | bool CropData::GetNutStatus | ( | int | *a\_plant\_num* | ) |  | | inline |

Referenced by CropRotation::GetFirstCrop().

{

return m\_growth[ a\_plant\_num ]->m\_lownut;

}

|  |  |  |  |  |  |  |  |
| --- | --- | --- | --- | --- | --- | --- | --- |
| |  |  |  |  |  |  | | --- | --- | --- | --- | --- | --- | | bool CropData::GetNutStatusExt | ( | int | *a\_plant* | ) |  | | inline |

{

return m\_growth[ m\_numbers[ a\_plant ]]->m\_lownut;

}

|  |  |  |  |  |  |  |  |  |  |  |  |  |  |  |  |  |  |
| --- | --- | --- | --- | --- | --- | --- | --- | --- | --- | --- | --- | --- | --- | --- | --- | --- | --- |
| |  |  |  |  | | --- | --- | --- | --- | | double CropData::GetStartValue | ( | int | *a\_veg\_type*, | |  |  | int | *a\_phase*, | |  |  | int | *a\_type* | |  | ) |  |  | | inline |

Referenced by VegElement::ForceGrowthInitialize(), and VegElement::SetGrowthPhase().

{

return m\_growth[ m\_numbers[ a\_veg\_type ]]->m\_start[ a\_phase ] [ a\_type ];

}

|  |  |  |  |  |  |  |  |
| --- | --- | --- | --- | --- | --- | --- | --- |
| |  |  |  |  |  |  | | --- | --- | --- | --- | --- | --- | | double CropData::GetWeedPercent | ( | TTypesOfVegetation | *a\_letype* | ) |  | | inline |

{

return m\_weed\_percent[ a\_letype ];

}

|  |  |  |  |  |  |  |  |
| --- | --- | --- | --- | --- | --- | --- | --- |
| |  |  |  |  |  |  | | --- | --- | --- | --- | --- | --- | | void CropData::MakeBugPercentArray | ( | void |  | ) |  | | private |

|  |  |  |  |  |  |  |  |
| --- | --- | --- | --- | --- | --- | --- | --- |
| |  |  |  |  |  |  | | --- | --- | --- | --- | --- | --- | | void CropData::ReadBugPercentageFile | ( | void |  | ) |  | | private |

|  |  |  |  |  |  |  |  |  |  |  |  |  |  |
| --- | --- | --- | --- | --- | --- | --- | --- | --- | --- | --- | --- | --- | --- |
| |  |  |  |  | | --- | --- | --- | --- | | void CropData::SetVegNum | ( | unsigned int | *a\_i*, | |  |  | const char \* | *a\_cropcurvefile* | |  | ) |  |  | | private |

References g\_msg, m\_growth, m\_ifile, m\_numbers, MapErrorMsg::Warn(), and WARN\_FILE.

Referenced by CropData().

{

int ThisPlant;

// Find out what crop and what nutrient status

fscanf( m\_ifile, "%d", &ThisPlant);

// Check if valid plant number (from the file).

if ( ThisPlant < 0 || ThisPlant > 200 ) {

g\_msg->Warn(WARN\_FILE, "CropData::FindCropNum(): Illegal plant number"

" specified in", a\_cropcurvefile );

exit(1);

}

m\_numbers[ ThisPlant ] = a\_i;

// if greater than 100 then it is low nutrient

if ( ThisPlant > 100 ) {

m\_growth[ a\_i ]->m\_lownut = true;

} else {

m\_growth[ a\_i ]->m\_lownut = false;

}

}

|  |  |  |  |
| --- | --- | --- | --- |
| bool CropData::StartValid | ( | int | *a\_veg\_type*, |
|  |  | int | *a\_phase* |
|  | ) |  |  |

References m\_growth, and m\_numbers.

Referenced by VegElement::ForceGrowthInitialize(), and VegElement::SetGrowthPhase().

{

int a=m\_numbers[ a\_veg\_type ];

CropGrowth\* p=m\_growth[a];

return p-> m\_start\_valid[ a\_phase ];

}

|  |  |  |  |  |  |
| --- | --- | --- | --- | --- | --- |
| int CropData::VegTypeToCurveNum | ( | TTypesOfVegetation | *VegReference* | ) |  |

References g\_msg, tov\_AgroChemIndustryCereal, tov\_Carrots, tov\_CloverGrassGrazed1, tov\_CloverGrassGrazed2, tov\_FieldPeas, tov\_FieldPeasStrigling, tov\_FodderBeet, tov\_FodderGrass, tov\_Lawn, tov\_Maize, tov\_MaizeSilage, tov\_MaizeStrigling, tov\_NaturalGrass, tov\_NoGrowth, tov\_Oats, tov\_OBarleyPeaCloverGrass, tov\_OCarrots, tov\_OCloverGrassGrazed1, tov\_OCloverGrassGrazed2, tov\_OCloverGrassSilage1, tov\_OFieldPeas, tov\_OFieldPeasSilage, tov\_OGrazingPigs, tov\_OMaizeSilage, tov\_OOats, tov\_OPermanentGrassGrazed, tov\_OPotatoes, tov\_OSBarleySilage, tov\_OSetaside, tov\_OSpringBarley, tov\_OSpringBarleyClover, tov\_OSpringBarleyExt, tov\_OSpringBarleyGrass, tov\_OSpringBarleyPigs, tov\_OWinterBarley, tov\_OWinterBarleyExt, tov\_OWinterRape, tov\_OWinterRye, tov\_OWinterWheatUndersown, tov\_PermanentGrassGrazed, tov\_PermanentGrassLowYield, tov\_PermanentGrassTussocky, tov\_PermanentSetaside, tov\_Potatoes, tov\_PotatoesIndustry, tov\_SeedGrass1, tov\_SeedGrass2, tov\_Setaside, tov\_SpringBarley, tov\_SpringBarleyCloverGrass, tov\_SpringBarleyCloverGrassStrigling, tov\_SpringBarleyPeaCloverGrassStrigling, tov\_SpringBarleyPTreatment, tov\_SpringBarleySeed, tov\_SpringBarleySilage, tov\_SpringBarleySKManagement, tov\_SpringBarleyStrigling, tov\_SpringBarleyStriglingCulm, tov\_SpringBarleyStriglingSingle, tov\_SpringRape, tov\_Triticale, tov\_WinterBarley, tov\_WinterBarleyStrigling, tov\_WinterRape, tov\_WinterRapeStrigling, tov\_WinterRye, tov\_WinterRyeStrigling, tov\_WinterWheat, tov\_WinterWheatShort, tov\_WinterWheatStrigling, tov\_WinterWheatStriglingCulm, tov\_WinterWheatStriglingSingle, tov\_WWheatPControl, tov\_WWheatPToxicControl, tov\_WWheatPTreatment, tov\_YoungForest, MapErrorMsg::Warn(), and WARN\_FILE.

Referenced by BeetleBank::BeetleBank(), FieldBoundary::FieldBoundary(), Heath::Heath(), HedgeBank::HedgeBank(), Hedges::Hedges(), Marsh::Marsh(), NaturalGrass::NaturalGrass(), Orchard::Orchard(), OrchardBand::OrchardBand(), OrchardGrass::OrchardGrass(), PermanentSetaside::PermanentSetaside(), VegElement::SetVegType(), UnsprayedFieldMargin::UnsprayedFieldMargin(), VegElement::VegElement(), and YoungForest::YoungForest().

{

char error\_num[20];

switch (VegReference)

{

case tov\_OSpringBarleyPigs:

case tov\_OSpringBarley:

case tov\_OSpringBarleyExt:

return 101;

case tov\_SpringBarleySilage:

case tov\_SpringBarley:

case tov\_SpringBarleyPTreatment:

case tov\_SpringBarleySKManagement:

case tov\_SpringBarleyStrigling:

case tov\_SpringBarleyStriglingSingle:

case tov\_SpringBarleyStriglingCulm:

return 1;

case tov\_WinterBarley:

case tov\_WinterBarleyStrigling:

return 2;

case tov\_OWinterBarley:

case tov\_OWinterBarleyExt:

return 102;

case tov\_WinterWheat:

case tov\_WinterWheatShort:

case tov\_WinterWheatStrigling:

case tov\_WinterWheatStriglingCulm:

case tov\_WinterWheatStriglingSingle:

case tov\_AgroChemIndustryCereal:

case tov\_WWheatPControl:

case tov\_WWheatPToxicControl:

case tov\_WWheatPTreatment:

return 4;

case tov\_OWinterWheatUndersown:

return 104;

case tov\_WinterRye:

case tov\_WinterRyeStrigling:

return 5;

case tov\_OWinterRye:

return 105;

case tov\_Oats:

return 6;

case tov\_OOats:

return 106;

case tov\_Maize:

case tov\_MaizeSilage:

case tov\_MaizeStrigling:

return 8;

case tov\_OMaizeSilage:

return 108;

case tov\_SpringBarleyCloverGrass:

case tov\_SpringBarleyCloverGrassStrigling:

case tov\_SpringBarleySeed:

case tov\_SpringBarleyPeaCloverGrassStrigling:

return 13;

case tov\_OBarleyPeaCloverGrass:

case tov\_OSBarleySilage:

case tov\_OSpringBarleyGrass:

case tov\_OSpringBarleyClover:

return 113;

case tov\_WinterRape:

case tov\_WinterRapeStrigling:

return 22;

case tov\_OWinterRape:

return 22;

case tov\_PermanentGrassGrazed:

case tov\_OPermanentGrassGrazed:

return 26;

case tov\_SeedGrass1:

case tov\_SeedGrass2:

return 27;

case tov\_FodderGrass:

case tov\_CloverGrassGrazed1:

case tov\_CloverGrassGrazed2:

case tov\_OCloverGrassGrazed1:

case tov\_OCloverGrassGrazed2:

case tov\_OCloverGrassSilage1:

case tov\_OGrazingPigs:

return 29;

case tov\_OFieldPeas:

case tov\_OFieldPeasSilage:

case tov\_FieldPeas:

case tov\_FieldPeasStrigling:

return 30;

case tov\_Carrots:

return 41;

case tov\_OCarrots:

return 141;

case tov\_Potatoes: return 50;

case tov\_OPotatoes: return 150;

case tov\_PotatoesIndustry: return 50;

case tov\_FodderBeet:

return 60;

case tov\_PermanentGrassLowYield:

case tov\_PermanentGrassTussocky:

return 90;

// Special growth mode for green but unused elements.

// tov\_PermanentSetaside Does not change growth phase no matter

// how hard one tries to do just that.

case tov\_PermanentSetaside: return 92;

case tov\_Setaside:

case tov\_OSetaside: return 112;

case tov\_YoungForest:

case tov\_NaturalGrass: return 90;

case tov\_NoGrowth: return 91;

case tov\_Lawn: return 94;

//case tov\_SpringWheat: return 3;

case tov\_Triticale: return 7;

case tov\_SpringRape: return 21;

default: // No matching code so we need an error message of some kind

sprintf( error\_num, "%d", VegReference );

g\_msg->Warn( WARN\_FILE,

"CropData::VegTypeToCurveNum(): Unknown vegetation type:",

error\_num );

exit( 1 );

}

}

---

## Member Data Documentation

|  |  |  |
| --- | --- | --- |
| |  | | --- | | double\* CropData::m\_bug\_percent\_a | | private |

|  |  |  |
| --- | --- | --- |
| |  | | --- | | double\* CropData::m\_bug\_percent\_b | | private |

|  |  |  |
| --- | --- | --- |
| |  | | --- | | double\* CropData::m\_bug\_percent\_c | | private |

|  |  |  |
| --- | --- | --- |
| |  | | --- | | int\* CropData::m\_bug\_percent\_d | | private |

|  |  |  |
| --- | --- | --- |
| |  | | --- | | vector< CropGrowth \* > CropData::m\_growth | | private |

Referenced by CropData(), FindCropNum(), FindDiff(), SetVegNum(), StartValid(), and ~CropData().

|  |  |  |
| --- | --- | --- |
| |  | | --- | | FILE\* CropData::m\_ifile | | private |

Referenced by CropData(), FindCropNum(), and SetVegNum().

|  |  |  |
| --- | --- | --- |
| |  | | --- | | int CropData::m\_num\_crops | | private |

Referenced by FindCropNum().

|  |  |  |
| --- | --- | --- |
| |  | | --- | | vector< int > CropData::m\_numbers | | private |

Referenced by FindCropNum(), FindDiff(), SetVegNum(), and StartValid().

|  |  |  |
| --- | --- | --- |
| |  | | --- | | double\* CropData::m\_weed\_percent | | private |

---

The documentation for this class was generated from the following files:

- plants.h
- plants.cpp


- CropData
- Generated on Thu Jan 10 2013 13:15:36 for ALMaSS Skylark ODdox by
   1.8.1.1
