# Supplementary material for: Modelling Skylarks (Alauda arvensis) to Predict Impacts of Changes in Land Management and Policy: Development and Testing of an Agent-Based Model
Source: PLoS One. 2013 Jun 6;8(6):e65803. doi: 10.1371/journal.pone.0065803 (PMC3675089; doi:10.1371/journal.pone.0065803)
Supplement: Supporting Information S4 — The skylark ODdox as a zipped archive. (ZIP) [file pone.0065803.s004.zip › Skylark_ODdox/class_crop_data-members.html]

ALMaSS Skylark ODdox: Member List


|  |
| --- |
| ALMaSS Skylark ODdox  2.0 |


- Main Page
- Related Pages
- Classes
- Files

- Class List
- Class Index
- Class Hierarchy
- Class Members

CropData Member List

This is the complete list of members for CropData, including all inherited members.

|  |  |  |
| --- | --- | --- |
| CropData(const char \*a\_cropcurvefile) | CropData |  |
| FindCropNum(const char \*a\_cropcurvefile) | CropData | private |
| FindDiff(double a\_ddegs, double a\_yddegs, int a\_plant, int a\_phase, int a\_type) | CropData | private |
| GetBugPercentA(TTypesOfVegetation a\_letype) | CropData | inline |
| GetBugPercentB(TTypesOfVegetation a\_letype) | CropData | inline |
| GetBugPercentC(TTypesOfVegetation a\_letype) | CropData | inline |
| GetBugPercentD(TTypesOfVegetation a\_letype) | CropData | inline |
| GetHeightDiff(double a\_ddegs, double a\_yddegs, int a\_plant, int a\_phase) | CropData |  |
| GetLAgreenDiff(double a\_ddegs, double a\_yddegs, int a\_plant, int a\_phase) | CropData |  |
| GetLAtotalDiff(double a\_ddegs, double a\_yddegs, int a\_plant, int a\_phase) | CropData |  |
| GetNumCrops() | CropData | inline |
| GetNutStatus(int a\_plant\_num) | CropData | inline |
| GetNutStatusExt(int a\_plant) | CropData | inline |
| GetStartValue(int a\_veg\_type, int a\_phase, int a\_type) | CropData | inline |
| GetWeedPercent(TTypesOfVegetation a\_letype) | CropData | inline |
| m\_bug\_percent\_a | CropData | private |
| m\_bug\_percent\_b | CropData | private |
| m\_bug\_percent\_c | CropData | private |
| m\_bug\_percent\_d | CropData | private |
| m\_growth | CropData | private |
| m\_ifile | CropData | private |
| m\_num\_crops | CropData | private |
| m\_numbers | CropData | private |
| m\_weed\_percent | CropData | private |
| MakeBugPercentArray(void) | CropData | private |
| ReadBugPercentageFile(void) | CropData | private |
| SetVegNum(unsigned int a\_i, const char \*a\_cropcurvefile) | CropData | private |
| StartValid(int a\_veg\_type, int a\_phase) | CropData |  |
| VegTypeToCurveNum(TTypesOfVegetation VegReference) | CropData |  |
| ~CropData() | CropData |  |


- Generated on Thu Jan 10 2013 13:15:36 for ALMaSS Skylark ODdox by
   1.8.1.1
