# Supplementary material for: Modelling Skylarks (Alauda arvensis) to Predict Impacts of Changes in Land Management and Policy: Development and Testing of an Agent-Based Model
Source: PLoS One. 2013 Jun 6;8(6):e65803. doi: 10.1371/journal.pone.0065803 (PMC3675089; doi:10.1371/journal.pone.0065803)
Supplement: Supporting Information S4 — The skylark ODdox as a zipped archive. (ZIP) [file pone.0065803.s004.zip › Skylark_ODdox/class_crop_growth.html]

ALMaSS Skylark ODdox: CropGrowth Class Reference


|  |
| --- |
| ALMaSS Skylark ODdox  2.0 |


- Main Page
- Related Pages
- Classes
- Files

- Class List
- Class Index
- Class Hierarchy
- Class Members

Public Member Functions |
Public Attributes

CropGrowth Class Reference

`#include <plants.h>`

List of all members.

|  |  |
| --- | --- |
| Public Member Functions | |
|  | CropGrowth (void) |

|  |  |
| --- | --- |
| Public Attributes | |
| double | m\_dds [5][MaxNoInflections] |
| bool | m\_lownut |
| double | m\_slopes [5][3][MaxNoInflections] |
| double | m\_start [5][3] |
| bool | m\_start\_valid [5] |

---

## Constructor & Destructor Documentation

|  |  |  |  |  |  |
| --- | --- | --- | --- | --- | --- |
| CropGrowth::CropGrowth | ( | void |  | ) |  |

References m\_start, and m\_start\_valid.

{

for (unsigned int j=0; j<5; j++) {

m\_start\_valid[j] = false;

for ( unsigned int k=0; k<3; k++) {

m\_start[j][k] = 0.0;

}

}

}

---

## Member Data Documentation

|  |
| --- |
| double CropGrowth::m\_dds[5][MaxNoInflections] |

|  |
| --- |
| bool CropGrowth::m\_lownut |

|  |
| --- |
| double CropGrowth::m\_slopes[5][3][MaxNoInflections] |

|  |
| --- |
| double CropGrowth::m\_start[5][3] |

Referenced by CropGrowth().

|  |
| --- |
| bool CropGrowth::m\_start\_valid[5] |

Referenced by CropGrowth().

---

The documentation for this class was generated from the following files:

- plants.h
- plants.cpp


- CropGrowth
- Generated on Thu Jan 10 2013 13:15:36 for ALMaSS Skylark ODdox by
   1.8.1.1
