# Supplementary material for: Modelling Skylarks (Alauda arvensis) to Predict Impacts of Changes in Land Management and Policy: Development and Testing of an Agent-Based Model
Source: PLoS One. 2013 Jun 6;8(6):e65803. doi: 10.1371/journal.pone.0065803 (PMC3675089; doi:10.1371/journal.pone.0065803)
Supplement: Supporting Information S4 — The skylark ODdox as a zipped archive. (ZIP) [file pone.0065803.s004.zip › Skylark_ODdox/class_crop_rotation.html]

ALMaSS Skylark ODdox: CropRotation Class Reference


|  |
| --- |
| ALMaSS Skylark ODdox  2.0 |


- Main Page
- Related Pages
- Classes
- Files

- Class List
- Class Index
- Class Hierarchy
- Class Members

Public Member Functions |
Private Attributes

CropRotation Class Reference

`#include <croprotation.h>`

List of all members.

|  |  |
| --- | --- |
| Public Member Functions | |
|  | CropRotation (int a\_num\_crops) |
| TTypesOfVegetation | GetFirstCrop (int a\_farmtype, bool \*a\_low\_nutrient) |
| TTypesOfVegetation | GetNextCrop (int a\_farmtype, int a\_current\_crop) |
|  | ~CropRotation (void) |

|  |  |
| --- | --- |
| Private Attributes | |
| vector< Rotation \* > | m\_rots |
| vector< Starter \* > | m\_start |

---

## Constructor & Destructor Documentation

|  |  |  |  |  |  |
| --- | --- | --- | --- | --- | --- |
| CropRotation::CropRotation | ( | int | *a\_num\_crops* | ) |  |

References g\_msg, l\_map\_rotation\_files\_prefix, m\_rots, m\_start, NoFarmTypes, CfgStr::value(), MapErrorMsg::Warn(), and WARN\_FILE.

{

FILE \*inpfile;

char filename[50];

m\_rots.resize( a\_num\_crops );

m\_start.resize( a\_num\_crops );

for (int i=0; i<a\_num\_crops; i++) {

m\_rots[ i ] = new Rotation;

m\_start[ i ] = new Starter;

}

for (int i=0; i<NoFarmTypes; i++) {

// Now works for any number of rotations.

sprintf( filename, "%sFarmType\_%d.rot",

l\_map\_rotation\_files\_prefix.value(),

i );

inpfile = fopen(filename, "r" );

if (!inpfile) {

g\_msg->Warn( WARN\_FILE, "CropRotation::CropRotation():"

" Unable to open file ", filename);

exit(1);

}

for (int j=0; j<a\_num\_crops; j++) {

fscanf( inpfile, "%d", (int\*)&(m\_rots[ j ]->CropNum[ i ]) );

for (int k=0; k<4; k++) {

fscanf( inpfile, "%d", (int\*)&m\_rots[ j ]->NewCrop[ i ][ k ] );

fscanf( inpfile, "%d", &m\_rots[ j ]->Percent[ i ][ k ] );

}

}

fclose( inpfile );

sprintf( filename, "FarmType\_%d.stt", i );

inpfile = fopen(filename, "r" );

if (!inpfile) {

g\_msg->Warn( WARN\_FILE, "CropRotation::CropRotation():"

" Unable to open file ", filename);

exit(1);

}

for (int j=0; j<a\_num\_crops; j++) {

fscanf( inpfile, "%d %d",

(int\*)&m\_start[ j ]->CropNum[ i ],

&m\_start[ j ]->Percent[ i ] );

}

fclose(inpfile);

}

}

|  |  |  |  |  |  |
| --- | --- | --- | --- | --- | --- |
| CropRotation::~CropRotation | ( | void |  | ) |  |

References m\_rots, and m\_start.

{

for (unsigned int i=0; i<m\_rots.size(); i++) {

delete m\_rots[ i ];

delete m\_start[ i ];

}

}

---

## Member Function Documentation

|  |  |  |  |
| --- | --- | --- | --- |
| TTypesOfVegetation CropRotation::GetFirstCrop | ( | int | *a\_farmtype*, |
|  |  | bool \* | *a\_low\_nutrient* |
|  | ) |  |  |

References g\_crops, g\_msg, CropData::GetNumCrops(), CropData::GetNutStatus(), m\_start, MapErrorMsg::Warn(), and WARN\_BUG.

{

int num\_crops = g\_crops->GetNumCrops();

for ( int i=0; i<num\_crops; i++ ) {

int percent = m\_start[ i ]->Percent[ a\_farmtype ];

if ( (num\_crops==i) ||

(-1 == m\_start[ i+1 ]->Percent[ a\_farmtype ]) ||

(rand()%100 < percent) ) {

\*a\_low\_nutrient = g\_crops->GetNutStatus( i );

return (TTypesOfVegetation) i;

}

}

g\_msg->Warn( WARN\_BUG, "CropRotation::GetFirstCrop():"

" Unable to recover crop type?!", "");

exit(1);

}

|  |  |  |  |
| --- | --- | --- | --- |
| TTypesOfVegetation CropRotation::GetNextCrop | ( | int | *a\_farmtype*, |
|  |  | int | *a\_current\_crop* |
|  | ) |  |  |

References g\_msg, m\_rots, MapErrorMsg::Warn(), and WARN\_BUG.

{

//int NumCrops = g\_crops->GetNumCrops();

for ( int i=0; i<4; i++ ) {

int percent = m\_rots[ a\_current\_crop ]->Percent[ a\_farmtype ][ i ];

// Lazy evaluation for experts. ;-)

if ( (3==i) ||

(-1 == m\_rots[ a\_current\_crop ]->Percent[ a\_farmtype ][ i+1 ]) ||

(rand()%100 < percent) ) {

return m\_rots[ a\_current\_crop ]->NewCrop[ a\_farmtype ][ i ];

}

}

g\_msg->Warn( WARN\_BUG, "CropRotation::GetNextCrop():"

" Unable to recover crop type?!", "");

exit(1);

}

---

## Member Data Documentation

|  |  |  |
| --- | --- | --- |
| |  | | --- | | vector< Rotation\* > CropRotation::m\_rots | | private |

Referenced by CropRotation(), GetNextCrop(), and ~CropRotation().

|  |  |  |
| --- | --- | --- |
| |  | | --- | | vector< Starter\* > CropRotation::m\_start | | private |

Referenced by CropRotation(), GetFirstCrop(), and ~CropRotation().

---

The documentation for this class was generated from the following files:

- croprotation.h
- croprotation.cpp


- CropRotation
- Generated on Thu Jan 10 2013 13:15:36 for ALMaSS Skylark ODdox by
   1.8.1.1
