# Supplementary material for: Modelling Skylarks (Alauda arvensis) to Predict Impacts of Changes in Land Management and Policy: Development and Testing of an Agent-Based Model
Source: PLoS One. 2013 Jun 6;8(6):e65803. doi: 10.1371/journal.pone.0065803 (PMC3675089; doi:10.1371/journal.pone.0065803)
Supplement: Supporting Information S4 — The skylark ODdox as a zipped archive. (ZIP) [file pone.0065803.s004.zip › Skylark_ODdox/class_crop-members.html]

ALMaSS Skylark ODdox: Member List


|  |
| --- |
| ALMaSS Skylark ODdox  2.0 |


- Main Page
- Related Pages
- Classes
- Files

- Class List
- Class Index
- Class Hierarchy
- Class Members

Crop Member List

This is the complete list of members for Crop, including all inherited members.

|  |  |  |
| --- | --- | --- |
| Crop() | Crop |  |
| Do(Farm \*a\_farm, LE \*a\_field, FarmEvent \*a\_ev) | Crop | virtual |
| GetFirstDate(void) | Crop | inline |
| m\_count | Crop | protected |
| m\_ev | Crop | protected |
| m\_farm | Crop | protected |
| m\_field | Crop | protected |
| m\_first\_date | Crop | protected |
| m\_last\_date | Crop | protected |
| SimpleEvent(long a\_date, int a\_todo, bool a\_lock) | Crop | protected |
| ~Crop() | Crop | inlinevirtual |


- Generated on Thu Jan 10 2013 13:15:36 for ALMaSS Skylark ODdox by
   1.8.1.1
