# Supplementary material for: Modelling Skylarks (Alauda arvensis) to Predict Impacts of Changes in Land Management and Policy: Development and Testing of an Agent-Based Model
Source: PLoS One. 2013 Jun 6;8(6):e65803. doi: 10.1371/journal.pone.0065803 (PMC3675089; doi:10.1371/journal.pone.0065803)
Supplement: Supporting Information S4 — The skylark ODdox as a zipped archive. (ZIP) [file pone.0065803.s004.zip › Skylark_ODdox/class_edge_growing_points.html]

ALMaSS Skylark ODdox: EdgeGrowingPoints Class Reference


|  |
| --- |
| ALMaSS Skylark ODdox  2.0 |


- Main Page
- Related Pages
- Classes
- Files

- Class List
- Class Index
- Class Hierarchy
- Class Members

Public Member Functions |
Private Attributes

EdgeGrowingPoints Class Reference

`#include <CIPELandscapeMaker.h>`

List of all members.

|  |  |
| --- | --- |
| Public Member Functions | |
|  | EdgeGrowingPoints (EGP\_Data egpd) |
| bool | GetCanGrow (int x, int y) |
| void | SetCanGrow (int x, int y, bool cg) |
| unsigned int | ShrinkList () |

|  |  |
| --- | --- |
| Private Attributes | |
| int | m\_centre\_x |
| int | m\_centre\_y |
| int | m\_originalsize |
| vector< Edges > | m\_OurEdges |
| int | m\_polynum |

---

## Constructor & Destructor Documentation

|  |  |  |  |  |  |
| --- | --- | --- | --- | --- | --- |
| EdgeGrowingPoints::EdgeGrowingPoints | ( | EGP\_Data | *egpd* | ) |  |

---

## Member Function Documentation

|  |  |  |  |
| --- | --- | --- | --- |
| bool EdgeGrowingPoints::GetCanGrow | ( | int | *x*, |
|  |  | int | *y* |
|  | ) |  |  |

|  |  |  |  |
| --- | --- | --- | --- |
| void EdgeGrowingPoints::SetCanGrow | ( | int | *x*, |
|  |  | int | *y*, |
|  |  | bool | *cg* |
|  | ) |  |  |

|  |  |  |  |  |
| --- | --- | --- | --- | --- |
| unsigned int EdgeGrowingPoints::ShrinkList | ( |  | ) |  |

---

## Member Data Documentation

|  |  |  |
| --- | --- | --- |
| |  | | --- | | int EdgeGrowingPoints::m\_centre\_x | | private |

|  |  |  |
| --- | --- | --- |
| |  | | --- | | int EdgeGrowingPoints::m\_centre\_y | | private |

|  |  |  |
| --- | --- | --- |
| |  | | --- | | int EdgeGrowingPoints::m\_originalsize | | private |

|  |  |  |
| --- | --- | --- |
| |  | | --- | | vector<Edges> EdgeGrowingPoints::m\_OurEdges | | private |

|  |  |  |
| --- | --- | --- |
| |  | | --- | | int EdgeGrowingPoints::m\_polynum | | private |

---

The documentation for this class was generated from the following file:

- CIPELandscapeMaker.h


- EdgeGrowingPoints
- Generated on Thu Jan 10 2013 13:15:36 for ALMaSS Skylark ODdox by
   1.8.1.1
