# Supplementary material for: Modelling Skylarks (Alauda arvensis) to Predict Impacts of Changes in Land Management and Policy: Development and Testing of an Agent-Based Model
Source: PLoS One. 2013 Jun 6;8(6):e65803. doi: 10.1371/journal.pone.0065803 (PMC3675089; doi:10.1371/journal.pone.0065803)
Supplement: Supporting Information S4 — The skylark ODdox as a zipped archive. (ZIP) [file pone.0065803.s004.zip › Skylark_ODdox/class_farm.html]

ALMaSS Skylark ODdox: Farm Class Reference


|  |
| --- |
| ALMaSS Skylark ODdox  2.0 |


- Main Page
- Related Pages
- Classes
- Files

- Class List
- Class Index
- Class Hierarchy
- Class Members

Public Member Functions |
Protected Member Functions |
Protected Attributes

Farm Class Reference

The base class for all farm types.
More...

`#include <farm.h>`

List of all members.

|  |  |
| --- | --- |
| Public Member Functions | |
| void | AddField (LE \*a\_newfield) |
|  | Adds a field to a farm. |
| void | AddNewEvent (TTypesOfVegetation a\_event, long a\_date, LE \*a\_field, int a\_todo, long a\_num, bool a\_lock, int a\_start, bool a\_first\_year, TTypesOfVegetation a\_crop) |
|  | Adds an event to the event queue for a farm. |
| virtual bool | AutumnHarrow (LE \*a\_field, double a\_user, int a\_days) |
|  | Carry out a harrow event in the autumn on a\_field. |
| virtual bool | AutumnPlough (LE \*a\_field, double a\_user, int a\_days) |
|  | Carry out a ploughing event in the autumn on a\_field. |
| virtual bool | AutumnRoll (LE \*a\_field, double a\_user, int a\_days) |
|  | Carry out a roll event in the autumn on a\_field. |
| virtual bool | AutumnSow (LE \*a\_field, double a\_user, int a\_days) |
|  | Carry out a sowing event in the autumn on a\_field. |
| virtual bool | BurnStrawStubble (LE \*a\_field, double a\_user, int a\_days) |
|  | Burn stubble on a\_field. |
| virtual bool | CattleIsOut (LE \*a\_field, double a\_user, int a\_days, int a\_max) |
|  | Generate a 'cattle\_out' event for every day the cattle are on a\_field. |
| virtual bool | CattleIsOutLow (LE \*a\_field, double a\_user, int a\_days, int a\_max) |
|  | Generate a 'cattle\_out\_low' event for every day the cattle are on a\_field. |
| virtual bool | CattleOut (LE \*a\_field, double a\_user, int a\_days) |
|  | Start a grazing event on a\_field today. |
| virtual bool | CattleOutLowGrazing (LE \*a\_field, double a\_user, int a\_days) |
|  | Start a extensive grazing event on a\_field today. |
| virtual bool | CutToHay (LE \*a\_field, double a\_user, int a\_days) |
|  | Carry out hay cutting on a\_field. |
| virtual bool | CutToSilage (LE \*a\_field, double a\_user, int a\_days) |
|  | Cut vegetation for silage on a\_field. |
| virtual bool | CutWeeds (LE \*a\_field, double a\_user, int a\_days) |
|  | Carry out weed topping on a\_field. |
| virtual bool | DeepPlough (LE \*a\_field, double a\_user, int a\_days) |
|  | Carry out a deep ploughing event on a\_field. |
| bool | DoIt (int a\_probability) |
|  | Return chance out of 100. |
| virtual bool | FA\_AmmoniumSulphate (LE \*a\_field, double a\_user, int a\_days) |
|  | Apply ammonium supahte to a\_field owned by an stock farmer. |
| virtual bool | FA\_GreenManure (LE \*a\_field, double a\_user, int a\_days) |
|  | Spread green manure on a\_field owned by an stock farmer. |
| virtual bool | FA\_Manure (LE \*a\_field, double a\_user, int a\_days) |
|  | Spread manure on a\_field owned by an stock farmer. |
| virtual bool | FA\_NPK (LE \*a\_field, double a\_user, int a\_days) |
|  | Apply NPK fertilizer to a\_field owned by an stock farmer. |
| virtual bool | FA\_PK (LE \*a\_field, double a\_user, int a\_days) |
|  | Apply PK fertilizer to a\_field owned by an stock farmer. |
| virtual bool | FA\_Sludge (LE \*a\_field, double a\_user, int a\_days) |
|  | Spread sewege sludge on a\_field owned by an stock farmer. |
| virtual bool | FA\_Slurry (LE \*a\_field, double a\_user, int a\_days) |
|  | Spready slurry on a\_field owned by an stock farmer. |
|  | Farm (void) |
|  | Farm constructor - creates an instance of each possible crop type. |
| virtual bool | FP\_GreenManure (LE \*a\_field, double a\_user, int a\_days) |
|  | Spread green manure on a\_field owned by an arable farmer. |
| virtual bool | FP\_LiquidNH3 (LE \*a\_field, double a\_user, int a\_days) |
|  | Apply liquid ammonia fertilizer to a\_field owned by an arable farmer. |
| virtual bool | FP\_ManganeseSulphate (LE \*a\_field, double a\_user, int a\_days) |
|  | Apply Manganse Sulphate to a\_field owned by an arable farmer. |
| virtual bool | FP\_Manure (LE \*a\_field, double a\_user, int a\_days) |
|  | Spread manure on a\_field owned by an arable farmer. |
| virtual bool | FP\_NPK (LE \*a\_field, double a\_user, int a\_days) |
|  | Apply NPK fertilizer, on a\_field owned by an arable farmer. |
| virtual bool | FP\_NPKS (LE \*a\_field, double a\_user, int a\_days) |
|  | Apply NPKS fertilizer, on a\_field owned by an arable farmer. |
| virtual bool | FP\_PK (LE \*a\_field, double a\_user, int a\_days) |
|  | Apply PK fertilizer, on a\_field owned by an arable farmer. |
| virtual bool | FP\_Sludge (LE \*a\_field, double a\_user, int a\_days) |
|  | Spread sewege on a\_field owned by an arable farmer. |
| virtual bool | FP\_Slurry (LE \*a\_field, double a\_user, int a\_days) |
|  | Apply slurry to a\_field owned by an arable farmer. |
| virtual bool | FungicideTreat (LE \*a\_field, double a\_user, int a\_days) |
|  | Apply fungicide to a\_field. |
| int | GetArea (void) |
|  | Returns the area of arable fields owned by that farm. |
| int | GetFarmNumber (void) |
| int | GetIntensity (void) |
| TTypesOfFarm | GetType (void) |
| virtual bool | Glyphosate (LE \*a\_field, double a\_user, int a\_days) |
| virtual bool | GrowthRegulator (LE \*a\_field, double a\_user, int a\_days) |
|  | Apply growth regulator to a\_field. |
| virtual bool | Harvest (LE \*a\_field, double a\_user, int a\_days) |
|  | Carry out a harvest on a\_field. |
| virtual bool | HayBailing (LE \*a\_field, double a\_user, int a\_days) |
|  | Carry out hay bailing on a\_field. |
| virtual bool | HayTurning (LE \*a\_field, double a\_user, int a\_days) |
|  | Carry out hay turning on a\_field. |
| virtual bool | HerbicideTreat (LE \*a\_field, double a\_user, int a\_days) |
|  | Apply herbicide to a\_field. |
| virtual bool | HillingUp (LE \*a\_field, double a\_user, int a\_days) |
|  | Do hilling up on a\_field, probably of potatoes. |
| virtual void | InitiateManagement (void) |
|  | Kicks off the farm's management. |
| virtual bool | InsecticideTreat (LE \*a\_field, double a\_user, int a\_days) |
|  | Apply insecticide to a\_field. |
| bool | IsStockFarmer (void) |
| virtual void | MakeStockFarmer (void) |
| virtual void | Management (void) |
|  | Starts the main management loop for the farm and performs some error checking. |
| virtual bool | Molluscicide (LE \*a\_field, double a\_user, int a\_days) |
|  | Apply molluscidie to a\_field. |
| virtual bool | PigsAreOut (LE \*a\_field, double a\_user, int a\_days) |
|  | Start a pig grazing event on a\_field today or soon. |
| virtual bool | PigsAreOutForced (LE \*a\_field, double a\_user, int a\_days) |
|  | Start a pig grazing event on a\_field today - no exceptions. |
| virtual bool | PigsOut (LE \*a\_field, double a\_user, int a\_days) |
|  | Generate a 'pigs\_out' event for every day the cattle are on a\_field. |
| virtual bool | ProductApplication0 (LE \*a\_field, double a\_user, int a\_days) |
|  | Apply test pesticide to a\_field. |
| virtual bool | ProductApplication1 (LE \*a\_field, double a\_user, int a\_days) |
|  | Apply test pesticide to a\_field. |
| void | RemoveField (LE \*a\_field) |
|  | Removes a field to a farm. |
| virtual bool | RowCultivation (LE \*a\_field, double a\_user, int a\_days) |
|  | Carry out a harrowing between crop rows on a\_field. |
| void | SetFarmNumber (int a\_farm\_num) |
| virtual bool | SleepAllDay (LE \*a\_field, double a\_user, int a\_days) |
|  | Nothing to to today on a\_field. |
| virtual bool | SpringHarrow (LE \*a\_field, double a\_user, int a\_days) |
|  | Carry out a harrow event in the spring on a\_field. |
| virtual bool | SpringPlough (LE \*a\_field, double a\_user, int a\_days) |
|  | Carry out a ploughing event in the spring on a\_field. |
| virtual bool | SpringRoll (LE \*a\_field, double a\_user, int a\_days) |
|  | Carry out a roll event in the spring on a\_field. |
| virtual bool | SpringSow (LE \*a\_field, double a\_user, int a\_days) |
|  | Carry out a sowing event in the spring on a\_field. |
| virtual bool | StrawChopping (LE \*a\_field, double a\_user, int a\_days) |
|  | Carry out straw chopping on a\_field. |
| virtual bool | Strigling (LE \*a\_field, double a\_user, int a\_days) |
|  | Carry out a mechanical weeding on a\_field. |
| virtual bool | StriglingSow (LE \*a\_field, double a\_user, int a\_days) |
|  | Carry out a mechanical weeding followed by sowing on a\_field. |
| virtual bool | StubbleHarrowing (LE \*a\_field, double a\_user, int a\_days) |
|  | Carry out stubble harrowing on a\_field. |
| virtual bool | Swathing (LE \*a\_field, double a\_user, int a\_days) |
|  | Cut the crop on a\_field and leave it lying (probably rape) |
| virtual bool | SynInsecticideTreat (LE \*a\_field, double a\_user, int a\_days) |
|  | Apply special insecticide to a\_field. |
| TTypesOfVegetation | TranslateCropCodes (std::string &str) |
| virtual bool | Trial\_Control (LE \*a\_field, double a\_user, int a\_days) |
|  | Special pesticide trial functionality. |
| virtual bool | Trial\_PesticideTreat (LE \*a\_field, double a\_user, int a\_days) |
|  | Special pesticide trial functionality. |
| virtual bool | Trial\_PesticideTreat\_GS (LE \*a\_field, double a\_user, int a\_days) |
|  | Special pesticide trial functionality. |
| virtual bool | Trial\_ToxicControl (LE \*a\_field, double a\_user, int a\_days) |
|  | Special pesticide trial functionality. |
| virtual bool | Water (LE \*a\_field, double a\_user, int a\_days) |
|  | Carry out a watering on a\_field. |
| virtual bool | WinterPlough (LE \*a\_field, double a\_user, int a\_days) |
|  | Carry out a ploughing event in the winter on a\_field. |
| virtual | ~Farm (void) |
|  | Farm destructor - deletes all crop instances and empties event queues. |

|  |  |
| --- | --- |
| Protected Member Functions | |
| void | CheckRotationManagementLoop (FarmEvent \*ev) |
| virtual int | GetFirstCropIndex (TTypesOfLandscapeElement a\_type) |
|  | Gets the first crop for the farm. |
| int | GetFirstDate (TTypesOfVegetation a\_tov) |
|  | Gets the start date for a crop type. |
| virtual int | GetNextCropIndex (int a\_rot\_index) |
|  | Returns the next crop in the rotation. |
| int | GetNextCropStartDate (LE \*a\_field, TTypesOfVegetation &a\_curr\_veg) |
|  | Returns the start date of the next crop in the rotation. |
| void | HandleEvents (void) |
|  | If there are events to carry out do this, and perhaps start a new crop. |
| bool | LeSwitch (FarmEvent \*ev) |
|  | Call do function for any crop with an outstanding event. Signal if the crop has terminated. |
| void | ReadRotation (std::string fname) |
|  | Reads a rotation file into the rotation. |

|  |  |
| --- | --- |
| Protected Attributes | |
| AgroChemIndustryCereal \* | m\_agrochemindustrycereal |
| Carrots \* | m\_carrots |
| CloverGrassGrazed1 \* | m\_CGG1 |
| CloverGrassGrazed2 \* | m\_CGG2 |
| int | m\_farm\_num |
| TTypesOfFarm | m\_farmtype |
| FieldPeas \* | m\_fieldpeas |
| FieldPeasStrigling \* | m\_fieldpeasstrigling |
| vector< LE \* > | m\_fields |
| Fodderbeet \* | m\_fodderbeet |
| FodderGrass \* | m\_foddergrass |
| int | m\_intensity |
| Maize \* | m\_maize |
| MaizeSilage \* | m\_maizesilage |
| MaizeStrigling \* | m\_maizestrigling |
| Oats \* | m\_oats |
| OBarleyPeaCloverGrass \* | m\_OBarleyPCG |
| OCarrots \* | m\_ocarrots |
| OCloverGrassGrazed1 \* | m\_OCGG1 |
| OCloverGrassGrazed2 \* | m\_OCGG2 |
| OCloverGrassSilage1 \* | m\_OCGS1 |
| OFieldPeas \* | m\_ofieldpeas |
| OFieldPeasSilage \* | m\_ofieldpeassilage |
| OFirstYearDanger \* | m\_ofirstyeardanger |
| OGrazingPigs \* | m\_ograzingpigs |
| OMaizeSilage \* | m\_omaizesilage |
| OOats \* | m\_ooats |
| OPermanentGrassGrazed \* | m\_opermgrassgrazed |
| OPotatoes \* | m\_opotatoes |
| OSBarleySilage \* | m\_osbarleysilage |
| OSpringBarley \* | m\_ospringbarley |
| OSpringBarleyExt \* | m\_ospringbarleyext |
| OSpringBarleyPigs \* | m\_ospringbarleypigs |
| OWinterBarley \* | m\_owinterbarley |
| OWinterBarleyExt \* | m\_owinterbarleyext |
| OWinterRape \* | m\_owinterrape |
| OWinterRye \* | m\_owinterrye |
| OWinterWheatUndersown \* | m\_owinterwheatundersown |
| OWinterWheatUndersownExt \* | m\_owinterwheatundersownext |
| PermanentSetAside \* | m\_permanentsetaside |
| vector< PermCropData > | m\_PermCrops |
| PermanentGrassGrazed \* | m\_permgrassgrazed |
| PermanentGrassLowYield \* | m\_permgrasslowyield |
| PermanentGrassTussocky \* | m\_permgrasstussocky |
| Potatoes \* | m\_potatoes |
| PotatoesIndustry \* | m\_potatoesindustry |
| LowPriority< FarmEvent \* > | m\_queue |
| vector< TTypesOfVegetation > | m\_rotation |
| int | m\_rotation\_sync\_index |
| SpringBarleyCloverGrass \* | m\_sbarleyclovergrass |
| SeedGrass1 \* | m\_seedgrass1 |
| SeedGrass2 \* | m\_seedgrass2 |
| SetAside \* | m\_setaside |
| SpringBarley \* | m\_springbarley |
| SpringBarleyCloverGrassStrigling \* | m\_springbarleyclovergrassstrigling |
| SpringBarleyPeaCloverGrassStrigling \* | m\_springbarleypeaclovergrassstrigling |
| SpringBarleyPTreatment \* | m\_springbarleyptreatment |
| SpringBarleySeed \* | m\_springbarleyseed |
| SpringBarleySilage \* | m\_springbarleysilage |
| SpringBarleySKManagement \* | m\_springbarleyskmanagement |
| SpringBarleyStrigling \* | m\_springbarleystrigling |
| SpringBarleyStriglingCulm \* | m\_springbarleystriglingculm |
| SpringBarleyStriglingSingle \* | m\_springbarleystriglingsingle |
| SpringRape \* | m\_springrape |
| bool | m\_stockfarmer |
| Triticale \* | m\_triticale |
| WinterBarley \* | m\_winterbarley |
| WinterBarleyStrigling \* | m\_winterbarleystrigling |
| WinterRape \* | m\_winterrape |
| WinterRapeStrigling \* | m\_winterrapestrigling |
| WinterRye \* | m\_winterrye |
| WinterRyeStrigling \* | m\_winterryestrigling |
| WinterWheat \* | m\_winterwheat |
| WinterWheatStrigling \* | m\_winterwheatstrigling |
| WinterWheatStriglingCulm \* | m\_winterwheatstriglingculm |
| WinterWheatStriglingSingle \* | m\_winterwheatstriglingsingle |
| WWheatPControl \* | m\_wwheatpcontrol |
| WWheatPToxicControl \* | m\_wwheatptoxiccontrol |
| WWheatPTreatment \* | m\_wwheatptreatment |
| YoungForestCrop \* | m\_youngforest |

---

## Detailed Description

The base class for all farm types.

---

## Constructor & Destructor Documentation

|  |  |  |  |  |  |
| --- | --- | --- | --- | --- | --- |
| Farm::Farm | ( | void |  | ) |  |

Farm constructor - creates an instance of each possible crop type.

{

m\_carrots = new Carrots;

m\_CGG1 = new CloverGrassGrazed1;

m\_CGG2 = new CloverGrassGrazed2;

m\_fieldpeas = new FieldPeas;

m\_fodderbeet = new Fodderbeet;

m\_foddergrass = new FodderGrass;

m\_maize = new Maize;

m\_maizesilage = new MaizeSilage;

m\_omaizesilage = new OMaizeSilage;

m\_OBarleyPCG = new OBarleyPeaCloverGrass;

m\_ocarrots = new OCarrots;

m\_OCGG1 = new OCloverGrassGrazed1;

m\_OCGG2 = new OCloverGrassGrazed2;

m\_OCGS1 = new OCloverGrassSilage1;

m\_ofieldpeas = new OFieldPeas;

m\_ofieldpeassilage = new OFieldPeasSilage;

m\_ofirstyeardanger = new OFirstYearDanger;

m\_ograzingpigs = new OGrazingPigs;

m\_oats = new Oats;

m\_ooats = new OOats;

m\_opermgrassgrazed = new OPermanentGrassGrazed;

m\_opotatoes = new OPotatoes;

m\_ospringbarley = new OSpringBarley;

m\_osbarleysilage = new OSBarleySilage;

m\_ospringbarleyext = new OSpringBarleyExt;

m\_ospringbarleypigs = new OSpringBarleyPigs;

m\_owinterbarley = new OWinterBarley;

m\_owinterbarleyext = new OWinterBarleyExt;

m\_owinterrape = new OWinterRape;

m\_owinterrye = new OWinterRye;

m\_owinterwheatundersown = new OWinterWheatUndersown;

m\_owinterwheatundersownext = new OWinterWheatUndersownExt;

m\_permanentsetaside = new PermanentSetAside;

m\_permgrassgrazed = new PermanentGrassGrazed;

m\_permgrasslowyield = new PermanentGrassLowYield;

m\_permgrasstussocky = new PermanentGrassTussocky;

m\_potatoes = new Potatoes;

m\_potatoesindustry = new PotatoesIndustry;

m\_sbarleyclovergrass = new SpringBarleyCloverGrass;

m\_seedgrass1 = new SeedGrass1;

m\_seedgrass2 = new SeedGrass2;

m\_setaside = new SetAside;

m\_springbarley = new SpringBarley;

m\_springbarleyptreatment = new SpringBarleyPTreatment;

m\_springbarleyskmanagement = new SpringBarleySKManagement;

m\_springbarleyseed = new SpringBarleySeed;

m\_springbarleysilage = new SpringBarleySilage;

m\_springrape = new SpringRape;

m\_triticale = new Triticale;

m\_winterbarley = new WinterBarley;

m\_winterrape = new WinterRape;

m\_winterrye = new WinterRye;

m\_winterwheat = new WinterWheat;

m\_wwheatpcontrol = new WWheatPControl;

m\_wwheatptoxiccontrol = new WWheatPToxicControl;

m\_wwheatptreatment = new WWheatPTreatment;

m\_agrochemindustrycereal = new AgroChemIndustryCereal;

m\_winterwheatstrigling = new WinterWheatStrigling;

m\_winterwheatstriglingsingle = new WinterWheatStriglingSingle;

m\_winterwheatstriglingculm = new WinterWheatStriglingCulm;

m\_springbarleyclovergrassstrigling = new SpringBarleyCloverGrassStrigling;

m\_springbarleystrigling = new SpringBarleyStrigling;

m\_springbarleystriglingsingle = new SpringBarleyStriglingSingle;

m\_springbarleystriglingculm = new SpringBarleyStriglingCulm;

m\_maizestrigling = new MaizeStrigling;

m\_winterrapestrigling = new WinterRapeStrigling;

m\_winterryestrigling = new WinterRyeStrigling;

m\_winterbarleystrigling = new WinterBarleyStrigling;

m\_fieldpeasstrigling = new FieldPeasStrigling;

m\_springbarleypeaclovergrassstrigling = new SpringBarleyPeaCloverGrassStrigling;

m\_youngforest = new YoungForestCrop;

m\_rotation\_sync\_index = -1;

// Defaults that need to be overridden when necessary

m\_stockfarmer = false;

m\_intensity = 0;

}

|  |  |  |  |  |  |  |  |
| --- | --- | --- | --- | --- | --- | --- | --- |
| |  |  |  |  |  |  | | --- | --- | --- | --- | --- | --- | | Farm::~Farm | ( | void |  | ) |  | | virtual |

Farm destructor - deletes all crop instances and empties event queues.

References LowPriPair< ELEMTYPE >::m\_element.

{

delete m\_carrots;

delete m\_CGG2;

delete m\_CGG1;

delete m\_fieldpeas;

delete m\_fodderbeet;

delete m\_foddergrass;

delete m\_maizesilage;

delete m\_omaizesilage;

delete m\_maize;

delete m\_ocarrots;

delete m\_OCGG1;

delete m\_OCGG2;

delete m\_OCGS1;

delete m\_ofieldpeas;

delete m\_ofieldpeassilage;

delete m\_ofirstyeardanger;

delete m\_ograzingpigs;

delete m\_ooats;

delete m\_oats;

delete m\_opermgrassgrazed;

delete m\_opotatoes;

delete m\_ospringbarley;

delete m\_ospringbarleyext;

delete m\_osbarleysilage;

delete m\_ospringbarleypigs;

delete m\_owinterbarley;

delete m\_owinterbarleyext;

delete m\_owinterrape;

delete m\_owinterrye;

delete m\_owinterwheatundersown;

delete m\_owinterwheatundersownext;

delete m\_OBarleyPCG;

delete m\_permanentsetaside;

delete m\_permgrassgrazed;

delete m\_permgrasstussocky;

delete m\_permgrasslowyield;

delete m\_potatoes;

delete m\_potatoesindustry;

delete m\_sbarleyclovergrass;

delete m\_seedgrass2;

delete m\_seedgrass1;

delete m\_setaside;

delete m\_springbarley;

delete m\_springbarleyptreatment;

delete m\_springbarleyskmanagement;

delete m\_springbarleyseed;

delete m\_springrape;

delete m\_springbarleysilage;

delete m\_agrochemindustrycereal;

delete m\_triticale;

delete m\_winterbarley;

delete m\_winterrape;

delete m\_winterrye;

delete m\_winterwheat;

delete m\_wwheatpcontrol;

delete m\_wwheatptoxiccontrol;

delete m\_wwheatptreatment;

delete m\_winterwheatstrigling;

delete m\_winterwheatstriglingsingle;

delete m\_winterwheatstriglingculm;

delete m\_springbarleyclovergrassstrigling;

delete m\_springbarleystrigling;

delete m\_springbarleystriglingsingle;

delete m\_springbarleystriglingculm;

delete m\_maizestrigling;

delete m\_winterrapestrigling;

delete m\_winterryestrigling;

delete m\_winterbarleystrigling;

delete m\_fieldpeasstrigling;

delete m\_springbarleypeaclovergrassstrigling;

delete m\_youngforest;

LowPriPair < FarmEvent \* > pair;

while ( !m\_queue.Empty() ) {

pair = m\_queue.Bottom();

m\_queue.Pop();

delete pair.m\_element;

}

}

---

## Member Function Documentation

|  |  |  |  |  |  |
| --- | --- | --- | --- | --- | --- |
| void Farm::AddField | ( | LE \* | *a\_newfield* | ) |  |

Adds a field to a farm.

References LE::GetElementType(), tole\_PermanentSetaside, tole\_PermPasture, tole\_PermPastureLowYield, tole\_PermPastureTussocky, and tole\_YoungForest.

{

int i = (int) m\_fields.size();

m\_fields.resize( i + 1 );

m\_fields[ i ] = a\_newfield;

// Must set the rot index to something other than -1, but identify it as not usefully set as yet.

TTypesOfLandscapeElement ele = a\_newfield->GetElementType();

switch (ele) {

case tole\_PermPastureLowYield:

case tole\_YoungForest:

case tole\_PermPasture:

case tole\_PermPastureTussocky:

case tole\_PermanentSetaside:

m\_fields[ i ]->SetRotIndex(-2);

break;

default:

m\_fields[ i ]->SetRotIndex(-1);

}

}

|  |  |  |  |
| --- | --- | --- | --- |
| void Farm::AddNewEvent | ( | TTypesOfVegetation | *a\_event*, |
|  |  | long | *a\_date*, |
|  |  | LE \* | *a\_field*, |
|  |  | int | *a\_todo*, |
|  |  | long | *a\_num*, |
|  |  | bool | *a\_lock*, |
|  |  | int | *a\_start*, |
|  |  | bool | *a\_first\_year*, |
|  |  | TTypesOfVegetation | *a\_crop* |
|  | ) |  |  |

Adds an event to the event queue for a farm.

{

FarmEvent \* ev = new FarmEvent( a\_event, a\_field, a\_todo, a\_run, a\_lock, a\_start, a\_first\_year, a\_crop );

m\_queue.Push( ev, a\_date );

}

|  |  |  |  |  |  |  |  |  |  |  |  |  |  |  |  |  |  |
| --- | --- | --- | --- | --- | --- | --- | --- | --- | --- | --- | --- | --- | --- | --- | --- | --- | --- |
| |  |  |  |  | | --- | --- | --- | --- | | bool Farm::AutumnHarrow | ( | LE \* | *a\_field*, | |  |  | double | *a\_user*, | |  |  | int | *a\_days* | |  | ) |  |  | | virtual |

Carry out a harrow event in the autumn on a\_field.

References autumn\_harrow, DO\_IT\_PROB, g\_landscape\_p, g\_weather, LE::GetUnsprayedMarginPolyRef(), LE::InsectMortality(), Weather::Raining(), LE::SetLastTreatment(), Landscape::SupplyLEPointer(), LE::Trace(), and LE::ZeroVeg().

{

if ( (0 >= a\_days) || (!g\_weather->Raining() && DoIt(DO\_IT\_PROB))) {

a\_field->Trace( autumn\_harrow );

a\_field->SetLastTreatment( autumn\_harrow );

// Apply 90% mortality to the insects

a\_field->InsectMortality( 0.1 );

// Reduce the vegetation to zero

a\_field->ZeroVeg();

int pref=a\_field->GetUnsprayedMarginPolyRef();

if (pref!=-1){

// Must have an unsprayed margin so need to pass the information on to it

LE\* um=g\_landscape\_p->SupplyLEPointer(pref);

um->SetLastTreatment( autumn\_harrow );

um->InsectMortality( 0.1 );

um->ZeroVeg();

}

return true;

}

return false;

}

|  |  |  |  |  |  |  |  |  |  |  |  |  |  |  |  |  |  |
| --- | --- | --- | --- | --- | --- | --- | --- | --- | --- | --- | --- | --- | --- | --- | --- | --- | --- |
| |  |  |  |  | | --- | --- | --- | --- | | bool Farm::AutumnPlough | ( | LE \* | *a\_field*, | |  |  | double | *a\_user*, | |  |  | int | *a\_days* | |  | ) |  |  | | virtual |

Carry out a ploughing event in the autumn on a\_field.

References autumn\_plough, DO\_IT\_PROB, g\_landscape\_p, g\_weather, LE::GetUnsprayedMarginPolyRef(), LE::InsectMortality(), Weather::Raining(), LE::SetLastTreatment(), Landscape::SupplyLEPointer(), LE::Trace(), and LE::ZeroVeg().

{

// LE is a pointer to the field element

// a\_user is a pointer to the farm

// a\_days is the end of the operation time - today

// if a\_days <0 then the time to do it is passed

// the line below reads 'plough if last day possible OR if not raining and pass a probability test

if ( (0 >= a\_days) || (!g\_weather->Raining() && DoIt(DO\_IT\_PROB))) {

// this bit sets up the events to occur when ploughing occurs

// The trace below is for debugging checks

a\_field->Trace( autumn\_plough );

// Record the event for this field, so other objects can find out it has happened

a\_field->SetLastTreatment( autumn\_plough );

// Apply mortality to the insects present, in this case 90%. This only affects the general insect model, any ALMaSS model species need to take their specific action.

a\_field->InsectMortality( 0.1 );

// Reduce the vegetation, in this case to zero

a\_field->ZeroVeg();

// If the field has a field margin, then do all this to the field margin too. In events that don't occur on an unsprayed margin, e.g. insecticide, then is part is skipped.

int pref=a\_field->GetUnsprayedMarginPolyRef();

if (pref!=-1){

// Must have an unsprayed margin so need to pass the information on to it

LE\* um=g\_landscape\_p->SupplyLEPointer(pref);

um->SetLastTreatment( autumn\_plough );

um->InsectMortality( 0.1 );

um->ZeroVeg();

}

return true; // completed

}

return false; // not completed

}

|  |  |  |  |  |  |  |  |  |  |  |  |  |  |  |  |  |  |
| --- | --- | --- | --- | --- | --- | --- | --- | --- | --- | --- | --- | --- | --- | --- | --- | --- | --- |
| |  |  |  |  | | --- | --- | --- | --- | | bool Farm::AutumnRoll | ( | LE \* | *a\_field*, | |  |  | double | *a\_user*, | |  |  | int | *a\_days* | |  | ) |  |  | | virtual |

Carry out a roll event in the autumn on a\_field.

References autumn\_roll, DO\_IT\_PROB, g\_landscape\_p, g\_weather, LE::GetUnsprayedMarginPolyRef(), Weather::Raining(), LE::SetLastTreatment(), Landscape::SupplyLEPointer(), LE::Trace(), and LE::ZeroVeg().

{

if ( (0 >= a\_days) || (!g\_weather->Raining() && DoIt(DO\_IT\_PROB))) {

a\_field->Trace( autumn\_roll );

a\_field->SetLastTreatment( autumn\_roll );

a\_field->ZeroVeg();

int pref=a\_field->GetUnsprayedMarginPolyRef();

if (pref!=-1){

// Must have an unsprayed margin so need to pass the information on to it

LE\* um=g\_landscape\_p->SupplyLEPointer(pref);

um->SetLastTreatment( autumn\_roll );

um->ZeroVeg();

}

return true;

}

return false;

}

|  |  |  |  |  |  |  |  |  |  |  |  |  |  |  |  |  |  |
| --- | --- | --- | --- | --- | --- | --- | --- | --- | --- | --- | --- | --- | --- | --- | --- | --- | --- |
| |  |  |  |  | | --- | --- | --- | --- | | bool Farm::AutumnSow | ( | LE \* | *a\_field*, | |  |  | double | *a\_user*, | |  |  | int | *a\_days* | |  | ) |  |  | | virtual |

Carry out a sowing event in the autumn on a\_field.

References autumn\_sow, DO\_IT\_PROB, g\_landscape\_p, g\_weather, LE::GetUnsprayedMarginPolyRef(), Weather::Raining(), LE::SetGrowthPhase(), LE::SetLastTreatment(), sow, Landscape::SupplyLEPointer(), LE::Trace(), and LE::ZeroVeg().

{

if ( (0 >= a\_days) || (!g\_weather->Raining() && DoIt(DO\_IT\_PROB))) {

a\_field->Trace( autumn\_sow );

a\_field->SetLastTreatment( autumn\_sow );

a\_field->SetGrowthPhase( sow );

// Reduce the vegetation to zero

a\_field->ZeroVeg();

int pref=a\_field->GetUnsprayedMarginPolyRef();

if (pref!=-1){

// Must have an unsprayed margin so need to pass the information on to it

LE\* um=g\_landscape\_p->SupplyLEPointer(pref);

um->SetLastTreatment( autumn\_sow );

um->SetGrowthPhase( sow );

um->ZeroVeg();

}

return true;

}

return false;

}

|  |  |  |  |  |  |  |  |  |  |  |  |  |  |  |  |  |  |
| --- | --- | --- | --- | --- | --- | --- | --- | --- | --- | --- | --- | --- | --- | --- | --- | --- | --- |
| |  |  |  |  | | --- | --- | --- | --- | | bool Farm::BurnStrawStubble | ( | LE \* | *a\_field*, | |  |  | double | *a\_user*, | |  |  | int | *a\_days* | |  | ) |  |  | | virtual |

Burn stubble on a\_field.

References burn\_straw\_stubble, Calendar::Date(), DO\_IT\_PROB, EL\_TRAMLINE\_DECAYTIME, g\_date, g\_landscape\_p, g\_weather, Weather::GetRainPeriod(), LE::GetUnsprayedMarginPolyRef(), LE::InsectMortality(), LE::ReduceVeg(), LE::SetLastTreatment(), LE::SetTramlinesDecay(), Landscape::SupplyLEPointer(), and LE::Trace().

{

if ( (0 >= a\_days) && (g\_weather->GetRainPeriod(g\_date->Date(),3)>0.1))

{

return true;

}

if ( (0 >= a\_days) || ((g\_weather->GetRainPeriod(g\_date->Date(),3)<0.1)

&& DoIt(DO\_IT\_PROB)))

{

a\_field->Trace( burn\_straw\_stubble );

a\_field->SetLastTreatment( burn\_straw\_stubble );

a\_field->InsectMortality( 0.4 );

a\_field->ReduceVeg( 0.2 );

a\_field->SetTramlinesDecay( EL\_TRAMLINE\_DECAYTIME );

int pref=a\_field->GetUnsprayedMarginPolyRef();

if (pref!=-1){

// Must have an unsprayed margin so need to pass the information on to it

LE\* um=g\_landscape\_p->SupplyLEPointer(pref);

um->SetLastTreatment( burn\_straw\_stubble );

um->ReduceVeg( 0.2 );

um->InsectMortality( 0.4 );

um->SetTramlinesDecay( EL\_TRAMLINE\_DECAYTIME );

}

return true;

}

return false;

}

|  |  |  |  |  |  |  |  |  |  |  |  |  |  |  |  |  |  |  |  |  |  |
| --- | --- | --- | --- | --- | --- | --- | --- | --- | --- | --- | --- | --- | --- | --- | --- | --- | --- | --- | --- | --- | --- |
| |  |  |  |  | | --- | --- | --- | --- | | bool Farm::CattleIsOut | ( | LE \* | *a\_field*, | |  |  | double | *a\_user*, | |  |  | int | *a\_days*, | |  |  | int | *a\_max* | |  | ) |  |  | | virtual |

Generate a 'cattle\_out' event for every day the cattle are on a\_field.

References cattle\_out, Calendar::DayInYear(), g\_date, g\_landscape\_p, LE::GetUnsprayedMarginPolyRef(), LE::GetVegHeight(), l\_farm\_cattle\_veg\_reduce, LE::ReduceVeg\_Extended(), LE::SetLastTreatment(), Landscape::SupplyLEPointer(), LE::ToggleCattleGrazing(), LE::Trace(), and CfgFloat::value().

{

a\_field->SetLastTreatment( cattle\_out );

a\_field->Trace( cattle\_out );

// Reduce the vegetation because of grazing

double h=a\_field->GetVegHeight();

double reduc = 1-(l\_farm\_cattle\_veg\_reduce.value()\*((h-15)/15));

a\_field->ReduceVeg\_Extended( reduc );

int pref=a\_field->GetUnsprayedMarginPolyRef();

if (pref!=-1){

// Must have an unsprayed margin so need to pass the information on to it

LE\* um=g\_landscape\_p->SupplyLEPointer(pref);

um->SetLastTreatment( cattle\_out );

um->ReduceVeg\_Extended( reduc );

}

// \*\*cjt\*\* added 24/05/03 to prevent conflict with date checking code

// in the management plans

int d1=g\_date->DayInYear(5,9);

if (d1>a\_max)

d1=a\_max;

if ( ( g\_date->DayInYear()> d1 ) &&

((0 >= a\_days)|| DoIt(50/a\_days))

) {

a\_field->ToggleCattleGrazing();

int pref=a\_field->GetUnsprayedMarginPolyRef();

if (pref!=-1){

// Must have an unsprayed margin so need to pass the information on to it

LE\* um=g\_landscape\_p->SupplyLEPointer(pref);

um->ToggleCattleGrazing();

}

return true;

}

return false;

}

|  |  |  |  |  |  |  |  |  |  |  |  |  |  |  |  |  |  |  |  |  |  |
| --- | --- | --- | --- | --- | --- | --- | --- | --- | --- | --- | --- | --- | --- | --- | --- | --- | --- | --- | --- | --- | --- |
| |  |  |  |  | | --- | --- | --- | --- | | bool Farm::CattleIsOutLow | ( | LE \* | *a\_field*, | |  |  | double | *a\_user*, | |  |  | int | *a\_days*, | |  |  | int | *a\_max* | |  | ) |  |  | | virtual |

Generate a 'cattle\_out\_low' event for every day the cattle are on a\_field.

References cattle\_out\_low, Calendar::DayInYear(), g\_date, g\_landscape\_p, LE::GetUnsprayedMarginPolyRef(), LE::GetVegHeight(), l\_farm\_cattle\_veg\_reduce2, LE::ReduceVeg\_Extended(), LE::SetLastTreatment(), Landscape::SupplyLEPointer(), LE::ToggleCattleGrazing(), LE::Trace(), and CfgFloat::value().

{

// Generate a 'cattle\_in\_out' event for every day the cattle is on the

// field.

a\_field->SetLastTreatment( cattle\_out\_low );

a\_field->Trace( cattle\_out\_low );

// Reduce the vegetation because of grazing

double h=a\_field->GetVegHeight();

double reduc = 1-(l\_farm\_cattle\_veg\_reduce2.value()\*((h-15)/15));

a\_field->ReduceVeg\_Extended( reduc );

int pref=a\_field->GetUnsprayedMarginPolyRef();

if (pref!=-1){

// Must have an unsprayed margin so need to pass the information on to it

LE\* um=g\_landscape\_p->SupplyLEPointer(pref);

um->SetLastTreatment( cattle\_out\_low );

um->ReduceVeg\_Extended( reduc );

}

// \*\*cjt\*\* added 24/05/03 to prevent conflict with date checking code

// in the management plans

int d1=g\_date->DayInYear(5,9);

if (d1>a\_max) d1=a\_max;

if ( ( g\_date->DayInYear()>g\_date->DayInYear(5,9) )&&

((0 >= a\_days)|| DoIt(50/a\_days)))

{

a\_field->ToggleCattleGrazing();

int pref=a\_field->GetUnsprayedMarginPolyRef();

if (pref!=-1){

// Must have an unsprayed margin so need to pass the information on to it

LE\* um=g\_landscape\_p->SupplyLEPointer(pref);

um->ToggleCattleGrazing();

}

return true;

}

return false;

}

|  |  |  |  |  |  |  |  |  |  |  |  |  |  |  |  |  |  |
| --- | --- | --- | --- | --- | --- | --- | --- | --- | --- | --- | --- | --- | --- | --- | --- | --- | --- |
| |  |  |  |  | | --- | --- | --- | --- | | bool Farm::CattleOut | ( | LE \* | *a\_field*, | |  |  | double | *a\_user*, | |  |  | int | *a\_days* | |  | ) |  |  | | virtual |

Start a grazing event on a\_field today.

References cattle\_out, DO\_IT\_PROB, g\_landscape\_p, LE::GetUnsprayedMarginPolyRef(), LE::GetVegHeight(), l\_farm\_cattle\_veg\_reduce, LE::ReduceVeg\_Extended(), LE::SetLastTreatment(), Landscape::SupplyLEPointer(), LE::ToggleCattleGrazing(), LE::Trace(), and CfgFloat::value().

{

if ( (0 >= a\_days)|| DoIt(DO\_IT\_PROB)) {

a\_field->ToggleCattleGrazing();

a\_field->Trace( cattle\_out );

a\_field->SetLastTreatment( cattle\_out );

// Reduce the vegetation because of grazing

double h=a\_field->GetVegHeight();

double reduc = 1-(l\_farm\_cattle\_veg\_reduce.value()\*((h-15)/15));

a\_field->ReduceVeg\_Extended( reduc );

int pref=a\_field->GetUnsprayedMarginPolyRef();

if (pref!=-1){

// Must have an unsprayed margin so need to pass the information on to it

// This happens if all arable fields are given unsprayed margins - they have no effect on grass unless it is sprayed with pesticides

LE\* um=g\_landscape\_p->SupplyLEPointer(pref);

um->ToggleCattleGrazing();

um->SetLastTreatment( cattle\_out );

um->ReduceVeg\_Extended( reduc );

}

return true;

}

return false;

}

|  |  |  |  |  |  |  |  |  |  |  |  |  |  |  |  |  |  |
| --- | --- | --- | --- | --- | --- | --- | --- | --- | --- | --- | --- | --- | --- | --- | --- | --- | --- |
| |  |  |  |  | | --- | --- | --- | --- | | bool Farm::CattleOutLowGrazing | ( | LE \* | *a\_field*, | |  |  | double | *a\_user*, | |  |  | int | *a\_days* | |  | ) |  |  | | virtual |

Start a extensive grazing event on a\_field today.

References cattle\_out\_low, DO\_IT\_PROB, g\_landscape\_p, LE::GetUnsprayedMarginPolyRef(), LE::GetVegHeight(), l\_farm\_cattle\_veg\_reduce2, LE::ReduceVeg\_Extended(), LE::SetLastTreatment(), Landscape::SupplyLEPointer(), LE::ToggleCattleGrazing(), LE::Trace(), and CfgFloat::value().

{

if ( (0 >= a\_days)|| DoIt(DO\_IT\_PROB)) {

a\_field->ToggleCattleGrazing();

a\_field->Trace( cattle\_out\_low );

a\_field->SetLastTreatment( cattle\_out\_low );

// Reduce the vegetation because of grazing

double h=a\_field->GetVegHeight();

double reduc = 1-(l\_farm\_cattle\_veg\_reduce2.value()\*((h-15)/15));

a\_field->ReduceVeg\_Extended( reduc );

int pref=a\_field->GetUnsprayedMarginPolyRef();

if (pref!=-1){

// Must have an unsprayed margin so need to pass the information on to it

// This happens if all arable fields are given unsprayed margins - they have no effect on grass unless it is sprayed with pesticides

LE\* um=g\_landscape\_p->SupplyLEPointer(pref);

um->ToggleCattleGrazing();

um->SetLastTreatment( cattle\_out\_low );

um->ReduceVeg\_Extended( reduc );

}

return true;

}

return false;

}

|  |  |  |  |  |  |  |  |
| --- | --- | --- | --- | --- | --- | --- | --- |
| |  |  |  |  |  |  | | --- | --- | --- | --- | --- | --- | | void Farm::CheckRotationManagementLoop | ( | FarmEvent \* | *ev* | ) |  | | protected |

Rotation error check function

References Calendar::Date(), g\_date, g\_msg, LE::GetMgtLoopDetectCount(), LE::GetMgtLoopDetectDate(), FarmEvent::m\_field, LE::SetMgtLoopDetectCount(), MapErrorMsg::Warn(), and WARN\_BUG.

{

if ( ev->m\_field->GetMgtLoopDetectDate() == g\_date->Date() ) {

// The last crop managment plan stopped on the same day as

// it was started.

// Bump loop counter.

ev->m\_field->SetMgtLoopDetectCount( ev->m\_field->GetMgtLoopDetectCount() + 1 );

if ( ev->m\_field->GetMgtLoopDetectCount() > ( long )( m\_rotation.size() + 2 ) ) {

// We have a loop.

char errornum[ 20 ];

sprintf( errornum, "%d", m\_farmtype );

g\_msg->Warn( WARN\_BUG, "Rotation management loop detected in farmtype ", errornum );

exit( 1 );

}

} else {

ev->m\_field->SetMgtLoopDetectCount( 0 );

}

}

|  |  |  |  |  |  |  |  |  |  |  |  |  |  |  |  |  |  |
| --- | --- | --- | --- | --- | --- | --- | --- | --- | --- | --- | --- | --- | --- | --- | --- | --- | --- |
| |  |  |  |  | | --- | --- | --- | --- | | bool Farm::CutToHay | ( | LE \* | *a\_field*, | |  |  | double | *a\_user*, | |  |  | int | *a\_days* | |  | ) |  |  | | virtual |

Carry out hay cutting on a\_field.

References cut\_to\_hay, Calendar::Date(), DO\_IT\_PROB, EL\_TRAMLINE\_DECAYTIME, g\_date, g\_landscape\_p, g\_weather, Weather::GetRainPeriod(), LE::GetUnsprayedMarginPolyRef(), LE::InsectMortality(), LE::ReduceVeg\_Extended(), LE::SetLastTreatment(), LE::SetTramlinesDecay(), LE::SetVegHeight(), Landscape::SupplyLEPointer(), and LE::Trace().

{

if ( (0 >= a\_days) || ((g\_weather->GetRainPeriod(g\_date->Date(),5)<0.1)

&& DoIt(DO\_IT\_PROB)))

{

a\_field->Trace( cut\_to\_hay );

a\_field->SetLastTreatment( cut\_to\_hay );

a\_field->InsectMortality( 0.4 );

a\_field->ReduceVeg\_Extended( 0.2 );

a\_field->SetVegHeight( 10, 0.3, 0.0, 0 );

a\_field->SetTramlinesDecay( EL\_TRAMLINE\_DECAYTIME );

int pref=a\_field->GetUnsprayedMarginPolyRef();

if (pref!=-1){

// Must have an unsprayed margin so need to pass the information on to it

LE\* um=g\_landscape\_p->SupplyLEPointer(pref);

um->SetLastTreatment( cut\_to\_hay );

um->InsectMortality( 0.4 );

um->ReduceVeg\_Extended( 0.2 );

um->SetVegHeight( 10, 0.3, 0.0, 0 );

um->SetTramlinesDecay( EL\_TRAMLINE\_DECAYTIME );

}

return true;

}

return false;

}

|  |  |  |  |  |  |  |  |  |  |  |  |  |  |  |  |  |  |
| --- | --- | --- | --- | --- | --- | --- | --- | --- | --- | --- | --- | --- | --- | --- | --- | --- | --- |
| |  |  |  |  | | --- | --- | --- | --- | | bool Farm::CutToSilage | ( | LE \* | *a\_field*, | |  |  | double | *a\_user*, | |  |  | int | *a\_days* | |  | ) |  |  | | virtual |

Cut vegetation for silage on a\_field.

References cut\_to\_silage, DO\_IT\_PROB, EL\_TRAMLINE\_DECAYTIME, g\_landscape\_p, g\_weather, LE::GetUnsprayedMarginPolyRef(), LE::InsectMortality(), Weather::Raining(), LE::ReduceVeg\_Extended(), LE::SetLastTreatment(), LE::SetTramlinesDecay(), LE::SetVegHeight(), Landscape::SupplyLEPointer(), and LE::Trace().

{

if ( (0 >= a\_days) || (!g\_weather->Raining() && DoIt(DO\_IT\_PROB))) {

a\_field->Trace( cut\_to\_silage );

a\_field->SetLastTreatment( cut\_to\_silage );

a\_field->ReduceVeg\_Extended( 0.2 );

a\_field->InsectMortality( 0.4 );

a\_field->SetVegHeight( 10, 0.3, 0.0, 0 );

a\_field->SetTramlinesDecay( EL\_TRAMLINE\_DECAYTIME );

int pref=a\_field->GetUnsprayedMarginPolyRef();

if (pref!=-1){

// Must have an unsprayed margin so need to pass the information on to it

LE\* um=g\_landscape\_p->SupplyLEPointer(pref);

um->SetLastTreatment( cut\_to\_silage );

um->ReduceVeg\_Extended( 0.2 );

um->InsectMortality( 0.4 );

um->SetVegHeight( 10, 0.3, 0.0, 0 );

um->SetTramlinesDecay( EL\_TRAMLINE\_DECAYTIME );

}

return true;

}

return false;

}

|  |  |  |  |  |  |  |  |  |  |  |  |  |  |  |  |  |  |
| --- | --- | --- | --- | --- | --- | --- | --- | --- | --- | --- | --- | --- | --- | --- | --- | --- | --- |
| |  |  |  |  | | --- | --- | --- | --- | | bool Farm::CutWeeds | ( | LE \* | *a\_field*, | |  |  | double | *a\_user*, | |  |  | int | *a\_days* | |  | ) |  |  | | virtual |

Carry out weed topping on a\_field.

References cut\_weeds, DO\_IT\_PROB, EL\_TRAMLINE\_DECAYTIME, g\_landscape\_p, g\_weather, LE::GetUnsprayedMarginPolyRef(), Weather::Raining(), LE::ReduceVeg(), LE::SetLastTreatment(), LE::SetTramlinesDecay(), Landscape::SupplyLEPointer(), and LE::Trace().

{

if ( (0 >= a\_days) || (!g\_weather->Raining() && DoIt(DO\_IT\_PROB)))

{

a\_field->Trace( cut\_weeds );

a\_field->SetLastTreatment( cut\_weeds );

a\_field->ReduceVeg( 0.8 );

a\_field->SetTramlinesDecay( EL\_TRAMLINE\_DECAYTIME );

int pref=a\_field->GetUnsprayedMarginPolyRef();

if (pref!=-1){

// Must have an unsprayed margin so need to pass the information on to it

LE\* um=g\_landscape\_p->SupplyLEPointer(pref);

um->SetLastTreatment( cut\_weeds );

um->ReduceVeg( 0.8 );

um->SetTramlinesDecay( EL\_TRAMLINE\_DECAYTIME );

}

return true;

}

return false;

}

|  |  |  |  |  |  |  |  |  |  |  |  |  |  |  |  |  |  |
| --- | --- | --- | --- | --- | --- | --- | --- | --- | --- | --- | --- | --- | --- | --- | --- | --- | --- |
| |  |  |  |  | | --- | --- | --- | --- | | bool Farm::DeepPlough | ( | LE \* | *a\_field*, | |  |  | double | *a\_user*, | |  |  | int | *a\_days* | |  | ) |  |  | | virtual |

Carry out a deep ploughing event on a\_field.

References deep\_ploughing, DO\_IT\_PROB, g\_landscape\_p, g\_weather, LE::GetUnsprayedMarginPolyRef(), LE::InsectMortality(), Weather::Raining(), LE::SetLastTreatment(), Landscape::SupplyLEPointer(), LE::Trace(), and LE::ZeroVeg().

{

if ( (0 >= a\_days) || (!g\_weather->Raining() && DoIt(DO\_IT\_PROB))) {

a\_field->Trace( deep\_ploughing );

a\_field->SetLastTreatment( deep\_ploughing );

// Apply 90% mortality to the insects

a\_field->InsectMortality( 0.1 );

// Reduce the vegetation to zero

a\_field->ZeroVeg();

int pref=a\_field->GetUnsprayedMarginPolyRef();

if (pref!=-1){

// Must have an unsprayed margin so need to pass the information on to it

LE\* um=g\_landscape\_p->SupplyLEPointer(pref);

um->SetLastTreatment( deep\_ploughing );

um->InsectMortality( 0.1 );

um->ZeroVeg();

}

return true;

}

return false;

}

|  |  |  |  |  |  |
| --- | --- | --- | --- | --- | --- |
| bool Farm::DoIt | ( | int | *a\_probability* | ) |  |

Return chance out of 100.

{

return ( a\_probability > ( int )( rand() % 100 ) );

}

|  |  |  |  |  |  |  |  |  |  |  |  |  |  |  |  |  |  |
| --- | --- | --- | --- | --- | --- | --- | --- | --- | --- | --- | --- | --- | --- | --- | --- | --- | --- |
| |  |  |  |  | | --- | --- | --- | --- | | bool Farm::FA\_AmmoniumSulphate | ( | LE \* | *a\_field*, | |  |  | double | *a\_user*, | |  |  | int | *a\_days* | |  | ) |  |  | | virtual |

Apply ammonium supahte to a\_field owned by an stock farmer.

References DO\_IT\_PROB, EL\_TRAMLINE\_DECAYTIME, fa\_ammoniumsulphate, g\_landscape\_p, g\_weather, LE::GetUnsprayedMarginPolyRef(), Weather::Raining(), LE::SetLastTreatment(), LE::SetTramlinesDecay(), Landscape::SupplyLEPointer(), and LE::Trace().

{

if ( (0 >= a\_days) || (!g\_weather->Raining() && DoIt(DO\_IT\_PROB))) {

a\_field->Trace( fa\_ammoniumsulphate );

a\_field->SetLastTreatment( fa\_ammoniumsulphate );

a\_field->SetTramlinesDecay( EL\_TRAMLINE\_DECAYTIME );

int pref=a\_field->GetUnsprayedMarginPolyRef();

if (pref!=-1){

// Must have an unsprayed margin so need to pass the information on to it

LE\* um=g\_landscape\_p->SupplyLEPointer(pref);

um->SetLastTreatment( fa\_ammoniumsulphate );

um->SetTramlinesDecay( EL\_TRAMLINE\_DECAYTIME );

}

return true;

}

return false;

}

|  |  |  |  |  |  |  |  |  |  |  |  |  |  |  |  |  |  |
| --- | --- | --- | --- | --- | --- | --- | --- | --- | --- | --- | --- | --- | --- | --- | --- | --- | --- |
| |  |  |  |  | | --- | --- | --- | --- | | bool Farm::FA\_GreenManure | ( | LE \* | *a\_field*, | |  |  | double | *a\_user*, | |  |  | int | *a\_days* | |  | ) |  |  | | virtual |

Spread green manure on a\_field owned by an stock farmer.

References DO\_IT\_PROB, EL\_TRAMLINE\_DECAYTIME, fa\_greenmanure, g\_landscape\_p, g\_weather, LE::GetUnsprayedMarginPolyRef(), Weather::Raining(), LE::SetLastTreatment(), LE::SetTramlinesDecay(), Landscape::SupplyLEPointer(), and LE::Trace().

{

if ( (0 >= a\_days) || (!g\_weather->Raining() && DoIt(DO\_IT\_PROB))) {

a\_field->Trace( fa\_greenmanure );

a\_field->SetLastTreatment( fa\_greenmanure );

a\_field->SetTramlinesDecay( EL\_TRAMLINE\_DECAYTIME );

int pref=a\_field->GetUnsprayedMarginPolyRef();

if (pref!=-1){

// Must have an unsprayed margin so need to pass the information on to it

LE\* um=g\_landscape\_p->SupplyLEPointer(pref);

um->SetLastTreatment( fa\_greenmanure );

um->SetTramlinesDecay( EL\_TRAMLINE\_DECAYTIME );

}

return true;

}

return false;

}

|  |  |  |  |  |  |  |  |  |  |  |  |  |  |  |  |  |  |
| --- | --- | --- | --- | --- | --- | --- | --- | --- | --- | --- | --- | --- | --- | --- | --- | --- | --- |
| |  |  |  |  | | --- | --- | --- | --- | | bool Farm::FA\_Manure | ( | LE \* | *a\_field*, | |  |  | double | *a\_user*, | |  |  | int | *a\_days* | |  | ) |  |  | | virtual |

Spread manure on a\_field owned by an stock farmer.

References DO\_IT\_PROB, EL\_TRAMLINE\_DECAYTIME, fa\_manure, g\_landscape\_p, g\_weather, Weather::GetTemp(), LE::GetUnsprayedMarginPolyRef(), Weather::Raining(), LE::SetLastTreatment(), LE::SetTramlinesDecay(), Landscape::SupplyLEPointer(), and LE::Trace().

{

if ( (0 >= a\_days) || ((g\_weather->GetTemp()>0)&&

!g\_weather->Raining() && DoIt(DO\_IT\_PROB)))

{

a\_field->Trace( fa\_manure );

a\_field->SetLastTreatment( fa\_manure );

a\_field->SetTramlinesDecay( EL\_TRAMLINE\_DECAYTIME );

int pref=a\_field->GetUnsprayedMarginPolyRef();

if (pref!=-1){

// Must have an unsprayed margin so need to pass the information on to it

LE\* um=g\_landscape\_p->SupplyLEPointer(pref);

um->SetLastTreatment( fa\_manure );

um->SetTramlinesDecay( EL\_TRAMLINE\_DECAYTIME );

}

return true;

}

return false;

}

|  |  |  |  |  |  |  |  |  |  |  |  |  |  |  |  |  |  |
| --- | --- | --- | --- | --- | --- | --- | --- | --- | --- | --- | --- | --- | --- | --- | --- | --- | --- |
| |  |  |  |  | | --- | --- | --- | --- | | bool Farm::FA\_NPK | ( | LE \* | *a\_field*, | |  |  | double | *a\_user*, | |  |  | int | *a\_days* | |  | ) |  |  | | virtual |

Apply NPK fertilizer to a\_field owned by an stock farmer.

References DO\_IT\_PROB, EL\_TRAMLINE\_DECAYTIME, fa\_npk, g\_landscape\_p, g\_weather, LE::GetUnsprayedMarginPolyRef(), Weather::Raining(), LE::SetLastTreatment(), LE::SetTramlinesDecay(), Landscape::SupplyLEPointer(), and LE::Trace().

{

if ( (0 >= a\_days) || (!g\_weather->Raining() && DoIt(DO\_IT\_PROB))) {

a\_field->Trace( fa\_npk );

a\_field->SetLastTreatment( fa\_npk );

a\_field->SetTramlinesDecay( EL\_TRAMLINE\_DECAYTIME );

int pref=a\_field->GetUnsprayedMarginPolyRef();

if (pref!=-1){

// Must have an unsprayed margin so need to pass the information on to it

LE\* um=g\_landscape\_p->SupplyLEPointer(pref);

um->SetLastTreatment( fa\_npk );

um->SetTramlinesDecay( EL\_TRAMLINE\_DECAYTIME );

}

return true;

}

return false;

}

|  |  |  |  |  |  |  |  |  |  |  |  |  |  |  |  |  |  |
| --- | --- | --- | --- | --- | --- | --- | --- | --- | --- | --- | --- | --- | --- | --- | --- | --- | --- |
| |  |  |  |  | | --- | --- | --- | --- | | bool Farm::FA\_PK | ( | LE \* | *a\_field*, | |  |  | double | *a\_user*, | |  |  | int | *a\_days* | |  | ) |  |  | | virtual |

Apply PK fertilizer to a\_field owned by an stock farmer.

References DO\_IT\_PROB, EL\_TRAMLINE\_DECAYTIME, fa\_pk, g\_landscape\_p, g\_weather, LE::GetUnsprayedMarginPolyRef(), Weather::Raining(), LE::SetLastTreatment(), LE::SetTramlinesDecay(), Landscape::SupplyLEPointer(), and LE::Trace().

{

if ( (0 >= a\_days) || (!g\_weather->Raining() && DoIt(DO\_IT\_PROB))) {

a\_field->Trace( fa\_pk );

a\_field->SetLastTreatment( fa\_pk );

a\_field->SetTramlinesDecay( EL\_TRAMLINE\_DECAYTIME );

int pref=a\_field->GetUnsprayedMarginPolyRef();

if (pref!=-1){

// Must have an unsprayed margin so need to pass the information on to it

LE\* um=g\_landscape\_p->SupplyLEPointer(pref);

um->SetLastTreatment( fa\_pk );

um->SetTramlinesDecay( EL\_TRAMLINE\_DECAYTIME );

}

return true;

}

return false;

}

|  |  |  |  |  |  |  |  |  |  |  |  |  |  |  |  |  |  |
| --- | --- | --- | --- | --- | --- | --- | --- | --- | --- | --- | --- | --- | --- | --- | --- | --- | --- |
| |  |  |  |  | | --- | --- | --- | --- | | bool Farm::FA\_Sludge | ( | LE \* | *a\_field*, | |  |  | double | *a\_user*, | |  |  | int | *a\_days* | |  | ) |  |  | | virtual |

Spread sewege sludge on a\_field owned by an stock farmer.

References DO\_IT\_PROB, EL\_TRAMLINE\_DECAYTIME, fa\_sludge, g\_landscape\_p, g\_weather, Weather::GetTemp(), LE::GetUnsprayedMarginPolyRef(), Weather::Raining(), LE::SetLastTreatment(), LE::SetTramlinesDecay(), Landscape::SupplyLEPointer(), and LE::Trace().

{

if ( (0 >= a\_days) || ((g\_weather->GetTemp()>0)&&

!g\_weather->Raining() && DoIt(DO\_IT\_PROB)))

{

a\_field->Trace( fa\_sludge );

a\_field->SetLastTreatment( fa\_sludge );

a\_field->SetTramlinesDecay( EL\_TRAMLINE\_DECAYTIME );

int pref=a\_field->GetUnsprayedMarginPolyRef();

if (pref!=-1){

// Must have an unsprayed margin so need to pass the information on to it

LE\* um=g\_landscape\_p->SupplyLEPointer(pref);

um->SetLastTreatment( fa\_sludge );

um->SetTramlinesDecay( EL\_TRAMLINE\_DECAYTIME );

}

return true;

}

return false;

}

|  |  |  |  |  |  |  |  |  |  |  |  |  |  |  |  |  |  |
| --- | --- | --- | --- | --- | --- | --- | --- | --- | --- | --- | --- | --- | --- | --- | --- | --- | --- |
| |  |  |  |  | | --- | --- | --- | --- | | bool Farm::FA\_Slurry | ( | LE \* | *a\_field*, | |  |  | double | *a\_user*, | |  |  | int | *a\_days* | |  | ) |  |  | | virtual |

Spready slurry on a\_field owned by an stock farmer.

References DO\_IT\_PROB, EL\_TRAMLINE\_DECAYTIME, fa\_slurry, g\_landscape\_p, g\_weather, Weather::GetTemp(), LE::GetUnsprayedMarginPolyRef(), Weather::Raining(), LE::SetLastTreatment(), LE::SetTramlinesDecay(), Landscape::SupplyLEPointer(), and LE::Trace().

{

if ( (0 >= a\_days) || ((g\_weather->GetTemp()>0)&&

!g\_weather->Raining() && DoIt(DO\_IT\_PROB)))

{

a\_field->Trace( fa\_slurry );

a\_field->SetLastTreatment( fa\_slurry );

a\_field->SetTramlinesDecay( EL\_TRAMLINE\_DECAYTIME );

int pref=a\_field->GetUnsprayedMarginPolyRef();

if (pref!=-1){

// Must have an unsprayed margin so need to pass the information on to it

LE\* um=g\_landscape\_p->SupplyLEPointer(pref);

um->SetLastTreatment( fa\_slurry );

um->SetTramlinesDecay( EL\_TRAMLINE\_DECAYTIME );

}

return true;

}

return false;

}

|  |  |  |  |  |  |  |  |  |  |  |  |  |  |  |  |  |  |
| --- | --- | --- | --- | --- | --- | --- | --- | --- | --- | --- | --- | --- | --- | --- | --- | --- | --- |
| |  |  |  |  | | --- | --- | --- | --- | | bool Farm::FP\_GreenManure | ( | LE \* | *a\_field*, | |  |  | double | *a\_user*, | |  |  | int | *a\_days* | |  | ) |  |  | | virtual |

Spread green manure on a\_field owned by an arable farmer.

References DO\_IT\_PROB, EL\_TRAMLINE\_DECAYTIME, fp\_greenmanure, g\_landscape\_p, g\_weather, LE::GetUnsprayedMarginPolyRef(), Weather::Raining(), LE::SetLastTreatment(), LE::SetTramlinesDecay(), Landscape::SupplyLEPointer(), and LE::Trace().

{

if ( (0 >= a\_days) || (!g\_weather->Raining() && DoIt(DO\_IT\_PROB))) {

a\_field->Trace( fp\_greenmanure );

a\_field->SetLastTreatment( fp\_greenmanure );

a\_field->SetTramlinesDecay( EL\_TRAMLINE\_DECAYTIME );

int pref=a\_field->GetUnsprayedMarginPolyRef();

if (pref!=-1){

// Must have an unsprayed margin so need to pass the information on to it

LE\* um=g\_landscape\_p->SupplyLEPointer(pref);

um->SetLastTreatment( fp\_greenmanure );

um->SetTramlinesDecay( EL\_TRAMLINE\_DECAYTIME );

}

return true;

}

return false;

}

|  |  |  |  |  |  |  |  |  |  |  |  |  |  |  |  |  |  |
| --- | --- | --- | --- | --- | --- | --- | --- | --- | --- | --- | --- | --- | --- | --- | --- | --- | --- |
| |  |  |  |  | | --- | --- | --- | --- | | bool Farm::FP\_LiquidNH3 | ( | LE \* | *a\_field*, | |  |  | double | *a\_user*, | |  |  | int | *a\_days* | |  | ) |  |  | | virtual |

Apply liquid ammonia fertilizer to a\_field owned by an arable farmer.

References DO\_IT\_PROB, EL\_TRAMLINE\_DECAYTIME, fp\_liquidNH3, g\_landscape\_p, g\_weather, LE::GetUnsprayedMarginPolyRef(), Weather::Raining(), LE::SetLastTreatment(), LE::SetTramlinesDecay(), Landscape::SupplyLEPointer(), and LE::Trace().

{

if ( (0 >= a\_days) || (!g\_weather->Raining() && DoIt(DO\_IT\_PROB))) {

a\_field->Trace( fp\_liquidNH3 );

a\_field->SetLastTreatment( fp\_liquidNH3 );

a\_field->SetTramlinesDecay( EL\_TRAMLINE\_DECAYTIME );

int pref=a\_field->GetUnsprayedMarginPolyRef();

if (pref!=-1){

// Must have an unsprayed margin so need to pass the information on to it

LE\* um=g\_landscape\_p->SupplyLEPointer(pref);

um->SetLastTreatment(fp\_liquidNH3);

um->SetTramlinesDecay( EL\_TRAMLINE\_DECAYTIME );

}

return true;

}

return false;

}

|  |  |  |  |  |  |  |  |  |  |  |  |  |  |  |  |  |  |
| --- | --- | --- | --- | --- | --- | --- | --- | --- | --- | --- | --- | --- | --- | --- | --- | --- | --- |
| |  |  |  |  | | --- | --- | --- | --- | | bool Farm::FP\_ManganeseSulphate | ( | LE \* | *a\_field*, | |  |  | double | *a\_user*, | |  |  | int | *a\_days* | |  | ) |  |  | | virtual |

Apply Manganse Sulphate to a\_field owned by an arable farmer.

References DO\_IT\_PROB, EL\_TRAMLINE\_DECAYTIME, fp\_manganesesulphate, g\_landscape\_p, g\_weather, LE::GetUnsprayedMarginPolyRef(), Weather::Raining(), LE::SetLastTreatment(), LE::SetTramlinesDecay(), Landscape::SupplyLEPointer(), and LE::Trace().

{

if ( (0 >= a\_days) || (!g\_weather->Raining() && DoIt(DO\_IT\_PROB))) {

a\_field->Trace( fp\_manganesesulphate );

a\_field->SetLastTreatment( fp\_manganesesulphate );

a\_field->SetTramlinesDecay( EL\_TRAMLINE\_DECAYTIME );

int pref=a\_field->GetUnsprayedMarginPolyRef();

if (pref!=-1){

// Must have an unsprayed margin so need to pass the information on to it

LE\* um=g\_landscape\_p->SupplyLEPointer(pref);

um->SetLastTreatment( fp\_manganesesulphate );

um->SetTramlinesDecay( EL\_TRAMLINE\_DECAYTIME );

}

return true;

}

return false;

}

|  |  |  |  |  |  |  |  |  |  |  |  |  |  |  |  |  |  |
| --- | --- | --- | --- | --- | --- | --- | --- | --- | --- | --- | --- | --- | --- | --- | --- | --- | --- |
| |  |  |  |  | | --- | --- | --- | --- | | bool Farm::FP\_Manure | ( | LE \* | *a\_field*, | |  |  | double | *a\_user*, | |  |  | int | *a\_days* | |  | ) |  |  | | virtual |

Spread manure on a\_field owned by an arable farmer.

References DO\_IT\_PROB, EL\_TRAMLINE\_DECAYTIME, fp\_manure, g\_landscape\_p, g\_weather, Weather::GetTemp(), LE::GetUnsprayedMarginPolyRef(), Weather::Raining(), LE::SetLastTreatment(), LE::SetTramlinesDecay(), Landscape::SupplyLEPointer(), and LE::Trace().

{

if ( (0 >= a\_days) || ((g\_weather->GetTemp()>0)&&

!g\_weather->Raining() && DoIt(DO\_IT\_PROB))) {

a\_field->Trace( fp\_manure );

a\_field->SetLastTreatment( fp\_manure );

a\_field->SetTramlinesDecay( EL\_TRAMLINE\_DECAYTIME );

int pref=a\_field->GetUnsprayedMarginPolyRef();

if (pref!=-1){

// Must have an unsprayed margin so need to pass the information on to it

LE\* um=g\_landscape\_p->SupplyLEPointer(pref);

um->SetLastTreatment( fp\_manure );

um->SetTramlinesDecay( EL\_TRAMLINE\_DECAYTIME );

}

return true;

}

return false;

}

|  |  |  |  |  |  |  |  |  |  |  |  |  |  |  |  |  |  |
| --- | --- | --- | --- | --- | --- | --- | --- | --- | --- | --- | --- | --- | --- | --- | --- | --- | --- |
| |  |  |  |  | | --- | --- | --- | --- | | bool Farm::FP\_NPK | ( | LE \* | *a\_field*, | |  |  | double | *a\_user*, | |  |  | int | *a\_days* | |  | ) |  |  | | virtual |

Apply NPK fertilizer, on a\_field owned by an arable farmer.

References DO\_IT\_PROB, EL\_TRAMLINE\_DECAYTIME, fp\_npk, g\_landscape\_p, g\_weather, LE::GetUnsprayedMarginPolyRef(), Weather::Raining(), LE::SetLastTreatment(), LE::SetTramlinesDecay(), Landscape::SupplyLEPointer(), and LE::Trace().

{

if ( (0 >= a\_days) || (!g\_weather->Raining() && DoIt(DO\_IT\_PROB))) {

a\_field->Trace( fp\_npk );

a\_field->SetLastTreatment( fp\_npk );

a\_field->SetTramlinesDecay( EL\_TRAMLINE\_DECAYTIME );

int pref=a\_field->GetUnsprayedMarginPolyRef();

if (pref!=-1){

// Must have an unsprayed margin so need to pass the information on to it

LE\* um=g\_landscape\_p->SupplyLEPointer(pref);

um->SetLastTreatment(fp\_npk);

um->SetTramlinesDecay( EL\_TRAMLINE\_DECAYTIME );

}

return true;

}

return false;

}

|  |  |  |  |  |  |  |  |  |  |  |  |  |  |  |  |  |  |
| --- | --- | --- | --- | --- | --- | --- | --- | --- | --- | --- | --- | --- | --- | --- | --- | --- | --- |
| |  |  |  |  | | --- | --- | --- | --- | | bool Farm::FP\_NPKS | ( | LE \* | *a\_field*, | |  |  | double | *a\_user*, | |  |  | int | *a\_days* | |  | ) |  |  | | virtual |

Apply NPKS fertilizer, on a\_field owned by an arable farmer.

References DO\_IT\_PROB, EL\_TRAMLINE\_DECAYTIME, fp\_npks, g\_landscape\_p, g\_weather, LE::GetUnsprayedMarginPolyRef(), harvest1, LE::InsectMortality(), Weather::Raining(), LE::SetGrowthPhase(), LE::SetLastTreatment(), LE::SetTramlinesDecay(), sleep\_all\_day, Landscape::SupplyLEPointer(), and LE::Trace().

{

if ( (0 >= a\_days) || (!g\_weather->Raining() && DoIt(DO\_IT\_PROB))) {

a\_field->Trace( fp\_npks );

a\_field->SetLastTreatment( fp\_npks );

a\_field->SetTramlinesDecay( EL\_TRAMLINE\_DECAYTIME );

int pref=a\_field->GetUnsprayedMarginPolyRef();

if (pref!=-1){

// Must have an unsprayed margin so need to pass the information on to it

LE\* um=g\_landscape\_p->SupplyLEPointer(pref);

um->SetLastTreatment(sleep\_all\_day);

um->SetGrowthPhase( harvest1 );

um->InsectMortality( 0.4 );

um->SetTramlinesDecay( EL\_TRAMLINE\_DECAYTIME );

}

return true;

}

return false;

}

|  |  |  |  |  |  |  |  |  |  |  |  |  |  |  |  |  |  |
| --- | --- | --- | --- | --- | --- | --- | --- | --- | --- | --- | --- | --- | --- | --- | --- | --- | --- |
| |  |  |  |  | | --- | --- | --- | --- | | bool Farm::FP\_PK | ( | LE \* | *a\_field*, | |  |  | double | *a\_user*, | |  |  | int | *a\_days* | |  | ) |  |  | | virtual |

Apply PK fertilizer, on a\_field owned by an arable farmer.

References DO\_IT\_PROB, EL\_TRAMLINE\_DECAYTIME, fp\_pk, g\_landscape\_p, g\_weather, LE::GetUnsprayedMarginPolyRef(), Weather::Raining(), LE::SetLastTreatment(), LE::SetTramlinesDecay(), Landscape::SupplyLEPointer(), and LE::Trace().

{

if ( (0 >= a\_days) || (!g\_weather->Raining() && DoIt(DO\_IT\_PROB))) {

a\_field->Trace( fp\_pk );

a\_field->SetLastTreatment( fp\_pk );

a\_field->SetTramlinesDecay( EL\_TRAMLINE\_DECAYTIME );

int pref=a\_field->GetUnsprayedMarginPolyRef();

if (pref!=-1){

// Must have an unsprayed margin so need to pass the information on to it

LE\* um=g\_landscape\_p->SupplyLEPointer(pref);

um->SetLastTreatment(fp\_pk);

um->SetTramlinesDecay( EL\_TRAMLINE\_DECAYTIME );

}

return true;

}

return false;

}

|  |  |  |  |  |  |  |  |  |  |  |  |  |  |  |  |  |  |
| --- | --- | --- | --- | --- | --- | --- | --- | --- | --- | --- | --- | --- | --- | --- | --- | --- | --- |
| |  |  |  |  | | --- | --- | --- | --- | | bool Farm::FP\_Sludge | ( | LE \* | *a\_field*, | |  |  | double | *a\_user*, | |  |  | int | *a\_days* | |  | ) |  |  | | virtual |

Spread sewege on a\_field owned by an arable farmer.

References DO\_IT\_PROB, EL\_TRAMLINE\_DECAYTIME, fp\_sludge, g\_landscape\_p, g\_weather, Weather::GetTemp(), LE::GetUnsprayedMarginPolyRef(), Weather::Raining(), LE::SetLastTreatment(), LE::SetTramlinesDecay(), Landscape::SupplyLEPointer(), and LE::Trace().

{

//

if ( (0 >= a\_days) || ((g\_weather->GetTemp()>0)&&

!g\_weather->Raining() && DoIt(DO\_IT\_PROB)))

{

a\_field->Trace( fp\_sludge );

a\_field->SetLastTreatment( fp\_sludge );

a\_field->SetTramlinesDecay( EL\_TRAMLINE\_DECAYTIME );

int pref=a\_field->GetUnsprayedMarginPolyRef();

if (pref!=-1){

// Must have an unsprayed margin so need to pass the information on to it

LE\* um=g\_landscape\_p->SupplyLEPointer(pref);

um->SetLastTreatment( fp\_sludge );

um->SetTramlinesDecay( EL\_TRAMLINE\_DECAYTIME );

}

return true;

}

return false;

}

|  |  |  |  |  |  |  |  |  |  |  |  |  |  |  |  |  |  |
| --- | --- | --- | --- | --- | --- | --- | --- | --- | --- | --- | --- | --- | --- | --- | --- | --- | --- |
| |  |  |  |  | | --- | --- | --- | --- | | bool Farm::FP\_Slurry | ( | LE \* | *a\_field*, | |  |  | double | *a\_user*, | |  |  | int | *a\_days* | |  | ) |  |  | | virtual |

Apply slurry to a\_field owned by an arable farmer.

References DO\_IT\_PROB, EL\_TRAMLINE\_DECAYTIME, fp\_slurry, g\_landscape\_p, g\_weather, Weather::GetTemp(), LE::GetUnsprayedMarginPolyRef(), Weather::Raining(), LE::SetLastTreatment(), LE::SetTramlinesDecay(), Landscape::SupplyLEPointer(), and LE::Trace().

{

if ( (0 >= a\_days) || ((g\_weather->GetTemp()>0)&&

!g\_weather->Raining() && DoIt(DO\_IT\_PROB)))

{

a\_field->Trace( fp\_slurry );

a\_field->SetLastTreatment( fp\_slurry );

a\_field->SetTramlinesDecay( EL\_TRAMLINE\_DECAYTIME );

int pref=a\_field->GetUnsprayedMarginPolyRef();

if (pref!=-1){

// Must have an unsprayed margin so need to pass the information on to it

LE\* um=g\_landscape\_p->SupplyLEPointer(pref);

um->SetLastTreatment(fp\_slurry);

um->SetTramlinesDecay( EL\_TRAMLINE\_DECAYTIME );

}

return true;

}

return false;

}

|  |  |  |  |  |  |  |  |  |  |  |  |  |  |  |  |  |  |
| --- | --- | --- | --- | --- | --- | --- | --- | --- | --- | --- | --- | --- | --- | --- | --- | --- | --- |
| |  |  |  |  | | --- | --- | --- | --- | | bool Farm::FungicideTreat | ( | LE \* | *a\_field*, | |  |  | double | *a\_user*, | |  |  | int | *a\_days* | |  | ) |  |  | | virtual |

Apply fungicide to a\_field.

References DO\_IT\_PROB, EL\_TRAMLINE\_DECAYTIME, fungicide\_treat, g\_weather, LE::GetSignal(), Weather::GetWind(), LE\_SIG\_NO\_FUNGICIDE, Weather::Raining(), LE::SetLastTreatment(), LE::SetTramlinesDecay(), and LE::Trace().

{

if (0 >= a\_days)

{

if ( (!g\_weather->Raining()) && (g\_weather->GetWind()<4.5) &&

( ! a\_field->GetSignal() & LE\_SIG\_NO\_FUNGICIDE )) {

a\_field->Trace( fungicide\_treat );

a\_field->SetLastTreatment( fungicide\_treat );

a\_field->SetTramlinesDecay( EL\_TRAMLINE\_DECAYTIME );

}

return true;

}

else if ( (g\_weather->GetWind()<4.5) &&

(!g\_weather->Raining()) && DoIt(DO\_IT\_PROB)) {

if ( ! (a\_field->GetSignal() & LE\_SIG\_NO\_FUNGICIDE) ) {

a\_field->Trace( fungicide\_treat );

a\_field->SetLastTreatment( fungicide\_treat );

a\_field->SetTramlinesDecay( EL\_TRAMLINE\_DECAYTIME );

}

return true;

}

return false;

}

|  |  |  |  |  |  |
| --- | --- | --- | --- | --- | --- |
| int Farm::GetArea | ( | void |  | ) |  |

Returns the area of arable fields owned by that farm.

References tole\_Field.

Referenced by UserDefinedFarm::AssignPermanentCrop().

{

int area = 0;

for ( unsigned int i = 0; i < m\_fields.size(); i++ ) {

if (m\_fields[i]->GetElementType()==tole\_Field) area += (int) m\_fields[i]->GetArea();

}

return area;

}

|  |  |  |  |  |  |  |  |
| --- | --- | --- | --- | --- | --- | --- | --- |
| |  |  |  |  |  |  | | --- | --- | --- | --- | --- | --- | | int Farm::GetFarmNumber | ( | void |  | ) |  | | inline |

References m\_farm\_num.

Referenced by UserDefinedFarm::InvIntPartition(), and CompareFarmNum::operator()().

{ return m\_farm\_num; }

|  |  |  |  |  |  |  |  |
| --- | --- | --- | --- | --- | --- | --- | --- |
| |  |  |  |  |  |  | | --- | --- | --- | --- | --- | --- | | int Farm::GetFirstCropIndex | ( | TTypesOfLandscapeElement | *a\_type* | ) |  | | protectedvirtual |

Gets the first crop for the farm.

This method also synchronises farm rotations either within or between farms if needed. This is useful to try simple what if scenarios.

References g\_farm\_fixed\_rotation\_enable, g\_farm\_fixed\_rotation\_farms\_async, and CfgBool::value().

{

// If g\_farm\_fixed\_rotation, then determine the first

// crop number in the rotation rotation number.

if ( g\_farm\_fixed\_rotation\_enable.value() ) {

if ( !g\_farm\_fixed\_rotation\_farms\_async.value() ) {

// We are running all the farms synchronized, so

// simply set the first crop to run on all farm fields.

return 0;

}

// Each farm runs its fields sync'ed but independently from

// the other farmers.

// Determine if this farm has selected its own start index

// and set it if not. m\_rotation\_sync\_index is initialized

// to -1 by the Farm::Farm() constructor.

if ( -1 == m\_rotation\_sync\_index ) {

m\_rotation\_sync\_index = (int) (rand() % m\_rotation.size());

}

// Return farm localized rotation index.

return m\_rotation\_sync\_index;

}

// Not synchronised, but we want to follow our rotation sequence, so check

// if we have started this process, if not set the sync value.

// afterwards just increment this.

if ( -1 == m\_rotation\_sync\_index ) {

m\_rotation\_sync\_index = (int) (rand() % m\_rotation.size());

}

else m\_rotation\_sync\_index = (int) ((m\_rotation\_sync\_index+1) % m\_rotation.size());

return m\_rotation\_sync\_index;

}

|  |  |  |  |  |  |  |  |
| --- | --- | --- | --- | --- | --- | --- | --- |
| |  |  |  |  |  |  | | --- | --- | --- | --- | --- | --- | | int Farm::GetFirstDate | ( | TTypesOfVegetation | *a\_tov* | ) |  | | protected |

Gets the start date for a crop type.

References tov\_AgroChemIndustryCereal, tov\_Carrots, tov\_CloverGrassGrazed1, tov\_CloverGrassGrazed2, tov\_FieldPeas, tov\_FodderBeet, tov\_FodderGrass, tov\_Maize, tov\_MaizeSilage, tov\_Oats, tov\_OBarleyPeaCloverGrass, tov\_OCarrots, tov\_OCloverGrassGrazed1, tov\_OCloverGrassGrazed2, tov\_OCloverGrassSilage1, tov\_OFieldPeas, tov\_OFirstYearDanger, tov\_OGrazingPigs, tov\_OMaizeSilage, tov\_OOats, tov\_OPermanentGrassGrazed, tov\_OPotatoes, tov\_OSBarleySilage, tov\_OSpringBarley, tov\_OSpringBarleyExt, tov\_OSpringBarleyPigs, tov\_OWinterBarley, tov\_OWinterBarleyExt, tov\_OWinterRape, tov\_OWinterRye, tov\_OWinterWheatUndersown, tov\_OWinterWheatUndersownExt, tov\_PermanentGrassGrazed, tov\_PermanentGrassLowYield, tov\_PermanentGrassTussocky, tov\_PermanentSetaside, tov\_Potatoes, tov\_SeedGrass1, tov\_SeedGrass2, tov\_Setaside, tov\_SpringBarley, tov\_SpringBarleyCloverGrass, tov\_SpringBarleyPTreatment, tov\_SpringBarleySeed, tov\_SpringBarleySilage, tov\_SpringBarleySKManagement, tov\_SpringBarleyStrigling, tov\_SpringBarleyStriglingCulm, tov\_SpringBarleyStriglingSingle, tov\_SpringRape, tov\_Triticale, tov\_WinterBarley, tov\_WinterRape, tov\_WinterRye, tov\_WinterWheat, tov\_WinterWheatStrigling, tov\_WinterWheatStriglingCulm, tov\_WinterWheatStriglingSingle, tov\_WWheatPControl, tov\_WWheatPToxicControl, tov\_WWheatPTreatment, and tov\_YoungForest.

{

switch ( a\_tov2 ) {

case tov\_Carrots:

return m\_carrots->GetFirstDate();

case tov\_FodderGrass:

return m\_foddergrass->GetFirstDate();

case tov\_CloverGrassGrazed1:

return m\_CGG1->GetFirstDate();

case tov\_CloverGrassGrazed2:

return m\_CGG2->GetFirstDate();

case tov\_FieldPeas:

return m\_fieldpeas->GetFirstDate();

case tov\_FodderBeet:

return m\_fodderbeet->GetFirstDate();

case tov\_Maize:

return m\_maize->GetFirstDate();

case tov\_OMaizeSilage:

return m\_omaizesilage->GetFirstDate();

case tov\_MaizeSilage:

return m\_maizesilage->GetFirstDate();

case tov\_OBarleyPeaCloverGrass:

return m\_OBarleyPCG->GetFirstDate();

case tov\_OCarrots:

return m\_ocarrots->GetFirstDate();

case tov\_OCloverGrassGrazed1:

return m\_OCGG1->GetFirstDate();

case tov\_OCloverGrassGrazed2:

return m\_OCGG2->GetFirstDate();

case tov\_OCloverGrassSilage1:

return m\_OCGS1->GetFirstDate();

case tov\_OFieldPeas:

return m\_ofieldpeas->GetFirstDate();

case tov\_OFirstYearDanger:

return m\_ofirstyeardanger->GetFirstDate();

case tov\_OGrazingPigs:

return m\_ograzingpigs->GetFirstDate();

case tov\_OOats:

return m\_ooats->GetFirstDate();

case tov\_Oats:

return m\_oats->GetFirstDate();

case tov\_OPermanentGrassGrazed:

return m\_opermgrassgrazed->GetFirstDate();

case tov\_OPotatoes:

return m\_opotatoes->GetFirstDate();

case tov\_OSpringBarley:

return m\_ospringbarley->GetFirstDate();

case tov\_OSBarleySilage:

return m\_osbarleysilage->GetFirstDate();

case tov\_OSpringBarleyExt:

return m\_ospringbarleyext->GetFirstDate();

case tov\_OSpringBarleyPigs:

return m\_ospringbarleypigs->GetFirstDate();

case tov\_OWinterBarley:

return m\_owinterbarley->GetFirstDate();

case tov\_OWinterBarleyExt:

return m\_owinterbarleyext->GetFirstDate();

case tov\_OWinterRape:

return m\_owinterrape->GetFirstDate();

case tov\_OWinterRye:

return m\_owinterrye->GetFirstDate();

case tov\_OWinterWheatUndersown:

return m\_owinterwheatundersown->GetFirstDate();

case tov\_OWinterWheatUndersownExt:

return m\_owinterwheatundersownext->GetFirstDate();

case tov\_PermanentGrassGrazed:

return m\_permgrassgrazed->GetFirstDate();

case tov\_PermanentGrassLowYield:

return m\_permgrasslowyield->GetFirstDate();

case tov\_PermanentGrassTussocky: // Only used for tole\_PermPastureTussocky

return m\_permgrasstussocky->GetFirstDate();

case tov\_PermanentSetaside:

return m\_permanentsetaside->GetFirstDate();

case tov\_Potatoes:

return m\_potatoes->GetFirstDate();

case tov\_SeedGrass1:

return m\_seedgrass1->GetFirstDate();

case tov\_SeedGrass2:

return m\_seedgrass2->GetFirstDate();

case tov\_Setaside:

return m\_setaside->GetFirstDate();

case tov\_SpringBarley:

return m\_springbarley->GetFirstDate();

case tov\_SpringBarleyPTreatment:

return m\_springbarleyptreatment->GetFirstDate();

case tov\_SpringBarleySKManagement:

return m\_springbarleyskmanagement->GetFirstDate();

case tov\_SpringBarleyCloverGrass:

return m\_sbarleyclovergrass->GetFirstDate();

case tov\_SpringBarleySeed:

return m\_springbarleyseed->GetFirstDate();

case tov\_SpringBarleySilage:

return m\_springbarleysilage->GetFirstDate();

case tov\_SpringBarleyStrigling:

return m\_springbarleystrigling->GetFirstDate();

case tov\_SpringBarleyStriglingSingle:

return m\_springbarleystriglingsingle->GetFirstDate();

case tov\_SpringBarleyStriglingCulm:

return m\_springbarleystriglingculm->GetFirstDate();

case tov\_SpringRape:

return m\_springrape->GetFirstDate();

case tov\_Triticale:

return m\_triticale->GetFirstDate();

case tov\_WinterBarley:

return m\_winterbarley->GetFirstDate();

case tov\_WinterRape:

return m\_winterrape->GetFirstDate();

case tov\_WinterRye:

return m\_winterrye->GetFirstDate();

case tov\_WinterWheat:

return m\_winterwheat->GetFirstDate();

case tov\_WinterWheatStrigling:

return m\_winterwheatstrigling->GetFirstDate();

case tov\_WinterWheatStriglingSingle:

return m\_winterwheatstriglingsingle->GetFirstDate();

case tov\_WinterWheatStriglingCulm:

return m\_winterwheatstriglingculm->GetFirstDate();

case tov\_WWheatPControl:

return m\_wwheatpcontrol->GetFirstDate();

case tov\_WWheatPToxicControl:

return m\_wwheatptoxiccontrol->GetFirstDate();

case tov\_WWheatPTreatment:

return m\_wwheatptreatment->GetFirstDate();

case tov\_AgroChemIndustryCereal:

return m\_agrochemindustrycereal->GetFirstDate();

case tov\_YoungForest:

return m\_youngforest->GetFirstDate();

default:

return 0;

}

}

|  |  |  |  |  |  |  |  |
| --- | --- | --- | --- | --- | --- | --- | --- |
| |  |  |  |  |  |  | | --- | --- | --- | --- | --- | --- | | int Farm::GetIntensity | ( | void |  | ) |  | | inline |

References m\_intensity.

{ return m\_intensity; }

|  |  |  |  |  |  |  |  |
| --- | --- | --- | --- | --- | --- | --- | --- |
| |  |  |  |  |  |  | | --- | --- | --- | --- | --- | --- | | int Farm::GetNextCropIndex | ( | int | *a\_rot\_index* | ) |  | | protectedvirtual |

Returns the next crop in the rotation.

Also provides the possibility of over-riding rotations using configuration settings

References g\_farm\_enable\_crop\_rotation, and CfgBool::value().

{

if ( !g\_farm\_enable\_crop\_rotation.value() ) {

// Rotation not enabled.

return a\_rot\_index;

}

if ( a\_rot\_index == -1 )

return -1;

if ( ( unsigned int ) ( ++a\_rot\_index ) == m\_rotation.size() )

a\_rot\_index = 0;

return a\_rot\_index;

}

|  |  |  |  |  |  |  |  |  |  |  |  |  |  |
| --- | --- | --- | --- | --- | --- | --- | --- | --- | --- | --- | --- | --- | --- |
| |  |  |  |  | | --- | --- | --- | --- | | int Farm::GetNextCropStartDate | ( | LE \* | *a\_field*, | |  |  | TTypesOfVegetation & | *a\_curr\_veg* | |  | ) |  |  | | protected |

Returns the start date of the next crop in the rotation.

References g\_farm\_fixed\_crop\_enable, LE::GetRotIndex(), and CfgBool::value().

{

TTypesOfVegetation l\_tov2;

if ( a\_field->GetRotIndex() < 0 || g\_farm\_fixed\_crop\_enable.value() //|| g\_farm\_test\_crop.value()

) {

l\_tov2 = a\_curr\_veg; // don't do it if no rotation

} else {

l\_tov2 = m\_rotation[ GetNextCropIndex( a\_field->GetRotIndex() ) ];

}

a\_curr\_veg = l\_tov2;

return GetFirstDate( l\_tov2 );

}

|  |  |  |  |  |  |  |  |
| --- | --- | --- | --- | --- | --- | --- | --- |
| |  |  |  |  |  |  | | --- | --- | --- | --- | --- | --- | | TTypesOfFarm Farm::GetType | ( | void |  | ) |  | | inline |

References m\_farmtype.

{ return m\_farmtype; }

|  |  |  |  |  |  |  |  |  |  |  |  |  |  |  |  |  |  |
| --- | --- | --- | --- | --- | --- | --- | --- | --- | --- | --- | --- | --- | --- | --- | --- | --- | --- |
| |  |  |  |  | | --- | --- | --- | --- | | bool Farm::Glyphosate | ( | LE \* | *a\_field*, | |  |  | double | *a\_user*, | |  |  | int | *a\_days* | |  | ) |  |  | | virtual |

References glyphosate, LE::InsectMortality(), LE::ReduceVeg\_Extended(), LE::SetLastTreatment(), and LE::Trace().

{

// Will always do this at the first chance

a\_field->Trace( glyphosate );

a\_field->SetLastTreatment( glyphosate );

a\_field->InsectMortality( 0.5 );

a\_field->ReduceVeg\_Extended( 0.05 );

return true;

}

|  |  |  |  |  |  |  |  |  |  |  |  |  |  |  |  |  |  |
| --- | --- | --- | --- | --- | --- | --- | --- | --- | --- | --- | --- | --- | --- | --- | --- | --- | --- |
| |  |  |  |  | | --- | --- | --- | --- | | bool Farm::GrowthRegulator | ( | LE \* | *a\_field*, | |  |  | double | *a\_user*, | |  |  | int | *a\_days* | |  | ) |  |  | | virtual |

Apply growth regulator to a\_field.

References DO\_IT\_PROB, EL\_TRAMLINE\_DECAYTIME, g\_weather, LE::GetSignal(), Weather::GetWind(), growth\_regulator, LE\_SIG\_NO\_GROWTH\_REG, Weather::Raining(), LE::SetLastTreatment(), LE::SetTramlinesDecay(), and LE::Trace().

{

if (0 >= a\_days)

{

if ( (!g\_weather->Raining()) && (g\_weather->GetWind()<4.5) &&

(!(a\_field->GetSignal() & LE\_SIG\_NO\_GROWTH\_REG) )) {

a\_field->Trace( growth\_regulator );

a\_field->SetLastTreatment( growth\_regulator );

a\_field->SetTramlinesDecay( EL\_TRAMLINE\_DECAYTIME );

}

return true;

}

else if ( (g\_weather->GetWind()<4.5) &&

(!g\_weather->Raining()) && DoIt(DO\_IT\_PROB)) {

if ( ! (a\_field->GetSignal() & LE\_SIG\_NO\_GROWTH\_REG) ) {

a\_field->Trace( growth\_regulator );

a\_field->SetLastTreatment( growth\_regulator );

a\_field->SetTramlinesDecay( EL\_TRAMLINE\_DECAYTIME );

}

return true;

}

return false;

}

|  |  |  |  |  |  |  |  |
| --- | --- | --- | --- | --- | --- | --- | --- |
| |  |  |  |  |  |  | | --- | --- | --- | --- | --- | --- | | void Farm::HandleEvents | ( | void |  | ) |  | | protected |

If there are events to carry out do this, and perhaps start a new crop.

References LE::BumpRunNum(), Calendar::Date(), LE::ForceGrowthTest(), g\_date, g\_farm\_fixed\_crop\_enable, g\_farm\_fixed\_crop\_type, g\_letype, LE::GetRotIndex(), LE::GetRunNum(), LE::GetVegType(), LowPriPair< ELEMTYPE >::m\_element, FarmEvent::m\_field, LowPriPair< ELEMTYPE >::m\_pri, PROG\_START, LE::ResetTrace(), LE::SetMgtLoopDetectDate(), LE::SetRotIndex(), LE::SetVegStore(), LE::SetVegType(), tov\_Undefined, LE\_TypeClass::TranslateVegTypes(), CfgInt::value(), and CfgBool::value().

{

if ( m\_queue.Empty() )

return;

LowPriPair < FarmEvent \* > pair = m\_queue.Bottom();

FarmEvent \* ev = pair.m\_element;

while ( pair.m\_pri <= g\_date->Date() ) {

m\_queue.Pop();

if ( LeSwitch( ev ) ) {

// This crop management plan has terminated.

// First check for an infinite loop in the rotation scheme,

// ie. a scenario where all crops decide not to run given

// the date.

CheckRotationManagementLoop( ev );

// Outdate any remaining events for this field.

ev->m\_field->BumpRunNum();

// Crop treatment done, select and initiate new crop if in rotation.

TTypesOfVegetation new\_veg = ev->m\_field->GetVegType();

if ( ev->m\_field->GetRotIndex() >= 0 ) {

int new\_index = GetNextCropIndex( ev->m\_field->GetRotIndex() );

new\_veg = m\_rotation[ new\_index ];

// Running in fixed crop mode?

if ( g\_farm\_fixed\_crop\_enable.value() ) {

new\_veg = g\_letype->TranslateVegTypes( g\_farm\_fixed\_crop\_type.value() );

}

/\*

if ( g\_farm\_test\_crop.value() ) {

new\_veg = g\_letype->TranslateVegTypes( g\_farm\_test\_crop\_type.value() );

}

\*/

ev->m\_field->SetRotIndex( new\_index );

ev->m\_field->SetVegType( new\_veg, tov\_Undefined );

ev->m\_field->ForceGrowthTest();

}

// Reset the event list for this field.

ev->m\_field->ResetTrace();

// Reset event timeout counter.

ev->m\_field->SetVegStore( 0 );

// The next bit simply determines the start date of the next crop in

// the rotation and passes this to the start crop event.

// The crop is responsible for raising an error if the next crop is

// not possible or otherwise handling the problem

// 19/5-2003: Note: This code was moved out into a dedicated

// method of the Farm class, GetNextCropStartDate(), as precisely

// the same piece of code needs to be run during initialization of

// farm management.

TTypesOfVegetation l\_tov = new\_veg;

int l\_nextcropstartdate = GetNextCropStartDate( ev->m\_field, l\_tov );

// Create 'start' event for today and put it on the queue.

AddNewEvent( new\_veg, g\_date->Date(), ev->m\_field, PROG\_START, ev->m\_field->GetRunNum(),

false, l\_nextcropstartdate, false, l\_tov );

// Set starting date for rotation mgmt loop detection.

ev->m\_field->SetMgtLoopDetectDate( g\_date->Date() );

}

delete ev;

if ( m\_queue.Empty() )

return;

pair = m\_queue.Bottom();

ev = pair.m\_element;

}

}

|  |  |  |  |  |  |  |  |  |  |  |  |  |  |  |  |  |  |
| --- | --- | --- | --- | --- | --- | --- | --- | --- | --- | --- | --- | --- | --- | --- | --- | --- | --- |
| |  |  |  |  | | --- | --- | --- | --- | | bool Farm::Harvest | ( | LE \* | *a\_field*, | |  |  | double | *a\_user*, | |  |  | int | *a\_days* | |  | ) |  |  | | virtual |

Carry out a harvest on a\_field.

References Calendar::Date(), DO\_IT\_PROB, EL\_TRAMLINE\_DECAYTIME, g\_date, g\_landscape\_p, g\_weather, LE::GetInsectPop(), Weather::GetRainPeriod(), LE::GetUnsprayedMarginPolyRef(), harvest, harvest1, LE::InsectMortality(), LE::RecalculateBugsNStuff(), LE::SetGrowthPhase(), LE::SetInsectPop(), LE::SetLastTreatment(), LE::SetTramlinesDecay(), Landscape::SupplyLEPointer(), and LE::Trace().

{

//5 days good weather before

if ( (0 >= a\_days) ||

((g\_weather->GetRainPeriod(g\_date->Date(),5)<0.1) && DoIt(DO\_IT\_PROB))

) {

a\_field->Trace( harvest );

a\_field->SetLastTreatment( harvest );

a\_field->SetGrowthPhase( harvest1 );

// Here we have to do a little skip to avoid too low insect populations after harvest, but a correct veg biomass

a\_field->InsectMortality( 0.4 );

double insects=a\_field->GetInsectPop();

a\_field->RecalculateBugsNStuff();

a\_field->SetInsectPop(insects);

a\_field->SetTramlinesDecay( EL\_TRAMLINE\_DECAYTIME );

int pref=a\_field->GetUnsprayedMarginPolyRef();

if (pref!=-1){

// Must have an unsprayed margin so need to pass the information on to it

LE\* um=g\_landscape\_p->SupplyLEPointer(pref);

um->SetLastTreatment(harvest);

um->SetGrowthPhase( harvest1 );

um->InsectMortality( 0.4 );

um->SetTramlinesDecay( EL\_TRAMLINE\_DECAYTIME );

}

return true;

}

return false;

}

|  |  |  |  |  |  |  |  |  |  |  |  |  |  |  |  |  |  |
| --- | --- | --- | --- | --- | --- | --- | --- | --- | --- | --- | --- | --- | --- | --- | --- | --- | --- |
| |  |  |  |  | | --- | --- | --- | --- | | bool Farm::HayBailing | ( | LE \* | *a\_field*, | |  |  | double | *a\_user*, | |  |  | int | *a\_days* | |  | ) |  |  | | virtual |

Carry out hay bailing on a\_field.

References Calendar::Date(), DO\_IT\_PROB, EL\_TRAMLINE\_DECAYTIME, g\_date, g\_landscape\_p, g\_weather, Weather::GetRainPeriod(), LE::GetUnsprayedMarginPolyRef(), hay\_bailing, LE::SetLastTreatment(), LE::SetTramlinesDecay(), Landscape::SupplyLEPointer(), and LE::Trace().

{

if ( (0 >= a\_days) ||

((g\_weather->GetRainPeriod(g\_date->Date(),5)<0.1) && DoIt(DO\_IT\_PROB))

) {

a\_field->Trace( hay\_bailing );

a\_field->SetLastTreatment( hay\_bailing );

a\_field->SetTramlinesDecay( EL\_TRAMLINE\_DECAYTIME );

int pref=a\_field->GetUnsprayedMarginPolyRef();

if (pref!=-1){

// Must have an unsprayed margin so need to pass the information on to it

LE\* um=g\_landscape\_p->SupplyLEPointer(pref);

um->SetLastTreatment( hay\_bailing );

um->SetTramlinesDecay( EL\_TRAMLINE\_DECAYTIME );

}

return true;

}

return false;

}

|  |  |  |  |  |  |  |  |  |  |  |  |  |  |  |  |  |  |
| --- | --- | --- | --- | --- | --- | --- | --- | --- | --- | --- | --- | --- | --- | --- | --- | --- | --- |
| |  |  |  |  | | --- | --- | --- | --- | | bool Farm::HayTurning | ( | LE \* | *a\_field*, | |  |  | double | *a\_user*, | |  |  | int | *a\_days* | |  | ) |  |  | | virtual |

Carry out hay turning on a\_field.

References Calendar::Date(), DO\_IT\_PROB, EL\_TRAMLINE\_DECAYTIME, g\_date, g\_landscape\_p, g\_weather, Weather::GetRainPeriod(), LE::GetUnsprayedMarginPolyRef(), hay\_turning, LE::SetLastTreatment(), LE::SetTramlinesDecay(), Landscape::SupplyLEPointer(), and LE::Trace().

{

if ( (0 >= a\_days) ||

((g\_weather->GetRainPeriod(g\_date->Date(),5)<0.1) && DoIt(DO\_IT\_PROB))

) {

a\_field->Trace( hay\_turning );

a\_field->SetLastTreatment( hay\_turning );

a\_field->SetTramlinesDecay( EL\_TRAMLINE\_DECAYTIME );

int pref=a\_field->GetUnsprayedMarginPolyRef();

if (pref!=-1){

// Must have an unsprayed margin so need to pass the information on to it

LE\* um=g\_landscape\_p->SupplyLEPointer(pref);

um->SetLastTreatment( hay\_turning );

um->SetTramlinesDecay( EL\_TRAMLINE\_DECAYTIME );

}

return true;

}

return false;

}

|  |  |  |  |  |  |  |  |  |  |  |  |  |  |  |  |  |  |
| --- | --- | --- | --- | --- | --- | --- | --- | --- | --- | --- | --- | --- | --- | --- | --- | --- | --- |
| |  |  |  |  | | --- | --- | --- | --- | | bool Farm::HerbicideTreat | ( | LE \* | *a\_field*, | |  |  | double | *a\_user*, | |  |  | int | *a\_days* | |  | ) |  |  | | virtual |

Apply herbicide to a\_field.

References DO\_IT\_PROB, EL\_HERBICIDE\_DELAYTIME, EL\_TRAMLINE\_DECAYTIME, g\_weather, LE::GetSignal(), Weather::GetWind(), herbicide\_treat, l\_farm\_herbicide\_kills, LE\_SIG\_NO\_HERBICIDE, Weather::Raining(), LE::ReduceWeedBiomass(), LE::SetHerbicideDelay(), LE::SetLastTreatment(), LE::SetTramlinesDecay(), LE::Trace(), and CfgBool::value().

{

if (0 >= a\_days) {

if ( (!g\_weather->Raining() && (g\_weather->GetWind()<4.5)) && ((a\_field->GetSignal() & LE\_SIG\_NO\_HERBICIDE)==0 )) {

a\_field->Trace( herbicide\_treat );

a\_field->SetLastTreatment( herbicide\_treat );

if ( l\_farm\_herbicide\_kills.value()) {

a\_field->ReduceWeedBiomass( 0.05 );

}

a\_field->SetTramlinesDecay( EL\_TRAMLINE\_DECAYTIME );

a\_field->SetHerbicideDelay( EL\_HERBICIDE\_DELAYTIME );

}

a\_field->SetTramlinesDecay( EL\_TRAMLINE\_DECAYTIME );

return true;

}

else if ((g\_weather->GetWind()<4.5) &&

(!g\_weather->Raining()) && DoIt(DO\_IT\_PROB)) {

if ( !(a\_field->GetSignal() & LE\_SIG\_NO\_HERBICIDE) ) {

a\_field->Trace( herbicide\_treat );

a\_field->SetLastTreatment( herbicide\_treat );

if ( l\_farm\_herbicide\_kills.value()) {

a\_field->ReduceWeedBiomass( 0.05 );

}

a\_field->SetTramlinesDecay( EL\_TRAMLINE\_DECAYTIME );

a\_field->SetHerbicideDelay( EL\_HERBICIDE\_DELAYTIME );

}

return true;

}

return false;

}

|  |  |  |  |  |  |  |  |  |  |  |  |  |  |  |  |  |  |
| --- | --- | --- | --- | --- | --- | --- | --- | --- | --- | --- | --- | --- | --- | --- | --- | --- | --- |
| |  |  |  |  | | --- | --- | --- | --- | | bool Farm::HillingUp | ( | LE \* | *a\_field*, | |  |  | double | *a\_user*, | |  |  | int | *a\_days* | |  | ) |  |  | | virtual |

Do hilling up on a\_field, probably of potatoes.

References DO\_IT\_PROB, EL\_TRAMLINE\_DECAYTIME, g\_landscape\_p, g\_weather, LE::GetUnsprayedMarginPolyRef(), hilling\_up, LE::InsectMortality(), Weather::Raining(), LE::ReduceWeedBiomass(), LE::SetLastTreatment(), LE::SetTramlinesDecay(), Landscape::SupplyLEPointer(), and LE::Trace().

{

if ( (0 >= a\_days) || (!g\_weather->Raining() && DoIt(DO\_IT\_PROB))) {

a\_field->Trace( hilling\_up );

a\_field->SetLastTreatment( hilling\_up );

a\_field->InsectMortality( 0.75 );

a\_field->ReduceWeedBiomass( 0.25 );

a\_field->SetTramlinesDecay( EL\_TRAMLINE\_DECAYTIME );

int pref=a\_field->GetUnsprayedMarginPolyRef();

if (pref!=-1){

// Must have an unsprayed margin so need to pass the information on to it

LE\* um=g\_landscape\_p->SupplyLEPointer(pref);

um->SetLastTreatment( hilling\_up );

um->ReduceWeedBiomass( 0.25 );

um->InsectMortality( 0.75 );

um->SetTramlinesDecay( EL\_TRAMLINE\_DECAYTIME );

}

return true;

}

return false;

}

|  |  |  |  |  |  |  |  |
| --- | --- | --- | --- | --- | --- | --- | --- |
| |  |  |  |  |  |  | | --- | --- | --- | --- | --- | --- | | void Farm::InitiateManagement | ( | void |  | ) |  | | virtual |

Kicks off the farm's management.

Reimplemented in UserDefinedFarm36, and UserDefinedFarm.

References Calendar::Date(), g\_date, g\_farm\_fixed\_crop\_enable, g\_farm\_fixed\_crop\_type, g\_letype, g\_msg, janfirst, PROG\_START, tole\_PermanentSetaside, tole\_PermPasture, tole\_PermPastureLowYield, tole\_PermPastureTussocky, tole\_YoungForest, tov\_PermanentGrassGrazed, tov\_PermanentGrassLowYield, tov\_PermanentGrassTussocky, tov\_PermanentSetaside, tov\_Undefined, tov\_YoungForest, LE\_TypeClass::TranslateVegTypes(), CfgInt::value(), CfgBool::value(), and MapErrorMsg::Warn().

Referenced by UserDefinedFarm::InitiateManagement().

{

for ( unsigned int i = 0; i < m\_fields.size(); i++ ) {

int rot\_index = m\_fields[ i ]->GetRotIndex();

TTypesOfVegetation new\_veg = tov\_Undefined;

// If the field has been designated as non-rotating and therefore already has its veg type, then skip it.

if ( rot\_index < -1 ) {

// Check for any type of permanent element type with management plan.

TTypesOfLandscapeElement ele = m\_fields[ i ]->GetElementType();

switch (ele) {

case tole\_PermPastureLowYield:

new\_veg = tov\_PermanentGrassLowYield;

break;

case tole\_YoungForest:

new\_veg = tov\_YoungForest;

break;

case tole\_PermPasture:

new\_veg = tov\_PermanentGrassGrazed;

break;

case tole\_PermPastureTussocky:

new\_veg = tov\_PermanentGrassTussocky;

break;

case tole\_PermanentSetaside:

new\_veg = tov\_PermanentSetaside;

break;

default:

if (rot\_index != -4) {

// Error

g\_msg->Warn("Unexpected negative value in Farm::InitiateManagement","");

exit(0);

} else new\_veg = m\_fields[ i ]->GetVegType();

}

}

else {

rot\_index = GetFirstCropIndex( m\_fields[ i ]->GetElementType() );

new\_veg = m\_rotation[ rot\_index ];

}

// Running in fixed crop mode?

if ( g\_farm\_fixed\_crop\_enable.value() ) {

int fv = g\_farm\_fixed\_crop\_type.value();

new\_veg = g\_letype->TranslateVegTypes( fv );

}

m\_fields[ i ]->SetVegType( new\_veg, tov\_Undefined );

m\_fields[ i ]->SetRotIndex( rot\_index );

m\_fields[ i ]->SetGrowthPhase( janfirst );

// Reset event timeout counter. We are now 800 days from

// oblivion.

long prog\_start\_date = g\_date->Date();

m\_fields[ i ]->SetVegStore( 0 );

TTypesOfVegetation l\_tov = new\_veg;

int l\_nextcropstartdate = GetNextCropStartDate( m\_fields[ i ], l\_tov );

AddNewEvent( new\_veg, prog\_start\_date, m\_fields[ i ], PROG\_START, 0, false, l\_nextcropstartdate, true, l\_tov );

}

}

|  |  |  |  |  |  |  |  |  |  |  |  |  |  |  |  |  |  |
| --- | --- | --- | --- | --- | --- | --- | --- | --- | --- | --- | --- | --- | --- | --- | --- | --- | --- |
| |  |  |  |  | | --- | --- | --- | --- | | bool Farm::InsecticideTreat | ( | LE \* | *a\_field*, | |  |  | double | *a\_user*, | |  |  | int | *a\_days* | |  | ) |  |  | | virtual |

Apply insecticide to a\_field.

References DO\_IT\_PROB, EL\_TRAMLINE\_DECAYTIME, g\_weather, LE::GetSignal(), Weather::GetWind(), LE::Insecticide(), insecticide\_treat, l\_farm\_insecticide\_kills, LE\_SIG\_NO\_INSECTICIDE, Weather::Raining(), LE::SetLastTreatment(), LE::SetTramlinesDecay(), LE::Trace(), and CfgBool::value().

{

if (0 >= a\_days) {

if ( (!g\_weather->Raining()) && (g\_weather->GetWind()<4.5) && ( ! (a\_field->GetSignal() & LE\_SIG\_NO\_INSECTICIDE) )) {

// \*\*CJT\*\* Turn this code on to use the pesticide engine with insecticides

// g\_pest->DailyQueueAdd( a\_field, l\_pest\_insecticide\_amount.value());

//

a\_field->Trace( insecticide\_treat );

a\_field->SetLastTreatment( insecticide\_treat );

if ( l\_farm\_insecticide\_kills.value()) {

a\_field->Insecticide( 0.36 );

}

a\_field->SetTramlinesDecay( EL\_TRAMLINE\_DECAYTIME );

}

return true;

}

else if ( (g\_weather->GetWind()<4.5) &&

(!g\_weather->Raining()) && DoIt(DO\_IT\_PROB)) {

if ( ! (a\_field->GetSignal() & LE\_SIG\_NO\_INSECTICIDE )) {

// \*\*CJT\*\* Turn this code on to use the pesticide engine with insecticides

// g\_pest->DailyQueueAdd( a\_field, l\_pest\_insecticide\_amount.value());

//

a\_field->Trace( insecticide\_treat );

a\_field->SetLastTreatment( insecticide\_treat );

if ( l\_farm\_insecticide\_kills.value()) {

a\_field->Insecticide( 0.36 );

}

a\_field->SetTramlinesDecay( EL\_TRAMLINE\_DECAYTIME );

}

return true;

}

return false;

}

|  |  |  |  |  |  |  |  |
| --- | --- | --- | --- | --- | --- | --- | --- |
| |  |  |  |  |  |  | | --- | --- | --- | --- | --- | --- | | bool Farm::IsStockFarmer | ( | void |  | ) |  | | inline |

References m\_stockfarmer.

{ return m\_stockfarmer; }

|  |  |  |  |  |  |  |  |
| --- | --- | --- | --- | --- | --- | --- | --- |
| |  |  |  |  |  |  | | --- | --- | --- | --- | --- | --- | | bool Farm::LeSwitch | ( | FarmEvent \* | *ev* | ) |  | | protected |

Call do function for any crop with an outstanding event. Signal if the crop has terminated.

References g\_msg, LE::GetRunNum(), FarmEvent::m\_event, FarmEvent::m\_field, FarmEvent::m\_run, FarmEvent::m\_todo, LE::m\_tried\_to\_do, tov\_AgroChemIndustryCereal, tov\_Carrots, tov\_CloverGrassGrazed1, tov\_CloverGrassGrazed2, tov\_FieldPeas, tov\_FieldPeasStrigling, tov\_FodderBeet, tov\_FodderGrass, tov\_Maize, tov\_MaizeSilage, tov\_MaizeStrigling, tov\_Oats, tov\_OBarleyPeaCloverGrass, tov\_OCarrots, tov\_OCloverGrassGrazed1, tov\_OCloverGrassGrazed2, tov\_OCloverGrassSilage1, tov\_OFieldPeas, tov\_OFieldPeasSilage, tov\_OGrazingPigs, tov\_OMaizeSilage, tov\_OOats, tov\_OPermanentGrassGrazed, tov\_OPotatoes, tov\_OSBarleySilage, tov\_OSpringBarley, tov\_OSpringBarleyExt, tov\_OSpringBarleyPigs, tov\_OWinterBarley, tov\_OWinterBarleyExt, tov\_OWinterRape, tov\_OWinterRye, tov\_OWinterWheatUndersown, tov\_OWinterWheatUndersownExt, tov\_PermanentGrassGrazed, tov\_PermanentGrassLowYield, tov\_PermanentGrassTussocky, tov\_PermanentSetaside, tov\_Potatoes, tov\_PotatoesIndustry, tov\_SeedGrass1, tov\_SeedGrass2, tov\_Setaside, tov\_SpringBarley, tov\_SpringBarleyCloverGrass, tov\_SpringBarleyCloverGrassStrigling, tov\_SpringBarleyPeaCloverGrassStrigling, tov\_SpringBarleyPTreatment, tov\_SpringBarleySeed, tov\_SpringBarleySilage, tov\_SpringBarleySKManagement, tov\_SpringBarleyStrigling, tov\_SpringBarleyStriglingCulm, tov\_SpringBarleyStriglingSingle, tov\_SpringRape, tov\_Triticale, tov\_WinterBarley, tov\_WinterBarleyStrigling, tov\_WinterRape, tov\_WinterRapeStrigling, tov\_WinterRye, tov\_WinterRyeStrigling, tov\_WinterWheat, tov\_WinterWheatStrigling, tov\_WinterWheatStriglingCulm, tov\_WinterWheatStriglingSingle, tov\_WWheatPControl, tov\_WWheatPToxicControl, tov\_WWheatPTreatment, tov\_YoungForest, MapErrorMsg::Warn(), and WARN\_BUG.

{

// Ignore this event if it is from the execution of

// a previous management plan.

if ( ev->m\_field->GetRunNum() > ev->m\_run )

return false;

// Store what we are trying to do this time.

// \*\*\*FN\*\*\* To be cleaned up later.

ev->m\_field->m\_tried\_to\_do = ev->m\_todo;

bool done;

switch ( ev->m\_event ) {

case tov\_Carrots:

done = m\_carrots->Do( this, ev->m\_field, ev );

break;

case tov\_FodderGrass:

done = m\_foddergrass->Do( this, ev->m\_field, ev );

break;

case tov\_CloverGrassGrazed1:

done = m\_CGG1->Do( this, ev->m\_field, ev );

break;

case tov\_CloverGrassGrazed2:

done = m\_CGG2->Do( this, ev->m\_field, ev );

break;

case tov\_FieldPeas:

done = m\_fieldpeas->Do( this, ev->m\_field, ev );

break;

case tov\_FodderBeet:

done = m\_fodderbeet->Do( this, ev->m\_field, ev );

break;

case tov\_Maize:

done = m\_maize->Do( this, ev->m\_field, ev );

break;

case tov\_OBarleyPeaCloverGrass:

done = m\_OBarleyPCG->Do( this, ev->m\_field, ev );

break;

case tov\_OCarrots:

done = m\_ocarrots->Do( this, ev->m\_field, ev );

break;

case tov\_OCloverGrassSilage1:

done = m\_OCGS1->Do( this, ev->m\_field, ev );

break;

case tov\_OCloverGrassGrazed1:

done = m\_OCGG1->Do( this, ev->m\_field, ev );

break;

case tov\_OCloverGrassGrazed2:

done = m\_OCGG2->Do( this, ev->m\_field, ev );

break;

case tov\_OFieldPeas:

done = m\_ofieldpeas->Do( this, ev->m\_field, ev );

break;

case tov\_OFieldPeasSilage:

done = m\_ofieldpeassilage->Do( this, ev->m\_field, ev );

break;

case tov\_OGrazingPigs:

done = m\_ograzingpigs->Do( this, ev->m\_field, ev );

break;

case tov\_OOats:

done = m\_ooats->Do( this, ev->m\_field, ev );

break;

case tov\_Oats:

done = m\_oats->Do( this, ev->m\_field, ev );

break;

case tov\_OPermanentGrassGrazed:

done = m\_opermgrassgrazed->Do( this, ev->m\_field, ev );

break;

case tov\_OPotatoes:

done = m\_opotatoes->Do( this, ev->m\_field, ev );

break;

case tov\_OSpringBarley:

done = m\_ospringbarley->Do( this, ev->m\_field, ev );

break;

case tov\_OSBarleySilage:

done = m\_osbarleysilage->Do( this, ev->m\_field, ev );

break;

case tov\_OSpringBarleyExt:

done = m\_ospringbarleyext->Do( this, ev->m\_field, ev );

break;

case tov\_OSpringBarleyPigs:

done = m\_ospringbarleypigs->Do( this, ev->m\_field, ev );

break;

case tov\_OWinterBarley:

done = m\_owinterbarley->Do( this, ev->m\_field, ev );

break;

case tov\_OWinterBarleyExt:

done = m\_owinterbarleyext->Do( this, ev->m\_field, ev );

break;

case tov\_OWinterWheatUndersown:

done = m\_owinterwheatundersown->Do( this, ev->m\_field, ev );

break;

case tov\_OWinterWheatUndersownExt:

done = m\_owinterwheatundersownext->Do( this, ev->m\_field, ev );

break;

case tov\_OWinterRape:

done = m\_owinterrape->Do( this, ev->m\_field, ev );

break;

case tov\_OWinterRye:

done = m\_owinterrye->Do( this, ev->m\_field, ev );

break;

case tov\_PermanentGrassGrazed:

done = m\_permgrassgrazed->Do( this, ev->m\_field, ev );

break;

case tov\_PermanentGrassLowYield:

done = m\_permgrasslowyield->Do( this, ev->m\_field, ev );

break;

case tov\_PermanentGrassTussocky:

done = m\_permgrasstussocky->Do( this, ev->m\_field, ev );

break;

case tov\_PermanentSetaside:

done = m\_permanentsetaside->Do( this, ev->m\_field, ev );

break;

case tov\_Potatoes:

done = m\_potatoes->Do( this, ev->m\_field, ev );

break;

case tov\_PotatoesIndustry:

done = m\_potatoesindustry->Do( this, ev->m\_field, ev );

break;

case tov\_SeedGrass1:

done = m\_seedgrass1->Do( this, ev->m\_field, ev );

break;

case tov\_SeedGrass2:

done = m\_seedgrass2->Do( this, ev->m\_field, ev );

break;

case tov\_Setaside:

done = m\_setaside->Do( this, ev->m\_field, ev );

break;

case tov\_SpringBarley:

done = m\_springbarley->Do( this, ev->m\_field, ev );

break;

case tov\_SpringBarleyPTreatment:

done = m\_springbarleyptreatment->Do( this, ev->m\_field, ev );

break;

case tov\_SpringBarleySKManagement:

done = m\_springbarleyskmanagement->Do( this, ev->m\_field, ev );

break;

case tov\_SpringBarleyCloverGrass:

done = m\_sbarleyclovergrass->Do( this, ev->m\_field, ev );

break;

case tov\_SpringBarleySeed:

done = m\_springbarleyseed->Do( this, ev->m\_field, ev );

break;

case tov\_SpringBarleySilage:

done = m\_springbarleysilage->Do( this, ev->m\_field, ev );

break;

case tov\_SpringRape:

done = m\_springrape->Do( this, ev->m\_field, ev );

break;

case tov\_Triticale:

done = m\_triticale->Do( this, ev->m\_field, ev );

break;

case tov\_WinterBarley:

done = m\_winterbarley->Do( this, ev->m\_field, ev );

break;

case tov\_WinterRape:

done = m\_winterrape->Do( this, ev->m\_field, ev );

break;

case tov\_WinterRye:

done = m\_winterrye->Do( this, ev->m\_field, ev );

break;

case tov\_WinterWheat:

done = m\_winterwheat->Do( this, ev->m\_field, ev );

break;

case tov\_WWheatPControl:

done = m\_wwheatpcontrol->Do( this, ev->m\_field, ev );

break;

case tov\_WWheatPToxicControl:

done = m\_wwheatptoxiccontrol->Do( this, ev->m\_field, ev );

break;

case tov\_WWheatPTreatment:

done = m\_wwheatptreatment->Do( this, ev->m\_field, ev );

break;

case tov\_AgroChemIndustryCereal:

done = m\_agrochemindustrycereal->Do( this, ev->m\_field, ev );

break;

case tov\_WinterWheatStrigling:

done = m\_winterwheatstrigling->Do( this, ev->m\_field, ev );

break;

case tov\_WinterWheatStriglingSingle:

done = m\_winterwheatstriglingsingle->Do( this, ev->m\_field, ev );

break;

case tov\_WinterWheatStriglingCulm:

done = m\_winterwheatstriglingculm->Do( this, ev->m\_field, ev );

break;

case tov\_SpringBarleyCloverGrassStrigling:

done = m\_springbarleyclovergrassstrigling->Do( this, ev->m\_field, ev );

break;

case tov\_SpringBarleyStrigling:

done = m\_springbarleystrigling->Do( this, ev->m\_field, ev );

break;

case tov\_SpringBarleyStriglingSingle:

done = m\_springbarleystriglingsingle->Do( this, ev->m\_field, ev );

break;

case tov\_SpringBarleyStriglingCulm:

done = m\_springbarleystriglingculm->Do( this, ev->m\_field, ev );

break;

case tov\_MaizeStrigling:

done = m\_maizestrigling->Do( this, ev->m\_field, ev );

break;

case tov\_MaizeSilage:

done = m\_maizesilage->Do( this, ev->m\_field, ev );

break;

case tov\_OMaizeSilage:

done = m\_omaizesilage->Do( this, ev->m\_field, ev );

break;

case tov\_WinterRapeStrigling:

done = m\_winterrapestrigling->Do( this, ev->m\_field, ev );

break;

case tov\_WinterRyeStrigling:

done = m\_winterryestrigling->Do( this, ev->m\_field, ev );

break;

case tov\_WinterBarleyStrigling:

done = m\_winterbarleystrigling->Do( this, ev->m\_field, ev );

break;

case tov\_FieldPeasStrigling:

done = m\_fieldpeasstrigling->Do( this, ev->m\_field, ev );

break;

case tov\_SpringBarleyPeaCloverGrassStrigling:

done = m\_springbarleypeaclovergrassstrigling->Do( this, ev->m\_field, ev );

break;

case tov\_YoungForest:

done = m\_youngforest->Do( this, ev->m\_field, ev );

break;

/\* case tov\_OFirstYearDanger: done = m\_ofirstyeardanger->Do( this, ev->m\_field, ev ); break; \*/

default:

g\_msg->Warn( WARN\_BUG, "Farm::LeSwitch(): ""Unknown crop type! ", "" );

exit( 1 );

}

return done;

}

|  |  |  |  |  |  |  |  |
| --- | --- | --- | --- | --- | --- | --- | --- |
| |  |  |  |  |  |  | | --- | --- | --- | --- | --- | --- | | virtual void Farm::MakeStockFarmer | ( | void |  | ) |  | | inlinevirtual |

Reimplemented in PesticideTrialTreatment, PesticideTrialToxicControl, PesticideTrialControl, OrganicPlant, and ConventionalPlant.

References m\_stockfarmer.

{ m\_stockfarmer = true; }

|  |  |  |  |  |  |  |  |
| --- | --- | --- | --- | --- | --- | --- | --- |
| |  |  |  |  |  |  | | --- | --- | --- | --- | --- | --- | | void Farm::Management | ( | void |  | ) |  | | virtual |

Starts the main management loop for the farm and performs some error checking.

References g\_msg, MapErrorMsg::Warn(), and WARN\_BUG.

{

HandleEvents();

for ( unsigned int i = 0; i < m\_fields.size(); i++ ) {

// Check for infinite loop in management plan.

int count = m\_fields[ i ]->GetVegStore();

if ( count >= 0 )

m\_fields[ i ]->SetVegStore( ++count );

if ( count > 800 ) {

// More than two years where nothing happened.

// Raise 'Merry Christmas'!

char error\_num[ 20 ];

sprintf( error\_num, "%d", m\_fields[ i ]->GetVegType() );

g\_msg->Warn( WARN\_BUG, "I the Farm Manager caught infinite loop in tov type:", error\_num );

sprintf( error\_num, "%d", m\_fields[ i ]->m\_tried\_to\_do );

g\_msg->Warn( WARN\_BUG, "It was last seen trying to perform action # ""(or thereabouts):", error\_num );

exit( 1 );

}

}

}

|  |  |  |  |  |  |  |  |  |  |  |  |  |  |  |  |  |  |
| --- | --- | --- | --- | --- | --- | --- | --- | --- | --- | --- | --- | --- | --- | --- | --- | --- | --- |
| |  |  |  |  | | --- | --- | --- | --- | | bool Farm::Molluscicide | ( | LE \* | *a\_field*, | |  |  | double | *a\_user*, | |  |  | int | *a\_days* | |  | ) |  |  | | virtual |

Apply molluscidie to a\_field.

References DO\_IT\_PROB, EL\_TRAMLINE\_DECAYTIME, g\_weather, LE::GetSignal(), Weather::GetWind(), LE\_SIG\_NO\_MOLLUSC, molluscicide, Weather::Raining(), LE::SetLastTreatment(), LE::SetTramlinesDecay(), and LE::Trace().

{

if (0 >= a\_days) {

if ( (!g\_weather->Raining()) && (g\_weather->GetWind()<4.5) &&

(! (a\_field->GetSignal() & LE\_SIG\_NO\_MOLLUSC ))) {

a\_field->Trace( molluscicide );

a\_field->SetLastTreatment( molluscicide );

a\_field->SetTramlinesDecay( EL\_TRAMLINE\_DECAYTIME );

}

return true;

}

else if ( (0 >= a\_days) || (!g\_weather->Raining() && DoIt(DO\_IT\_PROB))) {

if ( ! (a\_field->GetSignal() & LE\_SIG\_NO\_MOLLUSC) ) {

a\_field->Trace( molluscicide );

a\_field->SetLastTreatment( molluscicide );

a\_field->SetTramlinesDecay( EL\_TRAMLINE\_DECAYTIME );

}

return true;

}

return false;

}

|  |  |  |  |  |  |  |  |  |  |  |  |  |  |  |  |  |  |
| --- | --- | --- | --- | --- | --- | --- | --- | --- | --- | --- | --- | --- | --- | --- | --- | --- | --- |
| |  |  |  |  | | --- | --- | --- | --- | | bool Farm::PigsAreOut | ( | LE \* | *a\_field*, | |  |  | double | *a\_user*, | |  |  | int | *a\_days* | |  | ) |  |  | | virtual |

Start a pig grazing event on a\_field today or soon.

References g\_landscape\_p, LE::GetUnsprayedMarginPolyRef(), Landscape::SupplyLEPointer(), and LE::TogglePigGrazing().

{

if ( (0 >= a\_days)|| DoIt(50/a\_days)) {

a\_field->TogglePigGrazing();

int pref=a\_field->GetUnsprayedMarginPolyRef();

if (pref!=-1){

// Must have an unsprayed margin so need to pass the information on to it

LE\* um=g\_landscape\_p->SupplyLEPointer(pref);

um->TogglePigGrazing();

}

return true;

}

return false;

}

|  |  |  |  |  |  |  |  |  |  |  |  |  |  |  |  |  |  |
| --- | --- | --- | --- | --- | --- | --- | --- | --- | --- | --- | --- | --- | --- | --- | --- | --- | --- |
| |  |  |  |  | | --- | --- | --- | --- | | bool Farm::PigsAreOutForced | ( | LE \* | *a\_field*, | |  |  | double | *a\_user*, | |  |  | int | *a\_days* | |  | ) |  |  | | virtual |

Start a pig grazing event on a\_field today - no exceptions.

References g\_landscape\_p, LE::GetUnsprayedMarginPolyRef(), l\_farm\_pig\_veg\_reduce, pigs\_out, LE::ReduceVeg\_Extended(), LE::SetLastTreatment(), Landscape::SupplyLEPointer(), LE::Trace(), and CfgFloat::value().

{

a\_field->SetLastTreatment( pigs\_out );

a\_field->Trace( pigs\_out );

// Reduce the vegetation because of grazing

a\_field->ReduceVeg\_Extended( l\_farm\_pig\_veg\_reduce.value() );

// make this a function of grazing pressure

//and field size - perhaps in a later life

int pref=a\_field->GetUnsprayedMarginPolyRef();

if (pref!=-1){

// Must have an unsprayed margin so need to pass the information on to it

LE\* um=g\_landscape\_p->SupplyLEPointer(pref);

um->SetLastTreatment( pigs\_out );

um->ReduceVeg\_Extended( l\_farm\_pig\_veg\_reduce.value() );

}

return false;

}

|  |  |  |  |  |  |  |  |  |  |  |  |  |  |  |  |  |  |
| --- | --- | --- | --- | --- | --- | --- | --- | --- | --- | --- | --- | --- | --- | --- | --- | --- | --- |
| |  |  |  |  | | --- | --- | --- | --- | | bool Farm::PigsOut | ( | LE \* | *a\_field*, | |  |  | double | *a\_user*, | |  |  | int | *a\_days* | |  | ) |  |  | | virtual |

Generate a 'pigs\_out' event for every day the cattle are on a\_field.

References DO\_IT\_PROB, g\_landscape\_p, LE::GetUnsprayedMarginPolyRef(), l\_farm\_pig\_veg\_reduce, pigs\_out, LE::ReduceVeg\_Extended(), LE::SetLastTreatment(), Landscape::SupplyLEPointer(), LE::TogglePigGrazing(), LE::Trace(), and CfgFloat::value().

{

if ( (0 >= a\_days)|| DoIt(DO\_IT\_PROB))

{

a\_field->TogglePigGrazing();

a\_field->Trace( pigs\_out );

a\_field->SetLastTreatment( pigs\_out );

// Reduce the vegetation because of grazing

a\_field->ReduceVeg\_Extended( l\_farm\_pig\_veg\_reduce.value());

// make this a function of grazing pressure

//and field size - perhaps in a later life

int pref=a\_field->GetUnsprayedMarginPolyRef();

if (pref!=-1){

// Must have an unsprayed margin so need to pass the information on to it

LE\* um=g\_landscape\_p->SupplyLEPointer(pref);

um->SetLastTreatment( pigs\_out );

um->ReduceVeg\_Extended( l\_farm\_pig\_veg\_reduce.value() );

}

return true;

}

return false;

}

|  |  |  |  |  |  |  |  |  |  |  |  |  |  |  |  |  |  |
| --- | --- | --- | --- | --- | --- | --- | --- | --- | --- | --- | --- | --- | --- | --- | --- | --- | --- |
| |  |  |  |  | | --- | --- | --- | --- | | bool Farm::ProductApplication0 | ( | LE \* | *a\_field*, | |  |  | double | *a\_user*, | |  |  | int | *a\_days* | |  | ) |  |  | | virtual |

Apply test pesticide to a\_field.

References Pesticide::DailyQueueAdd(), EL\_TRAMLINE\_DECAYTIME, g\_pest, g\_weather, Weather::GetWind(), LE::Insecticide(), l\_farm\_insecticide\_kills, l\_pest\_product\_0\_amount, product\_treat, Weather::Raining(), LE::SetLastTreatment(), LE::SetTramlinesDecay(), LE::Trace(), CfgFloat::value(), and CfgBool::value().

{

// NOTE Differs from normal pesticide in that it will be done on the last

// day if not managed before

if (0 >= a\_days) {

a\_field->Trace( product\_treat );

a\_field->SetLastTreatment( product\_treat );

if ( l\_farm\_insecticide\_kills.value()) {

a\_field->Insecticide( 0.2 ); }

a\_field->SetTramlinesDecay( EL\_TRAMLINE\_DECAYTIME );

double p=l\_pest\_product\_0\_amount.value();

g\_pest->DailyQueueAdd( a\_field,p );

return true;

} else {

if ( (!g\_weather->Raining()) && (g\_weather->GetWind()<4.5)) {

a\_field->Trace( product\_treat );

a\_field->SetLastTreatment( product\_treat );

if ( l\_farm\_insecticide\_kills.value()) {

a\_field->Insecticide( 0.2 ); }

a\_field->SetTramlinesDecay( EL\_TRAMLINE\_DECAYTIME );

double p=l\_pest\_product\_0\_amount.value();

g\_pest->DailyQueueAdd( a\_field,p );

return true;

}

}

return false;

}

|  |  |  |  |  |  |  |  |  |  |  |  |  |  |  |  |  |  |
| --- | --- | --- | --- | --- | --- | --- | --- | --- | --- | --- | --- | --- | --- | --- | --- | --- | --- |
| |  |  |  |  | | --- | --- | --- | --- | | bool Farm::ProductApplication1 | ( | LE \* | *a\_field*, | |  |  | double | *a\_user*, | |  |  | int | *a\_days* | |  | ) |  |  | | virtual |

Apply test pesticide to a\_field.

References Pesticide::DailyQueueAdd(), EL\_TRAMLINE\_DECAYTIME, g\_pest, g\_weather, Weather::GetWind(), LE::Insecticide(), l\_farm\_insecticide\_kills, l\_pest\_product\_1\_amount, product\_treat, Weather::Raining(), LE::SetLastTreatment(), LE::SetTramlinesDecay(), LE::Trace(), CfgFloat::value(), and CfgBool::value().

{

// NOTE Differs from normal pesticide in that it will be done on the last

// day if not managed before

if (0 >= a\_days) {

a\_field->Trace( product\_treat );

a\_field->SetLastTreatment( product\_treat );

if ( l\_farm\_insecticide\_kills.value())

{

a\_field->Insecticide( 0.2 );

}

a\_field->SetTramlinesDecay( EL\_TRAMLINE\_DECAYTIME );

double p=l\_pest\_product\_1\_amount.value();

g\_pest->DailyQueueAdd( a\_field,p );

return true;

} else {

if ( (!g\_weather->Raining()) && (g\_weather->GetWind()<4.5)) {

a\_field->Trace( product\_treat );

a\_field->SetLastTreatment( product\_treat );

if ( l\_farm\_insecticide\_kills.value()) {

a\_field->Insecticide( 0.2 ); }

a\_field->SetTramlinesDecay( EL\_TRAMLINE\_DECAYTIME );

double p=l\_pest\_product\_1\_amount.value();

g\_pest->DailyQueueAdd( a\_field,p );

return true;

}

}

return false;

}

|  |  |  |  |  |  |  |  |
| --- | --- | --- | --- | --- | --- | --- | --- |
| |  |  |  |  |  |  | | --- | --- | --- | --- | --- | --- | | void Farm::ReadRotation | ( | std::string | *fname* | ) |  | | protected |

Reads a rotation file into the rotation.

References g\_msg, and MapErrorMsg::Warn().

Referenced by OrganicCattle::OrganicCattle(), OrganicPig::OrganicPig(), OrganicPlant::OrganicPlant(), PesticideTrialTreatment::PesticideTrialTreatment(), UserDefinedFarm1::UserDefinedFarm1(), UserDefinedFarm10::UserDefinedFarm10(), UserDefinedFarm11::UserDefinedFarm11(), UserDefinedFarm12::UserDefinedFarm12(), UserDefinedFarm13::UserDefinedFarm13(), UserDefinedFarm14::UserDefinedFarm14(), UserDefinedFarm15::UserDefinedFarm15(), UserDefinedFarm16::UserDefinedFarm16(), UserDefinedFarm17::UserDefinedFarm17(), UserDefinedFarm2::UserDefinedFarm2(), UserDefinedFarm3::UserDefinedFarm3(), UserDefinedFarm4::UserDefinedFarm4(), UserDefinedFarm5::UserDefinedFarm5(), UserDefinedFarm6::UserDefinedFarm6(), UserDefinedFarm7::UserDefinedFarm7(), UserDefinedFarm8::UserDefinedFarm8(), and UserDefinedFarm9::UserDefinedFarm9().

{

ifstream ifile;

ifile.open(str.c\_str(),ios::in);

if ( !ifile.is\_open() ) {

g\_msg->Warn( "Cannot open file: ", str.c\_str() );

exit( 1 );

}

int nocrops;

ifile >> nocrops;

m\_rotation.resize( nocrops );

std::string cropref;

for ( int i = 0; i < nocrops; i++ ) {

ifile >> cropref;

TTypesOfVegetation tov = TranslateCropCodes( cropref );

m\_rotation[ i ] = tov;

}

ifile.close();

}

|  |  |  |  |  |  |
| --- | --- | --- | --- | --- | --- |
| void Farm::RemoveField | ( | LE \* | *a\_field* | ) |  |

Removes a field to a farm.

References g\_msg, MapErrorMsg::Warn(), and WARN\_BUG.

{

int nf = (int) m\_fields.size();

for ( int i = 0; i < nf; i++ ) {

if ( m\_fields[ i ] == a\_field ) {

m\_fields.erase( m\_fields.begin() + i );

return;

}

}

// If we reach here there is something wrong because the field is not a

// member of this farm

g\_msg->Warn( WARN\_BUG, "Farm::RemoveField(LE\* a\_field): ""Unknown field! ", "" );

exit( 1 );

}

|  |  |  |  |  |  |  |  |  |  |  |  |  |  |  |  |  |  |
| --- | --- | --- | --- | --- | --- | --- | --- | --- | --- | --- | --- | --- | --- | --- | --- | --- | --- |
| |  |  |  |  | | --- | --- | --- | --- | | bool Farm::RowCultivation | ( | LE \* | *a\_field*, | |  |  | double | *a\_user*, | |  |  | int | *a\_days* | |  | ) |  |  | | virtual |

Carry out a harrowing between crop rows on a\_field.

References Calendar::Date(), DO\_IT\_PROB, EL\_TRAMLINE\_DECAYTIME, g\_date, g\_landscape\_p, g\_weather, Weather::GetRainPeriod(), LE::GetUnsprayedMarginPolyRef(), LE::InsectMortality(), LE::ReduceWeedBiomass(), row\_cultivation, LE::SetLastTreatment(), LE::SetTramlinesDecay(), Landscape::SupplyLEPointer(), and LE::Trace().

{

if ( (0 >= a\_days) && (g\_weather->GetRainPeriod(g\_date->Date(),3)<0.1) )

{

// Too much rain, just give up and claim we did it.

return true;

}

if ( (0 >= a\_days) ||

((g\_weather->GetRainPeriod(g\_date->Date(),3)<0.1) && DoIt(DO\_IT\_PROB))

) {

a\_field->Trace( row\_cultivation );

a\_field->SetLastTreatment( row\_cultivation );

a\_field->ReduceWeedBiomass( 0.5 );

a\_field->InsectMortality( 0.25 );

a\_field->SetTramlinesDecay( EL\_TRAMLINE\_DECAYTIME );

int pref=a\_field->GetUnsprayedMarginPolyRef();

if (pref!=-1){

// Must have an unsprayed margin so need to pass the information on to it

LE\* um=g\_landscape\_p->SupplyLEPointer(pref);

um->SetLastTreatment( row\_cultivation );

um->InsectMortality( 0.25 );

um->ReduceWeedBiomass( 0.5 );

um->SetTramlinesDecay( EL\_TRAMLINE\_DECAYTIME );

}

return true;

}

return false;

}

|  |  |  |  |  |  |  |  |
| --- | --- | --- | --- | --- | --- | --- | --- |
| |  |  |  |  |  |  | | --- | --- | --- | --- | --- | --- | | void Farm::SetFarmNumber | ( | int | *a\_farm\_num* | ) |  | | inline |

References m\_farm\_num.

{ m\_farm\_num = a\_farm\_num; }

|  |  |  |  |  |  |  |  |  |  |  |  |  |  |  |  |  |  |
| --- | --- | --- | --- | --- | --- | --- | --- | --- | --- | --- | --- | --- | --- | --- | --- | --- | --- |
| |  |  |  |  | | --- | --- | --- | --- | | bool Farm::SleepAllDay | ( | LE \* | *a\_field*, | |  |  | double | *a\_user*, | |  |  | int | *a\_days* | |  | ) |  |  | | virtual |

Nothing to to today on a\_field.

References g\_landscape\_p, LE::GetUnsprayedMarginPolyRef(), LE::SetLastTreatment(), sleep\_all\_day, Landscape::SupplyLEPointer(), and LE::Trace().

{

a\_field->Trace( sleep\_all\_day );

a\_field->SetLastTreatment( sleep\_all\_day );

int pref=a\_field->GetUnsprayedMarginPolyRef();

if (pref!=-1){

// Must have an unsprayed margin so need to pass the information on to it

LE\* um=g\_landscape\_p->SupplyLEPointer(pref);

um->SetLastTreatment(sleep\_all\_day);

}

return true;

}

|  |  |  |  |  |  |  |  |  |  |  |  |  |  |  |  |  |  |
| --- | --- | --- | --- | --- | --- | --- | --- | --- | --- | --- | --- | --- | --- | --- | --- | --- | --- |
| |  |  |  |  | | --- | --- | --- | --- | | bool Farm::SpringHarrow | ( | LE \* | *a\_field*, | |  |  | double | *a\_user*, | |  |  | int | *a\_days* | |  | ) |  |  | | virtual |

Carry out a harrow event in the spring on a\_field.

References DO\_IT\_PROB, g\_landscape\_p, g\_weather, LE::GetUnsprayedMarginPolyRef(), LE::InsectMortality(), Weather::Raining(), LE::SetLastTreatment(), spring\_harrow, Landscape::SupplyLEPointer(), LE::Trace(), and LE::ZeroVeg().

{

if ( (0 >= a\_days) || (!g\_weather->Raining() && DoIt(DO\_IT\_PROB))) {

a\_field->Trace( spring\_harrow );

a\_field->SetLastTreatment( spring\_harrow );

// 30% insect mortality

a\_field->InsectMortality( 0.7 );

// remove all vegetation

a\_field->ZeroVeg();

int pref=a\_field->GetUnsprayedMarginPolyRef();

if (pref!=-1){

// Must have an unsprayed margin so need to pass the information on to it

LE\* um=g\_landscape\_p->SupplyLEPointer(pref);

um->SetLastTreatment( spring\_harrow );

um->InsectMortality( 0.7 );

um->ZeroVeg();

}

return true;

}

return false;

}

|  |  |  |  |  |  |  |  |  |  |  |  |  |  |  |  |  |  |
| --- | --- | --- | --- | --- | --- | --- | --- | --- | --- | --- | --- | --- | --- | --- | --- | --- | --- |
| |  |  |  |  | | --- | --- | --- | --- | | bool Farm::SpringPlough | ( | LE \* | *a\_field*, | |  |  | double | *a\_user*, | |  |  | int | *a\_days* | |  | ) |  |  | | virtual |

Carry out a ploughing event in the spring on a\_field.

References DO\_IT\_PROB, g\_landscape\_p, g\_weather, LE::GetUnsprayedMarginPolyRef(), LE::InsectMortality(), Weather::Raining(), LE::SetLastTreatment(), spring\_plough, Landscape::SupplyLEPointer(), LE::Trace(), and LE::ZeroVeg().

{

if ( (0 >= a\_days) || (!g\_weather->Raining() && DoIt(DO\_IT\_PROB))) {

a\_field->Trace( spring\_plough );

a\_field->SetLastTreatment( spring\_plough );

// Apply 90% mortality to the insects

a\_field->InsectMortality( 0.1 );

// Reduce the vegetation to zero

int pref=a\_field->GetUnsprayedMarginPolyRef();

if (pref!=-1){

// Must have an unsprayed margin so need to pass the information on to it

LE\* um=g\_landscape\_p->SupplyLEPointer(pref);

um->SetLastTreatment( spring\_plough );

um->InsectMortality( 0.1);

um->ZeroVeg();

}

return true;

}

return false;

}

|  |  |  |  |  |  |  |  |  |  |  |  |  |  |  |  |  |  |
| --- | --- | --- | --- | --- | --- | --- | --- | --- | --- | --- | --- | --- | --- | --- | --- | --- | --- |
| |  |  |  |  | | --- | --- | --- | --- | | bool Farm::SpringRoll | ( | LE \* | *a\_field*, | |  |  | double | *a\_user*, | |  |  | int | *a\_days* | |  | ) |  |  | | virtual |

Carry out a roll event in the spring on a\_field.

References DO\_IT\_PROB, g\_landscape\_p, g\_weather, LE::GetUnsprayedMarginPolyRef(), Weather::Raining(), LE::SetLastTreatment(), spring\_roll, Landscape::SupplyLEPointer(), and LE::Trace().

{

if ( (0 >= a\_days) || (!g\_weather->Raining() && DoIt(DO\_IT\_PROB))) {

a\_field->Trace( spring\_roll );

a\_field->SetLastTreatment( spring\_roll );

int pref=a\_field->GetUnsprayedMarginPolyRef();

if (pref!=-1){

// Must have an unsprayed margin so need to pass the information on to it

LE\* um=g\_landscape\_p->SupplyLEPointer(pref);

um->SetLastTreatment( spring\_roll );

}

return true;

}

return false;

}

|  |  |  |  |  |  |  |  |  |  |  |  |  |  |  |  |  |  |
| --- | --- | --- | --- | --- | --- | --- | --- | --- | --- | --- | --- | --- | --- | --- | --- | --- | --- |
| |  |  |  |  | | --- | --- | --- | --- | | bool Farm::SpringSow | ( | LE \* | *a\_field*, | |  |  | double | *a\_user*, | |  |  | int | *a\_days* | |  | ) |  |  | | virtual |

Carry out a sowing event in the spring on a\_field.

References DO\_IT\_PROB, g\_landscape\_p, g\_weather, LE::GetUnsprayedMarginPolyRef(), Weather::Raining(), LE::SetGrowthPhase(), LE::SetLastTreatment(), sow, spring\_sow, Landscape::SupplyLEPointer(), LE::Trace(), and LE::ZeroVeg().

{

if ( (0 >= a\_days) || (!g\_weather->Raining() && DoIt(DO\_IT\_PROB))) {

a\_field->Trace( spring\_sow );

a\_field->SetLastTreatment( spring\_sow );

a\_field->SetGrowthPhase( sow );

// Reduce the vegetation to zero - should not strictly be necessary, but prevents any false starts in the crop growth.

a\_field->ZeroVeg();

int pref=a\_field->GetUnsprayedMarginPolyRef();

if (pref!=-1){

// Must have an unsprayed margin so need to pass the information on to it

LE\* um=g\_landscape\_p->SupplyLEPointer(pref);

um->SetLastTreatment( spring\_sow );

um->SetGrowthPhase( sow );

um->ZeroVeg();

}

return true;

}

return false;

}

|  |  |  |  |  |  |  |  |  |  |  |  |  |  |  |  |  |  |
| --- | --- | --- | --- | --- | --- | --- | --- | --- | --- | --- | --- | --- | --- | --- | --- | --- | --- |
| |  |  |  |  | | --- | --- | --- | --- | | bool Farm::StrawChopping | ( | LE \* | *a\_field*, | |  |  | double | *a\_user*, | |  |  | int | *a\_days* | |  | ) |  |  | | virtual |

Carry out straw chopping on a\_field.

References Calendar::Date(), DO\_IT\_PROB, EL\_TRAMLINE\_DECAYTIME, g\_date, g\_landscape\_p, g\_weather, Weather::GetRainPeriod(), LE::GetUnsprayedMarginPolyRef(), LE::InsectMortality(), LE::SetLastTreatment(), LE::SetTramlinesDecay(), straw\_chopping, Landscape::SupplyLEPointer(), and LE::Trace().

{

if ( (0 >= a\_days) ||

((g\_weather->GetRainPeriod(g\_date->Date(),5)<0.1) && DoIt(DO\_IT\_PROB))

) {

a\_field->Trace( straw\_chopping );

a\_field->SetLastTreatment( straw\_chopping );

a\_field->InsectMortality( 0.4 );

a\_field->SetTramlinesDecay( EL\_TRAMLINE\_DECAYTIME );

int pref=a\_field->GetUnsprayedMarginPolyRef();

if (pref!=-1){

// Must have an unsprayed margin so need to pass the information on to it

LE\* um=g\_landscape\_p->SupplyLEPointer(pref);

um->SetLastTreatment( straw\_chopping );

um->InsectMortality( 0.4 );

um->SetTramlinesDecay( EL\_TRAMLINE\_DECAYTIME );

}

return true;

}

return false;

}

|  |  |  |  |  |  |  |  |  |  |  |  |  |  |  |  |  |  |
| --- | --- | --- | --- | --- | --- | --- | --- | --- | --- | --- | --- | --- | --- | --- | --- | --- | --- |
| |  |  |  |  | | --- | --- | --- | --- | | bool Farm::Strigling | ( | LE \* | *a\_field*, | |  |  | double | *a\_user*, | |  |  | int | *a\_days* | |  | ) |  |  | | virtual |

Carry out a mechanical weeding on a\_field.

References Calendar::Date(), DO\_IT\_PROB, EL\_STRIGLING\_DELAYTIME, EL\_TRAMLINE\_DECAYTIME, g\_date, g\_landscape\_p, g\_weather, Weather::GetRainPeriod(), LE::GetUnsprayedMarginPolyRef(), LE::InsectMortality(), LE::ReduceWeedBiomass(), LE::SetHerbicideDelay(), LE::SetLastTreatment(), LE::SetTramlinesDecay(), strigling, Landscape::SupplyLEPointer(), and LE::Trace().

{

// Force strigling if it has not been done already!!! This happens regardless of weather as of 26/10/2005

if ( (0 >= a\_days) )// && (g\_weather->GetRainPeriod(g\_date->Date(),3)>0.1) )

{

a\_field->Trace( strigling );

a\_field->SetLastTreatment( strigling );

a\_field->ReduceWeedBiomass( 0.05 );

a\_field->InsectMortality( 0.7 );

a\_field->SetTramlinesDecay( EL\_TRAMLINE\_DECAYTIME );

a\_field->SetHerbicideDelay( EL\_STRIGLING\_DELAYTIME );

int pref=a\_field->GetUnsprayedMarginPolyRef();

if (pref!=-1){

// Must have an unsprayed margin so need to pass the information on to it

LE\* um=g\_landscape\_p->SupplyLEPointer(pref);

um->SetLastTreatment( strigling );

um->ReduceWeedBiomass( 0.05 );

um->InsectMortality( 0.7 );

um->SetTramlinesDecay( EL\_TRAMLINE\_DECAYTIME );

um->SetHerbicideDelay( EL\_STRIGLING\_DELAYTIME );

return true;

}

}

if ( (0 >= a\_days) ||

((g\_weather->GetRainPeriod(g\_date->Date(),3)<0.1) && DoIt(DO\_IT\_PROB))

) {

a\_field->Trace( strigling );

a\_field->SetLastTreatment( strigling );

a\_field->ReduceWeedBiomass( 0.05 );

a\_field->InsectMortality( 0.7 );

a\_field->SetTramlinesDecay( EL\_TRAMLINE\_DECAYTIME );

a\_field->SetHerbicideDelay( EL\_STRIGLING\_DELAYTIME );

int pref=a\_field->GetUnsprayedMarginPolyRef();

if (pref!=-1){

// Must have an unsprayed margin so need to pass the information on to it

LE\* um=g\_landscape\_p->SupplyLEPointer(pref);

um->SetLastTreatment( strigling );

um->ReduceWeedBiomass( 0.05 );

um->InsectMortality( 0.7 );

um->SetTramlinesDecay( EL\_TRAMLINE\_DECAYTIME );

um->SetHerbicideDelay( EL\_STRIGLING\_DELAYTIME );

}

return true;

}

return false;

}

|  |  |  |  |  |  |  |  |  |  |  |  |  |  |  |  |  |  |
| --- | --- | --- | --- | --- | --- | --- | --- | --- | --- | --- | --- | --- | --- | --- | --- | --- | --- |
| |  |  |  |  | | --- | --- | --- | --- | | bool Farm::StriglingSow | ( | LE \* | *a\_field*, | |  |  | double | *a\_user*, | |  |  | int | *a\_days* | |  | ) |  |  | | virtual |

Carry out a mechanical weeding followed by sowing on a\_field.

References Calendar::Date(), DO\_IT\_PROB, EL\_STRIGLING\_DELAYTIME, EL\_TRAMLINE\_DECAYTIME, g\_date, g\_landscape\_p, g\_weather, Weather::GetRainPeriod(), LE::GetUnsprayedMarginPolyRef(), LE::InsectMortality(), LE::ReduceWeedBiomass(), LE::SetHerbicideDelay(), LE::SetLastTreatment(), LE::SetTramlinesDecay(), strigling\_sow, Landscape::SupplyLEPointer(), and LE::Trace().

{

//2 days good weather afterwards

if ( (0 >= a\_days) && (g\_weather->GetRainPeriod(g\_date->Date(),3)<0.1) )

{

return true;

}

if ( (0 >= a\_days) ||

((g\_weather->GetRainPeriod(g\_date->Date(),3)<0.1) && DoIt(DO\_IT\_PROB))

) {

a\_field->Trace( strigling\_sow );

a\_field->SetLastTreatment( strigling\_sow );

a\_field->ReduceWeedBiomass( 0.05 );

a\_field->InsectMortality( 0.7 );

a\_field->SetTramlinesDecay( EL\_TRAMLINE\_DECAYTIME );

a\_field->SetHerbicideDelay( EL\_STRIGLING\_DELAYTIME );

int pref=a\_field->GetUnsprayedMarginPolyRef();

if (pref!=-1){

// Must have an unsprayed margin so need to pass the information on to it

LE\* um=g\_landscape\_p->SupplyLEPointer(pref);

um->SetLastTreatment( strigling\_sow );

um->ReduceWeedBiomass( 0.05 );

um->InsectMortality( 0.7 );

um->SetTramlinesDecay( EL\_TRAMLINE\_DECAYTIME );

um->SetHerbicideDelay( EL\_STRIGLING\_DELAYTIME );

}

return true;

}

return false;

}

|  |  |  |  |  |  |  |  |  |  |  |  |  |  |  |  |  |  |
| --- | --- | --- | --- | --- | --- | --- | --- | --- | --- | --- | --- | --- | --- | --- | --- | --- | --- |
| |  |  |  |  | | --- | --- | --- | --- | | bool Farm::StubbleHarrowing | ( | LE \* | *a\_field*, | |  |  | double | *a\_user*, | |  |  | int | *a\_days* | |  | ) |  |  | | virtual |

Carry out stubble harrowing on a\_field.

References Calendar::Date(), DO\_IT\_PROB, g\_date, g\_landscape\_p, g\_weather, Weather::GetRainPeriod(), LE::GetUnsprayedMarginPolyRef(), LE::InsectMortality(), LE::SetLastTreatment(), stubble\_harrowing, Landscape::SupplyLEPointer(), LE::Trace(), and LE::ZeroVeg().

{

if ( (0 >= a\_days) ||

((g\_weather->GetRainPeriod(g\_date->Date(),3)<0.1) && DoIt(DO\_IT\_PROB))

) {

a\_field->Trace( stubble\_harrowing );

a\_field->SetLastTreatment( stubble\_harrowing );

a\_field->InsectMortality( 0.25 );

a\_field->ZeroVeg();

int pref=a\_field->GetUnsprayedMarginPolyRef();

if (pref!=-1){

// Must have an unsprayed margin so need to pass the information on to it

LE\* um=g\_landscape\_p->SupplyLEPointer(pref);

um->SetLastTreatment( stubble\_harrowing );

um->ZeroVeg();

um->InsectMortality( 0.25 );

}

return true;

}

return false;

}

|  |  |  |  |  |  |  |  |  |  |  |  |  |  |  |  |  |  |
| --- | --- | --- | --- | --- | --- | --- | --- | --- | --- | --- | --- | --- | --- | --- | --- | --- | --- |
| |  |  |  |  | | --- | --- | --- | --- | | bool Farm::Swathing | ( | LE \* | *a\_field*, | |  |  | double | *a\_user*, | |  |  | int | *a\_days* | |  | ) |  |  | | virtual |

Cut the crop on a\_field and leave it lying (probably rape)

References DO\_IT\_PROB, EL\_TRAMLINE\_DECAYTIME, g\_landscape\_p, g\_weather, LE::GetUnsprayedMarginPolyRef(), LE::InsectMortality(), Weather::Raining(), LE::SetLastTreatment(), LE::SetTramlinesDecay(), Landscape::SupplyLEPointer(), swathing, and LE::Trace().

{

if ( (0 >= a\_days) || (!g\_weather->Raining() && DoIt(DO\_IT\_PROB))) {

a\_field->Trace( swathing );

a\_field->SetLastTreatment( swathing );

a\_field->InsectMortality( 0.5 );

a\_field->SetTramlinesDecay( EL\_TRAMLINE\_DECAYTIME );

int pref=a\_field->GetUnsprayedMarginPolyRef();

if (pref!=-1){

// Must have an unsprayed margin so need to pass the information on to it

LE\* um=g\_landscape\_p->SupplyLEPointer(pref);

um->SetLastTreatment( swathing );

um->InsectMortality( 0.5 );

um->SetTramlinesDecay( EL\_TRAMLINE\_DECAYTIME );

}

return true;

}

return false;

}

|  |  |  |  |  |  |  |  |  |  |  |  |  |  |  |  |  |  |
| --- | --- | --- | --- | --- | --- | --- | --- | --- | --- | --- | --- | --- | --- | --- | --- | --- | --- |
| |  |  |  |  | | --- | --- | --- | --- | | bool Farm::SynInsecticideTreat | ( | LE \* | *a\_field*, | |  |  | double | *a\_user*, | |  |  | int | *a\_days* | |  | ) |  |  | | virtual |

Apply special insecticide to a\_field.

References EL\_TRAMLINE\_DECAYTIME, g\_weather, LE::GetSignal(), Weather::GetWind(), LE::Insecticide(), l\_farm\_insecticide\_kills, LE\_SIG\_NO\_SYNG\_INSECT, Weather::Raining(), LE::SetLastTreatment(), LE::SetTramlinesDecay(), syninsecticide\_treat, LE::Trace(), and CfgBool::value().

{

// NOTE Differs from normal insecticide in that it will be done on the last

// day if not managed before

if (0 >= a\_days) {

if ( ! (a\_field->GetSignal() & LE\_SIG\_NO\_SYNG\_INSECT )) {

a\_field->Trace( syninsecticide\_treat );

a\_field->SetLastTreatment( syninsecticide\_treat );

if ( l\_farm\_insecticide\_kills.value()) {

a\_field->Insecticide( 0.2 );

}

a\_field->SetTramlinesDecay( EL\_TRAMLINE\_DECAYTIME );

}

// double p=l\_pest\_insecticide\_amount.value();

// g\_pest->DailyQueueAdd( a\_field,p );

return true;

} else {

if ( (!g\_weather->Raining()) && (g\_weather->GetWind()<4.5)) {

if ( ! (a\_field->GetSignal() & LE\_SIG\_NO\_SYNG\_INSECT ) ) {

a\_field->Trace( syninsecticide\_treat );

a\_field->SetLastTreatment( syninsecticide\_treat );

if ( l\_farm\_insecticide\_kills.value()) {

a\_field->Insecticide( 0.2 );

}

a\_field->SetTramlinesDecay( EL\_TRAMLINE\_DECAYTIME );

}

// g\_pest->DailyQueueAdd( a\_field, l\_pest\_insecticide\_amount.value());

return true;

}

}

return false;

}

|  |  |  |  |  |  |
| --- | --- | --- | --- | --- | --- |
| TTypesOfVegetation Farm::TranslateCropCodes | ( | std::string & | *str* | ) |  |

References g\_msg, tov\_AgroChemIndustryCereal, tov\_Carrots, tov\_CloverGrassGrazed1, tov\_CloverGrassGrazed2, tov\_FieldPeas, tov\_FieldPeasStrigling, tov\_FodderBeet, tov\_FodderGrass, tov\_Maize, tov\_MaizeSilage, tov\_MaizeStrigling, tov\_Oats, tov\_OBarleyPeaCloverGrass, tov\_OCarrots, tov\_OCloverGrassGrazed1, tov\_OCloverGrassGrazed2, tov\_OCloverGrassSilage1, tov\_OFieldPeas, tov\_OFieldPeasSilage, tov\_OGrazingPigs, tov\_OMaizeSilage, tov\_OOats, tov\_OPermanentGrassGrazed, tov\_OPotatoes, tov\_OSBarleySilage, tov\_OSeedGrass1, tov\_OSeedGrass2, tov\_OSpringBarley, tov\_OSpringBarleyClover, tov\_OSpringBarleyExt, tov\_OSpringBarleyGrass, tov\_OTriticale, tov\_OWinterBarley, tov\_OWinterBarleyExt, tov\_OWinterRape, tov\_OWinterRye, tov\_OWinterWheatUndersown, tov\_OWinterWheatUndersownExt, tov\_PermanentGrassGrazed, tov\_PermanentGrassLowYield, tov\_PermanentGrassTussocky, tov\_PermanentSetaside, tov\_Potatoes, tov\_PotatoesIndustry, tov\_SeedGrass1, tov\_SeedGrass2, tov\_Setaside, tov\_SpringBarley, tov\_SpringBarleyCloverGrass, tov\_SpringBarleyCloverGrassStrigling, tov\_SpringBarleyGrass, tov\_SpringBarleyPeaCloverGrassStrigling, tov\_SpringBarleyPTreatment, tov\_SpringBarleySeed, tov\_SpringBarleySilage, tov\_SpringBarleySKManagement, tov\_SpringBarleyStrigling, tov\_SpringBarleyStriglingCulm, tov\_SpringBarleyStriglingSingle, tov\_SpringRape, tov\_SpringWheat, tov\_Triticale, tov\_WinterBarley, tov\_WinterBarleyStrigling, tov\_WinterRape, tov\_WinterRapeStrigling, tov\_WinterRye, tov\_WinterRyeStrigling, tov\_WinterWheat, tov\_WinterWheatShort, tov\_WinterWheatStrigling, tov\_WinterWheatStriglingCulm, tov\_WinterWheatStriglingSingle, tov\_WWheatPControl, tov\_WWheatPToxicControl, tov\_WWheatPTreatment, tov\_YoungForest, MapErrorMsg::Warn(), and WARN\_FILE.

Referenced by UserDefinedFarm::UserDefinedFarm().

{

// This simply checks through the list of legal crop names and returns

// the correct tov type

string str = astr;

// Unfortunately switch cannot use string so the long way:

if ( str == "SpringBarley" ) return tov\_SpringBarley;

if ( str == "SpringBarleyPTreatment" ) return tov\_SpringBarleyPTreatment;

if ( str == "SpringBarleySKManagement" ) return tov\_SpringBarleySKManagement;

if ( str == "WinterBarley" ) return tov\_WinterBarley;

if ( str == "SpringWheat" ) return tov\_SpringWheat;

if ( str == "WinterWheat" ) return tov\_WinterWheat;

if ( str == "WinterRye" ) return tov\_WinterRye;

if ( str == "Oats" ) return tov\_Oats;

if ( str == "OOats" ) return tov\_OOats;

if ( str == "Triticale" ) return tov\_Triticale;

if ( str == "Maize" ) return tov\_Maize;

if ( str == "MaizeSilage" ) return tov\_MaizeSilage;

if ( str == "SpringBarleySeed" ) return tov\_SpringBarleySeed;

if ( str == "SpringRape" ) return tov\_SpringRape;

if ( str == "WinterRape" ) return tov\_WinterRape;

if ( str == "FieldPeas" ) return tov\_FieldPeas;

if ( str == "Setaside" ) return tov\_Setaside;

if ( str == "PermanentSetaside" ) return tov\_PermanentSetaside;

if ( str == "FodderBeet" ) return tov\_FodderBeet;

if ( str == "FodderGrass" ) return tov\_FodderGrass;

if ( str == "CloverGrassGrazed1" ) return tov\_CloverGrassGrazed1;

if ( str == "PotatoesIndustry" ) return tov\_PotatoesIndustry;

if ( str == "Potatoes" ) return tov\_Potatoes;

if ( str == "SeedGrass1" ) return tov\_SeedGrass1;

if ( str == "OWinterBarley" ) return tov\_OWinterBarley;

if ( str == "OWinterBarleyExt" ) return tov\_OWinterBarleyExt;

if ( str == "SpringBarleySilage" ) return tov\_SpringBarleySilage;

if ( str == "OWinterRye" ) return tov\_OWinterRye;

if ( str == "OFieldPeasSilage" ) return tov\_OFieldPeasSilage;

if ( str == "SpringBarleyGrass" ) return tov\_SpringBarleyGrass;

if ( str == "SpringBarleyCloverGrass" ) return tov\_SpringBarleyCloverGrass;

if ( str == "OBarleyPeaCloverGrass" ) return tov\_OBarleyPeaCloverGrass;

if ( str == "OWinterRape" ) return tov\_OWinterRape;

if ( str == "PermanentGrassGrazed" ) return tov\_PermanentGrassGrazed;

if ( str == "PermanentGrassLowYield" ) return tov\_PermanentGrassLowYield;

if ( str == "PermanentGrassTussocky" ) return tov\_PermanentGrassTussocky;

if ( str == "CloverGrassGrazed2" ) return tov\_CloverGrassGrazed2;

if ( str == "SeedGrass2" ) return tov\_SeedGrass2;

if ( str == "OSpringBarley" ) return tov\_OSpringBarley;

if ( str == "OSpringBarleyExt" ) return tov\_OSpringBarleyExt;

if ( str == "OWinterWheatUndersown" ) return tov\_OWinterWheatUndersown;

if ( str == "OWinterWheatUndersownExt" ) return tov\_OWinterWheatUndersownExt;

if ( str == "OOats" ) return tov\_OOats;

if ( str == "OTriticale" ) return tov\_OTriticale;

if ( str == "OFieldPeas" ) return tov\_OFieldPeas;

if ( str == "OCloverGrassGrazed1" ) return tov\_OCloverGrassGrazed1;

if ( str == "OGrazingPigs" ) return tov\_OGrazingPigs;

if ( str == "OCarrots" ) return tov\_OCarrots;

if ( str == "Carrots" ) return tov\_Carrots;

if ( str == "OPotatoes" ) return tov\_OPotatoes;

if ( str == "OSeedGrass1" ) return tov\_OSeedGrass1;

if ( str == "OSpringBarleyGrass" ) return tov\_OSpringBarleyGrass;

if ( str == "OSpringBarleyClover" ) return tov\_OSpringBarleyClover;

if ( str == "OPermanentGrassGrazed" ) return tov\_OPermanentGrassGrazed;

if ( str == "OCloverGrassSilage1" ) return tov\_OCloverGrassSilage1;

if ( str == "OCloverGrassGrazed2" ) return tov\_OCloverGrassGrazed2;

if ( str == "OSeedGrass2" ) return tov\_OSeedGrass2;

if ( str == "WWheatPControl" ) return tov\_WWheatPControl;

if ( str == "WWheatPToxicControl" ) return tov\_WWheatPToxicControl;

if ( str == "WWheatPTreatment" ) return tov\_WWheatPTreatment;

if ( str == "AgroChemIndustryCereal" ) return tov\_AgroChemIndustryCereal;

if ( str == "WinterWheatShort" ) return tov\_WinterWheatShort;

if ( str == "WinterWheatStrigling" ) return tov\_WinterWheatStrigling;

if ( str == "WinterWheatStriglingCulm" ) return tov\_WinterWheatStriglingCulm;

if ( str == "WinterWheatStriglingSgl" ) return tov\_WinterWheatStriglingSingle;

if ( str == "SpringBarleyCloverGrassStrigling" ) return tov\_SpringBarleyCloverGrassStrigling;

if ( str == "SpringBarleyStrigling" ) return tov\_SpringBarleyStrigling;

if ( str == "SpringBarleyStriglingSingle" ) return tov\_SpringBarleyStriglingSingle;

if ( str == "SpringBarleyStriglingCulm" ) return tov\_SpringBarleyStriglingCulm;

if ( str == "MaizeStrigling" ) return tov\_MaizeStrigling;

if ( str == "WinterRapeStrigling" ) return tov\_WinterRapeStrigling;

if ( str == "WinterRyeStrigling" ) return tov\_WinterRyeStrigling;

if ( str == "WinterBarleyStrigling" ) return tov\_WinterBarleyStrigling;

if ( str == "FieldPeasStrigling" ) return tov\_FieldPeasStrigling;

if ( str == "SpringBarleyPeaCloverGrassStrigling" ) return tov\_SpringBarleyPeaCloverGrassStrigling;

if (str == "YoungForest") return tov\_YoungForest;

if (str == "OMaizeSilage") return tov\_OMaizeSilage;

if (str == "OSBarleySilage") return tov\_OSBarleySilage;

// No match so issue a warning and quit

g\_msg->Warn( WARN\_FILE, "Farm::TranslateCropCodes():"" Unknown Crop Code ", str.c\_str() );

exit( 1 );

}

|  |  |  |  |  |  |  |  |  |  |  |  |  |  |  |  |  |  |
| --- | --- | --- | --- | --- | --- | --- | --- | --- | --- | --- | --- | --- | --- | --- | --- | --- | --- |
| |  |  |  |  | | --- | --- | --- | --- | | bool Farm::Trial\_Control | ( | LE \* | *a\_field*, | |  |  | double | *a\_user*, | |  |  | int | *a\_days* | |  | ) |  |  | | virtual |

Special pesticide trial functionality.

References EL\_TRAMLINE\_DECAYTIME, LE::SetLastTreatment(), LE::SetTramlinesDecay(), LE::Trace(), and trial\_control.

{

// Will always do this at the first chance

a\_field->Trace( trial\_control );

a\_field->SetLastTreatment( trial\_control );

a\_field->SetTramlinesDecay( EL\_TRAMLINE\_DECAYTIME );

return true;

}

|  |  |  |  |  |  |  |  |  |  |  |  |  |  |  |  |  |  |
| --- | --- | --- | --- | --- | --- | --- | --- | --- | --- | --- | --- | --- | --- | --- | --- | --- | --- |
| |  |  |  |  | | --- | --- | --- | --- | | bool Farm::Trial\_PesticideTreat | ( | LE \* | *a\_field*, | |  |  | double | *a\_user*, | |  |  | int | *a\_days* | |  | ) |  |  | | virtual |

Special pesticide trial functionality.

References EL\_TRAMLINE\_DECAYTIME, LE::SetLastTreatment(), LE::SetTramlinesDecay(), LE::Trace(), and trial\_insecticidetreat.

{

a\_field->Trace( trial\_insecticidetreat );

a\_field->SetLastTreatment( trial\_insecticidetreat );

//a\_field->InsectMortality( 0.1 ); // Change this manually if it is really a herbicide

a\_field->SetTramlinesDecay( EL\_TRAMLINE\_DECAYTIME );

return true;

}

|  |  |  |  |  |  |  |  |  |  |  |  |  |  |  |  |  |  |
| --- | --- | --- | --- | --- | --- | --- | --- | --- | --- | --- | --- | --- | --- | --- | --- | --- | --- |
| |  |  |  |  | | --- | --- | --- | --- | | bool Farm::Trial\_PesticideTreat\_GS | ( | LE \* | *a\_field*, | |  |  | double | *a\_user*, | |  |  | int | *a\_days* | |  | ) |  |  | | virtual |

Special pesticide trial functionality.

References EL\_HERBICIDE\_DELAYTIME, EL\_TRAMLINE\_DECAYTIME, LE::GetVegHeight(), LE::ReduceWeedBiomass(), LE::SetHerbicideDelay(), LE::SetLastTreatment(), LE::SetTramlinesDecay(), LE::Trace(), and trial\_insecticidetreat.

{

// Can spray between two certain heights only, but we have to hope that we get called in time for the first so

// right now we only care if we are above a certain height (timing is also important, so must not call this until

// after a ploughing and sowing event

if (a\_field->GetVegHeight() < 3.0) return false;

// All OK so here goes:

a\_field->Trace( trial\_insecticidetreat );

a\_field->SetLastTreatment( trial\_insecticidetreat );

//a\_field->InsectMortality( 0.1 ); // Change this manually if it is really a herbicide

a\_field->ReduceWeedBiomass( 0.05 );

a\_field->SetHerbicideDelay( EL\_HERBICIDE\_DELAYTIME );

a\_field->SetTramlinesDecay( EL\_TRAMLINE\_DECAYTIME );

return true;

}

|  |  |  |  |  |  |  |  |  |  |  |  |  |  |  |  |  |  |
| --- | --- | --- | --- | --- | --- | --- | --- | --- | --- | --- | --- | --- | --- | --- | --- | --- | --- |
| |  |  |  |  | | --- | --- | --- | --- | | bool Farm::Trial\_ToxicControl | ( | LE \* | *a\_field*, | |  |  | double | *a\_user*, | |  |  | int | *a\_days* | |  | ) |  |  | | virtual |

Special pesticide trial functionality.

References EL\_TRAMLINE\_DECAYTIME, LE::InsectMortality(), LE::SetLastTreatment(), LE::SetTramlinesDecay(), LE::Trace(), and trial\_toxiccontrol.

{

a\_field->Trace( trial\_toxiccontrol );

a\_field->SetLastTreatment( trial\_toxiccontrol );

a\_field->InsectMortality( 0.001 );

a\_field->SetTramlinesDecay( EL\_TRAMLINE\_DECAYTIME );

return true;

}

|  |  |  |  |  |  |  |  |  |  |  |  |  |  |  |  |  |  |
| --- | --- | --- | --- | --- | --- | --- | --- | --- | --- | --- | --- | --- | --- | --- | --- | --- | --- |
| |  |  |  |  | | --- | --- | --- | --- | | bool Farm::Water | ( | LE \* | *a\_field*, | |  |  | double | *a\_user*, | |  |  | int | *a\_days* | |  | ) |  |  | | virtual |

Carry out a watering on a\_field.

References DO\_IT\_PROB, g\_landscape\_p, g\_weather, LE::GetUnsprayedMarginPolyRef(), Weather::Raining(), LE::SetLastTreatment(), Landscape::SupplyLEPointer(), LE::Trace(), and water.

{

/\* Turn on this code to avoid watering on heavy soils

int soiltype = a\_field->GetSoilType();

if ( soiltype < 1 || soiltype > 4 )

return true;

\*/

if ( (0 >= a\_days) || (!g\_weather->Raining() && DoIt(DO\_IT\_PROB))) {

a\_field->Trace( water );

a\_field->SetLastTreatment( water );

int pref=a\_field->GetUnsprayedMarginPolyRef();

if (pref!=-1){

// Must have an unsprayed margin so need to pass the information on to it

LE\* um=g\_landscape\_p->SupplyLEPointer(pref);

um->SetLastTreatment( water );

}

return true;

}

return false;

}

|  |  |  |  |  |  |  |  |  |  |  |  |  |  |  |  |  |  |
| --- | --- | --- | --- | --- | --- | --- | --- | --- | --- | --- | --- | --- | --- | --- | --- | --- | --- |
| |  |  |  |  | | --- | --- | --- | --- | | bool Farm::WinterPlough | ( | LE \* | *a\_field*, | |  |  | double | *a\_user*, | |  |  | int | *a\_days* | |  | ) |  |  | | virtual |

Carry out a ploughing event in the winter on a\_field.

References DO\_IT\_PROB, g\_landscape\_p, g\_weather, LE::GetUnsprayedMarginPolyRef(), LE::InsectMortality(), Weather::Raining(), LE::SetLastTreatment(), Landscape::SupplyLEPointer(), LE::Trace(), winter\_plough, and LE::ZeroVeg().

{

if ( (0 >= a\_days) || (!g\_weather->Raining() && DoIt(DO\_IT\_PROB))) {

a\_field->Trace( winter\_plough );

a\_field->SetLastTreatment( winter\_plough );

a\_field->InsectMortality( 0.1 );

a\_field->ZeroVeg();

int pref=a\_field->GetUnsprayedMarginPolyRef();

if (pref!=-1){

// Must have an unsprayed margin so need to pass the information on to it

LE\* um=g\_landscape\_p->SupplyLEPointer(pref);

um->SetLastTreatment( winter\_plough );

um->InsectMortality( 0.1 );

um->ZeroVeg();

}

return true;

}

return false;

}

---

## Member Data Documentation

|  |  |  |
| --- | --- | --- |
| |  | | --- | | AgroChemIndustryCereal\* Farm::m\_agrochemindustrycereal | | protected |

|  |  |  |
| --- | --- | --- |
| |  | | --- | | Carrots\* Farm::m\_carrots | | protected |

|  |  |  |
| --- | --- | --- |
| |  | | --- | | CloverGrassGrazed1\* Farm::m\_CGG1 | | protected |

|  |  |  |
| --- | --- | --- |
| |  | | --- | | CloverGrassGrazed2\* Farm::m\_CGG2 | | protected |

|  |  |  |
| --- | --- | --- |
| |  | | --- | | int Farm::m\_farm\_num | | protected |

Referenced by GetFarmNumber(), and SetFarmNumber().

|  |  |  |
| --- | --- | --- |
| |  | | --- | | TTypesOfFarm Farm::m\_farmtype | | protected |

Referenced by AgroChemIndustryCerealFarm1::AgroChemIndustryCerealFarm1(), AgroChemIndustryCerealFarm2::AgroChemIndustryCerealFarm2(), AgroChemIndustryCerealFarm3::AgroChemIndustryCerealFarm3(), ConventionalCattle::ConventionalCattle(), ConventionalPig::ConventionalPig(), ConventionalPlant::ConventionalPlant(), ConvMarginalJord::ConvMarginalJord(), GetType(), NoPesticideBaseFarm::NoPesticideBaseFarm(), NoPesticideNoPFarm::NoPesticideNoPFarm(), OrganicCattle::OrganicCattle(), OrganicPig::OrganicPig(), OrganicPlant::OrganicPlant(), PesticideTrialControl::PesticideTrialControl(), PesticideTrialToxicControl::PesticideTrialToxicControl(), PesticideTrialTreatment::PesticideTrialTreatment(), UserDefinedFarm1::UserDefinedFarm1(), UserDefinedFarm10::UserDefinedFarm10(), UserDefinedFarm11::UserDefinedFarm11(), UserDefinedFarm12::UserDefinedFarm12(), UserDefinedFarm13::UserDefinedFarm13(), UserDefinedFarm14::UserDefinedFarm14(), UserDefinedFarm15::UserDefinedFarm15(), UserDefinedFarm16::UserDefinedFarm16(), UserDefinedFarm17::UserDefinedFarm17(), UserDefinedFarm18::UserDefinedFarm18(), UserDefinedFarm19::UserDefinedFarm19(), UserDefinedFarm2::UserDefinedFarm2(), UserDefinedFarm20::UserDefinedFarm20(), UserDefinedFarm21::UserDefinedFarm21(), UserDefinedFarm22::UserDefinedFarm22(), UserDefinedFarm23::UserDefinedFarm23(), UserDefinedFarm24::UserDefinedFarm24(), UserDefinedFarm25::UserDefinedFarm25(), UserDefinedFarm26::UserDefinedFarm26(), UserDefinedFarm27::UserDefinedFarm27(), UserDefinedFarm28::UserDefinedFarm28(), UserDefinedFarm29::UserDefinedFarm29(), UserDefinedFarm3::UserDefinedFarm3(), UserDefinedFarm30::UserDefinedFarm30(), UserDefinedFarm31::UserDefinedFarm31(), UserDefinedFarm32::UserDefinedFarm32(), UserDefinedFarm33::UserDefinedFarm33(), UserDefinedFarm34::UserDefinedFarm34(), UserDefinedFarm35::UserDefinedFarm35(), UserDefinedFarm4::UserDefinedFarm4(), UserDefinedFarm5::UserDefinedFarm5(), UserDefinedFarm6::UserDefinedFarm6(), UserDefinedFarm7::UserDefinedFarm7(), UserDefinedFarm8::UserDefinedFarm8(), and UserDefinedFarm9::UserDefinedFarm9().

|  |  |  |
| --- | --- | --- |
| |  | | --- | | FieldPeas\* Farm::m\_fieldpeas | | protected |

|  |  |  |
| --- | --- | --- |
| |  | | --- | | FieldPeasStrigling\* Farm::m\_fieldpeasstrigling | | protected |

|  |  |  |
| --- | --- | --- |
| |  | | --- | | vector< LE\* > Farm::m\_fields | | protected |

Referenced by UserDefinedFarm::AssignPermanentCrop().

|  |  |  |
| --- | --- | --- |
| |  | | --- | | Fodderbeet\* Farm::m\_fodderbeet | | protected |

|  |  |  |
| --- | --- | --- |
| |  | | --- | | FodderGrass\* Farm::m\_foddergrass | | protected |

|  |  |  |
| --- | --- | --- |
| |  | | --- | | int Farm::m\_intensity | | protected |

Referenced by GetIntensity().

|  |  |  |
| --- | --- | --- |
| |  | | --- | | Maize\* Farm::m\_maize | | protected |

|  |  |  |
| --- | --- | --- |
| |  | | --- | | MaizeSilage\* Farm::m\_maizesilage | | protected |

|  |  |  |
| --- | --- | --- |
| |  | | --- | | MaizeStrigling\* Farm::m\_maizestrigling | | protected |

|  |  |  |
| --- | --- | --- |
| |  | | --- | | Oats\* Farm::m\_oats | | protected |

|  |  |  |
| --- | --- | --- |
| |  | | --- | | OBarleyPeaCloverGrass\* Farm::m\_OBarleyPCG | | protected |

|  |  |  |
| --- | --- | --- |
| |  | | --- | | OCarrots\* Farm::m\_ocarrots | | protected |

|  |  |  |
| --- | --- | --- |
| |  | | --- | | OCloverGrassGrazed1\* Farm::m\_OCGG1 | | protected |

|  |  |  |
| --- | --- | --- |
| |  | | --- | | OCloverGrassGrazed2\* Farm::m\_OCGG2 | | protected |

|  |  |  |
| --- | --- | --- |
| |  | | --- | | OCloverGrassSilage1\* Farm::m\_OCGS1 | | protected |

|  |  |  |
| --- | --- | --- |
| |  | | --- | | OFieldPeas\* Farm::m\_ofieldpeas | | protected |

|  |  |  |
| --- | --- | --- |
| |  | | --- | | OFieldPeasSilage\* Farm::m\_ofieldpeassilage | | protected |

|  |  |  |
| --- | --- | --- |
| |  | | --- | | OFirstYearDanger\* Farm::m\_ofirstyeardanger | | protected |

|  |  |  |
| --- | --- | --- |
| |  | | --- | | OGrazingPigs\* Farm::m\_ograzingpigs | | protected |

|  |  |  |
| --- | --- | --- |
| |  | | --- | | OMaizeSilage\* Farm::m\_omaizesilage | | protected |

|  |  |  |
| --- | --- | --- |
| |  | | --- | | OOats\* Farm::m\_ooats | | protected |

|  |  |  |
| --- | --- | --- |
| |  | | --- | | OPermanentGrassGrazed\* Farm::m\_opermgrassgrazed | | protected |

|  |  |  |
| --- | --- | --- |
| |  | | --- | | OPotatoes\* Farm::m\_opotatoes | | protected |

|  |  |  |
| --- | --- | --- |
| |  | | --- | | OSBarleySilage\* Farm::m\_osbarleysilage | | protected |

|  |  |  |
| --- | --- | --- |
| |  | | --- | | OSpringBarley\* Farm::m\_ospringbarley | | protected |

|  |  |  |
| --- | --- | --- |
| |  | | --- | | OSpringBarleyExt\* Farm::m\_ospringbarleyext | | protected |

|  |  |  |
| --- | --- | --- |
| |  | | --- | | OSpringBarleyPigs\* Farm::m\_ospringbarleypigs | | protected |

|  |  |  |
| --- | --- | --- |
| |  | | --- | | OWinterBarley\* Farm::m\_owinterbarley | | protected |

|  |  |  |
| --- | --- | --- |
| |  | | --- | | OWinterBarleyExt\* Farm::m\_owinterbarleyext | | protected |

|  |  |  |
| --- | --- | --- |
| |  | | --- | | OWinterRape\* Farm::m\_owinterrape | | protected |

|  |  |  |
| --- | --- | --- |
| |  | | --- | | OWinterRye\* Farm::m\_owinterrye | | protected |

|  |  |  |
| --- | --- | --- |
| |  | | --- | | OWinterWheatUndersown\* Farm::m\_owinterwheatundersown | | protected |

|  |  |  |
| --- | --- | --- |
| |  | | --- | | OWinterWheatUndersownExt\* Farm::m\_owinterwheatundersownext | | protected |

|  |  |  |
| --- | --- | --- |
| |  | | --- | | PermanentSetAside\* Farm::m\_permanentsetaside | | protected |

|  |  |  |
| --- | --- | --- |
| |  | | --- | | vector<PermCropData> Farm::m\_PermCrops | | protected |

Referenced by UserDefinedFarm::UserDefinedFarm().

|  |  |  |
| --- | --- | --- |
| |  | | --- | | PermanentGrassGrazed\* Farm::m\_permgrassgrazed | | protected |

|  |  |  |
| --- | --- | --- |
| |  | | --- | | PermanentGrassLowYield\* Farm::m\_permgrasslowyield | | protected |

|  |  |  |
| --- | --- | --- |
| |  | | --- | | PermanentGrassTussocky\* Farm::m\_permgrasstussocky | | protected |

|  |  |  |
| --- | --- | --- |
| |  | | --- | | Potatoes\* Farm::m\_potatoes | | protected |

|  |  |  |
| --- | --- | --- |
| |  | | --- | | PotatoesIndustry\* Farm::m\_potatoesindustry | | protected |

|  |  |  |
| --- | --- | --- |
| |  | | --- | | LowPriority< FarmEvent\* > Farm::m\_queue | | protected |

|  |  |  |
| --- | --- | --- |
| |  | | --- | | vector<TTypesOfVegetation> Farm::m\_rotation | | protected |

Referenced by AgroChemIndustryCerealFarm1::AgroChemIndustryCerealFarm1(), AgroChemIndustryCerealFarm2::AgroChemIndustryCerealFarm2(), AgroChemIndustryCerealFarm3::AgroChemIndustryCerealFarm3(), ConventionalCattle::ConventionalCattle(), ConventionalPig::ConventionalPig(), ConventionalPlant::ConventionalPlant(), ConvMarginalJord::ConvMarginalJord(), NoPesticideBaseFarm::NoPesticideBaseFarm(), NoPesticideNoPFarm::NoPesticideNoPFarm(), PesticideTrialControl::PesticideTrialControl(), PesticideTrialToxicControl::PesticideTrialToxicControl(), and UserDefinedFarm::UserDefinedFarm().

|  |  |  |
| --- | --- | --- |
| |  | | --- | | int Farm::m\_rotation\_sync\_index | | protected |

|  |  |  |
| --- | --- | --- |
| |  | | --- | | SpringBarleyCloverGrass\* Farm::m\_sbarleyclovergrass | | protected |

|  |  |  |
| --- | --- | --- |
| |  | | --- | | SeedGrass1\* Farm::m\_seedgrass1 | | protected |

|  |  |  |
| --- | --- | --- |
| |  | | --- | | SeedGrass2\* Farm::m\_seedgrass2 | | protected |

|  |  |  |
| --- | --- | --- |
| |  | | --- | | SetAside\* Farm::m\_setaside | | protected |

|  |  |  |
| --- | --- | --- |
| |  | | --- | | SpringBarley\* Farm::m\_springbarley | | protected |

|  |  |  |
| --- | --- | --- |
| |  | | --- | | SpringBarleyCloverGrassStrigling\* Farm::m\_springbarleyclovergrassstrigling | | protected |

|  |  |  |
| --- | --- | --- |
| |  | | --- | | SpringBarleyPeaCloverGrassStrigling\* Farm::m\_springbarleypeaclovergrassstrigling | | protected |

|  |  |  |
| --- | --- | --- |
| |  | | --- | | SpringBarleyPTreatment\* Farm::m\_springbarleyptreatment | | protected |

|  |  |  |
| --- | --- | --- |
| |  | | --- | | SpringBarleySeed\* Farm::m\_springbarleyseed | | protected |

|  |  |  |
| --- | --- | --- |
| |  | | --- | | SpringBarleySilage\* Farm::m\_springbarleysilage | | protected |

|  |  |  |
| --- | --- | --- |
| |  | | --- | | SpringBarleySKManagement\* Farm::m\_springbarleyskmanagement | | protected |

|  |  |  |
| --- | --- | --- |
| |  | | --- | | SpringBarleyStrigling\* Farm::m\_springbarleystrigling | | protected |

|  |  |  |
| --- | --- | --- |
| |  | | --- | | SpringBarleyStriglingCulm\* Farm::m\_springbarleystriglingculm | | protected |

|  |  |  |
| --- | --- | --- |
| |  | | --- | | SpringBarleyStriglingSingle\* Farm::m\_springbarleystriglingsingle | | protected |

|  |  |  |
| --- | --- | --- |
| |  | | --- | | SpringRape\* Farm::m\_springrape | | protected |

|  |  |  |
| --- | --- | --- |
| |  | | --- | | bool Farm::m\_stockfarmer | | protected |

Referenced by AgroChemIndustryCerealFarm1::AgroChemIndustryCerealFarm1(), AgroChemIndustryCerealFarm2::AgroChemIndustryCerealFarm2(), AgroChemIndustryCerealFarm3::AgroChemIndustryCerealFarm3(), ConventionalCattle::ConventionalCattle(), ConventionalPig::ConventionalPig(), ConventionalPlant::ConventionalPlant(), ConvMarginalJord::ConvMarginalJord(), IsStockFarmer(), MakeStockFarmer(), ConventionalPlant::MakeStockFarmer(), OrganicPlant::MakeStockFarmer(), PesticideTrialControl::MakeStockFarmer(), PesticideTrialToxicControl::MakeStockFarmer(), PesticideTrialTreatment::MakeStockFarmer(), NoPesticideBaseFarm::NoPesticideBaseFarm(), NoPesticideNoPFarm::NoPesticideNoPFarm(), OrganicCattle::OrganicCattle(), OrganicPig::OrganicPig(), OrganicPlant::OrganicPlant(), PesticideTrialControl::PesticideTrialControl(), PesticideTrialToxicControl::PesticideTrialToxicControl(), PesticideTrialTreatment::PesticideTrialTreatment(), UserDefinedFarm::UserDefinedFarm(), UserDefinedFarm1::UserDefinedFarm1(), UserDefinedFarm10::UserDefinedFarm10(), UserDefinedFarm11::UserDefinedFarm11(), UserDefinedFarm12::UserDefinedFarm12(), UserDefinedFarm13::UserDefinedFarm13(), UserDefinedFarm14::UserDefinedFarm14(), UserDefinedFarm15::UserDefinedFarm15(), UserDefinedFarm16::UserDefinedFarm16(), UserDefinedFarm17::UserDefinedFarm17(), UserDefinedFarm2::UserDefinedFarm2(), UserDefinedFarm3::UserDefinedFarm3(), UserDefinedFarm4::UserDefinedFarm4(), UserDefinedFarm5::UserDefinedFarm5(), UserDefinedFarm6::UserDefinedFarm6(), UserDefinedFarm7::UserDefinedFarm7(), UserDefinedFarm8::UserDefinedFarm8(), and UserDefinedFarm9::UserDefinedFarm9().

|  |  |  |
| --- | --- | --- |
| |  | | --- | | Triticale\* Farm::m\_triticale | | protected |

|  |  |  |
| --- | --- | --- |
| |  | | --- | | WinterBarley\* Farm::m\_winterbarley | | protected |

|  |  |  |
| --- | --- | --- |
| |  | | --- | | WinterBarleyStrigling\* Farm::m\_winterbarleystrigling | | protected |

|  |  |  |
| --- | --- | --- |
| |  | | --- | | WinterRape\* Farm::m\_winterrape | | protected |

|  |  |  |
| --- | --- | --- |
| |  | | --- | | WinterRapeStrigling\* Farm::m\_winterrapestrigling | | protected |

|  |  |  |
| --- | --- | --- |
| |  | | --- | | WinterRye\* Farm::m\_winterrye | | protected |

|  |  |  |
| --- | --- | --- |
| |  | | --- | | WinterRyeStrigling\* Farm::m\_winterryestrigling | | protected |

|  |  |  |
| --- | --- | --- |
| |  | | --- | | WinterWheat\* Farm::m\_winterwheat | | protected |

|  |  |  |
| --- | --- | --- |
| |  | | --- | | WinterWheatStrigling\* Farm::m\_winterwheatstrigling | | protected |

|  |  |  |
| --- | --- | --- |
| |  | | --- | | WinterWheatStriglingCulm\* Farm::m\_winterwheatstriglingculm | | protected |

|  |  |  |
| --- | --- | --- |
| |  | | --- | | WinterWheatStriglingSingle\* Farm::m\_winterwheatstriglingsingle | | protected |

|  |  |  |
| --- | --- | --- |
| |  | | --- | | WWheatPControl\* Farm::m\_wwheatpcontrol | | protected |

|  |  |  |
| --- | --- | --- |
| |  | | --- | | WWheatPToxicControl\* Farm::m\_wwheatptoxiccontrol | | protected |

|  |  |  |
| --- | --- | --- |
| |  | | --- | | WWheatPTreatment\* Farm::m\_wwheatptreatment | | protected |

|  |  |  |
| --- | --- | --- |
| |  | | --- | | YoungForestCrop\* Farm::m\_youngforest | | protected |

---

The documentation for this class was generated from the following files:

- farm.h
- farm.cpp
- farmfuncs.cpp


- Farm
- Generated on Thu Jan 10 2013 13:15:36 for ALMaSS Skylark ODdox by
   1.8.1.1
