# Supplementary material for: Modelling Skylarks (Alauda arvensis) to Predict Impacts of Changes in Land Management and Policy: Development and Testing of an Agent-Based Model
Source: PLoS One. 2013 Jun 6;8(6):e65803. doi: 10.1371/journal.pone.0065803 (PMC3675089; doi:10.1371/journal.pone.0065803)
Supplement: Supporting Information S4 — The skylark ODdox as a zipped archive. (ZIP) [file pone.0065803.s004.zip › Skylark_ODdox/class_farm_manager.html]

ALMaSS Skylark ODdox: FarmManager Class Reference


|  |
| --- |
| ALMaSS Skylark ODdox  2.0 |


- Main Page
- Related Pages
- Classes
- Files

- Class List
- Class Index
- Class Hierarchy
- Class Members

Public Member Functions |
Protected Member Functions |
Protected Attributes

FarmManager Class Reference

The Farm Manager class.
More...

`#include <farm.h>`

List of all members.

|  |  |
| --- | --- |
| Public Member Functions | |
| void | AddField (int a\_OwnerIndex, LE \*a\_newland, int a\_Owner) |
| int | ConnectFarm (int a\_owner) |
| void | FarmManagement () |
|  | FarmManager () |
| int | GetFarmIndex (int a\_owner) |
| void | InitiateManagement (void) |
| void | RemoveField (int a\_OwnerIndex, LE \*a\_field) |
|  | ~FarmManager () |

|  |  |
| --- | --- |
| Protected Member Functions | |
| void | CreateFarms (const char \*a\_farmfile) |

|  |  |
| --- | --- |
| Protected Attributes | |
| vector< Farm \* > | m\_farms |

---

## Detailed Description

The Farm Manager class.

---

## Constructor & Destructor Documentation

|  |  |  |  |  |
| --- | --- | --- | --- | --- |
| FarmManager::FarmManager | ( |  | ) |  |

References CreateFarms(), l\_map\_farmref\_file, l\_map\_read\_farmfile, CfgBool::value(), and CfgStr::value().

{

if ( l\_map\_read\_farmfile.value() ) {

CreateFarms( l\_map\_farmref\_file.value() );

}

}

|  |  |  |  |  |
| --- | --- | --- | --- | --- |
| FarmManager::~FarmManager | ( |  | ) |  |

References m\_farms.

{

for ( unsigned int i = 0; i < m\_farms.size(); i++ )

delete m\_farms[ i ];

}

---

## Member Function Documentation

|  |  |  |  |
| --- | --- | --- | --- |
| void FarmManager::AddField | ( | int | *a\_OwnerIndex*, |
|  |  | LE \* | *a\_newland*, |
|  |  | int | *a\_Owner* |
|  | ) |  |  |

References m\_farms, and LE::SetOwner().

Referenced by Landscape::ChangeMapMapping(), and Landscape::ReadPolys().

{

m\_farms[ a\_OwnerIndex ]->AddField( a\_newland );

a\_newland->SetOwner( m\_farms[ a\_OwnerIndex ], a\_Owner, a\_OwnerIndex );

}

|  |  |  |  |  |  |
| --- | --- | --- | --- | --- | --- |
| int FarmManager::ConnectFarm | ( | int | *a\_owner* | ) |  |

References g\_msg, m\_farms, MapErrorMsg::Warn(), and WARN\_FILE.

Referenced by Landscape::ChangeMapMapping(), and Landscape::ReadPolys().

{

for ( unsigned int i = 0; i < m\_farms.size(); i++ )

{

if ( a\_owner == m\_farms[ i ]->GetFarmNumber() )

{

// Found it. Set mapping and return.

return i;

}

}

// We didn't find the owner in the list of farms,

// pregenerated in CreateFarms() above. Something

// is not correct here, so raise an appropriate

// error and exit.

char error\_num[ 20 ];

sprintf( error\_num, "%d", a\_owner );

g\_msg->Warn( WARN\_FILE, "FarmManager::ConnectFarm(): Unknown farm number"" referenced in polygon file:", error\_num );

exit( 1 );

}

|  |  |  |  |  |  |  |  |
| --- | --- | --- | --- | --- | --- | --- | --- |
| |  |  |  |  |  |  | | --- | --- | --- | --- | --- | --- | | void FarmManager::CreateFarms | ( | const char \* | *a\_farmfile* | ) |  | | protected |

References g\_farm\_fixed\_rotation\_enable, g\_farm\_fixed\_rotation\_farmtype, g\_msg, m\_farms, CfgInt::value(), CfgBool::value(), MapErrorMsg::Warn(), and WARN\_FILE.

Referenced by FarmManager().

{

int No, FType, NoFarms;

FILE \* inpf = fopen(a\_farmfile, "r" );

if (!inpf) {

g\_msg->Warn( WARN\_FILE, "Landscape::CreateFarms(): Unable to open file", a\_farmfile );

exit( 1 );

}

fscanf( inpf, "%d", & NoFarms );

m\_farms.resize( NoFarms );

for ( int i = 0; i < NoFarms; i++ )

{

// File format:

//

// Two colunms of numbers 0..number of farms, 0-number of farmtypes-1

// the second column determines what type of farm we have

fscanf( inpf, "%d %d", & No, & FType );

/\*

//If we are testing crop management, then ignore farm type from

// the file and set to fixed one instead.

if ( g\_farm\_test\_crop.value() ) {

FType = g\_farm\_test\_crop\_farmtype.value();

}

\*/

// If we are running in fixed, sync'ed rotation mode, set all farms to

// be of the requested type.

if ( g\_farm\_fixed\_rotation\_enable.value() )

{

FType = g\_farm\_fixed\_rotation\_farmtype.value();

}

switch ( FType ) {

case 0:

m\_farms[ i ] = new ConventionalCattle;

break;

case 1:

m\_farms[ i ] = new ConventionalPig;

break;

case 2:

m\_farms[ i ] = new ConventionalPlant;

break;

case 3:

m\_farms[ i ] = new OrganicCattle;

break;

case 4:

m\_farms[ i ] = new OrganicPig;

break;

case 5:

m\_farms[ i ] = new OrganicPlant;

break;

case 6:

m\_farms[ i ] = new PesticideTrialControl;

break;

case 7:

m\_farms[ i ] = new PesticideTrialToxicControl;

break;

case 8:

m\_farms[ i ] = new PesticideTrialTreatment;

break;

case 9:

m\_farms[ i ] = new ConvMarginalJord;

break;

case 10:

m\_farms[ i ] = new AgroChemIndustryCerealFarm1;

break;

case 11:

m\_farms[ i ] = new AgroChemIndustryCerealFarm2;

break;

case 12:

m\_farms[ i ] = new AgroChemIndustryCerealFarm3;

break;

case 13:

m\_farms[ i ] = new NoPesticideBaseFarm;

break;

case 14:

m\_farms[ i ] = new NoPesticideNoPFarm;

break;

case 15:

m\_farms[ i ] = new UserDefinedFarm1;

break;

case 16:

m\_farms[ i ] = new UserDefinedFarm2;

break;

case 17:

m\_farms[ i ] = new UserDefinedFarm3;

break;

case 18:

m\_farms[ i ] = new UserDefinedFarm4;

break;

case 19:

m\_farms[ i ] = new UserDefinedFarm5;

break;

case 20:

m\_farms[ i ] = new UserDefinedFarm6;

break;

case 21:

m\_farms[ i ] = new UserDefinedFarm7;

break;

case 22:

m\_farms[ i ] = new UserDefinedFarm8;

break;

case 23:

m\_farms[ i ] = new UserDefinedFarm9;

break;

case 24:

m\_farms[ i ] = new UserDefinedFarm10;

break;

case 25:

m\_farms[ i ] = new UserDefinedFarm11;

break;

case 26:

m\_farms[ i ] = new UserDefinedFarm12;

break;

case 27:

m\_farms[ i ] = new UserDefinedFarm13;

break;

case 28:

m\_farms[ i ] = new UserDefinedFarm14;

break;

case 29:

m\_farms[ i ] = new UserDefinedFarm15;

break;

case 30:

m\_farms[ i ] = new UserDefinedFarm16;

break;

case 31:

m\_farms[ i ] = new UserDefinedFarm17;

break;

// NB the user defing farms below require an extra parameter in the rotation file denoting the intensity (0 or 1 = high low)

case 32:

m\_farms[ i ] = new UserDefinedFarm18("UserDefinedFarm18.rot");

break;

case 33:

m\_farms[ i ] = new UserDefinedFarm19("UserDefinedFarm19.rot");

break;

case 34:

m\_farms[ i ] = new UserDefinedFarm20("UserDefinedFarm20.rot");

break;

case 35:

m\_farms[ i ] = new UserDefinedFarm21("UserDefinedFarm21.rot");

break;

case 36:

m\_farms[ i ] = new UserDefinedFarm22("UserDefinedFarm22.rot");

break;

case 37:

m\_farms[ i ] = new UserDefinedFarm23("UserDefinedFarm23.rot");

break;

case 38:

m\_farms[ i ] = new UserDefinedFarm24("UserDefinedFarm24.rot");

break;

case 39:

m\_farms[ i ] = new UserDefinedFarm25("UserDefinedFarm25.rot");

break;

case 40:

m\_farms[ i ] = new UserDefinedFarm26("UserDefinedFarm26.rot");

break;

case 41:

m\_farms[ i ] = new UserDefinedFarm27("UserDefinedFarm27.rot");

break;

case 42:

m\_farms[ i ] = new UserDefinedFarm28("UserDefinedFarm28.rot");

break;

case 43:

m\_farms[ i ] = new UserDefinedFarm29("UserDefinedFarm29.rot");

break;

case 44:

m\_farms[ i ] = new UserDefinedFarm30("UserDefinedFarm30.rot");

break;

case 45:

m\_farms[ i ] = new UserDefinedFarm31("UserDefinedFarm31.rot");

break;

case 46:

m\_farms[ i ] = new UserDefinedFarm32("UserDefinedFarm32.rot");

break;

case 47:

m\_farms[ i ] = new UserDefinedFarm33("UserDefinedFarm33.rot");

break;

case 48:

m\_farms[ i ] = new UserDefinedFarm34("UserDefinedFarm34.rot");

break;

case 49:

m\_farms[ i ] = new UserDefinedFarm35("UserDefinedFarm35.rot");

break;

default:

g\_msg->Warn( WARN\_FILE, "Landscape::CreateFarms(): Unknown farm ""type reference number", "" );

exit( 1 );

}

m\_farms[ i ]->SetFarmNumber( No );

}

fclose( inpf );

// Sort m\_farms w.r.t. farm number for fast searching

sort( m\_farms.begin(), m\_farms.end(), CompareFarmNum());

}

|  |  |  |  |  |
| --- | --- | --- | --- | --- |
| void FarmManager::FarmManagement | ( |  | ) |  |

References m\_farms.

Referenced by Landscape::Tick().

{

for ( unsigned int i = 0; i < m\_farms.size(); i++ )

{

m\_farms[ i ]->Management();

}

}

|  |  |  |  |  |  |
| --- | --- | --- | --- | --- | --- |
| int FarmManager::GetFarmIndex | ( | int | *a\_owner* | ) |  |

|  |  |  |  |  |  |
| --- | --- | --- | --- | --- | --- |
| void FarmManager::InitiateManagement | ( | void |  | ) |  |

References m\_farms.

Referenced by Landscape::Landscape().

{

for ( unsigned int i = 0; i < m\_farms.size(); i++ )

{

m\_farms[ i ]->InitiateManagement();

}

}

|  |  |  |  |
| --- | --- | --- | --- |
| void FarmManager::RemoveField | ( | int | *a\_OwnerIndex*, |
|  |  | LE \* | *a\_field* |
|  | ) |  |  |

References m\_farms.

Referenced by Landscape::ChangeMapMapping().

{

m\_farms[ a\_OwnerIndex ]->RemoveField( a\_field );

}

---

## Member Data Documentation

|  |  |  |
| --- | --- | --- |
| |  | | --- | | vector<Farm\*> FarmManager::m\_farms | | protected |

Referenced by AddField(), ConnectFarm(), CreateFarms(), FarmManagement(), InitiateManagement(), RemoveField(), and ~FarmManager().

---

The documentation for this class was generated from the following files:

- farm.h
- farm.cpp


- FarmManager
- Generated on Thu Jan 10 2013 13:15:36 for ALMaSS Skylark ODdox by
   1.8.1.1
