# Supplementary material for: Modelling Skylarks (Alauda arvensis) to Predict Impacts of Changes in Land Management and Policy: Development and Testing of an Agent-Based Model
Source: PLoS One. 2013 Jun 6;8(6):e65803. doi: 10.1371/journal.pone.0065803 (PMC3675089; doi:10.1371/journal.pone.0065803)
Supplement: Supporting Information S4 — The skylark ODdox as a zipped archive. (ZIP) [file pone.0065803.s004.zip › Skylark_ODdox/class_garden-members.html]

ALMaSS Skylark ODdox: Member List


|  |
| --- |
| ALMaSS Skylark ODdox  2.0 |


- Main Page
- Related Pages
- Classes
- Files

- Class List
- Class Index
- Class Hierarchy
- Class Members

Garden Member List

This is the complete list of members for Garden, including all inherited members.

|  |  |  |
| --- | --- | --- |
| AddArea(double a\_area\_diff) | LE | inline |
| BumpRunNum(void) | LE | inline |
| DoDevelopment(void) | LE | virtual |
| ForceGrowthDevelopment(void) | LE | inlinevirtual |
| ForceGrowthInitialize(void) | LE | inlinevirtual |
| ForceGrowthTest(void) | LE | inlinevirtual |
| Garden(void) | Garden |  |
| GetArea(void) | LE | inline |
| GetBorder(void) | LE | inline |
| GetCattleGrazing(void) | LE | inline |
| GetCentroidX() | LE | inlinevirtual |
| GetCentroidY() | LE | inlinevirtual |
| GetCountryDesignation(void) | LE | inline |
| GetDayDegrees(void) | LE | inlinevirtual |
| GetDeadBiomass(void) | LE | inlinevirtual |
| GetDigestability(void) | LE | inlinevirtual |
| GetElementType(void) | LE | inlinevirtual |
| GetFileType(void) | LE | inline |
| GetGreenBiomass(void) | LE | inlinevirtual |
| GetHigh(void) | LE | inline |
| GetInsectPop(void) | LE | inlinevirtual |
| GetLAGreen(void) | LE | inlinevirtual |
| GetLastTreatment(int \*a\_index) | LE |  |
| GetLATotal(void) | LE | inlinevirtual |
| GetMapIndex(void) | LE | inline |
| GetMapValid(void) | LE | inline |
| GetMaxX(void) | LE | inline |
| GetMaxY(void) | LE | inline |
| GetMConstants(int a) | LE | inline |
| GetMDates(int a, int b) | LE | inline |
| GetMgtLoopDetectCount(void) | LE | inline |
| GetMgtLoopDetectDate(void) | LE | inline |
| GetMinX(void) | LE | inline |
| GetMinY(void) | LE | inline |
| GetOldDays(void) | LE | inline |
| GetOwner(void) | LE | inline |
| GetOwnerFile(void) | LE | inline |
| GetOwnerIndex(void) | LE | inline |
| GetPesticideCell() | LE | inline |
| GetPigGrazing(void) | LE | inline |
| GetPoison(void) | LE | inline |
| GetPoly(void) | LE | inline |
| GetRotIndex(void) | LE | inline |
| GetRunNum(void) | LE | inline |
| GetSignal(void) | LE | inline |
| GetSkScrapes(void) | LE | inlinevirtual |
| GetSoilType() | LE | inline |
| GetSubType(void) | LE | inline |
| GetTrafficLoad(void) | LE | inlinevirtual |
| GetUnsprayedMarginPolyRef(void) | LE | inline |
| GetValidX(void) | LE | inline |
| GetValidY(void) | LE | inline |
| GetVegAge() | LE | inline |
| GetVegBiomass(void) | LE | inlinevirtual |
| GetVegCover(void) | LE | inlinevirtual |
| GetVegDensity(void) | LE | inlinevirtual |
| GetVegHeight(void) | LE | inlinevirtual |
| GetVegPatchy(void) | LE | inlinevirtual |
| GetVegStore(void) | LE | inline |
| GetVegType(void) | LE | inlinevirtual |
| GetWeedBiomass(void) | LE | inlinevirtual |
| HasTramlines(void) | LE | inline |
| Insecticide(double) | LE | inlinevirtual |
| InsectMortality(double) | LE | inlinevirtual |
| IsRecentlyMown(void) | LE | inline |
| IsRecentlySprayed(void) | LE | inline |
| LE(void) | LE |  |
| m\_area | LE | protected |
| m\_border | LE | protected |
| m\_cattle\_grazing | LE | protected |
| m\_centroidx | LE | protected |
| m\_centroidy | LE | protected |
| m\_countrydesignation | LE | protected |
| m\_days\_since\_insecticide\_spray | LE | protected |
| m\_ddegs | LE | protected |
| m\_farmfunc\_tried\_to\_do | LE | protected |
| m\_file\_type | LE | protected |
| m\_herbicidedelay | LE | protected |
| m\_high | LE | protected |
| m\_is\_in\_map | LE | protected |
| m\_largeroad\_load | LE | protectedstatic |
| m\_lastindex | LE | protected |
| m\_lasttreat | LE | protected |
| m\_management\_loop\_detect\_count | LE | protected |
| m\_management\_loop\_detect\_date | LE | protected |
| m\_map\_index | LE | protected |
| m\_maxx | LE | protected |
| m\_maxy | LE | protected |
| m\_minx | LE | protected |
| m\_miny | LE | protected |
| m\_monthly\_traffic | LE | protectedstatic |
| m\_mowndecay | LE | protected |
| m\_olddays | LE | protected |
| m\_owner | LE | protected |
| m\_owner\_file | LE | protected |
| m\_owner\_index | LE | protected |
| m\_PesticideGridCell | LE | protected |
| m\_pig\_grazing | LE | protected |
| m\_poison | LE | protected |
| m\_poly | LE | protected |
| m\_repeat\_start | LE |  |
| m\_rot\_index | LE | protected |
| m\_running | LE | protected |
| m\_signal\_mask | LE | protected |
| m\_skylarkscrapes | LE |  |
| m\_smallroad\_load | LE | protectedstatic |
| m\_soiltype | LE | protected |
| m\_squares\_in\_map | LE |  |
| m\_subtype | LE | protected |
| m\_tramlinesdecay | LE | protected |
| m\_tried\_to\_do | LE |  |
| m\_type | LE | protected |
| m\_unsprayedmarginpolyref | LE | protected |
| m\_user | LE |  |
| m\_valid\_x | LE | protected |
| m\_valid\_y | LE | protected |
| m\_vegage | LE | protected |
| m\_vege\_danger\_store | LE | protected |
| m\_yddegs | LE | protected |
| MConsts | LE | protected |
| MDates | LE | protected |
| NonVegElement(void) | NonVegElement |  |
| RecalculateBugsNStuff(void) | LE | inlinevirtual |
| ReduceVeg(double) | LE | inlinevirtual |
| ReduceVeg\_Extended(double) | LE | inlinevirtual |
| ReduceWeedBiomass(double) | LE | inlinevirtual |
| ResetTrace(void) | LE |  |
| SetArea(double a\_area) | LE | inline |
| SetBorder(LE \*a\_border) | LE | inline |
| SetCentroid(int x, int y) | LE | inlinevirtual |
| SetCopyTreatment(int a\_treatment) | LE |  |
| SetCountryDesignation(int a\_designation) | LE | inline |
| SetCropData(double, double, double, TTypesOfVegetation, double, bool) | LE | inlinevirtual |
| SetCropDataAll(double, double, double, double, TTypesOfVegetation, double, double, bool, double, bool, double) | LE | inlinevirtual |
| SetElementType(TTypesOfLandscapeElement a\_type) | LE | inline |
| SetFileType(int a\_file\_type) | LE | inline |
| SetGrowthPhase(int) | LE | inlinevirtual |
| SetHerbicideDelay(int a\_decaytime\_days) | LE | inline |
| SetHigh(bool a\_high) | LE | inline |
| SetInsectPop(double) | LE | inlinevirtual |
| SetLastTreatment(int a\_treatment) | LE |  |
| SetMapIndex(int a\_map\_index) | LE | inline |
| SetMapValid(bool a\_valid) | LE | inline |
| SetMaxX(int x) | LE | inline |
| SetMaxY(int y) | LE | inline |
| SetMConstants(int a, int c) | LE | inline |
| SetMDates(int a, int b, int c) | LE | inline |
| SetMgtLoopDetectCount(long a\_num) | LE | inline |
| SetMgtLoopDetectDate(long a\_num) | LE | inline |
| SetMinX(int x) | LE | inline |
| SetMinY(int y) | LE | inline |
| SetMownDecay(int a\_decaytime\_days) | LE | inline |
| SetOldDays(long a\_days) | LE | inline |
| SetOwner(Farm \*a\_owner, int a\_owner\_num, int a\_owner\_index) | LE | inline |
| SetPesticideCell(int a\_cell) | LE | inline |
| SetPoison(bool a\_poison) | LE | inline |
| SetPoly(int a\_poly) | LE | inline |
| SetRotIndex(int a\_index) | LE | inline |
| SetSignal(LE\_Signal a\_signal) | LE | inline |
| SetSoilType(int a\_st) | LE | inline |
| SetSubType(int a\_subtype) | LE | inline |
| SetTramlinesDecay(int a\_decaytime\_days) | LE | inline |
| SetUnsprayedMarginPolyRef(int a\_unsprayedmargin) | LE | inline |
| SetValidXY(int a\_valid\_x, int a\_valid\_y) | LE | inline |
| SetVegBiomass(int) | LE | inlinevirtual |
| SetVegHeight(double, double, double, double) | LE | inlinevirtual |
| SetVegPatchy(bool) | LE | inlinevirtual |
| SetVegStore(int a\_veg) | LE | inline |
| SetVegType(TTypesOfVegetation, TTypesOfVegetation) | LE | inlinevirtual |
| StoreLAItotal() | LE | inlinevirtual |
| Tick(void) | LE |  |
| ToggleCattleGrazing(void) | LE | inlinevirtual |
| TogglePigGrazing(void) | LE | inlinevirtual |
| Trace(int a\_value) | LE |  |
| ZeroVeg(void) | LE | inlinevirtual |
| ZeroVegAge() | LE | inline |
| ~LE(void) | LE | virtual |


- Generated on Thu Jan 10 2013 13:15:36 for ALMaSS Skylark ODdox by
   1.8.1.1
