# Supplementary material for: Modelling Skylarks (Alauda arvensis) to Predict Impacts of Changes in Land Management and Policy: Development and Testing of an Agent-Based Model
Source: PLoS One. 2013 Jun 6;8(6):e65803. doi: 10.1371/journal.pone.0065803 (PMC3675089; doi:10.1371/journal.pone.0065803)
Supplement: Supporting Information S4 — The skylark ODdox as a zipped archive. (ZIP) [file pone.0065803.s004.zip › Skylark_ODdox/class_i_d_map.html]

ALMaSS Skylark ODdox: IDMap Class Reference


|  |
| --- |
| ALMaSS Skylark ODdox  2.0 |


- Main Page
- Related Pages
- Classes
- Files

- Class List
- Class Index
- Class Hierarchy
- Class Members

Public Member Functions |
Public Attributes

IDMap Class Reference

Used to map locations of animals in space.
More...

`#include <MovementMap.h>`

List of all members.

|  |  |
| --- | --- |
| Public Member Functions | |
| void | ClearMapValue (unsigned x, unsigned y) |
| TAnimal \* | GetMapValue (unsigned x, unsigned y) |
|  | IDMap (Landscape \*L) |
| void | SetMapValue (unsigned x, unsigned y, TAnimal \*p) |
|  | ~IDMap () |

|  |  |
| --- | --- |
| Public Attributes | |
| TAnimal \*\* | m\_TheMap |
| int | maxx |
| int | maxy |

---

## Detailed Description

Used to map locations of animals in space.

---

## Constructor & Destructor Documentation

|  |  |  |  |  |  |
| --- | --- | --- | --- | --- | --- |
| IDMap::IDMap | ( | Landscape \* | *L* | ) |  |

References m\_TheMap, maxx, maxy, SetMapValue(), Landscape::SupplySimAreaHeight(), and Landscape::SupplySimAreaWidth().

{

maxx=L->SupplySimAreaWidth();

maxy=L->SupplySimAreaHeight();

m\_TheMap = new TAnimal\*[ maxx\*maxy ];

for (int y=0; y<maxx; y++)

{

for (int x=0; x<maxy; x++)

{

SetMapValue(x,y,NULL);

}

}

}

|  |  |  |  |  |
| --- | --- | --- | --- | --- |
| IDMap::~IDMap | ( |  | ) |  |

References m\_TheMap.

{

delete m\_TheMap;

}

---

## Member Function Documentation

|  |  |  |  |  |  |  |  |  |  |  |  |  |  |
| --- | --- | --- | --- | --- | --- | --- | --- | --- | --- | --- | --- | --- | --- |
| |  |  |  |  | | --- | --- | --- | --- | | void IDMap::ClearMapValue | ( | unsigned | *x*, | |  |  | unsigned | *y* | |  | ) |  |  | | inline |

References m\_TheMap, and maxx.

{m\_TheMap[x+(maxx\*y)] = NULL;}

|  |  |  |  |  |  |  |  |  |  |  |  |  |  |
| --- | --- | --- | --- | --- | --- | --- | --- | --- | --- | --- | --- | --- | --- |
| |  |  |  |  | | --- | --- | --- | --- | | TAnimal\* IDMap::GetMapValue | ( | unsigned | *x*, | |  |  | unsigned | *y* | |  | ) |  |  | | inline |

References m\_TheMap, and maxx.

{

return m\_TheMap[x+(maxx\*y)];

}

|  |  |  |  |  |  |  |  |  |  |  |  |  |  |  |  |  |  |
| --- | --- | --- | --- | --- | --- | --- | --- | --- | --- | --- | --- | --- | --- | --- | --- | --- | --- |
| |  |  |  |  | | --- | --- | --- | --- | | void IDMap::SetMapValue | ( | unsigned | *x*, | |  |  | unsigned | *y*, | |  |  | TAnimal \* | *p* | |  | ) |  |  | | inline |

References m\_TheMap, and maxx.

Referenced by IDMap().

{

m\_TheMap[x+(maxx\*y)] = p;

}

---

## Member Data Documentation

|  |
| --- |
| TAnimal\*\* IDMap::m\_TheMap |

Referenced by ClearMapValue(), GetMapValue(), IDMap(), SetMapValue(), and ~IDMap().

|  |
| --- |
| int IDMap::maxx |

Referenced by ClearMapValue(), GetMapValue(), IDMap(), and SetMapValue().

|  |
| --- |
| int IDMap::maxy |

Referenced by IDMap().

---

The documentation for this class was generated from the following files:

- MovementMap.h
- MovementMap.cpp


- IDMap
- Generated on Thu Jan 10 2013 13:15:36 for ALMaSS Skylark ODdox by
   1.8.1.1
