# Supplementary material for: Modelling Skylarks (Alauda arvensis) to Predict Impacts of Changes in Land Management and Policy: Development and Testing of an Agent-Based Model
Source: PLoS One. 2013 Jun 6;8(6):e65803. doi: 10.1371/journal.pone.0065803 (PMC3675089; doi:10.1371/journal.pone.0065803)
Supplement: Supporting Information S4 — The skylark ODdox as a zipped archive. (ZIP) [file pone.0065803.s004.zip › Skylark_ODdox/class_l_e.html]

ALMaSS Skylark ODdox: LE Class Reference


|  |
| --- |
| ALMaSS Skylark ODdox  2.0 |


- Main Page
- Related Pages
- Classes
- Files

- Class List
- Class Index
- Class Hierarchy
- Class Members

Public Member Functions |
Public Attributes |
Protected Attributes |
Static Protected Attributes

LE Class Reference

`#include <elements.h>`

List of all members.

|  |  |
| --- | --- |
| Public Member Functions | |
| void | AddArea (double a\_area\_diff) |
| void | BumpRunNum (void) |
| virtual void | DoDevelopment (void) |
| virtual void | ForceGrowthDevelopment (void) |
| virtual void | ForceGrowthInitialize (void) |
| virtual void | ForceGrowthTest (void) |
| double | GetArea (void) |
| LE \* | GetBorder (void) |
| bool | GetCattleGrazing (void) |
| virtual int | GetCentroidX () |
| virtual int | GetCentroidY () |
| int | GetCountryDesignation (void) |
| virtual double | GetDayDegrees (void) |
| virtual double | GetDeadBiomass (void) |
| virtual double | GetDigestability (void) |
| virtual TTypesOfLandscapeElement | GetElementType (void) |
| int | GetFileType (void) |
| virtual double | GetGreenBiomass (void) |
| bool | GetHigh (void) |
| virtual double | GetInsectPop (void) |
| virtual double | GetLAGreen (void) |
| int | GetLastTreatment (int \*a\_index) |
| virtual double | GetLATotal (void) |
| int | GetMapIndex (void) |
| bool | GetMapValid (void) |
| int | GetMaxX (void) |
| int | GetMaxY (void) |
| int | GetMConstants (int a) |
| int | GetMDates (int a, int b) |
| long | GetMgtLoopDetectCount (void) |
| long | GetMgtLoopDetectDate (void) |
| int | GetMinX (void) |
| int | GetMinY (void) |
| long | GetOldDays (void) |
| Farm \* | GetOwner (void) |
| int | GetOwnerFile (void) |
| int | GetOwnerIndex (void) |
| int | GetPesticideCell () |
| bool | GetPigGrazing (void) |
| bool | GetPoison (void) |
| int | GetPoly (void) |
| int | GetRotIndex (void) |
| long | GetRunNum (void) |
| LE\_Signal | GetSignal (void) |
| virtual bool | GetSkScrapes (void) |
| int | GetSoilType () |
| int | GetSubType (void) |
| virtual double | GetTrafficLoad (void) |
| int | GetUnsprayedMarginPolyRef (void) |
| int | GetValidX (void) |
| int | GetValidY (void) |
| int | GetVegAge () |
| virtual double | GetVegBiomass (void) |
| virtual double | GetVegCover (void) |
| virtual int | GetVegDensity (void) |
| virtual double | GetVegHeight (void) |
| virtual bool | GetVegPatchy (void) |
| int | GetVegStore (void) |
| virtual TTypesOfVegetation | GetVegType (void) |
| virtual double | GetWeedBiomass (void) |
| bool | HasTramlines (void) |
| virtual void | Insecticide (double) |
| virtual void | InsectMortality (double) |
| bool | IsRecentlyMown (void) |
| int | IsRecentlySprayed (void) |
|  | LE (void) |
| virtual void | RecalculateBugsNStuff (void) |
| virtual void | ReduceVeg (double) |
| virtual void | ReduceVeg\_Extended (double) |
| virtual void | ReduceWeedBiomass (double) |
| void | ResetTrace (void) |
| void | SetArea (double a\_area) |
| void | SetBorder (LE \*a\_border) |
| virtual void | SetCentroid (int x, int y) |
| void | SetCopyTreatment (int a\_treatment) |
| void | SetCountryDesignation (int a\_designation) |
| virtual void | SetCropData (double, double, double, TTypesOfVegetation, double, bool) |
| virtual void | SetCropDataAll (double, double, double, double, TTypesOfVegetation, double, double, bool, double, bool, double) |
| void | SetElementType (TTypesOfLandscapeElement a\_type) |
| void | SetFileType (int a\_file\_type) |
| virtual void | SetGrowthPhase (int) |
| void | SetHerbicideDelay (int a\_decaytime\_days) |
| void | SetHigh (bool a\_high) |
| virtual void | SetInsectPop (double) |
| void | SetLastTreatment (int a\_treatment) |
| void | SetMapIndex (int a\_map\_index) |
| void | SetMapValid (bool a\_valid) |
| void | SetMaxX (int x) |
| void | SetMaxY (int y) |
| void | SetMConstants (int a, int c) |
| void | SetMDates (int a, int b, int c) |
| void | SetMgtLoopDetectCount (long a\_num) |
| void | SetMgtLoopDetectDate (long a\_num) |
| void | SetMinX (int x) |
| void | SetMinY (int y) |
| void | SetMownDecay (int a\_decaytime\_days) |
| void | SetOldDays (long a\_days) |
| void | SetOwner (Farm \*a\_owner, int a\_owner\_num, int a\_owner\_index) |
| void | SetPesticideCell (int a\_cell) |
| void | SetPoison (bool a\_poison) |
| void | SetPoly (int a\_poly) |
| void | SetRotIndex (int a\_index) |
| void | SetSignal (LE\_Signal a\_signal) |
| void | SetSoilType (int a\_st) |
| void | SetSubType (int a\_subtype) |
| void | SetTramlinesDecay (int a\_decaytime\_days) |
| void | SetUnsprayedMarginPolyRef (int a\_unsprayedmargin) |
| void | SetValidXY (int a\_valid\_x, int a\_valid\_y) |
| virtual void | SetVegBiomass (int) |
| virtual void | SetVegHeight (double, double, double, double) |
| virtual void | SetVegPatchy (bool) |
| void | SetVegStore (int a\_veg) |
| virtual void | SetVegType (TTypesOfVegetation, TTypesOfVegetation) |
| virtual void | StoreLAItotal () |
| void | Tick (void) |
| virtual void | ToggleCattleGrazing (void) |
| virtual void | TogglePigGrazing (void) |
| void | Trace (int a\_value) |
| virtual void | ZeroVeg (void) |
| void | ZeroVegAge () |
| virtual | ~LE (void) |

|  |  |
| --- | --- |
| Public Attributes | |
| bool | m\_repeat\_start |
| bool | m\_skylarkscrapes |
|  | For management testing of skylark scrapes. |
| int | m\_squares\_in\_map |
| int | m\_tried\_to\_do |
| long | m\_user [EL\_MAX\_USERSPACE] |
|  | Variable used to record the current grazing pressure by e.g. voles. |

|  |  |
| --- | --- |
| Protected Attributes | |
| double | m\_area |
| LE \* | m\_border |
| bool | m\_cattle\_grazing |
| int | m\_centroidx |
| int | m\_centroidy |
| int | m\_countrydesignation |
| int | m\_days\_since\_insecticide\_spray |
| double | m\_ddegs |
| int | m\_farmfunc\_tried\_to\_do |
| int | m\_file\_type |
| int | m\_herbicidedelay |
| bool | m\_high |
| bool | m\_is\_in\_map |
| unsigned int | m\_lastindex |
| vector< int > | m\_lasttreat |
| long | m\_management\_loop\_detect\_count |
| long | m\_management\_loop\_detect\_date |
| int | m\_map\_index |
| int | m\_maxx |
| int | m\_maxy |
| int | m\_minx |
| int | m\_miny |
| int | m\_mowndecay |
| long | m\_olddays |
| Farm \* | m\_owner |
| int | m\_owner\_file |
| int | m\_owner\_index |
| int | m\_PesticideGridCell |
| bool | m\_pig\_grazing |
| bool | m\_poison |
| int | m\_poly |
| int | m\_rot\_index |
| long | m\_running |
| LE\_Signal | m\_signal\_mask |
| int | m\_soiltype |
| int | m\_subtype |
| int | m\_tramlinesdecay |
| TTypesOfLandscapeElement | m\_type |
| int | m\_unsprayedmarginpolyref |
| int | m\_valid\_x |
| int | m\_valid\_y |
| int | m\_vegage |
| int | m\_vege\_danger\_store |
| double | m\_yddegs |
| int | MConsts [10] |
| int | MDates [2][25] |

|  |  |
| --- | --- |
| Static Protected Attributes | |
| static double | m\_largeroad\_load [] |
| static double | m\_monthly\_traffic [] |
| static double | m\_smallroad\_load [] |

---

## Constructor & Destructor Documentation

|  |  |  |  |  |  |
| --- | --- | --- | --- | --- | --- |
| LE::LE | ( | void |  | ) |  |

References sleep\_all\_day, and tole\_Foobar.

{

m\_signal\_mask = 0;

m\_lasttreat.resize( 1 );

m\_lasttreat[ 0 ] = sleep\_all\_day;

m\_lastindex = 0;

m\_running = 0;

m\_poison = false;

m\_owner\_file = -1;

m\_owner\_index = -1;

m\_high = false;

m\_cattle\_grazing = false;

m\_pig\_grazing = false;

m\_yddegs = 0.0;

m\_olddays = 0;

m\_days\_since\_insecticide\_spray = 0;

m\_tramlinesdecay = 0;

m\_mowndecay = 0;

m\_herbicidedelay = 0;

m\_border = NULL;

// m\_unsprayedmargin= NULL;

m\_unsprayedmarginpolyref = -1;

m\_valid\_x = -1;

m\_valid\_y = -1;

m\_is\_in\_map = false;

m\_squares\_in\_map = 0;

m\_management\_loop\_detect\_date = 0;

m\_management\_loop\_detect\_count = 0;

m\_repeat\_start = false;

m\_skylarkscrapes = false;

m\_type = tole\_Foobar;

/\*/ The following lines are copyright to EcoSol all rights reserved /\*/

m\_maxx=-1; // a very small number

m\_maxy=-1;

m\_minx=9999999; // a ver big number

m\_miny=9999999;

/\*/ END EcoSol section /\*/

m\_countrydesignation = -1; // default not set

#ifdef FMDEBUG

m\_pindex = 0;

for ( int i = 0; i < 256; i++ ) {

m\_pdates[ i ] = 0;

m\_ptrace[ i ] = 0;

}

#endif

}

|  |  |  |  |  |  |  |  |
| --- | --- | --- | --- | --- | --- | --- | --- |
| |  |  |  |  |  |  | | --- | --- | --- | --- | --- | --- | | LE::~LE | ( | void |  | ) |  | | virtual |

{

}

---

## Member Function Documentation

|  |  |  |  |  |  |  |  |
| --- | --- | --- | --- | --- | --- | --- | --- |
| |  |  |  |  |  |  | | --- | --- | --- | --- | --- | --- | | void LE::AddArea | ( | double | *a\_area\_diff* | ) |  | | inline |

References m\_area.

Referenced by Landscape::BeetleBankAdd(), Landscape::BorderScan(), Landscape::BorderScan2(), and Landscape::UnsprayedMarginScan().

{ m\_area += a\_area\_diff; }

|  |  |  |  |  |  |  |  |
| --- | --- | --- | --- | --- | --- | --- | --- |
| |  |  |  |  |  |  | | --- | --- | --- | --- | --- | --- | | void LE::BumpRunNum | ( | void |  | ) |  | | inline |

References m\_running.

Referenced by Farm::HandleEvents().

{ m\_running++; }

|  |  |  |  |  |  |  |  |
| --- | --- | --- | --- | --- | --- | --- | --- |
| |  |  |  |  |  |  | | --- | --- | --- | --- | --- | --- | | void LE::DoDevelopment | ( | void |  | ) |  | | virtual |

Reimplemented in OrchardGrass, OrchardBand, Orchard, ForestElement, UnsprayedFieldMargin, NaturalGrass, Scrub, GreenElement, RoadsideVerge, BeetleBank, HedgeBank, Hedges, Field, and VegElement.

{

}

|  |  |  |  |  |  |  |  |
| --- | --- | --- | --- | --- | --- | --- | --- |
| |  |  |  |  |  |  | | --- | --- | --- | --- | --- | --- | | virtual void LE::ForceGrowthDevelopment | ( | void |  | ) |  | | inlinevirtual |

Reimplemented in VegElement.

{;}

|  |  |  |  |  |  |  |  |
| --- | --- | --- | --- | --- | --- | --- | --- |
| |  |  |  |  |  |  | | --- | --- | --- | --- | --- | --- | | virtual void LE::ForceGrowthInitialize | ( | void |  | ) |  | | inlinevirtual |

Reimplemented in VegElement.

{;}

|  |  |  |  |  |  |  |  |
| --- | --- | --- | --- | --- | --- | --- | --- |
| |  |  |  |  |  |  | | --- | --- | --- | --- | --- | --- | | virtual void LE::ForceGrowthTest | ( | void |  | ) |  | | inlinevirtual |

Reimplemented in VegElement.

Referenced by Farm::HandleEvents().

{;}

|  |  |  |  |  |  |  |  |
| --- | --- | --- | --- | --- | --- | --- | --- |
| |  |  |  |  |  |  | | --- | --- | --- | --- | --- | --- | | double LE::GetArea | ( | void |  | ) |  | | inline |

References m\_area.

Referenced by Landscape::BeetleBankPossible().

{ return m\_area; }

|  |  |  |  |  |  |  |  |
| --- | --- | --- | --- | --- | --- | --- | --- |
| |  |  |  |  |  |  | | --- | --- | --- | --- | --- | --- | | LE\* LE::GetBorder | ( | void |  | ) |  | | inline |

References m\_border.

Referenced by Landscape::BorderScan(), and Landscape::BorderScan2().

{ return m\_border; }

|  |  |  |  |  |  |  |  |
| --- | --- | --- | --- | --- | --- | --- | --- |
| |  |  |  |  |  |  | | --- | --- | --- | --- | --- | --- | | bool LE::GetCattleGrazing | ( | void |  | ) |  | | inline |

References m\_cattle\_grazing.

{ return m\_cattle\_grazing; }

|  |  |  |  |  |  |  |
| --- | --- | --- | --- | --- | --- | --- |
| |  |  |  |  |  | | --- | --- | --- | --- | --- | | virtual int LE::GetCentroidX | ( |  | ) |  | | inlinevirtual |

References m\_centroidx.

{ return m\_centroidx; }

|  |  |  |  |  |  |  |
| --- | --- | --- | --- | --- | --- | --- |
| |  |  |  |  |  | | --- | --- | --- | --- | --- | | virtual int LE::GetCentroidY | ( |  | ) |  | | inlinevirtual |

References m\_centroidy.

{ return m\_centroidy; }

|  |  |  |  |  |  |  |  |
| --- | --- | --- | --- | --- | --- | --- | --- |
| |  |  |  |  |  |  | | --- | --- | --- | --- | --- | --- | | int LE::GetCountryDesignation | ( | void |  | ) |  | | inline |

References m\_countrydesignation.

{ return m\_countrydesignation; }

|  |  |  |  |  |  |  |  |
| --- | --- | --- | --- | --- | --- | --- | --- |
| |  |  |  |  |  |  | | --- | --- | --- | --- | --- | --- | | virtual double LE::GetDayDegrees | ( | void |  | ) |  | | inlinevirtual |

References m\_ddegs.

{ return m\_ddegs; }

|  |  |  |  |  |  |  |  |
| --- | --- | --- | --- | --- | --- | --- | --- |
| |  |  |  |  |  |  | | --- | --- | --- | --- | --- | --- | | virtual double LE::GetDeadBiomass | ( | void |  | ) |  | | inlinevirtual |

Reimplemented in VegElement.

{ return 0.0; }

|  |  |  |  |  |  |  |  |
| --- | --- | --- | --- | --- | --- | --- | --- |
| |  |  |  |  |  |  | | --- | --- | --- | --- | --- | --- | | virtual double LE::GetDigestability | ( | void |  | ) |  | | inlinevirtual |

Reimplemented in VegElement.

{ return 0.0; }

|  |  |  |  |  |  |  |  |
| --- | --- | --- | --- | --- | --- | --- | --- |
| |  |  |  |  |  |  | | --- | --- | --- | --- | --- | --- | | virtual TTypesOfLandscapeElement LE::GetElementType | ( | void |  | ) |  | | inlinevirtual |

References m\_type.

Referenced by Farm::AddField(), and Landscape::ReadPolys().

{ return m\_type; }

|  |  |  |  |  |  |  |  |
| --- | --- | --- | --- | --- | --- | --- | --- |
| |  |  |  |  |  |  | | --- | --- | --- | --- | --- | --- | | int LE::GetFileType | ( | void |  | ) |  | | inline |

References m\_file\_type.

{ return m\_file\_type; }

|  |  |  |  |  |  |  |  |
| --- | --- | --- | --- | --- | --- | --- | --- |
| |  |  |  |  |  |  | | --- | --- | --- | --- | --- | --- | | virtual double LE::GetGreenBiomass | ( | void |  | ) |  | | inlinevirtual |

Reimplemented in VegElement.

{ return 0.0; }

|  |  |  |  |  |  |  |  |
| --- | --- | --- | --- | --- | --- | --- | --- |
| |  |  |  |  |  |  | | --- | --- | --- | --- | --- | --- | | bool LE::GetHigh | ( | void |  | ) |  | | inline |

References m\_high.

{ return m\_high; }

|  |  |  |  |  |  |  |  |
| --- | --- | --- | --- | --- | --- | --- | --- |
| |  |  |  |  |  |  | | --- | --- | --- | --- | --- | --- | | virtual double LE::GetInsectPop | ( | void |  | ) |  | | inlinevirtual |

Reimplemented in VegElement.

Referenced by Farm::Harvest().

{ return 0.0; }

|  |  |  |  |  |  |  |  |
| --- | --- | --- | --- | --- | --- | --- | --- |
| |  |  |  |  |  |  | | --- | --- | --- | --- | --- | --- | | virtual double LE::GetLAGreen | ( | void |  | ) |  | | inlinevirtual |

Reimplemented in VegElement.

{ return 0.0; }

|  |  |  |  |  |  |
| --- | --- | --- | --- | --- | --- |
| int LE::GetLastTreatment | ( | int \* | *a\_index* | ) |  |

References sleep\_all\_day.

{

if ( \* a\_index == ( int )m\_lastindex )

return sleep\_all\_day;

int i = ( \* a\_index ) ++;

int treat = m\_lasttreat[ i ];

return treat;

}

|  |  |  |  |  |  |  |  |
| --- | --- | --- | --- | --- | --- | --- | --- |
| |  |  |  |  |  |  | | --- | --- | --- | --- | --- | --- | | virtual double LE::GetLATotal | ( | void |  | ) |  | | inlinevirtual |

Reimplemented in VegElement.

{ return 0.0; }

|  |  |  |  |  |  |  |  |
| --- | --- | --- | --- | --- | --- | --- | --- |
| |  |  |  |  |  |  | | --- | --- | --- | --- | --- | --- | | int LE::GetMapIndex | ( | void |  | ) |  | | inline |

References m\_map\_index.

Referenced by Pesticide::TwinMapSpray().

{ return m\_map\_index; }

|  |  |  |  |  |  |  |  |
| --- | --- | --- | --- | --- | --- | --- | --- |
| |  |  |  |  |  |  | | --- | --- | --- | --- | --- | --- | | bool LE::GetMapValid | ( | void |  | ) |  | | inline |

References m\_is\_in\_map.

{ return m\_is\_in\_map; }

|  |  |  |  |  |  |  |  |
| --- | --- | --- | --- | --- | --- | --- | --- |
| |  |  |  |  |  |  | | --- | --- | --- | --- | --- | --- | | int LE::GetMaxX | ( | void |  | ) |  | | inline |

References m\_maxx.

Referenced by Pesticide::TwinMapSpray().

{ return m\_maxx; }

|  |  |  |  |  |  |  |  |
| --- | --- | --- | --- | --- | --- | --- | --- |
| |  |  |  |  |  |  | | --- | --- | --- | --- | --- | --- | | int LE::GetMaxY | ( | void |  | ) |  | | inline |

References m\_maxy.

Referenced by Pesticide::TwinMapSpray().

{ return m\_maxy; }

|  |  |  |  |  |  |  |  |
| --- | --- | --- | --- | --- | --- | --- | --- |
| |  |  |  |  |  |  | | --- | --- | --- | --- | --- | --- | | int LE::GetMConstants | ( | int | *a* | ) |  | | inline |

References MConsts.

{return MConsts[a];}

|  |  |  |  |  |  |  |  |  |  |  |  |  |  |
| --- | --- | --- | --- | --- | --- | --- | --- | --- | --- | --- | --- | --- | --- |
| |  |  |  |  | | --- | --- | --- | --- | | int LE::GetMDates | ( | int | *a*, | |  |  | int | *b* | |  | ) |  |  | | inline |

References MDates.

{return MDates[a][b];}

|  |  |  |  |  |  |  |  |
| --- | --- | --- | --- | --- | --- | --- | --- |
| |  |  |  |  |  |  | | --- | --- | --- | --- | --- | --- | | long LE::GetMgtLoopDetectCount | ( | void |  | ) |  | | inline |

References m\_management\_loop\_detect\_count.

Referenced by Farm::CheckRotationManagementLoop().

{ return m\_management\_loop\_detect\_count; }

|  |  |  |  |  |  |  |  |
| --- | --- | --- | --- | --- | --- | --- | --- |
| |  |  |  |  |  |  | | --- | --- | --- | --- | --- | --- | | long LE::GetMgtLoopDetectDate | ( | void |  | ) |  | | inline |

References m\_management\_loop\_detect\_date.

Referenced by Farm::CheckRotationManagementLoop().

{ return m\_management\_loop\_detect\_date; }

|  |  |  |  |  |  |  |  |
| --- | --- | --- | --- | --- | --- | --- | --- |
| |  |  |  |  |  |  | | --- | --- | --- | --- | --- | --- | | int LE::GetMinX | ( | void |  | ) |  | | inline |

References m\_minx.

Referenced by Pesticide::TwinMapSpray().

{ return m\_minx; }

|  |  |  |  |  |  |  |  |
| --- | --- | --- | --- | --- | --- | --- | --- |
| |  |  |  |  |  |  | | --- | --- | --- | --- | --- | --- | | int LE::GetMinY | ( | void |  | ) |  | | inline |

References m\_miny.

Referenced by Pesticide::TwinMapSpray().

{ return m\_miny; }

|  |  |  |  |  |  |  |  |
| --- | --- | --- | --- | --- | --- | --- | --- |
| |  |  |  |  |  |  | | --- | --- | --- | --- | --- | --- | | long LE::GetOldDays | ( | void |  | ) |  | | inline |

References m\_olddays.

{ return m\_olddays; }

|  |  |  |  |  |  |  |  |
| --- | --- | --- | --- | --- | --- | --- | --- |
| |  |  |  |  |  |  | | --- | --- | --- | --- | --- | --- | | Farm\* LE::GetOwner | ( | void |  | ) |  | | inline |

References m\_owner.

{ return m\_owner; }

|  |  |  |  |  |  |  |  |
| --- | --- | --- | --- | --- | --- | --- | --- |
| |  |  |  |  |  |  | | --- | --- | --- | --- | --- | --- | | int LE::GetOwnerFile | ( | void |  | ) |  | | inline |

References m\_owner\_file.

{ return m\_owner\_file; }

|  |  |  |  |  |  |  |  |
| --- | --- | --- | --- | --- | --- | --- | --- |
| |  |  |  |  |  |  | | --- | --- | --- | --- | --- | --- | | int LE::GetOwnerIndex | ( | void |  | ) |  | | inline |

References m\_owner\_index.

{ return m\_owner\_index; }

|  |  |  |  |  |  |  |
| --- | --- | --- | --- | --- | --- | --- |
| |  |  |  |  |  | | --- | --- | --- | --- | --- | | int LE::GetPesticideCell | ( |  | ) |  | | inline |

References m\_PesticideGridCell.

{ return m\_PesticideGridCell; }

|  |  |  |  |  |  |  |  |
| --- | --- | --- | --- | --- | --- | --- | --- |
| |  |  |  |  |  |  | | --- | --- | --- | --- | --- | --- | | bool LE::GetPigGrazing | ( | void |  | ) |  | | inline |

References m\_pig\_grazing.

{ return m\_pig\_grazing; }

|  |  |  |  |  |  |  |  |
| --- | --- | --- | --- | --- | --- | --- | --- |
| |  |  |  |  |  |  | | --- | --- | --- | --- | --- | --- | | bool LE::GetPoison | ( | void |  | ) |  | | inline |

References m\_poison.

{ return m\_poison; }

|  |  |  |  |  |  |  |  |
| --- | --- | --- | --- | --- | --- | --- | --- |
| |  |  |  |  |  |  | | --- | --- | --- | --- | --- | --- | | int LE::GetPoly | ( | void |  | ) |  | | inline |

References m\_poly.

Referenced by Landscape::BorderScan(), Landscape::BorderScan2(), Landscape::FindFieldCenter(), Landscape::hb\_StripingDist(), and Landscape::UnsprayedMarginScan().

{ return m\_poly; }

|  |  |  |  |  |  |  |  |
| --- | --- | --- | --- | --- | --- | --- | --- |
| |  |  |  |  |  |  | | --- | --- | --- | --- | --- | --- | | int LE::GetRotIndex | ( | void |  | ) |  | | inline |

References m\_rot\_index.

Referenced by Farm::GetNextCropStartDate(), and Farm::HandleEvents().

{ return m\_rot\_index; }

|  |  |  |  |  |  |  |  |
| --- | --- | --- | --- | --- | --- | --- | --- |
| |  |  |  |  |  |  | | --- | --- | --- | --- | --- | --- | | long LE::GetRunNum | ( | void |  | ) |  | | inline |

References m\_running.

Referenced by Farm::HandleEvents(), and Farm::LeSwitch().

{ return m\_running; }

|  |  |  |  |  |  |  |  |
| --- | --- | --- | --- | --- | --- | --- | --- |
| |  |  |  |  |  |  | | --- | --- | --- | --- | --- | --- | | LE\_Signal LE::GetSignal | ( | void |  | ) |  | | inline |

References m\_signal\_mask.

Referenced by Farm::FungicideTreat(), Farm::GrowthRegulator(), Farm::HerbicideTreat(), Farm::InsecticideTreat(), Farm::Molluscicide(), and Farm::SynInsecticideTreat().

{ return m\_signal\_mask; }

|  |  |  |  |  |  |  |  |
| --- | --- | --- | --- | --- | --- | --- | --- |
| |  |  |  |  |  |  | | --- | --- | --- | --- | --- | --- | | virtual bool LE::GetSkScrapes | ( | void |  | ) |  | | inlinevirtual |

Reimplemented in VegElement.

{ return false; }

|  |  |  |  |  |  |  |
| --- | --- | --- | --- | --- | --- | --- |
| |  |  |  |  |  | | --- | --- | --- | --- | --- | | int LE::GetSoilType | ( |  | ) |  | | inline |

/brief Gets the soil type ref number for the polygon

The soil type follows the Danish Soil Classification and is denoted by a JB number as follows:

References m\_soiltype.

{

return m\_soiltype;

}

|  |  |  |  |  |  |  |  |
| --- | --- | --- | --- | --- | --- | --- | --- |
| |  |  |  |  |  |  | | --- | --- | --- | --- | --- | --- | | int LE::GetSubType | ( | void |  | ) |  | | inline |

References m\_subtype.

{ return m\_subtype; }

|  |  |  |  |  |  |  |  |
| --- | --- | --- | --- | --- | --- | --- | --- |
| |  |  |  |  |  |  | | --- | --- | --- | --- | --- | --- | | virtual double LE::GetTrafficLoad | ( | void |  | ) |  | | inlinevirtual |

Reimplemented in SmallRoad, and LargeRoad.

{ return 0.0; }

|  |  |  |  |  |  |  |  |
| --- | --- | --- | --- | --- | --- | --- | --- |
| |  |  |  |  |  |  | | --- | --- | --- | --- | --- | --- | | int LE::GetUnsprayedMarginPolyRef | ( | void |  | ) |  | | inline |

References m\_unsprayedmarginpolyref.

Referenced by Farm::AutumnHarrow(), Farm::AutumnPlough(), Farm::AutumnRoll(), Farm::AutumnSow(), Farm::BurnStrawStubble(), Farm::CattleIsOut(), Farm::CattleIsOutLow(), Farm::CattleOut(), Farm::CattleOutLowGrazing(), Farm::CutToHay(), Farm::CutToSilage(), Farm::CutWeeds(), Farm::DeepPlough(), Field::DoDevelopment(), Farm::FA\_AmmoniumSulphate(), Farm::FA\_GreenManure(), Farm::FA\_Manure(), Farm::FA\_NPK(), Farm::FA\_PK(), Farm::FA\_Sludge(), Farm::FA\_Slurry(), Farm::FP\_GreenManure(), Farm::FP\_LiquidNH3(), Farm::FP\_ManganeseSulphate(), Farm::FP\_Manure(), Farm::FP\_NPK(), Farm::FP\_NPKS(), Farm::FP\_PK(), Farm::FP\_Sludge(), Farm::FP\_Slurry(), Farm::Harvest(), Farm::HayBailing(), Farm::HayTurning(), Farm::HillingUp(), Farm::PigsAreOut(), Farm::PigsAreOutForced(), Farm::PigsOut(), Farm::RowCultivation(), Farm::SleepAllDay(), Farm::SpringHarrow(), Farm::SpringPlough(), Farm::SpringRoll(), Farm::SpringSow(), Farm::StrawChopping(), Farm::Strigling(), Farm::StriglingSow(), Farm::StubbleHarrowing(), Farm::Swathing(), Landscape::UnsprayedMarginScan(), Farm::Water(), and Farm::WinterPlough().

{ return m\_unsprayedmarginpolyref; }

|  |  |  |  |  |  |  |  |
| --- | --- | --- | --- | --- | --- | --- | --- |
| |  |  |  |  |  |  | | --- | --- | --- | --- | --- | --- | | int LE::GetValidX | ( | void |  | ) |  | | inline |

References m\_valid\_x.

Referenced by Landscape::BeetleBankPossible(), Landscape::BorderAdd(), Landscape::BorderScan(), Landscape::BorderScan2(), Landscape::OrchardBorderAdd(), Landscape::UnsprayedMarginAdd(), and Landscape::UnsprayedMarginScan().

{ return m\_valid\_x; }

|  |  |  |  |  |  |  |  |
| --- | --- | --- | --- | --- | --- | --- | --- |
| |  |  |  |  |  |  | | --- | --- | --- | --- | --- | --- | | int LE::GetValidY | ( | void |  | ) |  | | inline |

References m\_valid\_y.

Referenced by Landscape::BeetleBankPossible(), Landscape::BorderAdd(), Landscape::BorderScan(), Landscape::BorderScan2(), Landscape::OrchardBorderAdd(), Landscape::UnsprayedMarginAdd(), and Landscape::UnsprayedMarginScan().

{ return m\_valid\_y; }

|  |  |  |  |  |  |  |
| --- | --- | --- | --- | --- | --- | --- |
| |  |  |  |  |  | | --- | --- | --- | --- | --- | | int LE::GetVegAge | ( |  | ) |  | | inline |

References m\_vegage.

{ return m\_vegage; }

|  |  |  |  |  |  |  |  |
| --- | --- | --- | --- | --- | --- | --- | --- |
| |  |  |  |  |  |  | | --- | --- | --- | --- | --- | --- | | virtual double LE::GetVegBiomass | ( | void |  | ) |  | | inlinevirtual |

Reimplemented in VegElement.

Referenced by Pesticide::TwinMapSpray().

{ return 0.0; }

|  |  |  |  |  |  |  |  |
| --- | --- | --- | --- | --- | --- | --- | --- |
| |  |  |  |  |  |  | | --- | --- | --- | --- | --- | --- | | virtual double LE::GetVegCover | ( | void |  | ) |  | | inlinevirtual |

Reimplemented in VegElement.

Referenced by Pesticide::TwinMapSpray().

{ return 0.0; }

|  |  |  |  |  |  |  |  |
| --- | --- | --- | --- | --- | --- | --- | --- |
| |  |  |  |  |  |  | | --- | --- | --- | --- | --- | --- | | virtual int LE::GetVegDensity | ( | void |  | ) |  | | inlinevirtual |

Reimplemented in VegElement.

{ return 0; }

|  |  |  |  |  |  |  |  |
| --- | --- | --- | --- | --- | --- | --- | --- |
| |  |  |  |  |  |  | | --- | --- | --- | --- | --- | --- | | virtual double LE::GetVegHeight | ( | void |  | ) |  | | inlinevirtual |

Reimplemented in VegElement.

Referenced by Farm::CattleIsOut(), Farm::CattleIsOutLow(), Farm::CattleOut(), Farm::CattleOutLowGrazing(), and Farm::Trial\_PesticideTreat\_GS().

{ return 0.0; }

|  |  |  |  |  |  |  |  |
| --- | --- | --- | --- | --- | --- | --- | --- |
| |  |  |  |  |  |  | | --- | --- | --- | --- | --- | --- | | virtual bool LE::GetVegPatchy | ( | void |  | ) |  | | inlinevirtual |

Reimplemented in VegElement.

{ return false; }

|  |  |  |  |  |  |  |  |
| --- | --- | --- | --- | --- | --- | --- | --- |
| |  |  |  |  |  |  | | --- | --- | --- | --- | --- | --- | | int LE::GetVegStore | ( | void |  | ) |  | | inline |

References m\_vege\_danger\_store.

{ return m\_vege\_danger\_store; }

|  |  |  |  |  |  |  |  |
| --- | --- | --- | --- | --- | --- | --- | --- |
| |  |  |  |  |  |  | | --- | --- | --- | --- | --- | --- | | virtual TTypesOfVegetation LE::GetVegType | ( | void |  | ) |  | | inlinevirtual |

Reimplemented in VegElement.

References tov\_None.

Referenced by Farm::HandleEvents().

{ return tov\_None; }

|  |  |  |  |  |  |  |  |
| --- | --- | --- | --- | --- | --- | --- | --- |
| |  |  |  |  |  |  | | --- | --- | --- | --- | --- | --- | | virtual double LE::GetWeedBiomass | ( | void |  | ) |  | | inlinevirtual |

Reimplemented in VegElement.

{ return 0.0; }

|  |  |  |  |  |  |  |  |
| --- | --- | --- | --- | --- | --- | --- | --- |
| |  |  |  |  |  |  | | --- | --- | --- | --- | --- | --- | | bool LE::HasTramlines | ( | void |  | ) |  | | inline |

References m\_tramlinesdecay.

{ return ( m\_tramlinesdecay>0 ); }

|  |  |  |  |  |  |  |  |
| --- | --- | --- | --- | --- | --- | --- | --- |
| |  |  |  |  |  |  | | --- | --- | --- | --- | --- | --- | | virtual void LE::Insecticide | ( | double |  | ) |  | | inlinevirtual |

Reimplemented in VegElement.

Referenced by Farm::InsecticideTreat(), Farm::ProductApplication0(), Farm::ProductApplication1(), and Farm::SynInsecticideTreat().

{ ; }

|  |  |  |  |  |  |  |  |
| --- | --- | --- | --- | --- | --- | --- | --- |
| |  |  |  |  |  |  | | --- | --- | --- | --- | --- | --- | | virtual void LE::InsectMortality | ( | double |  | ) |  | | inlinevirtual |

Reimplemented in VegElement.

Referenced by Farm::AutumnHarrow(), Farm::AutumnPlough(), Farm::BurnStrawStubble(), Farm::CutToHay(), Farm::CutToSilage(), Farm::DeepPlough(), Farm::FP\_NPKS(), Farm::Glyphosate(), Farm::Harvest(), Farm::HillingUp(), Farm::RowCultivation(), Farm::SpringHarrow(), Farm::SpringPlough(), Farm::StrawChopping(), Farm::Strigling(), Farm::StriglingSow(), Farm::StubbleHarrowing(), Farm::Swathing(), Farm::Trial\_ToxicControl(), and Farm::WinterPlough().

{ ; }

|  |  |  |  |  |  |  |  |
| --- | --- | --- | --- | --- | --- | --- | --- |
| |  |  |  |  |  |  | | --- | --- | --- | --- | --- | --- | | bool LE::IsRecentlyMown | ( | void |  | ) |  | | inline |

References m\_mowndecay.

{ return ( m\_mowndecay>0 ); }

|  |  |  |  |  |  |  |  |
| --- | --- | --- | --- | --- | --- | --- | --- |
| |  |  |  |  |  |  | | --- | --- | --- | --- | --- | --- | | int LE::IsRecentlySprayed | ( | void |  | ) |  | | inline |

References m\_herbicidedelay.

{ return ( m\_herbicidedelay ); }

|  |  |  |  |  |  |  |  |
| --- | --- | --- | --- | --- | --- | --- | --- |
| |  |  |  |  |  |  | | --- | --- | --- | --- | --- | --- | | virtual void LE::RecalculateBugsNStuff | ( | void |  | ) |  | | inlinevirtual |

Reimplemented in VegElement.

Referenced by Farm::Harvest().

{ }

|  |  |  |  |  |  |  |  |
| --- | --- | --- | --- | --- | --- | --- | --- |
| |  |  |  |  |  |  | | --- | --- | --- | --- | --- | --- | | virtual void LE::ReduceVeg | ( | double |  | ) |  | | inlinevirtual |

Reimplemented in VegElement.

Referenced by Farm::BurnStrawStubble(), and Farm::CutWeeds().

{;}

|  |  |  |  |  |  |  |  |
| --- | --- | --- | --- | --- | --- | --- | --- |
| |  |  |  |  |  |  | | --- | --- | --- | --- | --- | --- | | virtual void LE::ReduceVeg\_Extended | ( | double |  | ) |  | | inlinevirtual |

Reimplemented in VegElement.

Referenced by Farm::CattleIsOut(), Farm::CattleIsOutLow(), Farm::CattleOut(), Farm::CattleOutLowGrazing(), Farm::CutToHay(), Farm::CutToSilage(), Farm::Glyphosate(), Farm::PigsAreOutForced(), and Farm::PigsOut().

{;}

|  |  |  |  |  |  |  |  |
| --- | --- | --- | --- | --- | --- | --- | --- |
| |  |  |  |  |  |  | | --- | --- | --- | --- | --- | --- | | virtual void LE::ReduceWeedBiomass | ( | double |  | ) |  | | inlinevirtual |

Reimplemented in VegElement.

Referenced by Farm::HerbicideTreat(), Farm::HillingUp(), Farm::RowCultivation(), Farm::Strigling(), Farm::StriglingSow(), and Farm::Trial\_PesticideTreat\_GS().

{ ; }

|  |  |  |  |  |  |
| --- | --- | --- | --- | --- | --- |
| void LE::ResetTrace | ( | void |  | ) |  |

Referenced by Farm::HandleEvents().

{

}

|  |  |  |  |  |  |  |  |
| --- | --- | --- | --- | --- | --- | --- | --- |
| |  |  |  |  |  |  | | --- | --- | --- | --- | --- | --- | | void LE::SetArea | ( | double | *a\_area* | ) |  | | inline |

References m\_area.

Referenced by Landscape::BeetleBankAdd(), Landscape::BorderAdd(), Landscape::OrchardBorderAdd(), Landscape::ReadPolys(), and Landscape::UnsprayedMarginAdd().

{ m\_area = a\_area; }

|  |  |  |  |  |  |  |  |
| --- | --- | --- | --- | --- | --- | --- | --- |
| |  |  |  |  |  |  | | --- | --- | --- | --- | --- | --- | | void LE::SetBorder | ( | LE \* | *a\_border* | ) |  | | inline |

References m\_border.

Referenced by Landscape::BorderAdd(), Landscape::OrchardBorderAdd(), and Landscape::ReadPolys().

{ m\_border = a\_border; }

|  |  |  |  |  |  |  |  |  |  |  |  |  |  |
| --- | --- | --- | --- | --- | --- | --- | --- | --- | --- | --- | --- | --- | --- |
| |  |  |  |  | | --- | --- | --- | --- | | virtual void LE::SetCentroid | ( | int | *x*, | |  |  | int | *y* | |  | ) |  |  | | inlinevirtual |

References m\_centroidx, and m\_centroidy.

Referenced by Landscape::ReadPolys().

{ m\_centroidx=x; m\_centroidy=y; }

|  |  |  |  |  |  |
| --- | --- | --- | --- | --- | --- |
| void LE::SetCopyTreatment | ( | int | *a\_treatment* | ) |  |

{

SetLastTreatment( a\_treatment );

}

|  |  |  |  |  |  |  |  |
| --- | --- | --- | --- | --- | --- | --- | --- |
| |  |  |  |  |  |  | | --- | --- | --- | --- | --- | --- | | void LE::SetCountryDesignation | ( | int | *a\_designation* | ) |  | | inline |

References m\_countrydesignation.

{ m\_countrydesignation = a\_designation; }

|  |  |  |  |  |  |  |  |  |  |  |  |  |  |  |  |  |  |  |  |  |  |  |  |  |  |  |  |  |  |
| --- | --- | --- | --- | --- | --- | --- | --- | --- | --- | --- | --- | --- | --- | --- | --- | --- | --- | --- | --- | --- | --- | --- | --- | --- | --- | --- | --- | --- | --- |
| |  |  |  |  | | --- | --- | --- | --- | | virtual void LE::SetCropData | ( | double | , | |  |  | double | , | |  |  | double | , | |  |  | TTypesOfVegetation | , | |  |  | double | , | |  |  | bool |  | |  | ) |  |  | | inlinevirtual |

Reimplemented in VegElement.

Referenced by Field::DoDevelopment().

{;}

|  |  |  |  |  |  |  |  |  |  |  |  |  |  |  |  |  |  |  |  |  |  |  |  |  |  |  |  |  |  |  |  |  |  |  |  |  |  |  |  |  |  |  |  |  |  |  |  |  |  |
| --- | --- | --- | --- | --- | --- | --- | --- | --- | --- | --- | --- | --- | --- | --- | --- | --- | --- | --- | --- | --- | --- | --- | --- | --- | --- | --- | --- | --- | --- | --- | --- | --- | --- | --- | --- | --- | --- | --- | --- | --- | --- | --- | --- | --- | --- | --- | --- | --- | --- |
| |  |  |  |  | | --- | --- | --- | --- | | virtual void LE::SetCropDataAll | ( | double | , | |  |  | double | , | |  |  | double | , | |  |  | double | , | |  |  | TTypesOfVegetation | , | |  |  | double | , | |  |  | double | , | |  |  | bool | , | |  |  | double | , | |  |  | bool | , | |  |  | double |  | |  | ) |  |  | | inlinevirtual |

Reimplemented in VegElement.

Referenced by Field::DoDevelopment().

{;}

|  |  |  |  |  |  |  |  |
| --- | --- | --- | --- | --- | --- | --- | --- |
| |  |  |  |  |  |  | | --- | --- | --- | --- | --- | --- | | void LE::SetElementType | ( | TTypesOfLandscapeElement | *a\_type* | ) |  | | inline |

References m\_type.

Referenced by Landscape::NewElement().

{ m\_type = a\_type; }

|  |  |  |  |  |  |  |  |
| --- | --- | --- | --- | --- | --- | --- | --- |
| |  |  |  |  |  |  | | --- | --- | --- | --- | --- | --- | | void LE::SetFileType | ( | int | *a\_file\_type* | ) |  | | inline |

References m\_file\_type.

Referenced by NaturalGrass::NaturalGrass(), Landscape::NewElement(), and Landscape::ReadPolys().

{ m\_file\_type = a\_file\_type; }

|  |  |  |  |  |  |  |  |
| --- | --- | --- | --- | --- | --- | --- | --- |
| |  |  |  |  |  |  | | --- | --- | --- | --- | --- | --- | | virtual void LE::SetGrowthPhase | ( | int |  | ) |  | | inlinevirtual |

Reimplemented in VegElement.

Referenced by Farm::AutumnSow(), Farm::FP\_NPKS(), Farm::Harvest(), and Farm::SpringSow().

{;}

|  |  |  |  |  |  |  |  |
| --- | --- | --- | --- | --- | --- | --- | --- |
| |  |  |  |  |  |  | | --- | --- | --- | --- | --- | --- | | void LE::SetHerbicideDelay | ( | int | *a\_decaytime\_days* | ) |  | | inline |

References m\_herbicidedelay.

Referenced by Farm::HerbicideTreat(), Farm::Strigling(), Farm::StriglingSow(), and Farm::Trial\_PesticideTreat\_GS().

{ m\_herbicidedelay = a\_decaytime\_days; }

|  |  |  |  |  |  |  |  |
| --- | --- | --- | --- | --- | --- | --- | --- |
| |  |  |  |  |  |  | | --- | --- | --- | --- | --- | --- | | void LE::SetHigh | ( | bool | *a\_high* | ) |  | | inline |

References m\_high.

Referenced by ActivePit::ActivePit(), Building::Building(), ForestElement::ForestElement(), Garden::Garden(), Hedges::Hedges(), PitDisused::PitDisused(), RiversideTrees::RiversideTrees(), StoneWall::StoneWall(), Urban::Urban(), and YoungForest::YoungForest().

{ m\_high = a\_high; }

|  |  |  |  |  |  |  |  |
| --- | --- | --- | --- | --- | --- | --- | --- |
| |  |  |  |  |  |  | | --- | --- | --- | --- | --- | --- | | virtual void LE::SetInsectPop | ( | double |  | ) |  | | inlinevirtual |

Reimplemented in VegElement.

Referenced by Farm::Harvest().

{ ; }

|  |  |  |  |  |  |
| --- | --- | --- | --- | --- | --- |
| void LE::SetLastTreatment | ( | int | *a\_treatment* | ) |  |

Referenced by Farm::AutumnHarrow(), Farm::AutumnPlough(), Farm::AutumnRoll(), Farm::AutumnSow(), Farm::BurnStrawStubble(), Farm::CattleIsOut(), Farm::CattleIsOutLow(), Farm::CattleOut(), Farm::CattleOutLowGrazing(), RoadsideVerge::Cutting(), Orchard::Cutting(), OrchardGrass::Cutting(), Farm::CutToHay(), Farm::CutToSilage(), Farm::CutWeeds(), Farm::DeepPlough(), Farm::FA\_AmmoniumSulphate(), Farm::FA\_GreenManure(), Farm::FA\_Manure(), Farm::FA\_NPK(), Farm::FA\_PK(), Farm::FA\_Sludge(), Farm::FA\_Slurry(), Farm::FP\_GreenManure(), Farm::FP\_LiquidNH3(), Farm::FP\_ManganeseSulphate(), Farm::FP\_Manure(), Farm::FP\_NPK(), Farm::FP\_NPKS(), Farm::FP\_PK(), Farm::FP\_Sludge(), Farm::FP\_Slurry(), Farm::FungicideTreat(), Farm::Glyphosate(), Farm::GrowthRegulator(), Farm::Harvest(), Farm::HayBailing(), Farm::HayTurning(), Farm::HerbicideTreat(), Farm::HillingUp(), Farm::InsecticideTreat(), Farm::Molluscicide(), Farm::PigsAreOutForced(), Farm::PigsOut(), Farm::ProductApplication0(), Farm::ProductApplication1(), Farm::RowCultivation(), Farm::SleepAllDay(), Farm::SpringHarrow(), Farm::SpringPlough(), Farm::SpringRoll(), Farm::SpringSow(), Farm::StrawChopping(), Farm::Strigling(), Farm::StriglingSow(), Farm::StubbleHarrowing(), Farm::Swathing(), Farm::SynInsecticideTreat(), Farm::Trial\_Control(), Farm::Trial\_PesticideTreat(), Farm::Trial\_PesticideTreat\_GS(), Farm::Trial\_ToxicControl(), Farm::Water(), and Farm::WinterPlough().

{

unsigned sz = (int) m\_lasttreat.size();

if ( m\_lastindex == sz )

m\_lasttreat.resize( m\_lastindex + 1 );

m\_lasttreat[ m\_lastindex++ ] = a\_treatment;

// Count this treatment in the grand scope of things.

g\_landscape\_p->IncTreatCounter( a\_treatment );

// If we have a field margin then we need to tell it about this

// but not if it is an insecticide spray etc..

/\* if (m\_unsprayedmarginpolyref!=-1) { switch (a\_treatment) { case herbicide\_treat: case growth\_regulator:

case fungicide\_treat: case insecticide\_treat: case trial\_insecticidetreat: case syninsecticide\_treat: case molluscicide:

break; // Do not add sprayings default: LE\* le=g\_landscape\_p->SupplyLEPointer(m\_unsprayedmarginpolyref);

le->SetCopyTreatment(a\_treatment); // Now we also need to do something with the treatment

break; }

} \*/

}

|  |  |  |  |  |  |  |  |
| --- | --- | --- | --- | --- | --- | --- | --- |
| |  |  |  |  |  |  | | --- | --- | --- | --- | --- | --- | | void LE::SetMapIndex | ( | int | *a\_map\_index* | ) |  | | inline |

References m\_map\_index.

{ m\_map\_index = a\_map\_index; }

|  |  |  |  |  |  |  |  |
| --- | --- | --- | --- | --- | --- | --- | --- |
| |  |  |  |  |  |  | | --- | --- | --- | --- | --- | --- | | void LE::SetMapValid | ( | bool | *a\_valid* | ) |  | | inline |

References m\_is\_in\_map.

Referenced by Landscape::BeetleBankAdd().

{ m\_is\_in\_map = a\_valid; }

|  |  |  |  |  |  |  |  |
| --- | --- | --- | --- | --- | --- | --- | --- |
| |  |  |  |  |  |  | | --- | --- | --- | --- | --- | --- | | void LE::SetMaxX | ( | int | *x* | ) |  | | inline |

References m\_maxx.

{ m\_maxx=x; }

|  |  |  |  |  |  |  |  |
| --- | --- | --- | --- | --- | --- | --- | --- |
| |  |  |  |  |  |  | | --- | --- | --- | --- | --- | --- | | void LE::SetMaxY | ( | int | *y* | ) |  | | inline |

References m\_maxy.

{ m\_maxy=y; }

|  |  |  |  |  |  |  |  |  |  |  |  |  |  |
| --- | --- | --- | --- | --- | --- | --- | --- | --- | --- | --- | --- | --- | --- |
| |  |  |  |  | | --- | --- | --- | --- | | void LE::SetMConstants | ( | int | *a*, | |  |  | int | *c* | |  | ) |  |  | | inline |

References MConsts.

{MConsts[a]=c;}

|  |  |  |  |  |  |  |  |  |  |  |  |  |  |  |  |  |  |
| --- | --- | --- | --- | --- | --- | --- | --- | --- | --- | --- | --- | --- | --- | --- | --- | --- | --- |
| |  |  |  |  | | --- | --- | --- | --- | | void LE::SetMDates | ( | int | *a*, | |  |  | int | *b*, | |  |  | int | *c* | |  | ) |  |  | | inline |

References MDates.

{MDates[a][b]=c;}

|  |  |  |  |  |  |  |  |
| --- | --- | --- | --- | --- | --- | --- | --- |
| |  |  |  |  |  |  | | --- | --- | --- | --- | --- | --- | | void LE::SetMgtLoopDetectCount | ( | long | *a\_num* | ) |  | | inline |

References m\_management\_loop\_detect\_count.

Referenced by Farm::CheckRotationManagementLoop().

{ m\_management\_loop\_detect\_count = a\_num; }

|  |  |  |  |  |  |  |  |
| --- | --- | --- | --- | --- | --- | --- | --- |
| |  |  |  |  |  |  | | --- | --- | --- | --- | --- | --- | | void LE::SetMgtLoopDetectDate | ( | long | *a\_num* | ) |  | | inline |

References m\_management\_loop\_detect\_date.

Referenced by Farm::HandleEvents().

{ m\_management\_loop\_detect\_date = a\_num; }

|  |  |  |  |  |  |  |  |
| --- | --- | --- | --- | --- | --- | --- | --- |
| |  |  |  |  |  |  | | --- | --- | --- | --- | --- | --- | | void LE::SetMinX | ( | int | *x* | ) |  | | inline |

References m\_minx.

{ m\_minx=x; }

|  |  |  |  |  |  |  |  |
| --- | --- | --- | --- | --- | --- | --- | --- |
| |  |  |  |  |  |  | | --- | --- | --- | --- | --- | --- | | void LE::SetMinY | ( | int | *y* | ) |  | | inline |

References m\_miny.

{ m\_miny=y; }

|  |  |  |  |  |  |  |  |
| --- | --- | --- | --- | --- | --- | --- | --- |
| |  |  |  |  |  |  | | --- | --- | --- | --- | --- | --- | | void LE::SetMownDecay | ( | int | *a\_decaytime\_days* | ) |  | | inline |

References m\_mowndecay.

Referenced by Orchard::Cutting(), and OrchardGrass::Cutting().

{ m\_mowndecay = a\_decaytime\_days; }

|  |  |  |  |  |  |  |  |
| --- | --- | --- | --- | --- | --- | --- | --- |
| |  |  |  |  |  |  | | --- | --- | --- | --- | --- | --- | | void LE::SetOldDays | ( | long | *a\_days* | ) |  | | inline |

References m\_olddays.

{ m\_olddays = a\_days; }

|  |  |  |  |  |  |  |  |  |  |  |  |  |  |  |  |  |  |
| --- | --- | --- | --- | --- | --- | --- | --- | --- | --- | --- | --- | --- | --- | --- | --- | --- | --- |
| |  |  |  |  | | --- | --- | --- | --- | | void LE::SetOwner | ( | Farm \* | *a\_owner*, | |  |  | int | *a\_owner\_num*, | |  |  | int | *a\_owner\_index* | |  | ) |  |  | | inline |

References m\_owner, m\_owner\_file, and m\_owner\_index.

Referenced by FarmManager::AddField().

{

m\_owner = a\_owner;

m\_owner\_file = a\_owner\_num;

m\_owner\_index = a\_owner\_index;

}

|  |  |  |  |  |  |  |  |
| --- | --- | --- | --- | --- | --- | --- | --- |
| |  |  |  |  |  |  | | --- | --- | --- | --- | --- | --- | | void LE::SetPesticideCell | ( | int | *a\_cell* | ) |  | | inline |

References m\_PesticideGridCell.

{ m\_PesticideGridCell = a\_cell; }

|  |  |  |  |  |  |  |  |
| --- | --- | --- | --- | --- | --- | --- | --- |
| |  |  |  |  |  |  | | --- | --- | --- | --- | --- | --- | | void LE::SetPoison | ( | bool | *a\_poison* | ) |  | | inline |

References m\_poison.

{ m\_poison = a\_poison; }

|  |  |  |  |  |  |  |  |
| --- | --- | --- | --- | --- | --- | --- | --- |
| |  |  |  |  |  |  | | --- | --- | --- | --- | --- | --- | | void LE::SetPoly | ( | int | *a\_poly* | ) |  | | inline |

References m\_poly.

Referenced by Landscape::BeetleBankAdd(), Landscape::BorderAdd(), Landscape::OrchardBorderAdd(), Landscape::ReadPolys(), and Landscape::UnsprayedMarginAdd().

{ m\_poly = a\_poly; }

|  |  |  |  |  |  |  |  |
| --- | --- | --- | --- | --- | --- | --- | --- |
| |  |  |  |  |  |  | | --- | --- | --- | --- | --- | --- | | void LE::SetRotIndex | ( | int | *a\_index* | ) |  | | inline |

References m\_rot\_index.

Referenced by Farm::HandleEvents().

{ m\_rot\_index = a\_index; }

|  |  |  |  |  |  |  |  |
| --- | --- | --- | --- | --- | --- | --- | --- |
| |  |  |  |  |  |  | | --- | --- | --- | --- | --- | --- | | void LE::SetSignal | ( | LE\_Signal | *a\_signal* | ) |  | | inline |

References m\_signal\_mask.

{ m\_signal\_mask = a\_signal; }

|  |  |  |  |  |  |  |  |
| --- | --- | --- | --- | --- | --- | --- | --- |
| |  |  |  |  |  |  | | --- | --- | --- | --- | --- | --- | | void LE::SetSoilType | ( | int | *a\_st* | ) |  | | inline |

References m\_soiltype.

Referenced by Landscape::ReadPolys().

{ m\_soiltype = a\_st; }

|  |  |  |  |  |  |  |  |
| --- | --- | --- | --- | --- | --- | --- | --- |
| |  |  |  |  |  |  | | --- | --- | --- | --- | --- | --- | | void LE::SetSubType | ( | int | *a\_subtype* | ) |  | | inline |

References m\_subtype.

Referenced by HedgeBank::HedgeBank(), and Hedges::Hedges().

{ m\_subtype = a\_subtype; }

|  |  |  |  |  |  |  |  |
| --- | --- | --- | --- | --- | --- | --- | --- |
| |  |  |  |  |  |  | | --- | --- | --- | --- | --- | --- | | void LE::SetTramlinesDecay | ( | int | *a\_decaytime\_days* | ) |  | | inline |

References m\_tramlinesdecay.

Referenced by Farm::BurnStrawStubble(), Farm::CutToHay(), Farm::CutToSilage(), Farm::CutWeeds(), Farm::FA\_AmmoniumSulphate(), Farm::FA\_GreenManure(), Farm::FA\_Manure(), Farm::FA\_NPK(), Farm::FA\_PK(), Farm::FA\_Sludge(), Farm::FA\_Slurry(), Farm::FP\_GreenManure(), Farm::FP\_LiquidNH3(), Farm::FP\_ManganeseSulphate(), Farm::FP\_Manure(), Farm::FP\_NPK(), Farm::FP\_NPKS(), Farm::FP\_PK(), Farm::FP\_Sludge(), Farm::FP\_Slurry(), Farm::FungicideTreat(), Farm::GrowthRegulator(), Farm::Harvest(), Farm::HayBailing(), Farm::HayTurning(), Farm::HerbicideTreat(), Farm::HillingUp(), Farm::InsecticideTreat(), Farm::Molluscicide(), Farm::ProductApplication0(), Farm::ProductApplication1(), Farm::RowCultivation(), Farm::StrawChopping(), Farm::Strigling(), Farm::StriglingSow(), Farm::Swathing(), Farm::SynInsecticideTreat(), Farm::Trial\_Control(), Farm::Trial\_PesticideTreat(), Farm::Trial\_PesticideTreat\_GS(), and Farm::Trial\_ToxicControl().

{ m\_tramlinesdecay = a\_decaytime\_days; }

|  |  |  |  |  |  |  |  |
| --- | --- | --- | --- | --- | --- | --- | --- |
| |  |  |  |  |  |  | | --- | --- | --- | --- | --- | --- | | void LE::SetUnsprayedMarginPolyRef | ( | int | *a\_unsprayedmargin* | ) |  | | inline |

References m\_unsprayedmarginpolyref.

Referenced by Landscape::ReadPolys(), and Landscape::UnsprayedMarginAdd().

{ m\_unsprayedmarginpolyref = a\_unsprayedmargin; }

|  |  |  |  |  |  |  |  |  |  |  |  |  |  |
| --- | --- | --- | --- | --- | --- | --- | --- | --- | --- | --- | --- | --- | --- |
| |  |  |  |  | | --- | --- | --- | --- | | void LE::SetValidXY | ( | int | *a\_valid\_x*, | |  |  | int | *a\_valid\_y* | |  | ) |  |  | | inline |

References m\_valid\_x, and m\_valid\_y.

Referenced by Landscape::BeetleBankAdd().

{

m\_valid\_x = a\_valid\_x;

m\_valid\_y = a\_valid\_y;

}

|  |  |  |  |  |  |  |  |
| --- | --- | --- | --- | --- | --- | --- | --- |
| |  |  |  |  |  |  | | --- | --- | --- | --- | --- | --- | | virtual void LE::SetVegBiomass | ( | int |  | ) |  | | inlinevirtual |

{;}

|  |  |  |  |  |  |  |  |  |  |  |  |  |  |  |  |  |  |  |  |  |  |
| --- | --- | --- | --- | --- | --- | --- | --- | --- | --- | --- | --- | --- | --- | --- | --- | --- | --- | --- | --- | --- | --- |
| |  |  |  |  | | --- | --- | --- | --- | | virtual void LE::SetVegHeight | ( | double | , | |  |  | double | , | |  |  | double | , | |  |  | double |  | |  | ) |  |  | | inlinevirtual |

Reimplemented in VegElement.

Referenced by Farm::CutToHay(), and Farm::CutToSilage().

{;}

|  |  |  |  |  |  |  |  |
| --- | --- | --- | --- | --- | --- | --- | --- |
| |  |  |  |  |  |  | | --- | --- | --- | --- | --- | --- | | virtual void LE::SetVegPatchy | ( | bool |  | ) |  | | inlinevirtual |

Reimplemented in VegElement.

{ ;}

|  |  |  |  |  |  |  |  |
| --- | --- | --- | --- | --- | --- | --- | --- |
| |  |  |  |  |  |  | | --- | --- | --- | --- | --- | --- | | void LE::SetVegStore | ( | int | *a\_veg* | ) |  | | inline |

References m\_vege\_danger\_store.

Referenced by Farm::HandleEvents().

{ m\_vege\_danger\_store = a\_veg; }

|  |  |  |  |  |  |  |  |  |  |  |  |  |  |
| --- | --- | --- | --- | --- | --- | --- | --- | --- | --- | --- | --- | --- | --- |
| |  |  |  |  | | --- | --- | --- | --- | | virtual void LE::SetVegType | ( | TTypesOfVegetation | , | |  |  | TTypesOfVegetation |  | |  | ) |  |  | | inlinevirtual |

Reimplemented in VegElement.

Referenced by Farm::HandleEvents().

{;}

|  |  |  |  |  |  |  |
| --- | --- | --- | --- | --- | --- | --- |
| |  |  |  |  |  | | --- | --- | --- | --- | --- | | virtual void LE::StoreLAItotal | ( |  | ) |  | | inlinevirtual |

Reimplemented in VegElement.

{;}

|  |  |  |  |  |  |
| --- | --- | --- | --- | --- | --- |
| void LE::Tick | ( | void |  | ) |  |

References sleep\_all\_day.

{

m\_lastindex = 0;

m\_lasttreat[ 0 ] = sleep\_all\_day;

if ( m\_tramlinesdecay > 0 )

m\_tramlinesdecay--;

if ( m\_mowndecay > 0 )

m\_mowndecay--;

if ( m\_herbicidedelay > 0 ) m\_herbicidedelay--;

}

|  |  |  |  |  |  |  |  |
| --- | --- | --- | --- | --- | --- | --- | --- |
| |  |  |  |  |  |  | | --- | --- | --- | --- | --- | --- | | virtual void LE::ToggleCattleGrazing | ( | void |  | ) |  | | inlinevirtual |

Reimplemented in VegElement.

Referenced by Farm::CattleIsOut(), Farm::CattleIsOutLow(), Farm::CattleOut(), and Farm::CattleOutLowGrazing().

{;}

|  |  |  |  |  |  |  |  |
| --- | --- | --- | --- | --- | --- | --- | --- |
| |  |  |  |  |  |  | | --- | --- | --- | --- | --- | --- | | virtual void LE::TogglePigGrazing | ( | void |  | ) |  | | inlinevirtual |

Reimplemented in VegElement.

Referenced by Farm::PigsAreOut(), and Farm::PigsOut().

{;}

|  |  |  |  |  |  |
| --- | --- | --- | --- | --- | --- |
| void LE::Trace | ( | int | *a\_value* | ) |  |

Referenced by Farm::AutumnHarrow(), Farm::AutumnPlough(), Farm::AutumnRoll(), Farm::AutumnSow(), Farm::BurnStrawStubble(), Farm::CattleIsOut(), Farm::CattleIsOutLow(), Farm::CattleOut(), Farm::CattleOutLowGrazing(), Farm::CutToHay(), Farm::CutToSilage(), Farm::CutWeeds(), Farm::DeepPlough(), Farm::FA\_AmmoniumSulphate(), Farm::FA\_GreenManure(), Farm::FA\_Manure(), Farm::FA\_NPK(), Farm::FA\_PK(), Farm::FA\_Sludge(), Farm::FA\_Slurry(), Farm::FP\_GreenManure(), Farm::FP\_LiquidNH3(), Farm::FP\_ManganeseSulphate(), Farm::FP\_Manure(), Farm::FP\_NPK(), Farm::FP\_NPKS(), Farm::FP\_PK(), Farm::FP\_Sludge(), Farm::FP\_Slurry(), Farm::FungicideTreat(), Farm::Glyphosate(), Farm::GrowthRegulator(), Farm::Harvest(), Farm::HayBailing(), Farm::HayTurning(), Farm::HerbicideTreat(), Farm::HillingUp(), Farm::InsecticideTreat(), Farm::Molluscicide(), Farm::PigsAreOutForced(), Farm::PigsOut(), Farm::ProductApplication0(), Farm::ProductApplication1(), Farm::RowCultivation(), Farm::SleepAllDay(), Farm::SpringHarrow(), Farm::SpringPlough(), Farm::SpringRoll(), Farm::SpringSow(), Farm::StrawChopping(), Farm::Strigling(), Farm::StriglingSow(), Farm::StubbleHarrowing(), Farm::Swathing(), Farm::SynInsecticideTreat(), Farm::Trial\_Control(), Farm::Trial\_PesticideTreat(), Farm::Trial\_PesticideTreat\_GS(), Farm::Trial\_ToxicControl(), Farm::Water(), and Farm::WinterPlough().

{

m\_farmfunc\_tried\_to\_do = a\_value;

}

|  |  |  |  |  |  |  |  |
| --- | --- | --- | --- | --- | --- | --- | --- |
| |  |  |  |  |  |  | | --- | --- | --- | --- | --- | --- | | virtual void LE::ZeroVeg | ( | void |  | ) |  | | inlinevirtual |

Reimplemented in VegElement.

Referenced by Farm::AutumnHarrow(), Farm::AutumnPlough(), Farm::AutumnRoll(), Farm::AutumnSow(), Farm::DeepPlough(), Farm::SpringHarrow(), Farm::SpringPlough(), Farm::SpringSow(), Farm::StubbleHarrowing(), and Farm::WinterPlough().

{;}

|  |  |  |  |  |  |  |
| --- | --- | --- | --- | --- | --- | --- |
| |  |  |  |  |  | | --- | --- | --- | --- | --- | | void LE::ZeroVegAge | ( |  | ) |  | | inline |

References m\_vegage.

{ m\_vegage=0; }

---

## Member Data Documentation

|  |  |  |
| --- | --- | --- |
| |  | | --- | | double LE::m\_area | | protected |

Referenced by AddArea(), GetArea(), and SetArea().

|  |  |  |
| --- | --- | --- |
| |  | | --- | | LE\* LE::m\_border | | protected |

Referenced by GetBorder(), and SetBorder().

|  |  |  |
| --- | --- | --- |
| |  | | --- | | bool LE::m\_cattle\_grazing | | protected |

Referenced by Field::DoDevelopment(), GetCattleGrazing(), VegElement::SetCropData(), VegElement::SetCropDataAll(), and VegElement::ToggleCattleGrazing().

|  |  |  |
| --- | --- | --- |
| |  | | --- | | int LE::m\_centroidx | | protected |

Referenced by GetCentroidX(), and SetCentroid().

|  |  |  |
| --- | --- | --- |
| |  | | --- | | int LE::m\_centroidy | | protected |

Referenced by GetCentroidY(), and SetCentroid().

|  |  |  |
| --- | --- | --- |
| |  | | --- | | int LE::m\_countrydesignation | | protected |

Referenced by Building::Building(), GetCountryDesignation(), and SetCountryDesignation().

|  |  |  |
| --- | --- | --- |
| |  | | --- | | int LE::m\_days\_since\_insecticide\_spray | | protected |

Referenced by VegElement::Insecticide(), and VegElement::RecalculateBugsNStuff().

|  |  |  |
| --- | --- | --- |
| |  | | --- | | double LE::m\_ddegs | | protected |

Referenced by VegElement::DoDevelopment(), UnsprayedFieldMargin::DoDevelopment(), GetDayDegrees(), VegElement::ReduceVeg\_Extended(), VegElement::SetGrowthPhase(), and VegElement::VegElement().

|  |  |  |
| --- | --- | --- |
| |  | | --- | | int LE::m\_farmfunc\_tried\_to\_do | | protected |

|  |  |  |
| --- | --- | --- |
| |  | | --- | | int LE::m\_file\_type | | protected |

Referenced by GetFileType(), and SetFileType().

|  |  |  |
| --- | --- | --- |
| |  | | --- | | int LE::m\_herbicidedelay | | protected |

Referenced by VegElement::DoDevelopment(), OrchardBand::DoDevelopment(), IsRecentlySprayed(), and SetHerbicideDelay().

|  |  |  |
| --- | --- | --- |
| |  | | --- | | bool LE::m\_high | | protected |

Referenced by GetHigh(), and SetHigh().

|  |  |  |
| --- | --- | --- |
| |  | | --- | | bool LE::m\_is\_in\_map | | protected |

Referenced by GetMapValid(), and SetMapValid().

|  |  |  |
| --- | --- | --- |
| |  | | --- | | double LE::m\_largeroad\_load | | staticprotected |

**Initial value:**

{15,9,4,5,14,54,332,381,252,206,204,215,

231,256,335,470,384,270,191,130,91,100,99,60}

Referenced by LargeRoad::GetTrafficLoad().

|  |  |  |
| --- | --- | --- |
| |  | | --- | | unsigned int LE::m\_lastindex | | protected |

|  |  |  |
| --- | --- | --- |
| |  | | --- | | vector<int> LE::m\_lasttreat | | protected |

|  |  |  |
| --- | --- | --- |
| |  | | --- | | long LE::m\_management\_loop\_detect\_count | | protected |

Referenced by GetMgtLoopDetectCount(), and SetMgtLoopDetectCount().

|  |  |  |
| --- | --- | --- |
| |  | | --- | | long LE::m\_management\_loop\_detect\_date | | protected |

Referenced by GetMgtLoopDetectDate(), and SetMgtLoopDetectDate().

|  |  |  |
| --- | --- | --- |
| |  | | --- | | int LE::m\_map\_index | | protected |

Referenced by GetMapIndex(), and SetMapIndex().

|  |  |  |
| --- | --- | --- |
| |  | | --- | | int LE::m\_maxx | | protected |

Referenced by GetMaxX(), and SetMaxX().

|  |  |  |
| --- | --- | --- |
| |  | | --- | | int LE::m\_maxy | | protected |

Referenced by GetMaxY(), and SetMaxY().

|  |  |  |
| --- | --- | --- |
| |  | | --- | | int LE::m\_minx | | protected |

Referenced by GetMinX(), and SetMinX().

|  |  |  |
| --- | --- | --- |
| |  | | --- | | int LE::m\_miny | | protected |

Referenced by GetMinY(), and SetMinY().

|  |  |  |
| --- | --- | --- |
| |  | | --- | | double LE::m\_monthly\_traffic | | staticprotected |

**Initial value:**

{0.9009, 0.9434, 0.9901, 1.0101, 1.0417, 1.0870,

1.0870, 1.0753, 1.0753, 1.0101, 0.9804, 0.9434}

Referenced by LargeRoad::GetTrafficLoad(), and SmallRoad::GetTrafficLoad().

|  |  |  |
| --- | --- | --- |
| |  | | --- | | int LE::m\_mowndecay | | protected |

Referenced by IsRecentlyMown(), and SetMownDecay().

|  |  |  |
| --- | --- | --- |
| |  | | --- | | long LE::m\_olddays | | protected |

Referenced by GetOldDays(), and SetOldDays().

|  |  |  |
| --- | --- | --- |
| |  | | --- | | Farm\* LE::m\_owner | | protected |

Referenced by GetOwner(), and SetOwner().

|  |  |  |
| --- | --- | --- |
| |  | | --- | | int LE::m\_owner\_file | | protected |

Referenced by GetOwnerFile(), and SetOwner().

|  |  |  |
| --- | --- | --- |
| |  | | --- | | int LE::m\_owner\_index | | protected |

Referenced by VegElement::DoDevelopment(), GetOwnerIndex(), VegElement::RecalculateBugsNStuff(), and SetOwner().

|  |  |  |
| --- | --- | --- |
| |  | | --- | | int LE::m\_PesticideGridCell | | protected |

Referenced by GetPesticideCell(), and SetPesticideCell().

|  |  |  |
| --- | --- | --- |
| |  | | --- | | bool LE::m\_pig\_grazing | | protected |

Referenced by GetPigGrazing(), and VegElement::TogglePigGrazing().

|  |  |  |
| --- | --- | --- |
| |  | | --- | | bool LE::m\_poison | | protected |

Referenced by GetPoison(), and SetPoison().

|  |  |  |
| --- | --- | --- |
| |  | | --- | | int LE::m\_poly | | protected |

Referenced by VegElement::DoDevelopment(), GetPoly(), VegElement::RecalculateBugsNStuff(), and SetPoly().

|  |
| --- |
| bool LE::m\_repeat\_start |

|  |  |  |
| --- | --- | --- |
| |  | | --- | | int LE::m\_rot\_index | | protected |

Referenced by GetRotIndex(), and SetRotIndex().

|  |  |  |
| --- | --- | --- |
| |  | | --- | | long LE::m\_running | | protected |

Referenced by BumpRunNum(), and GetRunNum().

|  |  |  |
| --- | --- | --- |
| |  | | --- | | LE\_Signal LE::m\_signal\_mask | | protected |

Referenced by GetSignal(), and SetSignal().

|  |
| --- |
| bool LE::m\_skylarkscrapes |

For management testing of skylark scrapes.

Referenced by VegElement::GetSkScrapes().

|  |  |  |
| --- | --- | --- |
| |  | | --- | | double LE::m\_smallroad\_load | | staticprotected |

**Initial value:**

{4,3,1,1,4,15,94,108,71,58,58,61,

65,73,95,133,109,76,54,37,26,28,28,17}

Referenced by SmallRoad::GetTrafficLoad().

|  |  |  |
| --- | --- | --- |
| |  | | --- | | int LE::m\_soiltype | | protected |

Referenced by GetSoilType(), and SetSoilType().

|  |
| --- |
| int LE::m\_squares\_in\_map |

|  |  |  |
| --- | --- | --- |
| |  | | --- | | int LE::m\_subtype | | protected |

Referenced by GetSubType(), and SetSubType().

|  |  |  |
| --- | --- | --- |
| |  | | --- | | int LE::m\_tramlinesdecay | | protected |

Referenced by HasTramlines(), and SetTramlinesDecay().

|  |
| --- |
| int LE::m\_tried\_to\_do |

Referenced by Farm::LeSwitch().

|  |  |  |
| --- | --- | --- |
| |  | | --- | | TTypesOfLandscapeElement LE::m\_type | | protected |

Referenced by ActivePit::ActivePit(), AmenityGrass::AmenityGrass(), BareRock::BareRock(), BeetleBank::BeetleBank(), Building::Building(), BuiltUpWithParkland::BuiltUpWithParkland(), Coast::Coast(), ConiferousForest::ConiferousForest(), Copse::Copse(), DeciduousForest::DeciduousForest(), Field::DoDevelopment(), Field::Field(), FieldBoundary::FieldBoundary(), Freshwater::Freshwater(), Garden::Garden(), GetElementType(), Heath::Heath(), HedgeBank::HedgeBank(), Hedges::Hedges(), LargeRoad::LargeRoad(), Marsh::Marsh(), MixedForest::MixedForest(), NaturalGrass::NaturalGrass(), Orchard::Orchard(), OrchardBand::OrchardBand(), OrchardGrass::OrchardGrass(), Parkland::Parkland(), PermanentSetaside::PermanentSetaside(), PermPasture::PermPasture(), PermPastureLowYield::PermPastureLowYield(), PermPastureTussocky::PermPastureTussocky(), PitDisused::PitDisused(), Railway::Railway(), River::River(), RiversidePlants::RiversidePlants(), RiversideTrees::RiversideTrees(), RoadsideVerge::RoadsideVerge(), RuralResidential::RuralResidential(), Saltwater::Saltwater(), SandDune::SandDune(), Scrub::Scrub(), SetElementType(), SmallRoad::SmallRoad(), StoneWall::StoneWall(), Suburban::Suburban(), Track::Track(), UnsprayedFieldMargin::UnsprayedFieldMargin(), Urban::Urban(), UrbanNoVeg::UrbanNoVeg(), UrbanPark::UrbanPark(), and YoungForest::YoungForest().

|  |  |  |
| --- | --- | --- |
| |  | | --- | | int LE::m\_unsprayedmarginpolyref | | protected |

Referenced by GetUnsprayedMarginPolyRef(), and SetUnsprayedMarginPolyRef().

|  |
| --- |
| long LE::m\_user[EL\_MAX\_USERSPACE] |

Variable used to record the current grazing pressure by e.g. voles.

Variable used to reduce the grazing pressure Variable used to record the current grazing pressure by e.g. voles Change the current grazing pressure by e.g. voles Get the current total grazing pressure by e.g. voles Calculate the current grazing pressure by e.g. voles Get the current grazing pressure by e.g. voles Reset the current grazing pressure by e.g. voles

|  |  |  |
| --- | --- | --- |
| |  | | --- | | int LE::m\_valid\_x | | protected |

Referenced by GetValidX(), and SetValidXY().

|  |  |  |
| --- | --- | --- |
| |  | | --- | | int LE::m\_valid\_y | | protected |

Referenced by GetValidY(), and SetValidXY().

|  |  |  |
| --- | --- | --- |
| |  | | --- | | int LE::m\_vegage | | protected |

Referenced by GetVegAge(), and ZeroVegAge().

|  |  |  |
| --- | --- | --- |
| |  | | --- | | int LE::m\_vege\_danger\_store | | protected |

Referenced by GetVegStore(), and SetVegStore().

|  |  |  |
| --- | --- | --- |
| |  | | --- | | double LE::m\_yddegs | | protected |

Referenced by VegElement::DoDevelopment(), UnsprayedFieldMargin::DoDevelopment(), VegElement::ReduceVeg\_Extended(), VegElement::SetGrowthPhase(), and VegElement::VegElement().

|  |  |  |
| --- | --- | --- |
| |  | | --- | | int LE::MConsts[10] | | protected |

Referenced by GetMConstants(), and SetMConstants().

|  |  |  |
| --- | --- | --- |
| |  | | --- | | int LE::MDates[2][25] | | protected |

Referenced by GetMDates(), and SetMDates().

---

The documentation for this class was generated from the following files:

- elements.h
- elements.cpp


- LE
- Generated on Thu Jan 10 2013 13:15:36 for ALMaSS Skylark ODdox by
   1.8.1.1
