# Supplementary material for: Modelling Skylarks (Alauda arvensis) to Predict Impacts of Changes in Land Management and Policy: Development and Testing of an Agent-Based Model
Source: PLoS One. 2013 Jun 6;8(6):e65803. doi: 10.1371/journal.pone.0065803 (PMC3675089; doi:10.1371/journal.pone.0065803)
Supplement: Supporting Information S4 — The skylark ODdox as a zipped archive. (ZIP) [file pone.0065803.s004.zip › Skylark_ODdox/class_l_e___type_class.html]

ALMaSS Skylark ODdox: LE\_TypeClass Class Reference


|  |
| --- |
| ALMaSS Skylark ODdox  2.0 |


- Main Page
- Related Pages
- Classes
- Files

- Class List
- Class Index
- Class Hierarchy
- Class Members

Public Member Functions

LE\_TypeClass Class Reference

`#include <elements.h>`

List of all members.

|  |  |
| --- | --- |
| Public Member Functions | |
| int | BackTranslateEleTypes (TTypesOfLandscapeElement EleReference) |
| int | BackTranslateVegTypes (TTypesOfVegetation VegReference) |
| TTypesOfLandscapeElement | TranslateEleTypes (int EleReference) |
| TTypesOfVegetation | TranslateVegTypes (int VegReference) |
| int | VegTypeToCurveNum (TTypesOfVegetation VegReference) |

---

## Member Function Documentation

|  |  |  |  |  |  |
| --- | --- | --- | --- | --- | --- |
| int LE\_TypeClass::BackTranslateEleTypes | ( | TTypesOfLandscapeElement | *EleReference* | ) |  |

References g\_msg, tole\_ActivePit, tole\_AmenityGrass, tole\_BareRock, tole\_BeetleBank, tole\_Building, tole\_BuiltUpWithParkland, tole\_Coast, tole\_ConiferousForest, tole\_Copse, tole\_DeciduousForest, tole\_Field, tole\_FieldBoundary, tole\_Freshwater, tole\_Garden, tole\_Heath, tole\_HedgeBank, tole\_Hedges, tole\_LargeRoad, tole\_Marsh, tole\_MixedForest, tole\_NaturalGrass, tole\_Orchard, tole\_OrchardBand, tole\_OrchardGrass, tole\_Parkland, tole\_PermanentSetaside, tole\_PermPasture, tole\_PermPastureLowYield, tole\_PermPastureTussocky, tole\_PitDisused, tole\_Railway, tole\_River, tole\_RiversidePlants, tole\_RiversideTrees, tole\_RoadsideVerge, tole\_RuralResidential, tole\_Saltwater, tole\_SandDune, tole\_Scrub, tole\_SmallRoad, tole\_StoneWall, tole\_Suburban, tole\_Track, tole\_UnsprayedFieldMargin, tole\_Urban, tole\_UrbanNoVeg, tole\_UrbanPark, tole\_YoungForest, MapErrorMsg::Warn(), and WARN\_FILE.

Referenced by Landscape::BackTranslateEleTypes().

{

static char error\_num[ 20 ];

// This returns the vegetation type (or crop type) as applicable

switch ( EleReference ) {

case tole\_Building:

return 5;

case tole\_Copse:

return 41;

case tole\_UrbanNoVeg:

return 6;

case tole\_Suburban:

return 7;

case tole\_RuralResidential:

return 8;

case tole\_SandDune:

return 101;

case tole\_Urban:

return 10;

case tole\_Garden:

return 11;

case tole\_AmenityGrass:

return 12;

case tole\_RoadsideVerge:

return 13;

case tole\_Parkland:

return 14;

case tole\_StoneWall:

return 15;

case tole\_BuiltUpWithParkland:

return 16;

case tole\_UrbanPark:

return 17;

// \*FN\* case tole\_Field: return 20, 30;

case tole\_Field:

return 20;

case tole\_PermPastureTussocky:

return 27;

case tole\_PermPastureLowYield:

return 26;

case tole\_UnsprayedFieldMargin:

return 31;

case tole\_PermanentSetaside:

return 35;

case tole\_PermPasture:

return 35;

case tole\_DeciduousForest:

return 40;

case tole\_ConiferousForest:

return 50;

case tole\_YoungForest:

return 55;

case tole\_Orchard:

return 56;

case tole\_BareRock:

return 59;

case tole\_OrchardBand:

return 57;

case tole\_OrchardGrass:

return 58;

case tole\_MixedForest:

return 60;

case tole\_Scrub:

return 70;

case tole\_PitDisused:

return 75;

case tole\_Saltwater:

return 80;

case tole\_Freshwater:

return 90;

case tole\_Heath:

return 94;

case tole\_Marsh:

return 95;

case tole\_River:

return 96;

case tole\_RiversideTrees:

return 97;

case tole\_RiversidePlants:

return 98;

case tole\_Coast:

return 100;

case tole\_NaturalGrass:

return 110;

case tole\_ActivePit:

return 115;

case tole\_Railway:

return 118;

case tole\_LargeRoad:

return 121;

case tole\_SmallRoad:

return 122;

case tole\_Track:

return 123;

case tole\_Hedges:

return 130;

case tole\_HedgeBank:

return 140;

case tole\_BeetleBank:

return 141;

case tole\_FieldBoundary:

return 160;

//case tole\_Foobar: return 999;

// !! type unknown - should not happen

default:

sprintf( error\_num, "%d", EleReference );

g\_msg->Warn( WARN\_FILE, "LE\_TypeClass::BackTranslateEleTypes(): ""Unknown vegetation type:", error\_num );

exit( 1 );

}

}

|  |  |  |  |  |  |
| --- | --- | --- | --- | --- | --- |
| int LE\_TypeClass::BackTranslateVegTypes | ( | TTypesOfVegetation | *VegReference* | ) |  |

References g\_msg, tov\_AgroChemIndustryCereal, tov\_Carrots, tov\_CloverGrassGrazed1, tov\_CloverGrassGrazed2, tov\_FieldPeas, tov\_FodderBeet, tov\_FodderGrass, tov\_Maize, tov\_MaizeSilage, tov\_NaturalGrass, tov\_NoGrowth, tov\_None, tov\_Oats, tov\_OBarleyPeaCloverGrass, tov\_OCarrots, tov\_OCloverGrassGrazed1, tov\_OCloverGrassGrazed2, tov\_OCloverGrassSilage1, tov\_OFieldPeas, tov\_OFieldPeasSilage, tov\_OMaizeSilage, tov\_OOats, tov\_OPermanentGrassGrazed, tov\_OPotatoes, tov\_OSBarleySilage, tov\_OSeedGrass1, tov\_OSeedGrass2, tov\_OSpringBarley, tov\_OSpringBarleyClover, tov\_OSpringBarleyExt, tov\_OSpringBarleyGrass, tov\_OTriticale, tov\_OWinterBarley, tov\_OWinterBarleyExt, tov\_OWinterRape, tov\_OWinterRye, tov\_OWinterWheatUndersown, tov\_PermanentGrassGrazed, tov\_PermanentGrassLowYield, tov\_PermanentGrassTussocky, tov\_PermanentSetaside, tov\_Potatoes, tov\_PotatoesIndustry, tov\_SeedGrass1, tov\_SeedGrass2, tov\_Setaside, tov\_SpringBarley, tov\_SpringBarleyCloverGrass, tov\_SpringBarleyGrass, tov\_SpringBarleyPeaCloverGrassStrigling, tov\_SpringBarleyPTreatment, tov\_SpringBarleySeed, tov\_SpringBarleySKManagement, tov\_SpringBarleyStrigling, tov\_SpringBarleyStriglingCulm, tov\_SpringBarleyStriglingSingle, tov\_SpringRape, tov\_SpringWheat, tov\_Triticale, tov\_Undefined, tov\_WinterBarley, tov\_WinterRape, tov\_WinterRye, tov\_WinterWheat, tov\_WinterWheatShort, tov\_WinterWheatStrigling, tov\_WinterWheatStriglingCulm, tov\_WinterWheatStriglingSingle, tov\_WWheatPControl, tov\_WWheatPToxicControl, tov\_WWheatPTreatment, tov\_YoungForest, MapErrorMsg::Warn(), and WARN\_FILE.

Referenced by Landscape::BackTranslateVegTypes().

{

char error\_num[ 20 ];

// This returns the vegetation type (or crop type) as applicable

switch ( VegReference ) {

case tov\_SpringBarley:

return 1;

case tov\_WinterBarley:

return 2;

case tov\_SpringWheat:

return 3;

case tov\_WinterWheat:

return 4;

case tov\_WinterRye:

return 5;

case tov\_Oats:

return 6;

case tov\_Triticale:

return 7;

case tov\_Maize:

return 8;

case tov\_SpringBarleySeed:

return 13;

case tov\_SpringBarleyStrigling:

return 14;

case tov\_SpringBarleyStriglingSingle:

return 15;

case tov\_SpringBarleyStriglingCulm:

return 16;

case tov\_WinterWheatStrigling: return 17;

case tov\_WinterWheatStriglingSingle: return 18;

case tov\_WinterWheatStriglingCulm: return 19;

case tov\_SpringRape:

return 21;

case tov\_WinterRape:

return 22;

case tov\_FieldPeas:

return 30;

case tov\_Setaside:

return 50;

case tov\_PermanentSetaside:

return 54;

case tov\_YoungForest:

return 55;

case tov\_FodderBeet:

return 60;

case tov\_CloverGrassGrazed1:

return 65;

case tov\_PotatoesIndustry:

return 92;

case tov\_Potatoes:

return 93;

case tov\_SeedGrass1:

return 94;

case tov\_OWinterBarley:

return 102;

case tov\_OWinterBarleyExt:

return 611;

case tov\_OWinterRye:

return 105;

case tov\_SpringBarleyGrass:

return 107;

case tov\_SpringBarleyCloverGrass:

return 108;

case tov\_OBarleyPeaCloverGrass:

return 113;

case tov\_SpringBarleyPeaCloverGrassStrigling:

return 114;

case tov\_OWinterRape:

return 122;

case tov\_PermanentGrassGrazed:

return 140;

case tov\_PermanentGrassLowYield:

return 141;

case tov\_PermanentGrassTussocky:

return 142;

case tov\_CloverGrassGrazed2:

return 165;

case tov\_SeedGrass2:

return 194;

case tov\_OSpringBarley:

return 201;

case tov\_OWinterWheatUndersown:

return 204;

case tov\_OOats:

return 206;

case tov\_OTriticale:

return 207;

case tov\_OFieldPeas:

return 230;

case tov\_OFieldPeasSilage:

return 106;

case tov\_OCloverGrassGrazed1:

return 265;

case tov\_OCarrots:

return 270;

case tov\_Carrots:

return 271;

case tov\_OPotatoes:

return 293;

case tov\_OSeedGrass1:

return 294;

case tov\_OSpringBarleyGrass:

return 307;

case tov\_OSBarleySilage:

return 103;

case tov\_OSpringBarleyClover:

return 308;

case tov\_OPermanentGrassGrazed:

return 340;

case tov\_OCloverGrassGrazed2:

return 365;

case tov\_OCloverGrassSilage1:

return 366;

case tov\_OSeedGrass2:

return 394;

case tov\_NaturalGrass:

return 400;

case tov\_None:

return 401;

case tov\_NoGrowth:

return 402;

case tov\_WWheatPControl:

return 601;

case tov\_WWheatPToxicControl:

return 602;

case tov\_WWheatPTreatment:

return 603;

case tov\_AgroChemIndustryCereal:

return 604;

case tov\_WinterWheatShort:

return 605;

case tov\_MaizeSilage:

return 606;

case tov\_FodderGrass:

return 607;

case tov\_SpringBarleyPTreatment:

return 608;

case tov\_SpringBarleySKManagement:

return 612;

case tov\_OSpringBarleyExt:

return 609;

case tov\_OMaizeSilage:

return 610;

case tov\_Undefined:

return 999;

default: // No matching code so we need an error message of some kind

sprintf( error\_num, "%d", VegReference );

g\_msg->Warn( WARN\_FILE, "LE\_TypeClass::BackTranslateVegTypes(): ""Unknown vegetation type:", error\_num );

exit( 1 );

}

}

|  |  |  |  |  |  |
| --- | --- | --- | --- | --- | --- |
| TTypesOfLandscapeElement LE\_TypeClass::TranslateEleTypes | ( | int | *EleReference* | ) |  |

References g\_msg, tole\_ActivePit, tole\_AmenityGrass, tole\_BareRock, tole\_BeetleBank, tole\_Building, tole\_BuiltUpWithParkland, tole\_Coast, tole\_ConiferousForest, tole\_Copse, tole\_DeciduousForest, tole\_Field, tole\_FieldBoundary, tole\_Freshwater, tole\_Garden, tole\_Heath, tole\_HedgeBank, tole\_Hedges, tole\_LargeRoad, tole\_Marsh, tole\_MixedForest, tole\_NaturalGrass, tole\_Orchard, tole\_OrchardBand, tole\_OrchardGrass, tole\_Parkland, tole\_PermanentSetaside, tole\_PermPasture, tole\_PermPastureLowYield, tole\_PermPastureTussocky, tole\_PitDisused, tole\_Railway, tole\_River, tole\_RiversidePlants, tole\_RiversideTrees, tole\_RoadsideVerge, tole\_RuralResidential, tole\_Saltwater, tole\_SandDune, tole\_Scrub, tole\_SmallRoad, tole\_StoneWall, tole\_Suburban, tole\_Track, tole\_UnsprayedFieldMargin, tole\_Urban, tole\_UrbanNoVeg, tole\_UrbanPark, tole\_YoungForest, MapErrorMsg::Warn(), and WARN\_FILE.

Referenced by Landscape::ReadPolys(), and Landscape::TranslateEleTypes().

{

static char error\_num[ 20 ];

// This returns the vegetation type (or crop type) as applicable

switch ( EleReference ) {

case 5:

return tole\_Building;

case 6:

return tole\_UrbanNoVeg;

case 7:

return tole\_Suburban;

case 8:

return tole\_RuralResidential;

case 10:

return tole\_Urban;

case 11:

return tole\_Garden;

case 12:

return tole\_AmenityGrass;

case 13:

return tole\_RoadsideVerge;

case 14:

return tole\_Parkland;

case 15:

return tole\_StoneWall;

case 16:

return tole\_BuiltUpWithParkland;

case 17:

return tole\_UrbanPark;

case 20:

return tole\_Field;

case 26:

return tole\_PermPastureLowYield;

case 27:

return tole\_PermPastureTussocky;

case 30:

return tole\_Field;

case 31:

return tole\_UnsprayedFieldMargin;

case 33:

return tole\_PermanentSetaside;

case 35:

return tole\_PermPasture;

case 40:

return tole\_DeciduousForest;

case 41:

return tole\_Copse;

case 50:

return tole\_ConiferousForest;

case 55:

return tole\_YoungForest;

case 56:

return tole\_Orchard;

case 57:

return tole\_OrchardBand;

case 58:

return tole\_OrchardGrass;

case 59:

return tole\_BareRock;

case 60:

return tole\_MixedForest;

case 70:

return tole\_Scrub;

case 75:

return tole\_PitDisused;

case 80:

return tole\_Saltwater;

case 90:

return tole\_Freshwater;

case 94:

return tole\_Heath;

case 95:

return tole\_Marsh;

case 99: // This is stream less than 2m

case 96:

return tole\_River;

case 97:

return tole\_RiversideTrees;

case 98:

return tole\_RiversidePlants;

case 100:

return tole\_Coast;

case 101:

return tole\_SandDune;

case 110:

return tole\_NaturalGrass;

case 115:

return tole\_ActivePit;

case 118:

return tole\_Railway;

case 120:

case 121:

return tole\_LargeRoad;

case 122:

return tole\_SmallRoad;

case 123:

return tole\_Track;

case 130:

return tole\_Hedges;

case 140:

return tole\_HedgeBank;

case 141:

return tole\_BeetleBank;

case 160:

return tole\_FieldBoundary;

// case 999: return tole\_Foobar;

// !! type unknown - should not happen

default:

sprintf( error\_num, "%d", EleReference );

g\_msg->Warn( WARN\_FILE, "LE\_TypeClass::TranslateEleTypes(): ""Unknown landscape element type:", error\_num );

exit( 1 );

}

}

|  |  |  |  |  |  |
| --- | --- | --- | --- | --- | --- |
| TTypesOfVegetation LE\_TypeClass::TranslateVegTypes | ( | int | *VegReference* | ) |  |

References g\_msg, tov\_AgroChemIndustryCereal, tov\_Carrots, tov\_CloverGrassGrazed1, tov\_CloverGrassGrazed2, tov\_FieldPeas, tov\_FodderBeet, tov\_FodderGrass, tov\_Maize, tov\_MaizeSilage, tov\_NaturalGrass, tov\_None, tov\_Oats, tov\_OBarleyPeaCloverGrass, tov\_OCarrots, tov\_OCloverGrassGrazed1, tov\_OCloverGrassGrazed2, tov\_OCloverGrassSilage1, tov\_OFieldPeas, tov\_OFieldPeasSilage, tov\_OGrazingPigs, tov\_OMaizeSilage, tov\_OOats, tov\_OPermanentGrassGrazed, tov\_OPotatoes, tov\_OSeedGrass1, tov\_OSeedGrass2, tov\_OSpringBarley, tov\_OSpringBarleyClover, tov\_OSpringBarleyExt, tov\_OSpringBarleyGrass, tov\_OTriticale, tov\_OWinterBarley, tov\_OWinterBarleyExt, tov\_OWinterRape, tov\_OWinterRye, tov\_OWinterWheatUndersown, tov\_PermanentGrassGrazed, tov\_PermanentGrassLowYield, tov\_PermanentGrassTussocky, tov\_PermanentSetaside, tov\_Potatoes, tov\_PotatoesIndustry, tov\_SeedGrass1, tov\_SeedGrass2, tov\_Setaside, tov\_SpringBarley, tov\_SpringBarleyCloverGrass, tov\_SpringBarleyGrass, tov\_SpringBarleyPeaCloverGrassStrigling, tov\_SpringBarleyPTreatment, tov\_SpringBarleySeed, tov\_SpringBarleySilage, tov\_SpringBarleySKManagement, tov\_SpringBarleyStrigling, tov\_SpringBarleyStriglingCulm, tov\_SpringBarleyStriglingSingle, tov\_SpringRape, tov\_SpringWheat, tov\_Triticale, tov\_Undefined, tov\_WinterBarley, tov\_WinterRape, tov\_WinterRye, tov\_WinterWheat, tov\_WinterWheatShort, tov\_WinterWheatStrigling, tov\_WinterWheatStriglingCulm, tov\_WinterWheatStriglingSingle, tov\_WWheatPControl, tov\_WWheatPToxicControl, tov\_WWheatPTreatment, tov\_YoungForest, MapErrorMsg::Warn(), and WARN\_FILE.

Referenced by Farm::HandleEvents(), Farm::InitiateManagement(), and Landscape::TranslateVegTypes().

{

char error\_num[ 20 ];

// This returns the vegetation type (or crop type) as applicable

switch ( VegReference ) {

case 1:

return tov\_SpringBarley;

case 2:

return tov\_WinterBarley;

case 3:

return tov\_SpringWheat;

case 4:

return tov\_WinterWheat;

case 5:

return tov\_WinterRye;

case 6:

return tov\_Oats;

case 7:

return tov\_Triticale;

case 8:

return tov\_Maize;

case 13:

return tov\_SpringBarleySeed;

case 14: return tov\_SpringBarleyStrigling;

case 15: return tov\_SpringBarleyStriglingSingle;

case 16: return tov\_SpringBarleyStriglingCulm;

case 17: return tov\_WinterWheatStrigling;

case 18: return tov\_WinterWheatStriglingSingle;

case 19: return tov\_WinterWheatStriglingCulm;

case 21:

return tov\_SpringRape;

case 22:

return tov\_WinterRape;

case 30:

return tov\_FieldPeas;

case 50:

return tov\_Setaside;

case 54:

return tov\_PermanentSetaside;

case 55:

return tov\_YoungForest;

case 60:

return tov\_FodderBeet;

case 65:

return tov\_CloverGrassGrazed1;

case 92:

return tov\_PotatoesIndustry;

case 93:

return tov\_Potatoes;

case 94:

return tov\_SeedGrass1;

case 102:

return tov\_OWinterBarley;

case 611:

return tov\_OWinterBarleyExt;

case 103:

return tov\_SpringBarleySilage;

case 105:

return tov\_OWinterRye;

case 106:

return tov\_OFieldPeasSilage;

case 107:

return tov\_SpringBarleyGrass;

case 108:

return tov\_SpringBarleyCloverGrass;

case 113:

return tov\_OBarleyPeaCloverGrass;

case 114:

return tov\_SpringBarleyPeaCloverGrassStrigling;

case 122:

return tov\_OWinterRape;

case 140:

return tov\_PermanentGrassGrazed;

case 141:

return tov\_PermanentGrassLowYield;

case 142:

return tov\_PermanentGrassTussocky;

case 165:

return tov\_CloverGrassGrazed2;

case 194:

return tov\_SeedGrass2;

case 201:

return tov\_OSpringBarley;

case 204:

return tov\_OWinterWheatUndersown;

case 206:

return tov\_OOats;

case 207:

return tov\_OTriticale;

case 230:

return tov\_OFieldPeas;

case 265:

return tov\_OCloverGrassGrazed1;

case 270:

return tov\_OCarrots;

case 271:

return tov\_Carrots;

case 273:

return tov\_OGrazingPigs;

case 293:

return tov\_OPotatoes;

case 294:

return tov\_OSeedGrass1;

case 307:

return tov\_OSpringBarleyGrass;

case 308:

return tov\_OSpringBarleyClover;

case 340:

return tov\_OPermanentGrassGrazed;

case 365:

return tov\_OCloverGrassGrazed2;

case 366:

return tov\_OCloverGrassSilage1;

case 394:

return tov\_OSeedGrass2;

case 400:

return tov\_NaturalGrass;

case 401:

return tov\_None;

case 601:

return tov\_WWheatPControl;

case 602:

return tov\_WWheatPToxicControl;

case 603:

return tov\_WWheatPTreatment;

case 604:

return tov\_AgroChemIndustryCereal;

case 605:

return tov\_WinterWheatShort;

case 606:

return tov\_MaizeSilage;

case 607:

return tov\_FodderGrass;

case 608:

return tov\_SpringBarleyPTreatment;

case 609:

return tov\_OSpringBarleyExt;

case 610:

return tov\_OMaizeSilage;

case 612:

return tov\_SpringBarleySKManagement;

case 999:

return tov\_Undefined;

default: // No matching code so we need an error message of some kind

sprintf( error\_num, "%d", VegReference );

g\_msg->Warn( WARN\_FILE, "LE\_TypeClass::TranslateVegTypes(): ""Unknown vegetation type:", error\_num );

exit( 1 );

}

}

|  |  |  |  |  |  |
| --- | --- | --- | --- | --- | --- |
| int LE\_TypeClass::VegTypeToCurveNum | ( | TTypesOfVegetation | *VegReference* | ) |  |

---

The documentation for this class was generated from the following files:

- elements.h
- elements.cpp


- LE\_TypeClass
- Generated on Thu Jan 10 2013 13:15:36 for ALMaSS Skylark ODdox by
   1.8.1.1
