# Supplementary material for: Modelling Skylarks (Alauda arvensis) to Predict Impacts of Changes in Land Management and Policy: Development and Testing of an Agent-Based Model
Source: PLoS One. 2013 Jun 6;8(6):e65803. doi: 10.1371/journal.pone.0065803 (PMC3675089; doi:10.1371/journal.pone.0065803)
Supplement: Supporting Information S4 — The skylark ODdox as a zipped archive. (ZIP) [file pone.0065803.s004.zip › Skylark_ODdox/class_landscape.html]

ALMaSS Skylark ODdox: Landscape Class Reference


|  |
| --- |
| ALMaSS Skylark ODdox  2.0 |


- Main Page
- Related Pages
- Classes
- Files

- Class List
- Class Index
- Class Hierarchy
- Class Members

Public Member Functions |
Protected Member Functions |
Protected Attributes |
Private Attributes

Landscape Class Reference

The landscape class containing all environmental and topographical data.
More...

`#include <landscape.h>`

List of all members.

|  |  |
| --- | --- |
| Public Member Functions | |
| int | BackTranslateEleTypes (TTypesOfLandscapeElement EleReference) |
| int | BackTranslateVegTypes (TTypesOfVegetation VegReference) |
| void | BuildingDesignationCalc () |
|  | used to calculate whether a building is rural or town - for rodenticide use |
| void | CalculateCentroids (void) |
| void | CentroidSpiralOut (int a\_polyref, int &a\_x, int &a\_y) |
| void | CorrectCoords (int &x, int &y) |
|  | Function to prevent wrap around errors with co-ordinates. |
| int | CorrectHeight (int y) |
| int | CorrectWidth (int x) |
| void | DumpAllSymbolsAndExit (const char \*a\_dumpfile) |
| void | DumpCentroids (void) |
| void | DumpMapInfoByArea (const char \*a\_filename, bool a\_append, bool a\_dump\_zero\_areas, bool a\_write\_veg\_names) |
| void | DumpPublicSymbols (const char \*a\_dumpfile, CfgSecureLevel a\_level) |
| void | DumpVegAreaData (int a\_day) |
| char \* | EventtypeToString (int a\_event) |
| void | FillVegAreaData () |
| double | GetVegArea (int v) |
| void | IncTreatCounter (int a\_treat) |
|  | Landscape (const char \*a\_configfile, const char \*a\_errorfile) |
| int | MagicMapP2PolyRef (int a\_magic) |
| char \* | PolytypeToString (TTypesOfLandscapeElement a\_le\_type) |
| bool | ReadSymbols (const char \*a\_cfgfile) |
| void | SetLESignal (int a\_polyref, LE\_Signal a\_signal) |
| void | SetPolyMaxMinExtents (void) |
| void | SkylarkEvaluation (SkTerritories \*a\_skt) |
| int | SupplyCountryDesig (int a\_x, int a\_y) |
| double | SupplyDayDegrees (int a\_polyref) |
| int | SupplyDayInMonth (void) |
| long | SupplyDayInYear (void) |
| int | SupplyDaylength (void) |
| int | SupplyDaylength (long a\_date) |
| double | SupplyDeadBiomass (int a\_polyref) |
| double | SupplyDeadBiomass (int a\_x, int a\_y) |
| int | SupplyElementSubType (int a\_polyref) |
| int | SupplyElementSubType (int a\_x, int a\_y) |
| TTypesOfLandscapeElement | SupplyElementType (int a\_polyref) |
| TTypesOfLandscapeElement | SupplyElementType (int a\_x, int a\_y) |
| TTypesOfLandscapeElement | SupplyElementTypeCC (int a\_x, int a\_y) |
| TTypesOfLandscapeElement | SupplyElementTypeFromVector (unsigned int a\_index) |
| int | SupplyFarmArea (int a\_polyref) |
| int | SupplyFarmIntensity (int a\_x, int a\_y) |
| int | SupplyFarmIntensity (int a\_polyref) |
| int | SupplyFarmOwner (int a\_x, int a\_y) |
| int | SupplyFarmOwner (int a\_polyref) |
| int | SupplyFarmOwnerIndex (int a\_x, int a\_y) |
| int | SupplyFarmOwnerIndex (int a\_polyref) |
| TTypesOfFarm | SupplyFarmType (int a\_polyref) |
| TTypesOfFarm | SupplyFarmType (int a\_x, int a\_y) |
| long | SupplyGlobalDate (void) |
| double | SupplyGlobalRadiation () |
| double | SupplyGlobalRadiation (long a\_date) |
| bool | SupplyGrazingPressure (int a\_polyref) |
| bool | SupplyGrazingPressure (int a\_x, int a\_y) |
| bool | SupplyGrazingPressureVector (unsigned int a\_index) |
| double | SupplyGreenBiomass (int a\_polyref) |
| double | SupplyGreenBiomass (int a\_x, int a\_y) |
| bool | SupplyHasTramlines (int a\_x, int a\_y) |
| bool | SupplyHasTramlines (int a\_polyref) |
| double | SupplyInsects (int a\_polyref) |
| double | SupplyInsects (int a\_x, int a\_y) |
| bool | SupplyIsGrass (int a\_polyref) |
| bool | SupplyJustMown (int a\_polyref) |
| bool | SupplyJustMownVector (unsigned int a\_index) |
| int | SupplyJustSprayed (int a\_polyref) |
| int | SupplyJustSprayed (int a\_x, int a\_y) |
| int | SupplyJustSprayedVector (unsigned int a\_index) |
| double | SupplyLAGreen (int a\_polyref) |
| double | SupplyLAGreen (int a\_x, int a\_y) |
| int | SupplyLargestPolyNumUsed () |
| int | SupplyLastTreatment (int a\_polyref, int \*a\_index) |
| int | SupplyLastTreatment (int a\_x, int a\_y, int \*a\_index) |
| double | SupplyLATotal (int a\_x, int a\_y) |
| int | SupplyLECount (void) |
| int | SupplyLENext (void) |
| LE \* | SupplyLEPointer (int a\_polyref) |
| void | SupplyLEReset (void) |
| LE\_Signal | SupplyLESignal (int a\_polyref) |
| int \* | SupplyMagicMapP (int a\_x, int a\_y) |
| double | SupplyMeanTemp (long a\_date, unsigned int a\_period) |
| int | SupplyMonth (void) |
| unsigned int | SupplyNumberOfPolygons (void) |
| double | SupplyPesticide (int a\_x, int a\_y) |
| double | SupplyPesticide (int a\_polyref) |
| int | SupplyPesticideCell (int a\_polyref) |
| TTypesOfPesticide | SupplyPesticideType (void) |
| double | SupplyPolygonAreaVector (int a\_polyref) |
|  | Returns the area of a polygon using the vector index as a reference. |
| int | SupplyPolyRef (int a\_x, int a\_y) |
| int | SupplyPolyRefCC (int a\_x, int a\_y) |
| int | SupplyPolyRefIndex (int a\_x, int a\_y) |
| int | SupplyPolyRefVector (unsigned int a\_index) |
| double | SupplyRain (void) |
| double | SupplyRain (long a\_date) |
| double | SupplyRainPeriod (long a\_date, int a\_period) |
| int | SupplyRoadWidth (int, int) |
| double | SupplyRodenticide (int a\_x, int a\_y) |
| bool | SupplyShouldSpray () |
| int | SupplySimAreaHeight (void) |
| int | SupplySimAreaWidth (void) |
| bool | SupplySkScrapes (int a\_polyref) |
| bool | SupplySnowcover (void) |
| bool | SupplySnowcover (long a\_date) |
| double | SupplyTemp (void) |
| double | SupplyTemp (long a\_date) |
| double | SupplyTempPeriod (long a\_date, int a\_period) |
| double | SupplyTrafficLoad (int a\_x, int a\_y) |
| double | SupplyTrafficLoad (int a\_polyref) |
| int | SupplyTreeAge (int a\_Polyref) |
| int | SupplyTreeAge (int, int) |
| int | SupplyTreeHeight (int, int) |
| int | SupplyTreeHeight (int) |
| int | SupplyUnderGrowthWidth (int, int) |
| int | SupplyUnderGrowthWidth (int) |
| int | SupplyValidX (int a\_polyref) |
| int | SupplyValidY (int a\_polyref) |
| int | SupplyVegAge (int a\_Polyref) |
| int | SupplyVegAge (int a\_x, int a\_y) |
| double | SupplyVegBiomass (int a\_polyref) |
| double | SupplyVegBiomass (int a\_x, int a\_y) |
| double | SupplyVegBiomassVector (unsigned int a\_index) |
| double | SupplyVegCover (int a\_polyref) |
| double | SupplyVegCover (int a\_x, int a\_y) |
| double | SupplyVegCoverVector (unsigned int a\_index) |
| int | SupplyVegDensity (int a\_polyref) |
| int | SupplyVegDensity (int a\_x, int a\_y) |
| double | SupplyVegDigestability (int a\_polyref) |
| double | SupplyVegDigestability (int a\_x, int a\_y) |
| double | SupplyVegDigestabilityVector (unsigned int a\_index) |
| double | SupplyVegHeight (int a\_polyref) |
| double | SupplyVegHeight (int a\_x, int a\_y) |
| double | SupplyVegHeightVector (unsigned int a\_index) |
| bool | SupplyVegPatchy (int a\_polyref) |
| bool | SupplyVegPatchy (int a\_x, int a\_y) |
| TTypesOfVegetation | SupplyVegType (int a\_x, int a\_y) |
| TTypesOfVegetation | SupplyVegType (int polyref) |
| TTypesOfVegetation | SupplyVegTypeVector (unsigned int a\_index) |
| const char \* | SupplyVersion (void) |
| double | SupplyWeedBiomass (int a\_polyref) |
| double | SupplyWeedBiomass (int a\_x, int a\_y) |
| double | SupplyWind (void) |
| double | SupplyWind (long a\_date) |
| int | SupplyWindDirection (void) |
| double | SupplyWindPeriod (long a\_date, int a\_period) |
| int | SupplyYear (void) |
| int | SupplyYearNumber (void) |
| void | Tick (void) |
| void | TickHour (void) |
| void | TickMinute (void) |
| TTypesOfLandscapeElement | TranslateEleTypes (int EleReference) |
| TTypesOfVegetation | TranslateVegTypes (int VegReference) |
| void | TurnTheWorld (void) |
| char \* | VegtypeToString (TTypesOfVegetation a\_veg) |
| void | Warn (const char \*a\_msg1, const char \*a\_msg2) |
|  | ~Landscape (void) |

|  |  |
| --- | --- |
| Protected Member Functions | |
| void | AddBeetleBanks (void) |
| void | AddGreenElement (LE \*a\_green) |
| int | AddToClusterList (int \*, int \*, int \*, int \*, int \*) |
| void | AxisLoop (int a\_poly, int \*x1, int \*y1, int a\_axis) |
| void | BeetleBankAdd (int x, int y, int angle, int length, LE \*a\_field) |
| bool | BeetleBankPossible (LE \*a\_field) |
| void | BorderAdd (LE \*a\_field) |
| bool | BorderNeed (TTypesOfLandscapeElement a\_letype) |
| void | BorderScan (LE \*a\_field) |
| void | BorderScan2 (LE \*a\_poly) |
| bool | BorderStep (int a\_fieldpoly, int a\_borderpoly, int \*a\_x, int \*a\_y) |
| bool | BorderTest (int a\_fieldpoly, int a\_borderpoly, int a\_x, int a\_y) |
| void | ChangeMapMapping (void) |
| bool | CIPELandscapeMaker () |
| void | CountMapSquares (void) |
| void | DumpMap (const char \*a\_filename) |
| void | DumpMapGraphics (const char \*a\_filename) |
| void | DumpTreatCounters (const char \*a\_filename) |
| void | EventDump (int x, int y, int x2, int y2) |
| bool | FindFieldCenter (LE \*a\_field, int \*x, int \*y) |
| int | FindLongestAxis (int \*x, int \*y, int \*a\_length) |
| bool | FindValidXY (int a\_field, int &a\_x, int &a\_y) |
| void | ForceArea (void) |
| Point | GetNextSeed (int) |
| void | hb\_Add (void) |
| void | hb\_AddNewHedgebanks (int a\_orig\_poly\_num) |
| void | hb\_Cleanup (void) |
| void | hb\_ClearPolygon (int a\_poly\_num) |
| void | hb\_DownPolyNumbers (void) |
| bool | hb\_FindBoundingBox (int a\_poly\_num) |
| void | hb\_FindHedges (void) |
| void | hb\_GenerateHBPolys (void) |
| bool | hb\_HasNeighbourColor (int a\_x, int a\_y, int a\_neighbour\_color) |
| bool | hb\_HasOtherNeighbour (int a\_x, int a\_y) |
| bool | hb\_MapBorder (int a\_x, int a\_y) |
| void | hb\_MarkTheBresenhamWay (void) |
| void | hb\_MarkTopFromLocalMax (int a\_color) |
| int | hb\_MaxUnpaintedNegNeighbour (int a\_x, int a\_y) |
| void | hb\_PaintBorder (int a\_color) |
| bool | hb\_PaintWhoHasNeighbourColor (int a\_neighbour\_color, int a\_new\_color) |
| void | hb\_ResetColorBits (void) |
| void | hb\_RestoreHedgeCore (int a\_orig\_poly\_number) |
| int | hb\_StripingDist (void) |
| void | hb\_UpPolyNumbers (void) |
| void | MakeCluster (void) |
|  | Reset all polygons natural grazing level to zero. |
| void | ModifyPolyRef (int \*) |
| LE \* | NewElement (TTypesOfLandscapeElement a\_type) |
| void | OrchardBorderAdd (LE \*a\_field) |
| void | PolysDump (const char \*a\_filename) |
| void | PolysRemoveInvalid (void) |
| void | PolysValidate (bool a\_exit\_on\_invalid) |
| Point | RandomLocation (void) |
| void | ReadInput (int \*, int \*, int \*, Point \*) |
| void | ReadPolys (const char \*a\_polyfile) |
| bool | StepOneValid (int a\_polyindex, int a\_x, int a\_y, int step) |
| void | TestCropManagement (void) |
| bool | UMarginTest (int a\_fieldpoly, int a\_borderpoly, int a\_x, int a\_y, int a\_width) |
| void | UnsprayedMarginAdd (LE \*a\_field) |
| void | UnsprayedMarginScan (LE \*a\_field, int a\_width) |
| void | VegDump (int x, int y) |

|  |  |
| --- | --- |
| Protected Attributes | |
| int | hb\_border\_pixels |
| int | hb\_core\_pixels |
| int | hb\_first\_free\_poly\_num |
| vector< int > | hb\_hedges |
| int | hb\_height |
| int \* | hb\_map |
| int | hb\_max\_x |
| int | hb\_max\_y |
| int | hb\_min\_x |
| int | hb\_min\_y |
| vector< LE \* > | hb\_new\_hbs |
| int | hb\_size |
| int | hb\_width |
| int | le\_signal\_index |
| int | m\_LargestPolyNumUsed |
| TTypesOfPesticide | m\_PesticideType |
|  | An attribute to hold the pesticide type being tested, if there is one, if not default is -1. |
| bool | m\_toxShouldSpray |
| int | m\_treatment\_counts [last\_treatment] |
| int | m\_x\_add [8] |
| int | m\_y\_add [8] |

|  |  |
| --- | --- |
| Private Attributes | |
| double \* | l\_vegtype\_areas |
| vector< LE \* > | m\_elems |
| FarmManager \* | m\_FarmManager |
| int \* | m\_farmmapping |
| int | m\_height |
| int | m\_height10 |
| RasterMap \* | m\_land |
| RodenticideManager \* | m\_RodenticideManager |
| char | m\_versioninfo [30] |
| int | m\_width |
| int | m\_width10 |

---

## Detailed Description

The landscape class containing all environmental and topographical data.

---

## Constructor & Destructor Documentation

|  |  |  |  |  |  |
| --- | --- | --- | --- | --- | --- |
| Landscape::~Landscape | ( | void |  | ) |  |

References cfg\_rodenticide\_enable, DumpTreatCounters(), g\_crops, g\_date, g\_letype, g\_msg, g\_pest, g\_weather, l\_map\_dump\_treatcounts\_enable, l\_map\_dump\_treatcounts\_file, l\_vegtype\_areas, m\_elems, m\_FarmManager, m\_farmmapping, m\_land, m\_polymapping, m\_RodenticideManager, CfgBool::value(), and CfgStr::value().

{

if ( l\_map\_dump\_treatcounts\_enable.value() ) {

DumpTreatCounters( l\_map\_dump\_treatcounts\_file.value() );

}

for ( unsigned int i = 0; i < m\_elems.size(); i++ )

delete m\_elems[ i ];

free( m\_farmmapping );

free( m\_polymapping );

free( l\_vegtype\_areas );

delete m\_land;

//delete g\_rotation;

delete g\_crops;

delete g\_letype;

delete g\_weather;

delete g\_date;

delete g\_pest;

if (cfg\_rodenticide\_enable.value()) delete m\_RodenticideManager;

delete m\_FarmManager;

delete g\_msg; // Must be last.

}

|  |  |  |  |
| --- | --- | --- | --- |
| Landscape::Landscape | ( | const char \* | *a\_configfile*, |
|  |  | const char \* | *a\_errorfile* |
|  | ) |  |  |

Rodenticide handling code. If enabled then rodenticide mapping provides access to predicted relative density of poisoned rodents per unit area.

References AddBeetleBanks(), BorderAdd(), CalculateCentroids(), cfg\_AddBeetleBanks, cfg\_CalculateCentroids, cfg\_dumpvegjan, cfg\_dumpvegjanfile, cfg\_dumpvegjune, cfg\_dumpvegjunefile, cfg\_HedgeSubtypeMaximum, cfg\_HedgeSubtypeMinimum, cfg\_pesticidetesttype, cfg\_rodenticide\_enable, ChangeMapMapping(), CountMapSquares(), DumpCentroids(), DumpMap(), DumpMapGraphics(), ForceArea(), g\_cfg, g\_crops, g\_date, g\_letype, g\_map\_le\_borders, g\_map\_le\_unsprayedmargins, g\_map\_maxpolyref, g\_map\_orchards\_borders, g\_map\_orchardsborderwidth, g\_msg, g\_pest, g\_SpeedyDivides, g\_weather, hb\_Add(), hb\_first\_free\_poly\_num, FarmManager::InitiateManagement(), l\_map\_art\_hedgebanks, l\_map\_cropcurves\_file, l\_map\_dump\_enable, l\_map\_dump\_exit, l\_map\_dump\_gfx\_enable, l\_map\_dump\_gfx\_file, l\_map\_dump\_map\_file, l\_map\_dump\_poly\_file, l\_map\_dump\_veg\_enable, l\_map\_map\_file, l\_map\_no\_pesticide\_fields, l\_map\_poly\_file, l\_map\_print\_version\_info, l\_map\_weather\_file, l\_vegtype\_areas, last\_treatment, m\_elems, m\_FarmManager, m\_height, m\_height10, m\_land, m\_LargestPolyNumUsed, m\_PesticideType, m\_RodenticideManager, m\_toxShouldSpray, m\_treatment\_counts, m\_versioninfo, m\_width, m\_width10, m\_x\_add, m\_y\_add, OrchardBorderAdd(), PolysDump(), PolysRemoveInvalid(), PolysValidate(), random(), ReadPolys(), Configurator::ReadSymbols(), Calendar::Reset(), SetLESignal(), SetPolyMaxMinExtents(), MapErrorMsg::SetWarnLevel(), SupplyElementType(), SupplyLECount(), SupplyLENext(), SupplyLEReset(), SupplyLESignal(), Tick(), tole\_Field, tole\_HedgeBank, tole\_Hedges, tole\_Orchard, tov\_Undefined, UnsprayedMarginAdd(), CfgInt::value(), CfgBool::value(), CfgStr::value(), version\_date, version\_major, version\_minor, version\_revision, MapErrorMsg::Warn(), WARN\_ALL, WARN\_BUG, and WARN\_FILE.

{

// Set up globals

g\_landscape\_p = this;

for (int i=1; i<=2000; i++) {

g\_SpeedyDivides[i] = 1/ double (i);

}

int x\_add [8] = { 1, 1, 0, -1, -1, -1, 0, 1 }; // W,SW,S,SE,E,NE,N,NW

int y\_add [8] = { 0, -1, -1, -1, 0, 1, 1, 1 };

for (int i=0; i<8; i++) {

m\_x\_add[i]=x\_add[i];

m\_y\_add[i]=y\_add[i];

}

sprintf( m\_versioninfo, "%d.%d.%d :: %s", version\_major, version\_minor, version\_revision, version\_date );

if ( l\_map\_print\_version\_info.value() ) {

printf( "This program uses the Landscape simulator V%s\n", m\_versioninfo );

}

//g\_cfg->DumpAllSymbolsAndExit( "allsymbols.cfg" );

// Must come first. Used by the configurator below.

g\_msg = new MapErrorMsg( a\_errorfile );

g\_msg->SetWarnLevel( WARN\_ALL );

//g\_cfg->DumpAllSymbolsAndExit( "allsymbols.cfg" );

// Configurator instantiation is automatic.

g\_cfg->ReadSymbols( a\_configfile );

//g\_cfg->DumpAllSymbolsAndExit( "allsymbols.cfg" );

// For testing.

//g\_cfg->DumpPublicSymbols( "publicsymbols.cfg", CFG\_PUBLIC );

//exit(1);

g\_date = new Calendar;

g\_weather = new Weather( l\_map\_weather\_file.value() );

g\_letype = new LE\_TypeClass;

g\_crops = new CropData( l\_map\_cropcurves\_file.value() );

hb\_first\_free\_poly\_num = g\_map\_maxpolyref.value();

m\_LargestPolyNumUsed = -1;

// Outdated. Use with extreme caution.

//g\_rotation = new CropRotation( g\_crops->GetNumCrops() );

// Reset treatment counters.

for ( int i = 0; i < last\_treatment; i++ ) {

m\_treatment\_counts[ i ] = 0;

}

m\_FarmManager = new FarmManager();

ReadPolys( l\_map\_poly\_file.value() );

m\_land = new RasterMap( l\_map\_map\_file.value() );

m\_width = m\_land->MapWidth();

m\_height = m\_land->MapHeight();

m\_width10 = 10 \* m\_width;

m\_height10 = 10 \* m\_height;

PolysValidate( false );

// Add artificial hedgebanks to the hedges in the landscape,

// if requested.

if ( l\_map\_art\_hedgebanks.value() ) {

hb\_Add();

}

g\_pest = new Pesticide( m\_land, this );

m\_toxShouldSpray = false;

// Validate polygons, ie. ensure those reference in the

// polygon file also shows up in the map.

PolysValidate( false );

PolysRemoveInvalid();

PolysValidate( true );

// ChangeMapMapping() also enters a valid starting

// coordinate for the border generating farm method below.

ChangeMapMapping();

if ( g\_map\_le\_borders.value() ) {

// Generate border around each \*farm\* landscape element.

for ( unsigned int i = 0; i < m\_elems.size(); i++ ) {

if ( m\_elems[ i ]->GetBorder() != NULL ) {

// Border around this element, so must be a farm field.

BorderAdd( m\_elems[ i ] );

}

}

}

// Some special code to 'soften' the edges of orchards

if ( g\_map\_orchards\_borders.value() ) {

// Generate border around each \*farm\* landscape element.

for ( unsigned int i = 0; i < m\_elems.size(); i++ ) {

if ( m\_elems[ i ]->GetElementType() == tole\_Orchard )

{

for (int Z=0; Z<g\_map\_orchardsborderwidth.value(); Z++)

{

OrchardBorderAdd( m\_elems[ i ] );

}

}

}

}

// Unsprayed Margin Code....

if ( g\_map\_le\_unsprayedmargins.value() ) {

CountMapSquares();

ForceArea();

// Generate border around each \*farm\* landscape element.

for ( unsigned int i = 0; i < m\_elems.size(); i++ ) {

if ( m\_elems[ i ]->GetUnsprayedMarginPolyRef() != -1 ) {

// But not if the field is too small to have them (<1Ha)

if( m\_elems[ i ]->GetArea()>10000) {

// Border around this element, so must be a farm field.

UnsprayedMarginAdd( m\_elems[ i ] );

} else m\_elems[ i ]->SetUnsprayedMarginPolyRef( -1 );

}

}

}

// to here

CountMapSquares();

ForceArea();

if (cfg\_AddBeetleBanks.value()) {

AddBeetleBanks();

}

if ( l\_map\_dump\_gfx\_enable.value() ) {

DumpMapGraphics( l\_map\_dump\_gfx\_file.value() );

}

if ( l\_map\_dump\_enable.value() ) {

DumpMap( l\_map\_dump\_map\_file.value() );

PolysDump( l\_map\_dump\_poly\_file.value() );

if ( l\_map\_dump\_exit.value() ) {

g\_msg->Warn( WARN\_FILE, "Landscape::Landscape(): ""Normal exit after map dump.", "" );

exit( 0 );

}

}

/\*'''''''''''''''''' CIPE LANDSCAPE MAKER CODE HERE //'''''''''''''''''''''

if ( l\_map\_CIPEmaker\_enable.value() ) {

CIPELandscapeMaker();

if ( l\_map\_dump\_exit.value() ) {

g\_msg->Warn( WARN\_FILE, "Landscape::Landscape(): ""Normal exit after map dump.", "" );

exit( 0 );

}

}

//'''''''''''''''''' CIPE LANDSCAPE MAKER CODE ABOVE //'''''''''''''''''''''

\*/

// Set the type of hedgebanks.

int l\_subtype = cfg\_HedgeSubtypeMinimum.value();

for ( unsigned int i = 0; i < m\_elems.size(); i++ ) {

if ( m\_elems[ i ]->GetElementType() == tole\_HedgeBank ) {

m\_elems[ i ]->SetSubType( l\_subtype );

if ( ++l\_subtype >= cfg\_HedgeSubtypeMaximum.value() )

l\_subtype = cfg\_HedgeSubtypeMinimum.value();

}

}

// And another to set the type of hedges

// \*\*\*CJT\*\*\* 2003-12-02

l\_subtype = 0;

for ( unsigned int i = 0; i < m\_elems.size(); i++ ) {

if ( m\_elems[ i ]->GetElementType() == tole\_Hedges ) {

m\_elems[ i ]->SetSubType( l\_subtype );

if ( ++l\_subtype >= 3 )

l\_subtype = 0;

}

}

m\_FarmManager->InitiateManagement();

g\_date->Reset();

/\*

if ( g\_farm\_test\_crop.value() ) {

TestCropManagement();

exit( 0 );

}

\*/

// Set up treatment flags

// Reset internal state for the LE loop generator.

// Compulsory!

SupplyLEReset();

// Get number of \*all\* landscape elements.

int l\_count = SupplyLECount();

// Now loop through then.

for ( int i = 0; i < l\_count; i++ ) {

// Fetch next LE by its polygon reference number. Alternative

// loop mechanism: This will return -1 at end-of-loop.

int a\_poly = SupplyLENext();

// Skip uninteresting polygons by type, ownership,

// phase of the moon, whatever.

// if ( these\_are\_not\_the\_droids\_we\_are\_looking\_for( a\_poly )) {

if ( SupplyElementType( a\_poly ) != tole\_Field )

continue;

// Example: Set x% of them to ignore insecticide of all types.

if ( random( 100 ) < l\_map\_no\_pesticide\_fields.value() ) {

// Get current signal mask for polygon.

LE\_Signal l\_signal = SupplyLESignal( a\_poly );

// Logical OR in/AND out the signals you are interested in.

// The current signals are at the top of elements.h

//l\_signal |= LE\_SIG\_NO\_INSECTICIDE | LE\_SIG\_NO\_SYNG\_INSECT | LE\_SIG\_NO\_HERBICIDE | LE\_SIG\_NO\_FUNGICIDE | LE\_SIG\_NO\_GROWTH\_REG;

//l\_signal |= LE\_SIG\_NO\_INSECTICIDE | LE\_SIG\_NO\_SYNG\_INSECT | LE\_SIG\_NO\_HERBICIDE;

// Write the mask back out to the polygon.

SetLESignal( a\_poly, l\_signal );

}

}

l\_vegtype\_areas = ( double \* ) malloc( sizeof( double ) \* ( tov\_Undefined + 1 ) );

if ( l\_vegtype\_areas == NULL ) {

g\_msg->Warn( WARN\_BUG, "Landscape::Landscape(): Out of memory!", "" );

exit( 1 );

}

FILE \* outf;

if ( cfg\_dumpvegjan.value() ) {

outf=fopen(cfg\_dumpvegjanfile.value(), "w" );

if (!outf) {

g\_msg->Warn( WARN\_FILE, "Landscape::DumpMapInfoByArea(): ""Unable to create file", cfg\_dumpvegjanfile.value() );

exit( 1 );

} else

fclose( outf );

}

if ( cfg\_dumpvegjune.value() ) {

outf=fopen(cfg\_dumpvegjunefile.value(), "w" );

if (!outf) {

g\_msg->Warn( WARN\_FILE, "Landscape::DumpMapInfoByArea(): ""Unable to create file", cfg\_dumpvegjunefile.value() );

exit( 1 );

} else

fclose( outf );

}

// Dump veg information if necessary

if ( l\_map\_dump\_veg\_enable.value() ) {

FILE \* f;

f=fopen("VegDump.txt", "w" );

if ( !f ) {

g\_msg->Warn( WARN\_BUG, "Landscape::Landscape(): VegDump.txt could not be created", "" );

exit( 1 );

}

fprintf( f, "Day\tHeight\tBiomass\tDensity\tCover\tWeedBiomass\ttovNum\tinsect biomass\tLATotal\tDigestability\tGreen Bio.\tDead Bio.\n" );

fclose( f );

f=fopen("EventDump.txt", "w" );

if ( !f ) {

g\_msg->Warn( WARN\_BUG, "Landscape::Landscape(): EventDump.txt could not be created", "" );

exit( 1 );

}

fclose( f );

}

SetPolyMaxMinExtents();

if (cfg\_CalculateCentroids.value())

{

CalculateCentroids();

DumpCentroids();

}

// If we are testing a pesticide then set the enum attribute

m\_PesticideType = (TTypesOfPesticide) cfg\_pesticidetesttype.value();

if (cfg\_rodenticide\_enable.value())

{

m\_RodenticideManager = new RodenticideManager("BaitLocations\_input.txt",this);

}

// Run a year to remove any start up effects

for ( unsigned int i = 0; i < 365; i++ )

Tick();

}

---

## Member Function Documentation

|  |  |  |  |  |  |  |  |
| --- | --- | --- | --- | --- | --- | --- | --- |
| |  |  |  |  |  |  | | --- | --- | --- | --- | --- | --- | | void Landscape::AddBeetleBanks | ( | void |  | ) |  | | protected |

Beetle-bank addition - tests whether we can add a bank to this field, and then decides where to put it an adds it.

References BeetleBankPossible(), cfg\_BeetleBankChance, g\_msg, m\_elems, random(), tole\_Field, CfgInt::value(), MapErrorMsg::Warn(), and WARN\_MSG.

Referenced by Landscape().

{

// for each element, if it is a field then maybe it should have a beetle bank

int BBs=0;

unsigned sz=(unsigned)m\_elems.size();

for (unsigned i=0; i<sz; i++) {

if (m\_elems[ i ]->GetElementType() == tole\_Field) {

if (random(100)<cfg\_BeetleBankChance.value()) {

if (BeetleBankPossible( m\_elems[ i ] )) BBs++;

}

}

}

char str[25];

sprintf(str,"%d",BBs);

g\_msg->Warn( WARN\_MSG, "Landscape::AddBeetleBanks(): BeetleBanks successfully added:", str );

}

|  |  |  |  |  |  |  |  |
| --- | --- | --- | --- | --- | --- | --- | --- |
| |  |  |  |  |  |  | | --- | --- | --- | --- | --- | --- | | void Landscape::AddGreenElement | ( | LE \* | *a\_green* | ) |  | | protected |

|  |  |  |  |  |  |  |  |  |  |  |  |  |  |  |  |  |  |  |  |  |  |  |  |  |  |
| --- | --- | --- | --- | --- | --- | --- | --- | --- | --- | --- | --- | --- | --- | --- | --- | --- | --- | --- | --- | --- | --- | --- | --- | --- | --- |
| |  |  |  |  | | --- | --- | --- | --- | | int Landscape::AddToClusterList | ( | int \* | , | |  |  | int \* | , | |  |  | int \* | , | |  |  | int \* | , | |  |  | int \* |  | |  | ) |  |  | | protected |

|  |  |  |  |  |  |  |  |  |  |  |  |  |  |  |  |  |  |  |  |  |  |
| --- | --- | --- | --- | --- | --- | --- | --- | --- | --- | --- | --- | --- | --- | --- | --- | --- | --- | --- | --- | --- | --- |
| |  |  |  |  | | --- | --- | --- | --- | | void Landscape::AxisLoop | ( | int | *a\_poly*, | |  |  | int \* | *x1*, | |  |  | int \* | *y1*, | |  |  | int | *a\_axis* | |  | ) |  |  | | protected |

Starting at a\_x,a\_y each location is tested along a vector given by m\_x\_add & m\_y\_add until we step outside the polygon. a\_x & a\_y are modified on return.

References CorrectHeight(), CorrectWidth(), m\_x\_add, m\_y\_add, and SupplyPolyRef().

Referenced by FindFieldCenter(), and FindLongestAxis().

{

int ap1=a\_poly;

while (ap1==a\_poly) {

\*(a\_x)+=m\_x\_add[a\_axis];

\*(a\_y)+=m\_y\_add[a\_axis];

int cory=CorrectHeight( \*(a\_y)); // Ensure we don't step off the world y

int corx=CorrectWidth( \*(a\_x)); // Ensure we don't step off the world x

ap1=SupplyPolyRef(corx,cory);

}

}

|  |  |  |  |  |  |  |  |
| --- | --- | --- | --- | --- | --- | --- | --- |
| |  |  |  |  |  |  | | --- | --- | --- | --- | --- | --- | | int Landscape::BackTranslateEleTypes | ( | TTypesOfLandscapeElement | *EleReference* | ) |  | | inline |

References LE\_TypeClass::BackTranslateEleTypes(), and g\_letype.

{

return g\_letype->BackTranslateEleTypes( EleReference );

}

|  |  |  |  |  |  |  |  |
| --- | --- | --- | --- | --- | --- | --- | --- |
| |  |  |  |  |  |  | | --- | --- | --- | --- | --- | --- | | int Landscape::BackTranslateVegTypes | ( | TTypesOfVegetation | *VegReference* | ) |  | | inline |

References LE\_TypeClass::BackTranslateVegTypes(), and g\_letype.

{

return g\_letype->BackTranslateVegTypes( VegReference );

}

|  |  |  |  |  |  |  |  |  |  |  |  |  |  |  |  |  |  |  |  |  |  |  |  |  |  |
| --- | --- | --- | --- | --- | --- | --- | --- | --- | --- | --- | --- | --- | --- | --- | --- | --- | --- | --- | --- | --- | --- | --- | --- | --- | --- |
| |  |  |  |  | | --- | --- | --- | --- | | void Landscape::BeetleBankAdd | ( | int | *x*, | |  |  | int | *y*, | |  |  | int | *angle*, | |  |  | int | *length*, | |  |  | LE \* | *a\_field* | |  | ) |  |  | | protected |

References LE::AddArea(), cfg\_BeetleBankWidth, m\_elems, m\_land, m\_LargestPolyNumUsed, m\_polymapping, m\_x\_add, m\_y\_add, NewElement(), RasterMap::Put(), LE::SetArea(), LE::SetMapValid(), LE::SetPoly(), LE::SetValidXY(), start, tole\_BeetleBank, and CfgInt::value().

Referenced by BeetleBankPossible().

{

// Need to get a new number

int newpoly=++m\_LargestPolyNumUsed;

// Make the new landscape element

LE \* BeetleBank = NewElement( tole\_BeetleBank );

m\_polymapping[ m\_LargestPolyNumUsed ] = (int) m\_elems.size();

m\_elems.resize( m\_elems.size() + 1 );

m\_elems[ m\_elems.size() - 1 ] = BeetleBank;

BeetleBank->SetPoly( m\_LargestPolyNumUsed );

// write lengthx12m to the map at alignment angle

int area=0;

int angle2=0;

int width=cfg\_BeetleBankWidth.value();

if (a\_angle==0) angle2=2;

int start=(int)(a\_length\*0.1);

for (int i=start; i<a\_length; i++) {

for (int w=0-width; w<width; w++) {

int tx=w\*m\_x\_add[angle2];

int ty=w\*m\_y\_add[angle2];

m\_land->Put( tx+a\_x+i\*m\_x\_add[a\_angle], ty+a\_y+i\*m\_y\_add[a\_angle], newpoly );

m\_land->Put( tx+a\_x-i\*m\_x\_add[a\_angle], ty+a\_y-i\*m\_y\_add[a\_angle], newpoly );

area+=2;

a\_field->AddArea( -2.0 );

}

}

BeetleBank->SetArea( double(area) );

BeetleBank->SetValidXY( a\_x+start\*m\_x\_add[a\_angle], a\_y+start\*m\_y\_add[a\_angle] );

BeetleBank->SetMapValid(true);

}

|  |  |  |  |  |  |  |  |
| --- | --- | --- | --- | --- | --- | --- | --- |
| |  |  |  |  |  |  | | --- | --- | --- | --- | --- | --- | | bool Landscape::BeetleBankPossible | ( | LE \* | *a\_field* | ) |  | | protected |

Beetle bank placement rules are:  
No bank if the total bank area is going to be >=5% of the field area  
No bank if the field is < 1Ha   
No bank if the breadth of the field is < 100m

References BeetleBankAdd(), cfg\_BeetleBankMaxArea, cfg\_BeetleBankWidth, FindFieldCenter(), FindLongestAxis(), LE::GetArea(), LE::GetValidX(), LE::GetValidY(), CfgInt::value(), and CfgFloat::value().

Referenced by AddBeetleBanks().

{

int farea=(int)a\_field->GetArea();

if (farea<10000) return false;

int cx=a\_field->GetValidX();

int cy=a\_field->GetValidY();

// The centroid is the only estimate we have (and it at least should be in the field).

// So start here and find the centre

if (!FindFieldCenter(a\_field, &cx, &cy)) return false;

// now get the alignment

int length=0;

int alignment=FindLongestAxis(&cx, &cy, &length);

// reduce length by 20%

length=int(length\*0.8);

int area=2\*length\*cfg\_BeetleBankWidth.value(); // 12m wide fixed size

if (area>(farea\*cfg\_BeetleBankMaxArea.value())) return false;

// Must be small engough so lets draw it

BeetleBankAdd(cx, cy, alignment, length , a\_field);

return true;

}

|  |  |  |  |  |  |  |  |
| --- | --- | --- | --- | --- | --- | --- | --- |
| |  |  |  |  |  |  | | --- | --- | --- | --- | --- | --- | | void Landscape::BorderAdd | ( | LE \* | *a\_field* | ) |  | | protected |

References BorderScan(), g\_msg, LE::GetValidX(), LE::GetValidY(), hb\_first\_free\_poly\_num, m\_elems, m\_polymapping, NewElement(), LE::SetArea(), LE::SetBorder(), LE::SetPoly(), tole\_FieldBoundary, MapErrorMsg::Warn(), and WARN\_BUG.

Referenced by Landscape().

{

int x = a\_field->GetValidX();

int y = a\_field->GetValidY();

if ( ( x == -1 ) || ( y == -1 ) ) {

g\_msg->Warn( WARN\_BUG, "Landscape::BorderAdd(): Uninitialized border coordinate!", "" );

exit( 1 );

}

LE \* border = NewElement( tole\_FieldBoundary );

a\_field->SetBorder( border );

m\_polymapping[ hb\_first\_free\_poly\_num ] = (int) m\_elems.size();

m\_elems.resize( m\_elems.size() + 1 );

m\_elems[ m\_elems.size() - 1 ] = border;

border->SetPoly( hb\_first\_free\_poly\_num++ );

border->SetArea( 0.0 );

BorderScan( a\_field );

}

|  |  |  |  |  |  |  |  |
| --- | --- | --- | --- | --- | --- | --- | --- |
| |  |  |  |  |  |  | | --- | --- | --- | --- | --- | --- | | bool Landscape::BorderNeed | ( | TTypesOfLandscapeElement | *a\_letype* | ) |  | | protected |

References g\_msg, tole\_ActivePit, tole\_AmenityGrass, tole\_BareRock, tole\_BeetleBank, tole\_Building, tole\_BuiltUpWithParkland, tole\_Coast, tole\_ConiferousForest, tole\_Copse, tole\_DeciduousForest, tole\_Field, tole\_FieldBoundary, tole\_Freshwater, tole\_Garden, tole\_Heath, tole\_HedgeBank, tole\_Hedges, tole\_LargeRoad, tole\_Marsh, tole\_MixedForest, tole\_NaturalGrass, tole\_Orchard, tole\_OrchardBand, tole\_OrchardGrass, tole\_Parkland, tole\_PermanentSetaside, tole\_PermPasture, tole\_PermPastureLowYield, tole\_PermPastureTussocky, tole\_PitDisused, tole\_Railway, tole\_River, tole\_RiversidePlants, tole\_RiversideTrees, tole\_RoadsideVerge, tole\_RuralResidential, tole\_Saltwater, tole\_SandDune, tole\_Scrub, tole\_SmallRoad, tole\_StoneWall, tole\_Suburban, tole\_Track, tole\_UnsprayedFieldMargin, tole\_Urban, tole\_UrbanNoVeg, tole\_UrbanPark, tole\_YoungForest, MapErrorMsg::Warn(), and WARN\_BUG.

Referenced by BorderTest().

{

static char error\_num[ 20 ];

bool AddBorder = false;

switch ( a\_letype ) {

// No border is needed toward these neighbouring element types.

case tole\_Hedges:

case tole\_HedgeBank:

case tole\_BeetleBank:

case tole\_RoadsideVerge:

case tole\_Marsh:

case tole\_RiversidePlants:

case tole\_UnsprayedFieldMargin:

case tole\_OrchardBand:

case tole\_OrchardGrass:

break;

case tole\_NaturalGrass:

case tole\_Railway:

case tole\_FieldBoundary:

case tole\_Scrub:

case tole\_Field:

case tole\_PermanentSetaside:

case tole\_PermPasture:

case tole\_PermPastureTussocky:

case tole\_PermPastureLowYield:

case tole\_PitDisused:

case tole\_RiversideTrees:

case tole\_DeciduousForest:

case tole\_MixedForest:

case tole\_YoungForest:

case tole\_ConiferousForest:

case tole\_StoneWall:

case tole\_Garden:

case tole\_Track:

case tole\_SmallRoad:

case tole\_LargeRoad:

case tole\_Building:

case tole\_Urban:

case tole\_ActivePit:

case tole\_Freshwater:

case tole\_River:

case tole\_Saltwater:

case tole\_Coast:

case tole\_BareRock:

case tole\_Heath:

case tole\_Orchard:

case tole\_AmenityGrass:

case tole\_Parkland:

case tole\_UrbanNoVeg:

case tole\_UrbanPark:

case tole\_Suburban:

case tole\_RuralResidential:

case tole\_BuiltUpWithParkland:

case tole\_SandDune:

case tole\_Copse:

AddBorder = true;

break;

default:

sprintf( error\_num, "%d", a\_letype );

g\_msg->Warn( WARN\_BUG, "Landscape::BorderNeed(): Unknown element type:", error\_num );

exit( 1 );

}

return AddBorder;

}

|  |  |  |  |  |  |  |  |
| --- | --- | --- | --- | --- | --- | --- | --- |
| |  |  |  |  |  |  | | --- | --- | --- | --- | --- | --- | | void Landscape::BorderScan | ( | LE \* | *a\_field* | ) |  | | protected |

References LE::AddArea(), BorderStep(), BorderTest(), LE::GetBorder(), LE::GetPoly(), LE::GetValidX(), LE::GetValidY(), m\_land, m\_polymapping, and RasterMap::Put().

Referenced by BorderAdd().

{

LE \* border = a\_field->GetBorder();

int fieldpoly = a\_field->GetPoly();

int borderpoly = border->GetPoly();

int borderindex = m\_polymapping[ borderpoly ];

int fieldindex = m\_polymapping[ fieldpoly ];

int notforever = 5000;

// These two will be modified through pointer operations

// in BorderStep().

int x = a\_field->GetValidX();

int y = a\_field->GetValidY();

while ( --notforever ) {

// Check if this position should be made into a border.

if ( BorderTest( fieldindex, borderindex, x, y ) ) {

// Add this pixel to the border element in the big map.

m\_land->Put( x, y, borderindex );

a\_field->AddArea( -1.0 );

/\* if ( l\_map\_exit\_on\_zero\_area.value() && ! (--a\_field->m\_squares\_in\_map) ) { char polynum[20];

sprintf(polynum, "%d", a\_field->GetPoly()); g\_msg->Warn( WARN\_FILE,

"Landscape::BorderScan(): Polygon reached zero area " "when adding border. Poly num: ", polynum ); exit( 1 ); } \*/

border->AddArea( 1.0 );

//border->m\_squares\_in\_map++;

};

// Step to next coordinate. Quit when done.

if ( !BorderStep( fieldindex, borderindex, & x, & y ) ) return;

}

}

|  |  |  |  |  |  |  |  |
| --- | --- | --- | --- | --- | --- | --- | --- |
| |  |  |  |  |  |  | | --- | --- | --- | --- | --- | --- | | void Landscape::BorderScan2 | ( | LE \* | *a\_poly* | ) |  | | protected |

References LE::AddArea(), BorderStep(), BorderTest(), LE::GetBorder(), LE::GetPoly(), LE::GetValidX(), LE::GetValidY(), m\_land, m\_polymapping, RasterMap::Put(), and StepOneValid().

Referenced by OrchardBorderAdd().

{

LE \* border = a\_poly->GetBorder();

int fieldpoly = a\_poly->GetPoly();

int borderpoly = border->GetPoly();

int borderindex = m\_polymapping[ borderpoly ];

int fieldindex = m\_polymapping[ fieldpoly ];

int notforever = 5000;

// These two will be modified through pointer operations

// in BorderStep().

int x = a\_poly->GetValidX();

int y = a\_poly->GetValidY();

int oldx=x;

int oldy=y;

bool changed = false;

while ( --notforever )

{

// Check if this position should be made into a border.

if ( BorderTest( fieldindex, borderindex, x, y ) )

{

// Add this pixel to the border element in the big map.

m\_land->Put( x, y, borderindex );

a\_poly->AddArea( -1.0 );

/\* if ( l\_map\_exit\_on\_zero\_area.value() && ! (--a\_field->m\_squares\_in\_map) ) { char polynum[20];

sprintf(polynum, "%d", a\_field->GetPoly()); g\_msg->Warn( WARN\_FILE,

"Landscape::BorderScan(): Polygon reached zero area " "when adding border. Poly num: ", polynum ); exit( 1 ); } \*/

border->AddArea( 1.0 );

if (!changed) changed = true;

};

// Step to next coordinate. Quit when done.

if ( !BorderStep( fieldindex, borderindex, & x, & y ) )

{

int step=1;

while ((!StepOneValid(fieldindex, oldx, oldy, step)) && (step++<10));

return;

}

}

}

|  |  |  |  |  |  |  |  |  |  |  |  |  |  |  |  |  |  |  |  |  |  |
| --- | --- | --- | --- | --- | --- | --- | --- | --- | --- | --- | --- | --- | --- | --- | --- | --- | --- | --- | --- | --- | --- |
| |  |  |  |  | | --- | --- | --- | --- | | bool Landscape::BorderStep | ( | int | *a\_fieldpoly*, | |  |  | int | *a\_borderpoly*, | |  |  | int \* | *a\_x*, | |  |  | int \* | *a\_y* | |  | ) |  |  | | protected |

References RasterMap::Get(), m\_land, RasterMap::MapHeight(), and RasterMap::MapWidth().

Referenced by BorderScan(), BorderScan2(), and UnsprayedMarginScan().

{

int index;

int x\_add[ 8 ] = { 1, 1, 0, -1, -1, -1, 0, 1 };

int y\_add[ 8 ] = { 0, -1, -1, -1, 0, 1, 1, 1 };

int width = m\_land->MapWidth();

int height = m\_land->MapHeight();

int i = 7, counter = 8;

bool running = true;

// First scan for another pixel that belongs to this field.

while ( running )

{

if ( !( ( \* a\_x ) + x\_add[ i ] >= width ) && !( ( \* a\_x ) + x\_add[ i ] < 0 ) && !( ( \* a\_y ) + y\_add[ i ] >= height ) && !( ( \* a\_y ) + y\_add[ i ] < 0 ) )

{

index = m\_land->Get( ( \* a\_x ) + x\_add[ i ], ( \* a\_y ) + y\_add[ i ] );

if ( index == a\_fieldindex )

{

// Found the first field pixel while scanning around always

// in the same direction.

running = false;

}

}

if ( --i < 0 ) {

// Didn't find any of our pixels. We are in a blind alley. Exit

// gracefully.

return false; // Signal done scanning this field.

}

}

// Now scan around from our present facing direction and find the border

// (if any).

while ( --counter )

{

if ( !( ( \* a\_x ) + x\_add[ i ] >= width ) && !( ( \* a\_x ) + x\_add[ i ] < 0 ) && !( ( \* a\_y ) + y\_add[ i ] >= height ) && !( ( \* a\_y ) + y\_add[ i ] < 0 ) )

{

index = m\_land->Get( ( \* a\_x ) + x\_add[ i ], ( \* a\_y ) + y\_add[ i ] );

if ( index == a\_fieldindex )

{

if ( --i < 0 ) i = 7;

continue;

}

}

// Aha! This pixel is not ours. Step one step in the

// opposite(!) direction. If that pixel is ours, then

// modify hotspot coordinates and exit.

if ( ++i > 7 ) i = 0;

if ( !( ( \* a\_x ) + x\_add[ i ] + 1 > width ) && !( ( \* a\_x ) + x\_add[ i ] < 0 ) && !( ( \* a\_y ) + y\_add[ i ] + 1 > height ) &&

!( ( \* a\_y ) + y\_add[ i ] < 0 ) && ( m\_land->Get( ( \* a\_x ) + x\_add[ i ], ( \* a\_y ) + y\_add[ i ] ) == a\_fieldindex ) )

{

( \* a\_x ) += x\_add[ i ];

( \* a\_y ) += y\_add[ i ];

return true;

}

}

return false;

}

|  |  |  |  |  |  |  |  |  |  |  |  |  |  |  |  |  |  |  |  |  |  |
| --- | --- | --- | --- | --- | --- | --- | --- | --- | --- | --- | --- | --- | --- | --- | --- | --- | --- | --- | --- | --- | --- |
| |  |  |  |  | | --- | --- | --- | --- | | bool Landscape::BorderTest | ( | int | *a\_fieldpoly*, | |  |  | int | *a\_borderpoly*, | |  |  | int | *a\_x*, | |  |  | int | *a\_y* | |  | ) |  |  | | protected |

References BorderNeed(), RasterMap::Get(), m\_elems, m\_land, RasterMap::MapHeight(), and RasterMap::MapWidth().

Referenced by BorderScan(), and BorderScan2().

{

int index;

int x\_add[ 8 ] = { 1, 1, 0, -1, -1, -1, 0, 1 };

int y\_add[ 8 ] = { 0, -1, -1, -1, 0, 1, 1, 1 };

int width = m\_land->MapWidth();

int height = m\_land->MapHeight();

// Scan anti-clockwise from center pixel coordinate.

for ( unsigned int i = 0; i < 8; i++ ) {

if ( ( a\_x + x\_add[ i ] >= width ) || ( a\_x + x\_add[ i ] < 0 ) || ( a\_y + y\_add[ i ] >= height )

|| ( a\_y + y\_add[ i ] < 0 ) ) {

return true;

}

//continue;

index = m\_land->Get( a\_x + x\_add[ i ], a\_y + y\_add[ i ] );

if ( ( index != a\_fieldindex ) && ( index != a\_borderindex ) )

{

if ( BorderNeed( m\_elems[ index ]->GetElementType() ) ) return true;

else return false;

}

}

return false;

}

|  |  |  |  |  |
| --- | --- | --- | --- | --- |
| void Landscape::BuildingDesignationCalc | ( |  | ) |  |

used to calculate whether a building is rural or town - for rodenticide use

References cfg\_mintownbuildingdistance, cfg\_mintownbuildingnumber, m\_elems, tole\_Building, and CfgInt::value().

Referenced by CalculateCentroids().

{

for (int p = 0; p< (int)m\_elems.size(); p++)

{

if (m\_elems[p]->GetElementType() == tole\_Building)

{

int cx = m\_elems[p]->GetCentroidX();

int cy = m\_elems[p]->GetCentroidY();

int near = 0;

for (int j = 0; j< (int)m\_elems.size(); j++)

{

if (m\_elems[p]->GetElementType() == tole\_Building)

{

int nx = m\_elems[j]->GetCentroidX();

int ny = m\_elems[j]->GetCentroidY();

int dx =abs(cx-nx);

int dy =abs(cy-ny);

if ((dx < cfg\_mintownbuildingdistance.value()) && (dy < cfg\_mintownbuildingdistance.value())) near++;

if (near > cfg\_mintownbuildingdistance.value()) break;

}

}

if (near <= cfg\_mintownbuildingnumber.value()) m\_elems[p]->SetCountryDesignation(1); // Not enough buildings close by, so it is a country building

}

}

}

|  |  |  |  |  |  |
| --- | --- | --- | --- | --- | --- |
| void Landscape::CalculateCentroids | ( | void |  | ) |  |

Finds a location inside each polygon as a roughly calculated centre point. The point will be within the polygon. This also stores Max/Min coordinates for each polygon, forming a rectangle around it.

References BuildingDesignationCalc(), CentroidSpiralOut(), and m\_elems.

Referenced by Landscape().

{

// For each polygon

for (int p = 0; p< (int)m\_elems.size(); p++)

{

// Calcuate the actual centre

int x1 = m\_elems[p]->GetMinX();

int y1 = m\_elems[p]->GetMinY();

int x2 = m\_elems[p]->GetMaxX();

int y2 = m\_elems[p]->GetMaxY();

int midx = (x1+x2)/2;

int midy = (y1+y2)/2;

// Now from midx & midy we move outwards in concentric circles until we find a location that matches our polyref.

int polyref = m\_elems[p]->GetPoly();

CentroidSpiralOut(polyref, midx, midy);

m\_elems[p]->SetCentroid(midx,midy);

}

BuildingDesignationCalc();

}

|  |  |  |  |
| --- | --- | --- | --- |
| void Landscape::CentroidSpiralOut | ( | int | *a\_polyref*, |
|  |  | int & | *a\_x*, |
|  |  | int & | *a\_y* |
|  | ) |  |  |

References CorrectCoords(), m\_width, and SupplyPolyRef().

Referenced by CalculateCentroids().

{

if (SupplyPolyRef(a\_x,a\_y) == a\_polyref) return; // Found it so return

// Otherwise its not found so we need to start to spiral out

int loop = 1;

int sx=a\_x;

int sy=a\_y;

do {

a\_y = sy-loop;

for (int i = 0-loop; i<= loop; i++)

{

a\_x = sx+i;

CorrectCoords(a\_x, a\_y);

if (SupplyPolyRef(a\_x,a\_y) == a\_polyref) return; // Found it so return

}

a\_y = sy+loop;

for (int i = 0-loop; i<= loop; i++)

{

a\_x = sx+i;

CorrectCoords(a\_x, a\_y);

if (SupplyPolyRef(a\_x,a\_y) == a\_polyref) return; // Found it so return

}

a\_x = sx+loop;

for (int j = 0-(loop-1); j< loop; j++)

{

a\_y = sy+j;

CorrectCoords(a\_x, a\_y);

if (SupplyPolyRef(a\_x,a\_y) == a\_polyref) return; // Found it so return

}

a\_x = sx-loop;

for (int j = 0-(loop-1); j< loop; j++)

{

a\_y = sy+j;

CorrectCoords(a\_x, a\_y);

if (SupplyPolyRef(a\_x,a\_y) == a\_polyref) return; // Found it so return

}

loop++;

} while (loop<m\_width);

exit(0);

}

|  |  |  |  |  |  |  |  |
| --- | --- | --- | --- | --- | --- | --- | --- |
| |  |  |  |  |  |  | | --- | --- | --- | --- | --- | --- | | void Landscape::ChangeMapMapping | ( | void |  | ) |  | | protected |

Our map is an array of polygon indentifiers, where we really want to know the associated landscape element of a X-Y coordinate pair.   
Changing this to m\_elems[] indices will save us one redirection when inquiring information from the landscape, and only costs us the fixed translation step performed here at startup.

References FarmManager::AddField(), cfg\_l\_treatment\_size, cfg\_l\_treatment\_x, cfg\_l\_treatment\_y, cfg\_l\_usecustompoly, FarmManager::ConnectFarm(), g\_msg, RasterMap::Get(), l\_map\_check\_polygon\_xref, m\_elems, m\_FarmManager, m\_farmmapping, m\_land, m\_polymapping, RasterMap::MapHeight(), RasterMap::MapWidth(), PEST\_GRIDSIZE, PEST\_GRIDSIZE\_POW2, RasterMap::Put(), FarmManager::RemoveField(), SupplyPolyRef(), CfgInt::value(), CfgBool::value(), MapErrorMsg::Warn(), and WARN\_FILE.

Referenced by Landscape().

{

int mapwidth = m\_land->MapWidth();

int mapheight = m\_land->MapHeight();

int pest\_map\_width = mapwidth >> PEST\_GRIDSIZE\_POW2;

if ( mapwidth & ( PEST\_GRIDSIZE - 1 ) ) pest\_map\_width++;

int oldindex = -1;

for ( int x = 0; x < mapwidth; x++ ) {

for ( int y = 0; y < mapheight; y++ ) {

int polynum = m\_land->Get( x, y );

m\_elems[ m\_polymapping[ polynum ]]->SetMapIndex( m\_polymapping[ polynum ] );

m\_elems[ m\_polymapping[ polynum ]]->SetMapValid( true );

// Do the translation.

m\_land->Put( x, y, m\_polymapping[ polynum ] );

// This coordinate is now valid. Throw these coordinates into

// the associated landscape element.

int index = m\_polymapping[ SupplyPolyRef( x, y ) ];

if ( index != oldindex ) {

m\_elems[ index ]->SetValidXY( x, y );

int l\_x = x >> PEST\_GRIDSIZE\_POW2;

int l\_y = y >> PEST\_GRIDSIZE\_POW2;

int pref = l\_y \* pest\_map\_width + l\_x;

m\_elems[ index ]->SetPesticideCell( pref );

oldindex = index;

}

}

}

// Check that all of the polygons are mentioned in the map.

if ( l\_map\_check\_polygon\_xref.value() ) {

for ( unsigned int i = 0; i < m\_elems.size(); i++ ) {

if ( !m\_elems[ i ]->GetMapValid() ) {

char poly[ 20 ];

sprintf( poly, "%d", m\_elems[ i ]->GetPoly() );

g\_msg->Warn( WARN\_FILE, "Landscape::ChangeMapMapping(): ""Polygon number referenced but not in map file: ", poly );

exit( 1 );

}

}

}

// Now we can add some customised functionality

if ( cfg\_l\_usecustompoly.value() ) {

int x, y;

for ( unsigned int i = 0; i < m\_elems.size(); i++ ) {

// Line below is defined for each special case

int farmref = m\_elems[ i ]->GetOwnerFile();

if ( farmref != -1 ) {

x = m\_elems[ i ]->GetValidX();

y = m\_elems[ i ]->GetValidY();

if ( ( x >= cfg\_l\_treatment\_x.value() ) && ( x < ( cfg\_l\_treatment\_x.value() + cfg\_l\_treatment\_size.value() ) )

&& ( y >= cfg\_l\_treatment\_y.value() ) && ( y < ( cfg\_l\_treatment\_y.value() + cfg\_l\_treatment\_size.value() ) ) ) {

// Line below is defined for each special case

int OwnerIndex = m\_farmmapping[ farmref ];

// Remove the ownership from current farm

m\_FarmManager->RemoveField(OwnerIndex, m\_elems[ i ]);

// In case farm zero has not been recorded yet

if ( -1 == m\_farmmapping[ 0 ] )

m\_farmmapping[ 0 ] = m\_FarmManager->ConnectFarm( 0 );

// Now change the ownership to farm zero

OwnerIndex = m\_farmmapping[ 0 ];

m\_FarmManager->AddField(OwnerIndex, m\_elems[ i ], 0);

}

}

}

}

}

|  |  |  |  |  |  |  |
| --- | --- | --- | --- | --- | --- | --- |
| |  |  |  |  |  | | --- | --- | --- | --- | --- | | bool Landscape::CIPELandscapeMaker | ( |  | ) |  | | protected |

|  |  |  |  |  |  |  |  |  |  |  |  |  |  |
| --- | --- | --- | --- | --- | --- | --- | --- | --- | --- | --- | --- | --- | --- |
| |  |  |  |  | | --- | --- | --- | --- | | void Landscape::CorrectCoords | ( | int & | *x*, | |  |  | int & | *y* | |  | ) |  |  | | inline |

Function to prevent wrap around errors with co-ordinates.

m\_width10 & m\_height10 are used to avoid problems with co-ordinate values that are very large. Problems will only occur if coords passed are >10x the world width or height.

References m\_height, m\_height10, m\_width, and m\_width10.

Referenced by CentroidSpiralOut().

{

x=(m\_width10+x)%m\_width;

y=(m\_height10+y)%m\_height;

}

|  |  |  |  |  |  |  |  |
| --- | --- | --- | --- | --- | --- | --- | --- |
| |  |  |  |  |  |  | | --- | --- | --- | --- | --- | --- | | int Landscape::CorrectHeight | ( | int | *y* | ) |  | | inline |

References m\_height, and m\_height10.

Referenced by AxisLoop().

{

return (m\_height10+y)%m\_height;

}

|  |  |  |  |  |  |  |  |
| --- | --- | --- | --- | --- | --- | --- | --- |
| |  |  |  |  |  |  | | --- | --- | --- | --- | --- | --- | | int Landscape::CorrectWidth | ( | int | *x* | ) |  | | inline |

References m\_width, and m\_width10.

Referenced by AxisLoop().

{

return (m\_width10+x)%m\_width;

}

|  |  |  |  |  |  |  |  |
| --- | --- | --- | --- | --- | --- | --- | --- |
| |  |  |  |  |  |  | | --- | --- | --- | --- | --- | --- | | void Landscape::CountMapSquares | ( | void |  | ) |  | | protected |

References RasterMap::Get(), m\_elems, m\_land, RasterMap::MapHeight(), and RasterMap::MapWidth().

Referenced by Landscape().

{

int mapwidth = m\_land->MapWidth();

int mapheight = m\_land->MapHeight();

for ( unsigned int i = 0; i < m\_elems.size(); i++ ) {

m\_elems[i]->SetArea(0);

m\_elems[ i ]->m\_squares\_in\_map=0;

}

for ( int x = 0; x < mapwidth; x++ ) {

for ( int y = 0; y < mapheight; y++ ) {

int l\_ele = m\_land->Get( x, y );

m\_elems[ l\_ele ]->m\_squares\_in\_map++;

}

}

}

|  |  |  |  |  |  |  |  |
| --- | --- | --- | --- | --- | --- | --- | --- |
| |  |  |  |  |  |  | | --- | --- | --- | --- | --- | --- | | void Landscape::DumpAllSymbolsAndExit | ( | const char \* | *a\_dumpfile* | ) |  | | inline |

References Configurator::DumpAllSymbolsAndExit(), and g\_cfg.

{

g\_cfg->DumpAllSymbolsAndExit( a\_dumpfile );

}

|  |  |  |  |  |  |
| --- | --- | --- | --- | --- | --- |
| void Landscape::DumpCentroids | ( | void |  | ) |  |

References m\_elems.

Referenced by Landscape().

{

ofstream centroidfile("PolygonCentroids.txt", ios::out);

centroidfile<<"Polyref"<<'\t'<<"CX"<<'\t'<<"CY"<<'\t'<<"Type"<<'\t'<<"Area"<<'\t';

for (int p = 0; p< (int)m\_elems.size(); p++)

{

centroidfile<<m\_elems[p]->GetPoly()<<'\t'<<m\_elems[p]->GetCentroidX()<<'\t'<<m\_elems[p]->GetCentroidY()<<'\t'<<m\_elems[p]->GetElementType()<<'\t'<<m\_elems[p]->GetArea()<<'\t'<<m\_elems[p]->GetCountryDesignation()<<endl;

}

centroidfile.close();

}

|  |  |  |  |  |  |  |  |
| --- | --- | --- | --- | --- | --- | --- | --- |
| |  |  |  |  |  |  | | --- | --- | --- | --- | --- | --- | | void Landscape::DumpMap | ( | const char \* | *a\_filename* | ) |  | | protected |

References g\_msg, RasterMap::GetID(), RasterMap::GetMagicP(), l\_map\_renumberpolys, m\_elems, m\_height, m\_land, m\_width, CfgBool::value(), MapErrorMsg::Warn(), and WARN\_FILE.

Referenced by Landscape().

{

FILE \* l\_file;

l\_file = fopen(a\_filename, "wb" );

if ( !l\_file ) {

g\_msg->Warn( WARN\_FILE, "Landscape::DumpMap(): Unable to open file", a\_filename );

exit( 0 );

}

int \* l\_map = m\_land->GetMagicP( 0, 0 );

char \* l\_id = m\_land->GetID();

fwrite( l\_id, 1, 12, l\_file );

fwrite( & m\_height, 1, sizeof( int ), l\_file );

if ( !l\_map\_renumberpolys.value() ) {

for ( int i = 0; i < m\_width \* m\_height; i++ ) {

int l\_poly = m\_elems[ l\_map[ i ]]->GetPoly();

fwrite( & l\_poly, 1, sizeof( int ), l\_file );

}

} else {

for ( int i = 0; i < m\_width \* m\_height; i++ ) {

// If we want to re-number the polygons then we only need the index

int l\_poly = l\_map[ i ];

fwrite( & l\_poly, 1, sizeof( int ), l\_file );

}

fclose( l\_file );

}

}

|  |  |  |  |  |  |  |  |
| --- | --- | --- | --- | --- | --- | --- | --- |
| |  |  |  |  |  |  | | --- | --- | --- | --- | --- | --- | | void Landscape::DumpMapGraphics | ( | const char \* | *a\_filename* | ) |  | | protected |

References g\_msg, m\_height, m\_width, MapDumpColours, SupplyElementType(), SupplyVegHeight(), tole\_Field, MapErrorMsg::Warn(), and WARN\_FILE.

Referenced by Landscape().

{

unsigned int linesize = m\_width \* 3;

unsigned char \* frame\_buffer = ( unsigned char \* ) malloc( sizeof( unsigned char ) \* linesize );

if ( frame\_buffer == NULL ) {

g\_msg->Warn( WARN\_FILE, "Landscape::DumpMapGraphics(): Out of memory!", "" );

exit( 1 );

}

FILE \* l\_file = fopen(a\_filename, "w" );

if ( !l\_file ) {

g\_msg->Warn( WARN\_FILE, "Landscape::DumpMapGraphics(): ""Unable to open file for writing: %s\n", a\_filename );

exit( 1 );

}

fprintf( l\_file, "P6\n%d %d %d\n", m\_width, m\_height, 255 );

for ( int y = 0; y < m\_height; y++ ) {

int i = 0;

for ( int x = 0; x < m\_width; x++ ) {

int eletype = ( int )SupplyElementType( x, y );

int localcolor = MapDumpColours[ eletype ];

if ( eletype == ( int )tole\_Field ) {

int category;

double hei = SupplyVegHeight( x, y );

if ( hei > 50.0 ) category = 0; else category = ( int )( 200.0 - ( hei \* 4.0 ) );

localcolor = ( ( category \* 65536 ) + 65535 );

}

frame\_buffer[ i++ ] = ( unsigned char )(localcolor & 0xff);

frame\_buffer[ i++ ] = ( unsigned char )(( localcolor >> 8 ) & 0xff);

frame\_buffer[ i++ ] = ( unsigned char )(( localcolor >> 16 ) & 0xff);

}

fwrite( frame\_buffer, sizeof( unsigned char ), linesize, l\_file );

}

fclose( l\_file );

free( frame\_buffer );

}

|  |  |  |  |
| --- | --- | --- | --- |
| void Landscape::DumpMapInfoByArea | ( | const char \* | *a\_filename*, |
|  |  | bool | *a\_append*, |
|  |  | bool | *a\_dump\_zero\_areas*, |
|  |  | bool | *a\_write\_veg\_names* |
|  | ) |  |  |

References Calendar::DayInYear(), FillVegAreaData(), g\_date, g\_msg, l\_vegtype\_areas, Calendar::OldDays(), tov\_OFirstYearDanger, tov\_Undefined, VegtypeToString(), MapErrorMsg::Warn(), and WARN\_FILE.

Referenced by DumpVegAreaData().

{

FillVegAreaData();

FILE \* outf;

if ( a\_append ) {

outf = fopen(a\_filename, "a" );

if (!outf) {

g\_msg->Warn( WARN\_FILE, "Landscape::DumpMapInfoByArea(): ""Unable to open file for appending", a\_filename );

exit( 1 );

}

} else {

outf = fopen(a\_filename, "w" );

if (!outf) {

g\_msg->Warn( WARN\_FILE, "Landscape::DumpMapInfoByArea(): ""Unable to open file for writing", a\_filename );

exit( 1 );

}

}

// Emit element type info.

for ( unsigned int i = 0; i < tov\_Undefined + 1; i++ ) {

if ( i == tov\_OFirstYearDanger )

continue;

if ( !a\_dump\_zero\_areas && l\_vegtype\_areas[ i ] < 0.5 )

continue;

fprintf( outf, "%6ld\t%3d\t%10.0f", g\_date->OldDays() + g\_date->DayInYear() - 364, i, l\_vegtype\_areas[ i ] );

if ( a\_write\_veg\_names )

fprintf( outf, "\t%s\n", VegtypeToString( ( TTypesOfVegetation )i ) ); else

fprintf( outf, "\n" );

}

fclose( outf );

}

|  |  |  |  |  |  |  |  |  |  |  |  |  |  |
| --- | --- | --- | --- | --- | --- | --- | --- | --- | --- | --- | --- | --- | --- |
| |  |  |  |  | | --- | --- | --- | --- | | void Landscape::DumpPublicSymbols | ( | const char \* | *a\_dumpfile*, | |  |  | CfgSecureLevel | *a\_level* | |  | ) |  |  | | inline |

References Configurator::DumpPublicSymbols(), and g\_cfg.

{

g\_cfg->DumpPublicSymbols( a\_dumpfile, a\_level );

}

|  |  |  |  |  |  |  |  |
| --- | --- | --- | --- | --- | --- | --- | --- |
| |  |  |  |  |  |  | | --- | --- | --- | --- | --- | --- | | void Landscape::DumpTreatCounters | ( | const char \* | *a\_filename* | ) |  | | protected |

References EventtypeToString(), g\_msg, last\_treatment, m\_treatment\_counts, start, MapErrorMsg::Warn(), and WARN\_FILE.

Referenced by ~Landscape().

{

FILE \* l\_file = fopen(a\_filename, "w" );

if ( !l\_file ) {

g\_msg->Warn( WARN\_FILE, "Landscape::DumpTreatCounters(): ""Unable to open file for writing: %s\n", a\_filename );

exit( 1 );

}

for ( int i = start; i < last\_treatment; i++ ) {

fprintf( l\_file, "%3d %s %10d\n", i, EventtypeToString( i ), m\_treatment\_counts[ i ] );

}

fclose( l\_file );

}

|  |  |  |  |  |  |
| --- | --- | --- | --- | --- | --- |
| void Landscape::DumpVegAreaData | ( | int | *a\_day* | ) |  |

References DumpMapInfoByArea(), CfgBool::value(), and CfgStr::value().

Referenced by RunTheSim().

{

if ( cfg\_dumpvegjan.value() ) {

if ( ( a\_day % 365 ) == 0 ) { // Jan 1st

DumpMapInfoByArea( cfg\_dumpvegjanfile.value(), true, true, true );

return;

}

}

if ( cfg\_dumpvegjune.value() ) {

if ( ( a\_day % 365 ) == 152 ) { // 1st June

DumpMapInfoByArea( cfg\_dumpvegjunefile.value(), true, true, true );

}

}

}

|  |  |  |  |  |  |  |  |  |  |  |  |  |  |  |  |  |  |  |  |  |  |
| --- | --- | --- | --- | --- | --- | --- | --- | --- | --- | --- | --- | --- | --- | --- | --- | --- | --- | --- | --- | --- | --- |
| |  |  |  |  | | --- | --- | --- | --- | | void Landscape::EventDump | ( | int | *x*, | |  |  | int | *y*, | |  |  | int | *x2*, | |  |  | int | *y2* | |  | ) |  |  | | protected |

References g\_msg, sleep\_all\_day, SupplyDayInYear(), SupplyLastTreatment(), MapErrorMsg::Warn(), and WARN\_FILE.

Referenced by Tick().

{

FILE \* vfile=fopen("EventDump.txt", "a" );

if (!vfile) {

g\_msg->Warn( WARN\_FILE, "Landscape::EventDump(): Unable to open file", "EventDump.txt" );

exit( 1 );

}

FarmToDo event;

int i = 0;

int day = SupplyDayInYear();

fprintf( vfile, "%d: ", day );

while ( ( event = ( FarmToDo )SupplyLastTreatment( x1, y1, & i ) ) != sleep\_all\_day ) {

fprintf( vfile, "%d ", event );

}

i = 0;

fprintf( vfile, " - " );

while ( ( event = ( FarmToDo )SupplyLastTreatment( x2, y2, & i ) ) != sleep\_all\_day ) {

fprintf( vfile, "%d ", event );

}

fprintf( vfile, "\n" );

fclose( vfile );

}

|  |  |  |  |  |  |
| --- | --- | --- | --- | --- | --- |
| char \* Landscape::EventtypeToString | ( | int | *a\_event* | ) |  |

References autumn\_harrow, autumn\_or\_spring\_plough, autumn\_plough, autumn\_roll, autumn\_sow, burn\_straw\_stubble, cattle\_out, cut\_to\_hay, cut\_to\_silage, cut\_weeds, deep\_ploughing, fa\_ammoniumsulphate, fa\_greenmanure, fa\_manure, fa\_npk, fa\_pk, fa\_sludge, fa\_slurry, flammebehandling, fp\_greenmanure, fp\_liquidNH3, fp\_manganesesulphate, fp\_manure, fp\_npk, fp\_npks, fp\_pk, fp\_sludge, fp\_slurry, fungicide\_treat, g\_msg, glyphosate, growth\_regulator, harvest, hay\_bailing, hay\_turning, herbicide\_treat, hilling\_up, insecticide\_treat, molluscicide, mow, pigs\_out, product\_treat, row\_cultivation, sleep\_all\_day, spring\_harrow, spring\_plough, spring\_roll, spring\_sow, start, straw\_chopping, strigling, strigling\_sow, stubble\_harrowing, swathing, syninsecticide\_treat, trial\_control, trial\_insecticidetreat, trial\_toxiccontrol, MapErrorMsg::Warn(), WARN\_FILE, water, and winter\_plough.

Referenced by DumpTreatCounters(), Skylark\_Clutch::OnFarmEvent(), Skylark\_Nestling::OnFarmEvent(), Skylark\_PreFledgeling::OnFarmEvent(), Skylark\_Female::OnFarmEvent(), and Skylark\_Male::OnFarmEvent().

{

char error\_num[ 20 ];

switch ( a\_event ) {

case start:

return " start";

case sleep\_all\_day:

return " sleep\_all\_day";

case autumn\_plough:

return " autumn\_plough";

case autumn\_harrow:

return " autumn\_harrow";

case autumn\_roll:

return " autumn\_roll";

case autumn\_sow:

return " autumn\_sow";

case winter\_plough:

return " winter\_plough";

case deep\_ploughing:

return " deep\_ploughing";

case spring\_plough:

return " spring\_plough";

case spring\_harrow:

return " spring\_harrow";

case spring\_roll:

return " spring\_roll";

case spring\_sow:

return " spring\_sow";

case fp\_npks:

return " fp\_npks";

case fp\_npk:

return " fp\_npk";

case fp\_pk:

return " fp\_pk";

case fp\_liquidNH3:

return " fp\_liquidNH3";

case fp\_slurry:

return " fp\_slurry";

case fp\_manganesesulphate:

return " fp\_manganesesulphate";

case fp\_manure:

return " fp\_manure";

case fp\_greenmanure:

return " fp\_greenmanure";

case fp\_sludge:

return " fp\_sludge";

case fa\_npk:

return " fa\_npk";

case fa\_pk:

return " fa\_pk";

case fa\_slurry:

return " fa\_slurry";

case fa\_ammoniumsulphate:

return " fa\_ammoniumsulphate";

case fa\_manure:

return " fa\_manure";

case fa\_greenmanure:

return " fa\_greenmanure";

case fa\_sludge:

return " fa\_sludge";

case herbicide\_treat:

return " herbicide\_treat";

case growth\_regulator:

return " growth\_regulator";

case fungicide\_treat:

return " fungicide\_treat";

case insecticide\_treat:

return " insecticide\_treat";

case product\_treat:

return "pesticide\_product\_treat";

case syninsecticide\_treat:

return " syninsecticide\_treat";

case molluscicide:

return " molluscicide";

case row\_cultivation:

return " row\_cultivation";

case strigling:

return " strigling";

case flammebehandling:

return " flammebehandling";

case hilling\_up:

return " hilling\_up";

case water:

return " water";

case swathing:

return " swathing";

case harvest:

return " harvest";

case cattle\_out:

return " cattle\_out";

case pigs\_out:

return " pigs\_out";

case cut\_to\_hay:

return " cut\_to\_hay";

case cut\_to\_silage:

return " cut\_to\_silage";

case straw\_chopping:

return " straw\_chopping";

case hay\_turning:

return " hay\_turning";

case hay\_bailing:

return " hay\_bailing";

case stubble\_harrowing:

return " stubble\_harrowing";

case autumn\_or\_spring\_plough:

return "autumn\_or\_spring\_plough";

case burn\_straw\_stubble:

return " burn\_straw\_stubble";

case mow:

return " mow";

case cut\_weeds:

return " cut\_weeds";

case strigling\_sow:

return " strigling\_sow";

case trial\_insecticidetreat:

return "PesticideTrialTreatment";

case trial\_toxiccontrol:

return " PesticideTrialToxic";

case trial\_control:

return " PesticideTrialControl";

case glyphosate:

return " Glyphosate on setaside";

default:

sprintf( error\_num, "%d", a\_event );

g\_msg->Warn( WARN\_FILE, "Landscape::EventtypeToString(): Unknown event type:", error\_num );

exit( 1 );

}

}

|  |  |  |  |  |
| --- | --- | --- | --- | --- |
| void Landscape::FillVegAreaData | ( |  | ) |  |

References l\_vegtype\_areas, m\_elems, and tov\_Undefined.

Referenced by DumpMapInfoByArea().

{

for ( unsigned int i = 0; i < ( tov\_Undefined + 1 ); i++ ) {

l\_vegtype\_areas[ i ] = 0.0;

}

// Sum up statistics on element type.

for ( unsigned int i = 0; i < m\_elems.size(); i++ ) {

l\_vegtype\_areas[ m\_elems[ i ]->GetVegType() ] += m\_elems[ i ]->GetArea();

}

}

|  |  |  |  |  |  |  |  |  |  |  |  |  |  |  |  |  |  |
| --- | --- | --- | --- | --- | --- | --- | --- | --- | --- | --- | --- | --- | --- | --- | --- | --- | --- |
| |  |  |  |  | | --- | --- | --- | --- | | bool Landscape::FindFieldCenter | ( | LE \* | *a\_field*, | |  |  | int \* | *x*, | |  |  | int \* | *y* | |  | ) |  |  | | protected |

References AxisLoop(), LE::GetPoly(), m\_x\_add, m\_y\_add, and SupplyPolyRef().

Referenced by BeetleBankPossible().

{

// Start at x,y

// works by selecting the point that is a mean of the co-ords of the centers of 4 axes from this point that are in the field.

// Then do it again, and again until we don't move more than 1m or we have tried too many times

int ourpoly=SupplyPolyRef(\*(x),\*(y));

if (ourpoly!=a\_field->GetPoly()) return false;

int centers[2][8];

int tries=0;

int diff=999;

int x1=\*(x);

int y1=\*(y);

int centreX=x1;

int centreY=y1;

// NB we might escape without bounds checking here because the polygon number does not wrap round - will only ever be a problem if we go SimX+1,SimY+1

while ((diff>1) & (tries++<100)) {

for (unsigned v=0; v<4; v++) {

x1=centreX;

y1=centreY;

AxisLoop(ourpoly, &x1, &y1, v);

centers[0][v]=x1-m\_x\_add[v];

centers[1][v]=y1-m\_y\_add[v];

x1=centreX;

y1=centreY;

AxisLoop(ourpoly, &x1, &y1, v+4);

centers[0][v+4]=x1-m\_x\_add[v+4];

centers[1][v+4]=y1-m\_y\_add[v+4];

// centreX+=((centers[0][v]+x1-m\_x\_add[v+4])/2);

// centreY+=((centers[1][v]+y1-m\_y\_add[v+4])/2);

}

int oldx=centreX;

int oldy=centreY;

centreX=0;

centreY=0;

for (int h=0; h<8; h++) {

centreX+=centers[0][h];

centreY+=centers[1][h];

}

centreX/=8;

centreY/=8;

diff=abs(oldx-centreX)+abs(oldy-centreY);

}

\*(x)=centreX;

\*(y)=centreY;

int tourpoly=SupplyPolyRef(\*(x),\*(y));

if (tourpoly!=ourpoly) {

return false; // can happen eg if there is a pond in the middle of the field

}

return true;

}

|  |  |  |  |  |  |  |  |  |  |  |  |  |  |  |  |  |  |
| --- | --- | --- | --- | --- | --- | --- | --- | --- | --- | --- | --- | --- | --- | --- | --- | --- | --- |
| |  |  |  |  | | --- | --- | --- | --- | | int Landscape::FindLongestAxis | ( | int \* | *x*, | |  |  | int \* | *y*, | |  |  | int \* | *a\_length* | |  | ) |  |  | | protected |

References AxisLoop(), m\_x\_add, m\_y\_add, and SupplyPolyRef().

Referenced by BeetleBankPossible().

{

int ourpoly=SupplyPolyRef(\*(a\_x),\*(a\_y));

int dist=0;

int found=-1;

int dx=0;

int dy=0;

int fx=0;

int fy=0;

for (unsigned v=0; v<4; v++) {

int x1=\*(a\_x);

int y1=\*(a\_y);

AxisLoop(ourpoly, &x1, &y1, v);

dx=abs(\*(a\_x)-(x1-m\_x\_add[v]));

dy=abs(\*(a\_y)-(y1-m\_y\_add[v]));

if (dx>dist) {

fx=x1;

fy=y1;

found=v;

dist=dx;

} else

if (dy>dist) {

fx=x1;

fy=y1;

found=v;

dist=dy;

}

}

int x2=\*(a\_x);

int y2=\*(a\_y);

AxisLoop(ourpoly, &x2, &y2, found+4);

// get the centre of the this axis

\*(a\_x)=(fx+x2)/2;

\*(a\_y)=(fy+y2)/2;

// and the length, only S does not change x

if (found!=2) \*(a\_length)=abs(fx-x2)/2;

else \*(a\_length)=abs(fy-y2)/2;

return found;

}

|  |  |  |  |  |  |  |  |  |  |  |  |  |  |  |  |  |  |
| --- | --- | --- | --- | --- | --- | --- | --- | --- | --- | --- | --- | --- | --- | --- | --- | --- | --- |
| |  |  |  |  | | --- | --- | --- | --- | | bool Landscape::FindValidXY | ( | int | *a\_field*, | |  |  | int & | *a\_x*, | |  |  | int & | *a\_y* | |  | ) |  |  | | protected |

References RasterMap::Get(), m\_land, RasterMap::MapHeight(), and RasterMap::MapWidth().

Referenced by UnsprayedMarginScan().

{

// From a hopefully sensible starting point this method scans in the

// 8 directions to find a good valid x and y matching a\_field

int x\_add[ 8 ] = { 1, 1, 0, -1, -1, -1, 0, 1 };

int y\_add[ 8 ] = { 0, -1, -1, -1, 0, 1, 1, 1 };

int index;

int nx, ny;

int width = m\_land->MapWidth();

int height = m\_land->MapHeight();

// Assume it has to within 100m

for ( int i = 0; i < 100; i++ ) {

for ( int l = 0; l < 8; l++ ) {

nx = a\_x + x\_add[ l ] \* i;

ny = a\_y + y\_add[ l ] \* i;

if ( ( nx < width ) && ( nx >= 0 ) && ( ny < height ) && ( ny >= 0 ) ) {

index = m\_land->Get( nx, ny );

if ( index == a\_field ) {

a\_x = a\_x + x\_add[ l ] \* i;

a\_y = a\_y + y\_add[ l ] \* i;

return true;

}

}

}

}

return false;

}

|  |  |  |  |  |  |  |  |
| --- | --- | --- | --- | --- | --- | --- | --- |
| |  |  |  |  |  |  | | --- | --- | --- | --- | --- | --- | | void Landscape::ForceArea | ( | void |  | ) |  | | protected |

References g\_msg, m\_elems, m\_height, m\_width, MapErrorMsg::Warn(), and WARN\_BUG.

Referenced by Landscape().

{

int l\_area\_sum = 0;

for ( unsigned int i = 0; i < m\_elems.size(); i++ ) {

m\_elems[ i ]->SetArea( ( double )m\_elems[ i ]->m\_squares\_in\_map );

if ( m\_elems[ i ]->m\_squares\_in\_map > 0 ) {

m\_elems[ i ]->SetMapValid( true );

l\_area\_sum += m\_elems[ i ]->m\_squares\_in\_map;

}

}

if ( l\_area\_sum != m\_width \* m\_height ) {

g\_msg->Warn( WARN\_BUG, "Landscape::ForceArea(): Polygon areas doesn't"" sum up to map area!", "" );

exit( 1 );

}

}

|  |  |  |  |  |  |  |  |
| --- | --- | --- | --- | --- | --- | --- | --- |
| |  |  |  |  |  |  | | --- | --- | --- | --- | --- | --- | | Point Landscape::GetNextSeed | ( | int |  | ) |  | | protected |

|  |  |  |  |  |  |  |  |
| --- | --- | --- | --- | --- | --- | --- | --- |
| |  |  |  |  |  |  | | --- | --- | --- | --- | --- | --- | | double Landscape::GetVegArea | ( | int | *v* | ) |  | | inline |

References l\_vegtype\_areas.

Referenced by Skylark\_Population\_Manager::FledgelingProbeOutput().

{ return l\_vegtype\_areas[v]; }

|  |  |  |  |  |  |  |  |
| --- | --- | --- | --- | --- | --- | --- | --- |
| |  |  |  |  |  |  | | --- | --- | --- | --- | --- | --- | | void Landscape::hb\_Add | ( | void |  | ) |  | | protected |

References RasterMap::GetMagicP(), hb\_AddNewHedgebanks(), hb\_border\_pixels, hb\_ClearPolygon(), hb\_core\_pixels, hb\_DownPolyNumbers(), hb\_FindBoundingBox(), hb\_FindHedges(), hb\_hedges, hb\_height, HB\_MAGIC, hb\_map, hb\_MarkTheBresenhamWay(), hb\_MarkTopFromLocalMax(), hb\_PaintBorder(), hb\_PaintWhoHasNeighbourColor(), hb\_ResetColorBits(), hb\_RestoreHedgeCore(), hb\_size, hb\_UpPolyNumbers(), hb\_width, l\_map\_art\_hb\_core\_thres, l\_map\_art\_hb\_width, m\_elems, m\_land, m\_polymapping, RasterMap::MapHeight(), RasterMap::MapWidth(), PolytypeToString(), tole\_FieldBoundary, and CfgFloat::value().

Referenced by Landscape().

{

// Make a local copy of the actual map parameters for speed

// purposes. We are going to use these \*a lot\*.

hb\_map = m\_land->GetMagicP( 0, 0 );

hb\_width = m\_land->MapWidth();

hb\_height = m\_land->MapHeight();

hb\_size = hb\_width \* hb\_height;

//#ifdef HB\_TESTING

//m\_elems[m\_polymapping

//[hb\_map[750 + 600\*hb\_width]]]->SetElementType( tole\_Hedges );

//#endif

// Generate a list of polygon numbers + HB\_MAGIC for all hedge elements

// in our current map.

hb\_FindHedges();

// As one lemming at the cliff edge said to the other: "Let's Go!"

// For every hedge polygon:

printf("Hedges: %d\n", hb\_hedges.size());

fflush(stdout);

double l\_inc = 50.0/((double)hb\_hedges.size());

double l\_count = 0.001;

for ( unsigned int i=0; i<hb\_hedges.size(); i++ ) {

l\_count += l\_inc;

if ( l\_count > 1.0 ) {

l\_count = 0.001;

printf(".");

fflush(stdout);

}

// Find the enclosing bounding box for our current polygon.

if ( ! hb\_FindBoundingBox( hb\_hedges[i] )) {

// Might want to raise a runtime error upon ending here.

// The test for hb\_FindBoundingBox() fails if we have a polygon

// without any pixels in the main map!

continue;

}

// Move all polygon numbers in our bounding box up! by HB\_MAGIC

// to make room for our 'paint' values in the space below.

hb\_UpPolyNumbers();

hb\_ClearPolygon( hb\_hedges[i] + HB\_MAGIC );

hb\_PaintBorder( 0 );

int l\_color = 0;

while ( hb\_PaintWhoHasNeighbourColor( l\_color,

l\_color + 1 )) {

l\_color++;

}

double l\_percent = (double)hb\_core\_pixels/

((double)hb\_core\_pixels+(double)hb\_border\_pixels);

if ( l\_percent > l\_map\_art\_hb\_core\_thres.value()) {

// We ought to have at least \*some\* visible core area

// within the polygon, so do a simple scan.

// l\_color now happens to contain the maximum contour/color value

// within our current polygon.

int l\_min = (int)((double)l\_color \* l\_map\_art\_hb\_width.value());

while ( l\_color > l\_min ) {

hb\_MarkTopFromLocalMax( l\_color );

hb\_ResetColorBits();

l\_color--;

}

} else {

// Very thin hedge (1->2 meters),

// use special border case scan when adding hedge banks.

hb\_MarkTheBresenhamWay();

}

hb\_RestoreHedgeCore( hb\_hedges[i] + HB\_MAGIC );

// All the previous steps were just preparations.

// Now do the serious stuff.

hb\_AddNewHedgebanks( hb\_hedges[i] );

#ifdef HB\_TESTING

// Sanity check.

for ( int j=0; j<hb\_size; j++ ) {

if ( m\_polymapping[ hb\_map[ j ] - HB\_MAGIC ] == -1 ) {

printf("Oh dear!\n");

exit(1);

}

}

char ffname[24];

sprintf( ffname, "test%02d.ppm", i);

#endif

// Restore order to the map before continuing.

hb\_DownPolyNumbers();

#ifdef HB\_TESTING

hb\_dump\_map( 0, hb\_width, 0, hb\_height, ffname, false );

#endif

}

printf("\n");

#ifdef HB\_TESTING

// Quick reminder.

for ( unsigned int i=0; i<m\_elems.size(); i++ ) {

if ( m\_elems[i]->GetArea() < 0.9 &&

m\_elems[i]->GetElementType() != tole\_FieldBoundary ) {

// Note: Field boundaries have not been scanned yet at this point,

// so they will have a zero area right here and now.

printf("Warning: Polygon %6d of type %s came up with area %3.2f!\n",

m\_elems[i]->GetPoly(),

PolytypeToString( m\_elems[i]->GetElementType() ),

m\_elems[i]->GetArea() );

}

}

exit(0);

#endif

}

|  |  |  |  |  |  |  |  |
| --- | --- | --- | --- | --- | --- | --- | --- |
| |  |  |  |  |  |  | | --- | --- | --- | --- | --- | --- | | void Landscape::hb\_AddNewHedgebanks | ( | int | *a\_orig\_poly\_num* | ) |  | | protected |

References hb\_GenerateHBPolys(), HB\_MAGIC, hb\_map, hb\_max\_x, hb\_max\_y, hb\_min\_x, hb\_min\_y, hb\_StripingDist(), and hb\_width.

Referenced by hb\_Add().

{

hb\_GenerateHBPolys();

for ( int l\_y=hb\_min\_y; l\_y<=hb\_max\_y; l\_y++ ) {

for ( int l\_x=hb\_min\_x; l\_x<=hb\_max\_x; l\_x++ ) {

int i = l\_y\*hb\_width + l\_x;

int l\_val = hb\_map[ i ];

if ( l\_val >= 0 && l\_val < HB\_MAGIC ) {

hb\_map[ i ] = hb\_StripingDist() + HB\_MAGIC;

/\*

m\_elems[m\_polymapping[hb\_map[ i ]-HB\_MAGIC]]->AddArea( 1.0 );

m\_elems[m\_polymapping[hb\_map[ i ]-HB\_MAGIC]]->m\_squares\_in\_map++;

m\_elems[m\_polymapping[a\_orig\_poly\_num]]->AddArea( -1.0 );

if ( m\_elems[m\_polymapping[a\_orig\_poly\_num]]->GetArea() < 0.0 ) {

printf("%d %d !!!\n", l\_x, l\_y );

exit(1);

}

\*/

//m\_elems[m\_polymapping[a\_orig\_poly\_num]]->m\_squares\_in\_map--;

}

}

}

}

|  |  |  |  |  |  |  |  |
| --- | --- | --- | --- | --- | --- | --- | --- |
| |  |  |  |  |  |  | | --- | --- | --- | --- | --- | --- | | void Landscape::hb\_Cleanup | ( | void |  | ) |  | | protected |

|  |  |  |  |  |  |  |  |
| --- | --- | --- | --- | --- | --- | --- | --- |
| |  |  |  |  |  |  | | --- | --- | --- | --- | --- | --- | | void Landscape::hb\_ClearPolygon | ( | int | *a\_poly\_num* | ) |  | | protected |

References HB\_MAGIC\_COLOR, hb\_map, hb\_max\_x, hb\_max\_y, hb\_min\_x, hb\_min\_y, and hb\_width.

Referenced by hb\_Add().

{

// 'Paint' every pixel which belongs to the current polygon with the

// value HB\_MAGIC\_COLOR.

for ( int l\_y=hb\_min\_y; l\_y<=hb\_max\_y; l\_y++ ) {

for ( int l\_x=hb\_min\_x; l\_x<=hb\_max\_x; l\_x++ ) {

int l\_coord = l\_y\*hb\_width + l\_x;

if ( hb\_map[ l\_coord ] == a\_poly\_num ) {

hb\_map[ l\_coord ] = HB\_MAGIC\_COLOR;

}

}

}

}

|  |  |  |  |  |  |  |  |
| --- | --- | --- | --- | --- | --- | --- | --- |
| |  |  |  |  |  |  | | --- | --- | --- | --- | --- | --- | | void Landscape::hb\_DownPolyNumbers | ( | void |  | ) |  | | protected |

References HB\_MAGIC, hb\_map, and hb\_size.

Referenced by hb\_Add().

{

for ( int i=0; i<hb\_size; i++ ) {

if ( hb\_map[ i ] >= HB\_MAGIC ) {

hb\_map[ i ] -= HB\_MAGIC;

}

}

}

|  |  |  |  |  |  |  |  |
| --- | --- | --- | --- | --- | --- | --- | --- |
| |  |  |  |  |  |  | | --- | --- | --- | --- | --- | --- | | bool Landscape::hb\_FindBoundingBox | ( | int | *a\_poly\_num* | ) |  | | protected |

References hb\_height, hb\_map, hb\_max\_x, hb\_max\_y, hb\_min\_x, hb\_min\_y, and hb\_width.

Referenced by hb\_Add().

{

hb\_min\_x = hb\_width + 1;

hb\_max\_x = -1;

hb\_min\_y = hb\_height + 1;

hb\_max\_y = -1;

bool l\_found = false;

// Search for every pixel which belongs to the current polygon, and

// min and max values for x and y. Values are inclusive.

for ( int l\_y=0; l\_y<hb\_height; l\_y++ ) {

for ( int l\_x=0; l\_x<hb\_width; l\_x++ ) {

if ( hb\_map[ l\_y\*hb\_width + l\_x ] == a\_poly\_num ) {

l\_found = true;

if ( l\_x < hb\_min\_x)

hb\_min\_x = l\_x;

else if ( l\_x > hb\_max\_x)

hb\_max\_x = l\_x;

if ( l\_y < hb\_min\_y)

hb\_min\_y = l\_y;

else if ( l\_y > hb\_max\_y)

hb\_max\_y = l\_y;

}

}

}

return l\_found;

}

|  |  |  |  |  |  |  |  |
| --- | --- | --- | --- | --- | --- | --- | --- |
| |  |  |  |  |  |  | | --- | --- | --- | --- | --- | --- | | void Landscape::hb\_FindHedges | ( | void |  | ) |  | | protected |

References hb\_hedges, m\_elems, and tole\_Hedges.

Referenced by hb\_Add().

{

for ( unsigned int i=0; i<m\_elems.size(); i++ ) {

if ( tole\_Hedges == m\_elems[i]->GetElementType() ) {

hb\_hedges.resize( hb\_hedges.size() + 1 );

hb\_hedges[ hb\_hedges.size() - 1 ] =

m\_elems[i]->GetPoly();

}

}

}

|  |  |  |  |  |  |  |  |
| --- | --- | --- | --- | --- | --- | --- | --- |
| |  |  |  |  |  |  | | --- | --- | --- | --- | --- | --- | | void Landscape::hb\_GenerateHBPolys | ( | void |  | ) |  | | protected |

References hb\_first\_free\_poly\_num, hb\_new\_hbs, l\_map\_art\_hb\_nums, m\_elems, m\_polymapping, NewElement(), tole\_HedgeBank, and CfgInt::value().

Referenced by hb\_AddNewHedgebanks().

{

hb\_new\_hbs.resize( l\_map\_art\_hb\_nums.value() );

for ( int i =0; i<l\_map\_art\_hb\_nums.value(); i++ ) {

hb\_new\_hbs[ i ] = NewElement( tole\_HedgeBank );

hb\_new\_hbs[ i ]->SetArea( 0.0 );

hb\_new\_hbs[ i ]->SetSubType( i );

m\_elems.resize( m\_elems.size() + 1 );

m\_elems[ m\_elems.size() - 1 ] = hb\_new\_hbs[ i ];

m\_polymapping[ hb\_first\_free\_poly\_num ] =

(int) m\_elems.size() - 1;

hb\_new\_hbs[ i ]->SetPoly( hb\_first\_free\_poly\_num++ );

}

}

|  |  |  |  |  |  |  |  |  |  |  |  |  |  |  |  |  |  |
| --- | --- | --- | --- | --- | --- | --- | --- | --- | --- | --- | --- | --- | --- | --- | --- | --- | --- |
| |  |  |  |  | | --- | --- | --- | --- | | bool Landscape::hb\_HasNeighbourColor | ( | int | *a\_x*, | |  |  | int | *a\_y*, | |  |  | int | *a\_neighbour\_color* | |  | ) |  |  | | inlineprotected |

References hb\_height, hb\_map, and hb\_width.

Referenced by hb\_PaintWhoHasNeighbourColor().

{

bool l\_neighbour\_has\_color = false;

bool loop=false;

do {

a\_x -= 1;

a\_y -= 1;

if ( a\_x >= 0 && a\_y >= 0 &&

hb\_map[ a\_x + a\_y\*hb\_width ] == a\_neighbour\_color ) {

l\_neighbour\_has\_color = true;

break;

}

a\_x++;

if ( hb\_map[ a\_x + a\_y\*hb\_width ] == a\_neighbour\_color ) {

l\_neighbour\_has\_color = true;

break;

}

a\_x++;

if ( a\_x < hb\_width &&

hb\_map[ a\_x + a\_y\*hb\_width ] == a\_neighbour\_color ) {

l\_neighbour\_has\_color = true;

break;

}

a\_x -= 2;

a\_y += 1;

if ( a\_x >= 0 &&

hb\_map[ a\_x + a\_y\*hb\_width ] == a\_neighbour\_color ) {

l\_neighbour\_has\_color = true;

break;

}

a\_x += 2;

if ( a\_x < hb\_width &&

hb\_map[ a\_x + a\_y\*hb\_width ] == a\_neighbour\_color ) {

l\_neighbour\_has\_color = true;

break;

}

a\_x -= 2;

a\_y += 1;

if ( a\_y >= hb\_height )

break;

if ( a\_x >= 0 &&

hb\_map[ a\_x + a\_y\*hb\_width ] == a\_neighbour\_color ) {

l\_neighbour\_has\_color = true;

break;

}

a\_x++;

if ( hb\_map[ a\_x + a\_y\*hb\_width ] == a\_neighbour\_color ) {

l\_neighbour\_has\_color = true;

break;

}

a\_x++;

if ( a\_x < hb\_width &&

hb\_map[ a\_x + a\_y\*hb\_width ] == a\_neighbour\_color ) {

l\_neighbour\_has\_color = true;

break;

}

} while ( loop ); // was while (false) CJT 16-05-06

return l\_neighbour\_has\_color;

}

|  |  |  |  |  |  |  |  |  |  |  |  |  |  |
| --- | --- | --- | --- | --- | --- | --- | --- | --- | --- | --- | --- | --- | --- |
| |  |  |  |  | | --- | --- | --- | --- | | bool Landscape::hb\_HasOtherNeighbour | ( | int | *a\_x*, | |  |  | int | *a\_y* | |  | ) |  |  | | inlineprotected |

References HB\_MAGIC, hb\_map, hb\_MapBorder(), and hb\_width.

Referenced by hb\_PaintBorder().

{

bool l\_has\_neighbour = false;

bool loop=false;

// Who said C++ needs the goto statement? ;-)

do {

a\_x -= 1;

a\_y -= 1;

if ( hb\_MapBorder( a\_x, a\_y ) ||

hb\_map[ a\_x + a\_y\*hb\_width ] >= HB\_MAGIC ) {

l\_has\_neighbour = true;

break;

}

a\_x++;

if ( hb\_MapBorder( a\_x, a\_y ) ||

hb\_map[ a\_x + a\_y\*hb\_width ] >= HB\_MAGIC ) {

l\_has\_neighbour = true;

break;

}

a\_x++;

if ( hb\_MapBorder( a\_x, a\_y ) ||

hb\_map[ a\_x + a\_y\*hb\_width ] >= HB\_MAGIC ) {

l\_has\_neighbour = true;

break;

}

a\_x -= 2;

a\_y += 1;

if ( hb\_MapBorder( a\_x, a\_y ) ||

hb\_map[ a\_x + a\_y\*hb\_width ] >= HB\_MAGIC ) {

l\_has\_neighbour = true;

break;

}

a\_x += 2;

if ( hb\_MapBorder( a\_x, a\_y ) ||

hb\_map[ a\_x + a\_y\*hb\_width ] >= HB\_MAGIC ) {

l\_has\_neighbour = true;

break;

}

a\_x -= 2;

a\_y += 1;

if ( hb\_MapBorder( a\_x, a\_y ) ||

hb\_map[ a\_x + a\_y\*hb\_width ] >= HB\_MAGIC ) {

l\_has\_neighbour = true;

break;

}

a\_x++;

if ( hb\_MapBorder( a\_x, a\_y ) ||

hb\_map[ a\_x + a\_y\*hb\_width ] >= HB\_MAGIC ) {

l\_has\_neighbour = true;

break;

}

a\_x++;

if ( hb\_MapBorder( a\_x, a\_y ) ||

hb\_map[ a\_x + a\_y\*hb\_width ] >= HB\_MAGIC ) {

l\_has\_neighbour = true;

break;

}

} while ( loop ); // was while (false) CJT 16-05-06

return l\_has\_neighbour;

}

|  |  |  |  |  |  |  |  |  |  |  |  |  |  |
| --- | --- | --- | --- | --- | --- | --- | --- | --- | --- | --- | --- | --- | --- |
| |  |  |  |  | | --- | --- | --- | --- | | bool Landscape::hb\_MapBorder | ( | int | *a\_x*, | |  |  | int | *a\_y* | |  | ) |  |  | | inlineprotected |

References hb\_height, and hb\_width.

Referenced by hb\_HasOtherNeighbour().

{

if ( a\_x < 0 ||

a\_y < 0 ||

a\_x >= hb\_width ||

a\_y >= hb\_height ) {

return true;

}

return false;

}

|  |  |  |  |  |  |  |  |
| --- | --- | --- | --- | --- | --- | --- | --- |
| |  |  |  |  |  |  | | --- | --- | --- | --- | --- | --- | | void Landscape::hb\_MarkTheBresenhamWay | ( | void |  | ) |  | | protected |

References HB\_MAGIC, hb\_map, hb\_max\_x, hb\_max\_y, hb\_min\_x, hb\_min\_y, hb\_width, l\_map\_art\_hb\_seg\_len, l\_map\_art\_hb\_width, random(), and CfgFloat::value().

Referenced by hb\_Add().

{

double l\_hb = l\_map\_art\_hb\_width.value()\*10000.0;

double l\_up = 10000.0 - l\_hb;

bool l\_is\_hb = true;

double l\_length = (double)(random( (int)l\_hb )) \* 0.0001 \*

l\_map\_art\_hb\_seg\_len.value();

double l\_hb\_remain = 0.0;

double l\_up\_remain = 0.0;

for ( int l\_y=hb\_min\_y; l\_y<=hb\_max\_y; l\_y++) {

// Note: The missing update of l\_x below is intentional.

for ( int l\_x=hb\_min\_x; l\_x<=hb\_max\_x; ) {

int l\_coord = l\_y\*hb\_width + l\_x;

if ( hb\_map[ l\_coord ] < HB\_MAGIC ) {

// 'Painting' with a negative value turns a pixel into

// hedgebank 'core', ie. hedge!

// Paint segment length with appropriate type.

if ( l\_length >= 1.0 ) {

if ( l\_is\_hb ) {

hb\_map[ l\_coord ] = 0;

} else {

hb\_map[ l\_coord ] = -1;

}

l\_length -= 1.0;

l\_x++;

} else {

// Change state.

if ( l\_is\_hb ) {

l\_is\_hb = false;

l\_hb\_remain = l\_length;

l\_length = (double)(random( (int)l\_up )) \* 0.0001 \*

l\_map\_art\_hb\_seg\_len.value() + l\_up\_remain;

} else {

l\_is\_hb = true;

l\_up\_remain = l\_length;

l\_length = (double)(random( (int)l\_hb )) \* 0.0001 \*

l\_map\_art\_hb\_seg\_len.value() + l\_hb\_remain;

}

}

} else {

// Pixel not part of the hedge, just update the coordinate.

l\_x++;

}

}

}

}

|  |  |  |  |  |  |  |  |
| --- | --- | --- | --- | --- | --- | --- | --- |
| |  |  |  |  |  |  | | --- | --- | --- | --- | --- | --- | | void Landscape::hb\_MarkTopFromLocalMax | ( | int | *a\_color* | ) |  | | protected |

References HB\_MAGIC\_PAINTER\_BIT, hb\_map, hb\_max\_x, hb\_max\_y, hb\_MaxUnpaintedNegNeighbour(), hb\_min\_x, hb\_min\_y, and hb\_width.

Referenced by hb\_Add().

{

// Sweep through the map. If we find a pixel, which has a value

// equal to a\_color and \*not\* a negative neighbour, then mark it as

// negative with an absolute value equal to the number of steps from

// here and out to the center/perimeter polygons border, calculated

// as the given percentage.

// If one or more negative numbers are found among the neighbours,

// some that were not marked during this round, then assign a

// negative value equal to the minimum found plus one.

for ( int l\_y=hb\_min\_y; l\_y<=hb\_max\_y; l\_y++ ) {

for ( int l\_x=hb\_min\_x; l\_x<=hb\_max\_x; l\_x++ ) {

int l\_coord = l\_y\*hb\_width + l\_x;

int l\_max;

if ( hb\_map[ l\_coord ] == a\_color ) {

l\_max = hb\_MaxUnpaintedNegNeighbour( l\_x, l\_y );

if ( l\_max < 0 ) {

if ( ++l\_max < 0 ) {

hb\_map[ l\_coord ] = l\_max;

} else {

// l\_max was precisely -1

hb\_map[ l\_coord ] = -1;

}

} else {

// We are a local maxima.

hb\_map[ l\_coord ] = -a\_color;

}

hb\_map[ l\_coord ] ^= HB\_MAGIC\_PAINTER\_BIT;

}

}

}

}

|  |  |  |  |  |  |  |  |  |  |  |  |  |  |
| --- | --- | --- | --- | --- | --- | --- | --- | --- | --- | --- | --- | --- | --- |
| |  |  |  |  | | --- | --- | --- | --- | | int Landscape::hb\_MaxUnpaintedNegNeighbour | ( | int | *a\_x*, | |  |  | int | *a\_y* | |  | ) |  |  | | inlineprotected |

References hb\_height, HB\_MAGIC\_PAINTER\_BIT, hb\_map, and hb\_width.

Referenced by hb\_MarkTopFromLocalMax().

{

int l\_max\_neg = 0;

int l\_val;

bool loop=false; // This is just to avoid the warning about constant expression if we use: do {...} while (false)

do {

a\_x -= 1;

a\_y -= 1;

if ( a\_x >= 0 && a\_y >= 0 ) {

l\_val = hb\_map[ a\_x + a\_y\*hb\_width ];

if ( l\_val < l\_max\_neg && (l\_val & HB\_MAGIC\_PAINTER\_BIT)) {

l\_max\_neg = l\_val;

}

}

a\_x++;

l\_val = hb\_map[ a\_x + a\_y\*hb\_width ];

if ( l\_val < l\_max\_neg && (l\_val & HB\_MAGIC\_PAINTER\_BIT)) {

l\_max\_neg = l\_val;

}

a\_x++;

if ( a\_x < hb\_width ) {

l\_val = hb\_map[ a\_x + a\_y\*hb\_width ];

if ( l\_val < l\_max\_neg && (l\_val & HB\_MAGIC\_PAINTER\_BIT)) {

l\_max\_neg = l\_val;

}

}

a\_x -= 2;

a\_y += 1;

if ( a\_x >= 0 ) {

l\_val = hb\_map[ a\_x + a\_y\*hb\_width ];

if ( l\_val < l\_max\_neg && (l\_val & HB\_MAGIC\_PAINTER\_BIT)) {

l\_max\_neg = l\_val;

}

}

a\_x += 2;

if ( a\_x < hb\_width ) {

l\_val = hb\_map[ a\_x + a\_y\*hb\_width ];

if ( l\_val < l\_max\_neg && (l\_val & HB\_MAGIC\_PAINTER\_BIT)) {

l\_max\_neg = l\_val;

}

}

a\_x -= 2;

a\_y += 1;

if ( a\_y >= hb\_height )

break;

if ( a\_x >= 0 ) {

l\_val = hb\_map[ a\_x + a\_y\*hb\_width ];

if ( l\_val < l\_max\_neg && (l\_val & HB\_MAGIC\_PAINTER\_BIT)) {

l\_max\_neg = l\_val;

}

}

a\_x++;

l\_val = hb\_map[ a\_x + a\_y\*hb\_width ];

if ( l\_val < l\_max\_neg && (l\_val & HB\_MAGIC\_PAINTER\_BIT)) {

l\_max\_neg = l\_val;

}

a\_x++;

if ( a\_x < hb\_width ) {

l\_val = hb\_map[ a\_x + a\_y\*hb\_width ];

if ( l\_val < l\_max\_neg && (l\_val & HB\_MAGIC\_PAINTER\_BIT)) {

l\_max\_neg = l\_val;

}

}

} while ( loop );

return l\_max\_neg;

}

|  |  |  |  |  |  |  |  |
| --- | --- | --- | --- | --- | --- | --- | --- |
| |  |  |  |  |  |  | | --- | --- | --- | --- | --- | --- | | void Landscape::hb\_PaintBorder | ( | int | *a\_color* | ) |  | | protected |

References hb\_border\_pixels, hb\_core\_pixels, hb\_HasOtherNeighbour(), HB\_MAGIC\_COLOR, hb\_map, hb\_max\_x, hb\_max\_y, hb\_min\_x, hb\_min\_y, and hb\_width.

Referenced by hb\_Add().

{

hb\_border\_pixels = 0;

hb\_core\_pixels = 0;

// Paint every pixel which belongs to the current polygon, and which

// does have a neighbour \*not\* equal to HB\_MAGIC\_COLOR (our marker value).

for ( int l\_y=hb\_min\_y; l\_y<=hb\_max\_y; l\_y++ ) {

for ( int l\_x=hb\_min\_x; l\_x<=hb\_max\_x; l\_x++ ) {

int l\_coord = l\_y\*hb\_width + l\_x;

if ( hb\_map[ l\_coord ] == HB\_MAGIC\_COLOR ) {

if ( hb\_HasOtherNeighbour( l\_x, l\_y )) {

hb\_map[ l\_coord ] = a\_color;

hb\_border\_pixels++;

} else {

hb\_core\_pixels++;

}

}

}

}

}

|  |  |  |  |  |  |  |  |  |  |  |  |  |  |
| --- | --- | --- | --- | --- | --- | --- | --- | --- | --- | --- | --- | --- | --- |
| |  |  |  |  | | --- | --- | --- | --- | | bool Landscape::hb\_PaintWhoHasNeighbourColor | ( | int | *a\_neighbour\_color*, | |  |  | int | *a\_new\_color* | |  | ) |  |  | | protected |

References hb\_HasNeighbourColor(), HB\_MAGIC\_COLOR, hb\_map, hb\_max\_x, hb\_max\_y, hb\_min\_x, hb\_min\_y, and hb\_width.

Referenced by hb\_Add().

{

// Paint every pixel which belongs to the current polygon, and which

// has a neighbour with a color equal to a\_neighbour\_color.

bool l\_at\_least\_one = false;

for ( int l\_y=hb\_min\_y; l\_y<=hb\_max\_y; l\_y++ ) {

for ( int l\_x=hb\_min\_x; l\_x<=hb\_max\_x; l\_x++ ) {

if ( hb\_map[l\_y\*hb\_width + l\_x] == HB\_MAGIC\_COLOR &&

hb\_HasNeighbourColor( l\_x, l\_y, a\_neighbour\_color )) {

hb\_map[l\_y\*hb\_width + l\_x] = a\_new\_color;

l\_at\_least\_one = true;

}

}

}

return l\_at\_least\_one;

}

|  |  |  |  |  |  |  |  |
| --- | --- | --- | --- | --- | --- | --- | --- |
| |  |  |  |  |  |  | | --- | --- | --- | --- | --- | --- | | void Landscape::hb\_ResetColorBits | ( | void |  | ) |  | | protected |

References HB\_MAGIC\_PAINTER\_BIT, hb\_map, hb\_max\_x, hb\_max\_y, hb\_min\_x, hb\_min\_y, and hb\_width.

Referenced by hb\_Add().

{

for ( int l\_y=hb\_min\_y; l\_y<=hb\_max\_y; l\_y++ ) {

for ( int l\_x=hb\_min\_x; l\_x<=hb\_max\_x; l\_x++ ) {

int l\_coord = l\_y\*hb\_width + l\_x;

if ( hb\_map[l\_coord] < 0 )

hb\_map[l\_coord] |= HB\_MAGIC\_PAINTER\_BIT;

}

}

}

|  |  |  |  |  |  |  |  |
| --- | --- | --- | --- | --- | --- | --- | --- |
| |  |  |  |  |  |  | | --- | --- | --- | --- | --- | --- | | void Landscape::hb\_RestoreHedgeCore | ( | int | *a\_orig\_poly\_number* | ) |  | | protected |

References hb\_map, hb\_max\_x, hb\_max\_y, hb\_min\_x, hb\_min\_y, and hb\_width.

Referenced by hb\_Add().

{

for ( int l\_y=hb\_min\_y; l\_y<=hb\_max\_y; l\_y++ ) {

for ( int l\_x=hb\_min\_x; l\_x<=hb\_max\_x; l\_x++ ) {

int l\_coord = l\_y\*hb\_width + l\_x;

if ( hb\_map[l\_coord] < 0 ) {

hb\_map[l\_coord] = a\_orig\_poly\_number;

}

}

}

}

|  |  |  |  |  |  |  |  |
| --- | --- | --- | --- | --- | --- | --- | --- |
| |  |  |  |  |  |  | | --- | --- | --- | --- | --- | --- | | int Landscape::hb\_StripingDist | ( | void |  | ) |  | | protected |

References LE::GetPoly(), hb\_new\_hbs, l\_map\_art\_hb\_nums, l\_map\_art\_hb\_tran\_prob, random(), and CfgInt::value().

Referenced by hb\_AddNewHedgebanks().

{

// Striping distribution function for the new hedgebanks.

static LE\* l\_curr\_ele = NULL;

if ( !l\_curr\_ele ) {

l\_curr\_ele = hb\_new\_hbs[ random( l\_map\_art\_hb\_nums.value()) ];

return l\_curr\_ele->GetPoly();

}

if ( random(100) < l\_map\_art\_hb\_tran\_prob.value() )

l\_curr\_ele = hb\_new\_hbs[ random( l\_map\_art\_hb\_nums.value()) ];

return l\_curr\_ele->GetPoly();

}

|  |  |  |  |  |  |  |  |
| --- | --- | --- | --- | --- | --- | --- | --- |
| |  |  |  |  |  |  | | --- | --- | --- | --- | --- | --- | | void Landscape::hb\_UpPolyNumbers | ( | void |  | ) |  | | protected |

References HB\_MAGIC, hb\_map, and hb\_size.

Referenced by hb\_Add().

{

for ( int i=0; i<hb\_size; i++ ) {

hb\_map[ i ] += HB\_MAGIC;

}

}

|  |  |  |  |  |  |
| --- | --- | --- | --- | --- | --- |
| void Landscape::IncTreatCounter | ( | int | *a\_treat* | ) |  |

References g\_msg, last\_treatment, m\_treatment\_counts, MapErrorMsg::Warn(), and WARN\_BUG.

{

if ( a\_treat < 0 || a\_treat >= last\_treatment ) {

char errornum[ 20 ];

sprintf( errornum, "%d", a\_treat );

g\_msg->Warn( WARN\_BUG, "Landscape::IncTreatCounter(): Index"" out of range!", errornum );

exit( 1 );

}

m\_treatment\_counts[ a\_treat ] ++;

}

|  |  |  |  |  |  |  |  |
| --- | --- | --- | --- | --- | --- | --- | --- |
| |  |  |  |  |  |  | | --- | --- | --- | --- | --- | --- | | int Landscape::MagicMapP2PolyRef | ( | int | *a\_magic* | ) |  | | inline |

References m\_elems.

{

return m\_elems[ a\_magic ]->GetPoly();

}

|  |  |  |  |  |  |  |  |
| --- | --- | --- | --- | --- | --- | --- | --- |
| |  |  |  |  |  |  | | --- | --- | --- | --- | --- | --- | | void Landscape::MakeCluster | ( | void |  | ) |  | | protected |

Reset all polygons natural grazing level to zero.

Inc volegrazing at x,y Get volegrazing at x,y Calculate all vole grazing densities

|  |  |  |  |  |  |  |  |
| --- | --- | --- | --- | --- | --- | --- | --- |
| |  |  |  |  |  |  | | --- | --- | --- | --- | --- | --- | | void Landscape::ModifyPolyRef | ( | int \* |  | ) |  | | protected |

|  |  |  |  |  |  |  |  |
| --- | --- | --- | --- | --- | --- | --- | --- |
| |  |  |  |  |  |  | | --- | --- | --- | --- | --- | --- | | LE \* Landscape::NewElement | ( | TTypesOfLandscapeElement | *a\_type* | ) |  | | protected |

References g\_msg, LE::SetElementType(), LE::SetFileType(), tole\_ActivePit, tole\_AmenityGrass, tole\_BareRock, tole\_BeetleBank, tole\_Building, tole\_BuiltUpWithParkland, tole\_Coast, tole\_ConiferousForest, tole\_Copse, tole\_DeciduousForest, tole\_Field, tole\_FieldBoundary, tole\_Freshwater, tole\_Garden, tole\_Heath, tole\_HedgeBank, tole\_Hedges, tole\_LargeRoad, tole\_Marsh, tole\_MixedForest, tole\_NaturalGrass, tole\_Orchard, tole\_OrchardBand, tole\_OrchardGrass, tole\_Parkland, tole\_PermanentSetaside, tole\_PermPasture, tole\_PermPastureLowYield, tole\_PermPastureTussocky, tole\_PitDisused, tole\_Railway, tole\_River, tole\_RiversidePlants, tole\_RiversideTrees, tole\_RoadsideVerge, tole\_RuralResidential, tole\_Saltwater, tole\_SandDune, tole\_Scrub, tole\_SmallRoad, tole\_StoneWall, tole\_Suburban, tole\_Track, tole\_UnsprayedFieldMargin, tole\_Urban, tole\_UrbanNoVeg, tole\_UrbanPark, tole\_YoungForest, MapErrorMsg::Warn(), and WARN\_FILE.

Referenced by BeetleBankAdd(), BorderAdd(), hb\_GenerateHBPolys(), OrchardBorderAdd(), ReadPolys(), and UnsprayedMarginAdd().

{

LE \* elem;

static char error\_num[ 20 ];

switch ( a\_type ) {

case tole\_Hedges:

elem = new Hedges;

break;

case tole\_HedgeBank:

elem = new HedgeBank;

elem->SetFileType( 140 );

break;

case tole\_BeetleBank:

elem = new BeetleBank;

elem->SetFileType( 141 );

break;

case tole\_RoadsideVerge:

elem = new RoadsideVerge;

break;

case tole\_Railway:

elem = new Railway;

break;

case tole\_FieldBoundary:

elem = new FieldBoundary;

elem->SetFileType( 160 );

break;

case tole\_Marsh:

elem = new Marsh;

break;

case tole\_Orchard:

elem = new Orchard;

break;

case tole\_OrchardBand:

elem = new OrchardBand;

break;

case tole\_OrchardGrass:

elem = new OrchardGrass;

break;

case tole\_Heath:

elem = new Heath;

break;

case tole\_Scrub:

elem = new Scrub;

break;

case tole\_Field:

elem = new Field;

break;

case tole\_PermanentSetaside:

elem = new PermanentSetaside;

break;

case tole\_PermPasture:

elem = new PermPasture;

break;

case tole\_PermPastureLowYield:

elem = new PermPastureLowYield;

break;

case tole\_PermPastureTussocky:

elem = new PermPastureTussocky;

break;

case tole\_NaturalGrass:

elem = new NaturalGrass;

break;

case tole\_RiversidePlants:

elem = new RiversidePlants;

break;

case tole\_PitDisused:

elem = new PitDisused;

break;

case tole\_RiversideTrees:

elem = new RiversideTrees;

break;

case tole\_DeciduousForest:

elem = new DeciduousForest;

break;

case tole\_MixedForest:

elem = new MixedForest;

break;

case tole\_YoungForest:

elem = new YoungForest;

break;

case tole\_ConiferousForest:

elem = new ConiferousForest;

break;

case tole\_StoneWall:

elem = new StoneWall;

break;

case tole\_Garden:

elem = new Garden;

break;

case tole\_Track:

elem = new Track;

break;

case tole\_SmallRoad:

elem = new SmallRoad;

break;

case tole\_LargeRoad:

elem = new LargeRoad;

break;

case tole\_Building:

elem = new Building;

break;

case tole\_Urban:

elem = new Urban;

break;

case tole\_ActivePit:

elem = new ActivePit;

break;

case tole\_Freshwater:

elem = new Freshwater;

break;

case tole\_River:

elem = new River;

break;

case tole\_Saltwater:

elem = new Saltwater;

break;

case tole\_Coast:

elem = new Coast;

break;

case tole\_BareRock:

elem = new BareRock;

break;

case tole\_AmenityGrass:

elem = new AmenityGrass;

break;

case tole\_Parkland:

elem = new Parkland;

break;

case tole\_UrbanNoVeg:

elem = new UrbanNoVeg;

break;

case tole\_UrbanPark:

elem = new UrbanPark;

break;

case tole\_Suburban:

elem = new Suburban;

break;

case tole\_RuralResidential:

elem = new RuralResidential;

break;

case tole\_BuiltUpWithParkland:

elem = new BuiltUpWithParkland;

break;

case tole\_SandDune:

elem = new SandDune;

break;

case tole\_Copse:

elem = new Copse;

break;

case tole\_UnsprayedFieldMargin:

elem = new UnsprayedFieldMargin;

elem->SetFileType( 31 );

break;

default:

sprintf( error\_num, "%d", a\_type );

g\_msg->Warn( WARN\_FILE, "Landscape::NewElement(): Unknown landscape element requested:", error\_num );

exit( 1 );

} //switch

elem->SetElementType( a\_type );

return elem;

}

|  |  |  |  |  |  |  |  |
| --- | --- | --- | --- | --- | --- | --- | --- |
| |  |  |  |  |  |  | | --- | --- | --- | --- | --- | --- | | void Landscape::OrchardBorderAdd | ( | LE \* | *a\_field* | ) |  | | protected |

References BorderScan2(), g\_msg, RasterMap::Get(), LE::GetValidX(), LE::GetValidY(), hb\_first\_free\_poly\_num, m\_elems, m\_land, m\_polymapping, NewElement(), LE::SetArea(), LE::SetBorder(), LE::SetPoly(), tole\_NaturalGrass, MapErrorMsg::Warn(), and WARN\_BUG.

Referenced by Landscape().

{

int index;

int a\_x = a\_orchard->GetValidX();

int a\_y = a\_orchard->GetValidY();

if ( ( a\_x == -1 ) || ( a\_y == -1 ) ) {

g\_msg->Warn( WARN\_BUG, "Landscape::BorderAdd(): Uninitialized border coordinate!", "" );

exit( 1 );

}

index = m\_land->Get(a\_x,a\_y);

LE \* border = NewElement( tole\_NaturalGrass );

a\_orchard->SetBorder( border );

m\_polymapping[ hb\_first\_free\_poly\_num ] = (int) m\_elems.size();

m\_elems.resize( m\_elems.size() + 1 );

m\_elems[ m\_elems.size() - 1 ] = border;

border->SetPoly( hb\_first\_free\_poly\_num++ );

border->SetArea( 0.0 );

BorderScan2( a\_orchard );

}

|  |  |  |  |  |  |  |  |
| --- | --- | --- | --- | --- | --- | --- | --- |
| |  |  |  |  |  |  | | --- | --- | --- | --- | --- | --- | | void Landscape::PolysDump | ( | const char \* | *a\_filename* | ) |  | | protected |

References g\_msg, l\_map\_renumberpolys, m\_elems, CfgBool::value(), MapErrorMsg::Warn(), and WARN\_FILE.

Referenced by Landscape().

{

FILE \* outf;

int l\_num\_polys = 0;

// Count up number if active polygons in our list.

unsigned sz=(unsigned)m\_elems.size();

for ( unsigned int i = 0; i < sz; i++ ) {

if ( m\_elems[ i ]->GetMapValid() )

l\_num\_polys++;

}

outf = fopen(a\_filename, "w" );

if ( !outf ) {

g\_msg->Warn( WARN\_FILE, "Landscape::PolysDump(): Unable to open file", a\_filename );

exit( 1 );

}

fprintf( outf, "%d\n", l\_num\_polys );

if ( !l\_map\_renumberpolys.value() ) {

for ( unsigned int i = 0; i < m\_elems.size(); i++ ) {

if ( m\_elems[ i ]->GetMapValid() ) {

fprintf( outf, "%3d\t%6d\t%10.2f\t%4d\t%6d\n", m\_elems[ i ]->GetFileType(), m\_elems[ i ]->GetPoly(), m\_elems[ i ]->GetArea(),

m\_elems[ i ]->GetOwnerFile(), m\_elems[ i ]->GetUnsprayedMarginPolyRef() );

}

}

} else {

// First need a second list of polyrefs

vector < int > oldpolys;

oldpolys.resize( m\_elems.size() );

for ( unsigned int i = 0; i < m\_elems.size(); i++ ) {

if ( m\_elems[ i ]->GetMapValid() ) {

oldpolys[ i ] = m\_elems[ i ]->GetPoly();

// Need to reset the poly number

m\_elems[ i ]->SetPoly( i );

}

}

// Now need to go through and set the unsprayed margin poly refs

for ( unsigned int j = 0; j < m\_elems.size(); j++ ) {

int um = m\_elems[ j ]->GetUnsprayedMarginPolyRef();

if ( um != -1 ) {

unsigned ff = (int) oldpolys.size();

for ( unsigned int u = 0; u < ff; u++ ) {

if ( oldpolys[ u ] == um ) {

m\_elems[ j ]->SetUnsprayedMarginPolyRef( u );

break;

}

}

}

}

// Now we can output the file

for ( unsigned int i = 0; i < m\_elems.size(); i++ ) {

if ( m\_elems[ i ]->GetMapValid() ) {

fprintf( outf, "%3d\t%6d\t%10.2f\t%4d\t%6d\n", m\_elems[ i ]->GetFileType(), m\_elems[ i ]->GetPoly(), m\_elems[ i ]->GetArea(),

m\_elems[ i ]->GetOwnerFile(), m\_elems[ i ]->GetUnsprayedMarginPolyRef() );

}

}

}

fclose(outf);

}

|  |  |  |  |  |  |  |  |
| --- | --- | --- | --- | --- | --- | --- | --- |
| |  |  |  |  |  |  | | --- | --- | --- | --- | --- | --- | | void Landscape::PolysRemoveInvalid | ( | void |  | ) |  | | protected |

References m\_elems, and m\_polymapping.

Referenced by Landscape().

{

vector < LE \* > l\_temp;

unsigned int sz= (int) m\_elems.size();

for ( unsigned int i = 0; i < sz; i++ ) {

if ( m\_elems[ i ]->GetMapValid() ) {

unsigned int j = (int) l\_temp.size();

l\_temp.resize( j + 1 );

l\_temp[ j ] = m\_elems[ i ];

} else {

m\_polymapping[ m\_elems[ i ]->GetPoly() ] = -1;

delete m\_elems[ i ];

}

}

for ( unsigned int i = 0; i < l\_temp.size(); i++ ) {

m\_elems[ i ] = l\_temp[ i ];

m\_elems[ i ]->SetMapValid( true ); // CJT Changed from false 10/07/2006 - do not understand why it was false

}

m\_elems.resize( l\_temp.size() );

// Rebuild m\_polymapping.

sz= (int) m\_elems.size();

for ( unsigned int i = 0; i < sz; i++ ) {

m\_polymapping[ m\_elems[ i ]->GetPoly() ] = i;

}

}

|  |  |  |  |  |  |  |  |
| --- | --- | --- | --- | --- | --- | --- | --- |
| |  |  |  |  |  |  | | --- | --- | --- | --- | --- | --- | | void Landscape::PolysValidate | ( | bool | *a\_exit\_on\_invalid* | ) |  | | protected |

References g\_msg, RasterMap::Get(), m\_elems, m\_land, m\_polymapping, RasterMap::MapHeight(), RasterMap::MapWidth(), MapErrorMsg::Warn(), and WARN\_FILE.

Referenced by Landscape().

{

int mapwidth = m\_land->MapWidth();

int mapheight = m\_land->MapHeight();

// First loop just sets the MapValid as false (and checks for a major screw-up if this elemenent does not exist even in the list

for ( unsigned int i = 0; i < m\_elems.size(); i++ ) {

m\_elems[ i ]->SetMapValid( false );

if ( m\_polymapping[ m\_elems[ i ]->GetPoly() ] == -1 ) {

char l\_err[ 20 ];

sprintf( l\_err, "%d", m\_elems[ i ]->GetPoly() );

g\_msg->Warn( WARN\_FILE, "Landscape::PolysValidate(): Invalid polymapping ", l\_err );

exit( 1 );

}

}

// Now go through the whole map and for each polygon found set MapValid as true.

for ( int x = 0; x < mapwidth; x++ ) {

for ( int y = 0; y < mapheight; y++ ) {

int polynum = m\_land->Get( x, y );

// Mark that we have seen this polygon.

m\_elems[ m\_polymapping[ polynum ]]->SetMapValid( true );

}

}

if ( a\_exit\_on\_invalid ) {

for ( unsigned int i = 0; i < m\_elems.size(); i++ ) {

if ( !m\_elems[ i ]->GetMapValid() ) {

char l\_err[ 20 ];

sprintf( l\_err, "%d", m\_elems[ i ]->GetPoly() );

g\_msg->Warn( WARN\_FILE, "Landscape::PolysValidate(): Invalid polygon ", l\_err );

exit( 0 );

}

}

}

}

|  |  |  |  |  |  |
| --- | --- | --- | --- | --- | --- |
| char \* Landscape::PolytypeToString | ( | TTypesOfLandscapeElement | *a\_le\_type* | ) |  |

References g\_msg, tole\_ActivePit, tole\_AmenityGrass, tole\_BareRock, tole\_Building, tole\_BuiltUpWithParkland, tole\_Coast, tole\_ConiferousForest, tole\_Copse, tole\_DeciduousForest, tole\_Field, tole\_FieldBoundary, tole\_Foobar, tole\_Freshwater, tole\_Garden, tole\_Heath, tole\_HedgeBank, tole\_Hedges, tole\_LargeRoad, tole\_Marsh, tole\_MixedForest, tole\_NaturalGrass, tole\_Orchard, tole\_OrchardBand, tole\_OrchardGrass, tole\_Parkland, tole\_PermanentSetaside, tole\_PermPasture, tole\_PermPastureLowYield, tole\_PermPastureTussocky, tole\_PitDisused, tole\_Railway, tole\_River, tole\_RiversidePlants, tole\_RiversideTrees, tole\_RoadsideVerge, tole\_RuralResidential, tole\_Saltwater, tole\_SandDune, tole\_Scrub, tole\_SmallRoad, tole\_StoneWall, tole\_Suburban, tole\_Track, tole\_UnsprayedFieldMargin, tole\_Urban, tole\_UrbanNoVeg, tole\_UrbanPark, tole\_YoungForest, MapErrorMsg::Warn(), and WARN\_FILE.

Referenced by hb\_Add().

{

char error\_num[ 20 ];

switch ( a\_le\_type ) {

case tole\_Hedges:

return " Hedge";

case tole\_RoadsideVerge:

return " Roadside Verge";

case tole\_Railway:

return " Railway";

case tole\_FieldBoundary:

return " Field Boundary";

case tole\_Marsh:

return " Marsh";

case tole\_Scrub:

return " Scrub";

case tole\_Field:

return " Field";

case tole\_PermPastureTussocky:

return " PermPastureTussocky";

case tole\_PermanentSetaside:

return " Permanent Setaside";

case tole\_PermPasture:

return " Permanent Pasture";

case tole\_PermPastureLowYield:

return " PermPastureLowYield";

case tole\_NaturalGrass:

return " Natural Grass";

case tole\_RiversidePlants:

return " Riverside Plants";

case tole\_PitDisused:

return " Pit Disused";

case tole\_RiversideTrees:

return " Riverside Trees";

case tole\_DeciduousForest:

return " Deciduous Forest";

case tole\_MixedForest:

return " Mixed Forest";

case tole\_ConiferousForest:

return " Coniferous Forest";

case tole\_YoungForest:

return " Young Forest";

case tole\_StoneWall:

return " Stone Wall";

case tole\_Garden:

return " Garden";

case tole\_Track:

return " Track";

case tole\_SmallRoad:

return " Small Road";

case tole\_LargeRoad:

return " Large Road";

case tole\_Building:

return " Building";

case tole\_Urban:

return " Urban";

case tole\_ActivePit:

return " Active Pit";

case tole\_Freshwater:

return " Fresh Water";

case tole\_River:

return " River";

case tole\_Saltwater:

return " Saltwater";

case tole\_Coast:

return " Coast";

case tole\_BareRock:

return " Bare Rock";

case tole\_HedgeBank:

return " Hedgebank";

case tole\_Heath:

return " Heath";

case tole\_Orchard:

return " Orchard";

case tole\_OrchardBand:

return " Orchard Band";

case tole\_OrchardGrass:

return " Orchard Grass";

case tole\_UnsprayedFieldMargin:

return " UnsprayedFieldMargin";

case tole\_AmenityGrass:

return " AmenityGrass";

case tole\_Parkland:

return " Parkland";

case tole\_UrbanNoVeg:

return " UrbanNoVeg";

case tole\_UrbanPark:

return " UrbanPark";

case tole\_Suburban:

return " Suburban";

case tole\_RuralResidential:

return " RuralResidential";

case tole\_BuiltUpWithParkland:

return " BuiltUpWithParkland";

case tole\_SandDune:

return " SandDune";

case tole\_Copse:

return " Copse";

case tole\_Foobar:

default:

sprintf( error\_num, "%d", a\_le\_type );

g\_msg->Warn( WARN\_FILE, "Landscape::PolytypeToString(): Unknown event type:", error\_num );

exit( 1 );

}

}

|  |  |  |  |  |  |  |  |
| --- | --- | --- | --- | --- | --- | --- | --- |
| |  |  |  |  |  |  | | --- | --- | --- | --- | --- | --- | | Point Landscape::RandomLocation | ( | void |  | ) |  | | protected |

|  |  |  |  |  |  |  |  |  |  |  |  |  |  |  |  |  |  |  |  |  |  |
| --- | --- | --- | --- | --- | --- | --- | --- | --- | --- | --- | --- | --- | --- | --- | --- | --- | --- | --- | --- | --- | --- |
| |  |  |  |  | | --- | --- | --- | --- | | void Landscape::ReadInput | ( | int \* | , | |  |  | int \* | , | |  |  | int \* | , | |  |  | Point \* |  | |  | ) |  |  | | protected |

|  |  |  |  |  |  |  |  |
| --- | --- | --- | --- | --- | --- | --- | --- |
| |  |  |  |  |  |  | | --- | --- | --- | --- | --- | --- | | void Landscape::ReadPolys | ( | const char \* | *a\_polyfile* | ) |  | | protected |

References FarmManager::AddField(), cfg\_map\_usesoiltypes, FarmManager::ConnectFarm(), g\_letype, g\_map\_le\_border\_chance, g\_map\_le\_borders, g\_map\_le\_unsprayedmargins, g\_map\_le\_unsprayedmargins\_chance, g\_map\_maxpolyref, g\_msg, LE::GetElementType(), l\_map\_chameleon\_replace\_num, m\_elems, m\_FarmManager, m\_farmmapping, m\_LargestPolyNumUsed, m\_polymapping, NewElement(), random(), LE::SetArea(), LE::SetBorder(), LE::SetCentroid(), LE::SetFileType(), LE::SetPoly(), LE::SetSoilType(), LE::SetUnsprayedMarginPolyRef(), tole\_Field, tole\_PermanentSetaside, tole\_PermPasture, tole\_PermPastureLowYield, tole\_PermPastureTussocky, tole\_YoungForest, LE\_TypeClass::TranslateEleTypes(), CfgInt::value(), CfgBool::value(), MapErrorMsg::Warn(), and WARN\_FILE.

Referenced by Landscape().

{

// The polygon file consists of 4 columns:

// 1. Polygon Number 2. Type 3. Area as a double 4. Owner. 5- -1 or unsprayed margin polynum

// If Owner is '-1', then no owner.

// Columns 6 & 7 are the x,y centroids if used.

FILE \* inpf;

int NoPolygons;

char error\_num[ 20 ];

inpf = fopen(a\_polyfile, "r" );

if ( !inpf ) {

g\_msg->Warn( WARN\_FILE, "Landscape::ReadPolys(): Unable to open file", a\_polyfile );

exit( 1 );

}

fscanf( inpf, "%d", & NoPolygons );

m\_elems.resize( NoPolygons );

m\_polymapping = ( int \* ) malloc( sizeof( int ) \* ( g\_map\_maxpolyref.value() \* 10 + 1 ) );

m\_farmmapping = ( int \* ) malloc( sizeof( int ) \* ( g\_map\_maxpolyref.value() \* 2 + 1 ) );

if ( m\_polymapping == NULL || m\_farmmapping == NULL ) {

g\_msg->Warn( WARN\_FILE, "Landscape::ReadPolys(): Out of memory!", "" );

exit( 1 );

}

// Set all mappings to unused.

for ( int i = 0; i < g\_map\_maxpolyref.value() \* 2 + 1; i++ ) {

m\_polymapping[ i ] = -1;

m\_farmmapping[ i ] = -1;

}

int ElemIndex = 0;

for ( int x = 0; x < NoPolygons; x++ ) {

int PolyNum, Owner, FileType, RealFileType, URef, SoilType, Cx, Cy;

TTypesOfLandscapeElement Type;

float Area;

Cx=-1; Cy=-1; // These are not used unless the CIPE landscape maker is used

if (cfg\_map\_usesoiltypes.value()) {

if ( 6 != fscanf( inpf, "%d %d %g %d %d %d", & FileType, & PolyNum, & Area, & Owner, & URef, & SoilType) ) {

sprintf( error\_num, "%d", NoPolygons );

g\_msg->Warn( WARN\_FILE, "Landscape::ReadPolys(): Polygon file empty before "

"reading number of specified polygons:", error\_num );

exit( 1 );

}

}

else {

if ( 5 != fscanf( inpf, "%d %d %g %d %d", & FileType, & PolyNum, & Area, & Owner, & URef) ) {

sprintf( error\_num, "%d", NoPolygons );

g\_msg->Warn( WARN\_FILE, "Landscape::ReadPolys(): Polygon file empty before "

"reading number of specified polygons:", error\_num );

exit( 1 );

}

SoilType=-1;

}

RealFileType = FileType;

if ( FileType == 150 ) {

FileType = l\_map\_chameleon\_replace\_num.value();

}

Type = g\_letype->TranslateEleTypes( FileType );

if ( -1 == m\_polymapping[ PolyNum ] ) {

// First time we have encountered this polygon number.

// Borders are not mapped in this list.

m\_polymapping[ PolyNum ] = ElemIndex;

LE \* newland = NewElement( Type );

m\_elems[ ElemIndex++ ] = newland;

newland->SetPoly( PolyNum );

newland->SetArea( floor( 0.5 + Area ) );

newland->SetFileType( RealFileType );

newland->SetSoilType( SoilType );

newland->SetUnsprayedMarginPolyRef( URef );

newland->SetCentroid( Cx, Cy );

// Just for fun, or maybe because we might need it later, remember the actual largest polynum used

if (PolyNum>m\_LargestPolyNumUsed) m\_LargestPolyNumUsed=PolyNum;

// Two types of possible errors: Landscape element that is a field,

// but doesn't belong to a farm, or a farm element not of type field.

// Check for both cases.

if ( -1 == Owner && ( Type == tole\_Field || Type == tole\_PermPastureTussocky || Type == tole\_PermPasture || Type == tole\_PermanentSetaside || Type == tole\_PermPastureLowYield ) ) {

// No owner but field polygon.

sprintf( error\_num, "%d", PolyNum );

g\_msg->Warn( WARN\_FILE, "Landscape::ReadPolys(): Farm polygon does not belong"" to a farm:", error\_num );

exit( 1 );

}

if ( -1 != Owner && Type != tole\_Field && Type != tole\_YoungForest && Type != tole\_PermPastureTussocky && Type != tole\_PermPasture && Type != tole\_PermanentSetaside && Type != tole\_PermPastureLowYield) {

// An owner but not field elements.

sprintf( error\_num, "%d", PolyNum );

g\_msg->Warn( WARN\_FILE, "Landscape::ReadPolys(): Farm polygon does not have"" element type tole\_Field:", error\_num );

exit( 1 );

}

if ( -1 != Owner ) {

if ( -1 == m\_farmmapping[ Owner ] )

m\_farmmapping[ Owner ] = m\_FarmManager->ConnectFarm( Owner );

int OwnerIndex = m\_farmmapping[ Owner ];

m\_FarmManager->AddField(OwnerIndex,newland, Owner);

if ( g\_map\_le\_borders.value() ) {

if ( random( 100 ) < g\_map\_le\_border\_chance.value() ) {

// This is a farm element, so signal adding a border.

newland->SetBorder( ( LE \* ) 1 );

}

}

// Code to generate unsprayed margins....

if ( newland->GetElementType() == tole\_Field ) {

if ( g\_map\_le\_unsprayedmargins.value() ) {

if ( random( 100 ) < g\_map\_le\_unsprayedmargins\_chance.value() ) {

// This is a farm field, so signal adding a margin

newland->SetUnsprayedMarginPolyRef( 1 );

}

}

}

// ..to here

}

} else {

sprintf( error\_num, "%d", PolyNum );

g\_msg->Warn( WARN\_FILE, "Landscape::ReadPolys(): Duplicate polygon in file", error\_num );

exit( 1 );

}

}

fclose( inpf );

}

|  |  |  |  |  |  |  |  |
| --- | --- | --- | --- | --- | --- | --- | --- |
| |  |  |  |  |  |  | | --- | --- | --- | --- | --- | --- | | bool Landscape::ReadSymbols | ( | const char \* | *a\_cfgfile* | ) |  | | inline |

References g\_cfg, and Configurator::ReadSymbols().

{

return g\_cfg->ReadSymbols( a\_cfgfile );

}

|  |  |  |  |
| --- | --- | --- | --- |
| void Landscape::SetLESignal | ( | int | *a\_polyref*, |
|  |  | LE\_Signal | *a\_signal* |
|  | ) |  |  |

References m\_elems, and m\_polymapping.

Referenced by Landscape().

{

m\_elems[ m\_polymapping[ a\_polyref ]]->SetSignal( a\_signal );

}

|  |  |  |  |  |  |
| --- | --- | --- | --- | --- | --- |
| void Landscape::SetPolyMaxMinExtents | ( | void |  | ) |  |

References RasterMap::Get(), m\_elems, m\_land, RasterMap::MapHeight(), and RasterMap::MapWidth().

Referenced by Landscape().

{

// All polygon manipulation is settled now we need to give the polygons some information about themselves

// This takes a little time but save time later one

int mwidth = m\_land->MapWidth();

int mheight = m\_land->MapHeight();

for ( int x = 0; x < mwidth; x++ ) {

for ( int y = 0; y < mheight; y++ ) {

int polynum = m\_land->Get( x, y );

// Mark that we have seen this polygon.

unsigned int ele\_ref= polynum;

if (m\_elems[ ele\_ref]->GetMaxX( ) < x) m\_elems[ ele\_ref]->SetMaxX( x );

if (m\_elems[ ele\_ref]->GetMaxY( ) < y) m\_elems[ ele\_ref]->SetMaxY( y );

if (m\_elems[ ele\_ref]->GetMinX( ) > x) m\_elems[ ele\_ref]->SetMinX( x );

if (m\_elems[ ele\_ref]->GetMinY( ) > y) m\_elems[ ele\_ref]->SetMinY( y );

}

}

}

|  |  |  |  |  |  |
| --- | --- | --- | --- | --- | --- |
| void Landscape::SkylarkEvaluation | ( | SkTerritories \* | *a\_skt* | ) |  |

References m\_elems, and SkTerritories::PreCachePoly().

Referenced by SkTerritories::PreFillQualGrid().

{

for (unsigned i=0; i<m\_elems.size(); i++) {

a\_skt->PreCachePoly(m\_elems[i]->GetPoly());

}

}

|  |  |  |  |  |  |  |  |  |  |  |  |  |  |  |  |  |  |  |  |  |  |
| --- | --- | --- | --- | --- | --- | --- | --- | --- | --- | --- | --- | --- | --- | --- | --- | --- | --- | --- | --- | --- | --- |
| |  |  |  |  | | --- | --- | --- | --- | | bool Landscape::StepOneValid | ( | int | *a\_polyindex*, | |  |  | int | *a\_x*, | |  |  | int | *a\_y*, | |  |  | int | *step* | |  | ) |  |  | | protected |

References RasterMap::Get(), m\_elems, m\_land, RasterMap::MapHeight(), and RasterMap::MapWidth().

Referenced by BorderScan2().

{

int index;

int x\_add[ 8 ] = { 1\*a\_step, 1\*a\_step, 0, -1\*a\_step, -1\*a\_step, -1\*a\_step, 0, 1\*a\_step };

int y\_add[ 8 ] = { 0, -1\*a\_step, -1\*a\_step, -1\*a\_step, 0, 1\*a\_step, 1\*a\_step, 1\*a\_step };

int width = m\_land->MapWidth();

int height = m\_land->MapHeight();

// Scan anti-clockwise from center pixel coordinate.

for ( unsigned int i = 0; i < 8; i++ ) {

if ( ( a\_x + x\_add[ i ] < width ) && ( a\_x + x\_add[ i ] >= 0 ) && ( a\_y + y\_add[ i ] < height ) && ( a\_y + y\_add[ i ] >= 0 ) )

{

index = m\_land->Get( a\_x + x\_add[ i ], a\_y + y\_add[ i ] );

if ( index == a\_polyindex )

{

m\_elems[a\_polyindex]->SetValidXY(a\_x + x\_add[ i ], a\_y + y\_add[ i ]);

return true;

}

}

}

return false;

}

|  |  |  |  |  |  |  |  |  |  |  |  |  |  |
| --- | --- | --- | --- | --- | --- | --- | --- | --- | --- | --- | --- | --- | --- |
| |  |  |  |  | | --- | --- | --- | --- | | int Landscape::SupplyCountryDesig | ( | int | *a\_x*, | |  |  | int | *a\_y* | |  | ) |  |  | | inline |

References RasterMap::Get(), m\_elems, and m\_land.

{

return m\_elems[ m\_land->Get( a\_x, a\_y ) ]->GetCountryDesignation();

}

|  |  |  |  |  |  |  |  |
| --- | --- | --- | --- | --- | --- | --- | --- |
| |  |  |  |  |  |  | | --- | --- | --- | --- | --- | --- | | double Landscape::SupplyDayDegrees | ( | int | *a\_polyref* | ) |  | | inline |

References m\_elems, and m\_polymapping.

{

return m\_elems[ m\_polymapping[ a\_polyref ]]->GetDayDegrees();

}

|  |  |  |  |  |  |  |  |
| --- | --- | --- | --- | --- | --- | --- | --- |
| |  |  |  |  |  |  | | --- | --- | --- | --- | --- | --- | | int Landscape::SupplyDayInMonth | ( | void |  | ) |  | | inline |

References g\_date, and Calendar::GetDayInMonth().

Referenced by RunTheSim().

{

return g\_date->GetDayInMonth();

}

|  |  |  |  |  |  |  |  |
| --- | --- | --- | --- | --- | --- | --- | --- |
| |  |  |  |  |  |  | | --- | --- | --- | --- | --- | --- | | long Landscape::SupplyDayInYear | ( | void |  | ) |  | | inline |

References Calendar::DayInYear(), and g\_date.

Referenced by Skylark\_Population\_Manager::Catastrophe(), Skylark\_Population\_Manager::DoFirst(), EventDump(), Skylark\_Nestling::OnDeserted(), Skylark\_Female::OnMateDying(), Skylark\_Male::OnMateDying(), Skylark\_Female::OnMateHomeless(), Skylark\_Male::OnMateLeaving(), Skylark\_Nestling::OnYouHaveBeenEaten(), Skylark\_Male::st\_AttractingAMate(), Skylark\_Female::st\_BuildingUpResources(), Skylark\_Female::st\_CaringForYoung(), Skylark\_Clutch::st\_Developing(), Skylark\_Nestling::st\_Developing(), Skylark\_Female::st\_Dying(), Skylark\_Female::st\_Emigrating(), Skylark\_Male::st\_Emigrating(), Skylark\_Female::st\_Floating(), Skylark\_Male::st\_Floating(), Skylark\_Female::st\_Flocking(), Skylark\_Male::st\_Flocking(), Skylark\_Female::st\_GivingUpTerritory(), Skylark\_Female::st\_MakingNest(), Skylark\_Female::st\_PreparingForBreeding(), Skylark\_Female::st\_StartingNewBrood(), Skylark\_Female::st\_StoppingBreeding(), Skylark\_Female::Step(), and VegDump().

{

return g\_date->DayInYear();

}

|  |  |  |  |  |  |  |  |
| --- | --- | --- | --- | --- | --- | --- | --- |
| |  |  |  |  |  |  | | --- | --- | --- | --- | --- | --- | | int Landscape::SupplyDaylength | ( | void |  | ) |  | | inline |

References Calendar::DayLength(), and g\_date.

Referenced by Skylark\_Female::FeedYoung(), Skylark\_Male::st\_CaringForYoung(), Skylark\_Female::st\_Incubating(), and Skylark\_Female::st\_PreparingForBreeding().

{

return g\_date->DayLength();

}

|  |  |  |  |  |  |  |  |
| --- | --- | --- | --- | --- | --- | --- | --- |
| |  |  |  |  |  |  | | --- | --- | --- | --- | --- | --- | | int Landscape::SupplyDaylength | ( | long | *a\_date* | ) |  | | inline |

References Calendar::DayLength(), and g\_date.

{

return g\_date->DayLength( a\_date );

}

|  |  |  |  |  |  |  |  |
| --- | --- | --- | --- | --- | --- | --- | --- |
| |  |  |  |  |  |  | | --- | --- | --- | --- | --- | --- | | double Landscape::SupplyDeadBiomass | ( | int | *a\_polyref* | ) |  | | inline |

References m\_elems, and m\_polymapping.

Referenced by VegDump().

{

return m\_elems[ m\_polymapping[ a\_polyref ]]->GetDeadBiomass();

}

|  |  |  |  |  |  |  |  |  |  |  |  |  |  |
| --- | --- | --- | --- | --- | --- | --- | --- | --- | --- | --- | --- | --- | --- |
| |  |  |  |  | | --- | --- | --- | --- | | double Landscape::SupplyDeadBiomass | ( | int | *a\_x*, | |  |  | int | *a\_y* | |  | ) |  |  | | inline |

References RasterMap::Get(), m\_elems, and m\_land.

{

return m\_elems[ m\_land->Get( a\_x, a\_y ) ]->GetDeadBiomass();

}

|  |  |  |  |  |  |  |  |
| --- | --- | --- | --- | --- | --- | --- | --- |
| |  |  |  |  |  |  | | --- | --- | --- | --- | --- | --- | | int Landscape::SupplyElementSubType | ( | int | *a\_polyref* | ) |  | | inline |

References m\_elems, and m\_polymapping.

{

return m\_elems[ m\_polymapping[ a\_polyref ]]->GetSubType();

}

|  |  |  |  |  |  |  |  |  |  |  |  |  |  |
| --- | --- | --- | --- | --- | --- | --- | --- | --- | --- | --- | --- | --- | --- |
| |  |  |  |  | | --- | --- | --- | --- | | int Landscape::SupplyElementSubType | ( | int | *a\_x*, | |  |  | int | *a\_y* | |  | ) |  |  | | inline |

References RasterMap::Get(), m\_elems, and m\_land.

{

return m\_elems[ m\_land->Get( a\_x, a\_y ) ]->GetSubType();

}

|  |  |  |  |  |  |  |  |
| --- | --- | --- | --- | --- | --- | --- | --- |
| |  |  |  |  |  |  | | --- | --- | --- | --- | --- | --- | | TTypesOfLandscapeElement Landscape::SupplyElementType | ( | int | *a\_polyref* | ) |  | | inline |

References m\_elems, and m\_polymapping.

Referenced by DumpMapGraphics(), SkTerritories::DumpMapGraphics(), Pesticide::ElementIsWater(), MovementMap::Init(), Landscape(), SkTerritories::PrePoly2Qual(), SkTerritories::PrePolyNQual(), and skTTerritory::TestNestPossibility().

{

return m\_elems[ m\_polymapping[ a\_polyref ]]->GetElementType();

}

|  |  |  |  |  |  |  |  |  |  |  |  |  |  |
| --- | --- | --- | --- | --- | --- | --- | --- | --- | --- | --- | --- | --- | --- |
| |  |  |  |  | | --- | --- | --- | --- | | TTypesOfLandscapeElement Landscape::SupplyElementType | ( | int | *a\_x*, | |  |  | int | *a\_y* | |  | ) |  |  | | inline |

References RasterMap::Get(), m\_elems, and m\_land.

{

return m\_elems[ m\_land->Get( a\_x, a\_y ) ]->GetElementType();

}

|  |  |  |  |  |  |  |  |  |  |  |  |  |  |
| --- | --- | --- | --- | --- | --- | --- | --- | --- | --- | --- | --- | --- | --- |
| |  |  |  |  | | --- | --- | --- | --- | | TTypesOfLandscapeElement Landscape::SupplyElementTypeCC | ( | int | *a\_x*, | |  |  | int | *a\_y* | |  | ) |  |  | | inline |

References RasterMap::Get(), m\_elems, m\_height, m\_height10, m\_land, m\_width, and m\_width10.

{

a\_x = (a\_x + m\_width10) % m\_width;

a\_y = (a\_y + m\_height10) % m\_height;

return m\_elems[ m\_land->Get( a\_x, a\_y ) ]->GetElementType();

}

|  |  |  |  |  |  |  |  |
| --- | --- | --- | --- | --- | --- | --- | --- |
| |  |  |  |  |  |  | | --- | --- | --- | --- | --- | --- | | TTypesOfLandscapeElement Landscape::SupplyElementTypeFromVector | ( | unsigned int | *a\_index* | ) |  | | inline |

References m\_elems.

{

return m\_elems[ a\_index ]->GetElementType();

}

|  |  |  |  |  |  |  |  |
| --- | --- | --- | --- | --- | --- | --- | --- |
| |  |  |  |  |  |  | | --- | --- | --- | --- | --- | --- | | int Landscape::SupplyFarmArea | ( | int | *a\_polyref* | ) |  | | inline |

References m\_elems, and m\_polymapping.

{

return m\_elems[ m\_polymapping[ a\_polyref ]]->GetOwner()->GetArea();

}

|  |  |  |  |
| --- | --- | --- | --- |
| int Landscape::SupplyFarmIntensity | ( | int | *a\_x*, |
|  |  | int | *a\_y* |
|  | ) |  |  |

References RasterMap::Get(), m\_elems, m\_land, and m\_polymapping.

Referenced by VegElement::DoDevelopment(), and VegElement::RecalculateBugsNStuff().

{

return m\_elems[ m\_polymapping[ m\_land->Get( a\_x, a\_y ) ]]->GetOwner()->GetIntensity();

}

|  |  |  |  |  |  |
| --- | --- | --- | --- | --- | --- |
| int Landscape::SupplyFarmIntensity | ( | int | *a\_polyref* | ) |  |

References m\_elems, and m\_polymapping.

{

return m\_elems[ m\_polymapping[ a\_polyref ]]->GetOwner()->GetIntensity();

}

|  |  |  |  |  |  |  |  |  |  |  |  |  |  |
| --- | --- | --- | --- | --- | --- | --- | --- | --- | --- | --- | --- | --- | --- |
| |  |  |  |  | | --- | --- | --- | --- | | int Landscape::SupplyFarmOwner | ( | int | *a\_x*, | |  |  | int | *a\_y* | |  | ) |  |  | | inline |

References RasterMap::Get(), m\_elems, and m\_land.

Referenced by Skylark\_Population\_Manager::BreedingPairsOutput().

{

return m\_elems[ m\_land->Get( a\_x, a\_y ) ]->GetOwnerFile();

}

|  |  |  |  |  |  |  |  |
| --- | --- | --- | --- | --- | --- | --- | --- |
| |  |  |  |  |  |  | | --- | --- | --- | --- | --- | --- | | int Landscape::SupplyFarmOwner | ( | int | *a\_polyref* | ) |  | | inline |

References m\_elems, and m\_polymapping.

{

return m\_elems[ m\_polymapping[ a\_polyref ]]->GetOwnerFile();

}

|  |  |  |  |  |  |  |  |  |  |  |  |  |  |
| --- | --- | --- | --- | --- | --- | --- | --- | --- | --- | --- | --- | --- | --- |
| |  |  |  |  | | --- | --- | --- | --- | | int Landscape::SupplyFarmOwnerIndex | ( | int | *a\_x*, | |  |  | int | *a\_y* | |  | ) |  |  | | inline |

References RasterMap::Get(), m\_elems, and m\_land.

{

return m\_elems[ m\_land->Get( a\_x, a\_y ) ]->GetOwnerIndex();

}

|  |  |  |  |  |  |  |  |
| --- | --- | --- | --- | --- | --- | --- | --- |
| |  |  |  |  |  |  | | --- | --- | --- | --- | --- | --- | | int Landscape::SupplyFarmOwnerIndex | ( | int | *a\_polyref* | ) |  | | inline |

References m\_elems, and m\_polymapping.

{

return m\_elems[ m\_polymapping[ a\_polyref ]]->GetOwnerIndex();

}

|  |  |  |  |  |  |  |  |
| --- | --- | --- | --- | --- | --- | --- | --- |
| |  |  |  |  |  |  | | --- | --- | --- | --- | --- | --- | | TTypesOfFarm Landscape::SupplyFarmType | ( | int | *a\_polyref* | ) |  | | inline |

References m\_elems, and m\_polymapping.

{

return m\_elems[ m\_polymapping[ a\_polyref ]]->GetOwner()->GetType();

}

|  |  |  |  |  |  |  |  |  |  |  |  |  |  |
| --- | --- | --- | --- | --- | --- | --- | --- | --- | --- | --- | --- | --- | --- |
| |  |  |  |  | | --- | --- | --- | --- | | TTypesOfFarm Landscape::SupplyFarmType | ( | int | *a\_x*, | |  |  | int | *a\_y* | |  | ) |  |  | | inline |

References RasterMap::Get(), m\_elems, and m\_land.

{

return m\_elems[ m\_land->Get( a\_x, a\_y ) ]->GetOwner()->GetType();

}

|  |  |  |  |  |  |  |  |
| --- | --- | --- | --- | --- | --- | --- | --- |
| |  |  |  |  |  |  | | --- | --- | --- | --- | --- | --- | | long Landscape::SupplyGlobalDate | ( | void |  | ) |  | | inline |

References Calendar::Date(), and g\_date.

Referenced by Skylark\_Female::st\_PreparingForBreeding().

{

return g\_date->Date();

}

|  |  |  |  |  |  |  |
| --- | --- | --- | --- | --- | --- | --- |
| |  |  |  |  |  | | --- | --- | --- | --- | --- | | double Landscape::SupplyGlobalRadiation | ( |  | ) |  | | inline |

References g\_weather, and Weather::GetGlobalRadiation().

Referenced by VegElement::RecalculateBugsNStuff().

{

return g\_weather->GetGlobalRadiation( );

}

|  |  |  |  |  |  |  |  |
| --- | --- | --- | --- | --- | --- | --- | --- |
| |  |  |  |  |  |  | | --- | --- | --- | --- | --- | --- | | double Landscape::SupplyGlobalRadiation | ( | long | *a\_date* | ) |  | | inline |

References g\_weather, and Weather::GetGlobalRadiation().

{

return g\_weather->GetGlobalRadiation( a\_date );

}

|  |  |  |  |  |  |  |  |
| --- | --- | --- | --- | --- | --- | --- | --- |
| |  |  |  |  |  |  | | --- | --- | --- | --- | --- | --- | | bool Landscape::SupplyGrazingPressure | ( | int | *a\_polyref* | ) |  | | inline |

References m\_elems, and m\_polymapping.

Referenced by Skylark\_Clutch::OnFarmEvent(), and Skylark\_Nestling::OnFarmEvent().

{

return m\_elems[ m\_polymapping[ a\_polyref ] ]->GetCattleGrazing();

}

|  |  |  |  |  |  |  |  |  |  |  |  |  |  |
| --- | --- | --- | --- | --- | --- | --- | --- | --- | --- | --- | --- | --- | --- |
| |  |  |  |  | | --- | --- | --- | --- | | bool Landscape::SupplyGrazingPressure | ( | int | *a\_x*, | |  |  | int | *a\_y* | |  | ) |  |  | | inline |

References RasterMap::Get(), m\_elems, and m\_land.

{

return m\_elems[ m\_land->Get( a\_x, a\_y ) ]->GetCattleGrazing();

}

|  |  |  |  |  |  |  |  |
| --- | --- | --- | --- | --- | --- | --- | --- |
| |  |  |  |  |  |  | | --- | --- | --- | --- | --- | --- | | bool Landscape::SupplyGrazingPressureVector | ( | unsigned int | *a\_index* | ) |  | | inline |

References m\_elems.

{

return m\_elems[ a\_index ]->GetCattleGrazing();

}

|  |  |  |  |  |  |  |  |
| --- | --- | --- | --- | --- | --- | --- | --- |
| |  |  |  |  |  |  | | --- | --- | --- | --- | --- | --- | | double Landscape::SupplyGreenBiomass | ( | int | *a\_polyref* | ) |  | | inline |

References m\_elems, and m\_polymapping.

Referenced by VegDump().

{

return m\_elems[ m\_polymapping[ a\_polyref ]]->GetGreenBiomass();

}

|  |  |  |  |  |  |  |  |  |  |  |  |  |  |
| --- | --- | --- | --- | --- | --- | --- | --- | --- | --- | --- | --- | --- | --- |
| |  |  |  |  | | --- | --- | --- | --- | | double Landscape::SupplyGreenBiomass | ( | int | *a\_x*, | |  |  | int | *a\_y* | |  | ) |  |  | | inline |

References RasterMap::Get(), m\_elems, and m\_land.

{

return m\_elems[ m\_land->Get( a\_x, a\_y ) ]->GetGreenBiomass();

}

|  |  |  |  |  |  |  |  |  |  |  |  |  |  |
| --- | --- | --- | --- | --- | --- | --- | --- | --- | --- | --- | --- | --- | --- |
| |  |  |  |  | | --- | --- | --- | --- | | bool Landscape::SupplyHasTramlines | ( | int | *a\_x*, | |  |  | int | *a\_y* | |  | ) |  |  | | inline |

References RasterMap::Get(), m\_elems, and m\_land.

Referenced by Skylark\_Adult::GetVegHindrance(), and SkTerritories::PrePolyNQual().

{

return m\_elems[ m\_land->Get( a\_x, a\_y ) ]->HasTramlines();

}

|  |  |  |  |  |  |  |  |
| --- | --- | --- | --- | --- | --- | --- | --- |
| |  |  |  |  |  |  | | --- | --- | --- | --- | --- | --- | | bool Landscape::SupplyHasTramlines | ( | int | *a\_polyref* | ) |  | | inline |

References m\_elems, and m\_polymapping.

{

return m\_elems[ m\_polymapping[ a\_polyref ] ]->HasTramlines();

}

|  |  |  |  |  |  |  |  |
| --- | --- | --- | --- | --- | --- | --- | --- |
| |  |  |  |  |  |  | | --- | --- | --- | --- | --- | --- | | double Landscape::SupplyInsects | ( | int | *a\_polyref* | ) |  | | inline |

References m\_elems, and m\_polymapping.

Referenced by Skylark\_Male::GetFood(), and VegDump().

{

return m\_elems[ m\_polymapping[ a\_polyref ]]->GetInsectPop();

}

|  |  |  |  |  |  |  |  |  |  |  |  |  |  |
| --- | --- | --- | --- | --- | --- | --- | --- | --- | --- | --- | --- | --- | --- |
| |  |  |  |  | | --- | --- | --- | --- | | double Landscape::SupplyInsects | ( | int | *a\_x*, | |  |  | int | *a\_y* | |  | ) |  |  | | inline |

References RasterMap::Get(), m\_elems, and m\_land.

{

return m\_elems[ m\_land->Get( a\_x, a\_y ) ]->GetInsectPop();

}

|  |  |  |  |  |  |  |  |
| --- | --- | --- | --- | --- | --- | --- | --- |
| |  |  |  |  |  |  | | --- | --- | --- | --- | --- | --- | | bool Landscape::SupplyIsGrass | ( | int | *a\_polyref* | ) |  | | inline |

References m\_elems, m\_polymapping, tov\_CloverGrassGrazed1, tov\_CloverGrassGrazed2, tov\_FodderGrass, tov\_NaturalGrass, tov\_OCloverGrassGrazed1, tov\_OCloverGrassGrazed2, tov\_Orchard, tov\_OSeedGrass1, tov\_OSeedGrass2, tov\_PermanentGrassGrazed, tov\_PermanentGrassLowYield, tov\_PermanentGrassTussocky, tov\_PermanentSetaside, tov\_SeedGrass1, tov\_SeedGrass2, tov\_Setaside, and tov\_YoungForest.

{

TTypesOfVegetation vtype = m\_elems[ m\_polymapping[ a\_polyref ] ]->GetVegType();

switch (vtype) {

case tov\_NaturalGrass:

case tov\_PermanentGrassGrazed:

case tov\_PermanentGrassLowYield:

case tov\_PermanentGrassTussocky:

case tov\_PermanentSetaside:

case tov\_Setaside:

case tov\_SeedGrass1:

case tov\_SeedGrass2:

case tov\_OSeedGrass1:

case tov\_OSeedGrass2:

case tov\_CloverGrassGrazed1:

case tov\_CloverGrassGrazed2:

case tov\_OCloverGrassGrazed1:

case tov\_OCloverGrassGrazed2:

case tov\_Orchard:

case tov\_YoungForest:

case tov\_FodderGrass:

return true;

default: return false;

}

}

|  |  |  |  |  |  |  |  |
| --- | --- | --- | --- | --- | --- | --- | --- |
| |  |  |  |  |  |  | | --- | --- | --- | --- | --- | --- | | bool Landscape::SupplyJustMown | ( | int | *a\_polyref* | ) |  | | inline |

References m\_elems, and m\_polymapping.

{

return m\_elems[ m\_polymapping[ a\_polyref ] ]->IsRecentlyMown();

}

|  |  |  |  |  |  |  |  |
| --- | --- | --- | --- | --- | --- | --- | --- |
| |  |  |  |  |  |  | | --- | --- | --- | --- | --- | --- | | bool Landscape::SupplyJustMownVector | ( | unsigned int | *a\_index* | ) |  | | inline |

References m\_elems.

{

return m\_elems[ a\_index ]->IsRecentlyMown();

}

|  |  |  |  |  |  |  |  |
| --- | --- | --- | --- | --- | --- | --- | --- |
| |  |  |  |  |  |  | | --- | --- | --- | --- | --- | --- | | int Landscape::SupplyJustSprayed | ( | int | *a\_polyref* | ) |  | | inline |

References m\_elems, and m\_polymapping.

{

return m\_elems[ m\_polymapping[ a\_polyref ] ]->IsRecentlySprayed();

}

|  |  |  |  |  |  |  |  |  |  |  |  |  |  |
| --- | --- | --- | --- | --- | --- | --- | --- | --- | --- | --- | --- | --- | --- |
| |  |  |  |  | | --- | --- | --- | --- | | int Landscape::SupplyJustSprayed | ( | int | *a\_x*, | |  |  | int | *a\_y* | |  | ) |  |  | | inline |

References RasterMap::Get(), m\_elems, and m\_land.

{

return m\_elems[ m\_land->Get( a\_x, a\_y ) ]->IsRecentlySprayed();

}

|  |  |  |  |  |  |  |  |
| --- | --- | --- | --- | --- | --- | --- | --- |
| |  |  |  |  |  |  | | --- | --- | --- | --- | --- | --- | | int Landscape::SupplyJustSprayedVector | ( | unsigned int | *a\_index* | ) |  | | inline |

References m\_elems.

{

return m\_elems[ a\_index ]->IsRecentlySprayed();

}

|  |  |  |  |  |  |  |  |
| --- | --- | --- | --- | --- | --- | --- | --- |
| |  |  |  |  |  |  | | --- | --- | --- | --- | --- | --- | | double Landscape::SupplyLAGreen | ( | int | *a\_polyref* | ) |  | | inline |

References m\_elems, and m\_polymapping.

{

return m\_elems[ m\_polymapping[ a\_polyref ]]->GetLAGreen();

}

|  |  |  |  |  |  |  |  |  |  |  |  |  |  |
| --- | --- | --- | --- | --- | --- | --- | --- | --- | --- | --- | --- | --- | --- |
| |  |  |  |  | | --- | --- | --- | --- | | double Landscape::SupplyLAGreen | ( | int | *a\_x*, | |  |  | int | *a\_y* | |  | ) |  |  | | inline |

References RasterMap::Get(), m\_elems, and m\_land.

{

return m\_elems[ m\_land->Get( a\_x, a\_y ) ]->GetLAGreen();

}

|  |  |  |  |  |  |  |
| --- | --- | --- | --- | --- | --- | --- |
| |  |  |  |  |  | | --- | --- | --- | --- | --- | | int Landscape::SupplyLargestPolyNumUsed | ( |  | ) |  | | inline |

References m\_LargestPolyNumUsed.

Referenced by SkTerritories::PreFillQualGrid().

{ return m\_LargestPolyNumUsed; }

|  |  |  |  |  |  |  |  |  |  |  |  |  |  |
| --- | --- | --- | --- | --- | --- | --- | --- | --- | --- | --- | --- | --- | --- |
| |  |  |  |  | | --- | --- | --- | --- | | int Landscape::SupplyLastTreatment | ( | int | *a\_polyref*, | |  |  | int \* | *a\_index* | |  | ) |  |  | | inline |

References m\_elems, and m\_polymapping.

Referenced by EventDump().

{

return m\_elems[ m\_polymapping[ a\_polyref ]]->GetLastTreatment( a\_index );

}

|  |  |  |  |  |  |  |  |  |  |  |  |  |  |  |  |  |  |
| --- | --- | --- | --- | --- | --- | --- | --- | --- | --- | --- | --- | --- | --- | --- | --- | --- | --- |
| |  |  |  |  | | --- | --- | --- | --- | | int Landscape::SupplyLastTreatment | ( | int | *a\_x*, | |  |  | int | *a\_y*, | |  |  | int \* | *a\_index* | |  | ) |  |  | | inline |

References RasterMap::Get(), m\_elems, and m\_land.

{

return m\_elems[ m\_land->Get( a\_x, a\_y ) ]->GetLastTreatment( a\_index );

}

|  |  |  |  |  |  |  |  |  |  |  |  |  |  |
| --- | --- | --- | --- | --- | --- | --- | --- | --- | --- | --- | --- | --- | --- |
| |  |  |  |  | | --- | --- | --- | --- | | double Landscape::SupplyLATotal | ( | int | *a\_x*, | |  |  | int | *a\_y* | |  | ) |  |  | | inline |

References RasterMap::Get(), m\_elems, and m\_land.

Referenced by VegDump().

{

return m\_elems[ m\_land->Get( a\_x, a\_y ) ]->GetLATotal();

}

|  |  |  |  |  |  |
| --- | --- | --- | --- | --- | --- |
| int Landscape::SupplyLECount | ( | void |  | ) |  |

References m\_elems.

Referenced by Landscape().

{

return (int) m\_elems.size();

}

|  |  |  |  |  |  |
| --- | --- | --- | --- | --- | --- |
| int Landscape::SupplyLENext | ( | void |  | ) |  |

References le\_signal\_index, and m\_elems.

Referenced by Landscape().

{

if ( ( unsigned int ) le\_signal\_index == m\_elems.size() ) {

return -1;

}

return m\_elems[ le\_signal\_index++ ]->GetPoly();

}

|  |  |  |  |  |  |  |  |
| --- | --- | --- | --- | --- | --- | --- | --- |
| |  |  |  |  |  |  | | --- | --- | --- | --- | --- | --- | | LE \* Landscape::SupplyLEPointer | ( | int | *a\_polyref* | ) |  | | inline |

References m\_elems, and m\_polymapping.

Referenced by Farm::AutumnHarrow(), Farm::AutumnPlough(), Farm::AutumnRoll(), Farm::AutumnSow(), Farm::BurnStrawStubble(), Farm::CattleIsOut(), Farm::CattleIsOutLow(), Farm::CattleOut(), Farm::CattleOutLowGrazing(), Farm::CutToHay(), Farm::CutToSilage(), Farm::CutWeeds(), Farm::DeepPlough(), Field::DoDevelopment(), Farm::FA\_AmmoniumSulphate(), Farm::FA\_GreenManure(), Farm::FA\_Manure(), Farm::FA\_NPK(), Farm::FA\_PK(), Farm::FA\_Sludge(), Farm::FA\_Slurry(), Farm::FP\_GreenManure(), Farm::FP\_LiquidNH3(), Farm::FP\_ManganeseSulphate(), Farm::FP\_Manure(), Farm::FP\_NPK(), Farm::FP\_NPKS(), Farm::FP\_PK(), Farm::FP\_Sludge(), Farm::FP\_Slurry(), Farm::Harvest(), Farm::HayBailing(), Farm::HayTurning(), Farm::HillingUp(), Farm::PigsAreOut(), Farm::PigsAreOutForced(), Farm::PigsOut(), Farm::RowCultivation(), Farm::SleepAllDay(), Farm::SpringHarrow(), Farm::SpringPlough(), Farm::SpringRoll(), Farm::SpringSow(), Farm::StrawChopping(), Farm::Strigling(), Farm::StriglingSow(), Farm::StubbleHarrowing(), Farm::Swathing(), UnsprayedMarginScan(), Farm::Water(), and Farm::WinterPlough().

{

return m\_elems[ m\_polymapping[ a\_polyref ]];

}

|  |  |  |  |  |  |
| --- | --- | --- | --- | --- | --- |
| void Landscape::SupplyLEReset | ( | void |  | ) |  |

References le\_signal\_index.

Referenced by Landscape().

{

le\_signal\_index = 0;

}

|  |  |  |  |  |  |
| --- | --- | --- | --- | --- | --- |
| LE\_Signal Landscape::SupplyLESignal | ( | int | *a\_polyref* | ) |  |

References m\_elems, and m\_polymapping.

Referenced by Landscape().

{

return m\_elems[ m\_polymapping[ a\_polyref ]]->GetSignal();

}

|  |  |  |  |  |  |  |  |  |  |  |  |  |  |
| --- | --- | --- | --- | --- | --- | --- | --- | --- | --- | --- | --- | --- | --- |
| |  |  |  |  | | --- | --- | --- | --- | | int \* Landscape::SupplyMagicMapP | ( | int | *a\_x*, | |  |  | int | *a\_y* | |  | ) |  |  | | inline |

References RasterMap::GetMagicP(), and m\_land.

{

return m\_land->GetMagicP( a\_x, a\_y );

}

|  |  |  |  |  |  |  |  |  |  |  |  |  |  |
| --- | --- | --- | --- | --- | --- | --- | --- | --- | --- | --- | --- | --- | --- |
| |  |  |  |  | | --- | --- | --- | --- | | double Landscape::SupplyMeanTemp | ( | long | *a\_date*, | |  |  | unsigned int | *a\_period* | |  | ) |  |  | | inline |

References g\_weather, and Weather::GetMeanTemp().

{

return g\_weather->GetMeanTemp( a\_date, a\_period );

}

|  |  |  |  |  |  |  |  |
| --- | --- | --- | --- | --- | --- | --- | --- |
| |  |  |  |  |  |  | | --- | --- | --- | --- | --- | --- | | int Landscape::SupplyMonth | ( | void |  | ) |  | | inline |

References g\_date, and Calendar::GetMonth().

Referenced by RunTheSim().

{

return g\_date->GetMonth();

}

|  |  |  |  |  |  |  |  |
| --- | --- | --- | --- | --- | --- | --- | --- |
| |  |  |  |  |  |  | | --- | --- | --- | --- | --- | --- | | unsigned int Landscape::SupplyNumberOfPolygons | ( | void |  | ) |  | | inline |

References m\_elems.

{

return (unsigned int) m\_elems.size();

}

|  |  |  |  |
| --- | --- | --- | --- |
| double Landscape::SupplyPesticide | ( | int | *a\_x*, |
|  |  | int | *a\_y* |
|  | ) |  |  |

References g\_pest, and Pesticide::SupplyPesticide().

Referenced by Skylark\_Male::GetFood().

{

double pp;

pp = g\_pest->SupplyPesticide( a\_x, a\_y );

return pp;

}

|  |  |  |  |  |  |
| --- | --- | --- | --- | --- | --- |
| double Landscape::SupplyPesticide | ( | int | *a\_polyref* | ) |  |

References g\_pest, Pesticide::SupplyPesticide(), and CfgBool::value().

{

if (!l\_pest\_enable\_pesticide\_engine.value()) return 0.0;

double pp;

pp = g\_pest->SupplyPesticide( a\_polyref );

return pp;

}

|  |  |  |  |  |  |  |  |
| --- | --- | --- | --- | --- | --- | --- | --- |
| |  |  |  |  |  |  | | --- | --- | --- | --- | --- | --- | | int Landscape::SupplyPesticideCell | ( | int | *a\_polyref* | ) |  | | inline |

References m\_elems, and m\_polymapping.

Referenced by Pesticide::SupplyPesticide().

{

return m\_elems[ m\_polymapping[ a\_polyref ] ]->GetPesticideCell();

}

|  |  |  |  |  |  |  |  |
| --- | --- | --- | --- | --- | --- | --- | --- |
| |  |  |  |  |  |  | | --- | --- | --- | --- | --- | --- | | TTypesOfPesticide Landscape::SupplyPesticideType | ( | void |  | ) |  | | inline |

References m\_PesticideType.

{ return m\_PesticideType; }

|  |  |  |  |  |  |  |  |
| --- | --- | --- | --- | --- | --- | --- | --- |
| |  |  |  |  |  |  | | --- | --- | --- | --- | --- | --- | | double Landscape::SupplyPolygonAreaVector | ( | int | *a\_polyref* | ) |  | | inline |

Returns the area of a polygon using the vector index as a reference.

References m\_elems.

{

return m\_elems[ a\_polyref ]->GetArea();

}

|  |  |  |  |  |  |  |  |  |  |  |  |  |  |
| --- | --- | --- | --- | --- | --- | --- | --- | --- | --- | --- | --- | --- | --- |
| |  |  |  |  | | --- | --- | --- | --- | | int Landscape::SupplyPolyRef | ( | int | *a\_x*, | |  |  | int | *a\_y* | |  | ) |  |  | | inline |

References RasterMap::Get(), m\_elems, and m\_land.

Referenced by AxisLoop(), CentroidSpiralOut(), ChangeMapMapping(), FindFieldCenter(), FindLongestAxis(), SkTerritories::PreEvaluateQualGrid(), TAnimal::SupplyPolygonRef(), and Pesticide::Test().

{

return m\_elems[ m\_land->Get( a\_x, a\_y )]->GetPoly();

}

|  |  |  |  |  |  |  |  |  |  |  |  |  |  |
| --- | --- | --- | --- | --- | --- | --- | --- | --- | --- | --- | --- | --- | --- |
| |  |  |  |  | | --- | --- | --- | --- | | int Landscape::SupplyPolyRefCC | ( | int | *a\_x*, | |  |  | int | *a\_y* | |  | ) |  |  | | inline |

References RasterMap::Get(), m\_elems, m\_height, m\_height10, m\_land, m\_width, and m\_width10.

{

a\_x = (a\_x + m\_width10) % m\_width;

a\_y = (a\_y + m\_height10) % m\_height;

return m\_elems[ m\_land->Get( a\_x, a\_y )]->GetPoly();

}

|  |  |  |  |  |  |  |  |  |  |  |  |  |  |
| --- | --- | --- | --- | --- | --- | --- | --- | --- | --- | --- | --- | --- | --- |
| |  |  |  |  | | --- | --- | --- | --- | | int Landscape::SupplyPolyRefIndex | ( | int | *a\_x*, | |  |  | int | *a\_y* | |  | ) |  |  | | inline |

References RasterMap::Get(), and m\_land.

{

return m\_land->Get( a\_x, a\_y );

}

|  |  |  |  |  |  |  |  |
| --- | --- | --- | --- | --- | --- | --- | --- |
| |  |  |  |  |  |  | | --- | --- | --- | --- | --- | --- | | int Landscape::SupplyPolyRefVector | ( | unsigned int | *a\_index* | ) |  | | inline |

References m\_elems.

{

return m\_elems[ a\_index ]->GetPoly();

}

|  |  |  |  |  |  |  |  |
| --- | --- | --- | --- | --- | --- | --- | --- |
| |  |  |  |  |  |  | | --- | --- | --- | --- | --- | --- | | double Landscape::SupplyRain | ( | void |  | ) |  | | inline |

References g\_weather, and Weather::GetRain().

Referenced by Skylark\_Female::FeedYoung(), Skylark\_Adult::GetWeatherHindrance(), Skylark\_Male::st\_CaringForYoung(), and Skylark\_Female::st\_Incubating().

{

return g\_weather->GetRain();

}

|  |  |  |  |  |  |  |  |
| --- | --- | --- | --- | --- | --- | --- | --- |
| |  |  |  |  |  |  | | --- | --- | --- | --- | --- | --- | | double Landscape::SupplyRain | ( | long | *a\_date* | ) |  | | inline |

References g\_weather, and Weather::GetRain().

{

return g\_weather->GetRain( a\_date );

}

|  |  |  |  |  |  |  |  |  |  |  |  |  |  |
| --- | --- | --- | --- | --- | --- | --- | --- | --- | --- | --- | --- | --- | --- |
| |  |  |  |  | | --- | --- | --- | --- | | double Landscape::SupplyRainPeriod | ( | long | *a\_date*, | |  |  | int | *a\_period* | |  | ) |  |  | | inline |

References g\_weather, and Weather::GetRainPeriod().

{

return g\_weather->GetRainPeriod( a\_date, a\_period );

}

|  |  |  |  |  |  |  |  |  |  |  |  |  |  |
| --- | --- | --- | --- | --- | --- | --- | --- | --- | --- | --- | --- | --- | --- |
| |  |  |  |  | | --- | --- | --- | --- | | int Landscape::SupplyRoadWidth | ( | int | , | |  |  | int |  | |  | ) |  |  | | inline |

{return 0;}

|  |  |  |  |
| --- | --- | --- | --- |
| double Landscape::SupplyRodenticide | ( | int | *a\_x*, |
|  |  | int | *a\_y* |
|  | ) |  |  |

References cfg\_rodenticide\_enable, RodenticideManager::GetRodenticide(), m\_RodenticideManager, and CfgBool::value().

{

if (cfg\_rodenticide\_enable.value())

{

double pp;

pp = m\_RodenticideManager->GetRodenticide( a\_x, a\_y );

return pp;

}

return 0;

}

|  |  |  |  |  |  |  |
| --- | --- | --- | --- | --- | --- | --- |
| |  |  |  |  |  | | --- | --- | --- | --- | --- | | bool Landscape::SupplyShouldSpray | ( |  | ) |  | | inline |

References m\_toxShouldSpray.

Referenced by Orchard::DoDevelopment(), and OrchardBand::DoDevelopment().

{return m\_toxShouldSpray;}

|  |  |  |  |  |  |  |  |
| --- | --- | --- | --- | --- | --- | --- | --- |
| |  |  |  |  |  |  | | --- | --- | --- | --- | --- | --- | | int Landscape::SupplySimAreaHeight | ( | void |  | ) |  | | inline |

References m\_land, and RasterMap::MapHeight().

Referenced by TAnimal::CorrectWrapRound(), SkTerritories::DumpMapGraphics(), IDMap::IDMap(), MovementMap::Init(), MovementMap16::Init(), Skylark\_Population\_Manager::Init(), MovementMap::MovementMap(), MovementMap16::MovementMap16(), PositionMap::PositionMap(), ScalablePositionMap::ScalablePositionMap(), SimplePositionMap::SimplePositionMap(), SkTerritories::SkTerritories(), and Skylark\_Female::st\_Arriving().

{

return m\_land->MapHeight();

}

|  |  |  |  |  |  |  |  |
| --- | --- | --- | --- | --- | --- | --- | --- |
| |  |  |  |  |  |  | | --- | --- | --- | --- | --- | --- | | int Landscape::SupplySimAreaWidth | ( | void |  | ) |  | | inline |

References m\_land, and RasterMap::MapWidth().

Referenced by TAnimal::CorrectWrapRound(), SkTerritories::DumpMapGraphics(), IDMap::IDMap(), MovementMap::Init(), MovementMap16::Init(), Skylark\_Population\_Manager::Init(), MovementMap::MovementMap(), MovementMap16::MovementMap16(), PositionMap::PositionMap(), RodenticideManager::RodenticideManager(), ScalablePositionMap::ScalablePositionMap(), SimplePositionMap::SimplePositionMap(), SkTerritories::SkTerritories(), Skylark\_Female::st\_Arriving(), skTTerritory::TestNestPossibility(), and Skylark\_Population\_Manager::TheRipleysOutputProbe().

{

return m\_land->MapWidth();

}

|  |  |  |  |  |  |  |  |
| --- | --- | --- | --- | --- | --- | --- | --- |
| |  |  |  |  |  |  | | --- | --- | --- | --- | --- | --- | | bool Landscape::SupplySkScrapes | ( | int | *a\_polyref* | ) |  | | inline |

References m\_elems, and m\_polymapping.

Referenced by Skylark\_Adult::GetVegHindrance(), and SkTerritories::PrePolyNQual().

{

return m\_elems[ m\_polymapping[ a\_polyref ]]->GetSkScrapes();

}

|  |  |  |  |  |  |  |  |
| --- | --- | --- | --- | --- | --- | --- | --- |
| |  |  |  |  |  |  | | --- | --- | --- | --- | --- | --- | | bool Landscape::SupplySnowcover | ( | void |  | ) |  | | inline |

References g\_weather, and Weather::GetSnow().

Referenced by Skylark\_Female::st\_PreparingForBreeding().

{

return g\_weather->GetSnow();

}

|  |  |  |  |  |  |  |  |
| --- | --- | --- | --- | --- | --- | --- | --- |
| |  |  |  |  |  |  | | --- | --- | --- | --- | --- | --- | | bool Landscape::SupplySnowcover | ( | long | *a\_date* | ) |  | | inline |

References g\_weather, and Weather::GetSnow().

{

return g\_weather->GetSnow( a\_date );

}

|  |  |  |  |  |  |  |  |
| --- | --- | --- | --- | --- | --- | --- | --- |
| |  |  |  |  |  |  | | --- | --- | --- | --- | --- | --- | | double Landscape::SupplyTemp | ( | void |  | ) |  | | inline |

References g\_weather, and Weather::GetTemp().

Referenced by Skylark\_Population\_Manager::DoFirst(), Skylark\_Female::FeedYoung(), Skylark\_Adult::GetWeatherHindrance(), VegElement::RecalculateBugsNStuff(), Skylark\_Female::st\_BuildingUpResources(), Skylark\_Clutch::st\_Developing(), Skylark\_Nestling::st\_Developing(), Skylark\_Female::st\_Emigrating(), Skylark\_Male::st\_Emigrating(), and Skylark\_Female::st\_PreparingForBreeding().

{

return g\_weather->GetTemp();

}

|  |  |  |  |  |  |  |  |
| --- | --- | --- | --- | --- | --- | --- | --- |
| |  |  |  |  |  |  | | --- | --- | --- | --- | --- | --- | | double Landscape::SupplyTemp | ( | long | *a\_date* | ) |  | | inline |

References g\_weather, and Weather::GetTemp().

{

return g\_weather->GetTemp( a\_date );

}

|  |  |  |  |  |  |  |  |  |  |  |  |  |  |
| --- | --- | --- | --- | --- | --- | --- | --- | --- | --- | --- | --- | --- | --- |
| |  |  |  |  | | --- | --- | --- | --- | | double Landscape::SupplyTempPeriod | ( | long | *a\_date*, | |  |  | int | *a\_period* | |  | ) |  |  | | inline |

References g\_weather, and Weather::GetTempPeriod().

{

return g\_weather->GetTempPeriod( a\_date, a\_period );

}

|  |  |  |  |  |  |  |  |  |  |  |  |  |  |
| --- | --- | --- | --- | --- | --- | --- | --- | --- | --- | --- | --- | --- | --- |
| |  |  |  |  | | --- | --- | --- | --- | | double Landscape::SupplyTrafficLoad | ( | int | *a\_x*, | |  |  | int | *a\_y* | |  | ) |  |  | | inline |

References RasterMap::Get(), m\_elems, and m\_land.

{

return m\_elems[ m\_land->Get( a\_x, a\_y ) ]->GetTrafficLoad();

}

|  |  |  |  |  |  |  |  |
| --- | --- | --- | --- | --- | --- | --- | --- |
| |  |  |  |  |  |  | | --- | --- | --- | --- | --- | --- | | double Landscape::SupplyTrafficLoad | ( | int | *a\_polyref* | ) |  | | inline |

References m\_elems, and m\_polymapping.

{

return m\_elems[ m\_polymapping[ a\_polyref ] ]->GetTrafficLoad();

}

|  |  |  |  |  |  |  |  |
| --- | --- | --- | --- | --- | --- | --- | --- |
| |  |  |  |  |  |  | | --- | --- | --- | --- | --- | --- | | int Landscape::SupplyTreeAge | ( | int | *a\_Polyref* | ) |  | | inline |

{

return 1;

}

|  |  |  |  |  |  |  |  |  |  |  |  |  |  |
| --- | --- | --- | --- | --- | --- | --- | --- | --- | --- | --- | --- | --- | --- |
| |  |  |  |  | | --- | --- | --- | --- | | int Landscape::SupplyTreeAge | ( | int | , | |  |  | int |  | |  | ) |  |  | | inline |

{return 0;}

|  |  |  |  |  |  |  |  |  |  |  |  |  |  |
| --- | --- | --- | --- | --- | --- | --- | --- | --- | --- | --- | --- | --- | --- |
| |  |  |  |  | | --- | --- | --- | --- | | int Landscape::SupplyTreeHeight | ( | int | , | |  |  | int |  | |  | ) |  |  | | inline |

{return 0;}

|  |  |  |  |  |  |  |  |
| --- | --- | --- | --- | --- | --- | --- | --- |
| |  |  |  |  |  |  | | --- | --- | --- | --- | --- | --- | | int Landscape::SupplyTreeHeight | ( | int |  | ) |  | | inline |

{return 0;}

|  |  |  |  |  |  |  |  |  |  |  |  |  |  |
| --- | --- | --- | --- | --- | --- | --- | --- | --- | --- | --- | --- | --- | --- |
| |  |  |  |  | | --- | --- | --- | --- | | int Landscape::SupplyUnderGrowthWidth | ( | int | , | |  |  | int |  | |  | ) |  |  | | inline |

{return 0;}

|  |  |  |  |  |  |  |  |
| --- | --- | --- | --- | --- | --- | --- | --- |
| |  |  |  |  |  |  | | --- | --- | --- | --- | --- | --- | | int Landscape::SupplyUnderGrowthWidth | ( | int |  | ) |  | | inline |

{return 0;}

|  |  |  |  |  |  |  |  |
| --- | --- | --- | --- | --- | --- | --- | --- |
| |  |  |  |  |  |  | | --- | --- | --- | --- | --- | --- | | int Landscape::SupplyValidX | ( | int | *a\_polyref* | ) |  | | inline |

References m\_elems, and m\_polymapping.

{

return m\_elems[ m\_polymapping[ a\_polyref ] ]->GetValidX();

}

|  |  |  |  |  |  |  |  |
| --- | --- | --- | --- | --- | --- | --- | --- |
| |  |  |  |  |  |  | | --- | --- | --- | --- | --- | --- | | int Landscape::SupplyValidY | ( | int | *a\_polyref* | ) |  | | inline |

References m\_elems, and m\_polymapping.

{

return m\_elems[ m\_polymapping[ a\_polyref ] ]->GetValidY();

}

|  |  |  |  |  |  |  |  |
| --- | --- | --- | --- | --- | --- | --- | --- |
| |  |  |  |  |  |  | | --- | --- | --- | --- | --- | --- | | int Landscape::SupplyVegAge | ( | int | *a\_Polyref* | ) |  | | inline |

References m\_elems, and m\_polymapping.

{

return m\_elems[ m\_polymapping[ a\_polyref ]]->GetVegAge();

}

|  |  |  |  |  |  |  |  |  |  |  |  |  |  |
| --- | --- | --- | --- | --- | --- | --- | --- | --- | --- | --- | --- | --- | --- |
| |  |  |  |  | | --- | --- | --- | --- | | int Landscape::SupplyVegAge | ( | int | *a\_x*, | |  |  | int | *a\_y* | |  | ) |  |  | | inline |

References RasterMap::Get(), m\_elems, and m\_land.

{

return m\_elems[ m\_land->Get( a\_x, a\_y ) ]->GetVegAge();

}

|  |  |  |  |  |  |  |  |
| --- | --- | --- | --- | --- | --- | --- | --- |
| |  |  |  |  |  |  | | --- | --- | --- | --- | --- | --- | | double Landscape::SupplyVegBiomass | ( | int | *a\_polyref* | ) |  | | inline |

References m\_elems, and m\_polymapping.

Referenced by VegDump().

{

return m\_elems[ m\_polymapping[ a\_polyref ]]->GetVegBiomass();

}

|  |  |  |  |  |  |  |  |  |  |  |  |  |  |
| --- | --- | --- | --- | --- | --- | --- | --- | --- | --- | --- | --- | --- | --- |
| |  |  |  |  | | --- | --- | --- | --- | | double Landscape::SupplyVegBiomass | ( | int | *a\_x*, | |  |  | int | *a\_y* | |  | ) |  |  | | inline |

References RasterMap::Get(), m\_elems, and m\_land.

{

return m\_elems[ m\_land->Get( a\_x, a\_y ) ]->GetVegBiomass();

}

|  |  |  |  |  |  |  |  |
| --- | --- | --- | --- | --- | --- | --- | --- |
| |  |  |  |  |  |  | | --- | --- | --- | --- | --- | --- | | double Landscape::SupplyVegBiomassVector | ( | unsigned int | *a\_index* | ) |  | | inline |

References m\_elems.

{

return m\_elems[ a\_index ]->GetVegBiomass();

}

|  |  |  |  |  |  |  |  |
| --- | --- | --- | --- | --- | --- | --- | --- |
| |  |  |  |  |  |  | | --- | --- | --- | --- | --- | --- | | double Landscape::SupplyVegCover | ( | int | *a\_polyref* | ) |  | | inline |

References m\_elems, and m\_polymapping.

Referenced by VegDump().

{

return m\_elems[ m\_polymapping[ a\_polyref ]]->GetVegCover();

}

|  |  |  |  |  |  |  |  |  |  |  |  |  |  |
| --- | --- | --- | --- | --- | --- | --- | --- | --- | --- | --- | --- | --- | --- |
| |  |  |  |  | | --- | --- | --- | --- | | double Landscape::SupplyVegCover | ( | int | *a\_x*, | |  |  | int | *a\_y* | |  | ) |  |  | | inline |

References RasterMap::Get(), m\_elems, and m\_land.

{

return m\_elems[ m\_land->Get( a\_x, a\_y ) ]->GetVegCover();

}

|  |  |  |  |  |  |  |  |
| --- | --- | --- | --- | --- | --- | --- | --- |
| |  |  |  |  |  |  | | --- | --- | --- | --- | --- | --- | | double Landscape::SupplyVegCoverVector | ( | unsigned int | *a\_index* | ) |  | | inline |

References m\_elems.

{

return m\_elems[ a\_index ]->GetVegCover();

}

|  |  |  |  |  |  |  |  |
| --- | --- | --- | --- | --- | --- | --- | --- |
| |  |  |  |  |  |  | | --- | --- | --- | --- | --- | --- | | int Landscape::SupplyVegDensity | ( | int | *a\_polyref* | ) |  | | inline |

References m\_elems, and m\_polymapping.

Referenced by Skylark\_Adult::GetVegHindrance(), and SkTerritories::PrePolyNQual().

{

return m\_elems[ m\_polymapping[ a\_polyref ]]->GetVegDensity();

}

|  |  |  |  |  |  |  |  |  |  |  |  |  |  |
| --- | --- | --- | --- | --- | --- | --- | --- | --- | --- | --- | --- | --- | --- |
| |  |  |  |  | | --- | --- | --- | --- | | int Landscape::SupplyVegDensity | ( | int | *a\_x*, | |  |  | int | *a\_y* | |  | ) |  |  | | inline |

References RasterMap::Get(), m\_elems, and m\_land.

{

return m\_elems[ m\_land->Get( a\_x, a\_y ) ]->GetVegDensity();

}

|  |  |  |  |  |  |  |  |
| --- | --- | --- | --- | --- | --- | --- | --- |
| |  |  |  |  |  |  | | --- | --- | --- | --- | --- | --- | | double Landscape::SupplyVegDigestability | ( | int | *a\_polyref* | ) |  | | inline |

References m\_elems, and m\_polymapping.

Referenced by VegDump().

{

return m\_elems[ m\_polymapping[ a\_polyref ]]->GetDigestability();

}

|  |  |  |  |  |  |  |  |  |  |  |  |  |  |
| --- | --- | --- | --- | --- | --- | --- | --- | --- | --- | --- | --- | --- | --- |
| |  |  |  |  | | --- | --- | --- | --- | | double Landscape::SupplyVegDigestability | ( | int | *a\_x*, | |  |  | int | *a\_y* | |  | ) |  |  | | inline |

References RasterMap::Get(), m\_elems, and m\_land.

{

return m\_elems[ m\_land->Get( a\_x, a\_y ) ]->GetDigestability();

}

|  |  |  |  |  |  |  |  |
| --- | --- | --- | --- | --- | --- | --- | --- |
| |  |  |  |  |  |  | | --- | --- | --- | --- | --- | --- | | double Landscape::SupplyVegDigestabilityVector | ( | unsigned int | *a\_index* | ) |  | | inline |

References m\_elems.

{

return m\_elems[ a\_index ]->GetDigestability();

}

|  |  |  |  |  |  |  |  |
| --- | --- | --- | --- | --- | --- | --- | --- |
| |  |  |  |  |  |  | | --- | --- | --- | --- | --- | --- | | double Landscape::SupplyVegHeight | ( | int | *a\_polyref* | ) |  | | inline |

References m\_elems, and m\_polymapping.

Referenced by DumpMapGraphics(), SkTerritories::DumpMapGraphics(), Skylark\_Adult::GetVegHindrance(), SkTerritories::PrePoly2Qual(), SkTerritories::PrePolyNQual(), and VegDump().

{

return m\_elems[ m\_polymapping[ a\_polyref ]]->GetVegHeight();

}

|  |  |  |  |  |  |  |  |  |  |  |  |  |  |
| --- | --- | --- | --- | --- | --- | --- | --- | --- | --- | --- | --- | --- | --- |
| |  |  |  |  | | --- | --- | --- | --- | | double Landscape::SupplyVegHeight | ( | int | *a\_x*, | |  |  | int | *a\_y* | |  | ) |  |  | | inline |

References RasterMap::Get(), m\_elems, and m\_land.

{

return m\_elems[ m\_land->Get( a\_x, a\_y ) ]->GetVegHeight();

}

|  |  |  |  |  |  |  |  |
| --- | --- | --- | --- | --- | --- | --- | --- |
| |  |  |  |  |  |  | | --- | --- | --- | --- | --- | --- | | double Landscape::SupplyVegHeightVector | ( | unsigned int | *a\_index* | ) |  | | inline |

References m\_elems.

{

return m\_elems[ a\_index ]->GetVegHeight();

}

|  |  |  |  |  |  |  |  |
| --- | --- | --- | --- | --- | --- | --- | --- |
| |  |  |  |  |  |  | | --- | --- | --- | --- | --- | --- | | bool Landscape::SupplyVegPatchy | ( | int | *a\_polyref* | ) |  | | inline |

References m\_elems, and m\_polymapping.

Referenced by Skylark\_Adult::GetVegHindrance(), SkTerritories::PrePoly2Qual(), and SkTerritories::PrePolyNQual().

{

return m\_elems[ m\_polymapping[ a\_polyref ]]->GetVegPatchy();

}

|  |  |  |  |  |  |  |  |  |  |  |  |  |  |
| --- | --- | --- | --- | --- | --- | --- | --- | --- | --- | --- | --- | --- | --- |
| |  |  |  |  | | --- | --- | --- | --- | | bool Landscape::SupplyVegPatchy | ( | int | *a\_x*, | |  |  | int | *a\_y* | |  | ) |  |  | | inline |

References RasterMap::Get(), m\_elems, and m\_land.

{

return m\_elems[ m\_land->Get( a\_x, a\_y ) ]->GetVegPatchy();

}

|  |  |  |  |  |  |  |  |  |  |  |  |  |  |
| --- | --- | --- | --- | --- | --- | --- | --- | --- | --- | --- | --- | --- | --- |
| |  |  |  |  | | --- | --- | --- | --- | | TTypesOfVegetation Landscape::SupplyVegType | ( | int | *a\_x*, | |  |  | int | *a\_y* | |  | ) |  |  | | inline |

References RasterMap::Get(), m\_elems, and m\_land.

Referenced by Skylark\_Female::st\_Laying(), and VegDump().

{

return m\_elems[ m\_land->Get( a\_x, a\_y ) ]->GetVegType();

}

|  |  |  |  |  |  |  |  |
| --- | --- | --- | --- | --- | --- | --- | --- |
| |  |  |  |  |  |  | | --- | --- | --- | --- | --- | --- | | TTypesOfVegetation Landscape::SupplyVegType | ( | int | *polyref* | ) |  | | inline |

References m\_elems, and m\_polymapping.

{

return m\_elems[ m\_polymapping[ a\_polyref ] ]->GetVegType();

}

|  |  |  |  |  |  |  |  |
| --- | --- | --- | --- | --- | --- | --- | --- |
| |  |  |  |  |  |  | | --- | --- | --- | --- | --- | --- | | TTypesOfVegetation Landscape::SupplyVegTypeVector | ( | unsigned int | *a\_index* | ) |  | | inline |

References m\_elems.

{

return m\_elems[ a\_index ]->GetVegType();

}

|  |  |  |  |  |  |  |  |
| --- | --- | --- | --- | --- | --- | --- | --- |
| |  |  |  |  |  |  | | --- | --- | --- | --- | --- | --- | | const char\* Landscape::SupplyVersion | ( | void |  | ) |  | | inline |

References m\_versioninfo.

{ return m\_versioninfo; }

|  |  |  |  |  |  |  |  |
| --- | --- | --- | --- | --- | --- | --- | --- |
| |  |  |  |  |  |  | | --- | --- | --- | --- | --- | --- | | double Landscape::SupplyWeedBiomass | ( | int | *a\_polyref* | ) |  | | inline |

References m\_elems, and m\_polymapping.

Referenced by VegDump().

{

return m\_elems[ m\_polymapping[ a\_polyref ]]->GetWeedBiomass();

}

|  |  |  |  |  |  |  |  |  |  |  |  |  |  |
| --- | --- | --- | --- | --- | --- | --- | --- | --- | --- | --- | --- | --- | --- |
| |  |  |  |  | | --- | --- | --- | --- | | double Landscape::SupplyWeedBiomass | ( | int | *a\_x*, | |  |  | int | *a\_y* | |  | ) |  |  | | inline |

References RasterMap::Get(), m\_elems, and m\_land.

{

return m\_elems[ m\_land->Get( a\_x, a\_y ) ]->GetWeedBiomass();

}

|  |  |  |  |  |  |  |  |
| --- | --- | --- | --- | --- | --- | --- | --- |
| |  |  |  |  |  |  | | --- | --- | --- | --- | --- | --- | | double Landscape::SupplyWind | ( | void |  | ) |  | | inline |

References g\_weather, and Weather::GetWind().

{

return g\_weather->GetWind();

}

|  |  |  |  |  |  |  |  |
| --- | --- | --- | --- | --- | --- | --- | --- |
| |  |  |  |  |  |  | | --- | --- | --- | --- | --- | --- | | double Landscape::SupplyWind | ( | long | *a\_date* | ) |  | | inline |

References g\_weather, and Weather::GetWind().

{

return g\_weather->GetWind( a\_date );

}

|  |  |  |  |  |  |  |  |
| --- | --- | --- | --- | --- | --- | --- | --- |
| |  |  |  |  |  |  | | --- | --- | --- | --- | --- | --- | | int Landscape::SupplyWindDirection | ( | void |  | ) |  | | inline |

References g\_date, Calendar::GetMonth(), random(), and WindDirections.

{

return WindDirections[g\_date->GetMonth()][random(100)];

}

|  |  |  |  |  |  |  |  |  |  |  |  |  |  |
| --- | --- | --- | --- | --- | --- | --- | --- | --- | --- | --- | --- | --- | --- |
| |  |  |  |  | | --- | --- | --- | --- | | double Landscape::SupplyWindPeriod | ( | long | *a\_date*, | |  |  | int | *a\_period* | |  | ) |  |  | | inline |

References g\_weather, and Weather::GetWindPeriod().

{

return g\_weather->GetWindPeriod( a\_date, a\_period );

}

|  |  |  |  |  |  |  |  |
| --- | --- | --- | --- | --- | --- | --- | --- |
| |  |  |  |  |  |  | | --- | --- | --- | --- | --- | --- | | int Landscape::SupplyYear | ( | void |  | ) |  | | inline |

References g\_date, and Calendar::GetYear().

{

return g\_date->GetYear();

}

|  |  |  |  |  |  |  |  |
| --- | --- | --- | --- | --- | --- | --- | --- |
| |  |  |  |  |  |  | | --- | --- | --- | --- | --- | --- | | int Landscape::SupplyYearNumber | ( | void |  | ) |  | | inline |

References g\_date, and Calendar::GetYearNumber().

Referenced by Skylark\_Population\_Manager::Catastrophe(), and Tick().

{

return g\_date->GetYearNumber();

}

|  |  |  |  |  |  |  |  |
| --- | --- | --- | --- | --- | --- | --- | --- |
| |  |  |  |  |  |  | | --- | --- | --- | --- | --- | --- | | void Landscape::TestCropManagement | ( | void |  | ) |  | | protected |

|  |  |  |  |  |  |
| --- | --- | --- | --- | --- | --- |
| void Landscape::Tick | ( | void |  | ) |  |

References cfg\_productapplicendyear, cfg\_productapplicstartyear, cfg\_rodenticide\_enable, Calendar::DayInYear(), EventDump(), FarmManager::FarmManagement(), g\_date, g\_pest, g\_weather, janfirst, Calendar::JanFirst(), l\_map\_dump\_event\_enable, l\_map\_dump\_event\_x1, l\_map\_dump\_event\_x2, l\_map\_dump\_event\_y1, l\_map\_dump\_event\_y2, l\_map\_dump\_veg\_enable, l\_map\_dump\_veg\_x, l\_map\_dump\_veg\_y, m\_elems, m\_FarmManager, m\_RodenticideManager, m\_toxShouldSpray, marchfirst, Calendar::MarchFirst(), SupplyYearNumber(), Calendar::Tick(), RodenticideManager::Tick(), Pesticide::Tick(), Weather::Tick(), CfgInt::value(), CfgBool::value(), and VegDump().

Referenced by Landscape(), TickHour(), TickMinute(), and TurnTheWorld().

{

g\_date->Tick();

g\_weather->Tick();

// Remember todays LAItotal for veg elements

for ( unsigned int i = 0; i < m\_elems.size(); i++ ) {

m\_elems[ i ]->StoreLAItotal( );

}

// Update the growth curve phases if needed.

if ( g\_date->JanFirst() ) {

for ( unsigned int i = 0; i < m\_elems.size(); i++ ) {

m\_elems[ i ]->SetGrowthPhase( janfirst );

}

} else if ( g\_date->MarchFirst() ) {

for ( unsigned int i = 0; i < m\_elems.size(); i++ ) {

m\_elems[ i ]->SetGrowthPhase( marchfirst );

}

// Check and see if the pesticide engine flag should be set

if (( SupplyYearNumber() >= cfg\_productapplicstartyear.value() ) && (SupplyYearNumber() <= cfg\_productapplicendyear.value()))

m\_toxShouldSpray = true;

else m\_toxShouldSpray = false;

}

// Grow the green stuff and let the bugs have some too.

for ( unsigned int i = 0; i < m\_elems.size(); i++ ) {

m\_elems[ i ]->Tick();

m\_elems[ i ]->DoDevelopment();

}

if ( g\_date->DayInYear() == g\_date->DayInYear( 1, 11 ) ) {

// Set all elements to smooth curve transition mode at November 1st.

for ( unsigned int i = 0; i < m\_elems.size(); i++ ) {

m\_elems[ i ]->ForceGrowthInitialize();

}

}

// Put the farmers to work.

m\_FarmManager->FarmManagement();

// Update pesticide information.

g\_pest->Tick();

// Update rodenticide information if we are using this

if (cfg\_rodenticide\_enable.value()) m\_RodenticideManager->Tick();

// Dump veg information if necessary

if ( l\_map\_dump\_veg\_enable.value() ) VegDump( l\_map\_dump\_veg\_x.value(), l\_map\_dump\_veg\_y.value() );

if ( l\_map\_dump\_event\_enable.value() )

EventDump( l\_map\_dump\_event\_x1.value(), l\_map\_dump\_event\_y1.value(), l\_map\_dump\_event\_x2.value(),

l\_map\_dump\_event\_y2.value() );

}

|  |  |  |  |  |  |  |  |
| --- | --- | --- | --- | --- | --- | --- | --- |
| |  |  |  |  |  |  | | --- | --- | --- | --- | --- | --- | | void Landscape::TickHour | ( | void |  | ) |  | | inline |

References g\_date, Tick(), and Calendar::TickHour().

{

if ( g\_date->TickHour() )

Tick();

}

|  |  |  |  |  |  |  |  |
| --- | --- | --- | --- | --- | --- | --- | --- |
| |  |  |  |  |  |  | | --- | --- | --- | --- | --- | --- | | void Landscape::TickMinute | ( | void |  | ) |  | | inline |

References g\_date, Tick(), and Calendar::TickMinute().

{

if ( g\_date->TickMinute() )

Tick();

}

|  |  |  |  |  |  |  |  |
| --- | --- | --- | --- | --- | --- | --- | --- |
| |  |  |  |  |  |  | | --- | --- | --- | --- | --- | --- | | TTypesOfLandscapeElement Landscape::TranslateEleTypes | ( | int | *EleReference* | ) |  | | inline |

References g\_letype, and LE\_TypeClass::TranslateEleTypes().

{

return g\_letype->TranslateEleTypes( EleReference );

}

|  |  |  |  |  |  |  |  |
| --- | --- | --- | --- | --- | --- | --- | --- |
| |  |  |  |  |  |  | | --- | --- | --- | --- | --- | --- | | TTypesOfVegetation Landscape::TranslateVegTypes | ( | int | *VegReference* | ) |  | | inline |

References g\_letype, and LE\_TypeClass::TranslateVegTypes().

{

return g\_letype->TranslateVegTypes( VegReference );

}

|  |  |  |  |  |  |  |  |
| --- | --- | --- | --- | --- | --- | --- | --- |
| |  |  |  |  |  |  | | --- | --- | --- | --- | --- | --- | | void Landscape::TurnTheWorld | ( | void |  | ) |  | | inline |

References Tick().

Referenced by RunTheSim().

{

Tick();

}

|  |  |  |  |  |  |  |  |  |  |  |  |  |  |  |  |  |  |  |  |  |  |  |  |  |  |
| --- | --- | --- | --- | --- | --- | --- | --- | --- | --- | --- | --- | --- | --- | --- | --- | --- | --- | --- | --- | --- | --- | --- | --- | --- | --- |
| |  |  |  |  | | --- | --- | --- | --- | | bool Landscape::UMarginTest | ( | int | *a\_fieldpoly*, | |  |  | int | *a\_borderpoly*, | |  |  | int | *a\_x*, | |  |  | int | *a\_y*, | |  |  | int | *a\_width* | |  | ) |  |  | | protected |

References RasterMap::Get(), m\_land, RasterMap::MapHeight(), and RasterMap::MapWidth().

Referenced by UnsprayedMarginScan().

{

int index;

int x\_add[ 8 ] = { 1\*a\_width, 1\*a\_width, 0, -1\*a\_width, -1\*a\_width, -1\*a\_width, 0, 1\*a\_width };

int y\_add[ 8 ] = { 0, -1\*a\_width, -1\*a\_width, -1\*a\_width, 0, 1\*a\_width, 1\*a\_width, 1\*a\_width };

int width = m\_land->MapWidth();

int height = m\_land->MapHeight();

// Scan anti-clockwise from center pixel coordinate.

for ( unsigned int i = 0; i < 8; i++ ) {

if ( ( a\_x + x\_add[ i ] >= width ) || ( a\_x + x\_add[ i ] < 0 ) || ( a\_y + y\_add[ i ] >= height )

|| ( a\_y + y\_add[ i ] < 0 ) ) {

return true;

}

//continue;

index = m\_land->Get( a\_x + x\_add[ i ], a\_y + y\_add[ i ] );

if ( ( index != a\_fieldindex ) && ( index != a\_marginindex ) ) return true;

}

return false;

}

|  |  |  |  |  |  |  |  |
| --- | --- | --- | --- | --- | --- | --- | --- |
| |  |  |  |  |  |  | | --- | --- | --- | --- | --- | --- | | void Landscape::UnsprayedMarginAdd | ( | LE \* | *a\_field* | ) |  | | protected |

References g\_msg, LE::GetValidX(), LE::GetValidY(), hb\_first\_free\_poly\_num, l\_map\_umargin\_width, m\_elems, m\_polymapping, NewElement(), LE::SetArea(), LE::SetPoly(), LE::SetUnsprayedMarginPolyRef(), tole\_UnsprayedFieldMargin, UnsprayedMarginScan(), CfgInt::value(), MapErrorMsg::Warn(), and WARN\_BUG.

Referenced by Landscape().

{

int x = a\_field->GetValidX();

int y = a\_field->GetValidY();

if ( ( x == -1 ) || ( y == -1 ) ) {

// Tripping this probably means it is not a field

g\_msg->Warn( WARN\_BUG, "Landscape::UnsprayedMarginAdd(): Uninitialized border coordinate!", "" );

exit( 1 );

}

LE \* umargin = NewElement( tole\_UnsprayedFieldMargin );

m\_polymapping[ hb\_first\_free\_poly\_num ] = (int) m\_elems.size();

m\_elems.resize( m\_elems.size() + 1 );

m\_elems[ m\_elems.size() - 1 ] = umargin;

a\_field->SetUnsprayedMarginPolyRef( hb\_first\_free\_poly\_num );

umargin->SetPoly( hb\_first\_free\_poly\_num++ );

umargin->SetArea( 0.0 );

for ( int q = 0; q < l\_map\_umargin\_width.value(); q++ )

UnsprayedMarginScan( a\_field, q + 1 );

}

|  |  |  |  |  |  |  |  |  |  |  |  |  |  |
| --- | --- | --- | --- | --- | --- | --- | --- | --- | --- | --- | --- | --- | --- |
| |  |  |  |  | | --- | --- | --- | --- | | void Landscape::UnsprayedMarginScan | ( | LE \* | *a\_field*, | |  |  | int | *a\_width* | |  | ) |  |  | | protected |

References LE::AddArea(), BorderStep(), FindValidXY(), LE::GetPoly(), LE::GetUnsprayedMarginPolyRef(), LE::GetValidX(), LE::GetValidY(), m\_land, m\_polymapping, RasterMap::Put(), SupplyLEPointer(), and UMarginTest().

Referenced by UnsprayedMarginAdd().

{

LE \* umargin = g\_landscape\_p->SupplyLEPointer( a\_field->GetUnsprayedMarginPolyRef() );

int fieldpoly = a\_field->GetPoly();

int borderpoly = umargin->GetPoly();

int borderindex = m\_polymapping[ borderpoly ];

int fieldindex = m\_polymapping[ fieldpoly ];

int notforever = 5000;

// These two will be modified through pointer operations

// in BorderStep().

int x = a\_field->GetValidX();

int y = a\_field->GetValidY();

// Now the problem is that GetValid does not always return a valid co-ord!

// so we need to search for one

if ( !FindValidXY( fieldindex, x, y ) ) return;

while ( --notforever ) {

// Check if this position should be made into a border.

if ( UMarginTest( fieldindex, borderindex, x, y, a\_width ) ) {

// Add this pixel to the border element in the big map.

m\_land->Put( x, y, borderindex );

a\_field->AddArea( -1.0 );

umargin->AddArea( 1.0 );

};

// Step to next coordinate. Quit when done.

if ( !BorderStep( fieldindex, borderindex, & x, & y ) )

return;

}

}

|  |  |  |  |  |  |  |  |  |  |  |  |  |  |
| --- | --- | --- | --- | --- | --- | --- | --- | --- | --- | --- | --- | --- | --- |
| |  |  |  |  | | --- | --- | --- | --- | | void Landscape::VegDump | ( | int | *x*, | |  |  | int | *y* | |  | ) |  |  | | protected |

References g\_msg, SupplyDayInYear(), SupplyDeadBiomass(), SupplyGreenBiomass(), SupplyInsects(), SupplyLATotal(), SupplyVegBiomass(), SupplyVegCover(), SupplyVegDigestability(), SupplyVegHeight(), SupplyVegType(), SupplyWeedBiomass(), MapErrorMsg::Warn(), and WARN\_FILE.

Referenced by Tick().

{

FILE \* vfile=fopen("VegDump.txt", "a" );

if (!vfile) {

g\_msg->Warn( WARN\_FILE, "Landscape::VegDump(): Unable to open file", "VegDump.txt" );

exit( 1 );

}

int day = SupplyDayInYear();

double hei = SupplyVegHeight( x, y );

double bio = SupplyVegBiomass( x, y );

double cover = SupplyVegCover( x, y );

double density = bio / ( hei + 1 );

double weeds = SupplyWeedBiomass( x, y );

double insects = SupplyInsects( x, y );

double LATotal = SupplyLATotal(x,y);

double digest = SupplyVegDigestability(x,y);

double GreenBiomass = SupplyGreenBiomass(x,y);

double DeadBiomass = SupplyDeadBiomass(x,y);

TTypesOfVegetation VegType = SupplyVegType( x, y );

fprintf( vfile, "%d\t%g\t%g\t%g\t%g\t%g\t%i\t%g\t%g\t%g\t%g\t%g\t--- ", day, hei, bio, density, cover, weeds, VegType, insects, LATotal, digest, GreenBiomass, DeadBiomass );

y += 100;

x += 100;

hei = SupplyVegHeight( x, y );

bio = SupplyVegBiomass( x, y );

cover = SupplyVegCover( x, y );

density = bio / ( hei + 1 );

weeds = SupplyWeedBiomass( x, y );

VegType = SupplyVegType( x, y );

insects = SupplyInsects( x, y );

digest = SupplyVegDigestability(x,y);

fprintf( vfile, "%g\t%g\t%g\t%g\t%g\t%i\t%g\t%g\n", hei, bio, density, cover, weeds, VegType, insects, digest );

fclose( vfile );

}

|  |  |  |  |  |  |
| --- | --- | --- | --- | --- | --- |
| char \* Landscape::VegtypeToString | ( | TTypesOfVegetation | *a\_veg* | ) |  |

References g\_msg, tov\_AgroChemIndustryCereal, tov\_Carrots, tov\_CloverGrassGrazed1, tov\_CloverGrassGrazed2, tov\_FieldPeas, tov\_FieldPeasStrigling, tov\_FodderBeet, tov\_FodderGrass, tov\_Lawn, tov\_Maize, tov\_MaizeSilage, tov\_MaizeStrigling, tov\_NaturalGrass, tov\_NoGrowth, tov\_None, tov\_Oats, tov\_OBarleyPeaCloverGrass, tov\_OCarrots, tov\_OCloverGrassGrazed1, tov\_OCloverGrassGrazed2, tov\_OCloverGrassSilage1, tov\_OFieldPeas, tov\_OFieldPeasSilage, tov\_OFirstYearDanger, tov\_OGrazingPigs, tov\_OMaizeSilage, tov\_OOats, tov\_OPermanentGrassGrazed, tov\_OPotatoes, tov\_Orchard, tov\_OSBarleySilage, tov\_OSeedGrass1, tov\_OSeedGrass2, tov\_OSetaside, tov\_OSpringBarley, tov\_OSpringBarleyClover, tov\_OSpringBarleyExt, tov\_OSpringBarleyGrass, tov\_OSpringBarleyPigs, tov\_OTriticale, tov\_OWinterBarley, tov\_OWinterBarleyExt, tov\_OWinterRape, tov\_OWinterRye, tov\_OWinterWheatUndersown, tov\_OWinterWheatUndersownExt, tov\_PermanentGrassGrazed, tov\_PermanentGrassLowYield, tov\_PermanentGrassTussocky, tov\_PermanentSetaside, tov\_Potatoes, tov\_PotatoesIndustry, tov\_SeedGrass1, tov\_SeedGrass2, tov\_Setaside, tov\_SpringBarley, tov\_SpringBarleyCloverGrass, tov\_SpringBarleyCloverGrassStrigling, tov\_SpringBarleyGrass, tov\_SpringBarleyPeaCloverGrassStrigling, tov\_SpringBarleyPTreatment, tov\_SpringBarleySeed, tov\_SpringBarleySilage, tov\_SpringBarleySKManagement, tov\_SpringBarleyStrigling, tov\_SpringBarleyStriglingCulm, tov\_SpringBarleyStriglingSingle, tov\_SpringRape, tov\_SpringWheat, tov\_Triticale, tov\_Undefined, tov\_WinterBarley, tov\_WinterBarleyStrigling, tov\_WinterRape, tov\_WinterRapeStrigling, tov\_WinterRye, tov\_WinterRyeStrigling, tov\_WinterWheat, tov\_WinterWheatShort, tov\_WinterWheatStrigling, tov\_WinterWheatStriglingCulm, tov\_WinterWheatStriglingSingle, tov\_WWheatPControl, tov\_WWheatPToxicControl, tov\_WWheatPTreatment, tov\_YoungForest, MapErrorMsg::Warn(), and WARN\_FILE.

Referenced by DumpMapInfoByArea(), and Skylark\_Population\_Manager::OpenTheFledgelingProbe().

{

char error\_num[ 20 ];

switch ( a\_veg ) {

case tov\_Carrots:

return "Carrots ";

case tov\_FodderGrass:

return "FodderGrass ";

case tov\_CloverGrassGrazed1:

return "CloverGrassGrazed1 ";

case tov\_CloverGrassGrazed2:

return "CloverGrassGrazed2 ";

case tov\_FieldPeas:

return "FieldPeas ";

case tov\_FodderBeet:

return "FodderBeet ";

case tov\_Lawn:

return "Lawn ";

case tov\_Maize:

return "Maize ";

case tov\_MaizeSilage:

return "MaizeSilage ";

case tov\_OMaizeSilage:

return "OMaizeSilage ";

case tov\_NaturalGrass:

return "NaturalGrass ";

case tov\_NoGrowth:

return "NoGrowth ";

case tov\_None:

return "None ";

case tov\_Oats:

return "Oats ";

case tov\_OBarleyPeaCloverGrass:

return "OBarleyPeaCloverGrass";

case tov\_OCarrots:

return "OCarrots ";

case tov\_OCloverGrassGrazed1:

return "OCloverGrassGrazed1 ";

case tov\_OCloverGrassGrazed2:

return "OCloverGrassGrazed2 ";

case tov\_OCloverGrassSilage1:

return "OCloverGrassSilage1 ";

case tov\_OFieldPeas:

return "OFieldPeas ";

case tov\_OFieldPeasSilage:

return "OFieldPeasSilage ";

case tov\_OFirstYearDanger:

return "OFirstYearDanger ";

case tov\_OGrazingPigs:

return "OGrazingPigs ";

case tov\_OOats:

return "OOats ";

case tov\_OPermanentGrassGrazed:

return "OPermanentGrassGrazed";

case tov\_OPotatoes:

return "OPotatoesEat ";

case tov\_OSBarleySilage:

return "OSBarleySilage ";

case tov\_OSeedGrass1:

return "OSeedGrass1 ";

case tov\_OSeedGrass2:

return "OSeedGrass2 ";

case tov\_OSetaside:

return "OSetasid ";

case tov\_OSpringBarley:

return "OSpringBarley ";

case tov\_OSpringBarleyExt:

return "OSpringBarleyExt ";

case tov\_OSpringBarleyClover:

return "OSpringBarleyClover ";

case tov\_OSpringBarleyGrass:

return "OSpringBarleyGrass ";

case tov\_OSpringBarleyPigs:

return "OSpringBarleyPigs ";

case tov\_OTriticale:

return "OTriticale ";

case tov\_OWinterBarley:

return "OWinterBarley ";

case tov\_OWinterBarleyExt:

return "OWinterBarleyExt ";

case tov\_OWinterRape:

return "OWinterRape ";

case tov\_OWinterRye:

return "OWinterRye ";

case tov\_OWinterWheatUndersown:

return "OWinterWheatUndersown";

case tov\_OWinterWheatUndersownExt:

return "OWinterWheatUsowExt ";

case tov\_PermanentGrassGrazed:

return "PermanentGrassGrazed ";

case tov\_PermanentGrassLowYield:

return "PermGrassTussocky ";

case tov\_PermanentGrassTussocky:

return "PermGrassTussocky ";

case tov\_PermanentSetaside:

return "PermanentSetaside ";

case tov\_Potatoes:

return "PotatoesEat ";

case tov\_PotatoesIndustry:

return "PotatoesIndustry ";

case tov\_SeedGrass1:

return "SeedGrass1 ";

case tov\_SeedGrass2:

return "SeedGrass2 ";

case tov\_Setaside:

return "Setaside ";

case tov\_SpringBarley:

return "SpringBarley ";

case tov\_SpringBarleyPTreatment:

return "SpringBarleyPTreat ";

case tov\_SpringBarleySKManagement:

return "SpringBarleySKMan ";

case tov\_SpringBarleyCloverGrass:

return "SprBarleyCloverGrass ";

case tov\_SpringBarleyGrass:

return "SpringBarleyGrass ";

case tov\_SpringBarleySeed:

return "SpringBarleySeed ";

case tov\_SpringBarleySilage:

return "SpringBarleySilage ";

case tov\_SpringRape:

return "SpringRape ";

case tov\_SpringWheat:

return "SpringWheat ";

case tov\_AgroChemIndustryCereal:

return "AgroChemIndustry Cereal ";

case tov\_Triticale:

return "Triticale ";

case tov\_WinterBarley:

return "WinterBarley ";

case tov\_WinterRape:

return "WinterRape ";

case tov\_WinterRye:

return "WinterRye ";

case tov\_WinterWheat:

return "WinterWheat ";

case tov\_WinterWheatShort:

return "WinterWheatShort ";

case tov\_WWheatPControl:

return "P Trial Control ";

case tov\_WWheatPToxicControl:

return "P Trial Toxic Control";

case tov\_WWheatPTreatment:

return "P Trial Treatment ";

case tov\_Orchard:

return "Orchard ";

case tov\_Undefined:

return "Undefined ";

case tov\_WinterWheatStrigling:

return "WWStrigling ";

case tov\_WinterWheatStriglingSingle:

return "WWStriglingSingle ";

case tov\_WinterWheatStriglingCulm:

return "WWStriglingCulm ";

case tov\_SpringBarleyCloverGrassStrigling:

return "SBPCGStrigling ";

case tov\_SpringBarleyStrigling:

return "SBarleyStrigling ";

case tov\_SpringBarleyStriglingSingle:

return "SBarleyStriglingSgl ";

case tov\_SpringBarleyStriglingCulm:

return "SBarleyStriglingCulm ";

case tov\_MaizeStrigling:

return "MaizseStrigling ";

case tov\_WinterRapeStrigling:

return "WRapeStrigling ";

case tov\_WinterRyeStrigling:

return "WRyeStrigling ";

case tov\_WinterBarleyStrigling:

return "WBStrigling ";

case tov\_FieldPeasStrigling:

return "FieldPeasStrigling ";

case tov\_SpringBarleyPeaCloverGrassStrigling:

return "SBPeaCloverGrassStr ";

case tov\_YoungForest:

return "Young Forest ";

default:

sprintf( error\_num, "%d", a\_veg );

g\_msg->Warn( WARN\_FILE, "Landscape::VegtypeToString(): Unknown event type:", error\_num );

exit( 1 );

}

}

|  |  |  |  |  |  |  |  |  |  |  |  |  |  |
| --- | --- | --- | --- | --- | --- | --- | --- | --- | --- | --- | --- | --- | --- |
| |  |  |  |  | | --- | --- | --- | --- | | void Landscape::Warn | ( | const char \* | *a\_msg1*, | |  |  | const char \* | *a\_msg2* | |  | ) |  |  | | inline |

References g\_msg, MapErrorMsg::Warn(), and WARN\_MSG.

Referenced by Skylark\_Clutch::EndStep(), Skylark\_Nestling::EndStep(), Skylark\_PreFledgeling::EndStep(), SkTerritories::EvaluateHabitatN(), GetProbeInput\_ini(), Skylark\_Male::OnAddPreFledgeling(), Skylark\_Female::OnClutchDeath(), Skylark\_Male::OnEggHatch(), Skylark\_Female::OnEggsHatch(), Skylark\_Clutch::OnFarmEvent(), Skylark\_Nestling::OnFarmEvent(), Skylark\_PreFledgeling::OnFarmEvent(), Skylark\_Female::OnFarmEvent(), Skylark\_Male::OnFarmEvent(), Skylark\_Male::OnNestlingDeath(), Skylark\_Male::OnPreFledgelingDeath(), SkTerritories::PrePoly2Qual(), SkTerritories::PrePolyNQual(), SkTerritories::PreProcessLandscape2(), Skylark\_Female::st\_Dying(), Skylark\_Male::st\_Dying(), Skylark\_Clutch::st\_Hatching(), Skylark\_Clutch::Step(), Skylark\_Nestling::Step(), Skylark\_PreFledgeling::Step(), Skylark\_Female::Step(), and Skylark\_Male::Step().

{

g\_msg->Warn( WARN\_MSG, a\_msg1, a\_msg2 );

}

---

## Member Data Documentation

|  |  |  |
| --- | --- | --- |
| |  | | --- | | int Landscape::hb\_border\_pixels | | protected |

Referenced by hb\_Add(), and hb\_PaintBorder().

|  |  |  |
| --- | --- | --- |
| |  | | --- | | int Landscape::hb\_core\_pixels | | protected |

Referenced by hb\_Add(), and hb\_PaintBorder().

|  |  |  |
| --- | --- | --- |
| |  | | --- | | int Landscape::hb\_first\_free\_poly\_num | | protected |

Referenced by BorderAdd(), hb\_GenerateHBPolys(), Landscape(), OrchardBorderAdd(), and UnsprayedMarginAdd().

|  |  |  |
| --- | --- | --- |
| |  | | --- | | vector<int> Landscape::hb\_hedges | | protected |

Referenced by hb\_Add(), and hb\_FindHedges().

|  |  |  |
| --- | --- | --- |
| |  | | --- | | int Landscape::hb\_height | | protected |

Referenced by hb\_Add(), hb\_FindBoundingBox(), hb\_HasNeighbourColor(), hb\_MapBorder(), and hb\_MaxUnpaintedNegNeighbour().

|  |  |  |
| --- | --- | --- |
| |  | | --- | | int\* Landscape::hb\_map | | protected |

Referenced by hb\_Add(), hb\_AddNewHedgebanks(), hb\_ClearPolygon(), hb\_DownPolyNumbers(), hb\_FindBoundingBox(), hb\_HasNeighbourColor(), hb\_HasOtherNeighbour(), hb\_MarkTheBresenhamWay(), hb\_MarkTopFromLocalMax(), hb\_MaxUnpaintedNegNeighbour(), hb\_PaintBorder(), hb\_PaintWhoHasNeighbourColor(), hb\_ResetColorBits(), hb\_RestoreHedgeCore(), and hb\_UpPolyNumbers().

|  |  |  |
| --- | --- | --- |
| |  | | --- | | int Landscape::hb\_max\_x | | protected |

Referenced by hb\_AddNewHedgebanks(), hb\_ClearPolygon(), hb\_FindBoundingBox(), hb\_MarkTheBresenhamWay(), hb\_MarkTopFromLocalMax(), hb\_PaintBorder(), hb\_PaintWhoHasNeighbourColor(), hb\_ResetColorBits(), and hb\_RestoreHedgeCore().

|  |  |  |
| --- | --- | --- |
| |  | | --- | | int Landscape::hb\_max\_y | | protected |

Referenced by hb\_AddNewHedgebanks(), hb\_ClearPolygon(), hb\_FindBoundingBox(), hb\_MarkTheBresenhamWay(), hb\_MarkTopFromLocalMax(), hb\_PaintBorder(), hb\_PaintWhoHasNeighbourColor(), hb\_ResetColorBits(), and hb\_RestoreHedgeCore().

|  |  |  |
| --- | --- | --- |
| |  | | --- | | int Landscape::hb\_min\_x | | protected |

Referenced by hb\_AddNewHedgebanks(), hb\_ClearPolygon(), hb\_FindBoundingBox(), hb\_MarkTheBresenhamWay(), hb\_MarkTopFromLocalMax(), hb\_PaintBorder(), hb\_PaintWhoHasNeighbourColor(), hb\_ResetColorBits(), and hb\_RestoreHedgeCore().

|  |  |  |
| --- | --- | --- |
| |  | | --- | | int Landscape::hb\_min\_y | | protected |

Referenced by hb\_AddNewHedgebanks(), hb\_ClearPolygon(), hb\_FindBoundingBox(), hb\_MarkTheBresenhamWay(), hb\_MarkTopFromLocalMax(), hb\_PaintBorder(), hb\_PaintWhoHasNeighbourColor(), hb\_ResetColorBits(), and hb\_RestoreHedgeCore().

|  |  |  |
| --- | --- | --- |
| |  | | --- | | vector<LE\*> Landscape::hb\_new\_hbs | | protected |

Referenced by hb\_GenerateHBPolys(), and hb\_StripingDist().

|  |  |  |
| --- | --- | --- |
| |  | | --- | | int Landscape::hb\_size | | protected |

Referenced by hb\_Add(), hb\_DownPolyNumbers(), and hb\_UpPolyNumbers().

|  |  |  |
| --- | --- | --- |
| |  | | --- | | int Landscape::hb\_width | | protected |

Referenced by hb\_Add(), hb\_AddNewHedgebanks(), hb\_ClearPolygon(), hb\_FindBoundingBox(), hb\_HasNeighbourColor(), hb\_HasOtherNeighbour(), hb\_MapBorder(), hb\_MarkTheBresenhamWay(), hb\_MarkTopFromLocalMax(), hb\_MaxUnpaintedNegNeighbour(), hb\_PaintBorder(), hb\_PaintWhoHasNeighbourColor(), hb\_ResetColorBits(), and hb\_RestoreHedgeCore().

|  |  |  |
| --- | --- | --- |
| |  | | --- | | double\* Landscape::l\_vegtype\_areas | | private |

Referenced by DumpMapInfoByArea(), FillVegAreaData(), GetVegArea(), Landscape(), and ~Landscape().

|  |  |  |
| --- | --- | --- |
| |  | | --- | | int Landscape::le\_signal\_index | | protected |

Referenced by SupplyLENext(), and SupplyLEReset().

|  |  |  |
| --- | --- | --- |
| |  | | --- | | vector<LE\*> Landscape::m\_elems | | private |

Referenced by AddBeetleBanks(), BeetleBankAdd(), BorderAdd(), BorderTest(), BuildingDesignationCalc(), CalculateCentroids(), ChangeMapMapping(), CountMapSquares(), DumpCentroids(), DumpMap(), FillVegAreaData(), ForceArea(), hb\_Add(), hb\_FindHedges(), hb\_GenerateHBPolys(), Landscape(), MagicMapP2PolyRef(), OrchardBorderAdd(), PolysDump(), PolysRemoveInvalid(), PolysValidate(), ReadPolys(), SetLESignal(), SetPolyMaxMinExtents(), SkylarkEvaluation(), StepOneValid(), SupplyCountryDesig(), SupplyDayDegrees(), SupplyDeadBiomass(), SupplyElementSubType(), SupplyElementType(), SupplyElementTypeCC(), SupplyElementTypeFromVector(), SupplyFarmArea(), SupplyFarmIntensity(), SupplyFarmOwner(), SupplyFarmOwnerIndex(), SupplyFarmType(), SupplyGrazingPressure(), SupplyGrazingPressureVector(), SupplyGreenBiomass(), SupplyHasTramlines(), SupplyInsects(), SupplyIsGrass(), SupplyJustMown(), SupplyJustMownVector(), SupplyJustSprayed(), SupplyJustSprayedVector(), SupplyLAGreen(), SupplyLastTreatment(), SupplyLATotal(), SupplyLECount(), SupplyLENext(), SupplyLEPointer(), SupplyLESignal(), SupplyNumberOfPolygons(), SupplyPesticideCell(), SupplyPolygonAreaVector(), SupplyPolyRef(), SupplyPolyRefCC(), SupplyPolyRefVector(), SupplySkScrapes(), SupplyTrafficLoad(), SupplyValidX(), SupplyValidY(), SupplyVegAge(), SupplyVegBiomass(), SupplyVegBiomassVector(), SupplyVegCover(), SupplyVegCoverVector(), SupplyVegDensity(), SupplyVegDigestability(), SupplyVegDigestabilityVector(), SupplyVegHeight(), SupplyVegHeightVector(), SupplyVegPatchy(), SupplyVegType(), SupplyVegTypeVector(), SupplyWeedBiomass(), Tick(), UnsprayedMarginAdd(), and ~Landscape().

|  |  |  |
| --- | --- | --- |
| |  | | --- | | FarmManager\* Landscape::m\_FarmManager | | private |

Referenced by ChangeMapMapping(), Landscape(), ReadPolys(), Tick(), and ~Landscape().

|  |  |  |
| --- | --- | --- |
| |  | | --- | | int\* Landscape::m\_farmmapping | | private |

Referenced by ChangeMapMapping(), ReadPolys(), and ~Landscape().

|  |  |  |
| --- | --- | --- |
| |  | | --- | | int Landscape::m\_height | | private |

Referenced by CorrectCoords(), CorrectHeight(), DumpMap(), DumpMapGraphics(), ForceArea(), Landscape(), SupplyElementTypeCC(), and SupplyPolyRefCC().

|  |  |  |
| --- | --- | --- |
| |  | | --- | | int Landscape::m\_height10 | | private |

Referenced by CorrectCoords(), CorrectHeight(), Landscape(), SupplyElementTypeCC(), and SupplyPolyRefCC().

|  |  |  |
| --- | --- | --- |
| |  | | --- | | RasterMap\* Landscape::m\_land | | private |

Referenced by BeetleBankAdd(), BorderScan(), BorderScan2(), BorderStep(), BorderTest(), ChangeMapMapping(), CountMapSquares(), DumpMap(), FindValidXY(), hb\_Add(), Landscape(), OrchardBorderAdd(), PolysValidate(), SetPolyMaxMinExtents(), StepOneValid(), SupplyCountryDesig(), SupplyDeadBiomass(), SupplyElementSubType(), SupplyElementType(), SupplyElementTypeCC(), SupplyFarmIntensity(), SupplyFarmOwner(), SupplyFarmOwnerIndex(), SupplyFarmType(), SupplyGrazingPressure(), SupplyGreenBiomass(), SupplyHasTramlines(), SupplyInsects(), SupplyJustSprayed(), SupplyLAGreen(), SupplyLastTreatment(), SupplyLATotal(), SupplyMagicMapP(), SupplyPolyRef(), SupplyPolyRefCC(), SupplyPolyRefIndex(), SupplySimAreaHeight(), SupplySimAreaWidth(), SupplyTrafficLoad(), SupplyVegAge(), SupplyVegBiomass(), SupplyVegCover(), SupplyVegDensity(), SupplyVegDigestability(), SupplyVegHeight(), SupplyVegPatchy(), SupplyVegType(), SupplyWeedBiomass(), UMarginTest(), UnsprayedMarginScan(), and ~Landscape().

|  |  |  |
| --- | --- | --- |
| |  | | --- | | int Landscape::m\_LargestPolyNumUsed | | protected |

Referenced by BeetleBankAdd(), Landscape(), ReadPolys(), and SupplyLargestPolyNumUsed().

|  |  |  |
| --- | --- | --- |
| |  | | --- | | TTypesOfPesticide Landscape::m\_PesticideType | | protected |

An attribute to hold the pesticide type being tested, if there is one, if not default is -1.

Referenced by Landscape(), and SupplyPesticideType().

|  |  |  |
| --- | --- | --- |
| |  | | --- | | RodenticideManager\* Landscape::m\_RodenticideManager | | private |

Referenced by Landscape(), SupplyRodenticide(), Tick(), and ~Landscape().

|  |  |  |
| --- | --- | --- |
| |  | | --- | | bool Landscape::m\_toxShouldSpray | | protected |

Referenced by Landscape(), SupplyShouldSpray(), and Tick().

|  |  |  |
| --- | --- | --- |
| |  | | --- | | int Landscape::m\_treatment\_counts[last\_treatment] | | protected |

Referenced by DumpTreatCounters(), IncTreatCounter(), and Landscape().

|  |  |  |
| --- | --- | --- |
| |  | | --- | | char Landscape::m\_versioninfo[30] | | private |

Referenced by Landscape(), and SupplyVersion().

|  |  |  |
| --- | --- | --- |
| |  | | --- | | int Landscape::m\_width | | private |

Referenced by CentroidSpiralOut(), CorrectCoords(), CorrectWidth(), DumpMap(), DumpMapGraphics(), ForceArea(), Landscape(), SupplyElementTypeCC(), and SupplyPolyRefCC().

|  |  |  |
| --- | --- | --- |
| |  | | --- | | int Landscape::m\_width10 | | private |

Referenced by CorrectCoords(), CorrectWidth(), Landscape(), SupplyElementTypeCC(), and SupplyPolyRefCC().

|  |  |  |
| --- | --- | --- |
| |  | | --- | | int Landscape::m\_x\_add[8] | | protected |

Referenced by AxisLoop(), BeetleBankAdd(), FindFieldCenter(), FindLongestAxis(), and Landscape().

|  |  |  |
| --- | --- | --- |
| |  | | --- | | int Landscape::m\_y\_add[8] | | protected |

Referenced by AxisLoop(), BeetleBankAdd(), FindFieldCenter(), FindLongestAxis(), and Landscape().

---

The documentation for this class was generated from the following files:

- landscape.h
- hedgebanks.cpp
- Landscape.cpp
- misc.cpp


- Landscape
- Generated on Thu Jan 10 2013 13:15:36 for ALMaSS Skylark ODdox by
   1.8.1.1
