# Supplementary material for: Modelling Skylarks (Alauda arvensis) to Predict Impacts of Changes in Land Management and Policy: Development and Testing of an Agent-Based Model
Source: PLoS One. 2013 Jun 6;8(6):e65803. doi: 10.1371/journal.pone.0065803 (PMC3675089; doi:10.1371/journal.pone.0065803)
Supplement: Supporting Information S4 — The skylark ODdox as a zipped archive. (ZIP) [file pone.0065803.s004.zip › Skylark_ODdox/class_landscape-members.html]

ALMaSS Skylark ODdox: Member List


|  |
| --- |
| ALMaSS Skylark ODdox  2.0 |


- Main Page
- Related Pages
- Classes
- Files

- Class List
- Class Index
- Class Hierarchy
- Class Members

Landscape Member List

This is the complete list of members for Landscape, including all inherited members.

|  |  |  |
| --- | --- | --- |
| AddBeetleBanks(void) | Landscape | protected |
| AddGreenElement(LE \*a\_green) | Landscape | protected |
| AddToClusterList(int \*, int \*, int \*, int \*, int \*) | Landscape | protected |
| AxisLoop(int a\_poly, int \*x1, int \*y1, int a\_axis) | Landscape | protected |
| BackTranslateEleTypes(TTypesOfLandscapeElement EleReference) | Landscape | inline |
| BackTranslateVegTypes(TTypesOfVegetation VegReference) | Landscape | inline |
| BeetleBankAdd(int x, int y, int angle, int length, LE \*a\_field) | Landscape | protected |
| BeetleBankPossible(LE \*a\_field) | Landscape | protected |
| BorderAdd(LE \*a\_field) | Landscape | protected |
| BorderNeed(TTypesOfLandscapeElement a\_letype) | Landscape | protected |
| BorderScan(LE \*a\_field) | Landscape | protected |
| BorderScan2(LE \*a\_poly) | Landscape | protected |
| BorderStep(int a\_fieldpoly, int a\_borderpoly, int \*a\_x, int \*a\_y) | Landscape | protected |
| BorderTest(int a\_fieldpoly, int a\_borderpoly, int a\_x, int a\_y) | Landscape | protected |
| BuildingDesignationCalc() | Landscape |  |
| CalculateCentroids(void) | Landscape |  |
| CentroidSpiralOut(int a\_polyref, int &a\_x, int &a\_y) | Landscape |  |
| ChangeMapMapping(void) | Landscape | protected |
| CIPELandscapeMaker() | Landscape | protected |
| CorrectCoords(int &x, int &y) | Landscape | inline |
| CorrectHeight(int y) | Landscape | inline |
| CorrectWidth(int x) | Landscape | inline |
| CountMapSquares(void) | Landscape | protected |
| DumpAllSymbolsAndExit(const char \*a\_dumpfile) | Landscape | inline |
| DumpCentroids(void) | Landscape |  |
| DumpMap(const char \*a\_filename) | Landscape | protected |
| DumpMapGraphics(const char \*a\_filename) | Landscape | protected |
| DumpMapInfoByArea(const char \*a\_filename, bool a\_append, bool a\_dump\_zero\_areas, bool a\_write\_veg\_names) | Landscape |  |
| DumpPublicSymbols(const char \*a\_dumpfile, CfgSecureLevel a\_level) | Landscape | inline |
| DumpTreatCounters(const char \*a\_filename) | Landscape | protected |
| DumpVegAreaData(int a\_day) | Landscape |  |
| EventDump(int x, int y, int x2, int y2) | Landscape | protected |
| EventtypeToString(int a\_event) | Landscape |  |
| FillVegAreaData() | Landscape |  |
| FindFieldCenter(LE \*a\_field, int \*x, int \*y) | Landscape | protected |
| FindLongestAxis(int \*x, int \*y, int \*a\_length) | Landscape | protected |
| FindValidXY(int a\_field, int &a\_x, int &a\_y) | Landscape | protected |
| ForceArea(void) | Landscape | protected |
| GetNextSeed(int) | Landscape | protected |
| GetVegArea(int v) | Landscape | inline |
| hb\_Add(void) | Landscape | protected |
| hb\_AddNewHedgebanks(int a\_orig\_poly\_num) | Landscape | protected |
| hb\_border\_pixels | Landscape | protected |
| hb\_Cleanup(void) | Landscape | protected |
| hb\_ClearPolygon(int a\_poly\_num) | Landscape | protected |
| hb\_core\_pixels | Landscape | protected |
| hb\_DownPolyNumbers(void) | Landscape | protected |
| hb\_FindBoundingBox(int a\_poly\_num) | Landscape | protected |
| hb\_FindHedges(void) | Landscape | protected |
| hb\_first\_free\_poly\_num | Landscape | protected |
| hb\_GenerateHBPolys(void) | Landscape | protected |
| hb\_HasNeighbourColor(int a\_x, int a\_y, int a\_neighbour\_color) | Landscape | inlineprotected |
| hb\_HasOtherNeighbour(int a\_x, int a\_y) | Landscape | inlineprotected |
| hb\_hedges | Landscape | protected |
| hb\_height | Landscape | protected |
| hb\_map | Landscape | protected |
| hb\_MapBorder(int a\_x, int a\_y) | Landscape | inlineprotected |
| hb\_MarkTheBresenhamWay(void) | Landscape | protected |
| hb\_MarkTopFromLocalMax(int a\_color) | Landscape | protected |
| hb\_max\_x | Landscape | protected |
| hb\_max\_y | Landscape | protected |
| hb\_MaxUnpaintedNegNeighbour(int a\_x, int a\_y) | Landscape | inlineprotected |
| hb\_min\_x | Landscape | protected |
| hb\_min\_y | Landscape | protected |
| hb\_new\_hbs | Landscape | protected |
| hb\_PaintBorder(int a\_color) | Landscape | protected |
| hb\_PaintWhoHasNeighbourColor(int a\_neighbour\_color, int a\_new\_color) | Landscape | protected |
| hb\_ResetColorBits(void) | Landscape | protected |
| hb\_RestoreHedgeCore(int a\_orig\_poly\_number) | Landscape | protected |
| hb\_size | Landscape | protected |
| hb\_StripingDist(void) | Landscape | protected |
| hb\_UpPolyNumbers(void) | Landscape | protected |
| hb\_width | Landscape | protected |
| IncTreatCounter(int a\_treat) | Landscape |  |
| l\_vegtype\_areas | Landscape | private |
| Landscape(const char \*a\_configfile, const char \*a\_errorfile) | Landscape |  |
| le\_signal\_index | Landscape | protected |
| m\_elems | Landscape | private |
| m\_FarmManager | Landscape | private |
| m\_farmmapping | Landscape | private |
| m\_height | Landscape | private |
| m\_height10 | Landscape | private |
| m\_land | Landscape | private |
| m\_LargestPolyNumUsed | Landscape | protected |
| m\_PesticideType | Landscape | protected |
| m\_RodenticideManager | Landscape | private |
| m\_toxShouldSpray | Landscape | protected |
| m\_treatment\_counts | Landscape | protected |
| m\_versioninfo | Landscape | private |
| m\_width | Landscape | private |
| m\_width10 | Landscape | private |
| m\_x\_add | Landscape | protected |
| m\_y\_add | Landscape | protected |
| MagicMapP2PolyRef(int a\_magic) | Landscape | inline |
| MakeCluster(void) | Landscape | protected |
| ModifyPolyRef(int \*) | Landscape | protected |
| NewElement(TTypesOfLandscapeElement a\_type) | Landscape | protected |
| OrchardBorderAdd(LE \*a\_field) | Landscape | protected |
| PolysDump(const char \*a\_filename) | Landscape | protected |
| PolysRemoveInvalid(void) | Landscape | protected |
| PolysValidate(bool a\_exit\_on\_invalid) | Landscape | protected |
| PolytypeToString(TTypesOfLandscapeElement a\_le\_type) | Landscape |  |
| RandomLocation(void) | Landscape | protected |
| ReadInput(int \*, int \*, int \*, Point \*) | Landscape | protected |
| ReadPolys(const char \*a\_polyfile) | Landscape | protected |
| ReadSymbols(const char \*a\_cfgfile) | Landscape | inline |
| SetLESignal(int a\_polyref, LE\_Signal a\_signal) | Landscape |  |
| SetPolyMaxMinExtents(void) | Landscape |  |
| SkylarkEvaluation(SkTerritories \*a\_skt) | Landscape |  |
| StepOneValid(int a\_polyindex, int a\_x, int a\_y, int step) | Landscape | protected |
| SupplyCountryDesig(int a\_x, int a\_y) | Landscape | inline |
| SupplyDayDegrees(int a\_polyref) | Landscape | inline |
| SupplyDayInMonth(void) | Landscape | inline |
| SupplyDayInYear(void) | Landscape | inline |
| SupplyDaylength(void) | Landscape | inline |
| SupplyDaylength(long a\_date) | Landscape | inline |
| SupplyDeadBiomass(int a\_polyref) | Landscape | inline |
| SupplyDeadBiomass(int a\_x, int a\_y) | Landscape | inline |
| SupplyElementSubType(int a\_polyref) | Landscape | inline |
| SupplyElementSubType(int a\_x, int a\_y) | Landscape | inline |
| SupplyElementType(int a\_polyref) | Landscape | inline |
| SupplyElementType(int a\_x, int a\_y) | Landscape | inline |
| SupplyElementTypeCC(int a\_x, int a\_y) | Landscape | inline |
| SupplyElementTypeFromVector(unsigned int a\_index) | Landscape | inline |
| SupplyFarmArea(int a\_polyref) | Landscape | inline |
| SupplyFarmIntensity(int a\_x, int a\_y) | Landscape |  |
| SupplyFarmIntensity(int a\_polyref) | Landscape |  |
| SupplyFarmOwner(int a\_x, int a\_y) | Landscape | inline |
| SupplyFarmOwner(int a\_polyref) | Landscape | inline |
| SupplyFarmOwnerIndex(int a\_x, int a\_y) | Landscape | inline |
| SupplyFarmOwnerIndex(int a\_polyref) | Landscape | inline |
| SupplyFarmType(int a\_polyref) | Landscape | inline |
| SupplyFarmType(int a\_x, int a\_y) | Landscape | inline |
| SupplyGlobalDate(void) | Landscape | inline |
| SupplyGlobalRadiation() | Landscape | inline |
| SupplyGlobalRadiation(long a\_date) | Landscape | inline |
| SupplyGrazingPressure(int a\_polyref) | Landscape | inline |
| SupplyGrazingPressure(int a\_x, int a\_y) | Landscape | inline |
| SupplyGrazingPressureVector(unsigned int a\_index) | Landscape | inline |
| SupplyGreenBiomass(int a\_polyref) | Landscape | inline |
| SupplyGreenBiomass(int a\_x, int a\_y) | Landscape | inline |
| SupplyHasTramlines(int a\_x, int a\_y) | Landscape | inline |
| SupplyHasTramlines(int a\_polyref) | Landscape | inline |
| SupplyInsects(int a\_polyref) | Landscape | inline |
| SupplyInsects(int a\_x, int a\_y) | Landscape | inline |
| SupplyIsGrass(int a\_polyref) | Landscape | inline |
| SupplyJustMown(int a\_polyref) | Landscape | inline |
| SupplyJustMownVector(unsigned int a\_index) | Landscape | inline |
| SupplyJustSprayed(int a\_polyref) | Landscape | inline |
| SupplyJustSprayed(int a\_x, int a\_y) | Landscape | inline |
| SupplyJustSprayedVector(unsigned int a\_index) | Landscape | inline |
| SupplyLAGreen(int a\_polyref) | Landscape | inline |
| SupplyLAGreen(int a\_x, int a\_y) | Landscape | inline |
| SupplyLargestPolyNumUsed() | Landscape | inline |
| SupplyLastTreatment(int a\_polyref, int \*a\_index) | Landscape | inline |
| SupplyLastTreatment(int a\_x, int a\_y, int \*a\_index) | Landscape | inline |
| SupplyLATotal(int a\_x, int a\_y) | Landscape | inline |
| SupplyLECount(void) | Landscape |  |
| SupplyLENext(void) | Landscape |  |
| SupplyLEPointer(int a\_polyref) | Landscape | inline |
| SupplyLEReset(void) | Landscape |  |
| SupplyLESignal(int a\_polyref) | Landscape |  |
| SupplyMagicMapP(int a\_x, int a\_y) | Landscape | inline |
| SupplyMeanTemp(long a\_date, unsigned int a\_period) | Landscape | inline |
| SupplyMonth(void) | Landscape | inline |
| SupplyNumberOfPolygons(void) | Landscape | inline |
| SupplyPesticide(int a\_x, int a\_y) | Landscape |  |
| SupplyPesticide(int a\_polyref) | Landscape |  |
| SupplyPesticideCell(int a\_polyref) | Landscape | inline |
| SupplyPesticideType(void) | Landscape | inline |
| SupplyPolygonAreaVector(int a\_polyref) | Landscape | inline |
| SupplyPolyRef(int a\_x, int a\_y) | Landscape | inline |
| SupplyPolyRefCC(int a\_x, int a\_y) | Landscape | inline |
| SupplyPolyRefIndex(int a\_x, int a\_y) | Landscape | inline |
| SupplyPolyRefVector(unsigned int a\_index) | Landscape | inline |
| SupplyRain(void) | Landscape | inline |
| SupplyRain(long a\_date) | Landscape | inline |
| SupplyRainPeriod(long a\_date, int a\_period) | Landscape | inline |
| SupplyRoadWidth(int, int) | Landscape | inline |
| SupplyRodenticide(int a\_x, int a\_y) | Landscape |  |
| SupplyShouldSpray() | Landscape | inline |
| SupplySimAreaHeight(void) | Landscape | inline |
| SupplySimAreaWidth(void) | Landscape | inline |
| SupplySkScrapes(int a\_polyref) | Landscape | inline |
| SupplySnowcover(void) | Landscape | inline |
| SupplySnowcover(long a\_date) | Landscape | inline |
| SupplyTemp(void) | Landscape | inline |
| SupplyTemp(long a\_date) | Landscape | inline |
| SupplyTempPeriod(long a\_date, int a\_period) | Landscape | inline |
| SupplyTrafficLoad(int a\_x, int a\_y) | Landscape | inline |
| SupplyTrafficLoad(int a\_polyref) | Landscape | inline |
| SupplyTreeAge(int a\_Polyref) | Landscape | inline |
| SupplyTreeAge(int, int) | Landscape | inline |
| SupplyTreeHeight(int, int) | Landscape | inline |
| SupplyTreeHeight(int) | Landscape | inline |
| SupplyUnderGrowthWidth(int, int) | Landscape | inline |
| SupplyUnderGrowthWidth(int) | Landscape | inline |
| SupplyValidX(int a\_polyref) | Landscape | inline |
| SupplyValidY(int a\_polyref) | Landscape | inline |
| SupplyVegAge(int a\_Polyref) | Landscape | inline |
| SupplyVegAge(int a\_x, int a\_y) | Landscape | inline |
| SupplyVegBiomass(int a\_polyref) | Landscape | inline |
| SupplyVegBiomass(int a\_x, int a\_y) | Landscape | inline |
| SupplyVegBiomassVector(unsigned int a\_index) | Landscape | inline |
| SupplyVegCover(int a\_polyref) | Landscape | inline |
| SupplyVegCover(int a\_x, int a\_y) | Landscape | inline |
| SupplyVegCoverVector(unsigned int a\_index) | Landscape | inline |
| SupplyVegDensity(int a\_polyref) | Landscape | inline |
| SupplyVegDensity(int a\_x, int a\_y) | Landscape | inline |
| SupplyVegDigestability(int a\_polyref) | Landscape | inline |
| SupplyVegDigestability(int a\_x, int a\_y) | Landscape | inline |
| SupplyVegDigestabilityVector(unsigned int a\_index) | Landscape | inline |
| SupplyVegHeight(int a\_polyref) | Landscape | inline |
| SupplyVegHeight(int a\_x, int a\_y) | Landscape | inline |
| SupplyVegHeightVector(unsigned int a\_index) | Landscape | inline |
| SupplyVegPatchy(int a\_polyref) | Landscape | inline |
| SupplyVegPatchy(int a\_x, int a\_y) | Landscape | inline |
| SupplyVegType(int a\_x, int a\_y) | Landscape | inline |
| SupplyVegType(int polyref) | Landscape | inline |
| SupplyVegTypeVector(unsigned int a\_index) | Landscape | inline |
| SupplyVersion(void) | Landscape | inline |
| SupplyWeedBiomass(int a\_polyref) | Landscape | inline |
| SupplyWeedBiomass(int a\_x, int a\_y) | Landscape | inline |
| SupplyWind(void) | Landscape | inline |
| SupplyWind(long a\_date) | Landscape | inline |
| SupplyWindDirection(void) | Landscape | inline |
| SupplyWindPeriod(long a\_date, int a\_period) | Landscape | inline |
| SupplyYear(void) | Landscape | inline |
| SupplyYearNumber(void) | Landscape | inline |
| TestCropManagement(void) | Landscape | protected |
| Tick(void) | Landscape |  |
| TickHour(void) | Landscape | inline |
| TickMinute(void) | Landscape | inline |
| TranslateEleTypes(int EleReference) | Landscape | inline |
| TranslateVegTypes(int VegReference) | Landscape | inline |
| TurnTheWorld(void) | Landscape | inline |
| UMarginTest(int a\_fieldpoly, int a\_borderpoly, int a\_x, int a\_y, int a\_width) | Landscape | protected |
| UnsprayedMarginAdd(LE \*a\_field) | Landscape | protected |
| UnsprayedMarginScan(LE \*a\_field, int a\_width) | Landscape | protected |
| VegDump(int x, int y) | Landscape | protected |
| VegtypeToString(TTypesOfVegetation a\_veg) | Landscape |  |
| Warn(const char \*a\_msg1, const char \*a\_msg2) | Landscape | inline |
| ~Landscape(void) | Landscape |  |


- Generated on Thu Jan 10 2013 13:15:36 for ALMaSS Skylark ODdox by
   1.8.1.1
